# Investigating causal relationships between obesity and skin barrier function in a multi-ethnic Asian general population cohort

**DOI:** 10.1038/s41366-023-01343-z

**Published:** 2023-07-21

**Authors:** Yik Weng Yew, Theresia Mina, Hong Kiat Ng, Benjamin Chih Chiang Lam, Elio Riboli, Eng Sing Lee, Jimmy Lee, Joanne Ngeow, Paul Elliott, Steven Tien Guan Thng, John C. Chambers, Marie Loh

**Affiliations:** 1 National Skin Centre, Singapore, 308205 Singapore; 2 Lee Kong Chian School of Medicine, Nanyang Technological University, Clinical Sciences Building, Singapore, 308232 Singapore; 3 Khoo Teck Puat Hospital, Integrated Care for Obesity & Diabetes, Singapore, 768828 Singapore; 4 Department of Epidemiology and Biostatistics, School of Public Health, Imperial College London, St Mary’s Campus, London, W2 1NY United Kingdom; 5 Clinical Research Unit, National Healthcare Group Polyclinic, Nexus@one-north, Singapore, 138543 Singapore; 6 Research Division, Institute of Mental Health, Singapore, 539747 Singapore; 7 Division of Medical Oncology, National Cancer Centre, Singapore, 169610 Singapore; 8 Genome Institute of Singapore (GIS), Agency for Science, Technology and Research (A*STAR), Singapore, 138672 Singapore

**Keywords:** Physiology, Genetics, Risk factors

## Abstract

**Background:**

Skin diseases impact significantly on the quality of life and psychology of patients. Obesity has been observed as a risk factor for skin diseases. Skin epidermal barrier dysfunctions are typical manifestations across several dermatological disturbances.

**Objectives:**

We aim to establish the association between obesity and skin physiology measurements and investigate whether obesity may play a possible causal role on skin barrier dysfunction.

**Methods:**

We investigated the relationship of obesity with skin physiology measurements, namely transepidermal water loss (TEWL), skin surface moisture and skin pH in an Asian population cohort (*n* = 9990). To assess for a possible causal association between body mass index (BMI) and skin physiology measurements, we performed Mendelian Randomization (MR), along with subsequent additional analyses to assess the potential causal impact of known socioeconomic and comorbidities of obesity on TEWL.

**Results:**

Every 1 kg/m2 increase in BMI was associated with a 0.221% (95%CI: 0.144–0.298) increase in TEWL (*P* = 2.82E–08), a 0.336% (95%CI: 0.148–0.524) decrease in skin moisture (*P* = 4.66E–04) and a 0.184% (95%CI: 0.144–0.224) decrease in pH (*P* = 1.36E–19), adjusting for age, gender, and ethnicity. Relationships for both TEWL and pH with BMI remained strong (Beta 0.354; 95%CI: 0.189–0.520 and Beta –0.170; 95%CI: –0.253 to –0.087, respectively) even after adjusting for known confounders, with MR experiments further supporting BMI’s possible causal relationship with TEWL. Based on additional MR performed, none of the socioeconomic and comorbidities of obesity investigated are likely to have possible causal relationships with TEWL.

**Conclusion:**

We establish strong association of BMI with TEWL and skin pH, with MR results suggestive of a possible causal relationship of obesity with TEWL. It emphasizes the potential impact of obesity on skin barrier function and therefore opportunity for primary prevention.

## Introduction

Skin and related subcutaneous diseases are a major public health problem, responsible for 42.9 million disability-adjusted life years (DALYs) globally [1]. Beyond symptoms of itch and pain, skin diseases can also be physically disfiguring. Therefore, while mortality rate is low, individuals with skin diseases often suffer from impaired quality of life and may be affected psychologically [2, 3].

Obesity, defined as an abnormal or excessive accumulation of body fat, is recognized to be an important risk factor for a range of systemic diseases, including hypertension, diabetes, cancer and autoimmune disorders [4–6]. More recently, obesity has also been identified as a risk factor for skin diseases including psoriasis and atopic dermatitis (AD) [7, 8]. The associations of obesity with psoriasis and AD have been reported in both observational cohort studies and meta-analyses, with Mendelian Randomization (MR) experiments suggesting that the effect of increased adiposity on the development of both psoriasis and AD to likely be causal [7, 9, 10].

Skin epidermal barrier dysfunction is a typical finding across dermatological disturbances [11]. Disrupted skin barrier function is characterized by an increase in transepidermal water loss (TEWL), lower moisture content of skin surface corneocytes and a higher skin pH [12]. Experimental and human studies suggest that obesity may affect skin barrier functions [13–15]. In this study, we therefore set out to establish the association between obesity and skin physiology measurements in a population cohort of close to 10,000 individuals, and to investigate whether there is evidence supporting a possible causal association between obesity and skin barrier dysfunction via MR approaches [16].

## Methods

### Study population

We investigated the relationship of adiposity and skin barrier functions amongst 10,183 men and women of Asian ancestry, participating in the Health For Life in Singapore (HELIOS) study. The HELIOS study recruits Singaporean citizens and permanent residents aged 30–84 years old from the general population. Study participants undergo a series of self-administered electronic questionnaire surveys on demographic and medical information as well as measurements across various system domains, including anthropometric and skin physiology measurements (Supplementary Methods).

### Skin physiology measurements

Skin physiology was measured as TEWL, skin surface moisture and skin pH. TEWL measures the amount of water lost from within the skin to the external atmosphere, while skin surface moisture content refers to hydration values at the stratum corneum level. Skin surface pH refers to apparent skin surface pH, with high skin pH generally reflective of a less healthy skin barrier function. Measurements were made with a vapometer, MoistureMeter SC and a pH meter over the left ventral forearm area. (Supplementary Methods) As it has been reported that multiple measurements of several skin barrier function parameters could interfere with each other, it was further stated in our protocol that TEWL should always be measured first, followed by skin moisture and pH, in line with previous recommendation [17].

### Association analysis of adiposity and skin physiological measurements in population cohort

We excluded participants who were not of the three major ethnic groups in Singapore (*n* = 127). Skin physiological measurements for TEWL, skin moisture and pH were missing in 68, 77 and 289 participants respectively. Descriptive analysis was first performed to evaluate the demographic characteristic of the study participants.

Linear regression analyses were performed with log transformed skin physiology measurements as dependent variables and BMI as independent variable, adjusted by age, gender and ethnicity. We also performed additional analyses, stratified by ethnicity to assess whether the observed associations were ethnic-specific. Other known confounders affecting the relationship between adiposity and skin physiology were evaluated and included in the multiple regression models. (Supplementary Methods)

All statistical analysis was performed using the Statistical Package for the Social Sciences (v25; IBM SPSS, Armonk, NY, USA).

### Genotyping and genetic association with BMI and skin physiological measurements

Genotyping was carried out from a combination of low coverage (15x) sequencing and imputation [18]. Paired end 151 bp whole genome sequencing was performed on the Illumina HiSeq X with an average sequencing depth of 15.8X per sample (*n* = 2400) as detailed in the Supplementary Methods.

The TopMed Imputation Server was used to impute autosomal SNPs to the TopMed (Version R2) reference panel using the EAGLE2+Minimac4 pre-phasing and imputation pipeline (Supplementary Methods) [19]. A total of 7,150,557 SNPs were used for downstream analysis. Linear regression association analyses were carried out with BOLT-LMM v2.3.5 [20]. The BOLT-LMM algorithm has been reported to be robust to potential confounding, due to relatedness or population structure [21]. Age and sex were included as covariates.

### Assessment of causal relationship of BMI on skin physiological measurements—Mendelian Randomization (MR)

We performed a one-sample MR analysis with multiple genetic variants as instrumental variables in our study to assess the causal relationship and its strength between BMI and skin physiological measurements. It was performed in only one direction to establish the causality of adiposity, measured via BMI, on skin barrier functions. The validity of the instrumental variables was assessed according to the three key assumptions of MR (Supplementary Methods). To further ensure the robustness of our findings, both third party one-sample and one-sample individual-level MR analyses were performed. A third party one-sample MR refers to the use of an independent third-party dataset for genetic instrument selection while the exposure and outcome datasets are derived in a single population cohort. Using such a separate and non-overlapping dataset for instrument selection will ensure unbiasedness of the genetic effect estimates and bias from the “winner’s curse” could be attenuated [22, 23]. The benefits of a one sample setting allow the evaluation to be performed in a single population sample of similar population characteristics and avoids the concerns of possible differing linkage disquilibrium in two samples MR studies [22].

MR analyses were performed using the R statistical software (RStudio version 2021.09.0, R 4.1.1). We performed third party one-sample MR using the *TwoSampleMR* library, while the one-sample individual-level MR analysis was conducted with the *ivreg* R package [24, 25]. For the third party one-sample MR, genetic risk variants for BMI were first determined using the combined analysis of the Genetic Investigation of ANthropometric Traits (GIANT) consortium and the UK biobank dataset of about 700,000 individuals with European ancestry in total (Genetic instrument selection dataset) (Supplementary Methods) [26].

After these SNPs were identified, summary level SNPs-exposure associations were first extracted from our BMI GWAS performed on HELIOS samples as MR instruments. These instruments SNPs-outcome associations were then extracted from our GWAS dataset(s) on outcome (skin physiology measurements), also in the same HELIOS samples. Any missing exposure associated variants in our HELIOS GWAS dataset were replaced by linkage disequilibrium (LD) proxies of a minimum of 0.6. 214 SNPs out of the 941 SNPs (see Supplementary Table 1) in the third-party BMI GWAS dataset had missing effect measure in the our GWAS dataset for BMI and skin physiology and therefore required proxy SNPs (see Supplementary Table 1; LD: r^2^ ≥ 0.6) for analysis. One hundred and eighty-three missing SNPs had no proxy SNPs with adequate LD r^2^ values of greater than 0.6 and were therefore excluded from the analysis (Supplementary Table 1). Extracted summary statistics are summarised in Supplementary Table 1.

In order to examine the robustness of our MR estimates, we estimated the causal effect estimate of exposure on outcome using several MR methods: inverse variance weighted method, maximum likelihood method, weighted median-based method, weighted mode based method and MR-Egger regression analysis [24, 27–29].

### Pleiotropic analysis for the assessment of potential factors between BMI and TEWL

We performed third party one-sample MR analyses with multiple genetic variants as instrumental variables in our study to assess for a possible causal relationship and its strength of potential exposures previously reported to be linked to BMI to assess its effect on TEWL. These exposures included socioeconomic factors (education level and household income), glycaemic endpoints (HOMA-IR, HbA1c and DM), blood pressure endpoints (SBP, DBP, PP and hypertension), heart rate, Vitamin D, CRP, as well total white cells and neutrophil counts (as indicators of inflammation). The genetic risk variants for the exposures were determined based upon large well-powered GWAS and/or meta-analyses [30–40]. Extracted summary statistics for these MR analyses are summarised in Supplementary Tables 2.

## Results

### Cohort characteristics

The demographic characteristics of study participants included in our analysis are summarized in Supplementary Table 3 (*n* = 9990). Mean age was 52.3 ± 11.9 (SD) years old, with 59.8% of the participants being female. The participants were 68.4% Chinese, 17.7% Indians and 12.6% Malays. Forty-five percent of the study participants had education level at undergraduate or graduate level. The prevalence of diabetes, hypertension and hyperlipidemia were at 9.2%, 20.3% and 36.4% respectively. Forty-two percent of participants had never consume alcohol, with 72.5% being non-smokers. Consistent with national statistics, we noted significant differences in BMI across the three ethnic groups, with the highest mean BMI in Malays at 28.3 kg/m^2^, followed by Indians at 27.1 kg/m^2^, and the lowest in Chinese at 23.6 kg/m^2^ (P = 6.68E–16). Diabetes prevalence was highest in Indians (14.4%), followed by Malays (12.8%) and lastly Chinese (7.2%) (*P* = 1.78E–24]) (Supplementary Table 3).

### Skin physiology parameters in adiposity

**Fig. 1:**
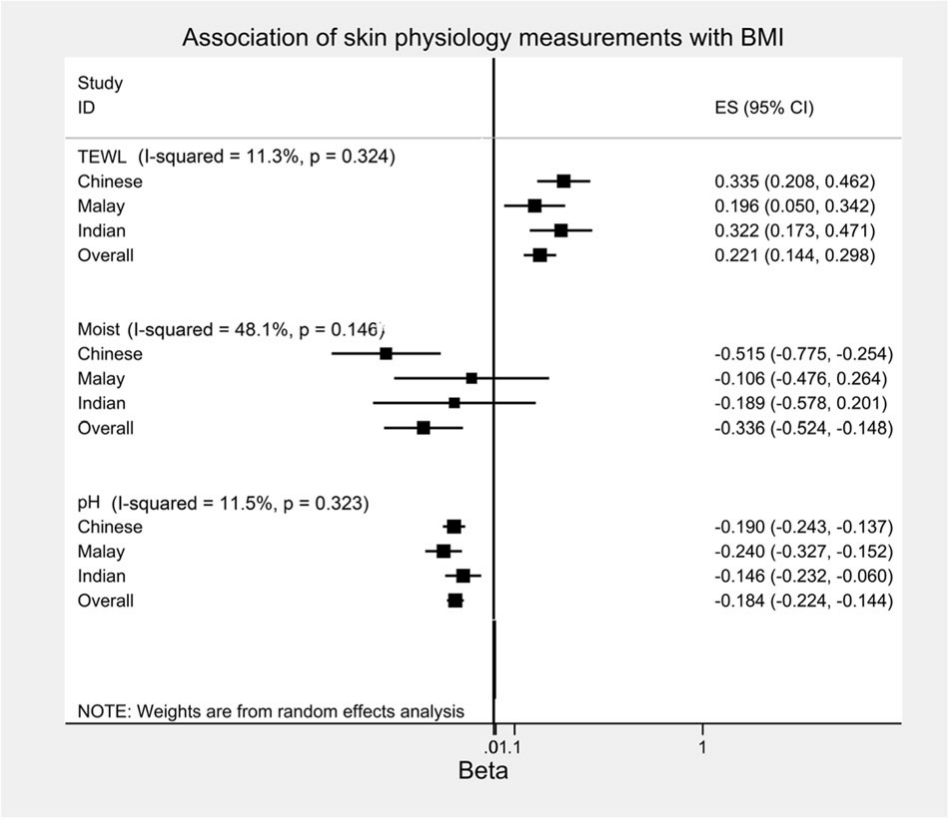
Forest plots showing the association of skin physiology measurements with Body Mass Index (BMI) stratified according to ethnic groups. The horizontal lines illustrates the 95% confidence intervals while the square represents the point estimates. The I-squared and p values represent the heterogeneity of the association across the different ethnic groups.

Mean TEWL, moisture and pH were 7.06 g/m^2^/h (SD:1.30), 27.90 units (SD:12.34) and 5.23 (SD:0.52) respectively in the entire study population (Supplementary Table 3). We first assessed the relationship of the three skin physiology measurements with BMI. TEWL and pH were strongly associated with BMI, with a 0.221% increase in TEWL (95% CI: [0.144,0.298]; *P* = 2.82E–42) and a 0.184% decrease in pH (95% CI: [–0.224,–0.144]; *P* = 1.36E–19), while a weak association with skin moisture was observed with a 0.336% decrease (95% CI: [–0.524,–0.148]; *P* = 4.66E–04) per every 1 kg/m^2^ increase in BMI, adjusted for age, gender, and ethnicity (Table 1). These associations were also consistent across all three ethnic groups with little evidence for heterogeneity (I^2^: 11.3-48.1%; *P*-het: 0.146–0.324; Fig. 1; Supplementary Table 4), suggesting that the observed associations were not ethnic-specific. We did not find any significant heterogeneity between gender groups across the skin physiological measurements (I^2^: 0-65.4%; *P*-het: 0.089-0.961).

**Table 1:** Relationship of skin physiology measurements with body mass index, BMI (kg/m^2^).

	Model 1			Model 2			Model 3		
	Beta	95% CI	*P* value	Beta	95% CI	*P* value	Beta	95% CI	*P* value
TEWL	0.221	(0.144, 0.298)	2.82E–08	0.215	(0.131,0.299)	5.20E–07	0.354	(0.189, 0.520)	2.90E–05
Moisture	–0.336	(–0.524,–0.148)	4.66E–04	–0.294	(–0.498,–0.090)	4.70E–03	–0.201	(–0.579,0.161)	2.87E–01
pH	–0.184	(–0.224,–0.144)	1.36E–19	–0.189	(–0.232,–0.146)	1.49E–17	–0.170	(–0.253,–0.087)	5.70E–05

Beta: % change in skin physiology measurements per unit (kg/m2) increase in BMI.

Model 1: adjusted for age, gender, ethnicity.

Model 2: adjusted for Model 1 + education level, household income.

Model 3: adjusted for Model 2 + smoking, atopic dermatitis, diabetes mellitus, Hba1c, Insulin Resistance (HOMA-IR), systolic blood pressure, diastolic blood pressure, heart rate, low-density lipoprotein, total white blood cell count, Vitamin D, C-reactive protein.

**Fig. 2:**
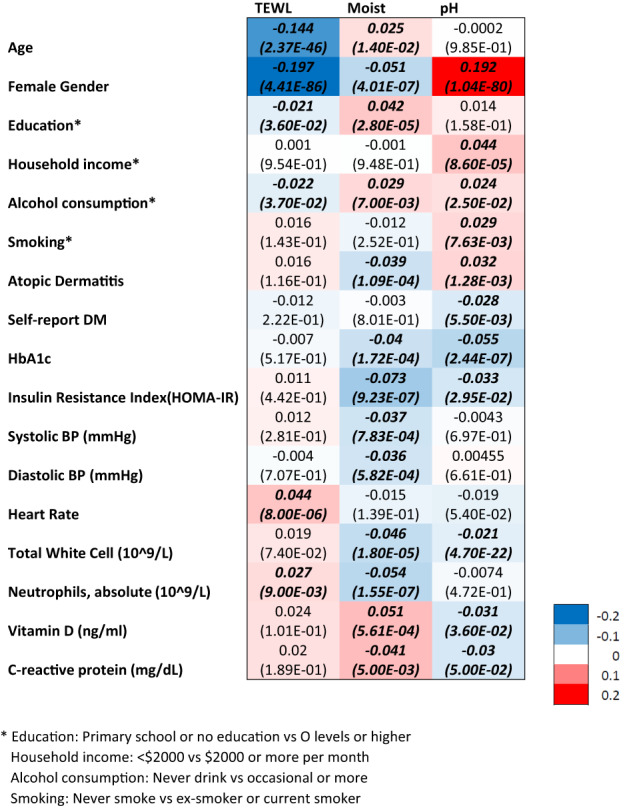
A heatmap depicting adjusted linear regression of various variables against log transformation of skin physiology measures. Standardized beta values and *p* values in brackets are reflected in the cells with italic front representing statistical significance at *p* < 0.05.

To identify potential confounders, we performed univariate analyses to assess the association of demographics characteristics, socioeconomic status, lifestyle habits and comorbidities with skin physiology measurements. Gender and alcohol consumption were significantly associated with all three skin physiological measurements. Age and socioeconomic status (household income and education level), smoking status, AD status, self-reported DM status, HOMA-IR and HbA1c, blood pressure (SBP and DBP), heart rate, total white cell and neutrophils counts, Vitamin D levels as well as CRP levels, were significantly associated with one or more of the skin physiology parameters (Fig. 2; Supplementary Table 5). Adjusting for these additional covariates did not impact the associations of TEWL and pH with BMI, with effect sizes remaining at 0.354% unit increase in TEWL (*P* = 2.90E–05) and 0.170% decrease in pH (*P* = 5.70E–05) with every 1 kg/m^2^ increase in BMI. In contrast, the association for skin moisture was attenuated and no longer significant (*P* = 2.87E–01) (Table 1).

### Mendelian randomization (MR) - BMI on TEWL

To assess for evidence for a possible causal associationand its strength between BMI and skin physiological measurements, we performed Mendelian Randomization. The MR estimate of BMI on TEWL with third-party one-sample MR using inverse variance weighted (IVW) yielded an effect size of 0.477% increase in TEWL per unit increase in BMI (kg/m^2^) (95% CI: [0.00941, 0.00861]; *P* = 0.015). The weighted median and MR Egger method yielded similar effect size of 0.0828% (95% CI: [0.0151, 0.150]; *P* = 0.016) and 0.0654% (95% CI: [0.00519, 0.125]; *P* = 0.033). MR-Egger regression analysis showed that the intercept did not significantly deviated from zero (*P* = 0.46), which suggested no horizontal pleiotropy. As part of sensitivity analyses, we repeated the MR studies removing SNPs with pleiotropic effects that might confound the relationship between BMI and TEWL (see Supplementary Methods; Supplementary Table 6). This modified IVW analysis yielded a similar effect size of 0.0483% (95% CI: [0.00380, 0.0929]; *P* = 0.033) increase in TEWL per unit increase in BMI (kg/m^2^) (Table 2).

**Table 2:** Summary results of third-party One-sample Mendelian Randomization (MR) analysis for effect of BMI on skin physiology measures.

MR method	Beta [95% CI]	*P* value
BMI on TEWL—% change in TEWL per unit (kg/m2) increase in BMI		
IVW (Random Effects)	4.77E–02 [9.41E–03, 8.61E–03]	0.015
Maximum Likelihood Method	4.79E–02 [−5.56E–02, 1.51E–01]	0.364
Weighted Median Method	8.28E–02 [1.51E–02, 1.50E–01]	0.016
Weighted Mode Method	–4.34E–02 [–2.05E–01, 2.04E–01]	0.997
MR Egger (Random Effect)	6.54E–02 [5.19E–03, 1.25E–01]	0.033
IVW (Random Effects, modified)*	4.83E–02 [3.80E–03, 9.29E–02]	0.033
Egger Intercept	–3.54E–02 [–1.29E–01, 5.79E–02]	0.458
BMI on Skin Moisture—% change in Moisture per unit (kg/m2) increase in BMI		
IVW (Random Effects)	1.37E–02 [–6.38E–02, 9.12E–02]	0.728
Maximum Likelihood Method	1.92E–02 [–2.30E–01, 2.69E–01]	0.883
Weighted Median Method	–6.68E–02 [–2.60E–01, 7.26E–02]	0.347
Weighted Mode Method	3.71E–02 [–3.09E–01, 3.10E–01]	0.998
MR Egger (Random Effect)	2.17E–02 [–9.99E–02, 1.43E–01]	0.727
IVW (Random Effects, modified)*	1.26E–02 [–7.79E–02, 1.03E–01]	0.785
Egger Intercept	–1.60E–02 [–2.05E–01, 1.73E–01]	0.868
BMI on Skin pH - % change in pH per unit (kg/m2) increase in BMI		
IVW (Random Effects)	–6.35E–03 [–2.49E–02, 1.22E–02]	0.502
Maximum Likelihood Method	–6.87E–03 [–5.58E–02,4.21E–02]	0.783
Weighted Median Method	3.74E–03 [–3.02E–02, 3.77E–02]	0.829
Weight Mode Method	–7.28E–03 [–1.83E–01, 1.83E–01]	0.999
MR Egger (Random Effect)	–1.13E–02 [–4.03E–02, 1.76E–02]	0.442
IVW (Random Effects, modified)*	4.50E–03 [–4.83E–02, 5.73E–02]	0.867
Egger Intercept	1.01E–02 [–3.49E–02, 5.51E–02]	0.659

*Excluding possible pleiotropic genetic factors in analysis.

To further evaluate the robustness of our findings, we next conducted one-sample individual level data MR to support the causal relationship of obesity on TEWL. Indeed, the MR estimate of BMI on TEWL using instrument-variable (IV) regression by two-stage least squares (2SLS), adjusting for age, gender and ethnicity, yielded an effect size of 0.374% (95% CI: [0.213], 0.535; *P* = 5.76E–06) increase in TEWL per unit increase in BMI (kg/m^2^). Additional diagnostic tests supported that the instruments were sufficiently strong (Weak Instruments F-test; *P* < 2E–16) and valid (Sargan test; *P* > 0.05).

BMI has been reported to be associated with education level and household income, as well as insulin resistance, autonomic activation and systemic inflammation. To assess the effect of these potential exposures on TEWL, we additionally performed Mendelian Randomization of the following variables: number of years of education completed, monthly household income, glycaemic endpoints (HOMA-IR, HbA1c and DM), blood pressure endpoints (SBP, DBP, PP and hypertension), heart rate, CRP, total white cells and neutrophil counts (as indicators of inflammation), Vitamin D and AD status. However, none of the MR estimates of the exposure on TEWL reached statistical significance (Table 3).

**Table 3:** Summary results of third-party one-sample Mendelian randomization (inverse variance weighted method) for effect of potential exposures on TEWL.

MR method	Beta [95% CI]	*P* value
Education (Number of years education completed): % change in TEWL per unit (years) increase in education	–3.93E–01 [–8.25E–01, 3.80E–02]	0.074
Monthly household income: % change in TEWL per unit (income tiers) increase in monthly household income	2.53E–01 [–2.79, 3.29]	0.871
Diabetes mellitus (DM): % change in TEWL between those with DM and those without	–3.02E–01 [–8.50, 7.98]	0.942
HbA1c: % change in TEWL per unit (%) increase in HbA1c	1.58 [–8.00E–01, 3.96]	0.194
Insulin Resistance (HOMA-IR): % change in TEWL per unit (%) increase in HOMA-IR	–3.23 [–9.52, 3.07]	0.315
Systolic BP (SBP): % change in TEWL per unit (mmHg) increase in SBP	8.68E–02 [–5.27E–02, 2.26E–01]	0.223
Diastolic BP (DBP): % change in TEWL per unit (mmHg) increase in DBP	4.73E–01 [–1.20E-01, 1.07]	0.118
Pulse Pressure (PP): % change in TEWL per unit (mmHg) increase in PP	9.60E–02 [–1.36E–01, 3.28E–01]	0.418
Hypertension: % change in TEWL between those with and without hypertension	–1.34E–02 [–3.64E–01, 3.37E–01]	0.940
Heart rate: % change in TEWL per unit increase in Heart rate	1.69E–01 [–3.19E–01, 6.7E–01]	0.496
C-reactive protein (CRP): % change in TEWL per unit (mg/dL) increase in CRP	–1.12E–01 [–7.24E–01, 4.99E–01]	0.720
Total White Count: % change in TEWL per unit (x10^9^) increase in Total White Count	5.23E–01 [–8.24E–01, 1.87]	0.446
Neutrophil Count: % change in TEWL per unit (x10^9^) increase in Neutrophil Count	6.91E–01 [–1.39, 2.77]	0.515
Vitamin D: % change in TEWL per unit (ng/ml) increase in Vitamin D	2.79E–02 [–1.36E–01, 1.92E–01]	0.738
Atopic dermatitis (AD): % change in TEWL between those with AD and those without	1.98E–01 [–2.17E–03, 6.13]	0.349

### Mendelian randomization (MR) - BMI on skin moisture and pH

MR of BMI on skin moisture and skin pH with third-party one-sample MR did not reach statistical significance across the different MR methods used (Table 2). MR analyses after removal of SNPs with pleiotropic effects (modified IVW) as part of sensitivity analyses remained not significant (*P* = 0.728 and 0.502 respectively).

## Discussion

Obesity has been identified as a risk factor for common skin diseases such as psoriasis and AD across both observational cohort studies and meta-analyses, with MR experiments suggestive of a possible causal association of increased adiposity on the development of these skin diseases [7–10]. Skin epidermal barrier dysfunction, which can be objectively assessed via TEWL, skin surface moisture and pH, is often observed in these dermatological conditions [11, 12]. In this study, we carried out epidemiological analysis to investigate the relationship between adiposity and skin barrier function in a large representative Asian population cohort, accompanied by Mendelian Randomization to investigate whether obesity is likely to have a possible causal relationship with skin barrier dysfunction.

We observed that increase in BMI was consistently associated with increased TEWL, which is in agreement with previous reports from smaller sample series [13–15]. The increase in BMI was also observed with a more acidic skin pH, with the relationships for both TEWL and pH with BMI remaining strong even after adjusting for known confounders. Using MR, we were then further able to demonstrate a causal relationship of BMI on TEWL, which is consistent with earlier studies demonstrating the likely causal effect of BMI on dermatological conditions such as AD [10]. The MR analysis did not demonstrate a causal relationship of BMI on pH. Our study therefore builds on current knowledge of association between obesity and skin diseases, demonstrating strong association between obesity and skin physiological measures in a large Asian population cohort, as well as initial MR evidence for a possible causal relationship between BMI and TEWL. Additional MRs performed on known confounders of obesity on TEWL also adds to our understanding of underlying of adiposity on skin barrier dysfunction.

To further our understanding of the underlying mechanisms leading from obesity to changes in skin physiology, we additionally assessed the relationships of known demographics and socioeconomic characteristics, as well as comorbidities of obesity on skin physiology measurements, and investigated their potential causal relationship with skin epidermal barrier dysfunction. We confirmed previously reported associations of skin physiology measures with female gender and age [41–43]. Higher socioeconomic status were observed to be significantly associated with lower TEWL and higher skin moisture [44–48]. Current smoking was also noted with drier skin, which is likely accounted for by nicotine use, leading to dry skin via impairment of blood flow due to vessel constriction [49, 50]. Presence of known comorbidities such as presence of diabetes and hypertension, as well as higher heart rate and inflammatory markers were consistently associated with higher TEWL, lower skin moisture and lower pH. However, from our MR experiments, we observed that the relationships of these known comorbidities of BMI with TEWL are unlikely to be causal, suggesting that they are more likely common consequences on the same pathways.

Indeed, the relationship between obesity and skin physiology is complex. The pro-inflammatory state of obesity is widely agreed upon, and adipose tissue is likely to contribute to chronic persistent low-grade systemic inflammation by the production of inflammatory cytokines such as IL6 [51]. Excessive skin fat tissue expansion may also impair the barrier function of the skin epidermis, with mechanical stretching of the skin due to obesity potentially contributing to skin inflammation by impairing the epidermal barrier function and pre-deposition of keratinocytes under activation state [52, 53].

The Impact of obesity has been demonstrated, at least in part, in animal studies as well. Mice on high-fat diet (HFD) were reported to be not only have severe obesity, but also had TEWL that was 33% higher relative to the controls [54]. The higher TEWL was indicative of impaired barrier function in the HFD mice, which was suggested to be attributable to structural fragility, abnormal glycerol transport, and dysregulated proliferation of epidermal cells. This was further accompanied by an increase in serum levels of inflammatory cytokines in the HFD-fed mice [54]. Although we too observed positive association of CRP, total white cells and neutrophil counts with TEWL in our cohort, this was not found to lie on the causal pathway from obesity to TEWL via our MR experiments. Nonetheless, this does not rule out the role of other unmeasured inflammatory markers. As an example, in the case of atherosclerosis, CRP was suggested to be not directly causal to the development of atherosclerosis, but rather marks the presence of atheroma or other pro-atherogenic exposures, as well as unmeasured risk factors [55].

Chronic sympathetic overactivity has been shown to be consistently present in obesity, especially in central adiposity. Indeed, obesity has being suggested as the cause of sympathetic nervous activation [56, 57]. Apart from the links of an elevated sympathetic outflow to organs such as the heart, kidneys and blood vessels, autonomic dysfunction is also linked to skin dryness. Specifically, it was demonstrated that unmyelinated fibers, which provide the sudomotor activity, part of sympathetic activity, and activation of sweat glands, are affected in patients with AD, hence supporting the hypothesis that sympathetic activity dysfunction contributes to skin dryness [58]. This further substantiates the possibility that known comorbidities of BMI may not lie on the causal pathway on TEWL, but are likely common consequences.

Compared to the relationship of BMI and TEWL, the understanding and evidence explaining the pathophysiological mechanisms between obesity and skin pH is considerably less common and more conflicting. It has been reported previously that individuals with obesity tend to have a more alkaline skin pH, especially over the intertriginous areas [59, 60]. The increased in sweating together with occlusion might increase skin humidity which in turn elevates skin surface pH [59]. The increased heat and moisture could also possibly lead to a dysregulated skin microbiome leading to a change in skin pH. However, we have consistently observed in our study that obesity was associated with a more acidic skin pH measured over the ventral forearms. This have previously been postulated to be associated with changes in skin lipids or fatty acids profile and increased inflammatory markers in the stratum corneum of individuals with obesity [61, 62]. The conflicting observations might therefore be secondary to local environmental factors such as humidity, overlying microbiome colonization and other local factors. This is also supported by our observation that genetically increased risk of obesity has no significant causal association with a change in skin pH, possibly suggesting a more predominant environmental influence.

Admittedly, there are some limitations with our study. One limitation of our study was that all of our study participants were of Asian ancestry, which may limit potential generalization to other populations such as Europeans. However, it is unlikely that causal mechanisms between adiposity and skin physiology will differ between ethnic populations. We also acknowledged that our MR experiments were underpowered due to the smaller sample sizes of our skin physiology GWAS.

However, despite the limitations, our study comes with notable strengths. Our study was conducted in a large series recruited from the general population. The choice of a one-sample MR approach also allowed us to be more confident that the genetic markers used in our analysis are independent of known confounding variables, as it is not reliant on the assumption of ancestral homogeneity in two-sample MR. As bias from weak instruments will be in the direction of the observational association (i.e. false positive), we have carefully checked for weak instruments as well as their validity, and confirmed that the instruments were strong enough and valid.

In summary, our study provides clear evidence that there exists a strong relationship between skin barrier dysfunction, specifically TEWL and skin pH, with adiposity measurements in an Asian population. We were also able to demonstrate possible evidence for a causal relationship of BMI with TEWL. Although we were not able to decipher the exact mechanisms yet via our MR experiments, our study has highlighted the importance of enhancing our understanding of underlying etiology and pathways of adiposity on skin barrier dysfunction, as this could underlie the risk of dermatological conditions including but not limited to psoriasis and AD. More generally, this study serves to further emphasize the importance of confronting the obesity epidemic, as increased adiposity not only underlie metabolic diseases such as diabetes mellitus and heart diseases, but could also impact on other phenotypes beyond, such as dermatological conditions.

### Supplementary information

Supplementary Methods

Supplementary Table 1

Supplementary Table 2

Supplementary Table 3

Supplementary Table 4

Supplementary Table 5

Supplementary Table 6

Supplementary Table 7

## Data Availability

Any data access request proposals should be directed to helios_science@ntu.edu.sg for the consideration of the HELIOS Study’s principal investigators.
