# Supplementary material for: Investigating causal relationships between obesity and skin barrier function in a multi-ethnic Asian general population cohort
Source: Int J Obes (Lond). 2023 Jul 21;47(10):963–9. doi: 10.1038/s41366-023-01343-z (PMC10511308; doi:10.1038/s41366-023-01343-z)
Supplement: Supplementary file 1 — Supplementary Methods [file 41366_2023_1343_MOESM1_ESM.docx]

**Supplementary Methods**

Study population

Study participants were from the Health For Life in Singapore (HELIOS) study. The study started in 2018, and we included all participants through to 31st January 2022. Multiple community engagement strategies are employed to ensure representative participation across ethnic populations, age group and socio-economic groups. People who are pregnant, breastfeeding, acutely ill or unable to give informed consent are excluded.

Participants’ ethnicity were recorded according to the Singapore Registry of Citizens. Chinese, Malays and Indians form the three primary ethnic groups in Singapore, which accounts for 76%, 15% and 8% of the population and corresponds to East Asian, South-east Asian and South Asian respectively.^1, 2^ Height and weight were measured using the BSM 370 automatic stadiometer (Inbody, Seoul, South Korea), using computerized measuring instruments with automated data capture. Body mass index (BMI) was calculated by dividing weight in kilograms by the square of height in meters. Educational level, household income, lifestyle and medical history data were collected via self-administered questionnaires. Resting blood pressure was measured three times using an automated device meeting international standard of blood pressure monitoring and was averaged. Total cholesterol, hemoglobin A1c (HbA1c), C-reactive protein (CRP), total white count and neutrophil counts were measured from fasting blood samples by the accredited laboratory (QuestLab, Singapore, SAC–SINGLAS ISO 15189:2012) using ADVIA 1800 chemistry system (Siemens Healthcare, Munich, Germany). Vitamin D level was measured on ADVIA Centaur XPT immunoassay system (Siemens Healthcare, Munich, Germany). Disease status was determined using the clinical measurement, self-reported status of chronic diseases, and the presence of relevant medication. The information on participants’ medication were obtained through a structured nurse interview.

Skin physiology measurements

Skin physiology measurements were made with a vapometer (SWL5001; Delfin Technologies, Finland), MoistureMeter SC (MSC1001; Delfin Technologies, Finland) and a pH meter (LAQUA-PH220; HORIBA Scientific, Japan) over the left ventral forearm area. at the intersection of midpoint between elbow crease and ventral wrist crease and the midpoint of the width of the forearm, over an area free of visible skin rash, unless contraindicated. The ventral forearm area was selected as it is deemed to be relatively sheltered from environmental factors such as draft and changes in humidity and therefore less susceptible to external factors. It is also relatively sheltered from the sun compared to the dorsal surfaces. This is also considered as representative of dry site of human skin and have relatively fewer sweat glands compared to moist surfaces which can affect measurements of skin physiology.^3^ Measurements were taken three times in the same room with a pre-set temperature and humidity setting of 23.0 degree Celsius and 60% respectively to reduce environmental variation.

Study participants were acclimatised for at least 20min before the measurement. Mean values of the three repeated measurements were presented. Skin physiology measurements below the 0.5^th^ percentile or above the 99.5^th^ percentile were deemed outliers and excluded from subsequent analysis. Devices are validated, and calibrated regularly according to manufacturer instructions.^4-6^ We also adjusted the skin physiology measurements via cubic smoothing spline using the *smooth.spline* function in R (version 4.1.1) with a smoothing parameter of 0.5 for any possible drift. These adjusted measurements were then used for all downstream analyses.

Association analysis of adiposity and skin physiological measurements

Skin physiology measures were assessed for normality and found to have skewed data distribution via Shapiro-Wilk test and Normal Q-Q plots. Log transformation was performed to transform these skin physiology values to approximately conform to normality before further analysis.

As BMI has been linked to education level and household income, as well as insulin resistance, autonomic activation and systemic inflammation, we additionally adjusted for these known confounders. We included education level, household income, other potential lifestyle and comorbidities confounders such as smoking, AD, diabetes mellitus and hypertension status, as well as glycaemic measurements (Homeostatic Model Assessment for Insulin Resistance (HOMA-IR) and HbA1c), blood pressure measurements (systolic blood pressure (SBP), diastolic blood pressure (DBP) and pulse pressure (PP)), heart rate, Vitamin D, C-reactive protein (CRP) level and total white cell and neutrophils. All regression models were age, gender and ethnicity adjusted. In multiple regression models, we checked for multicollinearity via variance inflation factor (VIF) and only retained independent variables, as defined by VIF<5.

Whole genome sequencing and imputation

Paired end 151 bp whole genome sequencing was performed on the Illumina HiSeq X with an average sequencing depth of 15.8X per sample (n=2,400). The paired-end reads were adaptor-trimmed using Trimadap and mapped using BWA mem onto the GATK resource bundle distributed human genome build GRCh38. Duplicated reads were identified and discarded using Picard MarkDuplicates with GATK "germline short variant per-sample calling" Reference Implementation defined parameters. Base quality scores were recalibrated using GATK BaseRecalibrator/ApplyBQSR on a per library basis. The resulting single-sample BAM file was converted to loseless CRAM format and indexed for later use in alignment-level QC and variant calling steps.

Pre-imputation checks were performed using the Rayner toolbox, which ensured all alleles were on the forward strand, coordinates are on the GRCh38 assembly and reference alleles are consistent with the reference panel.^7^ SNPs with allele frequency differences >0.20 between the TopMed reference panel and our dataset, palindromic SNPs with allele frequency >0.4 and genotyped SNPs that are not in the TopMed reference panel were excluded using PLINK 1.902. The Robust Unified Test for Hardy-Weinberg equilibrium (RUTH) method was applied to the raw variant call format (VCF) files for each chromosome in all individuals in order to identify SNPs that fail Hardy-Weinberg equilibrium (HWE) while accounting for population structure.^8^ HWE test statistics were generated using the genotype likelihoods and the top four genotypic principal components to account for population structure, with HWE P-values calculated using the likelihood ratio test. SNPs with RUTH HWE test P<10E-6 were excluded.

Additional pre-imputation quality control excluded autosomal genotyped SNPs with MAF <0.01 and SNP missingness call rate >5% within the three main ancestral groups (Chinese, Malays and Indians) using PLINK 1.902, with SNPs at the intersection brought forward for imputation.^9, 10^ A total of 3,245,000 autosomal SNPs survived quality control and were used as input for imputation. Approximately 285 million autosomal SNPs were available following imputation. Post-imputation quality control excluded imputed SNPs with MAF <0.001 in at least one of the three main ancestral groups (Chinese, Indians and Malays), as well SNPs with imputation INFO score <0.30 and RUTH HWE test P <10-3. A total of 7,857,631 imputed autosomal SNPs formed the final dataset. For the purpose of the GWAS, we applied a MAF filter of 0.01. This rendered 7,150,557 SNPs remaining for downstream analysis.

Mendelian Randomization analysis

We opted to perform one-sample MR analysis with multiple genetic variants as instrumental variables in our study to assess the causal relationship and its strength between BMI and skin physiological measurements. Although two-sample MR is often chosen for its increased statistical power due to the availability of summary statistics from larger GWAS studies and/or meta-analyses, it however assumes that the exposure and outcome datasets are ancestrally homogeneous.^11^ Given the genetic diversity of our study population, we have therefore decided to perform a one-sample MR in the current study.^12^ This allows us to be confident that genetic markers used in our analysis are independent of known confounding variables.

The validity of the instrumental variables for the MR analysis are defined by three key assumptions: 1) genetic variants are associated with the exposure factor of interest (Relevance assumption); 2) genetic variants are independently associated with the outcome with no unmeasured confounders (Independence assumption); 3) genetic variants only affect the outcome through their effect on the exposure factors with no evidence of other horizontal pleiotropic factors (Exclusion restriction assumption).^13^

The validity of the instrumental variables are important for the MR analysis and are defined by three key assumptions: 1) genetic variants are associated with the exposure factor of interest (Relevance assumption); 2) genetic variants are independently associated with the outcome with no unmeasured confounders (Independence assumption); 3) genetic variants only affect the outcome through their effect on the exposure factors with no evidence of other horizontal pleiotropic factors (Exclusion restriction assumption).^13^ We carried out sensitivity analyses by excluding SNPs that have associations with possible confounders in the MR analyses, and performed an MR-Egger test of pleiotropy, to assess the validity of the independence and exclusion restriction assumptions respectively.^14^

The GIANT consortium is a joint GWAS and metabochip meta-analysis of 114 studies that measured BMI as its phenotype of interest. Meta-analysis of summary statistics from the two studies identified 941 near-independent single nucleotide polymorphisms (SNPs) associated with BMI at a revised genome-wide significance threshold of P <1×10^−8^ (“BMI SNPs”). These SNPs were identified using an approximate conditional and joint multiple-SNP (COJO) analysis that takes into account LD (linkage disequilibrium) between SNPs at a given locus.

References

1. Singapore DoS. *Singapore Census of Population 2020*. 2021 [cited 2022 05 Jan]; Available from: <https://www.singstat.gov.sg/publications/reference/cop2020/cop2020-sr1>

2. Saw WY, Tantoso E, Begum H, et al. Establishing multiple omics baselines for three Southeast Asian populations in the Singapore Integrative Omics Study. *Nat Commun* 2017; **8**: 653.

3. Byrd AL, Belkaid Y, Segre JA. The human skin microbiome. *Nat Rev Microbiol* 2018; **16**: 143-55.

4. De Paepe K, Houben E, Adam R, Wiesemann F, Rogiers V. Validation of the VapoMeter, a closed unventilated chamber system to assess transepidermal water loss vs. the open chamber Tewameter. *Skin Res Technol* 2005; **11**: 61-9.

5. Alanen E, Nuutinen J, Nicklen K, Lahtinen T, Monkkonen J. Measurement of hydration in the stratum corneum with the MoistureMeter and comparison with the Corneometer. *Skin Res Technol* 2004; **10**: 32-7.

6. Stefaniak AB, Plessis J, John SM, et al. International guidelines for the in vivo assessment of skin properties in non-clinical settings: part 1. pH. *Skin Res Technol* 2013; **19**: 59-68.

7. Group M. *Rayner toolbox*. [cited 2022 11 Aug]; Available from: <https://www.well.ox.ac.uk/~wrayner/tools/>

8. Kwong AM, Blackwell TW, LeFaive J, et al. Robust, flexible, and scalable tests for Hardy-Weinberg equilibrium across diverse ancestries. *Genetics* 2021; **218**.

9. Purcell S, Neale B, Todd-Brown K, et al. PLINK: a tool set for whole-genome association and population-based linkage analyses. *Am J Hum Genet* 2007; **81**: 559-75.

10. Purcell S. *PLINK 1.902*. [cited 2022 13 May]; Available from: <http://pngu.mgh.harvard.edu/purcell/plink/>

11. Burgess S, Scott RA, Timpson NJ, Davey Smith G, Thompson SG, Consortium E-I. Using published data in Mendelian randomization: a blueprint for efficient identification of causal risk factors. *Eur J Epidemiol* 2015; **30**: 543-52.

12. Davey Smith G, Hemani G. Mendelian randomization: genetic anchors for causal inference in epidemiological studies. *Hum Mol Genet* 2014; **23**: R89-98.

13. Davies NM, Holmes MV, Davey Smith G. Reading Mendelian randomisation studies: a guide, glossary, and checklist for clinicians. *BMJ* 2018; **362**: k601.

14. Bowden J, Davey Smith G, Burgess S. Mendelian randomization with invalid instruments: effect estimation and bias detection through Egger regression. *Int J Epidemiol* 2015; **44**: 512-25.
