# Supplementary material for: Investigating causal relationships between obesity and skin barrier function in a multi-ethnic Asian general population cohort
Source: Int J Obes (Lond). 2023 Jul 21;47(10):963–9. doi: 10.1038/s41366-023-01343-z (PMC10511308; doi:10.1038/s41366-023-01343-z)
Supplement: Supplementary file 2 — Supplementary Table 1 [file 41366_2023_1343_MOESM2_ESM.pdf]

Supplementary Table 1. List of SNPs for body mass index (BMI) and their effect on logTEWL

List of BMI SNPs from GIANT Consortium 2018 Locke et al + UKBiobank									Effects of BMI SNPs on skin physio (using current dataset)														
SNP	Sample Size	Chromosome	Position	Effect Allele	Other Allele	Missing SNPs	Proxy SNPs	LD(r2)	BMI_SNPs_a	Effect Allele	Other Allele	Beta_BMI	seBeta_BMI	p-value_BMI	Beta_logTEWL	seBeta_logTE	p-value_logTI	Beta_logmois	seBeta_logn	p-value_logmc	Beta_logpH	seBeta_logpH	p-value_lo
rs1000096	656860	4	38692835	C	T				rs1000096	T	C	0.5696	1.4459	0.6900	0.0126	0.0082	0.1300	-0.0068038	0.0164843	0.68	-6.2933E-05	0.0039886	0.99
rs1000940	794558	17	5283252	G	A				rs1000940	A	G	0.1759	1.2276	0.8900	-0.0163	0.0070	0.0200	-0.0068894	0.0140228	0.62	-0.00048977	0.00339697	0.89
rs1003081	788712	11	118913993	T	C				rs1003081	C	T	0.3345	1.3722	0.8100	-0.0050	0.0079	0.5300	0.0134442	0.0156923	0.39	0.00185379	0.00378799	0.62
rs1003528	689769	5	153095918	A	G				rs10035289	A	G	1.5873	2.1636	0.4600	0.0102	0.0123	0.4100	-0.0414231	0.0245718	0.092	-0.00056436	0.00593454	0.92
rs1003704	680227	5	88219964	G	A	*																	
rs1006353	790767	13	28047269	A	G	*																	
rs1006683	662130	5	151254297	T	C				rs10066835	T	C	2.2336	4.6377	0.6300	-0.0027	0.0266	0.9200	-0.0502481	0.053115	0.34	0.000610012	0.0131692	0.96
rs1008380	690845	16	6701400	C	T				rs10083803	C	T	0.4741	1.3555	0.7300	-0.0075	0.0077	0.3300	-0.0025651	0.0155114	0.87	0.000182971	0.00374674	0.96
rs1011072	551363	8	14324437	A	G	*	rs7815841	0.99	rs7815841	G	C	1.9222	1.8761	0.3100	0.0011	0.0107	0.9200	-0.0409542	0.0214002	0.056	-0.00255731	0.00519147	0.62
rs1011870	692315	9	103061366	G	A	*	rs5029343	0.61	rs5029343	C	T	2.0825	1.7454	0.2300	0.0049	0.0100	0.6200	0.00625474	0.0199221	0.75	0.00702788	0.00486365	0.15
rs1013189	691777	14	97258752	C	A	*																	
rs1013228	786578	14	25928179	C	A				rs10132280	C	A	4.6079	2.1639	0.0330	0.0142	0.0124	0.2500	0.049652	0.0247524	0.045	-0.0007806	0.00594198	0.9
rs1014194	686107	5	168192944	A	C				rs1014194	C	A	1.3936	1.2230	0.2500	-0.0132	0.0070	0.0590	0.0136006	0.0139911	0.33	-0.00294194	0.00337058	0.38
rs1014312	686402	3	135621417	C	G	*																	
rs1014652	781339	14	79499850	T	C				rs10146527	T	C	1.1481	1.4822	0.4400	-0.0018	0.0085	0.8300	0.0266222	0.0168974	0.12	0.00151089	0.00409065	0.71
rs1015362	692525	20	32738612	C	T				rs1015362	C	T	1.4947	1.5762	0.3400	0.0132	0.0090	0.1400	-0.0043774	0.0180055	0.81	-0.00263387	0.00437445	0.55
rs1015363	687260	20	32738335	G	A	*	rs11700255	0.89	rs11700255	T	A	1.9402	1.7083	0.2600	-0.0116	0.0097	0.2300	-0.0060141	0.0194501	0.76	-0.00325405	0.0047398	0.49
rs1016819	689662	2	51834839	G	C				rs10168197	C	G	0.5132	1.3482	0.7000	0.0009	0.0077	0.9100	0.0201479	0.0153926	0.19	0.00364321	0.0037456	0.33
rs1018218	792111	2	25150296	G	A				rs10182181	A	G	0.4013	1.2171	0.7400	-0.0008	0.0069	0.9100	-0.0032633	0.0138897	0.81	0.000693228	0.00335198	0.84
rs1019033	527668	2	61619267	T	G				rs10190332	G	T	0.9483	1.2888	0.4600	-0.0056	0.0073	0.4400	0.00393821	0.0146824	0.79	0.00592156	0.00356491	0.097
rs1020327	690127	2	103496700	A	G				rs10203277	G	A	0.1876	1.5649	0.9000	0.0128	0.0090	0.1500	0.0336958	0.0179005	0.06	-0.00446314	0.00436243	0.31
rs1020548	688748	6	56810539	G	A	*																	
rs1021105	686577	2	229016917	C	T	*																	
rs1026805	794416	7	44522617	C	T				rs10268050	C	T	1.8875	4.6247	0.6800	0.0145	0.0266	0.5800	-0.0349953	0.0529244	0.51	-0.00568638	0.0127415	0.66
rs1026978	790551	7	49616203	A	G				rs10269783	G	A	0.5335	1.1894	0.6500	0.0026	0.0068	0.7000	-0.012506	0.0135624	0.36	-0.00070188	0.00327623	0.83
rs1027504	610254	7	1273845	T	A	*																	
rs1035010	637868	7	69598328	T	C				rs1035010	C	T	1.1692	1.3016	0.3700	0.0075	0.0074	0.3100	-0.0043932	0.0148317	0.77	0.00405518	0.00358987	0.26
rs1037587	687559	11	11796727	T	C				rs1037587	T	C	0.9550	1.2836	0.4600	0.0010	0.0073	0.8900	0.0208944	0.0146417	0.15	-0.00083395	0.00354837	0.81
rs1038088	794680	17	28074563	G	T				rs1038088	G	T	1.2107	1.2941	0.3500	0.0090	0.0074	0.2200	0.016549	0.0147542	0.26	-0.00149846	0.00360105	0.68
rs1040801	794820	19	33963766	T	C				rs10408013	C	T	0.6533	1.8619	0.7300	0.0057	0.0107	0.5900	-0.0029358	0.0213041	0.89	-0.00300625	0.00514181	0.56
rs1040881	691190	20	15099816	T	C				rs1040881	C	T	1.8938	4.9050	0.7000	-0.0047	0.0280	0.8700	0.0668381	0.0558644	0.23	0.0127991	0.0136027	0.35
rs1043896	686868	18	42950629	C	T	*																	
rs1045411	776153	13	31033232	C	T				rs1045411	C	T	1.0417	1.4320	0.4700	-0.0151	0.0082	0.0650	-0.0246306	0.0162923	0.13	0.00795487	0.00394057	0.044
rs1045663	788067	6	50763935	A	T	*																	
rs1045901	792142	11	55091574	A	C				rs10459012	A	C	1.0749	1.2672	0.4000	-0.0128	0.0072	0.0770	0.0107592	0.0144409	0.46	-0.00103549	0.00350866	0.77
rs1046096	793248	3	42308735	A	G	*	rs1799923	0.71	rs1799923	A	G	1.2748	1.3563	0.3500	-0.0129	0.0078	0.0970	-0.0051259	0.0154979	0.74	0.0094357	0.00377299	0.012
rs1046753	689848	13	54694130	C	G	*	rs4883848	1.00	rs4883848	C	A	0.9874	1.8930	0.6000	-0.0123	0.0108	0.2500	0.00282461	0.0216486	0.9	-0.00092881	0.00522402	0.86
rs1048365	636076	7	100804430	T	C				rs1048365	C	T	0.8479	1.2382	0.4900	-0.0018	0.0071	0.8000	0.0073644	0.0141078	0.6	-0.00014058	0.00341838	0.97
rs1048775	690082	17	79202329	C	G				rs1048775	G	C	2.2665	1.3171	0.0850	0.0049	0.0075	0.5200	-0.0080522	0.015054	0.59	-0.00157303	0.00365615	0.67
rs1048932	795167	11	115044850	C	A				rs1048932	C	A	2.9891	1.2188	0.0140	0.0093	0.0070	0.1800	-0.0141859	0.0139668	0.31	0.0027804	0.00338987	0.41
rs1049780	780080	2	198585087	G	C	*																	
rs1049927	794584	6	154309808	T	C				rs10499276	T	C	1.6615	2.6381	0.5300	-0.0128	0.0151	0.3900	-0.0541782	0.0303225	0.074	0.00855021	0.00735296	0.24
rs1050054	690612	16	69174141	T	C				rs10500548	T	C	2.3347	4.1040	0.5700	-0.0534	0.0235	0.0230	-0.0842066	0.0468001	0.072	-0.0119847	0.0115302	0.3
rs1051032	685852	3	6009092	T	C	*																	
rs1051109	692481	3	83763541	C	T	*																	
rs1051505	691102	17	51923847	C	T				rs10515050	C	T	2.5716	1.4901	0.0840	-0.0057	0.0085	0.5000	-0.0020411	0.0170315	0.9	0.00421149	0.00410524	0.3
rs1051915	692302	15	77156899	T	A				rs10519151	A	T	1.4428	3.0385	0.6300	-0.0005	0.0174	0.9700	0.00087838	0.0347526	0.98	0.00278769	0.00849574	0.74
rs1057452	692447	16	29833714	A	G				rs1057452	A	G	2.4797	3.1860	0.4400	0.0175	0.0180	0.3300	-0.0223789	0.0359717	0.53	-0.00608594	0.00896737	0.5
rs1073305	781928	1	167280354	A	G				rs10733051	A	G	0.7139	1.3100	0.5900	0.0009	0.0075	0.9000	-0.0093504	0.0149628	0.53	-0.00236899	0.00361773	0.51
rs1074414	689141	12	17212881	G	A				rs10744146	G	A	0.4530	1.2609	0.7200	0.0054	0.0072	0.4500	-0.0045699	0.0144086	0.75	0.00444219	0.00350759	0.21
rs1074578	691086	12	97586257	C	T				rs10745785	T	C	1.1846	1.2442	0.3400	0.0017	0.0071	0.8100	0.00180236	0.0141862	0.9	-0.00476529	0.00343793	0.17
rs1075421	692251	1	197012111	G	A				rs10754210	G	A	2.4969	1.7969	0.1600	0.0138	0.0102	0.1800	0.0210321	0.0204474	0.3	-0.004359	0.00492588	0.38
rs1075901	794789	17	15943910	C	T				rs1075901	T	C	0.3993	1.3933	0.7700	-0.0091	0.0080	0.2500	-0.0017954	0.0158736	0.91	-0.00494909	0.00386184	0.2
rs1076916	794571	11	45706453	C	T				rs10769165	T	C	0.4450	1.2165	0.7100	0.0041	0.0070	0.5500	0.00889465	0.0139017	0.52	-0.0068556	0.00336447	0.042
rs1077205	792168	12	33379440	G	C	*	rs1993512	0.62	rs1993512	C	G	0.9792	1.4371	0.5000	-0.0091	0.0082	0.2700	-0.0125366	0.0164379	0.45	0.00194293	0.00398959	0.63
rs1077304	790708	12	124506631	C	T				rs10773049	T	C	0.9637	1.2048	0.4200	-0.0062	0.0069	0.3700	0.0102042	0.013744	0.46	-0.00516656	0.00333254	0.12
rs1077975	794997	1	11284336	A	G	*	rs6670821	1.00	rs6670821	C	T	4.6827	2.2069	0.0340	0.0197	0.0126	0.1200	0.0450112	0.0252096	0.074	0.00156131	0.0061157	0.8
rs1079711	687215	9	92191256	T	C				rs10797115	T	C	1.8705	1.4148	0.1900	-0.0050	0.0081	0.5400	0.0178962	0.0161576	0.27	0.00124685	0.00391858	0.75
rs1081881	770877	9	126096522	A	G	*	rs7034840	0.71	rs7034840	T	C	0.8251	1.2166	0.5000	0.0094	0.0070	0.1800	-0.005758	0.0138887	0.68	0.000215014	0.0033855	0.95
rs1081893	691410	9	127049237	A	G				rs10818938	G	A	0.3274	1.2312	0.7900	-0.0042	0.0070	0.5500	0.00274819	0.0139874	0.84	0.00160234	0.00341299	0.64
rs1083045	690453	11	89966202	G	A				rs10830452	G	A	1.6193	1.1836	0.1700	-0.0044	0.0068	0.5200	-0.0028977	0.0135376	0.83	-0.00031975	0.00328532	0.92
rs1083277	783042	11	17394073	G	C	*	rs12146443	0.97	rs12146443	T	C	0.8495	1.2538	0.5000	-0.0007	0.0071	0.9200	0.0127419	0.0142737	0.37	-0.00207755	0.00345572	0.55
rs1083812	673207	11	43551416	C	T	*	rs10838123	0.91	rs10838123	G	C	0.9432	1.1915	0.4300	0.0165	0.0068	0.0150	-0.0043485	0.0135811	0.75	0.000484537	0.00328323	0.88
rs1083885	692513	11	48286256	T	C				rs10838852	T	C	1.0178	1.2287	0.4100	0.0116	0.0070	0.0990	-0.0040945	0.0140144	0.77	-0.00295538	0.0033918	0.38
rs1083947	691909	11	49994823	T	C	*	rs10734604	0.88	rs10734604	T	C	1.5667	1.4478	0.2800	0.0045	0.0083	0.5900	0.0004486	0.0165246	0.98	0.00287926	0.0040057	0.47
rs1084060	642006	11	2234690	G	A				rs10840606	A	G	1.5140	2.2720	0.5100	0.0037	0.0130	0.7800	-0.0079583	0.0260387	0.76	0.0138072	0.00634441	0.03
rs1084067	791973	12	17360254	G	A	*	rs10840672	0.93	rs10840672	G	A	2.1187	2.0369	0.3000	-0.0077	0.0116	0.5100	0.0165795	0.0231134	0.47	0.00749029	0.00565296	0.19
rs1084224	691476	12	24060075	C	G	*	rs4517619	0.69	rs4517619	T	G	1.1318	1.2759	0.3800	-0.0094	0.0073	0.2000	-0.0045578	0.0145579	0.75	-0.0030268	0.00353347	0.39
rs1085003	794630	12	112771063	G	T	*	rs7974266	0.68	rs7974266	C	T	0.0318	1.8444	0.9900	-0.0155	0.0105	0.1400	-0.0290018	0.0209589	0.17	0.00340776	0.00505307	0.5
rs1086585	691886	3	34700713	T	C	*	rs17676830	0.97	rs17676830	C	T	1.6384	1.2512	0.1900	0.0057	0.0072	0.4200	0.00541843	0.0142894	0.7	0.000189743	0.00347136	0.96
rs1086725	689493	9	81367391	C	T				rs10867256	T	C	2.3084	1.3134	0.0790	0.0044	0.0075	0.5600	-0.0262248	0.0149895	0.08	-0.0024907	0.00363035	0.49
rs1087641	787067	12	39428802	C	T				rs10876418	T	C	1.3479	5.9308	0.8200	0.0141	0.0340	0.6800	0.0370522	0.0677205	0.58	-0.0257111	0.0164268	0.12
rs1087894	685707	12	69642315	C	T	*	rs479971	0.99	rs479971	G	A	2.0037	1.4036	0.1500	0.0057	0.0080	0.4800	-0.0089034	0.0160358	0.58	0.00084594	0.00388205	0.83
rs1088355	690191	10	102635475	A	C	*																	
rs1088375	791911	10	104412049	G	A	*	rs3977743	1.00	rs3977743	A	G	1.0148	1.2565	0.4200	0.0085	0.0072	0.2400	0.0231126	0.0143721	0.11	0.0043634	0.00350099	0.21
rs1088601	786354	10	118672531	A	C	*	rs11598817	0.61	rs11598817	A	G	2.0119	1.1702	0.0860	0.0026	0.0067	0.7000	-0.0089946	0.0133358	0.5	-0.00228517	0.00325361	0.48
rs1088757	679172	10	88096047	C	G	*	rs11201985	0.93	rs11201985	A	G	0.2796	1.4466	0.8500	-0.0018	0.0082	0.8300	-0.0327758	0.0164717	0.047	0.00408771	0.00402313	0.31
rs1091584	684857	1	225668524	G	A				rs10915840	G	A	0.6646	1.6810	0.6900	-0.0036	0.0096	0.7000	-0.0367599	0.0191809	0.055	0.000673887	0.0046739	0.89
rs1092067	788624	1	190239907	A	G				rs10920678	G	A	0.7444	1.2519	0.5500	0.0045	0.0072	0.5300	0.00482076	0.0143099	0.74	0.00316139	0.00346019	0.36
rs1092372	789514	1	119546842	C	T				rs10923724	C	T	1.1429	1.1550	0.3200	-0.0038	0.0066	0.5700	-0.0173052	0.0131814	0.19	0.00116382	0.00318502	0.71
rs1092992	787036	2	6155557	C	A	*	rs2609182	0.67	rs2609182	G	T	0.2810	1.2213	0.8200	0.0056	0.0070	0.4200	-0.0210781	0.0139144	0.13	0.00535319	0.0033705	0.11
rs1093064	690575	2	175000711	G	A	*																	
rs1093514	688983	3	134665159	G	A	*																	

rs1093839	793518	4	45182527	G	A				rs10938397	A	G	0.3220	1.3266	0.8100	-0.0046	0.0076	0.5400	-0.0359626	0.0151465	0.018	-0.00173749	0.00367953	0.64
rs1095362	687660	7	109173373	C	A				rs10953620	A	C	0.7232	1.4517	0.6200	-0.0034	0.0083	0.6800	-0.0151171	0.0165441	0.36	-0.00342538	0.00399254	0.39
rs1095584	688388	8	118946541	A	G				rs10955841	A	G	0.5458	1.1905	0.6500	-0.0016	0.0068	0.8100	0.00830581	0.0135935	0.54	-0.00026185	0.00327537	0.94
rs1096254	692271	9	16719445	T	C				rs10962549	T	C	0.7625	1.3754	0.5800	0.0030	0.0079	0.7100	-0.0130644	0.0156649	0.4	-0.00114364	0.00379713	0.76
rs1097171	692587	9	33820938	C	T				rs10971712	T	C	0.7523	6.5444	0.9100	-0.0173	0.0371	0.6400	-0.145702	0.0738757	0.049	0.00377334	0.0180438	0.83
rs1098956	688508	9	104396304	A	G				rs10989568	A	G	0.8645	1.1940	0.4700	0.0070	0.0068	0.3100	-0.002713	0.0136243	0.84	0.00647977	0.00330183	0.05
rs1103038	692117	11	28629115	G	A				rs11030385	A	G	0.5325	1.3780	0.7000	0.0031	0.0079	0.7000	0.00853244	0.0156823	0.59	0.00420829	0.0038059	0.27
rs1103901	793931	11	46895378	A	G	*																	
rs1106630	795498	12	112871372	A	G				rs11066301	G	A	1.9746	4.9700	0.6900	-0.0338	0.0282	0.2300	0.0315811	0.0561129	0.57	0.00278498	0.0135611	0.84
rs1106908	795186	17	34942595	G	A				rs1106908	G	A	2.2762	1.2563	0.0700	0.0005	0.0072	0.9400	-0.0131464	0.0143439	0.36	0.0021642	0.00347612	0.53
rs1107444	773181	16	20255123	T	C				rs11074446	C	T	0.2414	1.4035	0.8600	0.0016	0.0080	0.8400	0.0006951	0.0160399	0.97	-0.0107319	0.00386994	0.0056
rs1107548	688802	16	62803841	C	T				rs11075489	T	C	0.0931	1.2049	0.9400	-0.0058	0.0069	0.4000	0.00117923	0.013748	0.93	-0.00241486	0.0033266	0.47
rs1107598	790364	16	53805344	C	G	*																	
rs1107984	687694	17	47090785	C	T	*																	
rs1111517	792384	12	82465797	T	C				rs11115176	C	T	0.4182	1.4100	0.7700	0.0015	0.0081	0.8500	0.0019076	0.0161372	0.91	-0.00460113	0.00392536	0.24
rs1111830	794625	1	219633869	A	G				rs11118308	G	A	0.3740	1.3039	0.7700	0.0104	0.0075	0.1700	-0.0071899	0.0149304	0.63	-0.00059305	0.00363336	0.87
rs1112876	690962	3	15873407	A	G				rs11128760	A	G	1.1555	1.2453	0.3500	0.0200	0.0071	0.0051	-0.0076273	0.014261	0.59	-0.00442815	0.00344123	0.2
rs1115091	781716	18	73498528	A	C				rs11150911	C	A	0.3558	1.3108	0.7900	0.0037	0.0075	0.6200	0.0127645	0.0149132	0.39	-0.00553958	0.00360931	0.12
rs1117270	681630	12	39982413	A	G				rs11172702	A	G	1.4153	1.3293	0.2900	0.0006	0.0076	0.9400	-0.0036674	0.0151514	0.81	0.00225575	0.00365585	0.54
rs1119066	690855	10	102452136	T	C				rs11190661	C	T	0.3987	1.3017	0.7600	0.0042	0.0074	0.5700	0.00952194	0.0148416	0.52	0.000383627	0.00359089	0.91
rs1120866	691220	1	65987164	C	G	*	rs11208660	0.80	rs11208660	T	C	2.4205	2.3064	0.2900	0.0123	0.0131	0.3500	0.0147421	0.0262114	0.57	-0.0034571	0.006359	0.59
rs1123154	613180	11	55686588	G	A	*																	
rs1125007	566163	8	10647823	A	G	*	rs4321967	0.67	rs4321967	T	C	2.3186	1.2128	0.0560	-0.0080	0.0069	0.2500	-0.0141186	0.0138073	0.31	-0.00404015	0.00335119	0.23
rs1125135	690804	10	2585792	G	A	*																	
rs1125993	703399	15	84580156	G	A				rs11259933	G	A	2.2268	1.3256	0.0930	0.0053	0.0076	0.4800	0.00012464	0.0151326	0.99	-0.00438352	0.00366763	0.23
rs1149612	684574	7	103417557	T	C				rs11496125	T	C	0.8186	1.2218	0.5000	-0.0014	0.0070	0.8400	-0.0111955	0.0139398	0.42	0.00116774	0.00338464	0.73
rs1157717	790199	1	155983710	G	A	*																	
rs1158103	688665	1	101024370	G	A				rs1158103	G	A	1.2502	1.2819	0.3300	0.0091	0.0073	0.2200	0.0364434	0.0146725	0.013	0.000742719	0.00353714	0.83
rs1158312	784393	1	38053458	T	C				rs11583122	T	C	1.8031	7.8322	0.8200	0.0195	0.0483	0.6900	0.0907473	0.0963824	0.35	0.0163801	0.0225533	0.47
rs1159047	685475	1	174972588	C	T				rs11590474	C	T	1.7331	2.8595	0.5400	0.0095	0.0163	0.5600	-0.0367874	0.0326997	0.26	-0.0188759	0.00792697	0.017
rs1160099	691086	11	64082807	C	T				rs11600990	T	C	1.8220	1.8506	0.3200	-0.0064	0.0106	0.5400	0.00262931	0.0211007	0.9	-0.00239158	0.00514941	0.64
rs1161124	779823	12	939480	T	G				rs11611246	G	T	1.6160	1.3185	0.2200	-0.0051	0.0076	0.5000	0.00484932	0.0150887	0.75	-0.00069066	0.00368716	0.85
rs1161149	690148	12	108413828	G	A	*	rs11113659	0.68	rs11113659	C	T	1.8458	1.5522	0.2300	0.0014	0.0089	0.8800	0.0206836	0.0177421	0.24	-0.00122213	0.00432444	0.78
rs1162978	690824	15	66741387	C	G	*	rs78136741	1.00	rs78136741	A	G	0.9373	1.5082	0.5300	-0.0081	0.0087	0.3500	-0.0151749	0.0172696	0.38	-0.00407257	0.00421984	0.33
rs1163567	688719	15	63793238	T	G	*																	
rs1164200	691916	16	71899586	G	A				rs11642001	G	A	0.8721	1.4322	0.5400	0.0123	0.0082	0.1300	0.0281091	0.0163779	0.086	-0.00101964	0.00398223	0.8
rs1165976	690582	18	53335512	T	A	*																	
rs1166830	691176	19	31016196	A	G	*	rs73022877	0.94	rs73022877	A	T	2.2044	7.2794	0.7600	0.0065	0.0412	0.8700	0.0982463	0.0821732	0.23	-0.0238715	0.0211003	0.26
rs1167266	768426	19	46180184	C	T				rs11672660	T	C	0.0174	1.4658	0.9900	0.0022	0.0084	0.7900	-0.0107758	0.016768	0.52	0.00303205	0.00409511	0.46
rs1171133	687496	3	21794904	T	A				rs11711337	A	T	0.0579	1.2599	0.9600	0.0019	0.0072	0.8000	0.0180349	0.0143863	0.21	0.00376126	0.00348756	0.28
rs1173622	691580	4	147376805	A	T				rs11736228	A	T	1.1534	1.4919	0.4400	-0.0024	0.0085	0.7800	-0.0150247	0.017035	0.38	-0.00058961	0.00410842	0.89
rs1175308	790084	6	20705590	T	G				rs11753081	T	G	0.4932	1.1965	0.6800	0.0010	0.0068	0.8900	0.00455357	0.0136366	0.74	-2.9436E-05	0.00330835	0.99
rs1175665	659948	6	50054296	G	C	*																	
rs1177336	690588	7	147668180	C	T				rs11773362	T	C	0.0543	1.2314	0.9600	-0.0022	0.0070	0.7500	0.0122436	0.0140298	0.38	-0.00111144	0.00341119	0.74
rs1178060	780840	6	50914343	A	G	*	rs72901027	0.91	rs72901027	G	A	3.0061	7.2796	0.6800	-0.0151	0.0412	0.7100	-0.0436517	0.0821279	0.6	-0.0193011	0.0206815	0.35
rs1178122	789828	8	23389571	T	C				rs11781222	T	C	1.6100	1.3186	0.2200	0.0029	0.0075	0.7000	-0.0147939	0.0150283	0.32	-0.00666707	0.00363874	0.067
rs1178169	784642	8	118863061	C	T	*	rs72673939	0.98	rs72673939	C	G	1.7609	3.3225	0.6000	0.0096	0.0189	0.6100	-0.065143	0.0377365	0.084	-0.00836159	0.00918189	0.36
rs1178324	790309	8	10788875	C	T	*																	
rs1179028	684876	9	14651283	C	T				rs11790280	C	T	1.9737	1.4923	0.1900	-0.0136	0.0085	0.1100	-0.0322435	0.0170129	0.058	0.000856831	0.00412898	0.84
rs1179206	644252	9	140646121	A	G				rs11792069	G	A	1.2915	5.3668	0.8100	0.0405	0.0307	0.1900	-0.0346625	0.0617053	0.57	0.00382396	0.0151707	0.8
rs1180311	662659	1	174002465	T	A	*																	
rs1184468	686135	14	65910844	G	C				rs11844682	C	G	0.7736	1.9875	0.7000	0.0076	0.0113	0.5000	0.00073603	0.0225714	0.97	-0.00469237	0.00552686	0.4
rs1185585	682564	15	78012618	C	T	*	rs11855821	0.84	rs11855821	A	G	1.7174	3.0054	0.5700	0.0229	0.0172	0.1800	0.0291955	0.0341914	0.39	0.000469316	0.00851515	0.96
rs1186681	792501	16	387867	C	T				rs11866815	T	C	1.5402	1.5177	0.3100	-0.0083	0.0087	0.3400	0.0276046	0.0173535	0.11	-0.00263812	0.00427301	0.54
rs1188953	688977	2	220163543	A	G				rs11889536	G	A	1.2157	2.0437	0.5500	0.0028	0.0117	0.8100	-0.0049556	0.0232697	0.83	0.00627166	0.005686	0.27
rs1191537	690829	3	70539559	C	A				rs11915371	C	A	1.7125	2.3601	0.4700	-0.0376	0.0135	0.0054	0.0117182	0.0270089	0.66	-0.00902353	0.00654279	0.17
rs1192902	675947	3	51754424	T	G	*																	
rs1194586	682451	4	65700865	G	A				rs11945861	A	G	2.5778	1.1992	0.0320	-0.0004	0.0068	0.9600	0.0236018	0.0136967	0.085	-0.00328377	0.00329426	0.32
rs1197104	689741	7	26698848	G	A	*																	
rs1198738	688014	8	15197115	C	A	*	rs7819078	0.83	rs7819078	G	A	1.5059	3.6312	0.6800	-0.0108	0.0208	0.6000	0.0112153	0.0416682	0.79	0.000376772	0.0104325	0.97
rs1203325	664083	1	112318484	A	G				rs12033257	A	G	0.9094	1.3158	0.4900	-0.0028	0.0075	0.7100	0.0313173	0.0149638	0.036	-0.00266648	0.00361422	0.46
rs1203514	691352	1	107885018	G	C				rs12035149	C	G	0.0129	1.5387	0.9900	0.0109	0.0088	0.2100	-0.0089547	0.0175918	0.61	-0.00176505	0.00422919	0.68
rs1203534	666990	1	77557339	G	A				rs12035349	G	A	2.0409	2.0280	0.3100	-0.0002	0.0116	0.9900	-0.000736	0.0232418	0.97	0.0029487	0.00560841	0.6
rs1203952	690240	1	173549827	A	G				rs12039524	G	A	0.2945	1.3053	0.8200	-0.0068	0.0075	0.3600	0.00040103	0.0148975	0.98	-0.00334673	0.00362188	0.36
rs1204125	688602	1	195047936	T	C	*	rs1888623	0.99	rs1888623	A	T	1.8334	1.3391	0.1700	-0.0029	0.0076	0.7000	0.0096137	0.0152661	0.53	-0.00226037	0.0037214	0.54
rs1204290	794074	1	74997762	A	G				rs12042908	A	G	2.4458	1.4933	0.1000	-0.0115	0.0085	0.1800	-0.0031675	0.0169999	0.85	-0.00148487	0.00412551	0.72
rs1204295	685384	1	243533273	A	G	*	rs10927006	0.97	rs10927006	C	T	1.5963	2.2019	0.4700	0.0020	0.0126	0.8700	0.0448841	0.0251177	0.074	-0.0056053	0.0062104	0.37
rs1204459	789125	1	1708801	G	A				rs12044597	G	A	0.0214	1.2202	0.9900	0.0023	0.0070	0.7400	-0.005532	0.0139441	0.69	0.00101919	0.00335854	0.76
rs1205106	685044	14	72269668	G	A				rs1205106	A	G	0.5088	1.2062	0.6700	-0.0086	0.0069	0.2100	-0.012921	0.0137788	0.35	0.00157906	0.00334036	0.64
rs1209828	686167	10	76047464	T	C				rs12098284	T	C	1.7047	1.8632	0.3600	0.0143	0.0106	0.1800	0.0232577	0.0212555	0.27	0.00597393	0.00514736	0.25
rs1210139	689675	15	92570921	C	G				rs12101393	G	C	3.1277	6.8303	0.6500	0.0175	0.0387	0.6500	0.190271	0.0771552	0.014	-0.036129	0.0202892	0.075
rs1214784	687445	14	101144596	T	C	*	rs2295659	0.71	rs2295659	T	G	1.3382	2.1178	0.5300	-0.0143	0.0121	0.2400	-0.0110804	0.0241097	0.65	0.01011	0.00587112	0.085
rs1214838	687020	15	77254544	C	T				rs12148386	T	C	1.6233	1.3121	0.2200	-0.0051	0.0075	0.4900	0.0295042	0.0149267	0.048	-0.00805107	0.00360905	0.026
rs1218822	794711	13	28011963	A	G	*																	
rs1218917	789640	5	50914726	T	C	*																	
rs1220724	680683	6	57642076	G	A				rs12207241	G	A	0.7483	1.2378	0.5500	0.0069	0.0071	0.3300	-0.0074026	0.0141456	0.6	0.00293034	0.00342387	0.39
rs1221533	758914	6	34644749	T	C	*	rs12199648	0.89	rs12199648	A	G	12.0566	4.5397	0.0079	0.0159	0.0259	0.5400	0.0693685	0.0512442	0.18	-0.0314688	0.0122343	0.01
rs1229057	683545	7	39054538	T	C	*																	
rs1230693	785165	12	38242029	C	T	*	rs10880614	0.84	rs10880614	A	G	0.3119	1.5603	0.8400	0.0023	0.0089	0.8000	-0.0082823	0.0178545	0.64	-0.00201591	0.00428395	0.64
rs1232727	690701	18	57726627	A	G				rs12327272	G	A	1.8217	1.5983	0.2500	0.0052	0.0092	0.5700	0.0145131	0.0182727	0.43	-0.00530567	0.00443104	0.23
rs1236447	787411	11	134601012	G	T	*	rs61909169	0.66	rs61909169	A	G	2.4956	5.3142	0.6400	-0.0110	0.0301	0.7100	0.0956923	0.0600918	0.11	-0.0223273	0.0145917	0.13
rs1236900	794474	12	112019799	G	T	*																	
rs1241188	792405	10	104685299	A	C				rs12411886	C	A	1.5917	1.3736	0.2500	0.0069	0.0078	0.3800	0.0172524	0.0156583	0.27	-0.00374693	0.00377882	0.32
rs1241707	680580	11	115623272	G	A				rs12417072	A	G	2.8299	2.5839	0.2700	-0.0102	0.0147	0.4900	-0.046624	0.0293433	0.11	-0.00902433	0.00710479	0.2
rs1241986	687757	18	6873954	G	A				rs1241986	G	A	0.1433	1.2575	0.9100	0.0011	0.0072	0.8800	-0.0249545	0.0143579	0.082	0.00283353	0.00351826	0.42
rs1242072	679599	11	117017530	G	A				rs12420725	G	A	1.4965	2.1309	0.4800	0.0095	0.0122	0.4400	-0.0107228	0.024334	0.66	0.00575564	0.00592031	0.33
rs1242255	689543	12	14413931	G	C				rs12422552	G	C	1.1428	1.2816	0.3700	0.0026	0.0073	0.7200	0.00805282	0.0146372	0.58	0.00124338	0.00356148	0.73

rs1242954	778918	13	54102206	A	G				rs12429545	G	A	0.7910	1.4196	0.5800	-0.0038	0.0081	0.6400	-0.0081399	0.0162261	0.62	-0.00113215	0.00393181	0.77
rs1243979	690093	15	46584787	T	G				rs12439798	T	G	1.2895	1.3116	0.3300	0.0056	0.0075	0.4500	-0.0126845	0.0149348	0.4	-0.00103138	0.00363084	0.78
rs1244362	688078	16	52548037	G	A				rs12443621	G	A	1.9360	1.1803	0.1000	0.0078	0.0067	0.2500	-0.0221617	0.0134896	0.1	0.00641263	0.00326645	0.05
rs1244663	788565	16	19935389	G	A	*																	
rs1244825	779628	16	3599655	A	G				rs12448257	G	A	0.8052	1.3756	0.5600	-0.0108	0.0078	0.1700	-0.0226331	0.0156502	0.15	0.00102717	0.00382384	0.79
rs1244873	691932	16	56489343	C	A				rs12448738	A	C	0.1783	1.7152	0.9200	-0.0243	0.0098	0.0130	0.00167371	0.019509	0.93	0.00687312	0.00473686	0.15
rs1245341	688156	17	31747629	G	A	*																	
rs12458	635629	8	11617240	A	T				rs12458	T	A	2.0755	1.1894	0.0810	0.0072	0.0068	0.2900	0.00593291	0.0136019	0.66	-0.00499295	0.00331062	0.13
rs1247923	692419	2	228891702	T	A				rs12479233	A	T	2.0547	1.2422	0.0980	0.0017	0.0071	0.8100	0.00133359	0.0141821	0.93	-3.1236E-05	0.00344137	0.99
rs1248071	663785	20	15801600	T	C				rs12480713	T	C	1.1008	1.1828	0.3500	0.0023	0.0067	0.7300	-0.0068698	0.0134769	0.61	-0.00200276	0.00327885	0.54
rs1248823	787551	3	56114861	C	T				rs12488237	C	T	1.3349	2.2131	0.5500	0.0046	0.0126	0.7200	0.0546548	0.0251516	0.03	-0.00619353	0.0060924	0.31
rs1251447	692548	5	80818639	T	C				rs12514473	T	C	1.8005	1.6320	0.2700	-0.0116	0.0093	0.2200	-0.0154434	0.0186333	0.41	0.00315193	0.00446692	0.48
rs1256499	795119	1	174478100	G	A				rs12564992	G	A	0.3809	1.4773	0.8000	-0.0079	0.0084	0.3500	0.00092893	0.0168506	0.96	-0.0010385	0.0040929	0.8
rs1257466	691639	11	46422686	A	C				rs12574668	C	A	2.1403	2.3932	0.3700	-0.0027	0.0138	0.8500	0.0365661	0.0275364	0.18	-0.00243576	0.00671125	0.72
rs1257525	781365	11	8694073	G	C				rs12575252	C	G	3.2221	1.1814	0.0064	0.0250	0.0068	0.0002	-0.0083002	0.0135057	0.54	-0.00243898	0.00327337	0.46
rs1257764	692353	11	43728534	T	A				rs12577642	A	T	1.5430	1.3470	0.2500	-0.0099	0.0077	0.2000	0.0178845	0.0154338	0.25	-0.00211955	0.00373493	0.57
rs1258741	688484	14	47272423	T	G	*																	
rs1259303	686055	15	81058652	A	G				rs12593036	A	G	0.6006	1.3578	0.6600	0.0050	0.0077	0.5200	-0.0203772	0.0155001	0.19	-0.00240715	0.00374622	0.52
rs1259515	775095	15	62316035	C	T				rs12595158	T	C	1.4890	1.5030	0.3200	0.0052	0.0086	0.5500	0.0255111	0.017074	0.14	0.00285586	0.00419571	0.5
rs1259574	692290	15	79432359	A	G	*																	
rs1260291	777510	17	65870073	T	C	*																	
rs1260974	691253	19	12994140	C	T				rs12609744	T	C	0.5774	1.2008	0.6300	0.0068	0.0069	0.3200	0.026504	0.0136936	0.053	0.00062379	0.00332393	0.85
rs1261577	684324	2	102436738	A	G				rs12615778	A	G	4.0806	1.4585	0.0051	-0.0060	0.0083	0.4700	-0.025955	0.016621	0.12	0.0029485	0.00402813	0.46
rs1262541	682052	20	62380542	C	T				rs12625413	T	C	0.8737	1.2265	0.4800	-0.0137	0.0070	0.0500	-0.0147956	0.0139865	0.29	2.36222E-05	0.00338022	0.99
rs1263124	793598	3	50080174	G	C				rs12631248	G	C	2.5136	3.1919	0.4300	0.0412	0.0183	0.0240	-0.0084979	0.0365718	0.82	0.00493907	0.00873299	0.57
rs1263757	637822	3	49237334	T	C	*	rs11920267	0.99	rs11920267	A	G	2.9155	2.6671	0.2700	0.0104	0.0152	0.4900	-0.0131372	0.0302334	0.66	-0.00064497	0.00734687	0.93
rs1263874	681758	3	89331055	G	A				rs12638746	G	A	2.2043	1.2096	0.0680	-0.0011	0.0069	0.8700	-0.0114416	0.0138149	0.41	0.00164327	0.00335287	0.62
rs1265575	685091	5	88858208	A	T				rs12655756	T	A	1.7382	1.6976	0.3100	-0.0058	0.0097	0.5500	0.0247621	0.0194089	0.2	0.0042808	0.00469973	0.36
rs1265980	794156	5	152274478	A	G				rs12659802	A	G	0.7455	1.8588	0.6900	0.0067	0.0106	0.5300	0.00912463	0.0212072	0.67	-0.00149051	0.00513063	0.77
rs1266657	687105	7	26526960	A	G				rs12666574	A	G	0.7284	1.4273	0.6100	-0.0044	0.0082	0.5900	-0.01384	0.0162936	0.4	0.00164077	0.00395418	0.68
rs1266922	689761	6	51822297	A	G				rs1266922	A	G	1.1863	1.5728	0.4500	0.0071	0.0090	0.4300	-7.129E-07	0.0179415	1	-0.00080736	0.00437123	0.85
rs1267506	789771	8	132879047	T	A				rs12675063	A	T	0.2005	1.2581	0.8700	0.0010	0.0072	0.8900	-0.0159911	0.0143459	0.26	0.0100321	0.00347581	0.0039
rs1268084	782549	8	95582606	A	G				rs12680842	G	A	1.4211	1.2037	0.2400	-0.0036	0.0069	0.6000	-0.003758	0.0137099	0.78	-5.622E-05	0.00333753	0.99
rs1268256	774114	8	10626280	A	G	*																	
rs1269402	690963	2	206084372	A	C				rs12694021	C	A	1.3146	1.2036	0.2700	-0.0045	0.0069	0.5100	0.0145337	0.0137333	0.29	-0.00054535	0.00332365	0.87
rs1270598	683609	7	114349212	A	T	*	rs13246064	0.99	rs13246064	A	G	2.0236	1.5834	0.2000	0.0039	0.0091	0.6700	-0.0121222	0.0180748	0.5	-0.00217351	0.00437353	0.62
rs1271343	692534	2	61307982	T	C				rs12713433	C	T	1.1875	2.8260	0.6700	-0.0076	0.0162	0.6400	-0.0119332	0.0322372	0.71	-0.0100418	0.00784769	0.2
rs1273137	791320	1	118852975	T	C				rs12731372	C	T	2.1111	1.4281	0.1400	-0.0080	0.0082	0.3300	0.0206327	0.0162818	0.21	-0.00481214	0.00396937	0.23
rs1277688	676100	10	19776828	A	T	*	rs11010002	0.79	rs11010002	T	C	2.7769	1.2247	0.0230	0.0089	0.0070	0.2000	-0.0195963	0.0139919	0.16	0.000768142	0.00339105	0.82
rs1285245	687342	17	77796889	G	C				rs1285245	C	G	0.3631	1.1896	0.7600	-0.0077	0.0068	0.2600	-0.0235398	0.0135866	0.083	0.000526491	0.00328153	0.87
rs1288545	793069	14	29736838	C	A				rs12885454	A	C	0.5758	1.1812	0.6300	0.0041	0.0068	0.5400	0.00242331	0.0135183	0.86	-0.00379608	0.00326912	0.25
rs1288854	688605	14	88308044	G	A				rs12888545	G	A	1.5724	5.4059	0.7700	0.0387	0.0309	0.2100	0.00214752	0.0616171	0.97	-0.0316185	0.0151625	0.037
rs1290274	690557	15	83647483	A	T				rs12902742	A	T	0.7738	7.3993	0.9200	0.0433	0.0419	0.3000	0.115724	0.0836027	0.17	-0.0221128	0.0206728	0.28
rs1292059	688182	16	67420603	C	T				rs12920590	C	T	0.2318	1.5472	0.8800	-0.0046	0.0088	0.6000	-0.0252199	0.0176632	0.15	-0.00067549	0.0042968	0.88
rs1292234	679615	16	82438337	C	G	*	rs35549276	0.70	rs35549276	A	G	2.0609	1.6575	0.2100	-0.0048	0.0095	0.6100	-0.0090067	0.0189473	0.63	-9.1069E-05	0.0045675	0.98
rs1293954	793950	17	78611724	A	G				rs12939549	G	A	0.7127	1.3114	0.5900	-0.0038	0.0075	0.6100	-0.0262981	0.0149665	0.079	0.00122723	0.00362026	0.73
rs1296328	683488	4	137083193	A	C	*	rs2405510	0.69	rs2405510	C	G	0.7593	1.4019	0.5900	0.0067	0.0080	0.4000	-0.015504	0.0160388	0.33	0.000407101	0.00388587	0.92
rs1296468	692097	18	21116998	A	G				rs12964689	A	G	0.5323	1.2284	0.6600	-0.0047	0.0070	0.5000	0.0116302	0.013985	0.41	0.00299243	0.00339092	0.38
rs1299198	684760	2	199169278	C	G	*																	
rs1302173	789534	2	632348	G	A				rs13021737	A	G	1.7818	2.3072	0.4400	0.0054	0.0131	0.6800	-0.0079708	0.0261993	0.76	-0.00064623	0.00642648	0.92
rs1304117	689601	20	32542814	G	A				rs13041173	A	G	0.0677	1.7572	0.9700	0.0053	0.0100	0.6000	0.0116487	0.0200266	0.56	0.00392578	0.00484233	0.42
rs1304553	560410	20	33595525	C	T	*																	
rs1304741	683228	21	40309436	C	G				rs13047416	G	C	1.2412	1.2066	0.3000	-0.0001	0.0069	0.9900	-0.0018026	0.0138042	0.9	0.00225851	0.00334383	0.5
rs1307209	795578	3	193639623	T	C	*																	
rs1308547	687974	3	171129859	T	C	*																	
rs1309565	675642	3	51243837	C	T	*	rs11712780	0.66	rs11712780	C	T	0.1170	1.2932	0.9300	-0.0038	0.0074	0.6000	-0.0181496	0.0147186	0.22	0.00216344	0.00355995	0.54
rs1310732	792045	4	103188709	T	C	*																	
rs1311026	791087	4	162129844	G	A				rs13110266	A	G	1.8265	1.1859	0.1200	0.0101	0.0068	0.1400	0.0117597	0.0135318	0.38	0.000314825	0.00328689	0.92
rs1316330	690998	5	136571959	G	A	*	rs2117401	0.99	rs2117401	G	A	0.0884	1.3053	0.9500	0.0078	0.0075	0.3000	0.00576666	0.0148841	0.7	0.00267843	0.00359872	0.46
rs1317486	773762	5	139080745	G	A				rs13174863	A	G	2.0522	2.2418	0.3600	0.0013	0.0129	0.9200	0.0282844	0.0257154	0.27	-0.00677135	0.00632962	0.28
rs1317589	663251	5	87866793	C	T	*																	
rs1319136	792699	6	163033350	A	G				rs13191362	G	A	5.1179	6.3732	0.4200	0.0072	0.0366	0.8400	-0.068395	0.0728968	0.35	-0.0199475	0.0175366	0.26
rs1320187	790570	6	137675541	G	A				rs13201877	G	A	1.8054	3.2231	0.5800	-0.0040	0.0184	0.8300	-0.0392496	0.0366046	0.28	0.0105985	0.00888091	0.23
rs1320315	689935	6	97374850	G	A				rs13203153	G	A	1.7974	1.5717	0.2500	0.0025	0.0090	0.7800	0.0243487	0.0179505	0.17	0.00473068	0.00434392	0.28
rs1320328	688204	6	49837540	G	T	*																	
rs1320708	691981	6	27251379	G	A	*																	
rs1320903	691519	3	131758077	A	G				rs1320903	A	G	2.3754	1.6633	0.1500	0.0107	0.0095	0.2600	0.00412738	0.0189193	0.83	0.00463603	0.00457023	0.31
rs1320975	665813	6	86354442	G	A	*	rs145252147	0.79	rs14525214	A	T	1.7772	5.4161	0.7400	-0.0167	0.0309	0.5900	-0.0223047	0.0616746	0.72	-0.00929766	0.0150139	0.54
rs1320987	686531	6	97753223	G	C				rs13209872	C	G	0.0925	1.2058	0.9400	0.0008	0.0069	0.9000	-0.0038398	0.0137528	0.78	0.00172968	0.00333927	0.6
rs1320996	692069	6	126089285	C	G	*																	
rs1322743	663356	7	74094721	G	T				rs13227433	G	T	1.8457	2.2627	0.4100	0.0101	0.0129	0.4400	0.017011	0.0258464	0.51	0.00261324	0.00630301	0.68
rs1322765	688960	7	113353254	C	T	*																	
rs1323068	691801	4	20263058	G	A	*	rs4697156	1.00	rs4697156	C	T	0.2254	1.2694	0.8600	0.0050	0.0073	0.4900	-0.0100171	0.0144958	0.49	0.00429264	0.00349716	0.22
rs1324060	692233	7	99064466	A	G	*	rs7794705	1.00	rs7794705	A	G	2.8835	1.2434	0.0200	-0.0059	0.0071	0.4100	-0.0123952	0.0142228	0.38	-0.00362263	0.00344924	0.29
rs1324110	786661	6	93913200	G	C	*																	
rs1324766	690623	7	93232057	C	T				rs13247665	T	C	0.0797	1.3075	0.9500	-0.0042	0.0075	0.5700	0.00220631	0.0149249	0.88	0.00559871	0.00361321	0.12
rs1326360	686196	8	14095900	C	A				rs13263601	C	A	2.0192	1.4266	0.1600	-0.0021	0.0081	0.7900	0.00536231	0.0162565	0.74	-0.00747108	0.00394549	0.058
rs1329079	688742	9	37183628	G	A				rs13290794	A	G	2.5487	1.8175	0.1600	-0.0011	0.0104	0.9200	-0.0103356	0.0206597	0.62	-0.00165272	0.00498519	0.74
rs1329297	692152	9	129467340	T	C				rs13292976	C	T	0.3943	1.5671	0.8000	-0.0014	0.0089	0.8700	0.0303379	0.0178233	0.089	-0.00173499	0.00428548	0.69
rs1332156	691569	3	107342662	G	A				rs13321566	A	G	67.3660	10.0876	0.0000	0.0710	0.0571	0.2100	-0.046366	0.113848	0.68	0.00814243	0.0266387	0.76
rs1332956	793953	15	68104367	C	T	*	rs4776977	0.99	rs4776977	C	T	0.4995	1.2162	0.6800	-0.0123	0.0070	0.0760	0.0136125	0.0139023	0.33	-0.00043448	0.00337726	0.9
rs1333423	689377	13	59425111	A	T	*	rs1413032	0.88	rs1413032	T	C	0.3803	1.5964	0.8100	-0.0030	0.0091	0.7400	-0.0350311	0.0182004	0.054	0.00224431	0.00440619	0.61
rs1341715	741230	2	62848319	C	T				rs13417156	C	T	3.7443	1.2909	0.0037	0.0067	0.0074	0.3700	0.00070537	0.0147298	0.96	0.000618491	0.00357177	0.86
rs1343205	790223	2	56603985	C	T	*	rs13427152	0.60	rs13427152	T	G	2.3873	2.5087	0.3400	-0.0132	0.0144	0.3600	-0.0235461	0.0288156	0.41	0.00443097	0.00690562	0.52
rs1345148	691590	4	30843533	C	T				rs1345148	C	T	1.3292	1.2372	0.2800	-0.0051	0.0071	0.4700	-0.0062929	0.0141095	0.66	-0.00181233	0.00341283	0.6
rs1358808	689176	6	51825285	G	C				rs1358808	G	C	0.1104	1.3788	0.9400	0.0063	0.0079	0.4200	0.00206608	0.0157094	0.9	-0.00243721	0.00381987	0.52
rs1362910	744652	8	30856464	A	G	*																	

rs1365466	791868	18	36182440	C	T				rs1365466	C	T	0.5689	1.3558	0.6700	0.0013	0.0077	0.8600	0.0107008	0.0154589	0.49	-0.00059558	0.00377263	0.87
rs1371108	684620	2	81816251	A	C	*	rs1371109	0.99	rs1371109	G	A	0.0086	1.5721	1.0000	-0.0033	0.0089	0.7100	0.0118578	0.0178674	0.51	0.0044935	0.00434902	0.3
rs1375561	782697	3	85658230	T	C	*	rs62250504	0.65	rs62250504	G	A	1.2848	1.1689	0.2700	-0.0037	0.0067	0.5800	0.00895671	0.0133701	0.5	-0.00425464	0.00325474	0.19
rs1394	783284	8	9511654	G	A				rs1394	G	A	1.1957	1.2018	0.3200	-0.0065	0.0069	0.3500	0.0122017	0.0137108	0.37	-0.00041301	0.00332857	0.9
rs1399054	690605	8	9785503	G	A				rs1399054	G	A	0.5491	1.3966	0.6900	0.0022	0.0080	0.7900	-0.0044743	0.0159155	0.78	-0.00015766	0.00386239	0.97
rs1403846	787537	4	119101723	T	C	*																	
rs1405348	692448	8	77228222	G	A				rs1405348	A	G	1.3060	1.3072	0.3200	-0.0099	0.0075	0.1900	0.0218206	0.0149356	0.14	-0.00140448	0.00365022	0.7
rs1412235	790147	9	28410996	C	G				rs1412235	G	C	2.2374	1.6522	0.1800	0.0122	0.0095	0.2000	-0.0189046	0.018978	0.32	0.00982427	0.00462185	0.034
rs1423627	678355	5	87125578	T	C	*																	
rs1431659	689739	8	73439070	A	G				rs1431659	A	G	0.8504	1.3005	0.5100	-0.0073	0.0074	0.3300	0.015995	0.014877	0.28	0.0036438	0.00360457	0.31
rs143384	794371	20	34025756	G	A	*	rs224371	0.63	rs224371	G	A	0.2345	1.2503	0.8500	-0.0030	0.0071	0.6700	0.0229219	0.0142326	0.11	0.00378397	0.00345747	0.27
rs1436343	692012	3	104606130	G	A				rs1436343	A	G	1.2572	1.2089	0.3000	-0.0067	0.0069	0.3300	0.0350031	0.0137644	0.011	0.000148684	0.00334189	0.96
rs1437929	690871	2	211983316	A	G	*	rs10804189	0.94	rs10804189	G	A	3.3573	1.5636	0.0320	-0.0011	0.0089	0.9000	0.026134	0.0177958	0.14	-0.00252213	0.00433082	0.56
rs1451533	680795	2	105466005	A	G				rs1451533	A	G	0.5129	1.2169	0.6700	-0.0080	0.0069	0.2500	0.0194959	0.0138541	0.16	-0.00273046	0.00335388	0.42
rs1452075	783729	3	62481063	T	C				rs1452075	C	T	2.3899	1.3539	0.0780	-0.0053	0.0077	0.4900	-0.0139535	0.0154442	0.37	0.00261569	0.00377105	0.49
rs1452134	690523	11	86133416	C	T				rs1452134	T	C	0.9830	1.2240	0.4200	-0.0052	0.0070	0.4600	-0.0199885	0.0139596	0.15	0.00392828	0.00337576	0.24
rs1454148	691136	3	176341188	T	C				rs1454148	C	T	3.2931	1.2445	0.0081	0.0076	0.0071	0.2800	-0.0157619	0.0141938	0.27	-0.00071609	0.00343272	0.83
rs1454687	692324	3	94038085	C	G	*	rs1609906	1.00	rs1609906	A	G	2.4114	1.2196	0.0480	-0.0010	0.0070	0.8900	-0.0234109	0.0139105	0.092	-0.00201416	0.00338699	0.55
rs1455137	792826	4	145986668	C	A	*	rs6823268	1.00	rs6823268	G	A	1.9456	1.7521	0.2700	0.0166	0.0100	0.0970	0.00937266	0.0200879	0.64	0.00102165	0.00485318	0.83
rs1473579	689453	11	48901553	G	A				rs1473579	G	A	1.2943	1.1852	0.2700	0.0070	0.0068	0.3000	-0.008983	0.0135335	0.51	-0.0029469	0.00326558	0.37
rs1475774	669802	6	35619554	A	G				rs1475774	A	G	1.3661	2.7427	0.6200	0.0003	0.0155	0.9900	-0.0075819	0.0310333	0.81	-0.00312289	0.00750744	0.68
rs1477887	679023	4	18514827	G	A				rs1477887	G	A	1.4358	1.1786	0.2200	0.0008	0.0067	0.9100	-0.0034641	0.0134433	0.8	-0.00048468	0.00326045	0.88
rs1492767	794161	4	55221467	T	C				rs1492767	C	T	1.7552	1.3671	0.2000	0.0074	0.0078	0.3400	0.0300761	0.0155771	0.054	-0.00857333	0.00376356	0.023
rs1498139	688263	18	51478026	A	C	*																	
rs1503139	692581	6	54724405	G	A	*	rs6459026	0.61	rs6459026	C	T	2.1388	1.1966	0.0740	-0.0031	0.0068	0.6500	0.00352876	0.0136273	0.8	0.000870525	0.0032972	0.79
rs150353	681804	15	89928189	G	T	*	rs183284	0.88	rs183284	C	T	0.7428	1.1866	0.5300	0.0005	0.0068	0.9500	0.0111913	0.0135279	0.41	0.000346555	0.00328761	0.92
rs1512065	670731	20	53453326	A	G	*																	
rs1522569	689573	4	171632637	T	G	*																	
rs1544459	795359	7	77417584	C	T	*																	
rs1549293	793130	16	31141993	C	T	*																	
rs1552717	692333	11	29158495	A	T	*	rs1491804	1.00	rs1491804	C	T	1.8735	1.6069	0.2400	0.0086	0.0092	0.3500	-0.001244	0.0183193	0.95	-0.00108568	0.00442863	0.81
rs1554193	686046	3	8138801	T	A				rs1554193	A	T	1.4756	1.1929	0.2200	0.0028	0.0068	0.6800	-0.0057936	0.0135958	0.67	0.00202624	0.00331103	0.54
rs1558236	697362	12	111780998	G	C	*	rs73412354	0.99	rs73412354	C	T	2.4164	1.7428	0.1700	0.0113	0.0100	0.2600	0.0129011	0.0199563	0.52	0.00154034	0.00477581	0.75
rs156151	692282	6	104799007	G	C	*	rs156153	1.00	rs156153	G	A	0.2736	1.6475	0.8700	-0.0057	0.0094	0.5400	-0.0155507	0.0188785	0.41	-4.2685E-05	0.0045621	0.99
rs1564981	793125	16	50986308	A	G				rs1564981	A	G	0.0829	1.3223	0.9500	0.0112	0.0075	0.1400	-0.0089648	0.0150524	0.55	0.000119145	0.00363752	0.97
rs159032	682583	5	94206202	T	C				rs159032	T	C	2.2270	2.0244	0.2700	-0.0052	0.0116	0.6500	0.00486271	0.023129	0.83	0.00465756	0.00560609	0.41
rs1593304	669507	7	131619847	A	G	*																	
rs1594830	691520	8	26334167	G	C	*																	
rs1600136	783350	3	136979939	C	A	*																	
rs160401	688395	5	138061341	T	C				rs160401	C	T	1.9221	1.6782	0.2500	0.0000	0.0096	1.0000	-0.0085151	0.0191547	0.66	0.00120076	0.0046902	0.8
rs1608445	683383	18	947954	G	A	*	rs11662628	0.91	rs11662628	T	C	0.2612	1.1928	0.8300	-0.0010	0.0068	0.8800	-0.0080642	0.0136003	0.55	0.00510033	0.00330069	0.12
rs1625427	676334	11	131957293	T	C				rs1625427	T	C	0.9479	1.5544	0.5400	0.0089	0.0089	0.3100	0.00781754	0.0177231	0.66	-0.00137315	0.00430526	0.75
rs1650586	786730	5	157516393	T	G	*	rs2962385	0.90	rs2962385	A	C	2.0247	1.5309	0.1900	0.0033	0.0087	0.7000	0.03185	0.0174097	0.067	-0.00429161	0.00424781	0.31
rs1657930	684138	15	57120989	G	A				rs1657930	A	G	3.7151	1.4501	0.0100	0.0090	0.0083	0.2800	0.0309592	0.0165152	0.061	0.00137577	0.00403513	0.73
rs1658820	669427	8	4288577	T	G	*																	
rs1668633	688791	13	78371890	T	C	*	rs1766355	0.99	rs1766355	C	G	2.0432	1.4663	0.1600	0.0091	0.0084	0.2800	0.00928993	0.0167208	0.58	0.000771022	0.00406536	0.85
rs1682367	678580	3	115071668	A	G				rs16823670	G	A	1.8209	2.4468	0.4600	-0.0132	0.0140	0.3400	-0.0598335	0.0278933	0.032	-0.00230358	0.00680128	0.73
rs1682808	675913	2	151198990	C	G				rs16828086	C	G	0.0398	1.2806	0.9800	0.0097	0.0073	0.1900	-0.0019077	0.0146271	0.9	0.00510052	0.00354653	0.15
rs1685148	692316	3	141275436	T	G				rs16851483	G	T	0.1411	1.5011	0.9300	0.0187	0.0086	0.0290	0.00576342	0.0170807	0.74	-0.00256265	0.00414602	0.54
rs1690684	692124	8	138215228	G	A				rs16906845	G	A	2.3651	1.7594	0.1800	0.0011	0.0101	0.9100	-0.0190391	0.0200699	0.34	0.00257642	0.00491914	0.6
rs1690775	776010	8	81375457	C	T				rs16907751	T	C	1.4871	1.4580	0.3100	-0.0078	0.0083	0.3500	0.0282213	0.0166383	0.09	0.000678479	0.00404237	0.87
rs1693276	685108	8	67202787	G	A				rs16932761	A	G	0.6761	1.4009	0.6300	-0.0098	0.0080	0.2200	-0.0166686	0.0159864	0.3	-0.00121335	0.0038816	0.75
rs1695131	692100	15	68103632	C	T				rs16951319	T	C	0.1052	1.6092	0.9500	-0.0118	0.0092	0.2000	0.0183042	0.0183827	0.32	-0.00118108	0.00446356	0.79
rs1695247	657783	16	53770578	T	A				rs16952479	A	T	2.1443	2.6081	0.4100	-0.0094	0.0149	0.5300	0.00079825	0.0296732	0.98	0.0151837	0.00727416	0.037
rs1696506	689125	18	31581247	T	C	*	rs17747955	0.73	rs17747955	T	C	0.6255	1.9022	0.7400	0.0112	0.0108	0.3000	0.0274355	0.0216313	0.2	0.00134004	0.00524714	0.8
rs1696680	692286	17	39573713	G	A				rs16966801	A	G	1.1869	1.2987	0.3600	-0.0082	0.0074	0.2700	-0.0228306	0.0148062	0.12	0.00141197	0.00359731	0.69
rs1700137	682364	8	89461609	C	T	*																	
rs1701437	690856	1	209543560	G	T				rs17014375	T	G	0.2575	1.7196	0.8800	-0.0065	0.0098	0.5100	-0.038328	0.0196171	0.051	-0.00588866	0.00470422	0.21
rs1703543	683944	2	46878616	A	G				rs17035438	A	G	1.0539	5.0584	0.8300	-0.0109	0.0286	0.7000	-0.0294372	0.0575481	0.61	0.00564455	0.0136994	0.68
rs1705630	688085	5	158271680	C	T				rs17056301	T	C	2.5254	1.3081	0.0540	0.0097	0.0075	0.2000	0.0194142	0.0149566	0.19	0.00659907	0.00362491	0.069
rs1706983	675848	8	4137396	C	T				rs17069831	C	T	0.7432	1.2139	0.5400	-0.0045	0.0069	0.5200	-0.0088849	0.0138712	0.52	-0.0020559	0.00335418	0.54
rs1708031	692606	3	48732480	T	C				rs17080319	C	T	2.2889	3.3256	0.4900	-0.0022	0.0190	0.9100	-0.0216569	0.0379226	0.57	0.00644463	0.00922809	0.48
rs1709147	692573	20	32606299	T	G				rs17091470	T	G	0.2250	1.6115	0.8900	0.0026	0.0092	0.7800	-0.0237417	0.0184298	0.2	-0.0117099	0.00447604	0.0089
rs1709422	791705	10	102395440	C	T				rs17094222	T	C	0.6063	1.3052	0.6400	0.0068	0.0075	0.3600	0.0100504	0.0148988	0.5	-0.00430883	0.00359742	0.23
rs1709654	768355	12	38529333	A	G	*	rs60068856	0.79	rs60068856	G	A	1.2688	1.2260	0.3000	-0.0023	0.0070	0.7400	-0.0106868	0.0139949	0.45	-0.0015875	0.00340163	0.64
rs1709655	691190	14	98626578	G	A				rs17096552	A	G	2.5902	5.6168	0.6400	-0.0211	0.0321	0.5100	-0.0560963	0.0640213	0.38	0.000179715	0.0153008	0.99
rs1710527	681028	14	77529783	T	C				rs17105272	C	T	1.3161	1.1985	0.2700	0.0144	0.0068	0.0360	0.026528	0.0136665	0.052	-0.00583539	0.00331809	0.079
rs1712517	692564	10	105033015	C	T	*	rs3123794	0.96	rs3123794	A	G	0.8053	1.1992	0.5000	-0.0051	0.0068	0.4600	0.00432902	0.0136447	0.75	0.000662757	0.00330839	0.84
rs1719711	795300	11	46520302	C	T				rs17197116	T	C	2.5486	2.4799	0.3000	-0.0054	0.0143	0.7100	0.047315	0.0285803	0.098	-0.00294208	0.00697873	0.67
rs1719997	692337	3	136056184	A	G				rs17199978	G	A	1.9717	6.3670	0.7600	-0.0360	0.0365	0.3300	-0.0220617	0.07278	0.76	0.00543868	0.0185974	0.77
rs1720301	786272	2	208255518	G	A				rs17203016	A	G	0.6315	1.7729	0.7200	-0.0089	0.0101	0.3800	-0.0267037	0.0201929	0.19	0.00401844	0.00494263	0.42
rs1720719	668894	7	75101065	C	T	*	rs794369	0.84	rs794369	C	T	0.2043	1.2330	0.8700	-0.0014	0.0070	0.8400	-0.024118	0.0140418	0.086	-0.00217841	0.00340021	0.52
rs1723811	775505	15	62150364	A	G				rs17238110	G	A	1.4774	1.4978	0.3200	0.0045	0.0085	0.6000	0.0283538	0.0170143	0.096	0.00165765	0.00418714	0.69
rs1728591	679214	5	86908920	T	C				rs17285919	T	C	2.3029	2.2418	0.3000	-0.0074	0.0129	0.5600	-0.0304033	0.0256351	0.24	0.000621551	0.0062166	0.92
rs1730234	632182	1	174983543	C	T	*																	
rs1730859	788691	1	107617707	G	A				rs1730859	A	G	1.3915	1.3053	0.2900	0.0149	0.0075	0.0460	0.0119594	0.014919	0.42	0.00072711	0.00358957	0.84
rs1732746	792231	2	35512183	T	C				rs17327461	C	T	11.4190	3.1325	0.0003	-0.0189	0.0178	0.2900	-0.0083155	0.0356862	0.82	0.0102939	0.00853744	0.23
rs1735179	690594	9	83263089	A	C				rs17351791	C	A	0.5365	1.2068	0.6600	-0.0064	0.0069	0.3500	-0.0091535	0.0137633	0.51	0.00550535	0.00333719	0.099
rs1739169	767720	1	78623626	T	C	*																	
rs174415	677816	5	95933162	T	A				rs174415	T	A	0.5584	1.2386	0.6500	0.0036	0.0071	0.6100	-0.0053983	0.0141597	0.7	-0.00301977	0.00343751	0.38
rs1744609	793600	8	27167942	C	T				rs17446091	T	C	2.9111	2.7266	0.2900	-0.0180	0.0158	0.2600	0.0369942	0.0316232	0.24	0.00693209	0.00769688	0.37
rs1744868	690680	1	15966713	T	C	*	rs34320273	0.69	rs34320273	G	C	2.0143	3.5203	0.5700	-0.0310	0.0202	0.1200	0.0275367	0.0401897	0.49	-0.0332115	0.00982997	0.00073
rs1752212	774342	14	33302882	T	G				rs17522122	G	T	0.3753	1.2040	0.7600	-0.0117	0.0069	0.0920	-0.0069312	0.0138035	0.62	-0.0011631	0.00334066	0.73
rs1752572	679540	5	167293652	G	A	*																	
rs1753136	687883	1	107977075	A	C				rs17531363	C	A	0.7896	1.3574	0.5600	-0.0003	0.0077	0.9700	-0.001381	0.0154787	0.93	0.000676611	0.00375862	0.86
rs1753847	675963	4	163038241	T	C				rs17538472	T	C	1.6058	1.8984	0.4000	0.0021	0.0108	0.8400	0.0199173	0.021581	0.36	0.00417182	0.00522382	0.42
rs1754246	687582	6	35614744	A	G	*																	

rs1755197	691115	2	142293146	C	A	*	rs7574682	0.62	rs7574682	A	C	1.1053	1.2453	0.3700	-0.0102	0.0071	0.1500	-0.0028886	0.01418	0.84	0.00532283	0.00342725	0.12
rs1759177	685869	5	86420392	A	G				rs17591778	A	G	0.3512	1.4259	0.8100	0.0062	0.0082	0.4500	0.0363685	0.0163021	0.026	0.00698332	0.00393751	0.076
rs1760815	791472	12	110046698	T	C				rs17608150	T	C	1.5648	3.3982	0.6500	-0.0176	0.0194	0.3600	-0.0178425	0.0387515	0.65	-0.00655684	0.00941072	0.49
rs1763603	782807	10	126594078	C	T	*																	
rs1768145	692317	3	114399296	G	A				rs17681451	A	G	3.5491	5.5720	0.5200	0.0242	0.0322	0.4500	-0.0101883	0.0641355	0.87	0.0215141	0.0150154	0.15
rs1768170	692361	17	9792872	C	T				rs17681708	C	T	1.9004	4.4441	0.6700	0.0491	0.0252	0.0510	0.0269561	0.0501705	0.59	-0.0123201	0.0121205	0.31
rs1772499	785851	19	18454825	A	G				rs17724992	A	G	1.3006	1.1697	0.2700	0.0055	0.0067	0.4100	-0.0124483	0.0133418	0.35	-0.00208125	0.00323863	0.52
rs1773929	692334	6	27293049	C	G	*	rs55797265	0.99	rs55797265	T	C	1.1746	1.9629	0.5500	-0.0068	0.0112	0.5400	0.0610384	0.0223672	0.0064	-0.0042869	0.00539341	0.43
rs1775797	690716	6	38214150	T	C				rs17757975	C	T	2.6327	5.8037	0.6500	-0.0020	0.0329	0.9500	-0.0325984	0.0654809	0.62	-0.0139399	0.0158428	0.38
rs1779593	794950	16	51926509	C	T				rs17795934	T	C	1.7599	1.2969	0.1700	0.0005	0.0074	0.9400	0.0208849	0.0148403	0.16	0.000160952	0.00360384	0.96
rs1780637	690043	20	51107290	C	T				rs17806379	T	C	1.9170	1.6974	0.2600	0.0166	0.0097	0.0860	0.0268482	0.0192744	0.16	-0.00148674	0.00466272	0.75
rs1782082	690065	9	11831420	T	G	*	rs10809628	0.64	rs10809628	T	A	1.6928	1.7489	0.3300	0.0060	0.0100	0.5500	-0.0271055	0.019844	0.17	-0.00105773	0.00481661	0.83
rs1782507	692310	11	30243868	G	T	*																	
rs1787267	692092	18	76742544	G	C				rs1787267	C	G	1.5246	2.5921	0.5600	0.0168	0.0148	0.2600	-0.0023761	0.0295468	0.94	-0.0122642	0.00710866	0.084
rs1789165	775128	11	69481969	A	G	*																	
rs1791253	684367	18	37103550	T	G				rs1791253	T	G	1.1833	1.3317	0.3700	-0.0108	0.0076	0.1600	0.0036985	0.0151773	0.81	0.00182077	0.00368665	0.62
rs1814170	686628	7	138794149	A	T	*	rs62485917	0.83	rs62485917	T	C	1.6910	1.2272	0.1700	0.0014	0.0070	0.8400	-0.0123948	0.0140003	0.38	0.0052798	0.00340081	0.12
rs181732	686436	3	136744386	G	T	*	rs900949	0.72	rs900949	G	T	0.7555	1.3948	0.5900	-0.0019	0.0080	0.8100	-0.0206407	0.0158998	0.19	-0.00352612	0.00386863	0.36
rs1819844	793238	12	68205604	A	G				rs1819844	A	G	0.6588	1.3913	0.6400	-0.0130	0.0079	0.1000	-0.0160644	0.0158398	0.31	0.00315371	0.00386064	0.41
rs1852006	692290	7	77829768	G	A				rs1852006	G	A	2.0059	1.2060	0.0960	-0.0024	0.0069	0.7300	0.00441287	0.0137576	0.75	-0.00239104	0.00332187	0.47
rs1853639	673714	6	87606842	G	A				rs1853639	A	G	0.2126	1.2033	0.8600	-0.0088	0.0069	0.2000	-0.0021201	0.0137274	0.88	-0.00249589	0.00331676	0.45
rs1862451	691653	16	24803620	A	G	*	rs964782	1.00	rs964782	C	T	1.9156	1.7896	0.2800	-0.0191	0.0102	0.0600	-0.0106985	0.0203243	0.6	-0.00644103	0.00499796	0.2
rs1865341	688863	9	8845911	T	C				rs1865341	T	C	0.1013	1.6210	0.9500	-0.0165	0.0093	0.0750	-0.0249319	0.018477	0.18	0.00069718	0.00450077	0.88
rs186543	682722	5	86754835	G	C				rs186543	G	C	1.3662	1.2040	0.2600	-0.0003	0.0069	0.9600	0.00351052	0.0137465	0.8	-0.00040633	0.00334107	0.9
rs1866510	691901	4	44514468	C	T				rs1866510	T	C	1.1941	1.1885	0.3200	-0.0088	0.0068	0.1900	0.0026815	0.0135391	0.84	0.00167183	0.00328862	0.61
rs1884389	683669	20	1410582	C	T	*																	
rs1884897	789534	20	6612832	G	A	*																	
rs1899689	794654	7	121964349	T	C				rs1899689	C	T	1.3990	1.2201	0.2500	-0.0025	0.0070	0.7200	-0.0217823	0.013956	0.12	-0.00097667	0.00339393	0.77
rs1899951	795281	3	12394840	T	C				rs1899951	T	C	1.6194	2.7796	0.5600	-0.0159	0.0160	0.3200	-0.0188294	0.0317846	0.55	0.0139005	0.0077306	0.072
rs1903579	690564	4	91253956	C	G	*	rs10008665	1.00	rs10008665	G	A	0.1643	1.5185	0.9100	-0.0062	0.0087	0.4700	0.00173127	0.017352	0.92	0.00125514	0.00418173	0.76
rs1909586	676854	3	124687767	G	T				rs1909586	T	G	0.2394	1.3670	0.8600	-0.0048	0.0078	0.5400	-0.0174323	0.0155577	0.26	-0.00216702	0.00376164	0.56
rs1927790	794326	13	96922191	C	T				rs1927790	C	T	0.3558	1.2589	0.7800	-0.0010	0.0072	0.8900	0.00193748	0.0143292	0.89	-0.00066312	0.00348067	0.85
rs1928295	793649	9	120378483	T	C				rs1928295	C	T	0.4114	1.2106	0.7300	-0.0023	0.0069	0.7400	0.00545397	0.0137773	0.69	0.00347819	0.00334393	0.3
rs1937684	690063	10	53680085	A	T				rs1937684	A	T	0.4891	1.2248	0.6900	0.0050	0.0070	0.4700	0.0102525	0.0140023	0.46	-0.00483621	0.00341654	0.16
rs1941697	692446	18	31251276	A	G	*	rs12456504	0.74	rs12456504	G	T	0.9330	1.4137	0.5100	-0.0182	0.0081	0.0240	-0.0253471	0.0161133	0.12	-0.00438801	0.00391815	0.26
rs1942866	781260	18	57741783	G	C	*	rs8092496	0.67	rs8092496	A	G	1.9191	1.5223	0.2100	0.0110	0.0087	0.2100	0.0107176	0.0173471	0.54	-0.00248016	0.00419022	0.55
rs1943477	793263	11	56973793	C	T				rs1943477	T	C	1.1875	5.1266	0.8200	0.0138	0.0290	0.6400	0.0242816	0.0583324	0.68	-0.00253799	0.0141355	0.86
rs1951455	690040	14	91512339	C	T				rs1951455	T	C	1.9691	1.5926	0.2200	0.0175	0.0091	0.0530	0.00192015	0.0181003	0.92	0.00333795	0.00439303	0.45
rs1956151	692204	14	40101060	G	A				rs1956151	G	A	1.3631	1.2976	0.2900	0.0100	0.0074	0.1800	0.0143997	0.0147815	0.33	-0.00302196	0.0036097	0.4
rs1973993	692549	1	96943994	C	T	*																	
rs1982441	687705	8	28021769	T	G	*																	
rs1983864	692553	10	100017453	T	G				rs1983864	T	G	0.8342	1.2183	0.4900	0.0111	0.0070	0.1100	-0.0025806	0.0139213	0.85	0.000841151	0.00337703	0.8
rs2000746	675837	18	57677294	A	G	*																	
rs2003616	684693	14	79903993	T	G	*	rs17758369	0.89	rs17758369	A	G	2.1370	1.5674	0.1700	0.0053	0.0089	0.5500	-0.0044442	0.0178475	0.8	-0.0101791	0.00432055	0.018
rs200968	795566	6	27859568	C	T				rs200968	T	C	2.0687	2.3118	0.3700	0.0090	0.0132	0.4900	-0.0376991	0.0263527	0.15	-0.00022236	0.00639909	0.97
rs2010281	794009	14	103862322	G	A				rs2010281	A	G	2.1179	1.6960	0.2100	0.0057	0.0097	0.5500	0.0103628	0.0192979	0.59	-0.00484911	0.00471154	0.3
rs2012927	792270	18	63297672	A	G				rs2012927	A	G	0.6455	1.2271	0.6000	0.0002	0.0070	0.9800	0.00773919	0.0140114	0.58	-0.00321842	0.00340346	0.34
rs2030342	686857	1	97388226	T	C	*	rs6671669	0.90	rs6671669	G	A	1.2415	1.2445	0.3200	0.0068	0.0071	0.3400	0.00716121	0.0141892	0.61	-0.00254713	0.00344281	0.46
rs2033529	792112	6	40348653	G	A	*																	
rs2047632	688132	3	37153431	G	C	*																	
rs2075205	691296	16	54153099	A	T				rs2075205	A	T	1.4172	1.1894	0.2300	0.0040	0.0068	0.5600	-0.0117602	0.0136026	0.39	0.00182265	0.00330059	0.58
rs2075650	779741	19	45395619	A	G				rs2075650	A	G	1.2352	2.0914	0.5500	-0.0004	0.0120	0.9700	0.00533231	0.024019	0.82	0.00984045	0.00578888	0.089
rs208015	691575	17	46252346	T	C				rs208015	T	C	0.2308	1.6316	0.8900	-0.0082	0.0093	0.3800	-0.0173716	0.0186445	0.35	0.00552118	0.00450452	0.22
rs2080454	784784	16	49062590	C	A	*																	
rs2098618	657277	5	87934851	T	A	*	rs59782145	0.93	rs59782145	T	C	1.4584	5.7378	0.8000	0.0291	0.0325	0.3700	-0.001662	0.0647604	0.98	-0.0010267	0.0168308	0.95
rs2100814	683658	8	28118130	A	G				rs2100814	A	G	0.8009	1.2041	0.5100	-0.0016	0.0069	0.8200	-0.0002318	0.0137467	0.99	0.0019834	0.00334001	0.55
rs2122042	793383	3	85866335	T	G	*																	
rs2124499	785955	3	123093541	G	C	*	rs12330631	0.99	rs12330631	C	T	2.4110	1.3031	0.0640	-0.0037	0.0074	0.6200	-0.016981	0.0148666	0.25	-0.00363004	0.00360875	0.31
rs2140418	631778	6	34975415	T	C	*																	
rs2143253	684760	20	41987392	G	A				rs2143253	G	A	0.1909	1.3175	0.8800	-0.0072	0.0075	0.3400	0.0166015	0.0150249	0.27	-0.00068322	0.0036477	0.85
rs2154297	690841	1	80791708	C	T	*	rs2154298	1.00	rs2154298	G	A	1.9508	1.3583	0.1500	0.0068	0.0078	0.3800	0.0159333	0.0155098	0.3	0.00523323	0.00375971	0.16
rs215632	684437	7	32368524	A	G				rs215632	A	G	0.4010	1.2064	0.7400	-0.0080	0.0069	0.2500	-0.0088029	0.0137794	0.52	0.00537907	0.00332746	0.11
rs2160077	795067	14	92428410	A	G				rs2160077	A	G	0.8897	1.3636	0.5100	0.0261	0.0078	0.0008	-0.009928	0.0155371	0.52	-0.00257457	0.00379285	0.5
rs2163188	686502	10	65314711	C	G	*	rs7919685	1.00	rs7919685	T	G	0.2485	1.2738	0.8500	0.0021	0.0073	0.7700	0.00614837	0.0145013	0.67	0.00650374	0.00351676	0.064
rs2170382	690485	8	74689288	T	C				rs2170382	C	T	15.8963	5.1312	0.0019	0.0198	0.0290	0.5000	-0.0102953	0.0578913	0.86	0.0286804	0.0140281	0.041
rs2174307	686559	9	73791849	C	G				rs2174307	C	G	0.1454	1.2276	0.9100	0.0029	0.0070	0.6800	-0.0243951	0.0140037	0.082	0.00284027	0.00340149	0.4
rs217671	691456	14	62360464	G	A				rs217671	G	A	0.6111	1.2024	0.6100	-0.0030	0.0069	0.6700	-0.0044725	0.0137655	0.75	-0.00333831	0.00334212	0.32
rs2185027	791606	6	153381622	C	A				rs2185027	C	A	2.8292	1.2231	0.0210	-0.0075	0.0070	0.2800	0.00085202	0.013928	0.95	0.00310624	0.0033639	0.36
rs2190788	683650	5	144484261	T	G				rs2190788	T	G	1.8311	1.9694	0.3500	0.0170	0.0112	0.1300	-0.0089271	0.0224162	0.69	-0.00194713	0.00543262	0.72
rs2192158	690727	4	55505360	A	G				rs2192158	G	A	0.7633	1.1939	0.5200	0.0055	0.0068	0.4200	-0.0174817	0.0135897	0.2	0.00228297	0.0033033	0.49
rs2194385	684190	2	50865334	A	C				rs2194385	A	C	0.2285	1.2725	0.8600	0.0018	0.0073	0.8000	-0.0032614	0.0145194	0.82	-0.0015286	0.0035448	0.67
rs2228213	795595	6	12124855	G	A				rs2228213	G	A	0.6889	1.4001	0.6200	-0.0018	0.0080	0.8200	-0.0231967	0.0159326	0.15	-0.00249233	0.00385335	0.52
rs2229616	760183	18	58039276	C	T				rs2229616	T	C	1.5964	4.1082	0.7000	0.0594	0.0235	0.0120	-0.0189541	0.0473324	0.69	-0.0121869	0.0115996	0.29
rs223051	689456	11	32131303	T	C				rs223051	T	C	2.0814	2.3077	0.3700	-0.0042	0.0132	0.7500	0.02159	0.0262419	0.41	-0.00315822	0.00638341	0.62
rs2230590	692189	3	49936102	C	T	*	rs9848497	1.00	rs9848497	C	T	0.4668	1.6580	0.7800	0.0069	0.0095	0.4700	-0.0179717	0.0189982	0.34	0.00497842	0.0045563	0.27
rs2230929	637221	3	49343175	G	A	*	rs56038006	1.00	rs56038006	C	T	5.8472	3.3049	0.0770	0.0071	0.0189	0.7100	-0.0076284	0.0376774	0.84	-0.0031217	0.00913388	0.73
rs2236950	692366	3	50420554	C	A	*																	
rs2240108	684395	12	48180508	C	T				rs2240108	T	C	3.0817	5.5664	0.5800	0.0675	0.0321	0.0360	-0.0049968	0.0640717	0.94	-0.0163752	0.0158256	0.3
rs2252300	772366	6	51096424	T	A	*																	
rs2275426	795468	1	46487552	A	G				rs2275426	G	A	1.4260	1.2199	0.2400	0.0070	0.0070	0.3200	-0.0177596	0.0139279	0.2	-0.00106741	0.00337656	0.75
rs2282231	689443	1	39569571	T	C	*	rs72660086	0.70	rs72660086	G	T	1.5923	1.5086	0.2900	0.0019	0.0086	0.8300	0.00664567	0.0171418	0.7	-0.00114817	0.00420818	0.78
rs2283093	691773	7	126721231	T	C				rs2283093	T	C	0.0397	1.4838	0.9800	0.0048	0.0085	0.5700	-0.007866	0.0169885	0.64	-0.00455938	0.00410455	0.27
rs2293605	668940	3	184044433	C	T				rs2293605	C	T	10.9601	3.0463	0.0003	0.0121	0.0173	0.4900	-0.023788	0.0346598	0.49	-0.0033369	0.00830695	0.69
rs2295896	793034	6	50566383	G	A				rs2295896	A	G	0.8881	1.2302	0.4700	0.0053	0.0070	0.4500	0.0052018	0.0139923	0.71	-0.00040771	0.00339586	0.9
rs2304130	783847	19	19789528	A	G				rs2304130	A	G	0.6191	1.7971	0.7300	-0.0043	0.0103	0.6800	0.0149975	0.020455	0.46	-0.00298233	0.00495446	0.55
rs2304607	684918	5	87988733	G	A				rs2304607	A	G	1.7456	1.3338	0.1900	0.0158	0.0076	0.0380	-0.0039038	0.0151941	0.8	0.000642974	0.00369046	0.86
rs2307022	795159	16	68381978	A	G				rs2307022	A	G	2.0805	1.5657	0.1800	-0.0193	0.0089	0.0310	-0.0158013	0.0178975	0.38	-0.00453068	0.00436903	0.3

rs2307111	795430	5	75003678	T	C	*	rs9942416	0.62	rs9942416	G	C	0.5931	1.2018	0.6200	0.0009	0.0069	0.9000	-0.0034474	0.0137438	0.8	0.000415391	0.00332326	0.9
rs2356865	691741	1	50836334	C	T	*																	
rs2357760	791053	6	120213880	A	G	*	rs6927250	0.92	rs6927250	G	T	2.3566	1.3193	0.0740	-0.0092	0.0075	0.2200	-0.0011002	0.0150344	0.94	-0.00488872	0.00361918	0.18
rs2365389	783625	3	61236462	C	T	*	rs13099397	0.66	rs13099397	C	A	2.2876	1.5920	0.1500	0.0045	0.0091	0.6200	-0.0491902	0.0181367	0.0067	0.00638888	0.00439522	0.15
rs2367112	794305	5	64168193	T	G				rs2367112	T	G	0.1690	1.6928	0.9200	0.0008	0.0096	0.9300	0.0132049	0.0192347	0.49	-0.00355821	0.00466895	0.45
rs2386802	688877	20	25971327	C	A				rs2386802	A	C	0.7160	1.2258	0.5600	-0.0020	0.0070	0.7700	0.0115102	0.0140033	0.41	-0.00012031	0.00337749	0.97
rs2397061	688481	6	51638877	C	T				rs2397061	T	C	1.0382	1.8921	0.5800	0.0100	0.0108	0.3500	-0.0004816	0.0215922	0.98	-0.00507038	0.00521564	0.33
rs2409730	788962	8	11060638	C	A	*																	
rs2412107	686465	14	65426216	T	G				rs2412107	G	T	0.6734	1.1986	0.5700	0.0050	0.0068	0.4700	0.00163824	0.0136724	0.9	0.00325239	0.00332036	0.33
rs2425241	689885	20	35018412	C	T	*																	
rs2425857	690825	20	44914134	A	G				rs2425857	G	A	0.1381	1.2489	0.9100	0.0082	0.0071	0.2500	0.021011	0.0142532	0.14	0.000971202	0.00346027	0.78
rs2429150	686276	12	2152655	C	A				rs2429150	C	A	1.1404	1.3127	0.3800	-0.0138	0.0075	0.0660	0.00827123	0.0149832	0.58	0.00841803	0.0036384	0.021
rs2440885	689699	11	70563286	A	G				rs2440885	A	G	1.1672	1.2976	0.3700	-0.0034	0.0074	0.6500	0.00286676	0.0148187	0.85	-0.00337468	0.00357998	0.35
rs2459359	677935	15	76863838	C	G	*																	
rs2467110	684290	12	23067302	C	T				rs2467110	C	T	0.6952	1.3892	0.6200	-0.0032	0.0079	0.6900	-0.0359705	0.0158345	0.023	-0.00464724	0.00385739	0.23
rs2479958	664268	13	111984244	A	G	*																	
rs248139	683675	5	167352783	A	G				rs248139	A	G	0.9133	1.3294	0.4900	-0.0144	0.0076	0.0580	-0.0243989	0.0151537	0.11	0.0038079	0.0036774	0.3
rs2481665	795247	1	62594677	T	C	*	rs17379217	0.72	rs17379217	T	C	1.1012	1.7111	0.5200	-0.0160	0.0098	0.1000	-0.0161912	0.0196112	0.41	0.00151336	0.00475389	0.75
rs2491864	690625	1	242986063	A	G				rs2491864	A	G	1.4790	1.2002	0.2200	-0.0067	0.0069	0.3300	0.00565907	0.0136991	0.68	0.00281189	0.00333899	0.4
rs2504674	690877	6	51160682	G	C				rs2504674	G	C	1.4657	1.1994	0.2200	-0.0043	0.0069	0.5300	0.0163949	0.0137161	0.23	0.00112549	0.00330969	0.73
rs2516739	761216	16	2097158	G	A				rs2516739	G	A	3.9276	2.9875	0.1900	0.0151	0.0171	0.3800	0.0324111	0.0341558	0.34	0.009497	0.00820204	0.25
rs2534760	677641	16	6509009	T	A	*	rs1935397	0.99	rs1935397	C	T	0.6697	1.3652	0.6200	-0.0074	0.0078	0.3400	-0.0024386	0.0155499	0.88	-0.00337443	0.00376571	0.37
rs2537847	689643	17	65694355	G	A	*	rs2625567	0.64	rs2625567	T	C	1.8397	1.6917	0.2800	-0.0052	0.0097	0.5900	-0.0336382	0.0193711	0.082	0.00806512	0.00467071	0.084
rs2543132	688326	8	15536311	C	G				rs2543132	C	G	1.0961	1.2028	0.3600	0.0011	0.0069	0.8800	0.0219674	0.0137	0.11	0.00128325	0.00332236	0.7
rs254428	774996	5	141777439	T	G				rs254428	G	T	0.5888	1.2555	0.6400	0.0033	0.0072	0.6400	0.0110845	0.0142958	0.44	0.00690754	0.00345447	0.046
rs25832	692083	5	66175682	A	G				rs25832	G	A	0.5732	1.1869	0.6300	0.0037	0.0068	0.5900	0.0169444	0.0135008	0.21	0.00292875	0.00326252	0.37
rs2590942	793971	1	72885281	T	G				rs2590942	G	T	1.6602	4.5727	0.7200	0.0336	0.0262	0.2000	-0.035271	0.0523054	0.5	-0.0205686	0.0129551	0.11
rs2593280	688016	15	76150965	A	G				rs2593280	A	G	0.0512	1.7654	0.9800	0.0019	0.0101	0.8500	0.0520178	0.0202044	0.01	0.0040097	0.00483908	0.41
rs2605603	790857	11	93221105	G	A				rs2605603	G	A	0.1868	1.5626	0.9000	0.0018	0.0089	0.8400	0.0117395	0.0177525	0.51	0.000730212	0.0043285	0.87
rs2611742	691646	5	95856501	C	T				rs2611742	T	C	0.2612	1.2222	0.8300	0.0067	0.0070	0.3400	-0.0039069	0.0139666	0.78	0.00622039	0.00338352	0.066
rs2612203	515871	11	54914689	A	G	*																	
rs2619976	684417	17	71754545	T	C				rs2619976	C	T	1.3782	1.3066	0.2900	0.0033	0.0075	0.6600	0.0210027	0.0148915	0.16	0.0032936	0.00361186	0.36
rs262956	686884	3	183486117	T	G	*																	
rs2631681	789736	10	93032943	C	T	*	rs2450445	0.99	rs2450445	G	A	1.3293	2.0470	0.5200	-0.0017	0.0117	0.8900	-0.0258613	0.0233137	0.27	0.00813643	0.00567094	0.15
rs2634047	683222	8	85696337	G	C	*	rs2634045	0.87	rs2634045	G	A	4.2904	1.7809	0.0160	-0.0050	0.0102	0.6300	-0.0128554	0.0204121	0.53	3.29931E-05	0.00494081	0.99
rs2635727	792938	6	50820940	C	T	*	rs2817419	0.88	rs2817419	G	A	2.0576	1.4372	0.1500	0.0024	0.0082	0.7700	0.03212	0.0163598	0.05	-0.00342205	0.00399054	0.39
rs2653365	689811	6	55169801	C	T				rs2653365	T	C	0.3066	1.8205	0.8700	-0.0188	0.0104	0.0710	0.00590912	0.0207483	0.78	0.00264579	0.00497437	0.59
rs2680648	686125	3	53777176	T	C				rs2680648	T	C	0.4194	1.2956	0.7500	0.0083	0.0074	0.2600	-0.0186752	0.0147475	0.21	-0.00186725	0.00358255	0.6
rs2710323	790805	3	52815905	C	T				rs2710323	C	T	0.9875	1.2297	0.4200	0.0017	0.0070	0.8100	0.0527679	0.0140004	0.00016	0.00485374	0.0034028	0.15
rs2721965	690170	8	116662038	A	C	*	rs2737251	1.00	rs2737251	C	T	2.0655	1.2353	0.0950	0.0006	0.0071	0.9300	-0.0212835	0.0141029	0.13	0.00452502	0.00342578	0.19
rs2724861	780412	2	471514	G	A				rs2724861	A	G	1.0857	1.2517	0.3900	0.0032	0.0072	0.6500	0.00240872	0.0142729	0.87	0.00142529	0.00345043	0.68
rs2731222	690932	12	90595383	A	C	*	rs2579115	0.94	rs2579115	G	T	3.6181	1.2026	0.0026	-0.0021	0.0069	0.7600	-0.0009058	0.0137116	0.95	0.000156793	0.00330685	0.96
rs2733287	681730	12	41880909	C	A				rs2733287	A	C	0.7392	1.2217	0.5500	-0.0013	0.0070	0.8500	-0.0149075	0.0139339	0.28	0.00069277	0.00335987	0.84
rs273504	690672	19	18215247	G	A				rs273504	A	G	0.0514	1.2544	0.9700	0.0009	0.0072	0.9000	-0.0054043	0.0143104	0.71	0.00414647	0.003487	0.23
rs273697	683992	18	23178748	G	A				rs273697	A	G	0.3000	1.1883	0.8000	-0.0017	0.0068	0.8100	0.0171223	0.0135432	0.21	0.000923552	0.00329836	0.78
rs2768950	754826	4	49064487	A	G	*	rs7655341	0.91	rs7655341	T	C	0.3356	1.2316	0.7900	-0.0018	0.0070	0.8000	0.00424686	0.0140587	0.76	0.00195144	0.0034046	0.57
rs2781668	678717	6	131897278	T	C				rs2781668	C	T	1.9381	1.3319	0.1500	0.0120	0.0076	0.1200	-0.0018627	0.015242	0.9	0.00306728	0.00373879	0.41
rs2814992	794002	6	34617144	G	A				rs2814992	A	G	0.3966	1.1890	0.7400	-0.0009	0.0068	0.9000	-0.001494	0.0135658	0.91	0.00214313	0.0032982	0.52
rs2820311	691876	1	201841476	G	A				rs2820311	A	G	0.7244	1.9369	0.7100	-0.0139	0.0111	0.2100	-0.0045768	0.0221955	0.84	-0.00047952	0.00538723	0.93
rs28350	686689	3	42418446	A	G				rs28350	G	A	3.1834	2.5761	0.2200	-0.0023	0.0147	0.8700	0.00229609	0.0292644	0.94	-0.00332361	0.00706157	0.64
rs2836961	793481	21	40627020	C	A				rs2836961	A	C	0.3552	1.3628	0.7900	0.0095	0.0078	0.2300	0.00943168	0.0156154	0.55	-0.0018667	0.00380719	0.62
rs2838006	686586	21	42653567	C	T	*	rs1041448	0.99	rs1041448	A	G	2.8832	1.7511	0.1000	0.0099	0.0100	0.3200	-0.000151	0.0200458	0.99	-0.00421788	0.00483462	0.38
rs284227	792227	1	82379446	C	T				rs284227	T	C	2.2386	2.3994	0.3500	-0.0169	0.0136	0.2100	-0.0485262	0.0271147	0.074	-0.00342159	0.00652208	0.6
rs2842385	689031	6	19078274	G	A				rs2842385	G	A	0.2667	1.5052	0.8600	0.0092	0.0086	0.2900	-0.0178436	0.0171848	0.3	0.00320211	0.00419356	0.45
rs2850969	691825	4	102183594	C	T				rs2850969	T	C	0.5177	1.1862	0.6600	-0.0066	0.0068	0.3300	-0.0030767	0.0135285	0.82	-0.00147724	0.00328809	0.65
rs2863981	794282	16	68295598	A	G	*																	
rs2866816	688980	19	30683879	T	C				rs2866816	T	C	1.4176	1.2024	0.2400	0.0069	0.0069	0.3200	-0.0111797	0.0137108	0.41	0.00243793	0.00331588	0.46
rs2875762	685199	6	124925032	C	G				rs2875762	C	G	0.8743	1.3343	0.5100	0.0067	0.0077	0.3800	0.0225166	0.0153128	0.14	0.0079152	0.00370669	0.033
rs2890652	674785	2	142959931	C	T				rs2890652	C	T	2.8452	7.6756	0.7100	0.0188	0.0435	0.6700	-0.0787759	0.0866501	0.36	0.0277158	0.0211036	0.19
rs2902021	677609	2	67825685	T	C				rs2902021	C	T	0.4781	1.8552	0.8000	-0.0124	0.0107	0.2500	0.00436524	0.0213031	0.84	-0.00834706	0.0052262	0.11
rs293566	691126	20	31097877	C	T	*																	
rs2974255	778107	2	516696	A	G	*																	
rs29938	788372	19	34311481	C	T	*	rs29937	0.84	rs29937	T	G	0.1333	1.2533	0.9200	0.0097	0.0072	0.1800	0.0181615	0.0143023	0.2	-0.00252777	0.00348076	0.47
rs310618	709614	20	62127121	C	T	*																	
rs3134353	679869	8	101947453	T	A	*	rs1470764	1.00	rs1470764	A	G	0.9868	1.2762	0.4400	0.0019	0.0073	0.7900	-0.004921	0.0145633	0.74	-0.00749574	0.00353747	0.034
rs3134438	685121	11	122765667	A	C				rs3134438	C	A	1.1855	1.2293	0.3300	0.0115	0.0070	0.1000	-0.011409	0.0139949	0.41	0.00186468	0.00340294	0.58
rs316611	677116	15	41751678	T	C	*	rs1757457	0.61	rs1757457	T	C	2.4303	4.1421	0.5600	0.0268	0.0237	0.2600	-0.131004	0.0475777	0.0059	-0.0073759	0.0116694	0.53
rs326889	688030	4	112713436	C	T				rs326889	C	T	0.1457	1.1843	0.9000	0.0101	0.0068	0.1400	-0.0260822	0.0135231	0.054	0.00287303	0.00327703	0.38
rs329124	690757	5	133865452	A	G				rs329124	G	A	1.3255	1.2277	0.2800	-0.0047	0.0070	0.5000	-0.0030958	0.0139981	0.82	-0.00179294	0.00337882	0.6
rs329277	686149	7	35080931	G	T				rs329277	G	T	0.0307	1.2779	0.9800	0.0054	0.0073	0.4600	0.00860354	0.0145753	0.56	0.00103923	0.00353413	0.77
rs329651	779232	11	133767622	T	G	*																	
rs331949	787844	4	143663206	C	T	*	rs1443187	0.66	rs1443187	G	A	2.2281	1.4491	0.1200	-0.0084	0.0083	0.3100	-0.0235571	0.0165013	0.15	0.000897418	0.00398819	0.82
rs340025	786830	15	60908307	C	T	*	rs340019	0.82	rs340019	A	G	0.4645	1.2906	0.7200	0.0045	0.0074	0.5400	-0.0052447	0.0146796	0.72	-0.00329376	0.00353967	0.35
rs346722	521901	1	45620134	C	T				rs346722	C	T	1.5588	1.5601	0.3200	-0.0124	0.0089	0.1600	-0.0063852	0.0177411	0.72	-0.00901027	0.00432263	0.037
rs355777	689978	3	154034950	C	G				rs355777	G	C	0.8764	1.1816	0.4600	-0.0081	0.0068	0.2300	0.00176457	0.0134676	0.9	-0.00209683	0.00327479	0.52
rs368863	683807	5	77372852	C	T				rs368863	T	C	1.7667	2.5355	0.4900	-0.0222	0.0144	0.1200	-0.0340637	0.0287446	0.24	-0.00781355	0.00708125	0.27
rs3731544	776530	3	48207997	A	C	*																	
rs3734572	786251	6	28059458	C	T				rs3734572	C	T	3.3412	6.7190	0.6200	0.0102	0.0382	0.7900	-0.0164958	0.0761104	0.83	0.0334173	0.0186378	0.073
rs3736485	790404	15	51748610	A	G				rs3736485	G	A	2.2698	1.5469	0.1400	-0.0015	0.0088	0.8700	-0.0087977	0.0176837	0.62	0.00364948	0.00430024	0.4
rs3739555	794521	9	129940416	T	G				rs3739555	G	T	1.5176	5.5719	0.7900	-0.0122	0.0319	0.7000	0.0487739	0.0641885	0.45	-0.00377248	0.0153376	0.81
rs3739733	637547	9	88897891	A	G				rs3739733	G	A	0.5424	1.1950	0.6500	-0.0067	0.0068	0.3300	0.00317778	0.0136238	0.82	0.00226023	0.00328917	0.49
rs3746429	692582	20	33703607	T	C				rs3746429	C	T	0.8092	2.0102	0.6900	0.0105	0.0114	0.3600	0.0136449	0.0228142	0.55	0.00502787	0.00557599	0.37
rs3751813	781755	16	53818708	T	G	*	rs7201850	0.66	rs7201850	T	C	1.2173	1.5171	0.4200	0.0040	0.0086	0.6400	-0.0086636	0.0172659	0.62	0.000031638	0.00416698	0.99
rs3753549	691062	1	243722892	C	T				rs3753549	T	C	2.4392	1.3152	0.0640	0.0076	0.0075	0.3100	0.0080265	0.0150075	0.59	0.00296059	0.00362463	0.41
rs3762396	795206	1	19510394	A	G	*	rs10799666	0.87	rs10799666	A	T	0.8591	1.4317	0.5500	0.0006	0.0082	0.9400	-0.023734	0.0163333	0.15	0.00385714	0.0039527	0.33
rs3768486	793120	1	110123971	G	A				rs3768486	G	A	0.9486	1.2497	0.4500	-0.0080	0.0072	0.2700	0.0189307	0.014287	0.19	-0.00085531	0.00345319	0.8
rs3772934	674038	3	115417865	T	C	*	rs3772935	0.92	rs3772935	A	G	2.6349	1.4547	0.0700	-0.0085	0.0083	0.3100	-0.0174248	0.0165818	0.29	0.006327	0.00400452	0.11

rs3781099	692416	10	27318776	T	C				rs3781099	T	C	1.0966	1.3402	0.4100	-0.0148	0.0076	0.0520	-0.0288484	0.0152803	0.059	-0.00289979	0.00369638	0.43
rs3794702	675189	16	3730613	A	T	*	rs67081976	0.83	rs67081976	G	A	0.3087	1.3622	0.8200	-0.0040	0.0078	0.6100	0.020848	0.0156204	0.18	0.00442046	0.00376836	0.24
rs3800229	792474	6	108996963	T	G				rs3800229	T	G	1.2559	1.2853	0.3300	0.0083	0.0073	0.2600	0.0229535	0.0146656	0.12	0.00260958	0.00352577	0.46
rs3800649	686803	7	137424509	A	G				rs3800649	A	G	0.2252	1.4636	0.8800	-0.0045	0.0084	0.5900	-0.0321802	0.0167283	0.054	0.00251534	0.00404287	0.53
rs3802924	670918	11	133827733	A	C				rs3802924	C	A	1.6255	2.9184	0.5800	-0.0166	0.0166	0.3200	0.00211823	0.0331219	0.95	0.00969603	0.00804188	0.23
rs3803286	691435	14	103246470	A	G				rs3803286	G	A	1.7149	1.2151	0.1600	-0.0027	0.0069	0.7000	0.00513439	0.0138891	0.71	-0.0085555	0.00337042	0.011
rs3807049	685765	6	35512955	C	T				rs3807049	C	T	1.0836	1.4719	0.4600	0.0089	0.0084	0.2900	0.03773	0.0167745	0.024	-0.00368924	0.00408778	0.37
rs3807566	687535	7	50564204	G	T				rs3807566	T	G	0.0809	1.2520	0.9500	-0.0058	0.0072	0.4200	0.0151101	0.0143191	0.29	-0.00271525	0.00347222	0.43
rs3808434	794701	8	116559435	A	G				rs3808434	A	G	0.1433	1.6339	0.9300	0.0170	0.0093	0.0680	0.0163093	0.0185992	0.38	0.0039384	0.00452953	0.38
rs3809272	570125	12	111800258	A	G	*																	
rs3810291	763296	19	47569003	A	G				rs3810291	A	G	1.0623	1.3142	0.4200	0.0189	0.0075	0.0120	0.0103696	0.0149929	0.49	0.00726795	0.00362207	0.045
rs3814883	685519	16	29994922	T	C				rs3814883	T	C	1.0194	1.2036	0.4000	0.0028	0.0069	0.6800	0.0100697	0.0137636	0.46	-0.00405629	0.00334521	0.23
rs3819299	758228	6	31322367	T	G	*	rs35267732	0.83	rs35267732	A	T	1.6669	2.3042	0.4700	0.0188	0.0132	0.1600	0.0298964	0.0263983	0.26	0.000218806	0.00645372	0.97
rs3821841	669848	3	52084040	C	T				rs3821841	C	T	0.2001	1.2537	0.8700	-0.0024	0.0072	0.7400	-0.0018319	0.0143513	0.9	-0.00401956	0.00347973	0.25
rs3822683	684509	5	96080883	A	G				rs3822683	A	G	1.7230	1.2014	0.1500	0.0020	0.0069	0.7700	-0.0074352	0.0136839	0.59	-0.0023389	0.00333022	0.48
rs3826705	690844	19	42637232	C	T				rs3826705	C	T	1.7867	1.7961	0.3200	-0.0208	0.0102	0.0430	0.0110147	0.0204538	0.59	0.00142106	0.00495064	0.77
rs3829849	793851	9	129390800	T	C	*	rs12377624	0.96	rs12377624	C	G	2.0434	1.8681	0.2700	-0.0077	0.0107	0.4700	-0.0104813	0.0212769	0.62	-0.00413363	0.0051953	0.43
rs3844598	690704	5	140992235	G	A	*																	
rs3849570	750266	3	81792112	A	C				rs3849570	C	A	0.1669	1.1919	0.8900	-0.0001	0.0068	0.9900	-0.0085202	0.0135916	0.53	0.00122721	0.00330981	0.71
rs3850422	782650	14	99671788	G	A				rs3850422	A	G	1.7122	2.7106	0.5300	0.0137	0.0154	0.3800	0.0408274	0.0308044	0.19	-0.0100868	0.0075083	0.18
rs3851083	793679	10	33862727	G	A				rs3851083	G	A	0.4805	1.1629	0.6800	0.0111	0.0066	0.0950	-0.0032311	0.013281	0.81	0.00385877	0.00321178	0.23
rs3887080	690624	12	121661966	A	G	*	rs75089355	0.79	rs75089355	G	A	1.6825	3.9582	0.6700	0.0032	0.0224	0.8900	0.0138889	0.0447349	0.76	-0.00395837	0.0110779	0.72
rs3902840	683761	9	129419025	A	G	*	rs61185870	0.87	rs61185870	T	C	0.0590	1.2533	0.9600	0.0079	0.0072	0.2700	0.00460834	0.0142694	0.75	0.000882121	0.00343592	0.8
rs3902951	773819	14	69789755	G	T				rs3902951	T	G	1.4909	1.1962	0.2100	-0.0164	0.0068	0.0170	0.00111544	0.0136705	0.93	-0.00291534	0.00331177	0.38
rs3915844	692229	3	9514856	A	G				rs3915844	A	G	1.2329	1.8491	0.5000	0.0002	0.0105	0.9800	-0.0012026	0.0210197	0.95	0.00224844	0.0051437	0.66
rs3922853	635146	2	5830599	A	C				rs3922853	A	C	1.7421	2.5018	0.4900	-0.0008	0.0143	0.9600	0.0436151	0.0284897	0.13	0.00361378	0.00692265	0.6
rs39654	687774	3	173095123	G	A				rs39654	A	G	0.1751	1.2439	0.8900	-0.0150	0.0071	0.0340	-0.000264	0.0142023	0.99	-0.00567914	0.00343507	0.098
rs40067	681695	5	107439012	G	A	*	rs149457	1.00	rs149457	C	T	2.5909	1.2157	0.0330	0.0051	0.0069	0.4600	-0.0009943	0.0138653	0.94	-0.00488447	0.00334738	0.14
rs40245	681704	7	21470536	A	T	*	rs12672629	0.79	rs12672629	A	G	2.3554	1.9510	0.2300	0.0182	0.0111	0.1000	-0.0047103	0.0223422	0.83	-0.00236297	0.00537202	0.66
rs403656	686605	15	76755506	A	G				rs403656	G	A	1.5361	1.7454	0.3800	0.0001	0.0100	0.9900	-0.0033201	0.0198997	0.87	-0.00418704	0.00479301	0.38
rs4076358	787955	8	144910239	A	G				rs4076358	A	G	0.4329	1.2560	0.7300	0.0000	0.0072	1.0000	0.00238061	0.0142914	0.87	-0.00284016	0.00346466	0.41
rs4077093	620584	12	51593616	T	G				rs4077093	T	G	1.7056	1.7801	0.3400	-0.0038	0.0102	0.7100	0.00206115	0.0202783	0.92	0.00219724	0.00491535	0.65
rs411717	691713	7	94033031	C	T				rs411717	T	C	0.8948	1.1974	0.4500	0.0063	0.0068	0.3500	-0.0111477	0.0136471	0.41	0.00304586	0.00330402	0.36
rs4148155	794889	4	89054667	A	G				rs4148155	A	G	1.7080	1.3036	0.1900	0.0061	0.0074	0.4100	-0.0033704	0.0148524	0.82	0.00422928	0.00357532	0.24
rs419261	792747	6	33554147	T	C				rs419261	T	C	1.5769	2.5047	0.5300	0.0021	0.0145	0.8800	0.00172536	0.0289022	0.95	-9.5919E-05	0.00705365	0.99
rs4273371	794363	3	108119071	C	T	*																	
rs4278019	691914	6	53693410	A	T				rs4278019	A	T	0.0890	1.2004	0.9400	0.0011	0.0069	0.8800	0.00275098	0.0137436	0.84	-0.00327623	0.00332737	0.32
rs427943	712095	21	46570896	C	A				rs427943	A	C	2.9631	1.2119	0.0140	0.0047	0.0069	0.5000	-0.005451	0.0138031	0.69	0.00279005	0.00334713	0.4
rs4284600	682852	15	31843528	C	T	*																	
rs4303732	691017	2	100830040	T	C	*	rs10496345	0.97	rs10496345	T	G	0.1258	1.1845	0.9200	0.0056	0.0068	0.4000	-0.0017855	0.0134952	0.89	-0.00059464	0.00326218	0.86
rs4307239	687289	7	24354300	G	A				rs4307239	A	G	1.1914	1.2758	0.3500	0.0015	0.0073	0.8400	-0.0002785	0.0145616	0.98	-0.0073858	0.00353746	0.037
rs4339513	528869	6	57964315	C	T				rs4339513	T	C	1.1968	1.4152	0.4000	0.0039	0.0081	0.6300	0.0141202	0.0161311	0.38	0.00137545	0.00392929	0.73
rs4342060	685895	3	137215820	C	T	*																	
rs4366093	688025	8	20639811	C	T	*																	
rs4372296	691179	1	98320492	C	A				rs4372296	A	C	6.1138	1.9962	0.0022	0.0065	0.0114	0.5700	0.0113718	0.0228476	0.62	-0.00372953	0.00543812	0.49
rs4372836	794417	2	28973883	T	C	*	rs4549034	0.99	rs4549034	T	C	0.5884	1.3314	0.6600	0.0079	0.0076	0.3000	-0.0009016	0.0152253	0.95	0.000527206	0.0036734	0.89
rs4383818	789791	6	50756951	T	G	*																	
rs450231	692346	9	101481205	G	A				rs450231	A	G	0.9754	1.2546	0.4400	0.0096	0.0072	0.1800	-0.0052018	0.0143024	0.72	-0.00013582	0.00347774	0.97
rs4515655	690151	9	128616073	C	T				rs4515655	C	T	2.7518	1.2217	0.0240	0.0081	0.0070	0.2500	0.00261521	0.0139413	0.85	0.00599739	0.00335833	0.074
rs4516268	786617	17	1846831	C	A				rs4516268	C	A	1.8943	1.5864	0.2300	-0.0047	0.0090	0.6000	0.0212419	0.0180463	0.24	0.000617146	0.00436533	0.89
rs4518345	688609	5	27185904	G	A				rs4518345	A	G	1.6776	2.0148	0.4100	-0.0172	0.0115	0.1300	-0.0341871	0.0229191	0.14	0.0120552	0.00558321	0.031
rs4524456	688219	4	6492739	G	A				rs4524456	A	G	0.4384	1.3897	0.7500	-0.0105	0.0080	0.1900	-0.0024498	0.0159055	0.88	-0.00123929	0.00387096	0.75
rs453520	689483	2	147907202	C	T	*	rs388614	0.99	rs388614	C	T	0.8400	1.3694	0.5400	0.0012	0.0078	0.8800	0.00417376	0.0155738	0.79	-0.00677963	0.00377422	0.072
rs4542429	507039	11	56446833	C	T				rs4542429	T	C	0.6499	1.2077	0.5900	0.0004	0.0069	0.9500	-0.0139234	0.0137735	0.31	0.000956497	0.00334033	0.77
rs4624596	691079	3	119571541	T	C				rs4624596	C	T	0.3517	1.1951	0.7700	-0.0035	0.0068	0.6100	0.0127186	0.0136444	0.35	-0.0043816	0.00330382	0.18
rs4639527	691706	2	416815	G	A				rs4639527	A	G	1.5569	1.2993	0.2300	-0.0009	0.0074	0.9100	0.0190424	0.0148508	0.2	-0.00320043	0.00361898	0.38
rs4643949	528920	5	138372345	C	T				rs4643949	C	T	1.4146	1.6118	0.3800	-0.0045	0.0092	0.6200	0.00301684	0.0184104	0.87	-0.00302242	0.00447572	0.5
rs4653017	686378	1	33776728	T	C				rs4653017	C	T	0.5124	1.3949	0.7100	-0.0051	0.0079	0.5200	-0.0044963	0.0158521	0.78	-0.00042217	0.0038331	0.91
rs4670626	681157	2	37046657	C	T	*																	
rs4671328	679487	2	58935282	T	G	*	rs1861410	0.96	rs1861410	C	T	1.7541	1.2277	0.1500	0.0083	0.0070	0.2400	0.0316753	0.014008	0.024	-0.00241201	0.00341184	0.48
rs4671358	688514	2	60164634	A	T	*	rs1559625	0.89	rs1559625	A	G	1.1839	1.1982	0.3200	-0.0011	0.0069	0.8800	-0.0182313	0.0136924	0.18	0.0016352	0.00331716	0.62
rs4673553	686078	2	211608379	G	T	*																	
rs4676084	685747	2	110010962	A	G	*	rs6739259	0.82	rs6739259	G	C	2.0435	1.7775	0.2500	-0.0019	0.0101	0.8500	0.00351548	0.0202457	0.86	-0.00540167	0.00491956	0.27
rs4678297	680675	3	136907111	T	C	*																	
rs4718966	686820	7	70040558	T	C				rs4718966	C	T	1.1832	1.2659	0.3500	-0.0100	0.0072	0.1600	0.0146561	0.0144287	0.31	-0.00565161	0.00349292	0.11
rs4722398	692509	7	3125220	T	C	*	rs1011189	0.89	rs1011189	G	C	4.9405	3.1267	0.1100	-0.0048	0.0177	0.7800	0.0484221	0.0353511	0.17	-0.0108391	0.00869576	0.21
rs4722672	784309	7	27231762	C	T	*																	
rs4725984	784486	7	150668514	C	T	*	rs3807370	0.67	rs3807370	A	G	1.4469	1.7972	0.4200	0.0149	0.0103	0.1500	0.018368	0.0205965	0.37	-0.00203915	0.00506703	0.69
rs4737183	685737	8	64720693	A	G	*	rs4737184	1.00	rs4737184	C	A	1.9759	1.2795	0.1200	0.0048	0.0073	0.5100	-0.0045733	0.0145684	0.75	0.00534729	0.00354341	0.13
rs4740383	662683	9	133783566	A	G	*	rs10751510	0.93	rs10751510	G	A	1.3925	1.2300	0.2600	0.0012	0.0070	0.8600	0.00138821	0.0140152	0.92	0.00309521	0.00339532	0.36
rs4744275	793296	9	96482633	A	G				rs4744275	A	G	0.4994	1.2009	0.6800	0.0082	0.0069	0.2300	0.0139629	0.0137336	0.31	-0.00141272	0.00332165	0.67
rs4759073	692071	12	54653258	G	A				rs4759073	A	G	0.8271	1.1985	0.4900	0.0069	0.0068	0.3100	-0.0067642	0.0136471	0.62	0.00132152	0.00330294	0.69
rs4759228	672778	12	56508409	G	C				rs4759228	C	G	1.7203	2.0093	0.3900	0.0076	0.0115	0.5100	0.0117617	0.0228739	0.61	0.00394488	0.00555861	0.48
rs4764949	691560	12	103658096	A	G	*	rs6539066	0.89	rs6539066	C	T	1.0703	1.2522	0.3900	0.0107	0.0072	0.1400	-0.0182729	0.0143348	0.2	3.87091E-05	0.00347473	0.99
rs4766710	691939	12	114437708	A	G	*																	
rs4783241	689741	16	82650384	G	C	*	rs8062260	0.85	rs8062260	A	G	0.2509	1.2552	0.8400	0.0011	0.0072	0.8700	0.023696	0.0143203	0.098	0.000823395	0.0034762	0.81
rs4796243	691686	17	35057883	G	A				rs4796243	A	G	0.7507	1.3985	0.5900	0.0059	0.0080	0.4600	-0.0020764	0.0159788	0.9	0.00137844	0.00385616	0.72
rs4814512	692559	20	16564210	A	C	*	rs6075079	0.98	rs6075079	T	C	1.4718	1.5453	0.3400	-0.0033	0.0089	0.7100	0.04162	0.0177223	0.019	0.00106638	0.00434034	0.81
rs4820408	794185	22	40604945	T	G	*																	
rs483752	515509	9	27612918	T	C				rs483752	C	T	1.6868	1.2152	0.1700	0.0009	0.0069	0.9000	-0.0099265	0.0138268	0.47	-0.00529392	0.00335905	0.12
rs4841659	687488	8	11828200	T	C	*																	
rs4854326	691927	2	603088	G	A	*	rs62104190	0.80	rs62104190	G	A	2.9437	1.2499	0.0190	0.0019	0.0071	0.7900	-0.0140902	0.0142349	0.32	0.00525872	0.00342866	0.13
rs4858193	686850	3	20441050	T	C				rs4858193	C	T	1.9821	2.1698	0.3600	0.0117	0.0124	0.3400	-0.0144524	0.0247582	0.56	-0.00212767	0.0059754	0.72
rs4864201	795263	4	130731284	T	C	*	rs11099020	0.95	rs11099020	T	C	1.7464	1.2687	0.1700	-0.0088	0.0073	0.2300	-0.0036165	0.0144958	0.8	-0.00528141	0.00351115	0.13
rs487060	789650	6	160774459	T	C				rs487060	T	C	0.7943	1.2854	0.5400	-0.0086	0.0073	0.2400	0.0114527	0.0145941	0.43	-0.00142215	0.00355164	0.69
rs4886506	691538	15	77207277	G	T	*	rs147629910	0.94	rs14762991	A	T	0.4239	1.1949	0.7200	-0.0068	0.0068	0.3200	0.029532	0.013612	0.03	-0.00731057	0.00330461	0.027
rs4886869	678149	15	77799657	G	A	*	rs11857450	0.80	rs11857450	G	C	1.7072	1.2475	0.1700	-0.0095	0.0071	0.1800	0.0198595	0.0141825	0.16	0.00212948	0.00342813	0.53

rs4889782	684598	17	78640510	C	T				rs4889782	C	T	1.2141	1.2614	0.3400	-0.0086	0.0072	0.2300	-0.0029902	0.0143802	0.84	7.65372E-06	0.00348401	1
rs4906908	691345	15	27040082	G	T				rs4906908	T	G	1.1940	1.2376	0.3300	0.0024	0.0071	0.7400	0.016342	0.0141531	0.25	-0.00422954	0.0034341	0.22
rs4916661	688639	5	87932809	G	T				rs4916661	G	T	0.5729	1.3644	0.6700	0.0044	0.0078	0.5700	-0.0023975	0.015529	0.88	-0.00999448	0.00374891	0.0077
rs4936175	692569	11	132641959	C	T	*																	
rs4936671	678888	11	121942512	C	G				rs4936671	C	G	2.0852	1.2975	0.1100	-0.0039	0.0074	0.6000	0.00526565	0.0147904	0.72	0.00150562	0.00357897	0.67
rs4939051	527251	11	56206141	G	C	*	rs11228592	0.99	rs11228592	G	A	1.6539	1.2226	0.1800	0.0039	0.0070	0.5700	7.5164E-05	0.0139682	1	-5.1973E-05	0.00338124	0.99
rs4969387	678051	17	79081724	G	C				rs4969387	G	C	1.5395	1.9079	0.4200	0.0104	0.0109	0.3400	-0.0043307	0.0217005	0.84	-0.0104737	0.00523886	0.046
rs497417	691046	8	9790041	T	A				rs497417	T	A	1.6109	1.3585	0.2400	0.0009	0.0078	0.9100	0.0192947	0.0154672	0.21	-0.00253867	0.00376074	0.5
rs4981693	689120	14	29680331	A	G	*	rs1569979	0.83	rs1569979	G	A	2.0513	1.2908	0.1100	0.0162	0.0074	0.0280	0.0124972	0.0147441	0.4	0.000793683	0.00357764	0.82
rs498240	679278	6	31892592	G	A	*	rs62395851	0.74	rs62395851	C	G	0.6927	6.2148	0.9100	0.0113	0.0356	0.7500	-0.0544764	0.0709941	0.44	-0.0119603	0.0172827	0.49
rs4985155	794269	16	15129459	A	G				rs4985155	A	G	1.1588	1.1744	0.3200	0.0081	0.0067	0.2300	-0.0142678	0.013421	0.29	-0.00296456	0.00325838	0.36
rs4986044	787219	17	21261560	C	T				rs4986044	C	T	1.7146	1.2162	0.1600	-0.0083	0.0070	0.2300	-0.0210154	0.0138808	0.13	0.00242153	0.00336802	0.47
rs524281	777942	11	65886662	C	A				rs524281	A	C	1.4284	1.1976	0.2300	0.0045	0.0068	0.5100	-0.0053894	0.0136634	0.69	-0.00384895	0.00330762	0.24
rs5396	785408	3	170744815	C	T				rs5396	T	C	0.4615	1.3968	0.7400	-0.0016	0.0080	0.8400	-0.0073795	0.0159603	0.64	0.00299297	0.0038396	0.44
rs543874	795504	1	177889480	G	A				rs543874	G	A	1.5510	1.8057	0.3900	-0.0044	0.0103	0.6700	0.0247266	0.0206106	0.23	-0.00020679	0.00503965	0.97
rs551137	529265	11	55323307	T	C				rs551137	C	T	1.9550	1.2557	0.1200	-0.0015	0.0072	0.8400	-0.011067	0.0143408	0.44	0.00304979	0.00346766	0.38
rs555267	788140	18	40992698	T	G	*																	
rs559267	794315	20	54157497	G	A				rs559267	G	A	1.5864	1.2211	0.1900	-0.0046	0.0070	0.5100	0.00030037	0.013932	0.98	-0.00147611	0.00338985	0.66
rs573455	795095	11	117267884	G	A	*	rs693147	0.76	rs693147	C	A	1.5702	1.2417	0.2100	0.0033	0.0071	0.6400	-0.0040405	0.0142162	0.78	0.00167307	0.00345762	0.63
rs5750913	794052	22	40640285	G	A	*																	
rs577525	690616	10	99769388	C	T				rs577525	C	T	0.9064	1.3521	0.5000	-0.0061	0.0077	0.4300	0.0250275	0.0154054	0.1	-0.00018562	0.00371761	0.96
rs580438	684407	3	13345450	T	C				rs580438	T	C	1.2456	1.3052	0.3400	0.0096	0.0075	0.2000	-0.0037065	0.0148923	0.8	-0.00181195	0.00362509	0.62
rs587230	775267	11	69299771	A	G				rs587230	A	G	1.9386	3.0343	0.5200	0.0059	0.0174	0.7300	0.0388155	0.0347375	0.26	6.91109E-05	0.00839424	0.99
rs6011457	690692	20	61530915	T	A				rs6011457	T	A	0.2106	1.2509	0.8700	-0.0032	0.0072	0.6500	0.0172786	0.0142754	0.23	0.00593932	0.00344268	0.084
rs6019482	685234	20	47495560	C	T				rs6019482	T	C	1.7022	2.1020	0.4200	0.0135	0.0120	0.2600	0.0128605	0.0239283	0.59	0.00592939	0.00584313	0.31
rs6058635	690979	20	30891925	G	C	*																	
rs6060151	692591	20	33594226	T	G				rs6060151	G	T	0.2157	1.2857	0.8700	-0.0002	0.0073	0.9800	-0.0034468	0.014647	0.81	-0.00369694	0.00355375	0.3
rs6061162	691901	20	29893501	T	C	*	rs35026126	0.69	rs35026126	T	C	3.1212	6.0568	0.6100	-0.0064	0.0347	0.8500	-0.0447016	0.0691794	0.52	-0.0069846	0.0165908	0.67
rs6076348	690971	20	25401827	G	A	*																	
rs6088529	688940	20	33170752	A	C	*	rs6141482	0.99	rs6141482	C	G	0.1531	1.2412	0.9000	-0.0012	0.0071	0.8600	0.0148311	0.0141609	0.29	0.00299652	0.00342995	0.38
rs6088943	686064	20	34420023	A	T	*																	
rs6121381	691119	20	30785593	T	A				rs6121381	A	T	1.2555	1.5537	0.4200	-0.0052	0.0089	0.5600	0.00240754	0.0178347	0.89	-0.00519099	0.00431989	0.23
rs6132918	665013	20	26073030	C	T				rs6132918	T	C	0.3140	1.1615	0.7900	0.0012	0.0066	0.8500	-0.014223	0.0132527	0.28	-0.00087246	0.00319527	0.78
rs6138482	795349	20	25059442	T	C	*	rs12624679	0.97	rs12624679	C	T	1.6522	1.2038	0.1700	0.0028	0.0069	0.6900	0.00909165	0.0137096	0.51	0.000561391	0.00331655	0.87
rs6142096	690983	20	32686658	A	G				rs6142096	A	G	0.4663	1.2094	0.7000	0.0026	0.0069	0.7100	-0.0004163	0.013827	0.98	-0.00491452	0.00334912	0.14
rs621042	689121	12	18789007	C	A				rs621042	A	C	2.5266	1.2421	0.0420	-0.0255	0.0071	0.0003	0.0143406	0.0141429	0.31	-0.00158895	0.00342765	0.64
rs6235	691708	5	95728898	G	C				rs6235	C	G	2.1029	1.2680	0.0970	-0.0002	0.0072	0.9800	-0.0215872	0.0144879	0.14	0.00783119	0.00350793	0.026
rs6265	795458	11	27679916	C	T	*	rs11030104	0.88	rs11030104	A	G	1.1897	1.2028	0.3200	0.0065	0.0069	0.3500	-0.0148234	0.0137241	0.28	-0.00174119	0.00331251	0.6
rs629443	790044	13	76386075	T	G	*	rs9544047	0.65	rs9544047	A	G	3.5656	1.4253	0.0120	-0.0063	0.0081	0.4400	-0.0183639	0.0162316	0.26	-0.00119922	0.00395699	0.76
rs6442021	777197	3	46742019	C	T	*																	
rs6442101	795233	3	48130893	C	T				rs6442101	T	C	0.4772	1.3470	0.7200	0.0074	0.0077	0.3400	-0.0004776	0.0153903	0.98	0.00306026	0.00374458	0.41
rs6443750	776837	3	181329682	C	T				rs6443750	T	C	1.2433	4.6638	0.7900	0.0040	0.0274	0.8800	-0.018171	0.054573	0.74	0.0147894	0.0130659	0.26
rs6448587	691097	4	28561990	A	C	*	rs68144005	0.79	rs68144005	G	A	0.5885	1.4814	0.6900	-0.0040	0.0085	0.6300	-0.0212338	0.0169195	0.21	0.00813505	0.00410251	0.047
rs6449531	691940	5	60712212	G	A				rs6449531	G	A	0.6959	1.4246	0.6300	0.0147	0.0081	0.0720	0.0007558	0.0162928	0.96	0.000846031	0.00393351	0.83
rs6459326	611474	6	58060143	C	G				rs6459326	G	C	0.1746	1.9056	0.9300	-0.0035	0.0109	0.7500	0.0162215	0.0216563	0.45	-0.00329958	0.00524491	0.53
rs6461115	791735	7	2103668	A	G	*	rs4721359	0.78	rs4721359	G	A	2.2533	1.3463	0.0940	-0.0015	0.0077	0.8500	-0.0297149	0.0153882	0.053	-0.00073996	0.00371789	0.84
rs6463489	791893	7	5542513	T	C	*	rs4724705	1.00	rs4724705	G	C	1.6294	2.1790	0.4500	0.0323	0.0125	0.0096	-0.0154297	0.0250321	0.54	-0.00443676	0.00600565	0.46
rs6474945	786471	9	15670492	G	T	*	rs7033236	0.90	rs7033236	G	A	2.1824	1.3311	0.1000	0.0051	0.0076	0.5100	0.00639989	0.0151762	0.67	-0.00307542	0.00370669	0.41
rs6477694	789230	9	111932342	C	T				rs6477694	C	T	0.5662	1.2217	0.6400	0.0080	0.0070	0.2500	-0.0132504	0.0139699	0.34	0.000329805	0.00339767	0.92
rs6490055	688822	12	111768973	G	A	*	rs4766502	0.65	rs4766502	G	T	1.2595	1.2709	0.3200	0.0016	0.0073	0.8300	-0.0059162	0.014506	0.68	-6.0065E-05	0.00354575	0.99
rs6494481	692540	15	64849904	T	G	*																	
rs6495252	685875	15	78117685	T	C	*	rs7176895	1.00	rs7176895	G	A	0.3542	1.1985	0.7700	0.0004	0.0068	0.9600	-0.0215223	0.0136462	0.11	-0.00463726	0.00331436	0.16
rs651548	680904	12	99560183	A	G				rs651548	A	G	1.7350	1.2432	0.1600	-0.0037	0.0071	0.6000	0.00809327	0.0142179	0.57	-0.00417519	0.00344558	0.23
rs6545714	793368	2	59307725	G	A	*	rs7424120	0.85	rs7424120	C	T	0.1500	1.2350	0.9000	0.0139	0.0071	0.0500	-0.0313379	0.0140942	0.026	-0.00680273	0.00341576	0.046
rs6548221	795394	2	295255	A	G				rs6548221	A	G	1.7745	1.6374	0.2800	0.0082	0.0093	0.3800	0.0014952	0.0186358	0.94	-0.00227828	0.00452212	0.61
rs6551410	668900	3	89442305	A	T				rs6551410	A	T	2.1357	1.3208	0.1100	0.0102	0.0075	0.1800	-0.0202897	0.0150605	0.18	0.00983265	0.00366659	0.0073
rs6564360	690911	16	76779612	G	A				rs6564360	G	A	0.7439	1.2937	0.5700	-0.0003	0.0074	0.9700	-0.0173635	0.0147993	0.24	0.000161078	0.00357845	0.96
rs6569648	795618	6	130349119	C	T				rs6569648	T	C	2.4552	2.4786	0.3200	0.0072	0.0142	0.6100	0.00044783	0.02831	0.99	-0.00211764	0.00691475	0.76
rs6577584	691824	1	6715390	G	T	*	rs6577585	1.00	rs6577585	A	G	0.0221	1.5596	0.9900	0.0184	0.0089	0.0390	0.0133025	0.0178189	0.46	-0.00083094	0.00427979	0.85
rs6580755	636095	12	39159190	C	T	*	rs11169508	0.98	rs11169508	C	T	1.1861	2.1367	0.5800	-0.0020	0.0122	0.8700	0.00836572	0.0243207	0.73	-0.00717274	0.00590989	0.22
rs6587552	689723	1	151018861	A	G	*	rs4970936	0.90	rs4970936	T	C	0.1776	1.7730	0.9200	-0.0040	0.0101	0.6900	0.00111494	0.0202582	0.96	0.00297404	0.00491831	0.55
rs6595205	794525	5	119372533	C	G	*	rs1811298	0.98	rs1811298	G	A	0.6458	1.4193	0.6500	0.0198	0.0081	0.0150	-0.0142021	0.016187	0.38	0.00106629	0.00389444	0.78
rs6606686	795209	12	110903380	G	C	*																	
rs663129	788948	18	57838401	A	G				rs663129	A	G	1.8521	1.5420	0.2300	0.0057	0.0088	0.5200	0.0165385	0.0175451	0.35	9.38799E-05	0.00424759	0.98
rs6681627	664345	1	174126742	A	C				rs6681627	A	C	1.9285	3.8686	0.6200	-0.0257	0.0220	0.2400	-0.0093379	0.0439319	0.83	-0.0169458	0.0109795	0.12
rs6690764	733760	1	92976590	G	A	*	rs10782922	1.00	rs10782922	G	A	1.2655	3.3541	0.7100	0.0128	0.0191	0.5000	0.0195531	0.0380061	0.61	-0.00012263	0.00909031	0.99
rs6700816	686392	1	174883994	A	T	*																	
rs6700838	690255	1	47700027	C	T	*																	
rs6710871	741157	2	143960593	A	G				rs6710871	G	A	0.4233	1.8657	0.8200	0.0127	0.0106	0.2300	0.0163979	0.0212329	0.44	0.00499927	0.00512989	0.33
rs6711584	680384	2	104421692	A	G	*																	
rs6713781	674261	2	40291940	G	C	*																	
rs6720868	691649	2	230663576	T	C				rs6720868	T	C	2.1360	1.3932	0.1300	0.0000	0.0080	1.0000	0.00157208	0.0159581	0.92	0.00414255	0.00382878	0.28
rs6738445	787966	2	172599615	C	T				rs6738445	T	C	0.1784	1.5187	0.9100	0.0045	0.0087	0.6000	-0.004748	0.0173173	0.78	-0.00186485	0.00417223	0.65
rs6753170	683741	2	58481863	C	T				rs6753170	C	T	2.0863	1.2183	0.0870	0.0050	0.0070	0.4800	0.00947428	0.0139204	0.5	0.00140935	0.00336459	0.68
rs6764533	690832	3	196088464	A	G	*																	
rs676749	691753	20	3026069	T	A				rs676749	T	A	2.2582	1.6920	0.1800	-0.0004	0.0097	0.9600	0.0157699	0.0193439	0.41	0.00149638	0.00470202	0.75
rs6767619	691865	3	156893782	C	G				rs6767619	G	C	0.5892	1.1857	0.6200	-0.0093	0.0068	0.1700	0.00395148	0.0135797	0.77	0.00287262	0.00328621	0.38
rs6786125	689715	3	28727523	G	C	*	rs4380374	0.64	rs4380374	G	T	0.7431	1.2136	0.5400	-0.0069	0.0069	0.3200	0.0211314	0.0138383	0.13	0.000335764	0.00335228	0.92
rs6786582	791202	3	135898984	C	T	*	rs6439629	0.88	rs6439629	A	G	1.9486	2.0792	0.3500	0.0017	0.0118	0.8800	-0.0549612	0.023716	0.02	-0.00570836	0.00569075	0.32
rs6803870	701791	3	89443784	T	C	*	rs4425279	0.65	rs4425279	A	G	0.4252	1.4578	0.7700	-0.0031	0.0084	0.7200	0.00738778	0.0167149	0.66	-0.00641334	0.00406049	0.11
rs6804181	687538	3	116937546	A	T				rs6804181	A	T	0.0735	1.5136	0.9600	0.0068	0.0086	0.4300	0.0170473	0.0172555	0.32	0.00478252	0.0041679	0.25
rs6804842	789179	3	25106437	G	A	*																	
rs6818414	685947	4	16600664	C	T				rs6818414	C	T	0.0926	1.2903	0.9400	-0.0039	0.0074	0.6000	-0.010722	0.0147408	0.47	0.00222434	0.00356526	0.53
rs6827083	794577	4	153075491	G	A	*																	
rs6843738	792315	4	103936001	G	A				rs6843738	A	G	1.3618	1.2336	0.2700	0.0017	0.0070	0.8100	-0.0112374	0.0140429	0.42	-0.0048598	0.00340418	0.15
rs6877851	685857	5	130356413	G	C	*	rs4705887	0.62	rs4705887	T	G	0.4180	1.3754	0.7600	-0.0054	0.0079	0.4900	-0.0058361	0.015751	0.71	0.000653588	0.00380102	0.86
rs6879326	690684	5	59208302	C	T				rs6879326	T	C	0.9177	1.3336	0.4900	0.0058	0.0076	0.4500	0.0173427	0.0152478	0.26	-0.00249815	0.00368117	0.5

rs6888159	691415	5	63932234	C	G				rs6888159	C	G	2.0338	1.2514	0.1000	-0.0124	0.0072	0.0830	0.00905255	0.0143099	0.53	-0.00065775	0.00347757	0.85
rs6900723	690104	6	23876240	C	T	*	rs73740848	0.85	rs73740848	C	A	1.8291	2.0444	0.3700	-0.0046	0.0117	0.6900	-0.0093154	0.0233993	0.69	-0.013437	0.00565226	0.017
rs6904676	677904	6	58705746	A	C				rs6904676	A	C	0.8589	1.3840	0.5300	0.0101	0.0079	0.2000	-0.0013385	0.0157921	0.93	0.0055296	0.00383221	0.15
rs6908295	593900	6	27527205	C	A	*	rs9468156	0.95	rs9468156	C	T	0.8269	1.2072	0.4900	0.0025	0.0069	0.7100	0.00247121	0.0137711	0.86	0.000431855	0.0033352	0.9
rs6919443	691422	6	104493098	G	A				rs6919443	G	A	2.4087	1.2074	0.0460	0.0142	0.0069	0.0400	0.0255957	0.013793	0.063	0.00213819	0.00335548	0.52
rs6921533	690769	6	73742334	T	C	*																	
rs6922214	683298	6	69968484	G	A				rs6922214	G	A	1.5935	6.4572	0.8100	-0.0646	0.0381	0.0900	-0.0333291	0.0759453	0.66	0.0117913	0.0186009	0.53
rs6922855	685455	6	51843542	A	G				rs6922855	A	G	1.4867	1.2282	0.2300	-0.0067	0.0070	0.3400	-0.0047507	0.0139811	0.73	0.00239623	0.00339644	0.48
rs6938239	691202	6	34683635	G	A				rs6938239	G	A	1.4653	4.5775	0.7500	0.0169	0.0268	0.5300	-0.0179393	0.053403	0.74	0.0302788	0.0129617	0.019
rs6963840	637209	7	78144371	T	C				rs6963840	C	T	1.4940	2.2991	0.5200	0.0152	0.0131	0.2500	0.00504842	0.0263376	0.85	-0.0068125	0.00636739	0.28
rs696606	660690	1	16828640	A	G	*																	
rs6968554	794218	7	17287106	G	A	*	rs2106727	0.94	rs2106727	A	G	0.4491	1.2318	0.7200	-0.0026	0.0070	0.7100	-0.0155879	0.0140598	0.27	0.00452445	0.00340877	0.18
rs7006629	691635	8	87519542	T	C				rs7006629	C	T	0.4653	1.2466	0.7100	-0.0204	0.0071	0.0042	0.0154556	0.0142564	0.28	-0.00063854	0.00346363	0.85
rs7024334	782431	9	109072075	T	G	*																	
rs7042372	681234	9	6959840	A	G	*	rs10815491	0.77	rs10815491	T	G	0.1677	1.2393	0.8900	0.0067	0.0071	0.3400	-0.0091417	0.01414	0.52	-0.00197468	0.00343356	0.57
rs7083450	795609	10	103984060	T	C				rs7083450	C	T	1.4626	1.7756	0.4100	-0.0013	0.0102	0.9000	0.00163519	0.0204338	0.94	-0.00180076	0.00498308	0.72
rs7084454	678564	10	21821274	A	G	*	rs1416901	0.86	rs1416901	A	G	1.9158	2.3070	0.4100	-0.0057	0.0132	0.6700	-0.0028661	0.0263085	0.91	-0.00024722	0.00630472	0.97
rs7102454	691134	11	65594820	C	T	*	rs11227314	0.99	rs11227314	A	G	0.4668	1.3327	0.7300	-0.0105	0.0076	0.1700	-0.0149655	0.0151739	0.32	0.00387176	0.00368098	0.29
rs710355	676206	5	87159003	G	C				rs710355	C	G	2.1774	7.1525	0.7600	-0.0529	0.0405	0.1900	0.0165116	0.0807234	0.84	0.0238443	0.0191974	0.21
rs711347	688432	4	52926216	T	A	*	rs398015	0.83	rs398015	C	T	0.4496	1.2277	0.7100	-0.0027	0.0070	0.7000	0.00199341	0.0139935	0.89	0.00186831	0.00338143	0.58
rs7117238	788879	11	78040259	G	A	*	rs7940841	0.98	rs7940841	A	G	1.1228	1.2473	0.3700	-0.0023	0.0071	0.7500	-0.0185148	0.014254	0.19	-0.00134716	0.00346804	0.7
rs7120873	691850	11	49459474	T	C				rs7120873	T	C	2.0495	1.5763	0.1900	-0.0135	0.0090	0.1300	0.0171604	0.0179469	0.34	0.00143051	0.0043451	0.74
rs7122539	795230	11	66662731	G	A				rs7122539	G	A	2.4348	1.1846	0.0400	0.0018	0.0068	0.7900	-0.0019713	0.013544	0.88	0.00457918	0.00327228	0.16
rs7123876	793551	11	72444583	C	T				rs7123876	T	C	0.1322	1.6150	0.9300	0.0126	0.0092	0.1700	0.0668789	0.0184495	0.00029	-0.00121583	0.00446313	0.79
rs7124442	793435	11	27677041	C	T	*																	
rs7124681	795474	11	47529947	A	C				rs7124681	A	C	0.8301	1.3674	0.5400	0.0053	0.0078	0.4900	0.0124345	0.0155637	0.42	-0.00548326	0.00375053	0.14
rs7131262	787486	11	47836302	T	A				rs7131262	T	A	1.3747	1.2105	0.2600	0.0036	0.0069	0.6000	0.00940749	0.0138074	0.5	-0.00234432	0.00337021	0.49
rs7133378	784423	12	124409502	A	G				rs7133378	A	G	1.8164	1.8039	0.3100	0.0022	0.0103	0.8300	0.00115419	0.0205837	0.96	-0.00335232	0.00497003	0.5
rs7134375	795589	12	20473758	A	C				rs7134375	C	A	0.9490	1.3979	0.5000	0.0109	0.0080	0.1700	0.002526	0.0159615	0.87	0.000178297	0.00391456	0.96
rs7138803	795588	12	50247468	A	G	*	rs112502508	0.90	rs112502508	G	A	1.7800	1.3154	0.1800	-0.0008	0.0075	0.9100	0.0123572	0.0150085	0.41	3.91664E-05	0.0036431	0.99
rs7144011	794117	14	79940383	T	G	*																	
rs7147503	691094	14	101539384	C	T				rs7147503	C	T	0.2308	1.2228	0.8500	-0.0045	0.0070	0.5200	0.0133697	0.0139612	0.34	-0.00377556	0.00338749	0.27
rs7164727	791156	15	73093991	T	C	*																	
rs7181498	690980	15	95271404	T	C	*	rs7179870	1.00	rs7179870	G	C	1.3547	1.3362	0.3100	-0.0039	0.0076	0.6100	0.0140622	0.0152698	0.36	0.00136583	0.00368432	0.71
rs7181610	667503	15	35826859	A	T	*	rs6495779	0.70	rs6495779	C	T	1.9052	6.9345	0.7800	0.0515	0.0393	0.1900	0.0357147	0.0783387	0.65	0.0230567	0.0192404	0.23
rs7187776	792563	16	28857645	G	A	*	rs7185232	0.75	rs7185232	T	C	2.1688	1.3910	0.1200	-0.0032	0.0080	0.6900	-0.002794	0.0159184	0.86	-0.00078023	0.00386128	0.84
rs7195386	687736	16	24578458	T	C	*	rs7204292	0.65	rs7204292	T	C	1.9976	1.1819	0.0910	-0.0036	0.0068	0.5900	-0.0016624	0.0135099	0.9	0.000817017	0.00327993	0.8
rs719802	692544	11	113234679	T	C	*																	
rs7200919	793695	16	67316600	A	G	*																	
rs7206608	689058	16	82872628	G	C	*																	
rs7209235	683445	17	73759552	G	A				rs7209235	G	A	1.9883	1.6051	0.2200	-0.0018	0.0092	0.8500	0.0179803	0.0183596	0.33	0.000696787	0.00444342	0.88
rs7211567	686595	17	31460899	C	T				rs7211567	T	C	0.8273	1.2854	0.5200	-0.0050	0.0073	0.5000	-0.0027217	0.0146792	0.85	-0.00027443	0.00355293	0.94
rs7217226	788202	17	2136065	G	T				rs7217226	T	G	0.2553	1.3102	0.8500	0.0006	0.0075	0.9400	-0.0039721	0.0149649	0.79	-0.00390759	0.00363431	0.28
rs7239114	769496	18	45921214	A	G	*																	
rs7243357	791550	18	56883319	T	G				rs7243357	G	T	1.7684	1.5175	0.2400	-0.0045	0.0087	0.6000	0.0214168	0.0172835	0.22	0.00017457	0.00418961	0.97
rs7313924	785448	12	89899912	C	G	*	rs10858892	1.00	rs10858892	T	A	1.3072	1.3295	0.3300	0.0085	0.0076	0.2600	-0.0070768	0.0151453	0.64	0.00726665	0.00369229	0.049
rs7334078	688374	13	99120484	T	C				rs7334078	C	T	0.0612	1.5762	0.9700	0.0035	0.0090	0.7000	0.0214963	0.018008	0.23	-0.00337719	0.00436753	0.44
rs733594	791828	8	85077686	T	C				rs733594	T	C	2.6840	1.1929	0.0240	0.0127	0.0068	0.0630	-0.0193374	0.0136033	0.16	-0.00165183	0.00329366	0.62
rs7377083	621633	4	102708997	A	C				rs7377083	C	A	1.5459	1.2269	0.2100	-0.0072	0.0070	0.3000	0.00824405	0.0139999	0.56	-0.00060082	0.00339983	0.86
rs740157	787152	7	77055885	A	G	*																	
rs7425440	793204	2	512490	T	C				rs7425440	C	T	2.7723	1.5385	0.0720	-0.0115	0.0088	0.1900	0.0125743	0.0175597	0.47	0.00187532	0.00423645	0.66
rs7444298	691820	5	87730027	A	G	*																	
rs7478904	691367	11	48630323	C	T	*	rs11039798	0.98	rs11039798	G	A	0.0511	1.6010	0.9700	-0.0094	0.0091	0.3000	-0.0081787	0.0182498	0.65	-0.00426468	0.00442877	0.34
rs753010	663295	20	29857455	G	C	*																	
rs7531118	781714	1	72837239	C	T				rs7531118	C	T	1.9401	1.3709	0.1600	-0.0071	0.0078	0.3700	0.00401051	0.0156013	0.8	-0.00046233	0.0038034	0.9
rs7531656	794253	1	49828663	A	G	*																	
rs7535528	632868	1	2444414	G	A				rs7535528	G	A	1.4096	1.2709	0.2700	0.0128	0.0073	0.0790	-0.0286407	0.0145049	0.048	0.00159595	0.00351979	0.65
rs7550711	769184	1	110082886	T	C	*																	
rs7556169	692527	1	8741401	G	A	*																	
rs7557796	692414	2	86766153	T	C	*																	
rs7560871	691571	2	145616899	A	G	*	rs115590225	0.70	rs115590225	A	G	1.9426	5.5723	0.7300	0.0006	0.0319	0.9800	-0.0096807	0.0641331	0.88	-0.0128689	0.0154949	0.41
rs7561278	677253	2	48954905	T	C				rs7561278	C	T	0.6053	1.2849	0.6400	-0.0075	0.0073	0.3100	0.00457704	0.0146541	0.75	-0.00169348	0.00355725	0.63
rs7564679	792149	2	204012790	G	A	*	rs6704959	0.85	rs6704959	A	G	0.5940	1.2612	0.6400	-0.0029	0.0072	0.6900	-0.0001432	0.014351	0.99	-0.00277553	0.00349176	0.43
rs756717	771976	16	72996162	G	A	*	rs756718	0.79	rs756718	T	C	1.2649	1.2814	0.3200	0.0065	0.0073	0.3700	0.00010872	0.0146535	0.99	-0.00327153	0.00354759	0.36
rs757318	792816	19	18820308	C	A	*																	
rs757608	794314	17	59497277	G	A	*	rs9905385	0.99	rs9905385	A	G	0.7531	1.3098	0.5700	0.0000	0.0075	1.0000	0.0177709	0.0149039	0.23	-0.00694258	0.00360817	0.054
rs7598402	673277	2	50735943	C	G	*	rs13001104	0.77	rs13001104	A	C	1.1387	1.1980	0.3400	-0.0068	0.0068	0.3200	0.00155324	0.0136838	0.91	-0.0048338	0.0033059	0.14
rs7599312	780823	2	213413231	G	A				rs7599312	A	G	2.0657	3.5936	0.5700	0.0123	0.0204	0.5500	0.0248438	0.0407636	0.54	0.0199932	0.0101977	0.05
rs7600699	691202	2	156018263	G	C				rs7600699	G	C	0.9326	1.3050	0.4700	-0.0021	0.0074	0.7700	0.022723	0.0148895	0.13	0.00375969	0.00361139	0.3
rs7601895	679924	2	55281901	C	G	*																	
rs7607351	685674	2	69562127	T	C				rs7607351	C	T	1.7451	1.3863	0.2100	0.0078	0.0079	0.3200	-0.0060168	0.0158159	0.7	0.00535665	0.0038087	0.16
rs7607369	790384	2	219279097	A	G				rs7607369	G	A	0.7247	1.3586	0.5900	0.0051	0.0078	0.5100	-0.0082916	0.0154916	0.59	-0.00167429	0.00375782	0.66
rs7607490	690798	2	12851120	A	G	*																	
rs760880	795087	6	27408181	G	T	*	rs9368504	0.80	rs9368504	G	T	2.2373	1.1888	0.0600	-0.0056	0.0068	0.4100	0.0218835	0.0135312	0.11	0.0028576	0.0032728	0.38
rs761423	692545	1	17301672	T	C	*																	
rs7615297	689710	3	156299313	C	G				rs7615297	C	G	4.1268	2.9195	0.1600	-0.0002	0.0167	0.9900	-0.0245475	0.0333619	0.46	0.00670786	0.00826523	0.42
rs7616371	775912	3	137047387	G	A	*																	
rs762147	692496	21	39238610	G	A				rs762147	A	G	2.3682	2.1162	0.2600	-0.0026	0.0121	0.8300	-0.0280216	0.024121	0.25	0.00348043	0.00581214	0.55
rs7640424	790612	3	107820063	C	T	*																	
rs765332	753766	6	51874745	G	T	*																	
rs7674623	685776	4	80794681	T	C				rs7674623	T	C	1.5552	2.5946	0.5500	-0.0125	0.0148	0.4000	-0.0047746	0.0295298	0.87	0.0105035	0.00726432	0.15
rs7683836	686968	4	180167906	G	A				rs7683836	A	G	0.2570	1.2138	0.8300	0.0115	0.0069	0.0960	-0.0324372	0.0138323	0.019	-0.00161097	0.00334152	0.63
rs7692088	688149	4	10491040	C	G				rs7692088	G	C	0.4737	1.2119	0.7000	0.0095	0.0069	0.1700	0.00868479	0.0138177	0.53	-0.00302694	0.00334264	0.37
rs7694732	690622	4	115124089	A	G	*	rs34331737	0.99	rs34331737	A	G	3.2051	1.2549	0.0110	-0.0032	0.0072	0.6500	0.0265033	0.0143512	0.065	-0.00578498	0.00348455	0.097
rs769674	685942	4	140881964	A	T				rs769674	A	T	1.4652	1.3321	0.2700	-0.0017	0.0076	0.8200	-0.0180725	0.0152188	0.24	7.40287E-05	0.00369893	0.98
rs7702514	684516	5	86727027	C	T	*																	
rs7710595	689024	5	106506697	A	C	*	rs10515368	0.98	rs10515368	T	C	0.2275	1.2755	0.8600	0.0039	0.0073	0.5900	0.00743619	0.0145501	0.61	-0.00186348	0.0035283	0.6

rs7711753	794802	5	122733317	G	A				rs7711753	G	A	1.2010	1.2037	0.3200	-0.0055	0.0069	0.4300	-0.0087081	0.0137058	0.53	0.0035295	0.00331648	0.29
rs7715256	795302	5	153537893	G	T	*																	
rs7716275	781833	5	137631073	G	T	*																	
rs7727781	683127	5	165185571	T	C				rs7727781	C	T	2.7220	1.2022	0.0240	-0.0063	0.0069	0.3600	-0.005493	0.0137468	0.69	-7.7371E-05	0.00332915	0.98
rs7730004	690164	5	43191033	T	C	*	rs7723426	0.94	rs7723426	T	C	1.5296	1.6193	0.3400	-0.0014	0.0092	0.8800	-0.0045206	0.0184158	0.81	0.00327464	0.00449276	0.47
rs7730898	792975	5	170459675	A	G				rs7730898	A	G	0.9160	1.8396	0.6200	-0.0075	0.0105	0.4800	-0.0345313	0.020918	0.099	0.00209005	0.00511415	0.68
rs7748777	790069	6	41133806	A	G				rs7748777	A	G	0.8887	1.2196	0.4700	-0.0059	0.0070	0.4000	0.00505867	0.0139242	0.72	-0.00263795	0.00337791	0.43
rs775731	684541	3	77624784	C	T				rs775731	T	C	0.2365	1.2932	0.8500	0.0012	0.0074	0.8700	-0.0013334	0.014724	0.93	0.00278628	0.00359697	0.44
rs7760082	690649	6	21919387	G	A	*																	
rs7777102	778320	7	73058017	G	A	*	rs35814008	0.95	rs35814008	A	C	0.2268	1.6093	0.8900	-0.0003	0.0092	0.9700	-0.0022351	0.01835	0.9	0.00158578	0.00445465	0.72
rs7779498	671584	7	130408415	C	T	*																	
rs7784465	685477	7	6418275	C	T				rs7784465	C	T	1.1767	4.6377	0.8000	0.0155	0.0266	0.5600	-0.096809	0.0534668	0.07	0.0218544	0.01288	0.09
rs7796608	601887	7	897847	A	G				rs7796608	G	A	1.4350	1.6501	0.3800	-0.0091	0.0095	0.3400	0.0119921	0.0188864	0.53	-0.00800338	0.00455805	0.079
rs7805441	682488	7	78121458	T	C	*	rs35114939	0.96	rs35114939	G	A	0.2132	1.1894	0.8600	-0.0070	0.0068	0.3000	-0.0059875	0.0135541	0.66	0.000203235	0.00329402	0.95
rs7827182	582727	8	8380471	C	G	*	rs13270194	0.97	rs13270194	T	C	0.1980	1.6336	0.9000	0.0092	0.0093	0.3200	-0.0268879	0.018575	0.15	0.000370306	0.00447396	0.93
rs7832003	682766	8	10782004	G	C	*																	
rs7844647	793703	8	34503776	T	C				rs7844647	T	C	0.2188	1.2212	0.8600	-0.0020	0.0070	0.7800	-0.0344164	0.0139496	0.014	0.000302121	0.0033784	0.93
rs785278	690853	1	33307987	T	A	*	rs811483	1.00	rs811483	C	T	2.6877	1.4582	0.0650	-0.0001	0.0083	0.9900	0.012336	0.0166427	0.46	-0.00380027	0.00405881	0.35
rs7865157	690934	9	122631560	T	C				rs7865157	T	C	2.5369	6.7246	0.7100	-0.0312	0.0387	0.4200	-0.0099943	0.0770956	0.9	-0.0188701	0.0192391	0.33
rs7869771	679436	9	94180627	A	C				rs7869771	C	A	2.1980	1.3437	0.1000	-0.0043	0.0077	0.5700	0.0107647	0.0153039	0.48	0.00751024	0.00372606	0.044
rs7871866	683494	9	131027982	C	G				rs7871866	G	C	6.0397	1.5427	0.0001	0.0042	0.0088	0.6300	0.00983163	0.01765	0.58	-0.00378042	0.00425516	0.37
rs7874154	685829	9	27777012	C	T	*	rs10757685	0.62	rs10757685	T	C	2.0540	1.9812	0.3000	0.0152	0.0114	0.1800	-0.0307177	0.0227245	0.18	0.00601113	0.00553961	0.28
rs7893571	687864	10	16750129	T	G	*	rs2356377	0.95	rs2356377	C	T	1.8164	1.7138	0.2900	0.0036	0.0098	0.7100	-0.0166279	0.0195066	0.39	-0.00335629	0.00472393	0.48
rs7899106	793689	10	87410904	G	A				rs7899106	G	A	2.9995	6.0627	0.6200	0.0736	0.0343	0.0320	0.00475403	0.0684238	0.94	0.0156147	0.0172805	0.37
rs7903146	795624	10	114758349	C	T	*																	
rs7919	685991	16	70514828	C	A	*	rs9939726	0.79	rs9939726	G	C	0.3989	1.2254	0.7400	-0.0036	0.0070	0.6100	-0.0004722	0.013981	0.97	-0.00132575	0.00339578	0.7
rs7924371	689201	11	49620595	C	T				rs7924371	C	T	1.8308	1.1992	0.1300	-0.0050	0.0069	0.4700	-0.0002287	0.0136932	0.99	0.0031099	0.00331571	0.35
rs793520	687049	10	99032375	A	G				rs793520	G	A	2.0239	1.7016	0.2300	0.0183	0.0097	0.0580	-0.0040897	0.019341	0.83	0.00495633	0.00472553	0.29
rs7941030	795457	11	122522375	C	T				rs7941030	C	T	0.0076	1.3195	1.0000	-0.0028	0.0075	0.7100	-0.0014298	0.0150593	0.92	0.00515324	0.00366304	0.16
rs7948120	756039	11	28763321	C	T	*	rs1607227	0.70	rs1607227	T	G	1.1614	1.2567	0.3600	-0.0067	0.0072	0.3500	-0.0067008	0.0143143	0.64	0.00214192	0.00346956	0.54
rs7958206	682999	12	39329294	A	G				rs7958206	G	A	1.3231	1.1728	0.2600	0.0033	0.0067	0.6200	-0.015596	0.0133736	0.24	-0.00185296	0.00326261	0.57
rs7965658	691448	12	49987929	G	A				rs7965658	G	A	0.4928	1.9239	0.8000	-0.0008	0.0110	0.9400	0.0224076	0.0218524	0.31	-0.00300448	0.00531738	0.57
rs7968230	690439	12	133481917	A	G	*																	
rs7973955	684108	12	118409640	G	A	*	rs7963783	0.99	rs7963783	T	G	2.7258	1.3022	0.0360	0.0034	0.0074	0.6500	0.00187443	0.0148284	0.9	0.00241028	0.00360093	0.5
rs7975187	691440	12	60964108	G	A				rs7975187	A	G	0.5128	1.3253	0.7000	-0.0082	0.0076	0.2800	-9.203E-05	0.015171	1	-0.00262341	0.00366705	0.47
rs799132	685905	14	82684748	T	A				rs799132	T	A	1.9603	1.7306	0.2600	-0.0139	0.0099	0.1600	-0.0034521	0.0196743	0.86	0.00562049	0.00479126	0.24
rs799449	684521	7	44784697	T	C				rs799449	C	T	0.5043	1.4375	0.7300	-0.0002	0.0082	0.9800	0.00885173	0.016448	0.59	-0.00044518	0.00400502	0.91
rs8016771	691604	14	102649451	G	T	*	rs12887554	0.77	rs12887554	C	T	1.1375	2.5894	0.6600	0.0002	0.0148	0.9900	0.00055075	0.0295063	0.99	0.00873368	0.00732521	0.23
rs8016859	781430	14	30484722	C	G	*																	
rs8024932	685606	15	77915282	T	G				rs8024932	G	T	2.2542	1.9146	0.2400	0.0092	0.0110	0.4100	0.00611576	0.0219579	0.78	-0.0051458	0.00529919	0.33
rs8033510	686532	15	61445514	T	C	*	rs4238351	0.95	rs4238351	G	A	1.2765	1.2696	0.3100	0.0110	0.0073	0.1300	-0.0024971	0.014546	0.86	-0.00215128	0.00353244	0.54
rs8036040	691068	15	36402716	A	C	*																	
rs8046061	680891	16	80752293	T	C				rs8046061	T	C	1.0130	1.2853	0.4300	-0.0026	0.0073	0.7200	-0.0211324	0.0146407	0.15	0.00208879	0.00355286	0.56
rs8047395	788856	16	53798523	A	G				rs8047395	A	G	2.1133	1.2362	0.0870	-0.0019	0.0070	0.7800	0.0116357	0.0140673	0.41	9.96999E-05	0.00340161	0.98
rs8067737	649518	17	29349688	T	C				rs8067737	T	C	0.4971	1.2423	0.6900	0.0187	0.0071	0.0085	-0.0188267	0.0141825	0.18	0.000192601	0.00340985	0.95
rs8069296	794875	17	42935059	C	T				rs8069296	C	T	2.0683	1.2291	0.0920	0.0181	0.0070	0.0098	-0.0135893	0.0140243	0.33	-0.00189257	0.00340024	0.58
rs8070454	795617	17	38160754	C	T				rs8070454	C	T	0.3491	1.2002	0.7700	0.0087	0.0068	0.2000	-0.0244395	0.0136755	0.074	-0.00120368	0.00331707	0.72
rs8071182	771437	17	55336155	A	G				rs8071182	A	G	2.0139	1.2929	0.1200	-0.0102	0.0074	0.1600	0.0156384	0.0147254	0.29	-0.00288562	0.00360597	0.42
rs8075273	795026	17	61728881	C	A				rs8075273	A	C	1.9345	2.5556	0.4500	0.0080	0.0145	0.5800	0.0001994	0.0289384	0.99	-0.00342366	0.00696058	0.62
rs8079034	687609	17	5412361	T	C	*	rs9889954	0.81	rs9889954	G	A	0.3510	1.7131	0.8400	0.0006	0.0098	0.9500	0.0157305	0.0195053	0.42	-0.00260246	0.00472465	0.58
rs8081039	677239	17	75995829	T	C				rs8081039	T	C	1.5047	4.6028	0.7400	0.0228	0.0261	0.3800	0.00681464	0.051964	0.9	0.00547674	0.0127653	0.67
rs8087550	681066	18	58371566	C	A				rs8087550	C	A	0.3384	1.2201	0.7800	-0.0161	0.0070	0.0210	0.0249983	0.0139139	0.072	0.000696278	0.00338014	0.84
rs8089514	668999	18	69224478	A	T				rs8089514	T	A	2.8660	1.4954	0.0550	-0.0020	0.0085	0.8100	-0.0146721	0.0170528	0.39	0.000452518	0.00413727	0.91
rs8092503	777619	18	52479487	G	A				rs8092503	G	A	2.4988	1.2243	0.0410	-0.0003	0.0070	0.9700	-0.021487	0.013955	0.12	-0.00116743	0.00338684	0.73
rs8094523	779758	18	57878155	G	A				rs8094523	A	G	3.5681	5.6276	0.5300	-0.0370	0.0319	0.2500	-0.0279333	0.0635203	0.66	-0.0191419	0.015852	0.23
rs8095404	781145	18	57804346	T	A	*																	
rs8097544	686083	18	1839564	G	A				rs8097544	G	A	2.7745	6.0682	0.6500	-0.0272	0.0343	0.4300	-0.0904803	0.068452	0.19	-0.00693991	0.0166189	0.68
rs8102137	692575	19	30296853	C	T				rs8102137	C	T	1.9074	1.8821	0.3100	-0.0063	0.0107	0.5600	-0.0033249	0.0214765	0.88	0.00359887	0.00522117	0.49
rs8121840	684197	20	30504530	A	G				rs8121840	G	A	0.4802	1.2190	0.6900	0.0035	0.0070	0.6200	0.0080133	0.0139673	0.57	0.00291834	0.00336576	0.39
rs8123881	793018	20	15819495	G	A				rs8123881	A	G	0.6746	1.7830	0.7100	-0.0094	0.0102	0.3600	-0.0361374	0.0203246	0.075	0.00216268	0.00491205	0.66
rs816364	692516	6	53990465	G	A				rs816364	A	G	0.3025	1.3397	0.8200	0.0116	0.0076	0.1300	0.0356871	0.0152812	0.02	0.00111723	0.00371103	0.76
rs816533	671852	20	26256565	G	A	*																	
rs820077	692106	6	35033854	G	A				rs820077	G	A	1.8628	1.8265	0.3100	-0.0092	0.0104	0.3800	-0.0069552	0.0208311	0.74	-0.00827374	0.0050274	0.1
rs823074	689143	1	205774839	T	C	*	rs823075	1.00	rs823075	T	C	2.2945	4.7604	0.6300	-0.0027	0.0270	0.9200	0.00235722	0.0537624	0.97	-0.0156235	0.0128642	0.22
rs825680	680731	16	73606563	A	T	*																	
rs827092	692482	3	157985182	T	C	*																	
rs845084	685413	10	125220036	A	G				rs845084	A	G	0.1510	1.2228	0.9000	0.0000	0.0070	0.9900	0.0112468	0.0139469	0.42	0.00425824	0.00337494	0.21
rs847747	685794	6	40080069	G	T	*	rs847750	1.00	rs847750	A	G	0.3298	1.2047	0.7800	-0.0035	0.0069	0.6100	0.011677	0.0137328	0.4	0.00475521	0.0033306	0.15
rs849135	793909	7	28196413	A	G	*	rs860262	0.77	rs860262	A	C	0.5814	1.4199	0.6800	-0.0122	0.0081	0.1300	0.0309597	0.0161549	0.055	0.00371093	0.00394683	0.35
rs853679	792572	6	28296863	A	C				rs853679	A	C	1.3162	1.8449	0.4800	0.0065	0.0105	0.5400	0.0283504	0.0211133	0.18	-0.00173687	0.00509396	0.73
rs8567	784841	20	62522315	G	A				rs8567	A	G	1.9917	1.5606	0.2000	0.0048	0.0089	0.5900	-0.0125583	0.0178172	0.48	-0.00050903	0.00429963	0.91
rs868554	681858	16	20050466	G	C				rs868554	C	G	1.2898	1.2629	0.3100	0.0221	0.0072	0.0022	0.00839217	0.0144247	0.56	0.00494748	0.00349767	0.16
rs874454	686146	7	131896813	G	A				rs874454	A	G	0.1212	1.5216	0.9400	0.0027	0.0087	0.7600	-0.0144969	0.0173691	0.4	0.000448057	0.00419255	0.91
rs879620	688377	16	4015729	T	C				rs879620	C	T	2.5470	1.3069	0.0510	-0.0024	0.0075	0.7500	-0.0050852	0.0149742	0.73	-0.00081703	0.00364357	0.82
rs881301	691753	8	38332318	C	T	*	rs881299	0.99	rs881299	T	C	1.3589	1.2341	0.2700	-0.0053	0.0070	0.4500	-0.009207	0.0140657	0.51	0.0114342	0.00340168	0.00078
rs884282	691002	12	117579274	C	T				rs884282	C	T	1.6516	1.2069	0.1700	0.0064	0.0069	0.3500	-0.0041956	0.0137195	0.76	-0.00595289	0.00333884	0.075
rs886444	793863	17	46051911	G	A	*																	
rs889398	789694	16	69556715	C	T				rs889398	T	C	1.9407	1.7558	0.2700	-0.0100	0.0101	0.3200	0.026651	0.0201329	0.19	-0.00419717	0.00482312	0.38
rs892261	691303	13	65884191	C	T				rs892261	C	T	1.9876	1.3881	0.1500	-0.0073	0.0079	0.3500	0.00653421	0.0158146	0.68	-0.00555338	0.00383	0.15
rs895330	684271	19	4060707	C	G	*	rs7254272	0.63	rs7254272	A	G	2.6032	3.4617	0.4500	0.0095	0.0196	0.6300	-0.0116827	0.0391156	0.77	-0.0234461	0.00932971	0.012
rs900144	692126	11	13294268	T	C				rs900144	C	T	0.5472	1.2275	0.6600	0.0127	0.0070	0.0710	-0.0203339	0.0140389	0.15	0.00295541	0.00342295	0.39
rs901630	794597	6	98539519	C	T	*	rs13194250	0.81	rs13194250	T	C	0.3518	1.2255	0.7700	0.0043	0.0070	0.5400	-0.0149489	0.0140172	0.29	-0.00053626	0.00341377	0.88
rs902695	678635	2	113955074	G	A	*	rs2241978	1.00	rs2241978	A	G	0.5934	1.2239	0.6300	0.0059	0.0070	0.4000	0.0205951	0.0139908	0.14	0.000559868	0.0033982	0.87
rs903959	689895	8	142630782	A	T				rs903959	A	T	0.4666	1.5877	0.7700	0.0031	0.0090	0.7300	-0.0115541	0.0180515	0.52	0.00612396	0.00442336	0.17
rs905938	795227	1	154991389	C	T				rs905938	C	T	2.3916	2.3734	0.3100	-0.0196	0.0135	0.1500	-0.0458208	0.0269299	0.089	0.0088516	0.00644752	0.17
rs907011	662024	16	54234492	T	G	*																	
rs9077	663139	1	202116238	G	A	*																	

rs912768	690979	1	173473713	C	G	*																	
rs919433	685331	2	198166565	A	G				rs919433	A	G	0.0207	1.2049	0.9900	0.0036	0.0069	0.6000	0.00539105	0.0137211	0.69	0.000206932	0.00332184	0.95
rs925018	792062	3	62713143	G	C	*																	
rs925421	674423	4	60253877	A	G	*																	
rs926279	725673	6	58298846	G	A				rs926279	G	A	0.0843	1.2252	0.9500	-0.0055	0.0070	0.4300	-0.003759	0.013986	0.79	-0.00056214	0.00340978	0.87
rs9289499	679548	3	135484734	C	T				rs9289499	C	T	3.4655	4.5396	0.4500	0.0144	0.0261	0.5800	-0.0407791	0.0519884	0.43	-0.0152853	0.0127738	0.23
rs9291467	688636	4	25428296	T	C				rs9291467	C	T	0.2130	1.5951	0.8900	-0.0075	0.0091	0.4100	-0.0127132	0.0181414	0.48	0.00154728	0.00435711	0.72
rs9294260	783533	6	83433228	A	G				rs9294260	A	G	1.1502	1.3939	0.4100	-0.0016	0.0079	0.8400	-0.0155462	0.0158588	0.33	0.00108822	0.00387365	0.78
rs929641	793752	2	58792377	A	G				rs929641	A	G	0.0852	1.1920	0.9400	0.0021	0.0068	0.7600	0.0181537	0.0136459	0.18	-0.00195489	0.00331938	0.56
rs9299	692577	17	46669430	T	C	*	rs11079830	0.70	rs11079830	A	G	1.0821	1.1873	0.3600	-0.0095	0.0068	0.1600	-0.0133184	0.0135568	0.33	-0.0043902	0.00328653	0.18
rs930295	690522	2	50233352	A	C				rs930295	A	C	1.1659	1.3809	0.4000	0.0055	0.0079	0.4800	0.0140261	0.0157957	0.37	-0.00090965	0.00382379	0.81
rs9332817	530761	11	118365210	G	C				rs9332817	C	G	1.6436	6.7311	0.8100	0.0371	0.0386	0.3400	-0.0062369	0.0770823	0.94	0.00424875	0.0186016	0.82
rs934515	686026	2	79482643	A	G				rs934515	G	A	1.1634	1.4875	0.4300	-0.0052	0.0085	0.5400	0.0263443	0.0169706	0.12	0.00779775	0.00409201	0.057
rs9349239	687917	6	42676480	A	G				rs9349239	G	A	1.1293	1.1884	0.3400	-0.0056	0.0068	0.4100	0.00193036	0.0135408	0.89	-0.00186353	0.00329196	0.57
rs9362662	683953	6	90296588	A	G	*																	
rs9367368	786723	6	13189275	T	C	*																	
rs9370042	691448	6	51546123	C	G				rs9370042	C	G	1.3816	1.2107	0.2500	-0.0070	0.0069	0.3100	0.00591389	0.0137986	0.67	-0.00319873	0.00333905	0.34
rs9394312	691704	6	35672330	C	G				rs9394312	C	G	1.6952	1.2466	0.1700	-0.0017	0.0071	0.8100	0.0315909	0.0141725	0.026	-0.00624603	0.00345867	0.071
rs9396763	684286	6	9952059	A	C				rs9396763	A	C	1.8664	1.7201	0.2800	-0.0011	0.0098	0.9100	0.0355233	0.0195679	0.069	-0.0033508	0.00474404	0.48
rs9419958	691816	10	105675946	C	T				rs9419958	T	C	3.2262	3.6491	0.3800	0.0004	0.0207	0.9900	0.00520395	0.0413572	0.9	0.00114535	0.00984661	0.91
rs9426003	682054	1	34602870	G	A				rs9426003	A	G	0.5418	1.4116	0.7000	0.0202	0.0081	0.0120	-0.0161801	0.0161138	0.32	-0.00112991	0.00391225	0.77
rs9460306	691559	6	19211776	T	C				rs9460306	C	T	0.1541	1.3421	0.9100	0.0023	0.0076	0.7600	-0.0167012	0.0152855	0.27	0.00765248	0.00371914	0.04
rs9463175	771669	6	9510030	C	T				rs9463175	T	C	1.6747	1.6097	0.3000	-0.0071	0.0092	0.4400	0.0033477	0.0184555	0.86	-0.00246738	0.00446031	0.58
rs946526	674813	1	46487168	C	T	*																	
rs9470086	738213	6	35709678	A	C	*																	
rs9475173	678491	6	55013291	A	G	*																	
rs9507983	692568	13	28620036	C	T	*	rs1933437	0.60	rs1933437	A	G	3.3608	1.4263	0.0180	-0.0106	0.0081	0.2000	-0.023143	0.0162851	0.16	0.00106395	0.00394227	0.79
rs952159	691197	16	71965915	G	A	*																	
rs9527706	794927	13	58402479	G	A				rs9527706	A	G	1.5233	2.0775	0.4600	0.0074	0.0118	0.5300	-0.0120393	0.0236414	0.61	0.00379443	0.00579277	0.51
rs9530843	674325	13	79563749	A	C	*																	
rs9538141	676674	13	59178258	A	G				rs9538141	A	G	0.0696	1.2552	0.9600	0.0089	0.0072	0.2200	-0.012863	0.0143443	0.37	0.00109698	0.00347952	0.75
rs954018	795474	18	42598463	G	A				rs954018	G	A	0.0433	1.1740	0.9700	0.0006	0.0067	0.9200	0.00518407	0.0133943	0.7	-0.00116677	0.00325697	0.72
rs9540493	785493	13	66205704	A	G				rs9540493	G	A	2.2581	1.3054	0.0840	-0.0010	0.0074	0.8900	0.00898622	0.0148868	0.55	-0.00321776	0.00360738	0.37
rs9544915	690945	13	36230485	T	C				rs9544915	T	C	0.3026	1.7208	0.8600	-0.0051	0.0099	0.6100	-0.0162887	0.019702	0.41	0.00319681	0.00478883	0.5
rs9554263	685732	13	28681228	G	C	*	rs4397962	0.98	rs4397962	T	C	2.2811	1.2601	0.0700	0.0034	0.0072	0.6300	-0.0218724	0.0143348	0.13	0.00144712	0.00345978	0.68
rs9571687	690974	13	67472713	C	A				rs9571687	A	C	1.0501	1.2183	0.3900	0.0035	0.0070	0.6200	0.0118924	0.0139103	0.39	0.00457724	0.00338046	0.18
rs9595908	794175	13	33184288	T	C	*	rs9591180	0.99	rs9591180	G	C	2.0373	1.6277	0.2100	-0.0099	0.0093	0.2900	-0.0139092	0.0184758	0.45	-0.00311574	0.00447794	0.49
rs9603697	688189	13	40783323	T	C	*	rs1336486	0.98	rs1336486	T	G	0.5420	1.1845	0.6500	0.0007	0.0067	0.9200	0.0305972	0.0134657	0.023	-0.00621795	0.00325989	0.056
rs9615905	690274	22	48875699	T	C	*																	
rs9630985	690619	2	181607676	C	A				rs9630985	A	C	0.9499	1.3299	0.4800	-0.0039	0.0076	0.6100	-0.0028279	0.0152099	0.85	-0.00325313	0.00367183	0.38
rs9657542	684813	8	10864363	G	C	*																	
rs9675376	783569	18	57969244	A	G	*	rs73455661	0.98	rs73455661	A	G	0.3781	1.8119	0.8300	-0.0090	0.0103	0.3800	-0.0072773	0.0206271	0.72	-0.00242065	0.00505186	0.63
rs967605	691316	1	23399932	C	T	*																	
rs9688431	789356	6	73922654	T	C				rs9688431	T	C	2.5342	1.7437	0.1500	0.0005	0.0100	0.9600	0.00223047	0.020026	0.91	0.0108034	0.00491156	0.028
rs968972	688147	2	51171962	A	G				rs968972	G	A	0.9242	1.2145	0.4500	-0.0063	0.0069	0.3600	0.0166552	0.0138571	0.23	0.00414614	0.00335791	0.22
rs9714342	524851	3	90428286	C	T	*	rs12494891	0.97	rs12494891	T	G	0.2485	1.2340	0.8400	-0.0096	0.0071	0.1700	-0.0187535	0.0141059	0.18	-0.00731508	0.00342939	0.033
rs972283	795592	7	130466854	A	G	*	rs10954284	0.90	rs10954284	T	A	0.6166	1.2642	0.6300	-0.0178	0.0072	0.0140	0.0195105	0.0143909	0.18	-0.00014207	0.00348125	0.97
rs972540	794958	2	207244783	G	A				rs972540	G	A	1.6454	1.4603	0.2600	0.0096	0.0083	0.2500	-0.0190807	0.0165918	0.25	4.29701E-05	0.00402256	0.99
rs977540	789485	16	9724750	A	G				rs977540	A	G	2.8305	1.2073	0.0190	0.0075	0.0069	0.2800	0.00675715	0.0137977	0.62	-0.00372478	0.00334006	0.26
rs9787495	689779	10	103206115	G	A				rs9787495	G	A	0.3856	1.7665	0.8300	0.0043	0.0101	0.6700	-0.0196262	0.0201064	0.33	-0.00262034	0.00487855	0.59
rs980329	692518	2	60285100	C	T	*	rs1861151	0.94	rs1861151	C	T	1.2807	1.3316	0.3400	-0.0137	0.0076	0.0720	0.0162344	0.0152005	0.29	0.00145893	0.00366984	0.69
rs9806058	690566	14	35673470	A	T	*																	
rs9809534	692357	3	125189782	C	G				rs9809534	C	G	1.9809	1.6839	0.2400	0.0051	0.0096	0.6000	0.00227843	0.0191858	0.91	-0.0032748	0.0046683	0.48
rs9814633	691120	3	41310470	A	G	*																	
rs9816226	778333	3	185834499	T	A				rs9816226	A	T	1.8428	2.1434	0.3900	-0.0155	0.0123	0.2100	0.0071269	0.0245761	0.77	-0.00296596	0.00600533	0.62
rs9827072	669035	3	51184893	G	A	*	rs4293735	1.00	rs4293735	A	G	5.9512	2.3690	0.0120	0.0338	0.0135	0.0130	0.037607	0.0269851	0.16	-0.00275933	0.00660426	0.68
rs9832305	678660	3	89392778	C	T				rs9832305	C	T	1.7300	1.8943	0.3600	0.0173	0.0108	0.1100	-0.0252217	0.0215116	0.24	0.0105103	0.00519848	0.043
rs9838283	692402	3	50820486	A	G	*	rs11130263	1.00	rs11130263	G	A	7.5249	2.6169	0.0040	0.0309	0.0150	0.0390	0.0297229	0.029873	0.32	0.00515611	0.00729927	0.48
rs9846123	692518	3	49088112	C	T				rs9846123	C	T	3.6836	2.8787	0.2000	0.0062	0.0164	0.7100	-0.0269241	0.0326687	0.41	-0.00060323	0.0079066	0.94
rs984902	692068	2	81358877	A	T				rs984902	T	A	3.4261	4.8245	0.4800	0.0140	0.0277	0.6100	-0.0187952	0.0552384	0.73	-0.00192608	0.0132349	0.88
rs987237	795612	6	50803050	G	A				rs987237	G	A	0.2532	1.6998	0.8800	-0.0067	0.0097	0.4900	-0.008303	0.0193959	0.67	0.00617134	0.00468963	0.19
rs9881036	787623	3	136845666	G	A	*																	
rs9905991	687945	17	80052073	A	G				rs9905991	G	A	1.7488	1.6480	0.2900	0.0087	0.0094	0.3600	-0.0152265	0.0187462	0.42	0.00191956	0.00454209	0.67
rs9922708	794334	16	53831146	T	C	*	rs9931494	0.89	rs9931494	G	C	1.2541	1.5159	0.4100	0.0044	0.0086	0.6100	-0.0036097	0.0172542	0.83	-0.00081249	0.00416458	0.85
rs9927848	687060	16	23833071	C	A				rs9927848	C	A	2.0166	1.3645	0.1400	0.0045	0.0078	0.5700	0.0254876	0.0156166	0.1	0.00174859	0.00377798	0.64
rs9931164	749059	16	53825238	A	G				rs9931164	G	A	1.3628	2.8752	0.6400	0.0014	0.0163	0.9300	0.0531151	0.0327706	0.11	-0.00611717	0.00804771	0.45
rs9931407	680058	16	67290155	C	T	*																	
rs9931967	684371	16	20375351	T	G	*	rs12926567	0.85	rs12926567	T	C	1.4677	1.6760	0.3800	-0.0033	0.0096	0.7300	0.0139358	0.0191949	0.47	-0.00398507	0.00463892	0.39
rs993931	676675	7	71888157	G	A	*																	
rs993954	689310	2	188325474	T	G	*	rs763684	0.99	rs763684	T	G	0.9324	1.2345	0.4500	0.0022	0.0070	0.7500	0.0091347	0.0140413	0.52	0.00042917	0.00341235	0.9
rs9951893	682456	18	60739250	C	T				rs9951893	T	C	2.5277	1.2180	0.0380	-0.0047	0.0070	0.5000	-0.0055746	0.0139354	0.69	-0.00563529	0.00339117	0.097
rs9961813	792281	18	57853056	A	C	*	rs73961491	0.98	rs73961491	T	C	1.7722	4.5442	0.7000	-0.0458	0.0259	0.0770	-0.0404444	0.0519811	0.44	0.00405175	0.0125932	0.75
rs9979651	689520	21	34153330	G	C				rs9979651	G	C	1.1460	2.3719	0.6300	0.0000	0.0135	1.0000	0.049239	0.0268916	0.067	0.00575766	0.00648394	0.37
rs998584	757136	6	43757896	C	A				rs998584	A	C	0.3032	1.1996	0.8000	0.0145	0.0069	0.0350	-0.0073679	0.0137138	0.59	0.00705043	0.00333882	0.035
rs998732	793852	19	19378671	A	G	*	rs72999053	0.99	rs72999053	A	G	2.0413	5.1179	0.6900	0.0151	0.0292	0.6100	-0.0413667	0.0582864	0.48	0.00179086	0.0153032	0.91
