# Supplementary material for: Investigating causal relationships between obesity and skin barrier function in a multi-ethnic Asian general population cohort
Source: Int J Obes (Lond). 2023 Jul 21;47(10):963–9. doi: 10.1038/s41366-023-01343-z (PMC10511308; doi:10.1038/s41366-023-01343-z)
Supplement: Supplementary file 3 — Supplementary Table 2 [file 41366_2023_1343_MOESM3_ESM.pdf]

Supplementary Table 2a. List of SNPs for Education (Number of years education completed) and their effects on TEWL

SNP	effect_allele.Edu	other_allel	effect_alle	other_allel	beta.Educa	beta.TEWL	eaf.Educat	eaf.TEWL	chr.TEWL	pos.TEWL	se.TEWL	pval.TEWL	chr.Educat	pos.Educa	se.Educati	pval.Education
chr1:107050579:t:a	A	T	A	T	0.0086	0.0142835	0.6615	0.698127	1	107050579	0.007455	0.055	1	1.08E+08	0.0015	5.99E-09
chr1:110177778:a:g	A	G	A	G	0.0078	0.00855781	0.4556	0.684198	1	110177778	0.007301	0.24	1	1.11E+08	0.0014	3.29E-08
chr1:113832344:c:g	C	G	C	G	0.0248	-0.0372184	0.9689	0.992555	1	113832344	0.040036	0.35	1	1.14E+08	0.0041	1.44E-09
chr1:11471002:g:a	A	G	A	G	0.0099	-0.00895906	0.2128	0.124159	1	11471002	0.010423	0.39	1	11531059	0.0018	3.35E-08
chr1:159197500:a:c	A	C	A	C	0.0138	0.0251142	0.7606	0.954611	1	159197500	0.016411	0.13	1	1.59E+08	0.0016	5.42E-17
chr1:159430569:c:t	T	C	T	C	0.0083	0.00381074	0.3346	0.727666	1	159430569	0.007529	0.61	1	1.59E+08	0.0015	2.14E-08
chr1:174107726:g:c	C	G	C	G	0.0094	-0.012694	0.4478	0.630403	1	174107726	0.007001	0.07	1	1.74E+08	0.0014	6.42E-11
chr1:181667462:a:g	A	G	A	G	0.0085	-0.00934562	0.3386	0.306676	1	181667462	0.007242	0.2	1	1.82E+08	0.0015	9.94E-09
chr1:197938424:a:g	A	G	A	G	0.0111	0.0056505	0.7635	0.733429	1	197938424	0.0077	0.46	1	1.98E+08	0.0017	2.05E-11
chr1:201876447:c:a	A	C	A	C	-0.0115	0.0132765	0.3294	0.104467	1	201876447	0.011103	0.23	1	2.02E+08	0.0015	1.13E-14
chr1:20214877:a:g	A	G	A	G	-0.0089	-0.00843214	0.2525	0.517771	1	20214877	0.006899	0.22	1	20541370	0.0016	4.11E-08
chr1:204617919:g:a	A	G	A	G	0.0199	0.00388288	0.2131	0.298031	1	204617919	0.007683	0.61	1	2.05E+08	0.0017	4.45E-31
chr1:211236502:c:t	T	C	T	C	-0.009	-0.00303241	0.4325	0.413785	1	211236502	0.007007	0.67	1	2.11E+08	0.0014	3.88E-10
chr1:212978238:t:g	T	G	T	G	-0.0091	-0.000542258	0.2902	0.338136	1	212978238	0.007264	0.94	1	2.13E+08	0.0015	3.52E-09
chr1:216519921:t:c	T	C	T	C	-0.0095	-0.00551871	0.6911	0.450528	1	216519921	0.006889	0.42	1	2.17E+08	0.0015	5.25E-10
chr1:235433937:t:g	T	G	T	G	-0.0098	0.00397997	0.489	0.336695	1	235433937	0.007099	0.58	1	2.36E+08	0.0014	2.30E-12
chr1:43484139:a:g	A	G	A	G	0.0149	0.0121684	0.3223	0.055716	1	43484139	0.01498	0.42	1	43949810	0.0015	4.18E-23
chr1:43999747:t:c	T	C	T	C	-0.0173	0.00621345	0.1076	0.105427	1	43999747	0.011289	0.58	1	44465419	0.0023	2.19E-14
chr1:4509376:t:a	A	T	A	T	-0.0085	0.00690052	0.7201	0.825408	1	4509376	0.008835	0.43	1	4569436	0.0016	4.98E-08
chr1:45491937:g:a	A	G	A	G	0.0104	-0.00262107	0.2228	0.329011	1	45491937	0.007284	0.72	1	45957609	0.0017	7.62E-10
chr1:57822645:t:c	T	C	T	C	0.0092	5.24E-06	0.6963	0.881604	1	57822645	0.010938	1	1	58288317	0.0015	1.84E-09
chr1:68865616:t:a	A	T	A	T	0.0148	-0.0150963	0.1013	0.036023	1	68865616	0.018626	0.42	1	69331299	0.0023	1.78E-10
chr1:70100338:g:t	T	G	T	G	0.0081	-0.00966262	0.6532	0.504563	1	70100338	0.006814	0.16	1	70566021	0.0015	4.02E-08
chr1:72044059:c:g	C	G	C	G	0.0084	-0.0118569	0.503	0.867675	1	72044059	0.009869	0.23	1	72509742	0.0014	4.16E-09
chr1:73512804:g:c	A	C	G	C	-0.0153	-0.0360568	0.8881	0.009606	1	73512804	0.032921	0.27	1	73978572	0.0022	6.46E-12
chr1:90631242:c:a	A	C	A	C	-0.0097	-0.00286798	0.6926	0.899135	1	90631242	0.011364	0.8	1	91096799	0.0015	1.53E-10
chr1:93282126:t:g	T	G	T	G	0.0124	0.00516102	0.6406	0.65658	1	93282126	0.007235	0.48	1	93747683	0.0015	2.07E-17
chr10:102190030:c:t	T	C	T	C	0.0132	0.00637181	0.2225	0.115754	10	102190030	0.010225	0.53	10	1.04E+08	0.0024	1.96E-08
chr10:103236552:g:a	A	G	A	G	-0.0111	-0.00536256	0.6725	0.599183	10	103236552	0.006977	0.44	10	1.05E+08	0.002	1.58E-08
chr10:104694914:t:c	T	C	T	C	-0.0106	-0.000176357	0.5838	0.315802	10	104694914	0.007301	0.98	10	1.06E+08	0.0014	9.94E-14
chr10:104978917:t:c	T	C	T	C	-0.0139	-0.0167303	0.7832	0.966619	10	104978917	0.018333	0.36	10	1.07E+08	0.0022	1.94E-10
chr10:105860174:t:c	T	C	T	C	-0.0098	0.00787331	0.7441	0.960615	10	105860174	0.017348	0.65	10	1.08E+08	0.0016	1.87E-09
chr10:109122310:c:t	T	C	T	C	-0.009	-0.0124935	0.2561	0.276417	10	109122310	0.007669	0.1	10	1.11E+08	0.0016	2.35E-08
chr10:116790510:c:g	C	G	C	G	0.0078	0.00492895	0.5663	0.560999	10	116790510	0.006802	0.47	10	1.19E+08	0.0014	4.24E-08
chr10:31997014:t:a	A	T	A	T	0.0101	0.00984846	0.7498	0.976705	10	31997014	0.022797	0.67	10	32285942	0.0016	4.80E-10
chr10:3989327:g:a	A	G	A	G	0.01	-0.00455335	0.3818	0.53146	10	3989327	0.006763	0.5	10	4031519	0.0014	3.97E-12
chr10:61839198:t:c	T	C	T	C	-0.008	0.0176481	0.5019	0.568204	10	61839198	0.006977	0.011	10	63598957	0.0014	9.88E-09
chr10:63431885:g:t	T	G	T	G	0.0133	-0.00137707	0.5124	0.650576	10	63431885	0.007114	0.85	10	65191645	0.0014	2.55E-21
chr10:65054170:c:g	C	G	C	G	0.0113	0.0037324	0.6512	0.650336	10	65054170	0.007265	0.61	10	66813928	0.002	7.47E-09
chr10:75160441:t:c	T	C	T	C	0.0085	0.000207904	0.5884	0.137608	10	75160441	0.009425	0.98	10	76920199	0.0014	2.08E-09
chr11:109418812:c:t	T	C	T	C	-0.01	-0.00280263	0.3038	0.29683	11	109418812	0.007595	0.71	11	1.09E+08	0.0016	1.32E-10
chr11:111086611:g:c	C	G	C	G	-0.0108	-0.00817253	0.1721	0.027858	11	111086611	0.020949	0.7	11	1.11E+08	0.0019	6.45E-09
chr11:114160077:a:g	A	G	A	G	0.0083	0.0054351	0.3892	0.457973	11	114160077	0.006849	0.43	11	1.14E+08	0.0015	8.89E-09
chr11:115602656:c:t	T	C	T	C	0.008	-0.00713764	0.4773	0.231268	11	115602656	0.007968	0.37	11	1.15E+08	0.0014	1.11E-08
chr11:12859851:g:c	C	G	C	G	0.0133	-0.010039	0.6911	0.721902	11	12859851	0.007466	0.18	11	12881398	0.0015	1.79E-18
chr11:131419926:c:a	A	C	A	C	0.0111	0.00760747	0.6475	0.869356	11	131419926	0.01011	0.45	11	1.31E+08	0.0015	3.91E-14
chr11:132632317:c:t	T	C	T	C	0.0091	0.0110764	0.7606	0.589097	11	132632317	0.006959	0.11	11	1.33E+08	0.0016	2.81E-08
chr11:132789027:c:t	A	G	A	G	0.0134	-0.00658215	0.9103	0.010567	11	132789027	0.033005	0.84	11	1.33E+08	0.0025	4.40E-08
chr11:20937639:a:t	A	T	A	T	-0.0097	-0.0019416	0.2618	0.408982	11	20937639	0.006885	0.78	11	20959185	0.0016	1.11E-09
chr11:27622178:c:t	T	C	T	C	0.0106	-0.00397656	0.1845	0.447406	11	27622178	0.006821	0.56	11	27643725	0.0018	4.07E-09
chr11:65567241:c:g	C	G	C	G	-0.0175	-0.0480671	0.9432	0.987032	11	65567241	0.02994	0.11	11	65334712	0.003	7.90E-09
chr11:66325096:c:g	C	G	C	G	0.0141	-0.000681877	0.7609	0.710375	11	66325096	0.007483	0.93	11	66092567	0.0017	3.10E-17
chr11:79433812:c:g	C	G	C	G	-0.015	0.0117596	0.8925	0.966138	11	79433812	0.018756	0.53	11	79144856	0.0023	3.12E-11

chr11:88174544:c:t	T	C	T	C	-0.0082	0.00241327	0.4427	0.289145	11	88174544	0.007626	0.75	11	87907712	0.0014	7.10E-09
chr12:13264683:g:c	C	G	C	G	0.0142	0.00209099	0.8887	0.911143	12	13264683	0.011872	0.86	12	13417617	0.0022	1.69E-10
chr12:23544791:c:a	A	C	A	C	-0.0085	-0.0158248	0.3062	0.311479	12	23544791	0.00732	0.031	12	23697725	0.0015	2.33E-08
chr12:26531410:g:a	A	G	A	G	-0.0094	0.0114212	0.262	0.034582	12	26531410	0.018705	0.54	12	26684343	0.0016	6.71E-09
chr12:32316667:a:g	A	G	A	G	0.0104	-0.00589938	0.2257	0.532661	12	32316667	0.006584	0.37	12	32469601	0.0017	5.03E-10
chr12:49549339:a:g	A	G	A	G	-0.011	0.000654904	0.8193	0.898895	12	49549339	0.011334	0.95	12	49943122	0.0018	2.08E-09
chr12:57896495:g:a	A	G	A	G	-0.0097	0.000326002	0.3797	0.660423	12	57896495	0.007201	0.96	12	58290278	0.0014	1.75E-11
chr12:67304076:g:a	A	G	A	G	0.0082	-0.000786932	0.645	0.383525	12	67304076	0.007073	0.91	12	67697856	0.0015	2.09E-08
chr12:74509286:c:t	A	G	A	G	-0.017	0.0232279	0.0717	0.007445	12	74509286	0.038845	0.55	12	74936526	0.0027	4.10E-10
chr12:78236137:c:t	T	C	T	C	-0.0114	-0.00129201	0.1341	0.45341	12	78236137	0.006825	0.85	12	78629917	0.0021	3.24E-08
chr13:100101542:c:g	C	G	C	G	0.0109	-0.00104139	0.7355	0.324928	13	100101542	0.007362	0.89	13	1.01E+08	0.0016	7.77E-12
chr13:55147898:a:c	A	C	A	C	-0.0108	-0.00540436	0.6449	0.513929	13	55147898	0.006867	0.43	13	55722033	0.0015	1.71E-13
chr13:59847085:a:t	A	T	A	T	0.0099	0.00392532	0.362	0.539866	13	59847085	0.006718	0.56	13	60421219	0.0015	2.22E-11
chr13:66346783:t:a	A	T	A	T	-0.0089	-0.000150495	0.4893	0.646494	13	66346783	0.007151	0.98	13	66920915	0.0014	1.88E-10
chr13:80896978:g:c	C	G	C	G	-0.0103	0.00259908	0.5204	0.502642	13	80896978	0.006801	0.7	13	81471113	0.0014	2.89E-13
chr13:90963584:g:a	A	G	A	G	-0.0128	0.0105093	0.2761	0.192363	13	90963584	0.00857	0.22	13	91615838	0.0016	3.42E-16
chr13:91401392:a:g	A	G	A	G	0.0182	0.0124069	0.0912	0.98391	13	91401392	0.027475	0.65	13	92044587	0.0025	1.09E-13
chr13:98447792:c:t	T	C	T	C	-0.011	-0.0106034	0.5233	0.432517	13	98447792	0.00689	0.12	13	99100046	0.0014	5.96E-15
chr14:103588088:t:c	T	C	T	C	-0.0106	0.0010578	0.3009	0.496158	14	103588088	0.006924	0.88	14	1.04E+08	0.0015	4.07E-12
chr14:21462773:g:t	T	G	T	G	-0.0122	0.00891413	0.8031	0.841499	14	21462773	0.009327	0.34	14	21930932	0.0018	4.78E-12
chr14:26510206:t:c	T	C	T	C	0.0145	0.00745833	0.2455	0.416427	14	26510206	0.006857	0.28	14	26979412	0.0016	4.81E-19
chr14:34619429:t:a	A	T	A	T	0.008	-0.0220089	0.5687	0.873199	14	34619429	0.010273	0.032	14	35088635	0.0014	1.58E-08
chr14:56813328:c:t	T	C	T	C	-0.0144	0.0109343	0.1511	0.037704	14	56813328	0.017585	0.53	14	57280046	0.002	2.24E-13
chr14:60537171:g:c	C	G	C	G	-0.0103	-0.02003	0.6019	0.230067	14	60537171	0.00803	0.013	14	61003889	0.0014	8.06E-13
chr14:71968988:c:t	T	C	T	C	0.012	0.00768843	0.1991	0.048991	14	71968988	0.015646	0.62	14	72435705	0.0018	9.42E-12
chr14:84646294:g:t	T	G	T	G	0.0127	-0.000123275	0.1412	0.01561	14	84646294	0.025618	1	14	85112638	0.002	4.94E-10
chr14:93821484:t:c	T	C	T	C	0.0086	-0.00101169	0.3931	0.198847	14	93821484	0.008693	0.91	14	94287830	0.0014	2.49E-09
chr14:99283827:c:t	T	C	T	C	0.0121	0.0378272	0.4161	0.059078	14	99283827	0.014314	0.0082	14	99750164	0.0015	9.20E-17
chr15:38131529:c:t	T	C	T	C	0.0092	-0.0025132	0.2819	0.352305	15	38131529	0.007287	0.73	15	38423730	0.0016	4.21E-09
chr15:51095207:g:t	T	G	T	G	0.0113	-0.0120296	0.1549	0.017771	15	51095207	0.023156	0.6	15	51387404	0.0019	4.83E-09
chr15:57261634:a:t	A	T	A	T	0.0093	-0.0119571	0.4151	0.619837	15	57261634	0.007149	0.094	15	57553832	0.0014	5.76E-11
chr15:64731033:g:t	T	G	T	G	0.0174	-0.00699322	0.0683	0.709414	15	64731033	0.006923	0.31	15	65023232	0.0028	7.76E-10
chr15:83753754:c:g	C	G	C	G	-0.0096	0.0191097	0.2277	0.040586	15	83753754	0.01715	0.27	15	84422506	0.0017	1.10E-08
chr16:11991486:a:c	A	C	A	C	-0.0083	-0.0108917	0.3609	0.643372	16	11991486	0.007178	0.13	16	12085343	0.0015	1.36E-08
chr16:12137882:g:t	T	G	T	G	-0.0117	-0.0123288	0.6825	0.979107	16	12137882	0.023702	0.6	16	12231739	0.0015	1.41E-14
chr16:14471531:c:t	A	G	A	G	0.0105	0.022762	0.2308	0.013209	16	14471531	0.029194	0.44	16	14674204	0.0017	2.66E-10
chr16:15059860:t:c	T	C	T	C	-0.013	-0.00154232	0.149	0.237512	16	15059860	0.007955	0.85	16	15153717	0.002	6.22E-11
chr16:19255822:c:g	C	G	C	G	0.0093	-0.00672096	0.3828	0.021374	16	19255822	0.022931	0.77	16	19267144	0.0015	1.81E-10
chr16:24700485:c:g	C	G	C	G	0.0112	0.000109927	0.3789	0.502161	16	24700485	0.006723	0.99	16	24711806	0.0014	9.77E-15
chr16:30622979:a:g	A	G	A	G	0.0082	0.00198059	0.5298	0.814361	16	30622979	0.007955	0.8	16	30634300	0.0014	5.11E-09
chr16:3512075:a:g	A	G	A	G	0.0101	-0.000864393	0.8017	0.731988	16	3512075	0.007722	0.91	16	3562075	0.0018	9.04E-09
chr16:51154521:g:a	A	G	A	G	0.0112	-0.00646555	0.5959	0.597262	16	51154521	0.006989	0.35	16	51188432	0.0014	7.25E-15
chr16:62159736:t:g	T	G	T	G	-0.0099	-0.000476191	0.5688	0.746158	16	62159736	0.007796	0.95	16	62193640	0.0014	2.31E-12
chr16:65414176:t:g	T	G	T	G	-0.011	0.00983135	0.8092	0.696926	16	65414176	0.007333	0.18	16	65448079	0.0018	8.19E-10
chr16:71826105:g:a	A	G	A	G	0.0099	0.00772641	0.7453	0.966859	16	71826105	0.019217	0.69	16	71860008	0.0016	9.28E-10
chr16:7199471:g:a	A	G	A	G	-0.0091	-0.00702024	0.3236	0.075648	16	7199471	0.012946	0.59	16	7249472	0.0015	1.12E-09
chr16:72176966:g:a	A	G	A	G	0.0127	0.00298832	0.1852	0.065322	16	72176966	0.013566	0.83	16	72210865	0.0018	2.36E-12
chr16:7614873:c:t	T	C	T	C	-0.0102	0.0017485	0.361	0.177714	16	7614873	0.00882	0.84	16	7664875	0.0015	3.18E-12
chr16:87410128:c:t	T	C	T	C	0.0092	0.000951144	0.5626	0.01489	16	87410128	0.02762	0.97	16	87443734	0.0014	9.20E-11
chr17:20011233:c:t	T	C	T	C	-0.0078	-0.00797918	0.5214	0.205572	17	20011233	0.008555	0.35	17	19914546	0.0014	2.59E-08
chr17:39613752:c:t	T	C	T	C	0.0102	-0.000474257	0.6947	0.424592	17	39613752	0.006802	0.94	17	37770005	0.0015	1.81E-11
chr17:44981374:t:c	T	C	T	C	0.0087	-0.00101919	0.3463	0.166186	17	44981374	0.009301	0.91	17	43058742	0.0015	4.11E-09
chr17:48951110:a:g	A	G	A	G	0.0088	-0.00547289	0.4756	0.337896	17	48951110	0.007034	0.44	17	47028472	0.0014	4.10E-10
chr17:52196810:c:t	T	C	T	C	-0.0087	0.00329967	0.4176	0.320125	17	52196810	0.007577	0.66	17	50274170	0.0014	7.90E-10
chr18:23494291:g:a	A	G	A	G	-0.011	-0.0119677	0.4067	0.085975	18	23494291	0.012013	0.32	18	21074255	0.0014	1.47E-14

chr18:25044744:g:a	A	G	A	G	-0.0124	-0.00868091	0.5714	0.701009	18	25044744	0.007387	0.24	18	22624708	0.0014	2.61E-18
chr18:33612565:a:g	A	G	A	G	-0.008	0.0133491	0.5847	0.916427	18	33612565	0.012287	0.28	18	31192529	0.0014	1.89E-08
chr18:38546022:g:a	A	G	A	G	0.0099	-0.00479754	0.7506	0.612152	18	38546022	0.006962	0.49	18	36125986	0.0016	8.80E-10
chr18:38985457:c:t	T	C	T	C	-0.0103	-0.000684864	0.2594	0.330692	18	38985457	0.007386	0.93	18	36565421	0.0016	9.85E-11
chr18:51253303:c:t	T	C	T	C	-0.0087	-0.0115754	0.4856	0.362392	18	51253303	0.007125	0.1	18	48779673	0.0014	4.86E-10
chr18:79818191:c:a	A	C	A	C	-0.013	0.00350352	0.2761	0.409462	18	79818191	0.007009	0.62	18	77578191	0.0016	9.27E-17
chr19:13101211:c:t	T	C	T	C	-0.0149	-0.00289193	0.1141	0.022815	19	13101211	0.022787	0.9	19	13212025	0.0023	3.81E-11
chr19:32481390:c:t	T	C	T	C	0.0124	-0.0102195	0.8208	0.855908	19	32481390	0.009769	0.3	19	32972296	0.0018	9.92E-12
chr19:4954443:g:a	A	G	A	G	-0.0108	-0.00392281	0.1918	0.032181	19	4954443	0.018894	0.84	19	4954455	0.0018	1.29E-09
chr19:54449569:c:t	T	C	T	C	-0.0319	0.0100388	0.0268	0.045629	19	54449569	0.016383	0.54	19	54960747	0.0045	2.06E-12
chr2:100205083:t:c	T	C	T	C	-0.0215	0.00603644	0.6017	0.45389	2	100205083	0.006747	0.37	2	1.01E+08	0.0014	1.05E-50
chr2:100712266:g:t	T	G	T	G	0.0187	-0.000120088	0.0907	0.03098	2	100712266	0.019955	1	2	1.01E+08	0.0025	4.17E-14
chr2:103443545:c:a	A	C	A	C	0.0185	-0.00358247	0.0876	0.005524	2	103443545	0.044526	0.94	2	1.04E+08	0.0025	3.72E-13
chr2:106947659:t:c	T	C	T	C	-0.0107	0.0217769	0.3962	0.420749	2	106947659	0.006977	0.0018	2	1.08E+08	0.0014	6.88E-14
chr2:124234310:a:c	A	C	A	C	-0.0117	-0.00489337	0.71	0.603987	2	124234310	0.007072	0.49	2	1.25E+08	0.0015	3.56E-14
chr2:125115631:a:c	A	C	A	C	-0.0091	-0.0262639	0.4718	0.160903	2	125115631	0.009229	0.0044	2	1.26E+08	0.0014	1.03E-10
chr2:125134647:c:t	A	G	A	G	-0.0165	-0.036875	0.0926	0.006484	2	125134647	0.041359	0.37	2	1.26E+08	0.0024	8.36E-12
chr2:138662822:t:c	T	C	T	C	-0.0104	-0.0148963	0.8287	0.956772	2	138662822	0.016958	0.38	2	1.39E+08	0.0019	2.19E-08
chr2:140839360:c:g	A	C	G	C	0.0182	-0.0318872	0.9333	0.014409	2	140839360	0.027192	0.24	2	1.42E+08	0.0028	8.97E-11
chr2:141311445:g:c	C	G	C	G	0.0088	0.000368301	0.3688	0.063881	2	141311445	0.01368	0.98	2	1.42E+08	0.0015	1.68E-09
chr2:142105261:a:g	A	G	A	G	0.0102	-0.00812595	0.8265	0.974784	2	142105261	0.02176	0.71	2	1.43E+08	0.0019	3.31E-08
chr2:144845739:c:t	A	G	A	G	-0.0193	0.0112131	0.0739	0.012968	2	144845739	0.030516	0.71	2	1.46E+08	0.0027	5.24E-13
chr2:145038684:g:a	A	G	A	G	0.0107	-0.000393596	0.4538	0.720221	2	145038684	0.00775	0.96	2	1.46E+08	0.0014	3.43E-14
chr2:160379470:g:c	C	G	C	G	-0.0097	0.00642985	0.2363	0.04611	2	160379470	0.016042	0.69	2	1.61E+08	0.0017	7.22E-09
chr2:161003249:t:c	T	C	T	C	0.0155	-7.78E-05	0.2447	0.179155	2	161003249	0.009029	0.99	2	1.62E+08	0.0022	1.17E-12
chr2:180750186:a:g	A	G	A	G	-0.0087	-0.00289716	0.3053	0.657061	2	180750186	0.007141	0.68	2	1.82E+08	0.0015	9.94E-09
chr2:192841785:t:c	T	C	T	C	-0.0082	-0.000880979	0.5121	0.54611	2	192841785	0.007004	0.9	2	1.94E+08	0.0014	7.47E-09
chr2:192988543:c:t	T	C	T	C	-0.0127	0.00743351	0.5224	0.59438	2	192988543	0.006793	0.27	2	1.94E+08	0.0014	1.37E-19
chr2:21105953:a:g	A	G	A	G	-0.0091	-0.00329403	0.3051	0.147214	2	21105953	0.009608	0.73	2	21328825	0.0015	2.34E-09
chr2:211734578:t:c	T	C	T	C	0.0137	0.0146194	0.6894	0.947166	2	211734578	0.014863	0.33	2	2.13E+08	0.0015	2.55E-19
chr2:211815798:t:g	T	G	T	G	-0.0085	-0.00248541	0.5755	0.581652	2	211815798	0.006928	0.72	2	2.13E+08	0.0014	2.55E-09
chr2:214144634:t:c	T	C	T	C	-0.0094	0.00603635	0.4915	0.769693	2	214144634	0.008113	0.46	2	2.15E+08	0.0014	2.46E-11
chr2:225425989:c:g	C	G	C	G	0.0119	-0.0123176	0.1842	0.392891	2	225425989	0.00717	0.086	2	2.26E+08	0.0018	5.34E-11
chr2:228197152:c:t	T	C	T	C	0.0153	-0.0272549	0.1124	0.098223	2	228197152	0.011619	0.019	2	2.29E+08	0.0022	6.29E-12
chr2:235835982:t:a	A	T	A	T	0.0104	-0.00085383	0.235	0.466619	2	235835982	0.006799	0.9	2	2.37E+08	0.0017	4.12E-10
chr2:241461182:a:g	A	G	A	G	0.0085	0.00495328	0.6337	0.866234	2	241461182	0.010206	0.63	2	2.42E+08	0.0015	6.48E-09
chr2:34154956:c:t	T	C	T	C	0.0082	0.00234626	0.3771	0.423631	2	34154956	0.006982	0.74	2	34380023	0.0015	2.11E-08
chr2:49681013:g:a	A	G	A	G	-0.008	0.00345546	0.5117	0.512968	2	49681013	0.006873	0.62	2	49908151	0.0014	1.15E-08
chr2:50767117:g:t	T	G	T	G	-0.0167	-0.0132881	0.1489	0.154419	2	50767117	0.009493	0.16	2	50994255	0.002	2.12E-17
chr2:57155998:g:t	T	G	T	G	-0.0089	0.0101584	0.4079	0.292027	2	57155998	0.007454	0.17	2	57383133	0.0014	3.95E-10
chr2:80194680:c:g	C	G	C	G	-0.016	-0.0433854	0.9129	0.991835	2	80194680	0.038259	0.26	2	80421805	0.0025	1.72E-10
chr2:80360966:a:c	A	C	A	C	0.0209	-0.0106762	0.9524	0.862392	2	80360966	0.009965	0.28	2	80588091	0.0033	2.14E-10
chr2:97712734:c:t	T	C	T	C	-0.0082	0.00470638	0.4076	0.23511	2	97712734	0.007891	0.55	2	98329197	0.0014	9.29E-09
chr2:99806696:c:t	T	C	T	C	0.0212	0.0200646	0.0378	0.010567	2	99806696	0.032312	0.53	2	1E+08	0.0037	1.36E-08
chr2:99896691:c:t	T	C	T	C	0.0135	-0.00721673	0.1713	0.271854	2	99896691	0.007604	0.34	2	1.01E+08	0.0019	7.68E-13
chr20:14936402:c:g	C	G	C	G	0.0096	0.012034	0.7193	0.495677	20	14936402	0.006845	0.079	20	14917048	0.0016	7.09E-10
chr20:15759100:t:c	T	C	T	C	-0.0083	0.0127253	0.6035	0.492795	20	15759100	0.006831	0.062	20	15739745	0.0014	9.72E-09
chr20:43346755:g:t	T	G	T	G	0.0171	-0.0290551	0.9248	0.990154	20	43346755	0.032673	0.37	20	41975395	0.0027	1.88E-10
chr20:45936043:g:a	A	G	A	G	-0.008	0.00431676	0.5129	0.529539	20	45936043	0.0069	0.53	20	44564682	0.0014	1.07E-08
chr20:48916462:a:g	A	G	A	G	-0.0085	0.00780313	0.3706	0.377762	20	48916462	0.007036	0.27	20	47532999	0.0015	4.38E-09
chr20:59644709:a:g	A	G	A	G	0.0129	-0.000204522	0.8445	0.510567	20	59644709	0.006838	0.98	20	58219764	0.0019	3.20E-11
chr21:33880392:t:c	T	C	T	C	0.0112	0.00348266	0.7915	0.840298	21	33880392	0.009352	0.71	21	35252696	0.0017	8.21E-11
chr21:41281640:t:c	T	C	T	C	0.0132	-0.012384	0.3597	0.137848	21	41281640	0.010044	0.22	21	42653567	0.0015	1.38E-19
chr21:45067815:t:c	T	C	T	C	-0.0178	0.00258569	0.9428	0.845821	21	45067815	0.009462	0.78	21	46487730	0.0031	5.92E-09
chr21:45075080:t:c	T	C	T	C	-0.0092	-0.0225823	0.3655	0.135447	21	45075080	0.00997	0.024	21	46494995	0.0015	3.37E-10

chr22:29484784:a:g	A	G	A	G	-0.0116	-0.0173458	0.7575	0.888088	22	29484784	0.010659	0.1	22	29880773	0.0016	1.27E-12
chr22:29806785:g:c	C	G	C	G	-0.0115	0.000923547	0.4442	0.617435	22	29806785	0.006862	0.89	22	30202774	0.0014	3.73E-16
chr22:33514750:c:t	T	C	T	C	-0.0082	-0.000860766	0.5542	0.486071	22	33514750	0.006969	0.9	22	33910736	0.0014	5.85E-09
chr22:50666252:g:a	A	G	A	G	0.0122	-0.0184473	0.4477	0.07733	22	50666252	0.012224	0.13	22	51104680	0.0014	8.31E-18
chr22:50697167:a:t	A	T	A	T	0.0162	-0.00680767	0.8844	0.782661	22	50697167	0.008217	0.41	22	51135595	0.0022	3.03E-13
chr22:50713203:c:t	T	C	T	C	-0.0134	0.01528	0.3818	0.824688	22	50713203	0.008905	0.086	22	51151631	0.0015	3.58E-20
chr3:116863339:t:c	T	C	T	C	-0.0111	-0.0241135	0.2863	0.21902	3	116863339	0.007914	0.0023	3	1.17E+08	0.0016	9.38E-13
chr3:118956349:a:g	A	G	A	G	-0.009	-0.0100739	0.7356	0.665946	3	118956349	0.007199	0.16	3	1.19E+08	0.0016	1.70E-08
chr3:122495198:c:t	T	C	T	C	0.0085	0.00401502	0.306	0.111671	3	122495198	0.010812	0.71	3	1.22E+08	0.0015	2.57E-08
chr3:123875572:t:g	T	G	T	G	-0.0104	-0.00751433	0.3617	0.927714	3	123875572	0.012436	0.55	3	1.24E+08	0.0015	9.77E-13
chr3:161104181:a:g	A	G	A	G	-0.0097	-0.00303506	0.5223	0.211575	3	161104181	0.008248	0.71	3	1.61E+08	0.0014	4.33E-12
chr3:16940191:g:t	T	G	T	G	-0.0111	0.00751507	0.1824	0.534582	3	16940191	0.006845	0.27	3	16981683	0.0018	8.69E-10
chr3:20589806:g:a	A	G	A	G	0.0086	0.00326792	0.4804	0.319645	3	20589806	0.007437	0.66	3	20631298	0.0014	1.02E-09
chr3:24908896:c:g	C	G	C	G	0.0103	-0.00449367	0.474	0.369116	3	24908896	0.007101	0.53	3	24950387	0.0014	2.40E-13
chr3:34229550:c:t	T	C	T	C	-0.009	-0.00781795	0.2542	0.235351	3	34229550	0.008243	0.34	3	34271042	0.0016	2.68E-08
chr3:48484583:a:t	A	T	A	T	-0.0121	0.00547075	0.6593	0.822286	3	48484583	0.008775	0.53	3	48525955	0.0015	4.72E-16
chr3:49894327:g:t	T	G	T	G	-0.0201	-0.00512648	0.1773	0.100624	3	49894327	0.011501	0.66	3	49931760	0.0018	1.35E-27
chr3:50150163:g:c	C	G	C	G	-0.0222	0.000280066	0.3468	0.020413	3	50150163	0.023953	0.99	3	50187596	0.0015	2.47E-51
chr3:65668805:a:g	A	G	A	G	-0.0107	-0.000933119	0.2992	0.043948	3	65668805	0.016222	0.95	3	65654480	0.0015	3.13E-12
chr3:68820772:t:a	A	T	A	T	-0.009	-0.00772746	0.5836	0.915946	3	68820772	0.012067	0.52	3	68869923	0.0014	2.51E-10
chr3:70491196:a:t	A	T	A	T	0.0104	0.046345	0.7926	0.945005	3	70491196	0.014483	0.0014	3	70540347	0.0017	1.64E-09
chr3:76180910:c:g	C	G	C	G	-0.0082	0.00610148	0.5502	0.810519	3	76180910	0.008604	0.48	3	76230061	0.0014	7.10E-09
chr3:78391414:g:a	A	G	A	G	0.0083	-0.00537062	0.3354	0.219981	3	78391414	0.008314	0.52	3	78440564	0.0015	4.64E-08
chr3:8216487:g:a	A	G	A	G	0.0108	0.00780378	0.3426	0.482229	3	8216487	0.006854	0.25	3	8258174	0.0015	3.10E-13
chr3:85347968:t:c	T	C	T	C	-0.0147	0.0149869	0.1117	0.060038	3	85347968	0.014177	0.29	3	85397118	0.0022	4.70E-11
chr4:105457127:t:c	T	C	T	C	-0.0083	-0.00345407	0.4083	0.632805	4	105457127	0.007136	0.63	4	1.06E+08	0.0014	6.23E-09
chr4:111402093:g:c	C	G	C	G	-0.0078	0.024907	0.48	0.748319	4	111402093	0.007897	0.0016	4	1.12E+08	0.0014	3.38E-08
chr4:122105714:t:c	T	C	T	C	0.0103	-0.00240477	0.3034	0.31244	4	122105714	0.007256	0.74	4	1.23E+08	0.0015	1.46E-11
chr4:136608692:t:a	A	T	A	T	-0.0106	-0.000714844	0.3909	0.520173	4	136608692	0.006821	0.92	4	1.38E+08	0.0014	2.75E-13
chr4:15056621:a:g	A	G	A	G	-0.009	0.0100391	0.3829	0.566763	4	15056621	0.006861	0.14	4	15058245	0.0014	4.41E-10
chr4:15400491:t:c	T	C	T	C	-0.008	0.00785143	0.6365	0.473823	4	15400491	0.006878	0.25	4	15402115	0.0015	3.42E-08
chr4:171505922:g:a	A	G	A	G	-0.0126	0.00451521	0.7504	0.438761	4	171505922	0.006903	0.51	4	1.72E+08	0.0016	6.24E-15
chr4:175726486:t:c	T	C	T	C	-0.0116	-0.00499434	0.5132	0.908021	4	175726486	0.011687	0.67	4	1.77E+08	0.0014	1.37E-16
chr4:176019184:g:t	T	G	T	G	0.0103	0.00124373	0.6254	0.450768	4	176019184	0.006906	0.86	4	1.77E+08	0.0014	1.47E-12
chr4:177619009:a:g	A	G	A	G	0.0085	0.00684622	0.6865	0.785062	4	177619009	0.008403	0.42	4	1.79E+08	0.0015	2.03E-08
chr4:21944310:g:t	T	G	T	G	0.0121	0.0199433	0.407	0.099424	4	21944310	0.011277	0.077	4	21945933	0.0014	4.88E-17
chr4:22163632:g:c	C	G	C	G	-0.0081	-0.0069358	0.3907	0.43708	4	22163632	0.006897	0.31	4	22165255	0.0014	1.82E-08
chr4:25407216:g:a	A	G	A	G	0.0104	0.0280211	0.2242	0.018492	4	25407216	0.02571	0.28	4	25408838	0.0017	2.75E-09
chr4:28607854:t:c	T	C	T	C	-0.0164	-0.0101796	0.1475	0.020173	4	28607854	0.024079	0.67	4	28609476	0.0027	9.45E-10
chr4:30969200:g:a	A	G	A	G	0.0109	0.00225256	0.6937	0.7817	4	30969200	0.008328	0.79	4	30970822	0.0015	8.69E-13
chr4:35444680:t:c	T	C	T	C	0.0078	0.00227823	0.5048	0.633525	4	35444680	0.007063	0.75	4	35446302	0.0014	2.50E-08
chr4:38590239:a:g	A	G	A	G	0.0088	-0.000856145	0.483	0.571326	4	38590239	0.006822	0.9	4	38591860	0.0014	3.02E-10
chr4:61261573:g:c	C	G	C	G	0.0084	-0.00317878	0.6293	0.690442	4	61261573	0.007341	0.66	4	62127291	0.0015	6.67E-09
chr4:91024421:c:a	A	C	A	C	-0.0083	0.00427447	0.441	0.673871	4	91024421	0.007183	0.55	4	91945572	0.0014	4.35E-09
chr5:104620815:c:g	C	G	C	G	0.0133	0.00508033	0.6712	0.871518	5	104620815	0.010278	0.62	5	1.04E+08	0.0015	4.85E-19
chr5:107431645:a:g	A	G	A	G	0.0095	-0.00807687	0.3695	0.6256	5	107431645	0.006914	0.24	5	1.07E+08	0.0015	6.30E-11
chr5:11471767:c:a	A	C	A	C	0.0091	-0.00315695	0.5321	0.24952	5	11471767	0.007601	0.68	5	11471879	0.0014	1.06E-10
chr5:124968984:t:c	T	C	T	C	0.011	0.00206795	0.3276	0.431796	5	124968984	0.006862	0.76	5	1.24E+08	0.0015	1.88E-13
chr5:137221366:t:g	T	G	T	G	-0.0137	0.00110973	0.1479	0.178194	5	137221366	0.008963	0.9	5	1.37E+08	0.002	4.63E-12
chr5:146326106:t:g	T	G	T	G	-0.0091	0.00667777	0.7621	0.787704	5	146326106	0.00835	0.42	5	1.46E+08	0.0017	3.77E-08
chr5:153999327:g:c	A	C	G	C	-0.03	0.0250333	0.0186	0.008165	5	153999327	0.038305	0.51	5	1.53E+08	0.0053	1.17E-08
chr5:166981783:g:c	C	G	C	G	-0.0098	-0.00991339	0.7122	0.42147	5	166981783	0.006904	0.15	5	1.66E+08	0.0016	2.74E-10
chr5:169551144:a:t	A	T	A	T	-0.0093	0.00462457	0.3032	0.067723	5	169551144	0.013276	0.73	5	1.69E+08	0.0015	1.06E-09
chr5:30808538:t:g	T	G	T	G	0.0111	-0.00541115	0.4194	0.633045	5	30808538	0.007161	0.45	5	30808645	0.0014	1.31E-14
chr5:58236948:t:c	T	C	T	C	-0.0099	-0.00703948	0.3149	0.261047	5	58236948	0.007647	0.36	5	57532775	0.0015	4.61E-11

chr5:58475260:a:g	A	G	A	G	-0.0116	-0.0145814	0.2929	0.220461	5	58475260	0.008402	0.083	5	57771087	0.0015	4.62E-14
chr5:60274431:a:g	A	G	A	G	-0.0084	0.00857347	0.4787	0.354947	5	60274431	0.007036	0.22	5	59570258	0.0014	7.55E-09
chr5:72883747:g:c	C	G	C	G	0.0105	0.0135	0.1696	0.106388	5	72883747	0.011112	0.22	5	72179574	0.0019	3.00E-08
chr5:87788144:t:c	T	C	T	C	-0.0129	0.0190154	0.8647	0.988953	5	87788144	0.033042	0.56	5	87083961	0.0021	2.95E-10
chr6:107708314:c:a	A	C	A	C	0.0089	0.0077941	0.7272	0.521374	6	107708314	0.006858	0.26	6	1.08E+08	0.0016	1.32E-08
chr6:118894238:a:c	A	C	A	C	0.0112	0.0129712	0.6947	0.366715	6	118894238	0.006996	0.064	6	1.19E+08	0.0015	2.44E-13
chr6:130981349:g:c	C	G	C	G	0.008	0.00226951	0.3692	0.634006	6	130981349	0.007115	0.75	6	1.31E+08	0.0015	4.33E-08
chr6:144702986:t:c	T	C	T	C	0.0093	0.00400646	0.5472	0.058838	6	144702986	0.01394	0.77	6	1.45E+08	0.0014	5.31E-11
chr6:151913458:t:c	T	C	T	C	-0.0155	-0.007103	0.3232	0.574928	6	151913458	0.006933	0.31	6	1.52E+08	0.0015	5.48E-25
chr6:16965821:a:g	A	G	A	G	-0.0149	0.00702104	0.761	0.833333	6	16965821	0.009488	0.46	6	16966052	0.0016	1.52E-19
chr6:31587353:a:g	A	G	A	G	0.0106	-0.00140902	0.4188	0.237752	6	31587353	0.008003	0.86	6	31555130	0.0014	2.40E-13
chr6:31917046:t:c	T	C	T	C	0.0138	-0.0519279	0.1428	0.012008	6	31917046	0.03105	0.094	6	31845985	0.002	5.25E-12
chr6:32107786:t:c	T	C	T	C	0.0085	0.00129744	0.5292	0.483429	6	32107786	0.006836	0.85	6	32075563	0.0014	1.50E-09
chr6:33807171:c:t	T	C	T	C	0.009	0.0124272	0.6627	0.817723	6	33807171	0.008887	0.16	6	33774948	0.0015	1.11E-09
chr6:78801277:a:c	A	C	A	C	0.0084	-0.00505025	0.6187	0.317963	6	78801277	0.007352	0.49	6	79510994	0.0015	1.05E-08
chr6:96071781:t:c	T	C	T	C	-0.0087	-0.0104034	0.3906	0.290586	6	96071781	0.007659	0.17	6	96519657	0.0014	1.20E-09
chr7:102052537:c:g	C	G	C	G	-0.0097	-0.00640156	0.39	0.069404	7	102052537	0.012811	0.62	7	1.02E+08	0.0014	1.72E-11
chr7:104880081:a:t	A	T	A	T	-0.0081	-0.00589815	0.4257	0.056436	7	104880081	0.013745	0.67	7	1.05E+08	0.0014	2.18E-08
chr7:128736846:a:g	A	G	A	G	-0.0081	0.00532016	0.4185	0.360711	7	128736846	0.007038	0.45	7	1.28E+08	0.0014	1.26E-08
chr7:13988366:t:c	T	C	T	C	0.0082	0.00578107	0.4725	0.431316	7	13988366	0.006861	0.4	7	14027991	0.0014	5.59E-09
chr7:2154761:t:c	T	C	T	C	0.0176	0.018471	0.7812	0.930836	7	2154761	0.012998	0.16	7	2194396	0.0017	2.36E-24
chr7:2283308:t:c	T	C	T	C	0.0109	0.00651185	0.3698	0.35975	7	2283308	0.007031	0.35	7	2322943	0.0015	7.84E-14
chr7:3456400:t:c	T	C	T	C	-0.0121	-0.000822311	0.5462	0.512728	7	3456400	0.006867	0.9	7	3496032	0.0014	9.25E-18
chr7:49830175:a:g	A	G	A	G	0.0141	0.00722134	0.8378	0.888329	7	49830175	0.010821	0.5	7	49869771	0.0019	1.53E-13
chr7:53790128:t:c	T	C	T	C	0.0095	-0.0108115	0.7867	0.806916	7	53790128	0.008741	0.22	7	53857821	0.0017	4.77E-08
chr7:69153783:g:c	C	G	C	G	0.0167	0.00305027	0.0583	0.069885	7	69153783	0.013519	0.82	7	68618770	0.003	2.38E-08
chr7:76216765:c:g	C	G	C	G	-0.0118	0.00625932	0.844	0.71854	7	76216765	0.00762	0.41	7	75846083	0.0019	1.08E-09
chr7:99158974:c:t	T	C	T	C	0.0098	0.000601186	0.7342	0.855427	7	99158974	0.009465	0.95	7	98756597	0.0016	6.44E-10
chr8:119070014:g:c	C	G	C	G	0.0098	0.000994766	0.6682	0.684438	8	119070014	0.007302	0.89	8	1.2E+08	0.0015	1.14E-10
chr8:119174851:g:c	C	G	C	G	0.0122	0.00193692	0.1602	0.084294	8	119174851	0.01223	0.87	8	1.2E+08	0.0019	3.12E-10
chr8:129916920:t:c	T	C	T	C	-0.0104	0.00349287	0.5258	0.502882	8	129916920	0.006897	0.61	8	1.31E+08	0.0014	1.15E-13
chr8:134428365:g:a	A	G	A	G	-0.0158	-0.00350065	0.1142	0.047791	8	134428365	0.015513	0.82	8	1.35E+08	0.0022	9.06E-13
chr8:141607161:c:g	C	G	C	G	0.0135	-0.00432086	0.6283	0.836695	8	141607161	0.00924	0.64	8	1.43E+08	0.0015	1.19E-20
chr8:142599411:c:t	T	C	T	C	-0.0133	-2.70E-05	0.1299	0.486551	8	142599411	0.006854	1	8	1.44E+08	0.0021	2.24E-10
chr8:142683195:g:t	T	G	T	G	0.0081	0.00333653	0.4317	0.176273	8	142683195	0.008892	0.71	8	1.44E+08	0.0014	1.30E-08
chr8:19514256:t:c	T	C	T	C	-0.0088	0.00742076	0.3526	0.186359	8	19514256	0.008691	0.39	8	19371767	0.0015	2.35E-09
chr8:26421657:c:a	A	C	A	C	0.0106	0.00438551	0.1683	0.336215	8	26421657	0.007253	0.55	8	26279173	0.0019	1.97E-08
chr8:42505713:t:c	T	C	T	C	0.0132	0.0159155	0.0987	0.034102	8	42505713	0.01897	0.4	8	42363231	0.0024	4.00E-08
chr8:4938572:g:a	A	G	A	G	-0.0094	-0.00890085	0.3363	0.371998	8	4938572	0.007171	0.21	8	4796094	0.0015	2.03E-10
chr8:65562495:t:c	T	C	T	C	-0.0091	-0.00429097	0.4033	0.970701	8	65562495	0.018167	0.81	8	66474730	0.0014	2.00E-10
chr8:70255759:c:t	T	C	T	C	-0.0124	0.00350048	0.8739	0.602546	8	70255759	0.006847	0.61	8	71167994	0.0021	4.91E-09
chr8:86288546:t:c	T	C	T	C	-0.0092	0.0211097	0.4369	0.647935	8	86288546	0.007346	0.0041	8	87300775	0.0014	8.63E-11
chr8:86757733:a:g	A	G	A	G	0.0109	-0.0122341	0.5093	0.450288	8	86757733	0.006895	0.076	8	87769961	0.0014	2.24E-14
chr8:92223447:g:a	A	G	A	G	0.0083	-0.00544521	0.327	0.487272	8	92223447	0.00689	0.43	8	93235675	0.0015	2.74E-08
chr8:94577112:a:g	A	G	A	G	-0.0086	0.00425769	0.6741	0.528578	8	94577112	0.006901	0.54	8	95589340	0.0015	1.62E-08
chr8:9483422:g:a	A	G	A	G	0.0102	-0.012504	0.4055	0.723583	8	9483422	0.007595	0.1	8	9340932	0.0014	1.11E-12
chr9:106915136:t:c	T	C	T	C	0.0106	-0.00824653	0.7585	0.670509	9	106915136	0.007183	0.25	9	1.1E+08	0.0016	1.47E-10
chr9:116723058:c:a	A	C	A	C	0.0084	0.00779935	0.3041	0.507205	9	116723058	0.006873	0.26	9	1.19E+08	0.0015	3.55E-08
chr9:119218308:g:a	A	G	A	G	0.0115	-0.00138845	0.4604	0.663064	9	119218308	0.007289	0.85	9	1.22E+08	0.0014	2.58E-16
chr9:123566991:g:a	A	G	A	G	-0.0128	0.00095129	0.2441	0.10999	9	123566991	0.010971	0.93	9	1.26E+08	0.0017	1.11E-14
chr9:125650397:g:a	A	G	A	G	0.0084	-0.00968959	0.558	0.471662	9	125650397	0.006993	0.17	9	1.28E+08	0.0014	2.52E-09
chr9:131862364:g:a	A	G	A	G	0.0096	-0.0144151	0.656	0.274496	9	131862364	0.007698	0.061	9	1.35E+08	0.0015	7.42E-11
chr9:132008032:c:a	A	C	A	C	0.0145	-0.00887736	0.684	0.57853	9	132008032	0.0069	0.2	9	1.35E+08	0.0015	7.63E-22
chr9:132615104:t:c	T	C	T	C	-0.0119	-0.00297854	0.7754	0.564601	9	132615104	0.006875	0.66	9	1.35E+08	0.0017	1.26E-12
chr9:14430694:g:a	A	G	A	G	0.0078	0.00906087	0.4144	0.409702	9	14430694	0.00698	0.19	9	14430692	0.0014	4.00E-08

chr9:1757274:t:g	T	G	T	G	-0.0167	-0.00925332	0.6578	0.561479	9	1757274	0.006907	0.18	9	1757274	0.0015	1.25E-29
chr9:23747793:t:c	T	C	T	C	0.0134	-0.00803767	0.8276	0.764169	9	23747793	0.008167	0.33	9	23747791	0.0019	6.28E-13
chr9:3952892:g:a	A	G	A	G	-0.0093	-0.00774155	0.3411	0.329971	9	3952892	0.007282	0.29	9	3952892	0.0015	4.12E-10
chr9:79827816:a:t	A	T	A	T	-0.0101	0.00155907	0.4417	0.314121	9	79827816	0.007199	0.83	9	82442731	0.0014	7.78E-13
chr9:89611257:c:g	C	G	C	G	0.0097	0.00368883	0.5324	0.739673	9	89611257	0.00792	0.64	9	92226172	0.0014	5.39E-12
chr9:93604365:a:g	A	G	A	G	-0.0092	-0.00719488	0.244	0.256484	9	93604365	0.00774	0.35	9	96366647	0.0017	2.89E-08
rs10006235	T	C	T	C	-0.0093	-0.0214552	0.2689	0.42171	4	129748953	0.006943	0.002	4	1.31E+08	0.0016	4.48E-09
rs10041403	T	C	T	C	0.0151	-0.0293558	0.8339	0.979347	5	68509445	0.022534	0.19	5	67805272	0.0019	1.55E-15
rs10042828	A	G	A	G	-0.0145	0.0204006	0.8885	0.986551	5	89012960	0.028473	0.47	5	88308777	0.0022	1.06E-10
rs10046069	A	G	A	G	0.0127	0.0215633	0.1203	0.14073	5	89819759	0.009791	0.028	5	89115576	0.0022	4.40E-09
rs10057590	A	C	A	C	0.0106	-0.00170624	0.4724	0.286023	5	124999554	0.007567	0.82	5	1.24E+08	0.0014	4.08E-14
rs10061420	A	T	A	T	0.0082	0.000597952	0.4741	0.665946	5	105342378	0.007201	0.93	5	1.05E+08	0.0014	6.63E-09
rs1006749	A	G	A	G	0.0079	-0.00143501	0.5237	0.732229	20	43088013	0.007669	0.85	20	41716653	0.0014	2.43E-08
rs10074178	A	G	A	G	0.0079	-0.00121923	0.5129	0.643852	5	94592000	0.007079	0.86	5	93927705	0.0014	1.58E-08
rs1007731	A	C	A	C	-0.0143	-0.0259314	0.1159	0.012968	14	36585385	0.030511	0.4	14	37054590	0.0022	1.05E-10
rs10080647	A	C	A	C	0.0124	0.00342207	0.1405	0.166667	6	114421171	0.009218	0.71	6	1.15E+08	0.002	7.05E-10
rs1008078	T	C	T	C	-0.0182	0.00189249	0.4019	0.388809	1	90724174	0.00712	0.79	1	91189731	0.0014	4.50E-37
rs1009470	T	G	T	G	0.0086	0.0158702	0.4414	0.601105	9	119526909	0.006853	0.021	9	1.22E+08	0.0014	1.34E-09
rs10098073	A	C	A	C	-0.0091	0.00927303	0.4775	0.289145	8	142228143	0.00767	0.23	8	1.43E+08	0.0014	1.03E-10
rs10122669	A	G	A	G	-0.0079	0.0161683	0.4143	0.303554	9	113793491	0.007403	0.029	9	1.17E+08	0.0014	3.00E-08
rs10128888	A	G	A	G	-0.0083	0.00165459	0.3149	0.393132	12	97405746	0.006859	0.81	12	97799524	0.0015	4.04E-08
rs10166286	T	C	T	C	0.0151	0.000368067	0.173	0.539385	2	100511633	0.006733	0.96	2	1.01E+08	0.0019	3.79E-16
rs10169002	A	G	A	G	-0.008	0.0123973	0.5305	0.298751	2	139805799	0.007552	0.1	2	1.41E+08	0.0014	1.15E-08
rs10189857	A	G	A	G	0.0158	0.00258422	0.5654	0.231028	2	60486100	0.007993	0.75	2	60713235	0.0014	4.69E-29
rs10192369	A	G	A	G	-0.0078	0.00956316	0.496	0.68756	2	160524377	0.007249	0.19	2	1.61E+08	0.0014	2.36E-08
rs10192834	T	C	T	C	0.0083	-0.00403967	0.655	0.652978	2	206860892	0.007222	0.58	2	2.08E+08	0.0015	2.05E-08
rs10193498	A	T	A	T	0.0097	0.0057421	0.755	0.425552	2	173229617	0.00669	0.39	2	1.74E+08	0.0016	3.10E-09
rs10204051	T	G	T	G	-0.0101	0.00019943	0.4055	0.360711	2	103158977	0.007226	0.98	2	1.04E+08	0.0014	1.92E-12
rs10205057	T	C	T	C	0.009	-0.00582705	0.5619	0.527378	2	185275099	0.006858	0.4	2	1.86E+08	0.0014	3.67E-10
rs10208	T	C	T	C	0.0091	0.00459272	0.3173	0.689481	17	44222910	0.007169	0.52	17	42300278	0.0015	2.07E-09
rs10215082	A	G	A	G	-0.0149	-0.0278848	0.4661	0.074448	7	93028671	0.012547	0.026	7	92657985	0.0014	7.03E-26
rs1024268	T	C	T	C	-0.0084	-0.00267116	0.4186	0.458453	1	69651721	0.006807	0.69	1	70117404	0.0014	3.40E-09
rs10251438	A	C	A	C	0.0078	-0.00141462	0.4955	0.290586	7	137322222	0.007444	0.85	7	1.37E+08	0.0014	2.65E-08
rs1035578	A	G	A	G	-0.0094	-0.0130713	0.5272	0.647935	16	12437508	0.007163	0.068	16	12531365	0.0014	2.57E-11
rs10402747	T	C	T	C	0.0077	-0.0111131	0.5175	0.832373	19	45311990	0.008575	0.19	19	45815248	0.0014	4.79E-08
rs10411759	A	G	A	G	0.0116	0.0111928	0.1561	0.288425	19	1857298	0.007633	0.14	19	1857297	0.002	2.88E-09
rs10434747	A	G	A	G	-0.008	-0.00271312	0.4248	0.384966	5	137072341	0.007243	0.71	5	1.36E+08	0.0014	1.48E-08
rs10444280	T	C	T	C	0.01	-0.00185365	0.7982	0.619356	11	41134347	0.006875	0.79	11	41155897	0.0018	1.47E-08
rs10450809	A	G	T	A	-0.0116	-0.0113058	0.8437	0.016331	13	98413817	0.025416	0.66	13	99055774	0.0019	2.18E-09
rs10480450	A	G	A	G	0.0158	-0.0198686	0.9341	0.878722	7	108643162	0.010733	0.064	7	1.08E+08	0.0028	2.05E-08
rs10496091	A	G	A	G	-0.0131	0.00197056	0.2841	0.021854	2	61255126	0.0232	0.93	2	61482261	0.0016	3.71E-17
rs10499535	A	G	A	G	0.008	-0.0037777	0.5013	0.764409	7	21650988	0.00816	0.64	7	21690606	0.0014	1.40E-08
rs10509251	T	C	T	C	-0.0093	0.0124597	0.22	0.404179	10	66068678	0.006988	0.075	10	67828436	0.0017	3.61E-08
rs10515007	T	C	T	C	0.0143	0.0083744	0.161	0.278338	17	52318179	0.007485	0.26	17	50395539	0.0019	8.07E-14
rs1051860	A	G	A	G	-0.0079	-0.00197345	0.4071	0.510807	14	58371950	0.006766	0.77	14	58838668	0.0014	3.29E-08
rs10519504	T	G	T	G	0.0119	0.0149572	0.1561	0.160183	4	139719129	0.009168	0.1	4	1.41E+08	0.0019	7.31E-10
rs1054442	A	C	A	C	-0.0136	0.000476362	0.6223	0.538425	12	48995537	0.006805	0.94	12	49389320	0.0015	1.10E-20
rs1058993	A	G	A	T	-0.0248	0.0170664	0.965	0.018972	19	13023687	0.025407	0.5	19	13109531	0.0038	8.47E-11
rs1061801	A	G	A	G	-0.011	-0.00474662	0.187	0.123919	6	33314561	0.010469	0.65	6	33282338	0.0018	1.06E-09
rs1066769	A	G	A	G	-0.023	-0.00682754	0.0311	0.038184	2	188715421	0.017936	0.7	2	1.9E+08	0.0041	1.38E-08
rs10742591	A	T	A	T	-0.0084	-0.0118123	0.6097	0.364313	11	41527406	0.007043	0.093	11	41548956	0.0014	4.26E-09
rs10750539	A	G	A	G	-0.0095	-0.00547465	0.3517	0.334534	11	133678978	0.007333	0.46	11	1.34E+08	0.0015	1.11E-10
rs10752262	T	C	T	C	0.0102	-0.00712261	0.4192	0.587656	10	12353101	0.006969	0.31	10	12395100	0.0014	1.28E-12
rs10761202	T	C	T	C	0.0088	-0.0098047	0.4946	0.413305	9	93257688	0.006883	0.15	9	96019970	0.0014	4.28E-10
rs10761251	A	T	A	T	0.0113	-0.00787448	0.6633	0.451969	9	93659264	0.006842	0.25	9	96421546	0.0015	2.52E-14

rs10773002	A	T	A	T	0.0185	-0.0130468	0.252	0.255524	12	123262414	0.00804	0.1	12	1.24E+08	0.0016	2.50E-30
rs10773208	T	C	T	C	-0.011	0.0162024	0.2526	0.15634	12	122485609	0.00933	0.082	12	1.23E+08	0.0016	8.09E-12
rs10782651	T	C	T	C	-0.0082	-0.00664422	0.5516	0.574207	1	77468650	0.006908	0.34	1	77934335	0.0014	6.37E-09
rs10783243	A	G	A	G	-0.0092	0.000521248	0.5228	0.386407	12	48259220	0.007022	0.94	12	48653003	0.0014	5.80E-11
rs10789285	T	G	T	G	0.0091	0.00097083	0.7542	0.674352	1	69322799	0.007448	0.9	1	69788482	0.0016	2.07E-08
rs10793903	A	G	A	G	0.0126	0.00214132	0.1146	0.317003	9	132101265	0.007218	0.77	9	1.35E+08	0.0022	1.37E-08
rs10795831	T	G	T	G	-0.0099	-0.00145984	0.212	0.621758	10	10901416	0.007021	0.84	10	10943379	0.0017	6.98E-09
rs10798888	T	G	T	G	-0.014	0.00259946	0.1725	0.242555	1	31733498	0.007808	0.74	1	32199099	0.0019	5.15E-14
rs10803585	A	G	A	G	-0.0084	0.00637838	0.3555	0.577089	2	127372532	0.006711	0.34	2	1.28E+08	0.0015	1.05E-08
rs10805383	A	G	A	G	-0.0103	0.00459279	0.4879	0.815562	5	63738779	0.008911	0.61	5	63034606	0.0014	2.22E-13
rs10810099	A	G	A	G	-0.0146	0.00100316	0.2734	0.410903	9	14161928	0.007028	0.89	9	14161927	0.0016	1.91E-20
rs10830858	T	C	T	C	0.0092	-0.00496147	0.5104	0.747358	11	92163472	0.007757	0.52	11	91896638	0.0014	5.31E-11
rs10831656	T	C	T	C	0.009	0.00970944	0.3134	0.335255	11	11639268	0.007343	0.19	11	11660815	0.0015	3.10E-09
rs10845051	A	T	A	T	0.0083	0.012838	0.5055	0.775216	12	10138847	0.008231	0.12	12	10291446	0.0014	3.75E-09
rs10862376	A	G	T	A	0.0187	0.0112818	0.0995	0.212776	12	81863854	0.00833	0.18	12	82258775	0.0023	1.67E-15
rs10875121	C	G	C	G	0.0174	0.00430049	0.8361	0.931076	1	97951890	0.013531	0.75	1	98417446	0.0019	4.46E-20
rs10877283	T	G	T	G	0.0085	0.00367609	0.5973	0.419789	12	59443953	0.006648	0.58	12	59837734	0.0014	2.86E-09
rs10879676	T	C	T	C	-0.0083	-0.00830261	0.5833	0.790106	12	73758622	0.008431	0.32	12	74152402	0.0014	5.29E-09
rs10892807	T	C	T	C	0.0109	-0.00790457	0.5671	0.56244	11	122127546	0.006848	0.25	11	1.22E+08	0.0014	1.09E-14
rs10896334	C	G	T	C	-0.0128	-0.0105125	0.2609	0.20317	11	68514026	0.00855	0.22	11	68282986	0.0021	1.15E-09
rs10899282	A	G	A	G	0.014	0.00683425	0.2279	0.18684	11	76793654	0.008921	0.44	11	76504698	0.0017	1.05E-16
rs10927053	A	T	A	T	-0.0125	-0.00403236	0.8886	0.76489	1	243648019	0.007993	0.61	1	2.44E+08	0.0022	2.13E-08
rs10928190	T	C	T	C	-0.0087	-0.00350388	0.452	0.741835	2	143618464	0.007761	0.65	2	1.44E+08	0.0014	7.76E-10
rs10931821	A	T	A	T	-0.0141	0.00406818	0.4852	0.708694	2	198631106	0.007264	0.58	2	1.99E+08	0.0014	8.65E-24
rs10937240	T	C	T	C	-0.0123	0.00959173	0.1899	0.586455	3	186085034	0.006737	0.15	3	1.86E+08	0.0018	6.95E-12
rs10940540	A	T	A	T	0.0078	0.00236523	0.5522	0.669549	5	57381819	0.007186	0.74	5	56677646	0.0014	3.44E-08
rs10949263	A	G	A	G	-0.0091	-0.00738596	0.2443	0.350144	6	14886746	0.007138	0.3	6	14886977	0.0016	2.33E-08
rs10951590	T	C	T	C	-0.0109	-0.00318019	0.3275	0.680836	7	39051443	0.00721	0.66	7	39091043	0.0015	3.62E-13
rs1097784	T	C	T	C	-0.0083	-0.000343145	0.5021	0.15682	16	10155559	0.009059	0.97	16	10249416	0.0014	3.05E-09
rs10979613	T	C	T	C	-0.0117	-0.00638733	0.6389	0.603506	9	108925304	0.006983	0.36	9	1.12E+08	0.0015	1.25E-15
rs10985402	T	G	T	G	0.0089	0.00375965	0.2472	0.399616	9	121816503	0.006987	0.59	9	1.25E+08	0.0016	4.47E-08
rs10995639	T	C	T	C	0.0078	-0.0100695	0.4199	0.576849	10	63813435	0.006967	0.15	10	65573195	0.0014	4.26E-08
rs11003463	T	G	T	G	0.0091	-0.0186527	0.6114	0.949087	10	50042033	0.015065	0.22	10	51801793	0.0015	5.58E-10
rs11021432	A	T	A	T	0.0103	0.00422299	0.3692	0.229587	11	96104510	0.00796	0.6	11	95837674	0.0015	1.25E-12
rs11023764	A	G	A	G	0.0091	0.00910352	0.6418	0.247598	11	15889393	0.007853	0.25	11	15910939	0.0015	5.32E-10
rs11030102	C	G	C	G	0.0101	0.00360197	0.7479	0.981989	11	27660049	0.024396	0.88	11	27681596	0.0016	4.60E-10
rs1106090	A	G	A	G	0.0094	0.00116858	0.6228	0.48439	2	57841606	0.006827	0.86	2	58068741	0.0014	1.00E-10
rs11076962	T	C	T	C	0.0086	-0.00264993	0.7189	0.449808	16	5761366	0.006827	0.7	16	5811367	0.0016	4.29E-08
rs1107871	A	G	A	G	-0.0083	-0.0123823	0.5506	0.482229	12	131756114	0.006892	0.072	12	1.32E+08	0.0014	4.18E-09
rs11081529	T	C	T	C	0.0126	-0.0110182	0.7112	0.641451	18	78142735	0.007081	0.12	18	75902735	0.0015	5.22E-16
rs11082011	T	C	T	C	0.0194	0.00510864	0.6716	0.65682	18	37565159	0.007402	0.49	18	35145122	0.0015	2.06E-38
rs11100237	A	G	A	G	-0.0101	-0.00439307	0.5242	0.338377	4	159555889	0.007185	0.54	4	1.6E+08	0.0014	8.17E-13
rs11121177	A	G	A	G	0.0151	0.0137323	0.1785	0.079491	1	8385886	0.012042	0.25	1	8445946	0.0018	1.44E-16
rs111235962	T	C	T	C	0.0133	-0.0112102	0.0962	0.052354	1	20545257	0.015447	0.47	1	20871750	0.0024	2.21E-08
rs11130335	T	C	T	C	0.0095	-0.0153304	0.3778	0.108069	3	52989619	0.010897	0.16	3	53028375	0.0015	1.05E-10
rs11130380	T	G	T	G	0.009	0.00037023	0.6135	0.877281	3	53724923	0.01033	0.97	3	53758950	0.0014	4.17E-10
rs11138947	T	C	T	C	0.0105	-0.00275243	0.7236	0.537944	9	69495646	0.0069	0.69	9	72110562	0.0016	2.36E-11
rs11154410	T	C	T	C	-0.0092	-0.0247829	0.3311	0.01609	6	127457700	0.024653	0.31	6	1.28E+08	0.0015	7.71E-10
rs11155813	T	C	T	C	-0.0129	0.000944405	0.8937	0.716138	6	151828300	0.007683	0.9	6	1.52E+08	0.0023	1.57E-08
rs11157931	A	C	A	C	-0.0139	0.0116663	0.3924	0.200048	14	22933984	0.008538	0.17	14	23403193	0.0014	3.86E-22
rs11158800	A	G	A	G	-0.0091	0.0145052	0.5044	0.611912	14	69280329	0.006843	0.034	14	69747046	0.0014	7.15E-11
rs1117152	T	G	T	G	0.0106	-0.0149691	0.701	0.73391	12	15285098	0.007748	0.053	12	15438032	0.0015	4.47E-12
rs11174399	A	G	A	G	0.0111	-0.00187438	0.2008	0.158501	12	62252775	0.009458	0.84	12	62646556	0.0018	2.42E-10
rs111821073	T	C	T	C	0.0124	-0.0146288	0.1567	0.019933	9	96322511	0.023925	0.54	9	99084793	0.0019	1.58E-10
rs11190955	T	C	T	C	-0.0083	-0.0037935	0.354	0.122478	10	101352408	0.010398	0.72	10	1.03E+08	0.0015	1.74E-08

rs1120924	T	C	T	C	0.0108	0.00662523	0.3362	0.305956	4	5227212	0.007417	0.37	4	5228939	0.0015	2.79E-13
rs11209894	A	G	A	G	0.0119	-0.00327147	0.1377	0.122959	1	41332996	0.010429	0.75	1	41798668	0.0021	8.03E-09
rs11210228	T	G	T	G	-0.0105	-0.00172981	0.4356	0.245197	1	73393607	0.007815	0.82	1	73859290	0.0019	2.20E-08
rs11210400	A	C	A	C	0.012	0.019425	0.538	0.794669	1	74024775	0.008351	0.02	1	74490459	0.0014	1.63E-17
rs11213482	A	G	A	G	-0.0106	-0.000761358	0.836	0.959894	11	110564912	0.016999	0.96	11	1.1E+08	0.0019	2.68E-08
rs1123285	C	G	C	G	-0.0094	0.00413632	0.6659	0.682517	14	56807801	0.007325	0.57	14	57274519	0.0015	2.38E-10
rs112375785	A	G	A	G	0.0184	0.017285	0.1435	0.012488	6	97776341	0.030482	0.57	6	98224217	0.0021	6.00E-19
rs112603734	A	C	A	C	-0.0131	-0.00869119	0.7699	0.986792	6	6069355	0.028753	0.76	6	6069588	0.0022	2.72E-09
rs1128956	T	G	T	G	-0.014	-0.00755274	0.8251	0.755764	4	182802852	0.007994	0.34	4	1.84E+08	0.0019	5.34E-14
rs113205706	A	G	A	G	-0.0135	-0.0278271	0.0944	0.007685	7	39207388	0.039485	0.48	7	39246987	0.0024	2.00E-08
rs113520408	A	G	A	G	0.0155	-0.00200781	0.2756	0.065802	7	128762728	0.013697	0.88	7	1.28E+08	0.0016	1.03E-22
rs113588399	T	C	T	C	-0.0104	0.00859494	0.2079	0.077089	6	156915292	0.012618	0.5	6	1.57E+08	0.0017	1.67E-09
rs114142672	A	C	A	C	0.0149	0.026678	0.9112	0.988953	3	165836740	0.033021	0.42	3	1.66E+08	0.0025	1.39E-09
rs114593137	A	T	A	T	-0.0122	0.00816626	0.793	0.916427	1	241713263	0.012492	0.51	1	2.42E+08	0.0017	1.65E-12
rs114810763	A	G	A	G	-0.0121	-0.00623835	0.1549	0.035543	6	30430613	0.018223	0.73	6	30398390	0.002	1.98E-09
rs11492857	T	G	T	C	0.0122	-0.0402495	0.1952	0.018012	10	85277976	0.026064	0.12	10	87039251	0.0018	5.08E-12
rs11591870	T	C	T	C	0.0098	0.0371781	0.798	0.989914	10	63734276	0.033721	0.27	10	65494036	0.0017	2.25E-08
rs11592299	A	G	A	G	0.0097	0.00430548	0.1958	0.056196	10	102466184	0.015002	0.77	10	1.04E+08	0.0018	4.19E-08
rs11598765	A	G	A	G	-0.0106	0.00420863	0.1966	0.185159	10	23664796	0.008738	0.63	10	23953725	0.0018	1.73E-09
rs11623285	T	G	T	G	-0.0135	-0.0299719	0.863	0.95365	14	24088433	0.016094	0.063	14	24557642	0.0021	5.58E-11
rs11640569	A	G	A	G	0.0112	0.00702982	0.142	0.019933	16	48757481	0.023928	0.77	16	48791392	0.002	2.77E-08
rs11644446	A	G	A	G	0.0117	-0.00596853	0.1562	0.016571	16	7893550	0.027122	0.83	16	7943552	0.0019	1.61E-09
rs11647188	A	G	A	G	0.0079	0.000654192	0.6066	0.285543	16	82614909	0.007491	0.93	16	82648514	0.0014	4.92E-08
rs11652522	A	C	A	C	-0.0154	0.00179597	0.1045	0.079731	17	44978211	0.012671	0.89	17	43055579	0.0023	2.29E-11
rs11657342	A	G	A	G	0.014	0.00448038	0.367	0.036023	17	81381494	0.01701	0.79	17	79355294	0.0019	2.32E-13
rs11657979	A	G	A	G	-0.0091	-0.00515032	0.2449	0.162344	17	34573818	0.009232	0.58	17	32900837	0.0017	4.57E-08
rs11662423	A	G	C	A	0.0088	0.00162155	0.3998	0.434198	18	76469034	0.006784	0.81	18	74141190	0.0014	8.95E-10
rs11663678	T	C	T	C	-0.0121	0.0103332	0.8842	0.924352	18	45328761	0.012582	0.41	18	42908726	0.0022	3.42E-08
rs1167827	A	G	A	G	0.0104	-0.00290299	0.4347	0.875841	7	75533848	0.009986	0.77	7	75163169	0.0014	2.93E-13
rs11678980	A	G	A	G	-0.0166	0.00914988	0.4527	0.376801	2	161244750	0.007165	0.2	2	1.62E+08	0.0014	1.60E-31
rs11687736	A	T	A	T	-0.0105	-0.00620536	0.6764	0.692123	2	224540405	0.007456	0.41	2	2.25E+08	0.0015	4.66E-12
rs11693885	A	G	A	G	-0.0091	-0.0121096	0.4522	0.304515	2	59894911	0.00747	0.11	2	60122046	0.0014	1.22E-10
rs117005905	T	C	T	C	0.015	0.000869463	0.1143	0.148415	6	113897444	0.00946	0.93	6	1.14E+08	0.0022	9.35E-12
rs11703948	A	G	A	G	-0.0168	0.000950066	0.9026	0.851825	22	38421042	0.009669	0.92	22	38817047	0.0024	1.26E-12
rs1171040	A	G	A	G	-0.0113	-0.0023906	0.7866	0.673151	1	190431854	0.007297	0.74	1	1.9E+08	0.0017	5.07E-11
rs11716398	A	G	A	G	0.0159	0.0150972	0.8663	0.62488	3	50662284	0.006888	0.028	3	50699715	0.0021	1.89E-14
rs11720093	T	C	T	C	0.015	0.0123379	0.0809	0.250961	3	50524611	0.007874	0.12	3	50562042	0.0026	6.48E-09
rs11724690	T	G	T	G	0.0093	-0.0194123	0.2887	0.080692	4	185843593	0.012466	0.12	4	1.87E+08	0.0015	2.08E-09
rs11732160	A	G	A	G	0.0105	-0.00415427	0.7154	0.819164	4	66205106	0.008907	0.64	4	67070824	0.0016	1.46E-11
rs11733439	A	G	A	G	-0.0096	0.000637248	0.7883	0.607349	4	30533070	0.007284	0.93	4	30534692	0.0017	2.10E-08
rs11736863	A	G	A	G	0.0142	0.00155166	0.1895	0.043708	4	23727943	0.016522	0.93	4	23729566	0.0018	2.56E-15
rs117398064	C	G	C	G	-0.0141	0.0220937	0.0912	0.007205	12	26370695	0.039414	0.58	12	26523628	0.0025	1.16E-08
rs117623407	A	G	A	G	-0.0118	0.0328136	0.8536	0.974544	19	31713583	0.021653	0.13	19	32204489	0.002	4.30E-09
rs11765387	T	C	T	C	0.0086	0.000490128	0.6734	0.418348	7	32455178	0.006981	0.94	7	32494790	0.0015	9.40E-09
rs11772232	T	C	T	C	0.0177	-0.00394546	0.1691	0.115274	7	1816637	0.010733	0.71	7	1856273	0.0019	1.20E-20
rs11774212	T	C	T	C	0.0128	-0.00571543	0.5163	0.270173	8	144461122	0.007685	0.46	8	1.46E+08	0.0014	1.40E-19
rs117799466	C	G	C	G	0.0106	0.00636548	0.3619	0.282901	15	34367316	0.007601	0.4	15	34659517	0.0016	1.19E-11
rs11780023	T	C	T	C	0.0085	0.00156312	0.5051	0.585014	8	140982679	0.006965	0.82	8	1.42E+08	0.0014	1.17E-09
rs118093058	T	G	T	G	0.0146	-0.00166048	0.133	0.222382	11	62646201	0.008165	0.84	11	62413673	0.0021	3.78E-12
rs11820113	T	C	T	C	-0.0104	-0.00986516	0.2459	0.271134	11	107296760	0.007788	0.21	11	1.07E+08	0.0016	1.61E-10
rs11845781	T	C	T	C	-0.0079	0.0158635	0.5552	0.601105	14	88810087	0.00704	0.024	14	89276431	0.0014	2.43E-08
rs11871429	A	G	A	G	0.0128	-0.00223253	0.7727	0.666186	17	44843561	0.00724	0.76	17	42920929	0.0017	2.52E-14
rs11917701	A	G	A	G	-0.0087	-0.00244314	0.4041	0.600624	3	117455721	0.006891	0.72	3	1.17E+08	0.0014	9.45E-10
rs11919835	C	G	C	G	0.0089	0.00569764	0.3857	0.367915	3	70905122	0.007066	0.42	3	70954273	0.0015	7.95E-10
rs12005151	A	G	A	G	0.0087	0.0171903	0.3615	0.139769	9	132155262	0.010081	0.088	9	1.35E+08	0.0015	3.73E-09

rs12028010	T	C	T	C	0.0164	-0.0128219	0.7711	0.660183	1	41298799	0.007079	0.07	1	41764471	0.0017	9.78E-23
rs12028229	T	G	T	G	0.0099	0.00344979	0.2631	0.431076	1	72520453	0.006797	0.61	1	72986136	0.0016	1.67E-09
rs12054166	C	G	C	G	0.0096	-0.00688524	0.7399	0.935159	3	150375658	0.013812	0.62	3	1.5E+08	0.0016	2.30E-09
rs12076635	C	G	C	G	0.0207	-0.00508589	0.7811	0.771614	1	43560985	0.007923	0.52	1	44026656	0.0017	3.33E-34
rs12113634	T	C	T	C	0.0088	-0.00164023	0.6504	0.669549	7	112356897	0.007361	0.82	7	1.12E+08	0.0015	2.50E-09
rs12123293	T	C	T	C	0.0086	0.011715	0.6476	0.800913	1	210739493	0.008633	0.17	1	2.11E+08	0.0015	4.48E-09
rs12126231	A	G	A	G	0.0095	0.0043271	0.6094	0.451729	1	184729682	0.006988	0.54	1	1.85E+08	0.0014	4.82E-11
rs12127928	T	C	T	C	0.0108	0.0157604	0.8057	0.912104	1	28382018	0.012193	0.2	1	28708529	0.0018	1.69E-09
rs12145078	A	T	A	T	-0.0127	-0.00162139	0.2074	0.115514	1	197853847	0.01048	0.88	1	1.98E+08	0.0017	2.56E-13
rs12170452	A	G	A	G	0.0105	9.97E-05	0.4507	0.053314	22	39623768	0.014005	0.99	22	40019773	0.0014	7.78E-14
rs1220779	A	G	A	G	-0.0079	-0.0108369	0.4679	0.158261	7	54666980	0.009128	0.24	7	54734673	0.0014	1.58E-08
rs12234369	A	G	A	G	0.0087	-0.0035903	0.3142	0.126561	7	11853649	0.010018	0.72	7	11893275	0.0015	9.61E-09
rs12238011	T	C	T	C	-0.0141	0.00899519	0.0809	0.137368	9	14210898	0.009932	0.37	9	14210897	0.0026	4.67E-08
rs12273435	A	G	A	G	-0.0132	-0.00096701	0.207	0.064601	11	133955960	0.013463	0.94	11	1.34E+08	0.0017	4.72E-14
rs12285074	A	G	A	G	0.0161	0.00655487	0.1012	0.51513	11	116891312	0.006962	0.35	11	1.17E+08	0.0023	5.11E-12
rs12304188	A	G	A	G	0.0111	0.00565052	0.842	0.653698	12	97587494	0.007292	0.44	12	97981272	0.0019	8.17E-09
rs12332731	A	T	A	T	0.0135	0.00608461	0.1882	0.054755	5	93697376	0.014827	0.68	5	93033082	0.0018	5.96E-14
rs12359372	T	C	T	C	-0.0092	0.0256608	0.6624	0.837656	10	66008193	0.009145	0.005	10	67767951	0.0015	6.33E-10
rs12375949	T	C	T	C	-0.0138	-0.000591436	0.4312	0.283862	9	121855621	0.007619	0.94	9	1.25E+08	0.0014	1.61E-22
rs12431682	T	C	T	C	-0.0093	0.00492871	0.3699	0.438761	14	101073662	0.006994	0.48	14	1.02E+08	0.0015	1.73E-10
rs12438177	A	G	A	G	0.0093	0.000228952	0.3676	0.570845	15	55659234	0.006694	0.97	15	55951432	0.0015	1.48E-10
rs1245829	A	T	A	T	-0.0099	0.00087089	0.5814	0.867915	12	79254390	0.010075	0.93	12	79648170	0.0014	3.29E-12
rs12467175	T	C	T	C	-0.0081	0.00333619	0.4923	0.31244	2	228325144	0.007329	0.65	2	2.29E+08	0.0014	6.90E-09
rs12468040	T	G	T	G	0.0137	-0.00349836	0.3819	0.694524	2	44627842	0.007334	0.63	2	44854981	0.0014	3.12E-21
rs12473986	T	C	T	C	-0.0085	0.00876129	0.2907	0.409942	2	172818220	0.006761	0.2	2	1.74E+08	0.0015	3.57E-08
rs12477385	T	G	T	G	0.0109	-0.00155702	0.2264	0.356868	2	165288340	0.007004	0.82	2	1.66E+08	0.0017	7.15E-11
rs12478156	T	C	T	C	0.0108	0.00318178	0.6378	0.610471	2	163523148	0.007019	0.65	2	1.64E+08	0.0015	1.24E-13
rs12503522	T	C	T	C	-0.0113	-0.000417903	0.282	0.534822	4	93622082	0.006777	0.95	4	94543233	0.0016	3.93E-13
rs12506221	T	G	T	G	-0.013	0.00211391	0.436	0.441883	4	67033517	0.006782	0.76	4	67899235	0.0014	4.23E-20
rs12516990	C	G	C	G	-0.0099	-0.0437458	0.2106	0.010087	5	146548125	0.034517	0.21	5	1.46E+08	0.0017	7.90E-09
rs12519073	T	C	T	C	-0.0107	0.000116365	0.2317	0.079251	5	137441073	0.012699	0.99	5	1.37E+08	0.0017	1.16E-10
rs12524795	T	C	T	C	0.0102	0.00644377	0.4374	0.516811	6	3463840	0.006885	0.35	6	3464074	0.0014	5.28E-13
rs12571549	A	G	A	G	0.0151	-0.00380145	0.1368	0.419308	10	110010255	0.006871	0.58	10	1.12E+08	0.002	1.45E-13
rs12574281	A	C	A	C	-0.008	-0.00129784	0.631	0.739914	11	131335526	0.007732	0.87	11	1.31E+08	0.0015	3.04E-08
rs12591647	T	C	T	C	-0.0152	0.000920812	0.8171	0.28098	15	65667391	0.007417	0.9	15	65959729	0.0018	7.78E-17
rs12601380	A	C	A	C	-0.0082	-0.00285064	0.5739	0.604947	17	36549144	0.007006	0.68	17	34904985	0.0014	7.42E-09
rs12602286	T	G	T	G	0.016	-0.00408149	0.8731	0.180355	17	19333641	0.008774	0.64	17	19236954	0.0021	4.62E-14
rs12613500	C	G	C	G	0.0098	-0.000226002	0.4238	0.418588	2	180063738	0.007048	0.97	2	1.81E+08	0.0014	4.82E-12
rs12614263	A	G	A	G	0.0083	-0.0232764	0.4959	0.213256	2	4076206	0.008287	0.005	2	4123797	0.0014	3.38E-09
rs12620796	A	C	A	C	0.0121	-0.00703234	0.5496	0.725985	2	51060711	0.007647	0.36	2	51287849	0.0019	9.25E-11
rs12643771	T	C	T	C	0.013	0.00835698	0.3094	0.612632	4	139831949	0.007087	0.24	4	1.41E+08	0.0015	1.32E-17
rs12646216	T	C	T	C	0.0084	-0.00973323	0.3889	0.380644	4	45978039	0.006976	0.16	4	45980056	0.0014	4.56E-09
rs12646523	T	C	T	C	-0.0132	0.00809212	0.2483	0.663785	4	170023547	0.007036	0.25	4	1.71E+08	0.0016	3.79E-16
rs12659776	T	G	T	G	0.0085	0.0208087	0.5399	0.345581	5	168194269	0.007107	0.0034	5	1.68E+08	0.0014	1.64E-09
rs12667929	T	C	A	C	-0.0123	0.00277971	0.133	0.28122	7	114871962	0.007676	0.72	7	1.15E+08	0.0021	2.98E-09
rs12670376	A	G	A	G	0.0092	0.00281289	0.4464	0.555716	7	48783558	0.006932	0.68	7	48823154	0.0014	5.99E-11
rs1267062	C	G	C	G	0.0113	0.0149496	0.1781	0.017531	2	161143755	0.025014	0.55	2	1.62E+08	0.0018	7.40E-10
rs12694681	T	G	T	G	0.0102	0.00710432	0.6883	0.627522	2	225744525	0.006897	0.3	2	2.27E+08	0.0015	2.13E-11
rs12750688	C	G	C	G	0.0116	0.0362229	0.7406	0.936119	1	98292064	0.014222	0.011	1	98757620	0.0016	6.64E-13
rs12761761	T	C	T	C	0.0153	-0.0127248	0.2395	0.138329	10	131961871	0.009813	0.19	10	1.34E+08	0.0017	4.79E-20
rs12765185	A	T	A	T	-0.0088	0.001711	0.2769	0.139769	10	133163573	0.009983	0.86	10	1.35E+08	0.0016	2.96E-08
rs12774577	T	C	T	C	0.0142	0.0161538	0.8545	0.84318	10	103199730	0.009464	0.088	10	1.05E+08	0.002	8.69E-13
rs12789313	T	C	T	C	0.0092	-0.000734273	0.4907	0.880163	11	25037391	0.010058	0.94	11	25058937	0.0014	6.79E-11
rs12790196	T	C	T	C	-0.0101	-0.00595512	0.3263	0.125841	11	57730375	0.010388	0.57	11	57497847	0.0015	3.29E-11
rs12810587	T	C	T	C	0.0094	0.00143322	0.3256	0.715418	12	122191029	0.007393	0.85	12	1.23E+08	0.0015	3.70E-10

rs12891042	T	C	T	C	0.0092	-0.00825668	0.5853	0.622238	14	33549780	0.006977	0.24	14	34018986	0.0014	1.02E-10
rs12891191	C	G	C	G	0.008	0.00558412	0.5179	0.146254	14	57633565	0.009568	0.56	14	58100283	0.0014	1.21E-08
rs12912465	T	C	T	C	-0.0104	-0.0114906	0.269	0.017771	15	77017118	0.025179	0.65	15	77309459	0.0016	1.17E-10
rs12952191	T	C	T	C	-0.0081	0.012637	0.4895	0.620797	17	54000025	0.006897	0.067	17	52077386	0.0014	6.52E-09
rs12957463	A	G	A	G	0.0146	-0.00423353	0.7953	0.784582	18	39832264	0.008205	0.61	18	37412228	0.0018	9.20E-17
rs12959103	A	G	A	G	-0.009	-0.0165264	0.3021	0.056436	18	52641394	0.014104	0.24	18	50167764	0.0015	3.91E-09
rs12962845	C	G	C	G	-0.0123	-0.0247571	0.1325	0.191883	18	25074138	0.008654	0.0042	18	22654102	0.0021	2.38E-09
rs12967010	T	C	T	C	0.0114	-0.00891514	0.7746	0.920509	18	9645037	0.012441	0.47	18	9645035	0.0017	1.33E-11
rs12970264	A	G	A	G	-0.0085	-0.00728673	0.4386	0.389529	18	79867950	0.00709	0.3	18	77627950	0.0014	1.61E-09
rs12981405	T	C	T	C	-0.0112	-0.0078868	0.1664	0.289866	19	19540768	0.007419	0.29	19	19651577	0.0019	2.75E-09
rs13010288	T	G	T	G	0.0198	-0.0377746	0.1288	0.012968	2	51597374	0.029961	0.21	2	51824512	0.0021	3.44E-21
rs13016316	A	C	A	C	-0.0138	-0.0139355	0.0908	0.254803	2	224472471	0.007976	0.081	2	2.25E+08	0.0024	1.67E-08
rs1301838	T	C	T	C	-0.0096	0.0122242	0.3227	0.172911	11	99799783	0.009162	0.18	11	99670514	0.0015	1.29E-10
rs13029602	T	C	T	C	-0.0092	0.000706593	0.6059	0.753842	2	29352009	0.007812	0.93	2	29574875	0.0014	1.33E-10
rs13050131	A	C	A	C	-0.0081	-0.00452853	0.6647	0.888329	21	18645800	0.010494	0.67	21	20018118	0.0015	4.40E-08
rs13072665	A	T	A	G	-0.025	0.00592594	0.9451	0.064842	3	69944162	0.014202	0.68	3	69930625	0.0031	1.09E-15
rs13099165	T	G	T	G	-0.0118	-0.0115545	0.2029	0.057877	3	169022637	0.014846	0.44	3	1.69E+08	0.0017	1.09E-11
rs13117856	A	T	A	T	0.0084	0.0084746	0.7054	0.926513	4	173087608	0.013108	0.52	4	1.74E+08	0.0015	4.64E-08
rs13130765	C	G	C	G	-0.0091	-0.00280886	0.4721	0.327089	4	45161316	0.007323	0.7	4	45163333	0.0014	1.89E-10
rs13133213	A	G	A	G	0.0096	-0.00121832	0.498	0.623679	4	15645171	0.007082	0.86	4	15646794	0.0014	7.57E-12
rs13145650	T	C	T	C	-0.0173	-0.00783667	0.917	0.787464	4	122042189	0.008362	0.35	4	1.23E+08	0.0025	8.99E-12
rs13154429	C	G	C	G	-0.0136	0.00785953	0.1016	0.110711	5	60313123	0.010863	0.47	5	59608950	0.0023	5.02E-09
rs13163062	T	C	T	C	0.0115	-0.0100784	0.426	0.416427	5	107619750	0.006843	0.14	5	1.07E+08	0.0014	4.43E-16
rs13163845	T	C	T	C	-0.0149	-0.00165674	0.8488	0.67195	5	3264275	0.007234	0.82	5	3264389	0.002	4.66E-14
rs13168136	A	G	A	G	-0.0092	0.014441	0.7668	0.42171	5	26898520	0.006831	0.035	5	26898629	0.0017	2.57E-08
rs13169187	A	G	A	G	-0.0079	-0.00652103	0.5374	0.727906	5	52421123	0.007588	0.39	5	51716957	0.0014	2.01E-09
rs13177031	A	T	A	T	-0.0085	0.018179	0.3703	0.331892	5	61557696	0.007262	0.012	5	60853523	0.0015	4.88E-09
rs13190235	T	C	T	C	-0.0138	0.0272499	0.9036	0.720221	5	66668955	0.007585	0.00033	5	65964783	0.0024	5.66E-09
rs13197257	T	G	T	G	0.0109	0.00365827	0.279	0.111191	6	128012537	0.010825	0.74	6	1.28E+08	0.0016	2.83E-12
rs1320139	C	G	C	G	-0.0149	0.00564571	0.427	0.568684	2	143400608	0.006795	0.41	2	1.44E+08	0.0014	7.62E-26
rs13212041	T	C	T	C	0.0101	0.00825724	0.7978	0.804755	6	77461407	0.008539	0.33	6	78171124	0.0018	7.42E-09
rs13246220	T	G	T	G	-0.0093	0.00394139	0.6117	0.651777	7	39284654	0.007166	0.58	7	39324253	0.0014	8.53E-11
rs13266287	A	T	A	T	-0.0085	-0.00206179	0.6687	0.240154	8	18014089	0.007914	0.79	8	17871598	0.0015	1.75E-08
rs13281564	A	G	A	G	0.0088	-0.010765	0.5129	0.619597	8	118471077	0.006973	0.12	8	1.19E+08	0.0014	4.02E-10
rs13284516	C	G	C	G	-0.0082	-0.00256618	0.5452	0.379923	9	23423787	0.00694	0.71	9	23423785	0.0014	6.78E-09
rs1329125	T	C	T	C	-0.0097	0.013441	0.3273	0.04635	1	234605134	0.016354	0.41	1	2.35E+08	0.0015	8.37E-11
rs13296345	T	C	T	C	0.009	0.00969176	0.6299	0.681076	9	14791350	0.007248	0.18	9	14791348	0.0015	5.35E-10
rs13318986	A	G	A	G	0.0124	0.00630709	0.3311	0.394813	3	82484488	0.007134	0.38	3	82533639	0.0015	1.17E-16
rs13327482	A	G	A	G	-0.0121	0.00649265	0.8221	0.96806	3	197061492	0.018813	0.73	3	1.97E+08	0.0018	4.47E-11
rs1334297	A	G	A	G	0.0257	0.00812431	0.7375	0.908261	13	57761241	0.011932	0.5	13	58335375	0.0016	2.53E-58
rs1335482	T	C	T	C	0.0096	0.00391158	0.5295	0.959414	1	78137779	0.016445	0.81	1	78603463	0.0014	1.01E-11
rs13361043	T	C	T	C	-0.0158	-0.00768825	0.9329	0.597743	5	59023136	0.006868	0.26	5	58318963	0.0028	1.84E-08
rs13381557	A	G	A	G	-0.0087	-0.00474961	0.5413	0.629683	18	46933408	0.00714	0.51	18	44513371	0.0014	7.40E-10
rs13397444	A	G	A	G	0.0095	0.000691771	0.2977	0.184438	2	171759913	0.008867	0.94	2	1.73E+08	0.0015	6.00E-10
rs13398860	A	G	A	G	0.0086	-0.00589345	0.6529	0.774015	2	50465802	0.00805	0.46	2	50692940	0.0015	5.89E-09
rs13402497	A	T	A	T	-0.008	-0.000348073	0.4902	0.487752	2	115068741	0.006897	0.96	2	1.16E+08	0.0014	1.33E-08
rs1340561	T	C	A	C	0.0152	0.0432193	0.0738	0.017531	13	58910478	0.024738	0.081	13	59496462	0.0027	1.56E-08
rs13417733	T	C	T	C	-0.0178	-0.0540439	0.0583	0.988953	2	174357348	0.032306	0.094	2	1.75E+08	0.003	2.91E-09
rs13422673	T	C	T	C	-0.011	0.0022627	0.4659	0.165946	2	154617843	0.009176	0.81	2	1.55E+08	0.0014	6.48E-15
rs13425585	C	G	C	G	-0.0089	0.0196653	0.5084	0.243276	2	156630761	0.007864	0.012	2	1.57E+08	0.0014	2.32E-10
rs137079	T	C	T	C	0.0128	0.0130112	0.1375	0.129203	22	42581901	0.010222	0.2	22	42977907	0.002	3.06E-10
rs139244147	A	G	A	G	-0.0193	0.000581064	0.0604	0.130163	7	112272683	0.010228	0.95	7	1.12E+08	0.003	7.71E-11
rs1392816	T	C	T	C	0.0082	-0.00483966	0.3831	0.15586	1	66015505	0.009324	0.6	1	66481188	0.0014	1.61E-08
rs1404549	A	G	A	G	-0.0089	-0.00959957	0.3278	0.67243	3	65469248	0.00714	0.18	3	65454923	0.0015	2.38E-09
rs1405876	T	G	T	G	0.0109	0.0055993	0.6403	0.902257	7	86608265	0.0114	0.62	7	86237581	0.0015	7.84E-14

rs1408284	C	G	C	G	-0.0127	-0.0125109	0.8639	0.959894	6	93183868	0.017408	0.47	6	93893586	0.002	5.71E-10
rs140954946	T	C	T	C	0.0176	0.0148096	0.0843	0.009606	16	89943764	0.035319	0.67	16	89990843	0.0026	6.25E-12
rs141729694	T	C	T	C	0.0195	-0.00544745	0.0768	0.054515	5	88703554	0.014983	0.72	5	87999371	0.0026	1.49E-13
rs142014757	A	G	A	G	0.0102	0.0359538	0.8085	0.994236	18	79751268	0.045477	0.43	18	77511268	0.0018	1.74E-08
rs1426619	T	C	T	C	0.0096	0.00153447	0.4346	0.527378	10	88331783	0.006843	0.82	10	90091540	0.0014	1.40E-11
rs1427298	T	C	T	C	0.0086	-0.00052741	0.4264	0.557397	2	144456854	0.006813	0.94	2	1.45E+08	0.0014	1.33E-09
rs1427829	A	G	A	G	-0.0083	-0.0106353	0.4546	0.395053	12	89366967	0.007216	0.14	12	89760744	0.0014	3.29E-09
rs143743568	A	G	A	G	0.0118	0.00029474	0.154	0.449808	22	38893009	0.006697	0.96	22	39289014	0.002	4.99E-09
rs143812851	A	G	A	G	-0.0113	-0.00924319	0.1735	0.180836	16	61739945	0.009007	0.3	16	61773849	0.0019	1.03E-09
rs1440930	C	G	C	G	0.0081	0.000829348	0.4535	0.48439	4	65036624	0.006812	0.9	4	65902342	0.0014	8.94E-09
rs1445591	A	G	A	G	0.0085	0.00186681	0.6986	0.948847	1	72494328	0.014823	0.9	1	72960011	0.0015	4.02E-08
rs145489304	T	C	T	A	0.024	0.00742687	0.0426	0.055716	1	75180787	0.014662	0.61	1	75562222	0.0035	5.88E-12
rs1464297	T	C	T	C	-0.0109	0.00651944	0.649	0.505043	2	139896180	0.006792	0.34	2	1.41E+08	0.0015	1.49E-13
rs1467737	T	C	T	C	-0.0084	-0.000147422	0.4746	0.438761	9	122220221	0.006894	0.98	9	1.25E+08	0.0014	3.52E-09
rs150537577	A	G	A	G	-0.0151	0.00232838	0.9195	0.878482	4	711840	0.01043	0.82	4	705629	0.0026	9.50E-09
rs151381	T	C	T	C	-0.0087	0.0066075	0.4927	0.442363	4	102197611	0.006841	0.33	4	1.03E+08	0.0014	7.01E-10
rs1527878	A	G	A	G	-0.0105	-0.00464379	0.7566	0.944525	2	182581808	0.01501	0.76	2	1.83E+08	0.0016	1.49E-10
rs1529597	A	C	A	C	0.0223	-0.0604895	0.9637	0.992315	15	56897866	0.037141	0.1	15	57190064	0.0038	3.36E-09
rs1538389	T	C	T	C	-0.0109	0.000670539	0.1894	0.087176	1	111784522	0.012265	0.96	1	1.12E+08	0.0018	1.27E-09
rs1542354	A	G	A	G	-0.0084	-0.011098	0.4764	0.615754	2	220949365	0.007165	0.12	2	2.22E+08	0.0014	2.72E-09
rs1544	A	G	A	G	-0.0096	0.00744361	0.2636	0.293228	21	31713272	0.007495	0.32	21	33085585	0.0016	1.76E-09
rs1554798	A	C	A	C	-0.008	-0.00493877	0.4667	0.600624	2	155978547	0.007031	0.48	2	1.57E+08	0.0014	1.22E-08
rs1564347	T	G	T	G	0.0088	-0.00111625	0.3433	0.555716	15	73146507	0.0068	0.87	15	73438848	0.0015	2.39E-09
rs1569092	A	G	A	G	0.016	-0.0118079	0.152	0.092219	1	74393040	0.011743	0.31	1	74858724	0.002	2.12E-16
rs1593022	T	G	T	G	-0.0099	-0.00357065	0.2256	0.150576	5	107349862	0.009269	0.7	5	1.07E+08	0.0017	4.69E-09
rs1603460	T	G	T	G	0.009	0.000356336	0.4206	0.345341	12	22939692	0.00716	0.96	12	23092626	0.0014	2.20E-10
rs162445	A	G	A	G	0.0152	-0.00931522	0.0788	0.221182	5	7943133	0.008396	0.27	5	7943246	0.0026	5.72E-09
rs164938	T	G	T	G	-0.0088	-0.00185598	0.3922	0.71854	3	10273419	0.007561	0.81	3	10315103	0.0014	1.05E-09
rs1656614	C	G	C	G	0.0122	0.00550377	0.314	0.094861	15	47508316	0.011525	0.63	15	47800513	0.0015	6.91E-16
rs1671269	T	C	T	C	-0.01	0.0111324	0.7524	0.32757	18	52583601	0.007302	0.13	18	50109971	0.0016	7.40E-10
rs1671770	A	C	A	C	0.0125	0.00243409	0.1786	0.170029	12	120508765	0.009197	0.79	12	1.21E+08	0.0018	9.23E-12
rs16851779	T	C	T	C	-0.0153	0.0223172	0.9246	0.977426	2	214325226	0.023109	0.33	2	2.15E+08	0.0027	9.24E-09
rs16871807	T	C	T	C	0.009	-0.00202185	0.3297	0.563401	5	3437183	0.006888	0.77	5	3437297	0.0015	1.59E-09
rs1689510	C	G	C	G	0.0194	-0.00113333	0.3315	0.196206	12	56002984	0.008567	0.89	12	56396768	0.0015	1.35E-38
rs16901689	T	G	T	G	0.0111	0.000351523	0.1634	0.082613	5	11507256	0.012389	0.98	5	11507368	0.0019	5.17E-09
rs1693584	T	C	T	C	-0.0087	-0.0106004	0.6856	0.735591	8	100736689	0.007836	0.18	8	1.02E+08	0.0015	8.79E-09
rs16958559	T	G	T	G	0.0096	0.0537902	0.7865	0.989433	17	57581872	0.032256	0.095	17	55659233	0.0017	2.07E-08
rs16975275	A	G	A	G	-0.0102	0.00798763	0.2306	0.247358	15	95503327	0.007915	0.31	15	96046556	0.0017	8.14E-10
rs17069646	T	C	T	C	0.0085	0.0150398	0.6881	0.630163	5	168025246	0.007081	0.034	5	1.67E+08	0.0015	2.01E-08
rs17110109	T	C	T	C	-0.0093	-0.00872295	0.6163	0.526177	12	54275124	0.006851	0.2	12	54668908	0.0014	1.47E-10
rs17133297	A	T	A	T	-0.0146	0.00909237	0.8958	0.661864	7	3415769	0.007108	0.2	7	3455401	0.0023	2.28E-10
rs17144467	A	C	A	C	0.0098	0.000458658	0.6867	0.633285	7	122458728	0.007208	0.95	7	1.22E+08	0.0015	8.74E-11
rs17148998	A	G	A	G	0.0101	-0.00104113	0.2042	0.027618	10	10867438	0.021095	0.96	10	10909401	0.0017	6.98E-09
rs17170519	C	G	C	G	0.0132	0.00527481	0.8991	0.712296	7	32821618	0.007343	0.47	7	32861230	0.0023	1.99E-08
rs1717204	A	C	A	C	-0.0117	0.00299569	0.1818	0.204371	3	118742298	0.008378	0.72	3	1.18E+08	0.0018	1.20E-10
rs1718188	A	T	A	T	0.0096	-0.004465	0.4365	0.560279	12	120906090	0.006744	0.51	12	1.21E+08	0.0014	9.05E-12
rs17186106	T	G	T	G	0.0108	0.00312421	0.1771	0.145293	4	90782090	0.009683	0.75	4	91703241	0.0018	3.95E-09
rs17246529	T	C	T	A	0.0185	0.0643856	0.942	0.012968	2	156136046	0.029948	0.032	2	1.57E+08	0.003	6.44E-10
rs17248751	A	G	A	G	-0.0136	0.00702968	0.7827	0.991835	16	61545714	0.037158	0.85	16	61579618	0.0017	1.28E-15
rs1729412	T	C	T	C	-0.0107	-0.00539344	0.4224	0.74928	2	200218875	0.007915	0.5	2	2.01E+08	0.0014	4.05E-14
rs173003	A	C	A	C	-0.0082	-0.0107025	0.4934	0.331652	19	35750059	0.007216	0.14	19	36240960	0.0014	5.50E-09
rs1734370	A	G	A	G	0.0099	0.0129091	0.7171	0.620077	2	10838584	0.007051	0.067	2	10978710	0.0016	2.42E-10
rs1738050	C	G	C	G	-0.0093	0.00572145	0.6197	0.523535	1	44241623	0.00676	0.4	1	44707295	0.0014	1.42E-10
rs17411339	A	G	A	G	0.014	-0.00370545	0.5612	0.959894	18	53280989	0.017097	0.83	18	50807359	0.0014	5.10E-23
rs17428076	C	G	C	G	0.0137	0.00527273	0.7598	0.928674	2	171987022	0.013088	0.69	2	1.73E+08	0.0016	6.41E-17

rs1747714	T	C	T	C	0.0106	0.00441852	0.5287	0.229587	10	11119353	0.008089	0.58	10	11161316	0.0014	3.82E-14
rs1747817	T	C	T	C	-0.0092	0.0108493	0.7614	0.90682	1	197742362	0.011497	0.35	1	1.98E+08	0.0016	2.29E-08
rs17489649	A	G	A	G	0.0127	0.0259511	0.6788	0.978867	5	109820483	0.023837	0.28	5	1.09E+08	0.0015	2.77E-17
rs175325	A	T	A	T	-0.01	0.0019284	0.5966	0.289625	20	22336672	0.00734	0.79	20	22317310	0.0014	2.24E-12
rs17563464	A	C	A	C	-0.0147	0.00137561	0.2173	0.012728	5	26913666	0.030792	0.96	5	26913774	0.0017	1.74E-17
rs17565975	A	G	A	G	-0.0106	-0.00517172	0.5563	0.629203	11	111716226	0.007134	0.47	11	1.12E+08	0.0014	5.54E-14
rs17568389	A	T	A	T	0.0127	-0.000602101	0.5087	0.266811	7	8052246	0.007744	0.94	7	8091876	0.0014	1.62E-19
rs1758747	A	G	A	G	0.0092	0.0050374	0.6962	0.633045	9	25300645	0.007091	0.48	9	25300643	0.0015	1.64E-09
rs176218	T	G	T	G	0.02	0.0014372	0.1958	0.519212	14	29131300	0.006662	0.83	14	29600506	0.0018	1.10E-29
rs17622379	T	C	T	C	-0.0113	0.0152584	0.1853	0.151537	17	52649486	0.00956	0.11	17	50726846	0.0018	4.52E-10
rs17669337	T	C	T	C	-0.011	0.0143027	0.4133	0.497358	5	92852225	0.007053	0.043	5	92187932	0.0014	1.10E-14
rs17686649	T	C	T	C	-0.0098	-0.0279639	0.2192	0.058598	6	92858809	0.014015	0.046	6	93568527	0.0017	6.37E-09
rs17732878	T	C	T	C	-0.0095	-0.00762691	0.7912	0.935879	17	7459040	0.013406	0.57	17	7362359	0.0017	4.06E-08
rs17742342	A	C	A	C	-0.0119	0.00188564	0.802	0.893852	2	147876367	0.011009	0.86	2	1.49E+08	0.0018	1.59E-11
rs17747544	A	G	A	G	-0.009	-0.000461763	0.4701	0.238953	20	42583273	0.007943	0.95	20	41211913	0.0014	1.74E-10
rs1779549	A	C	A	C	0.0081	0.00291824	0.46	0.792027	14	84173672	0.008313	0.73	14	84640016	0.0014	8.45E-09
rs17882802	A	G	A	G	0.0084	0.0173897	0.4295	0.073247	10	100223656	0.013181	0.19	10	1.02E+08	0.0014	3.00E-09
rs1792602	A	G	A	G	-0.0097	0.0125574	0.3824	0.301393	11	90706465	0.00744	0.091	11	90439633	0.0014	1.90E-11
rs181216	T	C	T	C	-0.0104	-0.0256044	0.2225	0.978867	17	58104711	0.023835	0.28	17	56179482	0.0017	8.19E-10
rs1835339	T	C	T	C	0.0084	-0.00654268	0.6132	0.64073	2	182528953	0.007074	0.36	2	1.83E+08	0.0014	5.59E-09
rs183869217	C	G	C	G	0.0174	-0.0047914	0.9409	0.95365	2	81524216	0.016185	0.77	2	81751340	0.003	4.72E-09
rs1841023	A	C	A	C	-0.0092	6.57E-05	0.6904	0.802834	4	162814432	0.008593	0.99	4	1.64E+08	0.0015	1.64E-09
rs1842713	A	G	A	G	-0.0117	0.00373658	0.7882	0.462776	17	34873366	0.006745	0.58	17	33200385	0.0017	9.67E-12
rs1843815	A	T	A	T	0.0077	-0.000130979	0.4635	0.428915	1	98124159	0.00697	0.99	1	98589715	0.0014	4.40E-08
rs1861786	A	G	A	G	-0.0081	0.00132579	0.3813	0.492795	12	13847533	0.006938	0.85	12	14000467	0.0014	2.39E-08
rs1865955	T	C	T	C	0.0122	-0.00197663	0.8244	0.463256	10	117078479	0.006628	0.77	10	1.19E+08	0.0019	4.06E-11
rs1866823	A	G	A	G	0.0107	0.00667783	0.5444	0.404179	8	56524018	0.00695	0.34	8	57436577	0.0014	3.09E-14
rs187580	T	G	T	G	-0.0104	0.0035326	0.7671	0.820365	5	103291654	0.00879	0.69	5	1.03E+08	0.0017	3.56E-10
rs1880692	A	G	A	G	0.0086	0.00896624	0.5346	0.483429	11	80627025	0.006897	0.19	11	80338069	0.0014	1.09E-09
rs1890132	T	C	T	C	-0.0089	-0.0224287	0.7056	0.974304	1	199458193	0.019791	0.26	1	1.99E+08	0.0015	6.56E-09
rs1898111	A	G	A	G	0.0101	0.00626879	0.8245	0.739433	15	47600101	0.007947	0.43	15	47892298	0.0018	4.72E-08
rs1910005	T	C	T	C	-0.009	0.00247014	0.708	0.683718	5	81815047	0.00726	0.73	5	81110866	0.0015	5.66E-09
rs1918394	T	C	T	C	0.0119	0.00135621	0.1658	0.303554	3	74854753	0.007418	0.85	3	74903904	0.0019	3.04E-10
rs1933264	T	C	T	C	0.01	0.00268979	0.7491	0.65658	6	153070483	0.007162	0.71	6	1.53E+08	0.0016	5.85E-10
rs1952183	A	G	A	G	-0.0089	-4.75E-05	0.5078	0.731028	14	89377767	0.007836	1	14	89844111	0.0014	2.76E-10
rs1955250	A	C	A	C	-0.014	0.00888833	0.9137	0.85951	4	117593081	0.00957	0.35	4	1.19E+08	0.0025	2.26E-08
rs1980129	A	G	A	G	0.0085	-0.00321285	0.4738	0.548991	4	17044288	0.006796	0.64	4	17045911	0.0014	1.17E-09
rs1991585	T	C	T	C	-0.0101	-0.014116	0.706	0.225985	2	16465902	0.007893	0.074	2	16647170	0.0015	6.26E-11
rs1995181	A	T	A	T	-0.0078	0.0158313	0.4598	0.302354	12	24042114	0.007458	0.034	12	24195048	0.0014	3.61E-08
rs199968	T	C	C	G	0.0207	-0.0106691	0.0485	0.020893	1	181638002	0.023685	0.65	1	1.82E+08	0.0033	2.48E-10
rs2007655	T	G	T	G	0.0086	0.011387	0.4976	0.592939	10	34297761	0.006864	0.097	10	34586689	0.0014	8.64E-10
rs2011603	A	G	A	G	-0.0091	0.00613617	0.7359	0.589817	4	18023861	0.006827	0.37	4	18025484	0.0016	1.12E-08
rs2014830	T	C	T	C	0.011	-0.00252767	0.3139	0.753122	3	50134964	0.007895	0.75	3	50172397	0.0015	4.72E-13
rs201490	T	C	T	C	-0.0112	-0.00071029	0.559	0.132565	7	102108630	0.009774	0.94	7	1.02E+08	0.0019	5.92E-09
rs2023016	C	G	C	G	0.0107	-0.00372163	0.7899	0.535062	6	162560234	0.006892	0.59	6	1.63E+08	0.0017	4.91E-10
rs2024568	T	C	T	C	-0.0097	0.00822212	0.2535	0.457012	20	46103450	0.006825	0.23	20	44732089	0.0016	1.61E-09
rs2029401	A	G	A	G	-0.0091	0.00286815	0.4278	0.222863	5	93555323	0.007939	0.72	5	92891029	0.0014	1.53E-10
rs2034631	T	C	T	C	0.0089	-0.00477779	0.6508	0.558117	4	81304375	0.006981	0.49	4	82225529	0.0015	2.64E-09
rs2039204	A	T	A	T	0.0082	0.00201959	0.5296	0.329971	9	127608828	0.007189	0.78	9	1.3E+08	0.0014	5.23E-09
rs2055940	A	G	A	G	0.0082	0.00704961	0.3215	0.143612	4	46995896	0.009796	0.47	4	46997913	0.0015	4.95E-08
rs2077235	T	C	T	C	0.0097	0.0133229	0.2373	0.31196	16	3579226	0.007341	0.07	16	3629227	0.0017	5.20E-09
rs2081652	A	T	A	T	0.0123	0.00895222	0.661	0.864553	4	158941761	0.010011	0.37	4	1.6E+08	0.0015	1.09E-16
rs2082317	T	C	T	C	-0.0078	-0.0120694	0.5301	0.506484	4	170903398	0.006888	0.08	4	1.72E+08	0.0014	3.19E-08
rs2088913	A	G	A	G	0.0081	-0.00137433	0.5732	0.497358	3	117591222	0.006832	0.84	3	1.17E+08	0.0014	9.61E-09
rs2092248	T	C	T	C	-0.0083	0.00616817	0.331	0.329971	6	19205227	0.007424	0.41	6	19205458	0.0015	2.43E-08

rs2098526	A	G	A	G	-0.0241	-0.0160039	0.0279	0.102546	2	161988766	0.011198	0.15	2	1.63E+08	0.0043	1.62E-08
rs2100249	T	G	T	G	-0.0089	0.0104341	0.3488	0.529059	7	114208442	0.006914	0.13	7	1.14E+08	0.0015	1.91E-09
rs2131167	A	G	A	G	0.008	-0.00108739	0.4458	0.212296	12	99239411	0.008388	0.9	12	99633189	0.0014	1.52E-08
rs214626	A	G	A	G	0.0101	-0.00593239	0.1894	0.42243	9	132843197	0.006924	0.39	9	1.36E+08	0.0018	1.58E-08
rs2160514	A	C	A	C	-0.0094	0.00757645	0.5548	0.655139	12	16603574	0.007191	0.29	12	16756508	0.0014	2.54E-11
rs2179152	T	C	T	C	-0.0131	-0.00265261	0.3673	0.132565	6	26325660	0.009823	0.79	6	26325888	0.0015	1.68E-19
rs2183271	T	C	T	C	0.0084	0.00844827	0.6364	0.919549	10	21668300	0.01189	0.48	10	21957229	0.0015	1.91E-08
rs2196281	A	G	A	G	0.0172	0.0117337	0.0884	0.982469	5	59907581	0.025678	0.65	5	59152140	0.0025	3.63E-12
rs2199409	T	C	T	C	0.0103	0.00385287	0.7962	0.805956	11	39893684	0.008649	0.66	11	39915234	0.0017	3.14E-09
rs2216144	T	C	T	C	0.0098	-0.00342483	0.5306	0.527618	12	97272700	0.006791	0.61	12	97666478	0.0014	2.58E-12
rs2220926	T	C	T	C	-0.0095	-0.0025349	0.4308	0.500961	18	61276153	0.006817	0.71	18	58943386	0.0014	1.84E-11
rs2250660	C	G	C	G	0.008	0.00852945	0.5009	0.209414	2	232887841	0.008507	0.32	2	2.34E+08	0.0014	1.20E-08
rs2252098	T	C	T	C	-0.0077	0.00520944	0.4703	0.130644	20	53003762	0.0103	0.61	20	51620301	0.0014	4.74E-08
rs2254681	A	G	A	G	0.0101	0.00377832	0.2517	0.219741	1	159278258	0.008266	0.65	1	1.59E+08	0.0016	4.25E-10
rs2276209	A	C	A	C	0.0085	0.00601332	0.704	0.470221	18	39362493	0.006745	0.37	18	36942457	0.0015	3.49E-08
rs2283076	A	G	A	G	0.0115	-0.0152559	0.7786	0.9378	7	126838136	0.014098	0.28	7	1.26E+08	0.0017	1.05E-11
rs2287838	A	G	A	G	-0.0104	-0.0112402	0.5504	0.285783	19	9848338	0.007506	0.13	19	9959014	0.0014	1.27E-13
rs2290601	T	C	T	C	-0.0149	0.0121793	0.772	0.5622	3	108393913	0.00681	0.074	3	1.08E+08	0.0017	4.49E-19
rs2297293	C	G	C	G	0.0099	0.00192913	0.3141	0.630644	21	45705132	0.006985	0.78	21	47125046	0.0015	8.80E-11
rs2299156	T	C	T	C	0.0099	-0.00480356	0.189	0.299472	7	50696707	0.007229	0.51	7	50764404	0.0018	3.15E-08
rs2314338	T	C	T	C	0.0093	-0.023702	0.7304	0.705091	17	40188233	0.007529	0.0016	17	38344485	0.0016	9.50E-09
rs2321157	A	G	A	G	-0.0081	-0.000764358	0.5303	0.279059	13	58162767	0.007602	0.92	13	58736901	0.0014	7.64E-09
rs232496	T	C	T	C	0.009	-0.00463115	0.3657	0.39049	21	21362089	0.007021	0.51	21	22734409	0.0015	7.14E-10
rs2332179	A	G	A	G	0.0121	0.0225624	0.861	0.946686	3	122360583	0.015064	0.13	3	1.22E+08	0.002	2.76E-09
rs2358628	A	C	A	C	-0.0091	-0.00805781	0.3122	0.804035	14	74165054	0.008606	0.35	14	74631757	0.0015	1.78E-09
rs236318	C	G	C	G	-0.0102	-0.0101308	0.2346	0.355427	1	93584664	0.007165	0.16	1	94050221	0.0017	7.40E-10
rs2364544	A	G	A	G	-0.0105	0.00590633	0.3912	0.337176	1	41367490	0.007166	0.41	1	41833162	0.0014	2.91E-13
rs2365376	A	C	A	C	0.0092	0.00116263	0.3503	0.253842	5	89444506	0.007785	0.88	5	88740323	0.0015	3.93E-10
rs2368831	T	C	T	C	-0.0092	0.0071802	0.5736	0.216138	12	79119	0.008273	0.39	12	188285	0.0014	1.63E-10
rs2373124	A	G	A	G	-0.0096	-0.0061181	0.7604	0.950288	7	86200956	0.015879	0.7	7	85830272	0.0016	4.35E-09
rs2406253	A	G	A	G	0.0135	-0.00883364	0.8053	0.955572	7	100479650	0.016764	0.6	7	1E+08	0.0018	3.58E-14
rs2414072	A	T	A	T	-0.0083	-0.0167823	0.4443	0.476465	15	50558365	0.006688	0.012	15	50850562	0.0014	4.45E-09
rs2416214	A	C	A	C	0.0079	0.00679689	0.4367	0.565082	5	109607784	0.006831	0.32	5	1.09E+08	0.0014	2.09E-08
rs2416759	A	G	A	G	-0.0093	-0.0154234	0.6986	0.595101	9	120595984	0.006948	0.026	9	1.23E+08	0.0015	1.45E-09
rs2416845	T	C	T	C	0.0263	-0.0300655	0.9775	0.96878	9	121993153	0.019733	0.13	9	1.25E+08	0.0048	4.19E-08
rs2436760	T	C	T	C	0.0232	-0.00877094	0.9717	0.947887	6	40406549	0.015518	0.57	6	40374288	0.0042	4.52E-08
rs2447097	T	G	T	G	0.0096	0.0134162	0.4596	0.269693	17	2374770	0.007702	0.082	17	2278064	0.0014	1.10E-11
rs2458370	T	C	T	C	-0.0086	0.00525019	0.6409	0.557397	1	117332322	0.006844	0.44	1	1.18E+08	0.0015	4.23E-09
rs2469226	A	T	A	T	0.0096	0.00742318	0.2228	0.169308	15	76954649	0.009077	0.41	15	77246990	0.0017	1.28E-08
rs2470966	C	G	C	G	-0.0103	-0.00343262	0.6998	0.895533	7	104790442	0.010564	0.75	7	1.04E+08	0.0015	1.62E-11
rs2517086	C	G	C	G	-0.0088	-0.00111584	0.5721	0.164025	8	17184907	0.009318	0.9	8	17042416	0.0014	8.00E-10
rs2521602	A	G	A	G	-0.0266	-0.01832	0.0219	0.043948	17	44259056	0.016927	0.28	17	42336424	0.0049	4.17E-08
rs2529069	T	C	T	C	-0.0106	-0.00626616	0.7343	0.284822	7	24531419	0.007602	0.41	7	24571038	0.0016	2.66E-11
rs2542673	A	C	A	C	0.0088	0.0149123	0.3243	0.358549	16	54172803	0.007112	0.036	16	54206715	0.0015	6.30E-09
rs2545795	A	C	A	C	0.0125	0.00139123	0.4437	0.75048	5	177460105	0.007901	0.86	5	1.77E+08	0.0014	1.03E-18
rs255053	A	G	A	G	-0.0111	-0.0175661	0.1913	0.112632	16	67986589	0.010657	0.099	16	68020492	0.0018	6.68E-10
rs2569041	T	C	T	C	0.0077	0.0031939	0.5421	0.12464	5	168083352	0.010129	0.75	5	1.68E+08	0.0014	4.85E-08
rs2570497	T	C	T	C	-0.0121	-0.0038705	0.6367	0.603746	2	103825088	0.006945	0.58	2	1.04E+08	0.0015	1.27E-16
rs2665668	A	G	A	G	-0.0094	-0.0118639	0.6413	0.754563	2	60541843	0.007897	0.13	2	60768978	0.0015	1.25E-10
rs2718277	T	C	T	C	0.0142	0.0106636	0.0832	0.041066	7	74719610	0.017237	0.54	7	74133929	0.0026	2.99E-08
rs2718791	T	C	T	C	-0.0081	0.00101074	0.6452	0.814121	3	181213607	0.00868	0.91	3	1.81E+08	0.0015	4.02E-08
rs27220	A	G	A	G	-0.0114	0.00275097	0.3562	0.611912	5	60479309	0.00693	0.69	5	59775136	0.0015	7.52E-15
rs2725370	T	C	T	C	-0.0141	0.00840556	0.3017	0.066523	8	30995310	0.012631	0.51	8	30852826	0.0015	4.96E-20
rs2736752	T	G	T	G	0.0095	-0.0111309	0.7869	0.987032	3	60831661	0.028926	0.7	3	60817322	0.0017	3.24E-08
rs2740795	A	T	A	T	0.0098	-0.00322093	0.7377	0.43684	8	90915227	0.006818	0.64	8	91927455	0.0016	7.71E-10

rs2761438	A	G	A	G	0.0098	0.00339898	0.3807	0.683477	1	110209517	0.007478	0.65	1	1.11E+08	0.0015	1.59E-11
rs2764684	T	C	T	C	0.0143	-0.0696713	0.8266	0.992795	1	58072247	0.040705	0.087	1	58537919	0.0019	9.99E-15
rs2805064	C	G	C	G	0.0093	-0.023831	0.7291	0.821806	9	24530617	0.008909	0.0075	9	24530615	0.0016	3.54E-09
rs28482086	A	G	A	G	0.0086	0.00726473	0.3494	0.226705	1	74760568	0.00809	0.37	1	75226252	0.0015	4.51E-09
rs28505285	C	G	C	G	0.0106	0.0207368	0.1884	0.012248	7	44338055	0.030205	0.49	7	44377654	0.0018	3.50E-09
rs28513882	A	G	A	G	-0.011	-0.0249142	0.1783	0.005283	3	107577781	0.045421	0.58	3	1.07E+08	0.0018	2.03E-09
rs2852349	T	C	T	C	-0.0093	0.00115056	0.5278	0.579011	18	39602943	0.006943	0.87	18	37182907	0.0014	3.78E-11
rs28540013	A	G	A	G	-0.0213	-0.0682346	0.0614	0.005043	8	142215968	0.048539	0.16	8	1.43E+08	0.0029	3.62E-13
rs28587776	T	C	T	C	0.0088	-0.00637683	0.3541	0.278578	5	177603176	0.007481	0.39	5	1.77E+08	0.0015	2.55E-09
rs28669886	A	G	A	G	-0.0084	0.0179111	0.3548	0.087656	12	106328923	0.011447	0.12	12	1.07E+08	0.0015	8.31E-09
rs2885198	A	G	A	G	0.0088	0.00116395	0.5318	0.438761	3	143917579	0.006688	0.86	3	1.44E+08	0.0014	4.63E-10
rs2898191	A	C	A	C	0.0093	-0.00504735	0.6921	0.788425	21	32916201	0.00827	0.54	21	34288509	0.0015	1.88E-09
rs2910823	T	G	T	G	0.0115	0.00725599	0.5359	0.147935	5	60202348	0.009592	0.45	5	59498175	0.0014	2.50E-16
rs2916490	A	G	A	G	-0.0092	0.00277642	0.3023	0.354467	2	79965226	0.007129	0.7	2	80192352	0.0015	1.92E-09
rs2923424	A	G	A	G	0.012	0.0100524	0.6069	0.549952	8	42527081	0.006905	0.15	8	42382222	0.0014	6.31E-17
rs2929032	A	G	A	G	-0.0081	0.00350606	0.4654	0.456052	8	55459185	0.006674	0.6	8	56371745	0.0014	8.40E-09
rs2929860	T	C	T	C	0.011	-0.0244456	0.1878	0.045869	3	128973098	0.016277	0.13	3	1.29E+08	0.0018	1.33E-09
rs2942884	A	G	A	G	-0.009	-0.00110744	0.4639	0.696446	2	100707116	0.007394	0.88	2	1.01E+08	0.0014	2.27E-10
rs2954114	A	C	A	C	-0.0081	0.0044244	0.468	0.558117	12	121731978	0.006952	0.52	12	1.22E+08	0.0014	9.40E-09
rs2958182	A	T	A	T	0.0082	0.00336498	0.3414	0.165226	18	55481790	0.009336	0.72	18	53149021	0.0015	3.46E-08
rs2962378	A	G	A	G	0.0101	0.00341697	0.7872	0.6244	5	91654393	0.007016	0.63	5	90950210	0.0017	4.30E-09
rs2964255	A	G	A	G	0.0088	0.00378292	0.3066	0.612152	5	152672772	0.006942	0.59	5	1.52E+08	0.0015	6.34E-09
rs2977464	T	C	T	C	0.0102	-0.00773025	0.1869	0.318444	8	140535094	0.007379	0.29	8	1.42E+08	0.0018	1.46E-08
rs2980813	A	T	A	T	0.0088	-0.00101002	0.4692	0.448367	8	40192491	0.00682	0.88	8	40050010	0.0014	4.28E-10
rs2989476	C	G	C	G	0.0078	0.00049478	0.4193	0.644573	1	60593587	0.00728	0.95	1	61059259	0.0014	3.73E-08
rs2994326	T	C	T	C	-0.01	0.00580575	0.1852	0.125841	1	243487724	0.010023	0.56	1	2.44E+08	0.0018	3.38E-08
rs2998299	T	G	T	G	-0.0136	-0.0112419	0.2137	0.242555	14	84531019	0.008104	0.17	14	84997363	0.0017	1.51E-15
rs3128341	T	C	T	C	-0.0165	0.00122986	0.2481	0.092699	1	72284165	0.011454	0.91	1	72762169	0.0016	2.51E-24
rs312927	A	G	A	G	-0.0147	0.00325349	0.1203	0.024976	19	3274772	0.020865	0.88	19	3274770	0.0022	2.52E-11
rs322614	A	G	A	G	-0.0087	0.00707466	0.3817	0.473583	11	29734000	0.006841	0.3	11	29755547	0.0014	1.43E-09
rs322744	T	C	T	C	-0.009	0.00240072	0.2609	0.362632	7	128140349	0.006998	0.73	7	1.28E+08	0.0016	2.04E-08
rs324885	A	C	A	C	0.0142	0.00385056	0.5257	0.708934	5	88601453	0.007442	0.6	5	87897271	0.0014	4.11E-24
rs32940	T	C	T	C	-0.0096	-0.00458109	0.3001	0.299232	5	141752719	0.007507	0.54	5	1.41E+08	0.0015	3.72E-10
rs34098770	A	G	A	G	-0.016	0.000278747	0.152	0.21806	16	12143955	0.008374	0.97	16	12237812	0.002	2.95E-16
rs34155847	A	G	A	G	0.0135	0.00158322	0.2483	0.363112	4	3250403	0.007146	0.82	4	3252130	0.0016	1.43E-16
rs34262657	T	G	T	G	-0.024	-0.000257385	0.9619	0.500961	15	63557088	0.006553	0.97	15	63849287	0.0038	2.84E-10
rs34286836	T	C	T	C	-0.0089	-0.00114532	0.4479	0.409462	6	147952528	0.007146	0.87	6	1.48E+08	0.0014	3.44E-10
rs34298584	A	G	A	G	0.0111	0.000615213	0.1688	0.043228	18	41446731	0.016666	0.97	18	39026695	0.0019	3.67E-09
rs34305371	A	G	A	G	0.0314	-0.045108	0.0985	0.006244	1	72267927	0.043688	0.3	1	72733610	0.0024	1.10E-39
rs34309	A	G	A	G	0.008	-0.000782364	0.3816	0.339577	5	68268555	0.007206	0.91	5	67564383	0.0014	2.94E-08
rs34316274	A	G	A	G	0.0097	0.00860794	0.762	0.675552	16	968697	0.007467	0.25	16	1018697	0.0017	3.86E-09
rs34363861	A	G	A	G	0.0087	0.00682246	0.5194	0.659222	2	73263284	0.007029	0.33	2	73490412	0.0014	8.69E-10
rs34394051	A	G	A	G	-0.0117	0.000417462	0.8429	0.746158	1	6793031	0.007816	0.96	1	6853091	0.002	2.30E-09
rs34410	C	G	C	G	-0.0083	-0.00582368	0.4628	0.228146	5	108133983	0.008005	0.47	5	1.07E+08	0.0014	3.86E-09
rs34485537	A	G	A	G	0.011	0.014879	0.4113	0.536503	16	83575319	0.006748	0.027	16	83608599	0.0014	1.13E-14
rs34624793	T	C	T	C	0.0116	-0.0252213	0.8636	0.989433	2	144159237	0.032263	0.43	2	1.45E+08	0.0021	1.94E-08
rs34720381	T	C	T	C	-0.018	0.0240461	0.0907	0.084054	1	171486183	0.012315	0.051	1	1.71E+08	0.0024	1.90E-13
rs347661	T	C	T	C	-0.0081	0.0192021	0.4347	0.836936	5	63253314	0.008955	0.032	5	62549141	0.0014	1.58E-08
rs34807077	A	C	A	C	0.0128	-0.00161553	0.1569	0.415946	8	28823252	0.006794	0.81	8	28680769	0.0019	3.29E-11
rs34967082	A	G	A	G	-0.008	-0.0115667	0.4161	0.28074	2	214517930	0.007455	0.12	2	2.15E+08	0.0014	1.77E-08
rs35016816	T	C	T	C	0.0176	0.023379	0.899	0.940922	16	10172258	0.014325	0.1	16	10266115	0.0023	3.56E-14
rs35104491	A	G	A	G	0.0124	-0.00348283	0.1849	0.172671	6	96942587	0.008966	0.7	6	97390463	0.0018	6.81E-12
rs35319653	T	C	T	C	0.0119	0.00260565	0.3433	0.270893	4	139982001	0.007677	0.73	4	1.41E+08	0.0015	8.01E-16
rs35417702	T	C	T	C	-0.0152	-0.0162898	0.5235	0.579491	7	72274931	0.007017	0.02	7	71739916	0.0014	2.40E-27
rs35493937	C	G	C	G	0.0124	0.00865998	0.2305	0.080932	2	65344005	0.01233	0.48	2	65571139	0.0017	9.73E-14

rs355579	A	G	A	G	0.0236	-0.000975262	0.9346	0.008646	2	141621829	0.037199	0.98	2	1.42E+08	0.0028	7.71E-17
rs35606437	A	G	A	G	0.0105	0.0105132	0.2675	0.240154	8	92315304	0.00797	0.19	8	93327532	0.0016	3.98E-11
rs356999	A	G	A	G	-0.0101	-0.00743428	0.3802	0.110951	2	60584449	0.010911	0.5	2	60811584	0.0014	2.91E-12
rs35745455	A	G	A	G	0.008	0.00204286	0.501	0.871518	7	5784469	0.009641	0.83	7	5824100	0.0014	1.43E-08
rs35754740	T	C	T	C	0.0083	0.00367297	0.5811	0.408982	16	63093966	0.006909	0.6	16	63127870	0.0014	4.77E-09
rs35929923	A	G	A	G	-0.0114	-0.00806418	0.2495	0.173631	7	21360907	0.008886	0.36	7	21400525	0.0016	1.77E-12
rs36085856	C	G	C	G	0.0201	-0.0120801	0.0734	0.106868	15	70086897	0.010903	0.27	15	70379236	0.0036	1.52E-08
rs36120534	T	C	T	C	0.01	-0.012289	0.1811	0.028578	20	17457476	0.019882	0.54	20	17438121	0.0018	3.77E-08
rs363096	T	C	T	C	-0.0143	-0.00560815	0.4245	0.592939	4	3178294	0.007032	0.43	4	3180021	0.0014	5.77E-24
rs3735478	T	G	T	G	0.0108	0.0120199	0.2932	0.145533	7	44760577	0.009774	0.22	7	44800176	0.0016	3.66E-12
rs3768480	C	G	C	G	0.0101	-0.00659346	0.4523	0.197887	1	109988373	0.008563	0.44	1	1.11E+08	0.0014	8.40E-13
rs3778995	T	C	T	C	0.0191	-0.00541149	0.0683	0.816042	7	2136236	0.008918	0.54	7	2190385	0.0028	7.32E-12
rs3781339	T	C	T	C	-0.0104	0.00331932	0.1962	0.237992	10	103668394	0.008128	0.68	10	1.05E+08	0.0018	7.55E-09
rs3790609	T	C	T	C	0.0107	-0.00711733	0.1744	0.135207	1	112514368	0.010092	0.48	1	1.13E+08	0.0019	8.35E-09
rs3809169	T	C	T	C	0.014	0.00588327	0.9128	0.957493	12	113473727	0.016895	0.73	12	1.14E+08	0.0025	3.85E-08
rs3809634	A	G	A	G	-0.0109	0.0140248	0.6842	0.924832	16	53504245	0.012925	0.28	16	53538157	0.0015	6.82E-13
rs3812281	T	C	T	C	0.0113	-0.00514099	0.6016	0.726225	7	135397999	0.007494	0.49	7	1.35E+08	0.0014	2.70E-15
rs3817923	A	G	A	G	-0.0121	-0.00581669	0.1133	0.041547	1	171840071	0.016047	0.72	1	1.72E+08	0.0022	4.11E-08
rs382196	T	G	T	G	-0.0082	-0.00485142	0.3814	0.345341	3	9103870	0.007296	0.51	3	9145554	0.0014	1.47E-08
rs3847228	T	C	T	C	0.0083	0.0119037	0.406	0.202209	9	1798805	0.008647	0.17	9	1798805	0.0014	7.47E-09
rs3895736	A	C	A	C	0.0144	7.31E-06	0.1728	0.051153	3	48621034	0.015243	1	3	48658467	0.0019	9.92E-15
rs3897821	A	G	A	G	0.0154	0.00271379	0.6654	0.737512	1	243257086	0.007656	0.72	1	2.43E+08	0.0015	3.58E-25
rs399821	A	G	A	G	-0.0078	-0.000462121	0.5321	0.473103	3	21458832	0.006859	0.95	3	21500324	0.0014	2.34E-08
rs39998	A	C	A	C	0.009	0.0299212	0.2967	0.130403	16	28121831	0.010238	0.0035	16	28133152	0.0015	5.02E-09
rs4127499	A	G	A	G	0.0087	0.0176106	0.3495	0.059558	11	122305675	0.013985	0.21	11	1.22E+08	0.0015	3.56E-09
rs42302	A	G	A	G	0.0086	-0.00323378	0.3441	0.626081	5	75669729	0.00708	0.65	5	74965554	0.0015	5.96E-09
rs4255791	A	G	A	G	-0.0084	0.00824193	0.3301	0.09342	16	28263483	0.011792	0.48	16	28274804	0.0015	2.51E-08
rs4257287	T	C	T	C	0.0143	0.00741756	0.0973	0.988233	18	55076600	0.031405	0.81	18	52737309	0.0024	1.52E-09
rs4263475	A	G	A	G	0.0078	-0.00329247	0.5814	0.643852	5	26774033	0.007063	0.64	5	26774142	0.0014	4.40E-08
rs4298514	T	C	T	C	0.0104	-0.0100819	0.6986	0.536023	8	117920648	0.00684	0.14	8	1.19E+08	0.0015	1.07E-11
rs4328757	T	C	T	C	0.0096	-0.00251151	0.6119	0.612632	3	36896689	0.006999	0.72	3	36938180	0.0014	2.95E-11
rs4352658	T	C	T	C	-0.019	-0.00622944	0.081	0.110471	6	87570154	0.010727	0.56	6	88279872	0.0026	1.71E-13
rs4358081	A	C	A	C	-0.0095	0.00835317	0.5285	0.245437	2	28877776	0.007761	0.28	2	29100642	0.0014	1.28E-11
rs4369924	A	G	A	G	0.0125	0.00934735	0.1615	0.302113	20	43325743	0.007488	0.21	20	41954383	0.0019	5.58E-11
rs4384309	A	G	A	G	0.0102	-0.0102471	0.4641	0.536984	10	131312333	0.006775	0.13	10	1.33E+08	0.0014	4.89E-13
rs4396896	A	T	A	T	-0.0107	0.000370249	0.3916	0.558357	3	86212650	0.006899	0.96	3	86261800	0.0014	1.12E-13
rs4423373	A	G	A	G	-0.0087	-0.00416205	0.5386	0.114313	15	27743194	0.010733	0.7	15	27988340	0.0014	6.00E-10
rs4426420	T	C	T	C	-0.014	0.000887204	0.16	0.126321	18	37524423	0.010446	0.93	18	35104386	0.0019	2.63E-13
rs4434676	A	T	A	T	-0.0089	0.00534574	0.4292	0.158501	9	70417560	0.009391	0.57	9	73032476	0.0014	2.83E-10
rs4458044	C	G	C	G	0.0101	-0.0173141	0.2581	0.279299	17	45796361	0.007655	0.024	17	43873727	0.0016	5.71E-10
rs4469771	T	C	T	C	0.0101	-0.00677025	0.6802	0.670989	10	66432956	0.007344	0.36	10	68192714	0.0015	1.88E-11
rs4497562	A	G	A	G	0.0112	0.000774222	0.7295	0.814601	13	62038471	0.008803	0.93	13	62612604	0.0016	1.05E-12
rs4500930	T	C	T	C	-0.0109	0.0104814	0.3449	0.478386	2	228120789	0.00683	0.12	2	2.29E+08	0.0015	1.67E-13
rs4500960	T	C	T	C	-0.0131	0.010344	0.4647	0.429395	2	161962111	0.006769	0.13	2	1.63E+08	0.0014	1.04E-20
rs4502401	T	C	T	C	0.0085	0.00800619	0.6542	0.270893	2	198458802	0.007364	0.28	2	1.99E+08	0.0015	7.55E-09
rs4652135	A	C	A	C	0.0103	-0.00374441	0.7201	0.572046	1	175868537	0.006792	0.58	1	1.76E+08	0.0016	4.35E-11
rs4658019	T	C	T	C	0.0084	0.00501367	0.4531	0.48463	1	196180806	0.006798	0.46	1	1.96E+08	0.0014	2.19E-09
rs4664983	T	C	T	C	-0.0103	0.0109988	0.1987	0.193564	2	158597926	0.008651	0.2	2	1.59E+08	0.0018	4.11E-09
rs4673840	T	C	T	C	-0.0138	-0.00336597	0.842	0.723583	2	214498517	0.00766	0.66	2	2.15E+08	0.0019	5.86E-13
rs4675248	A	G	A	G	-0.0094	-0.000698721	0.3995	0.323727	2	202015507	0.007445	0.93	2	2.03E+08	0.0014	4.50E-11
rs4691601	A	T	A	T	-0.0115	-0.00266302	0.4571	0.318924	4	159678189	0.00724	0.71	4	1.61E+08	0.0014	2.46E-16
rs4705763	C	G	C	G	0.0089	0.00618936	0.5358	0.623199	5	113058806	0.007139	0.39	5	1.12E+08	0.0014	2.08E-10
rs4712371	A	G	A	G	-0.0124	-0.00123039	0.1217	0.152978	6	19227436	0.009436	0.9	6	19227667	0.0021	6.63E-09
rs4719460	T	C	T	C	-0.0088	0.00416586	0.656	0.649616	7	819973	0.007203	0.56	7	859610	0.0015	2.34E-09
rs4724083	T	C	T	C	0.0083	0.00593769	0.3227	0.743036	7	41967473	0.00757	0.43	7	42007071	0.0015	3.53E-08

rs4726070	A	G	A	G	0.0122	0.0105638	0.6021	0.274256	7	151631132	0.007657	0.17	7	1.51E+08	0.0014	1.36E-17
rs4730020	T	C	T	C	0.009	0.00478042	0.2697	0.104467	7	104548635	0.011236	0.67	7	1.04E+08	0.0016	1.21E-08
rs4731413	A	G	A	G	0.0111	0.012455	0.2058	0.044669	7	128196079	0.016629	0.45	7	1.28E+08	0.0017	2.31E-10
rs4739235	A	G	A	G	0.0109	0.00490556	0.1701	0.349183	8	21429594	0.007199	0.5	8	21287105	0.0019	5.26E-09
rs4741571	A	G	A	G	-0.0102	-0.00105517	0.6723	0.82877	9	1667814	0.009078	0.91	9	1667814	0.0015	8.09E-12
rs4778058	T	C	T	C	-0.0092	-0.00115277	0.4964	0.361191	15	92912839	0.007037	0.87	15	93456069	0.0014	4.92E-11
rs4785187	A	G	A	G	-0.01	-0.000247688	0.2253	0.379923	16	49732861	0.007032	0.97	16	49766772	0.0017	3.12E-09
rs4787028	T	C	T	C	0.0086	0.0117823	0.6622	0.614313	16	7483019	0.006974	0.091	16	7533021	0.0015	8.08E-09
rs4787457	A	G	A	G	0.0162	0.0418857	0.6339	0.948847	16	28544079	0.01553	0.007	16	28555400	0.0015	1.23E-28
rs4788115	A	T	A	T	0.0111	-0.0332471	0.1804	0.091979	16	28986790	0.011789	0.0048	16	28998111	0.0019	2.27E-09
rs479018	A	G	A	G	0.0109	0.00704167	0.3255	0.03122	11	66293075	0.018473	0.7	11	66060546	0.0015	3.10E-13
rs4793090	A	G	A	G	0.0084	-0.00179048	0.6635	0.590538	17	42534324	0.007022	0.8	17	40686342	0.0015	1.67E-08
rs481940	T	C	T	C	0.01	-0.00855935	0.2546	0.045629	1	70976774	0.016285	0.6	1	71442457	0.0016	4.94E-10
rs482787	T	C	T	C	0.01	0.00617949	0.6749	0.756724	10	98007267	0.00796	0.44	10	99767024	0.0015	1.95E-11
rs4839155	T	G	T	G	0.0115	-0.0046695	0.7671	0.759846	1	111618867	0.0077	0.54	1	1.12E+08	0.0017	4.66E-12
rs4846724	A	G	A	G	0.0098	-0.00700497	0.5347	0.370557	1	221794475	0.007073	0.32	1	2.22E+08	0.0014	2.89E-12
rs4850954	T	C	T	C	0.0077	0.00976108	0.4628	0.805476	2	100956752	0.00817	0.23	2	1.02E+08	0.0014	3.73E-08
rs4851263	A	G	A	G	-0.0151	0.0115242	0.103	0.23439	2	100185405	0.007941	0.15	2	1.01E+08	0.0023	7.86E-11
rs488476	C	G	C	G	-0.0113	0.00712452	0.6191	0.757925	11	95810551	0.008046	0.38	11	95543715	0.0015	8.29E-15
rs4894658	C	G	C	G	0.0091	0.00616978	0.2619	0.168108	3	174210946	0.008976	0.49	3	1.74E+08	0.0016	1.34E-08
rs4904523	A	G	A	G	-0.0082	0.00620177	0.5208	0.579731	14	89257286	0.007067	0.38	14	89723630	0.0014	6.27E-09
rs4915735	A	G	A	G	0.0116	0.0120089	0.1408	0.051393	1	61342772	0.015269	0.43	1	61808444	0.002	9.56E-09
rs4919624	A	G	A	G	0.0193	0.00666519	0.8022	0.75072	10	102261328	0.008015	0.41	10	1.04E+08	0.0018	6.52E-28
rs4925109	A	G	A	G	-0.0096	-0.0227371	0.3172	0.858549	17	17758488	0.009705	0.019	17	17661802	0.0015	1.91E-10
rs4938815	T	G	T	G	0.0085	-0.0072484	0.7041	0.682757	11	120570938	0.007526	0.34	11	1.2E+08	0.0015	2.99E-08
rs4941735	T	C	T	C	0.0092	-0.0151959	0.5703	0.79731	13	31067111	0.008659	0.079	13	31641248	0.0014	8.63E-11
rs4972748	T	C	T	C	0.0105	-0.0170163	0.8204	0.662104	2	175233282	0.007213	0.018	2	1.76E+08	0.0018	9.50E-09
rs4977885	A	G	A	G	-0.0084	-0.0162198	0.3971	0.769933	9	23687985	0.008005	0.043	9	23687983	0.0014	6.78E-09
rs4984541	A	G	A	G	-0.0138	0.00612099	0.7666	0.541066	15	96367910	0.006751	0.36	15	96911139	0.0017	3.40E-16
rs4984682	C	G	C	G	-0.0138	0.0025333	0.7674	0.32853	16	690404	0.007086	0.72	16	740404	0.0017	1.82E-16
rs503796	C	G	C	T	-0.026	-0.0227743	0.9775	0.005043	2	44921077	0.04864	0.64	2	45157163	0.0047	4.13E-08
rs548897	A	G	A	G	0.0081	-0.00316377	0.45	0.367435	1	57252357	0.007048	0.65	1	57718030	0.0014	1.10E-08
rs55675587	T	C	T	C	-0.0103	0.000377091	0.1926	0.435399	1	65759198	0.006795	0.96	1	66224881	0.0018	5.92E-09
rs55736314	C	G	C	G	-0.0154	-0.00968519	0.599	0.975985	3	71537142	0.02203	0.66	3	71586293	0.0014	4.59E-27
rs56085180	A	G	A	G	-0.0269	-0.0309932	0.0366	0.010567	7	2103959	0.032984	0.35	7	2143594	0.0037	5.94E-13
rs56099375	T	C	T	C	0.012	-0.000545972	0.241	0.017531	8	76460753	0.025377	0.98	8	77372988	0.0017	3.10E-13
rs56171318	T	C	T	C	-0.0137	-0.00318738	0.1431	0.048991	18	37692576	0.015216	0.83	18	35272539	0.002	7.09E-12
rs56174996	A	G	A	G	-0.0143	0.0198438	0.1359	0.068924	1	90679624	0.013641	0.15	1	91145181	0.002	2.81E-12
rs56306882	A	G	A	G	0.0086	-0.0219218	0.2741	0.039385	3	70204542	0.017442	0.21	3	70253693	0.0016	4.82E-08
rs56319902	T	C	T	C	-0.0206	0.0195947	0.2155	0.006724	17	45794616	0.042106	0.64	17	43871982	0.0017	5.81E-33
rs56391344	A	G	A	G	0.0148	0.0134339	0.2526	0.036503	15	77714557	0.01745	0.44	15	78006899	0.0016	7.49E-20
rs563954	A	G	A	G	-0.0081	-0.0170386	0.4639	0.829011	11	132521762	0.00915	0.063	11	1.32E+08	0.0014	1.34E-08
rs56405138	A	T	A	T	0.0153	0.0174961	0.1231	0.066282	4	165205593	0.013388	0.19	4	1.66E+08	0.0022	1.31E-12
rs567003	A	G	A	G	0.0094	-0.0132121	0.3538	0.057637	7	105701161	0.014292	0.36	7	1.05E+08	0.0015	2.27E-10
rs56794817	A	G	A	G	0.0122	-0.00115699	0.1615	0.512728	17	61924617	0.006583	0.86	17	60001978	0.0019	1.69E-10
rs57016874	T	C	T	C	0.0209	0.0371803	0.0387	0.006004	2	219445390	0.04284	0.39	2	2.2E+08	0.0037	1.90E-08
rs57148205	A	G	A	G	0.0095	-0.000861242	0.2438	0.029539	2	224824704	0.020411	0.97	2	2.26E+08	0.0016	5.17E-09
rs57204268	A	G	A	G	0.0114	-0.00719371	0.8497	0.810279	11	133974129	0.008766	0.41	11	1.34E+08	0.002	9.94E-09
rs57349798	A	G	A	G	0.0093	-0.011428	0.4094	0.145773	6	37518276	0.009667	0.24	6	37486052	0.0014	8.63E-11
rs57352738	A	T	A	T	-0.0152	-0.017416	0.204	0.060038	7	133619940	0.014352	0.22	7	1.33E+08	0.0017	2.51E-18
rs57437407	A	G	A	G	-0.0145	-0.010686	0.89	0.965178	3	86124473	0.018663	0.57	3	86173623	0.0023	2.67E-10
rs575113	A	G	A	G	0.0133	0.00695305	0.2941	0.3122	1	109503751	0.007376	0.35	1	1.1E+08	0.0015	7.52E-18
rs5754753	T	C	T	C	-0.0126	-0.016989	0.7178	0.964457	22	33900763	0.016201	0.29	22	34296751	0.0016	6.15E-16
rs5754762	A	G	A	G	0.0197	-0.00665499	0.9405	0.739433	22	33933235	0.007566	0.38	22	34329224	0.003	2.95E-11
rs580652	T	C	T	C	-0.0147	-0.00215896	0.9017	0.695005	11	85193698	0.007509	0.77	11	84904742	0.0024	4.65E-10

rs585557	A	G	A	G	0.0128	-0.00879496	0.8014	0.62464	11	65896076	0.006902	0.2	11	65663547	0.0018	3.25E-13
rs58779949	A	C	A	C	0.011	-0.0190696	0.1698	0.064842	11	64239687	0.013809	0.17	11	64007159	0.0019	3.84E-09
rs58859557	T	C	T	C	0.0187	0.000109095	0.0702	0.149856	1	43544785	0.009517	0.99	1	44010456	0.0028	2.82E-11
rs58996896	A	G	A	G	-0.0094	0.00211385	0.7388	0.824448	2	122977429	0.009046	0.82	2	1.24E+08	0.0016	4.23E-09
rs590013	T	C	T	C	0.01	0.0123643	0.6772	0.793228	1	28829226	0.008156	0.13	1	29155738	0.0015	2.47E-11
rs59123361	C	G	A	G	0.0203	-0.0251342	0.9006	0.027137	1	110223832	0.021028	0.23	1	1.11E+08	0.0024	7.78E-18
rs59300999	T	C	T	C	-0.0164	0.00616747	0.9325	0.491835	21	31700050	0.006778	0.36	21	33072363	0.0028	4.85E-09
rs59480703	C	G	C	G	-0.0123	-0.00238965	0.1936	0.365994	8	9795620	0.007187	0.74	8	9653130	0.0018	3.97E-12
rs59718180	A	C	G	A	0.0083	-0.0213444	0.3496	0.115034	2	139690168	0.010866	0.049	2	1.4E+08	0.0015	2.94E-08
rs59813324	T	G	T	G	0.0097	0.0078774	0.1983	0.346302	12	60485892	0.007199	0.27	12	60879673	0.0018	4.15E-08
rs60096640	A	G	A	G	0.0159	-0.00163646	0.8926	0.621037	6	27668534	0.007019	0.82	6	27636313	0.0023	2.24E-12
rs6020560	T	C	T	C	-0.0083	0.0012906	0.5258	0.662104	20	50502882	0.007262	0.86	20	49119419	0.0014	3.84E-09
rs60589532	A	G	A	G	0.0168	0.00377812	0.9345	0.865994	2	57845470	0.009817	0.7	2	58072605	0.0028	3.03E-09
rs6060308	A	G	A	G	0.0095	-0.00952566	0.2788	0.15562	20	35206575	0.00928	0.3	20	33794378	0.0016	1.76E-09
rs6065080	T	C	T	C	-0.013	-0.0108976	0.3589	0.455091	20	61257735	0.006823	0.11	20	59832791	0.0015	7.49E-19
rs6065784	C	G	C	G	0.011	-0.0144432	0.6953	0.969501	20	45088794	0.019331	0.45	20	43717435	0.0015	5.78E-13
rs6091570	A	G	A	G	0.0081	-0.00695349	0.3491	0.247358	20	52683345	0.007787	0.37	20	51299884	0.0015	3.20E-08
rs61104616	A	G	A	G	-0.0172	0.0163922	0.5249	0.528338	5	88867954	0.006928	0.018	5	88163771	0.0014	1.56E-34
rs61387839	A	G	A	G	0.0088	-0.0308662	0.695	0.98463	2	48461496	0.02767	0.26	2	48688635	0.0015	1.11E-08
rs61739710	A	G	A	G	-0.0099	-0.00896046	0.2813	0.157061	21	33553383	0.009449	0.34	21	34925689	0.0016	3.74E-10
rs61755388	T	C	T	C	0.0125	-0.0452538	0.7878	0.965658	7	126901263	0.018535	0.015	7	1.27E+08	0.0017	3.88E-13
rs61798586	A	C	A	C	-0.0112	0.00797723	0.1444	0.115034	4	36690073	0.01059	0.45	4	36691695	0.002	2.05E-08
rs61853335	T	C	T	C	-0.0109	0.000581103	0.1769	0.35879	10	63124977	0.007157	0.94	10	64884737	0.0018	3.29E-09
rs618869	T	G	A	G	-0.0163	-0.010495	0.8285	0.016811	18	55580920	0.025836	0.68	18	53210302	0.0019	1.95E-18
rs61958175	A	G	A	G	-0.0198	-0.00791251	0.9538	0.962536	13	58106116	0.018164	0.66	13	58680250	0.0034	8.22E-09
rs62051146	A	C	A	C	-0.0134	-0.00162395	0.8886	0.794909	16	72463822	0.008269	0.84	16	72497721	0.0022	1.79E-09
rs62090515	A	G	A	G	-0.009	0.0146864	0.3605	0.157541	18	45091671	0.009376	0.12	18	42671636	0.0015	6.88E-10
rs62103236	T	C	T	C	-0.0147	0.0114765	0.9087	0.013689	18	79880561	0.029799	0.7	18	77634276	0.0025	2.69E-09
rs62109862	A	G	A	G	0.0136	-0.00111688	0.1418	0.574928	19	12900995	0.006786	0.87	19	13011809	0.002	2.85E-11
rs62124721	T	G	T	C	-0.0127	-0.00354378	0.1216	0.012488	2	12687624	0.029953	0.91	2	12836640	0.0021	3.16E-09
rs62142891	A	G	A	G	0.0108	0.0155815	0.2632	0.016811	2	51311975	0.025475	0.54	2	51539113	0.0016	1.05E-11
rs62155770	A	G	A	G	-0.0196	-0.0140531	0.9474	0.993276	2	99364759	0.0421	0.74	2	99981222	0.0032	6.88E-10
rs62155873	T	C	T	C	-0.0151	0.0131717	0.1221	0.028338	2	105352905	0.020617	0.52	2	1.06E+08	0.0021	1.65E-12
rs62174974	A	G	A	G	-0.0105	-0.00774443	0.1949	0.337896	2	184188658	0.007255	0.29	2	1.85E+08	0.0018	2.76E-09
rs62179650	A	G	A	G	0.0112	-0.0194507	0.2995	0.086936	2	188271157	0.011739	0.098	2	1.89E+08	0.0016	5.66E-13
rs62190914	T	C	T	C	0.0089	-6.99E-05	0.636	0.602546	2	235879531	0.006853	0.99	2	2.37E+08	0.0015	1.25E-09
rs62194170	A	G	A	G	0.0111	-0.0119576	0.2025	0.436359	2	161009993	0.006957	0.086	2	1.62E+08	0.0017	2.41E-10
rs622169	T	C	T	C	0.0086	0.00274204	0.4536	0.056196	1	244274105	0.013453	0.84	1	2.44E+08	0.0014	2.73E-09
rs62247449	C	G	C	G	0.0114	0.00386503	0.4357	0.178915	3	64444875	0.008804	0.66	3	64430551	0.0014	8.79E-16
rs62252819	A	T	A	T	-0.0094	0.00803589	0.7522	0.922911	3	53406101	0.012551	0.52	3	53440128	0.0017	1.20E-08
rs62256284	T	C	T	C	0.0111	-0.00229963	0.2093	0.314841	3	70477154	0.007405	0.76	3	70526305	0.0017	1.32E-10
rs62260764	C	G	C	G	-0.0132	-0.00444388	0.2559	0.147935	3	47949010	0.009497	0.64	3	47990500	0.0016	3.45E-16
rs62262671	A	G	A	G	0.0213	0.00779144	0.8634	0.993036	3	49612440	0.0414	0.85	3	49649873	0.002	2.22E-25
rs62370510	A	T	A	T	-0.0109	0.0193831	0.1771	0.017531	5	53493368	0.026372	0.46	5	52789198	0.0019	5.59E-09
rs62379838	T	C	T	C	0.0121	0.00581268	0.6953	0.85975	5	120766333	0.009968	0.56	5	1.2E+08	0.0015	1.77E-15
rs62409395	T	C	T	C	0.0101	0.000766395	0.7755	0.763929	4	25603414	0.007901	0.92	4	25605036	0.0018	3.55E-08
rs62506074	T	C	T	C	0.0091	0.00447938	0.343	0.283381	8	31048573	0.007553	0.55	8	30906089	0.0015	8.64E-10
rs62506104	A	G	A	G	-0.0098	0.0170965	0.1923	0.111431	8	31162492	0.010871	0.12	8	31020008	0.0018	3.93E-08
rs628993	A	G	A	G	0.0128	-0.00420952	0.1073	0.325889	11	61772219	0.007195	0.56	11	61539691	0.0023	3.83E-08
rs633279	A	T	A	T	-0.0089	-0.0130046	0.2957	0.258165	6	93130987	0.007878	0.099	6	93840705	0.0015	8.12E-09
rs635754	A	G	A	G	0.0119	-0.017629	0.5809	0.478626	1	96019384	0.006894	0.011	1	96484940	0.0014	6.36E-17
rs6428587	T	C	T	C	0.0084	-0.0206053	0.6323	0.731268	1	90359630	0.007812	0.0083	1	90825188	0.0015	8.59E-09
rs6435326	A	T	A	T	-0.0091	0.00406845	0.5148	0.710855	2	206137835	0.007623	0.59	2	2.07E+08	0.0014	7.56E-11
rs6449503	A	G	A	G	0.0185	0.00261655	0.5038	0.844861	5	60799445	0.008814	0.77	5	60095272	0.0014	1.84E-39
rs6452793	T	G	T	G	0.0175	-0.00794881	0.234	0.036744	5	88522356	0.016847	0.64	5	87818174	0.0017	4.47E-26

rs6461536	A	G	A	G	0.0088	0.00673389	0.7118	0.622959	7	21092188	0.006938	0.33	7	21131807	0.0015	1.28E-08
rs6469654	C	G	C	G	-0.0103	-0.00415766	0.7769	0.537704	8	116620726	0.006825	0.54	8	1.18E+08	0.0017	1.10E-09
rs6480234	T	C	T	C	-0.0084	0.00199117	0.525	0.847022	10	66877136	0.009418	0.83	10	68636894	0.0014	1.94E-09
rs6490618	T	C	T	C	0.0088	0.00521264	0.327	0.772334	13	20764204	0.008048	0.52	13	21338343	0.0015	3.48E-09
rs6493265	T	C	T	C	-0.0122	-0.00559293	0.3915	0.116955	15	47221056	0.010563	0.6	15	47513253	0.0014	2.26E-17
rs6493275	A	T	A	T	0.0131	0.0148808	0.7885	0.961335	15	47384382	0.01781	0.4	15	47676579	0.0017	2.98E-14
rs6567288	A	G	A	G	-0.0079	-0.0124833	0.5719	0.251201	18	62551101	0.007732	0.11	18	60218334	0.0014	2.81E-08
rs6569077	T	C	T	C	0.0118	0.00546433	0.3894	0.571326	6	97764533	0.006815	0.42	6	98212409	0.0014	3.29E-16
rs6573552	T	C	T	C	-0.009	-0.0092663	0.496	0.770413	14	64283515	0.008089	0.25	14	64750233	0.0014	1.17E-10
rs6573559	T	G	T	G	0.0105	0.0138999	0.3152	0.396494	14	64456448	0.006933	0.045	14	64923166	0.0015	3.11E-12
rs660001	A	G	A	G	-0.0164	0.00523481	0.2113	0.227906	5	114530901	0.00818	0.52	5	1.14E+08	0.0017	1.66E-21
rs66482320	C	G	C	G	0.0124	-0.00206444	0.888	0.899376	21	41317004	0.011232	0.85	21	42688931	0.0022	2.57E-08
rs66568921	T	G	T	G	-0.0181	-0.00350071	0.6441	0.466859	3	85622868	0.006735	0.6	3	85672018	0.0015	7.60E-34
rs66721975	A	G	A	G	-0.0087	0.0100311	0.2903	0.135927	2	190836459	0.009613	0.3	2	1.92E+08	0.0015	1.51E-08
rs6690195	T	C	T	C	-0.0113	-0.0305243	0.4907	0.036503	1	95756097	0.017682	0.084	1	96221653	0.0014	7.89E-16
rs6695132	T	C	T	C	0.0097	0.00431085	0.2216	0.727906	1	234598909	0.007654	0.57	1	2.35E+08	0.0017	1.01E-08
rs6696068	T	G	T	G	-0.0088	-0.00643524	0.3838	0.580692	1	53275125	0.006723	0.34	1	53740797	0.0014	9.50E-10
rs6697584	T	C	T	C	-0.0126	0.00578284	0.7852	0.922671	1	181540582	0.012901	0.65	1	1.82E+08	0.0017	1.43E-13
rs6704768	A	G	A	G	-0.0116	0.00901615	0.5648	0.48415	2	232727791	0.006928	0.19	2	2.34E+08	0.0014	1.84E-16
rs6706275	T	C	T	C	0.0083	0.00602314	0.3246	0.063641	2	239343922	0.013981	0.67	2	2.4E+08	0.0015	2.81E-08
rs6711399	T	C	T	C	-0.0107	-0.00077362	0.1779	0.450048	2	199598117	0.006752	0.91	2	2E+08	0.0019	1.16E-08
rs6715321	T	C	T	C	-0.0097	-0.00525353	0.4277	0.317483	2	99492539	0.007508	0.48	2	1E+08	0.0014	7.62E-12
rs6715849	A	G	A	G	-0.0122	0.0120684	0.4401	0.236792	2	99689916	0.008113	0.14	2	1E+08	0.0014	6.87E-18
rs6720515	A	T	A	T	0.0103	-0.0112968	0.7763	0.614073	2	155747023	0.007044	0.11	2	1.57E+08	0.0017	7.95E-10
rs67224963	A	G	A	G	0.0113	0.004684	0.2052	0.65562	5	64491983	0.006955	0.5	5	63787810	0.0017	7.19E-11
rs6729612	T	C	T	C	0.0092	0.00526703	0.676	0.215418	2	151923653	0.008155	0.52	2	1.53E+08	0.0015	9.39E-10
rs6731373	A	G	A	G	-0.0115	0.00153345	0.339	0.147935	2	68275912	0.009508	0.87	2	68503044	0.0015	1.39E-14
rs6736025	T	G	T	G	0.0079	-0.00176252	0.4354	0.57853	2	58570499	0.006944	0.8	2	58797634	0.0014	2.77E-08
rs6738860	A	T	A	T	-0.0088	-0.00857325	0.4574	0.880884	2	22219227	0.010282	0.4	2	22442099	0.0014	4.65E-10
rs67456868	A	G	A	G	0.0119	0.0131729	0.1839	0.038665	6	118758637	0.018034	0.47	6	1.19E+08	0.0018	5.37E-11
rs6752228	T	G	T	G	-0.008	-0.00844669	0.5523	0.449328	2	160691186	0.006872	0.22	2	1.62E+08	0.0014	1.26E-08
rs6774533	T	C	T	C	0.009	0.00778315	0.6985	0.680355	3	62485411	0.007347	0.29	3	62471086	0.0015	4.18E-09
rs67790232	A	G	A	G	0.0101	-0.0142128	0.794	0.882325	12	45685389	0.010625	0.18	12	46079172	0.0017	6.90E-09
rs6780414	T	C	T	C	0.0105	-0.014804	0.8176	0.839337	3	67334246	0.00929	0.11	3	67384670	0.0018	7.10E-09
rs68044584	T	C	T	C	0.0113	0.0157124	0.3063	0.977906	3	108126548	0.022469	0.48	3	1.08E+08	0.0015	1.02E-13
rs6812533	T	C	T	C	0.0106	0.0129558	0.7444	0.709414	4	39686218	0.007615	0.089	4	39687838	0.0016	4.85E-11
rs68145588	T	G	T	G	-0.0127	-0.0151414	0.1395	0.086455	17	77868723	0.012316	0.22	17	75864805	0.0021	7.53E-10
rs6824567	T	C	T	C	0.013	0.000976432	0.2827	0.047791	4	2881445	0.015747	0.95	4	2883172	0.0016	8.91E-17
rs6853599	T	C	T	C	0.0108	0.027618	0.1787	0.014169	4	3443883	0.02874	0.34	4	3445610	0.0019	6.27E-09
rs6861925	C	G	C	G	-0.009	-0.0103148	0.4902	0.418588	5	176897728	0.006743	0.13	5	1.76E+08	0.0014	1.76E-10
rs6871635	A	G	A	G	-0.0087	0.0192188	0.43	0.278098	5	134494704	0.00763	0.012	5	1.34E+08	0.0014	1.16E-09
rs6881581	A	G	A	G	-0.0096	0.000941919	0.3278	0.196926	5	141566788	0.008457	0.91	5	1.41E+08	0.0015	1.08E-10
rs6881733	T	C	T	C	-0.0086	-0.00478154	0.287	0.366715	5	93251285	0.007067	0.5	5	92586991	0.0016	4.08E-08
rs6917154	T	C	T	C	0.0127	-0.00826646	0.1236	0.048511	6	166721668	0.015789	0.6	6	1.67E+08	0.0021	2.55E-09
rs6917204	T	C	T	C	0.0119	-0.00291669	0.7951	0.456772	6	144963580	0.006937	0.67	6	1.45E+08	0.0017	8.14E-12
rs6918506	C	G	C	G	0.0113	0.00651675	0.5379	0.290826	6	26577629	0.007508	0.39	6	26577857	0.0014	9.57E-16
rs6924023	A	G	A	G	0.0107	-0.00356864	0.7548	0.085975	6	169675945	0.011337	0.75	6	1.7E+08	0.0016	5.17E-11
rs6932108	C	G	C	G	-0.021	-0.00720724	0.0798	0.011287	6	98266116	0.032654	0.83	6	98713992	0.0027	6.10E-15
rs6946136	A	G	A	G	0.0081	-0.00714866	0.6116	0.669549	7	135738676	0.007302	0.33	7	1.35E+08	0.0014	1.99E-08
rs6946362	T	C	T	C	0.0091	-0.00172712	0.6884	0.451969	7	6535176	0.00698	0.8	7	6574807	0.0015	1.89E-09
rs6951996	A	T	A	T	-0.0182	-0.0119668	0.0522	0.035303	7	97017125	0.018057	0.51	7	96646437	0.0032	1.41E-08
rs6969783	A	T	A	T	-0.0098	-0.00999151	0.4705	0.317243	7	117953254	0.007295	0.17	7	1.18E+08	0.0014	2.87E-12
rs6989141	A	G	A	G	0.01	-0.017499	0.208	0.019933	8	141671360	0.024481	0.47	8	1.43E+08	0.0017	8.40E-09
rs7012546	T	C	T	C	0.0092	-0.0029104	0.4166	0.185399	8	104055509	0.008708	0.74	8	1.05E+08	0.0014	1.25E-10
rs7016302	C	G	C	G	-0.0125	-0.000571311	0.8308	0.897454	8	4975519	0.011077	0.96	8	4833041	0.0019	2.26E-11

rs702606	T	C	T	C	0.0118	-0.00135488	0.8654	0.875841	5	53871287	0.010607	0.9	5	53167117	0.0021	9.19E-09
rs7029718	A	G	A	G	0.0244	-0.00451958	0.4108	0.469981	9	23358497	0.006803	0.51	9	23358495	0.0014	7.82E-65
rs7030373	A	G	A	G	-0.0098	0.0100742	0.2074	0.439481	9	118399648	0.007033	0.15	9	1.21E+08	0.0017	1.49E-08
rs7035315	A	G	A	G	-0.009	-0.000354434	0.6292	0.601585	9	80616596	0.007014	0.96	9	83231511	0.0015	5.85E-10
rs7041702	A	G	A	G	-0.0111	0.00621766	0.7406	0.869356	9	33053432	0.010272	0.54	9	33053430	0.0016	4.53E-12
rs7084508	T	C	T	C	-0.0085	0.00613646	0.3479	0.267291	10	132119442	0.00758	0.42	10	1.34E+08	0.0015	8.79E-09
rs711793	T	C	T	C	0.0103	-0.0154143	0.3474	0.292747	2	173311193	0.007489	0.04	2	1.74E+08	0.0015	5.01E-12
rs7146625	A	G	A	G	0.0091	-0.00668284	0.7313	0.65658	14	84321809	0.007128	0.35	14	84788153	0.0016	7.55E-09
rs7147473	A	G	A	G	-0.0103	-0.0127411	0.6719	0.981748	14	30139332	0.024269	0.6	14	30608538	0.0015	6.33E-12
rs7158218	A	C	A	C	-0.009	-0.00232522	0.7117	0.853987	14	73468175	0.009852	0.81	14	73934880	0.0015	5.63E-09
rs71646142	T	C	T	C	0.0104	-0.0168559	0.1893	0.024015	1	212195886	0.02099	0.42	1	2.12E+08	0.0018	5.69E-09
rs716513	A	T	A	T	0.0084	-0.000574834	0.536	0.192603	13	68914776	0.008658	0.95	13	69488908	0.0014	2.83E-09
rs7167688	T	C	T	C	-0.0094	-0.00417725	0.4917	0.194765	15	26567990	0.008704	0.63	15	26813137	0.0014	1.71E-11
rs7171405	A	G	A	G	-0.0101	0.00225606	0.2432	0.189481	15	61174694	0.008734	0.8	15	61466893	0.0016	7.53E-10
rs717996	T	C	T	C	-0.0122	-0.00393361	0.5986	0.747118	12	83740041	0.007954	0.62	12	84133820	0.0014	1.89E-17
rs7190	A	G	A	G	-0.0095	0.00478335	0.3514	0.772334	18	5889766	0.008144	0.56	18	5889765	0.0015	1.50E-10
rs7196496	T	G	C	T	0.0177	-0.00277417	0.0528	0.027618	16	66718992	0.020852	0.89	16	66750040	0.0031	1.58E-08
rs721579	T	C	T	C	-0.011	0.00269995	0.2617	0.473343	17	45293115	0.006837	0.69	17	43370481	0.0016	5.96E-12
rs7218235	A	G	A	G	-0.0097	0.00839213	0.2135	0.179875	17	2660973	0.008922	0.35	17	2564267	0.0017	2.44E-08
rs7223311	C	G	C	G	-0.0079	0.00201233	0.5169	0.544669	17	80966814	0.006839	0.77	17	78940614	0.0014	1.77E-08
rs7226824	T	C	T	C	-0.0078	-0.000402616	0.5305	0.48439	18	30096862	0.006866	0.95	18	27676827	0.0014	2.72E-08
rs72482130	T	C	T	C	0.0141	-0.00184053	0.8464	0.769452	2	236145712	0.008255	0.82	2	2.37E+08	0.0019	4.59E-13
rs7254263	T	C	T	C	-0.0119	-0.00136001	0.285	0.428194	19	30250350	0.006952	0.84	19	30741257	0.0016	1.84E-14
rs7255223	A	C	A	C	0.0097	0.00174643	0.2691	0.177954	19	32333404	0.008953	0.85	19	32824310	0.0016	1.06E-09
rs72622559	T	C	T	C	-0.012	-0.00776357	0.2301	0.3122	3	175955109	0.007349	0.29	3	1.76E+08	0.0017	5.50E-13
rs72636697	T	C	T	C	-0.014	-0.00308317	0.1551	0.090298	4	67150891	0.011895	0.8	4	68016609	0.002	1.31E-12
rs72667460	T	C	T	C	0.0197	0.0476006	0.0539	0.005043	1	66070329	0.048548	0.33	1	66536012	0.0031	2.44E-10
rs72671456	A	G	A	G	-0.0174	0.0322052	0.1374	0.020173	14	29193531	0.024077	0.18	14	29662737	0.002	1.27E-17
rs72672052	A	T	A	T	-0.0101	-0.00889893	0.1753	0.139529	8	114786195	0.009684	0.36	8	1.16E+08	0.0018	4.87E-08
rs72677177	A	G	A	G	0.01	-0.00108505	0.4017	0.382325	1	71654351	0.006987	0.88	1	72120034	0.0014	3.03E-12
rs72792395	T	C	T	C	-0.0145	0.017875	0.1093	0.096302	2	44659005	0.011708	0.13	2	44886144	0.0022	1.09E-10
rs728054	A	G	A	G	-0.0128	0.0134395	0.3561	0.318444	7	137387618	0.007469	0.072	7	1.37E+08	0.0015	2.34E-18
rs72819118	T	C	T	C	-0.0231	-0.0273284	0.0942	0.012248	2	99834895	0.031399	0.38	2	1E+08	0.0024	8.29E-22
rs72824753	T	C	T	C	0.0133	-0.00446445	0.9005	0.82829	6	14618917	0.0091	0.62	6	14619148	0.0024	1.93E-08
rs72828517	T	C	T	C	-0.0168	-0.00807434	0.8273	0.825648	6	19035804	0.009063	0.37	6	19036035	0.0019	1.38E-19
rs72834698	A	G	A	G	0.0117	-0.0145222	0.144	0.01537	6	26176289	0.028131	0.61	6	26176517	0.002	4.91E-09
rs72881110	A	T	A	T	0.0146	0.00173309	0.0753	0.028578	2	44526881	0.020202	0.93	2	44754020	0.0027	3.59E-08
rs72883760	A	G	A	G	0.0101	0.00262191	0.1985	0.138569	11	29310422	0.009642	0.79	11	29331969	0.0018	8.64E-09
rs72894681	T	G	T	G	0.0162	0.00934529	0.0707	0.951009	2	182643020	0.016141	0.56	2	1.84E+08	0.0028	4.88E-09
rs72919450	T	C	T	C	-0.0132	0.00747086	0.0997	0.051873	2	81772444	0.015079	0.62	2	81999568	0.0023	1.67E-08
rs72944064	T	C	T	C	-0.0098	-0.0208208	0.7437	0.958213	18	65849019	0.016813	0.22	18	63516255	0.0016	9.39E-10
rs72962169	T	C	T	C	-0.0174	-0.0234268	0.1595	0.072526	11	72654625	0.012987	0.071	11	72365669	0.0019	1.26E-19
rs72993796	T	C	T	C	0.0127	-0.0356228	0.8836	0.98391	2	239399357	0.025936	0.17	2	2.4E+08	0.0022	1.12E-08
rs73034295	A	G	A	G	0.0099	-0.023023	0.2016	0.043468	11	133952238	0.016715	0.17	11	1.34E+08	0.0018	2.11E-08
rs730384	A	G	A	G	0.0101	0.00141298	0.4388	0.219741	14	74423167	0.008218	0.86	14	74889870	0.0014	9.38E-13
rs73039077	C	G	C	G	0.0092	-0.0146955	0.2471	0.018252	3	16994895	0.024563	0.55	3	17036387	0.0016	1.75E-08
rs73046392	A	G	A	G	0.0175	-0.0295996	0.9446	0.008646	7	1884597	0.037248	0.43	7	1944591	0.0032	3.79E-08
rs73055556	A	G	A	G	0.0111	0.00492851	0.1385	0.380884	3	27986047	0.006981	0.48	3	28027538	0.002	4.95E-08
rs7321274	A	G	A	G	0.0114	0.00793808	0.7999	0.792027	13	68572054	0.00845	0.35	13	69146186	0.0018	8.21E-11
rs7323027	A	G	A	G	0.013	0.00324005	0.3719	0.257685	13	58119656	0.007873	0.68	13	58693790	0.0015	3.91E-19
rs73344830	A	G	A	G	0.017	-0.00284305	0.4181	0.288184	10	102057071	0.007387	0.7	10	1.04E+08	0.0014	1.00E-32
rs73366608	A	G	G	C	0.0388	-0.00791873	0.0181	0.04707	8	142261769	0.016369	0.63	8	1.43E+08	0.0053	2.56E-13
rs73405293	A	G	A	G	0.0113	-0.00315606	0.1516	0.168588	12	117085112	0.009173	0.73	12	1.18E+08	0.002	7.42E-09
rs73581580	A	G	A	G	-0.0181	-0.0525397	0.1248	0.018492	9	137357006	0.02572	0.041	9	1.4E+08	0.0022	5.78E-16
rs73643713	T	C	T	C	-0.0165	-0.004922	0.928	0.854707	9	4163612	0.009661	0.61	9	4163612	0.0027	1.24E-09

rs736471	T	C	T	C	0.0083	0.00849196	0.4653	0.127281	3	50481961	0.009885	0.39	3	50519392	0.0014	4.69E-09
rs73648455	T	C	T	C	-0.0183	-0.000768943	0.0797	0.075889	9	14077379	0.013047	0.95	9	14077378	0.0026	1.99E-12
rs73874335	T	C	T	C	-0.0178	0.00532307	0.0607	0.116955	3	158215694	0.010549	0.61	3	1.58E+08	0.0029	1.29E-09
rs73961845	A	G	A	G	-0.0114	0.000400515	0.617	0.857109	2	143721414	0.009588	0.97	2	1.44E+08	0.0014	2.52E-15
rs74459683	A	G	C	A	-0.0147	0.00125442	0.9026	0.013449	12	122612067	0.028491	0.96	12	1.23E+08	0.0024	5.71E-10
rs74462621	T	C	T	C	0.0125	0.0225816	0.1357	0.063881	8	132720610	0.014237	0.11	8	1.34E+08	0.002	1.21E-09
rs7449561	A	G	A	G	0.0102	0.00740351	0.226	0.26633	6	65505656	0.007844	0.35	6	66215549	0.0017	9.73E-10
rs7451726	A	G	A	G	0.0109	-0.00247276	0.7947	0.748559	6	28414717	0.007886	0.75	6	28382494	0.0017	2.86E-10
rs7460106	T	C	T	C	-0.0123	0.0072711	0.762	0.444524	8	142453416	0.006908	0.29	8	1.44E+08	0.0017	2.06E-13
rs74615093	A	G	A	G	0.0145	0.00543741	0.0772	0.024256	16	69088140	0.022118	0.81	16	69122043	0.0026	3.67E-08
rs74915041	A	G	A	G	0.0241	0.0333073	0.966	0.018972	9	118439186	0.024176	0.17	9	1.21E+08	0.0039	4.52E-10
rs74944857	T	G	T	G	-0.0089	0.000567941	0.3223	0.028338	4	65631196	0.019067	0.98	4	66496914	0.0015	3.62E-09
rs7495033	A	T	A	T	0.0095	0.0146642	0.689	0.831412	15	74913884	0.009147	0.11	15	75206225	0.0015	5.92E-10
rs7561705	A	G	A	G	0.0078	-0.00234558	0.472	0.404899	2	142688903	0.006894	0.73	2	1.43E+08	0.0014	2.71E-08
rs7575938	A	G	A	G	0.009	0.000708241	0.3408	0.577089	2	166054167	0.006825	0.92	2	1.67E+08	0.0015	1.13E-09
rs758161	C	G	C	T	0.0142	-0.0793379	0.1237	0.009126	12	1857461	0.03624	0.029	12	1954096	0.0021	2.47E-11
rs7590368	T	C	T	C	-0.0146	-0.00394262	0.7335	0.902257	2	10821348	0.011422	0.73	2	10961474	0.0016	3.98E-20
rs7603132	A	G	A	G	0.0137	0.00419089	0.1938	0.139529	2	4903958	0.00981	0.67	2	4951548	0.0018	1.20E-14
rs76076331	T	C	T	C	0.0206	0.0138833	0.1346	0.024015	2	10837459	0.021376	0.52	2	10977585	0.0021	1.71E-23
rs76077165	A	C	A	C	0.0165	-0.00366657	0.0898	0.125841	2	162036828	0.010384	0.72	2	1.63E+08	0.0025	2.62E-11
rs76241605	A	G	A	G	0.0139	0.0180548	0.1142	0.013209	4	139796006	0.029696	0.54	4	1.41E+08	0.0022	6.48E-10
rs76246107	A	G	A	G	-0.0162	-0.000429571	0.0941	0.263929	19	49618017	0.007688	0.96	19	50121274	0.0026	6.52E-10
rs7625428	T	C	T	C	0.0098	0.00158246	0.6036	0.097743	3	56536879	0.010956	0.89	3	56570907	0.0014	1.07E-11
rs7630133	A	G	A	G	0.0082	-0.00149056	0.6553	0.46854	3	124204441	0.006875	0.83	3	1.24E+08	0.0015	2.80E-08
rs763553	A	G	A	G	0.0087	0.00679436	0.4452	0.783141	8	31587980	0.008161	0.41	8	31445496	0.0014	6.41E-10
rs7650602	T	C	T	C	-0.0089	0.0105408	0.5599	0.698367	3	141428572	0.007494	0.16	3	1.41E+08	0.0014	2.77E-10
rs76552497	T	C	T	C	-0.012	0.0102928	0.1704	0.162344	8	12822171	0.00932	0.27	8	12679680	0.0019	1.17E-10
rs7672622	A	G	A	G	-0.0088	0.000198031	0.7459	0.716859	4	156784399	0.0075	0.98	4	1.58E+08	0.0016	4.72E-08
rs767943	A	C	A	C	-0.0122	-0.013237	0.269	0.733429	6	23446463	0.007598	0.081	6	23446691	0.0016	1.54E-14
rs7683416	T	C	T	C	0.0139	0.00981642	0.4592	0.768732	4	105231827	0.007992	0.22	4	1.06E+08	0.0014	6.05E-23
rs7692359	T	C	T	C	-0.0109	-0.0144999	0.7786	0.958213	4	82288193	0.017012	0.39	4	83209346	0.0017	1.40E-10
rs77025239	A	G	A	G	-0.014	-0.00446759	0.1559	0.207253	3	181016397	0.00841	0.6	3	1.81E+08	0.0019	4.65E-13
rs7714719	C	G	C	G	0.0116	-0.021442	0.8602	0.948127	5	107163226	0.015213	0.16	5	1.06E+08	0.002	1.12E-08
rs7766240	T	C	T	C	-0.0134	0.00806428	0.0964	0.080452	6	16954137	0.012546	0.52	6	16954368	0.0024	2.00E-08
rs7772172	A	G	A	G	0.0091	-0.00932803	0.4009	0.146254	6	16662697	0.009622	0.33	6	16662928	0.0014	1.74E-10
rs77826402	T	C	T	C	0.0151	0.0121582	0.073	0.120317	2	99634927	0.010647	0.25	2	1E+08	0.0027	2.05E-08
rs7796103	C	G	C	G	-0.0077	-0.00680951	0.5168	0.350144	7	41780557	0.007195	0.34	7	41820155	0.0014	3.81E-08
rs7799141	A	G	A	G	0.0099	-0.0039839	0.3104	0.318684	7	132975729	0.007457	0.59	7	1.33E+08	0.0015	5.31E-11
rs7803932	A	G	A	G	0.0127	0.00878361	0.1667	0.036023	7	70738687	0.018371	0.63	7	70203673	0.0019	1.74E-11
rs78116078	C	G	C	G	0.0086	-0.0162642	0.718	0.936359	1	18107631	0.013957	0.24	1	18434125	0.0016	3.33E-08
rs78193153	A	G	A	G	0.0191	0.00801505	0.0647	0.379443	7	72255696	0.007079	0.26	7	71720681	0.0029	2.76E-11
rs7849480	A	G	A	G	-0.0084	-0.0139042	0.4353	0.228866	9	14648132	0.007863	0.077	9	14648130	0.0014	3.54E-09
rs7855503	C	G	C	G	0.0088	0.0011205	0.3478	0.492075	9	83766723	0.006891	0.87	9	86381638	0.0015	2.39E-09
rs78648104	T	C	T	C	-0.0141	0.00102321	0.9137	0.814121	6	50715296	0.009009	0.91	6	50683009	0.0025	1.70E-08
rs78714229	T	C	T	C	0.0172	-0.0124546	0.9436	0.990394	6	25792827	0.035345	0.72	6	25793055	0.003	1.55E-08
rs7875078	A	C	A	C	-0.0078	-0.000350856	0.4564	0.591739	9	14494847	0.00701	0.96	9	14494845	0.0014	2.72E-08
rs78918150	T	C	T	C	-0.008	0.00598272	0.6227	0.670269	2	116853949	0.007279	0.41	2	1.18E+08	0.0015	4.26E-08
rs7894722	T	C	T	C	0.0085	0.00400321	0.3748	0.371998	10	9930083	0.007037	0.57	10	9972046	0.0014	5.41E-09
rs7899270	A	C	A	C	-0.0084	-0.00868731	0.6524	0.692603	10	109498376	0.007462	0.24	10	1.11E+08	0.0015	1.74E-08
rs7905192	T	C	T	C	0.0084	-0.00244196	0.572	0.652978	10	12669950	0.007209	0.73	10	12711949	0.0014	3.14E-09
rs790647	A	C	A	C	-0.0145	-0.00337746	0.2278	0.313881	10	105016726	0.007466	0.65	10	1.07E+08	0.0017	3.81E-18
rs7920624	A	T	A	T	0.012	-0.00724257	0.4783	0.435159	10	66203428	0.00686	0.29	10	67963186	0.0014	1.06E-17
rs79265434	A	G	A	G	-0.0197	-0.00362126	0.8834	0.93804	7	24581762	0.014189	0.8	7	24621381	0.0022	1.98E-19
rs7928017	A	C	A	C	0.0096	-0.00411441	0.4341	0.306676	11	113578040	0.007417	0.58	11	1.13E+08	0.0014	1.43E-11
rs7931563	T	G	T	G	-0.0109	0.00197069	0.6214	0.581892	11	61678953	0.006938	0.78	11	61446425	0.0014	5.15E-14

rs795230	T	C	T	C	0.0081	-0.00385524	0.4283	0.509846	11	30752978	0.006834	0.57	11	30774525	0.0014	9.56E-09
rs7958371	A	T	A	T	-0.0085	0.007758	0.3552	0.509126	12	103062306	0.006737	0.25	12	1.03E+08	0.0015	5.99E-09
rs7967550	A	G	A	G	-0.0086	0.00753918	0.3998	0.581412	12	91751530	0.006932	0.28	12	92145307	0.0014	1.79E-09
rs7972246	T	C	T	C	0.011	-0.0223223	0.3451	0.844621	12	27056395	0.009247	0.016	12	27209328	0.0015	1.01E-13
rs79728014	A	G	A	G	-0.0134	-0.00637798	0.8553	0.959894	11	46040694	0.017297	0.71	11	46062245	0.002	2.06E-11
rs7974852	A	C	A	C	0.0117	-0.00897158	0.5235	0.598943	12	14358745	0.007049	0.2	12	14511679	0.0014	1.14E-16
rs7977614	A	G	A	G	-0.0134	0.0110165	0.7143	0.712296	12	109677481	0.007597	0.15	12	1.1E+08	0.0016	6.84E-17
rs80037907	T	C	T	C	-0.0107	0.024373	0.1589	0.039145	8	86158684	0.016861	0.15	8	87170913	0.0019	2.16E-08
rs8009933	A	G	A	G	0.0088	-0.00640379	0.6783	0.686599	14	39131008	0.007484	0.39	14	39600212	0.0015	4.72E-09
rs8016504	A	C	A	C	-0.0087	-0.00165777	0.5427	0.704851	14	32838815	0.007509	0.83	14	33308021	0.0014	6.84E-10
rs80223410	T	C	T	C	-0.0118	0.0202965	0.1426	0.037464	12	55974182	0.01797	0.26	12	56367966	0.002	8.64E-09
rs8030487	A	G	A	G	0.0085	-0.0180918	0.6907	0.923631	15	96576365	0.012709	0.15	15	97119595	0.0015	2.52E-08
rs8052523	T	C	T	C	-0.0084	0.00220034	0.5697	0.37512	16	9199389	0.00712	0.76	16	9293246	0.0014	2.73E-09
rs8066044	A	G	A	G	0.009	0.00713398	0.2712	0.426513	17	1464058	0.006828	0.3	17	1367352	0.0016	1.17E-08
rs8097125	T	C	T	C	0.0086	-0.0195179	0.3802	0.23415	18	13005474	0.008097	0.016	18	13005473	0.0014	2.31E-09
rs8103741	A	G	A	G	-0.0108	0.00557654	0.822	0.757925	19	2325006	0.007878	0.48	19	2325005	0.0019	5.17E-09
rs853286	T	G	T	G	0.0124	-0.0071312	0.8921	0.724784	3	64299826	0.007716	0.36	3	64285502	0.0023	4.13E-08
rs854796	A	G	A	G	0.009	-0.00634271	0.6932	0.59414	17	18167447	0.006791	0.35	17	18070761	0.0015	4.66E-09
rs868456	A	G	A	G	0.0089	-0.00441778	0.7117	0.728146	4	32148407	0.007642	0.56	4	32150029	0.0015	1.07E-08
rs870589	A	T	A	T	-0.0079	0.00468201	0.4595	0.736551	17	33287436	0.0074	0.53	17	31614454	0.0014	2.24E-08
rs884108	A	G	A	G	-0.0094	-0.0103153	0.222	0.118156	1	204622109	0.010576	0.33	1	2.05E+08	0.0017	2.39E-08
rs891793	C	G	C	G	-0.0099	-0.000224895	0.5403	0.309798	15	27010874	0.007309	0.98	15	27256021	0.0014	1.87E-12
rs902820	A	T	A	T	-0.0096	0.000893265	0.4224	0.368636	8	142018511	0.007029	0.9	8	1.43E+08	0.0014	1.28E-11
rs911149	T	C	T	C	0.0091	-0.012014	0.2357	0.113112	20	59639582	0.01043	0.25	20	58214637	0.0017	3.93E-08
rs912883	T	C	T	C	0.0087	-0.0135554	0.6781	0.692363	6	41584302	0.007366	0.066	6	41552040	0.0015	6.20E-09
rs9267677	T	C	T	C	0.018	-0.00872932	0.9029	0.90586	6	31924864	0.011726	0.46	6	31892641	0.0024	3.66E-14
rs9289300	T	C	T	C	-0.0164	0.024393	0.8412	0.949568	3	127426145	0.015181	0.11	3	1.27E+08	0.0019	1.00E-17
rs9294770	T	C	T	C	0.0083	0.000936827	0.3879	0.25024	6	67532973	0.008067	0.91	6	68242866	0.0014	8.74E-09
rs929511	T	C	T	C	-0.016	0.0122482	0.1254	0.198847	7	7963386	0.008519	0.15	7	8003017	0.0021	3.91E-14
rs9300612	T	C	T	C	0.0084	-0.000999897	0.6944	0.432037	13	100630965	0.006782	0.88	13	1.01E+08	0.0015	3.07E-08
rs9349956	A	C	A	C	-0.0159	-0.0406914	0.8211	0.919068	6	14718029	0.012455	0.0011	6	14718260	0.0018	4.89E-18
rs9359939	A	C	A	C	-0.0105	-0.00484592	0.2431	0.295149	6	91423523	0.007587	0.52	6	92133241	0.0016	1.51E-10
rs936496	A	G	A	G	-0.0079	0.00933008	0.6229	0.289145	13	35526404	0.007352	0.2	13	36100541	0.0014	4.38E-08
rs9371883	C	G	C	G	-0.0087	-0.00874715	0.643	0.695245	6	155323844	0.007487	0.24	6	1.56E+08	0.0015	3.42E-09
rs9373363	A	G	A	G	-0.0111	0.000149567	0.7485	0.714457	6	142828906	0.007396	0.98	6	1.43E+08	0.0016	5.54E-12
rs9375188	T	C	T	C	0.0212	0.00507803	0.4847	0.413064	6	98107396	0.006943	0.46	6	98555272	0.0014	8.78E-52
rs9375403	C	G	C	G	0.0126	-0.0047718	0.2423	0.240394	6	98383760	0.008116	0.56	6	98831636	0.0016	1.66E-14
rs9386110	T	C	T	C	-0.0163	0.0037735	0.8917	0.899856	6	145305072	0.01145	0.74	6	1.46E+08	0.0023	5.66E-13
rs9388490	T	C	T	C	0.0091	0.00241402	0.4437	0.924111	6	126383649	0.012161	0.84	6	1.27E+08	0.0014	1.15E-10
rs939400	T	G	T	G	-0.0095	-0.00811522	0.3685	0.777137	2	50418752	0.007892	0.3	2	50645890	0.0015	7.52E-11
rs9442750	A	G	A	G	-0.0089	0.00688568	0.7439	0.962776	6	72176309	0.017322	0.69	6	72886012	0.0016	3.15E-08
rs9446060	A	G	A	G	0.0077	-0.0175404	0.5566	0.152017	6	68842379	0.009383	0.062	6	69552271	0.0014	4.33E-08
rs9465509	A	G	A	G	-0.0084	0.00229288	0.4824	0.40562	6	19743898	0.006947	0.74	6	19744129	0.0014	2.35E-09
rs9490512	A	G	A	G	-0.0136	-0.0111124	0.4496	0.352546	6	98090117	0.007084	0.12	6	98537993	0.0014	4.00E-22
rs9513754	T	C	T	C	0.0096	-0.00686749	0.2906	0.152257	13	100391166	0.009569	0.47	13	1.01E+08	0.0016	6.64E-10
rs9527662	A	T	A	T	0.0094	0.00139645	0.4193	0.065082	13	57446131	0.012365	0.91	13	58020265	0.0014	3.37E-11
rs9527905	A	G	A	G	-0.0094	-0.00138723	0.5828	0.590298	13	58828899	0.006953	0.84	13	59403033	0.0014	4.11E-11
rs9529100	A	G	A	C	0.0126	-0.0120583	0.8609	0.07829	13	66587647	0.012507	0.33	13	67141932	0.002	5.82E-10
rs9529146	T	C	T	C	0.0127	0.0104364	0.2282	0.473103	13	31211267	0.00683	0.13	13	31785404	0.0017	3.48E-14
rs9536462	A	G	A	G	0.0133	-0.0112244	0.1454	0.088617	13	53550515	0.011904	0.35	13	54124650	0.002	2.10E-11
rs9545395	T	C	T	C	0.0138	0.00727703	0.8702	0.697887	13	35796833	0.007278	0.32	13	36370970	0.0021	3.81E-11
rs9563168	A	G	A	G	0.0101	-0.014897	0.2117	0.143132	13	53673692	0.00985	0.13	13	54247827	0.0017	4.07E-09
rs9568798	T	C	T	C	-0.0093	0.00216322	0.2912	0.245437	13	53040419	0.00797	0.79	13	53614554	0.0016	2.30E-09
rs9611597	A	T	A	T	-0.0107	-0.0119594	0.835	0.988953	22	41468186	0.03303	0.72	22	41864190	0.0019	2.30E-08
rs9649	T	C	T	C	0.0108	-0.00879605	0.1749	0.060038	17	35723915	0.013963	0.53	17	34050934	0.0019	6.13E-09

rs9683585	C	G	C	G	0.0084	0.00265078	0.5677	0.623199	4	2701077	0.007002	0.71	4	2702804	0.0014	2.46E-09
rs969512	A	T	A	T	-0.0106	0.00201089	0.6596	0.536503	4	146951590	0.006908	0.77	4	1.48E+08	0.0015	9.84E-13
rs9771228	T	C	T	C	0.0096	-0.00158259	0.6491	0.497839	7	32282884	0.006846	0.82	7	32322496	0.0015	5.91E-11
rs977143	A	G	A	G	0.0085	-0.00491365	0.3725	0.021134	18	40448201	0.02256	0.83	18	38028165	0.0015	3.95E-09
rs978807	A	G	A	G	0.0139	0.00694913	0.1885	0.246638	18	39318495	0.00783	0.37	18	36898459	0.0018	9.14E-15
rs981230	T	C	T	C	0.01	0.021557	0.4793	0.186119	5	59744032	0.008624	0.012	5	59039858	0.0014	1.28E-12
rs981883	A	G	A	G	0.0099	-0.00489204	0.3886	0.300672	5	57812162	0.00743	0.51	5	57107989	0.0014	6.67E-12
rs9820604	T	C	T	C	0.0115	0.0112432	0.8254	0.614793	3	72314523	0.007078	0.11	3	72363674	0.0018	4.80E-10
rs9821664	A	G	A	G	0.0094	0.0125846	0.2105	0.579251	3	127284267	0.006869	0.067	3	1.27E+08	0.0017	4.52E-08
rs9853928	T	C	T	C	-0.0127	0.0106199	0.2102	0.42195	3	103576540	0.006925	0.13	3	1.03E+08	0.0017	1.59E-13
rs9858921	A	G	A	G	-0.0087	0.00166859	0.4661	0.43804	3	161431158	0.006838	0.81	3	1.61E+08	0.0014	5.19E-10
rs9859556	T	G	T	G	0.029	-0.0198273	0.3132	0.06292	3	49418553	0.013974	0.16	3	49455986	0.0015	4.61E-82
rs9870317	A	C	A	C	0.0086	-0.000879718	0.3212	0.576609	3	182796848	0.006871	0.9	3	1.83E+08	0.0015	9.35E-09
rs9877225	T	C	T	C	0.009	-0.00844779	0.7617	0.661383	3	70582439	0.007217	0.24	3	70631590	0.0016	4.22E-08
rs9882532	T	C	T	C	0.0124	0.0059841	0.6368	0.922671	3	16824346	0.012344	0.63	3	16865845	0.0015	2.15E-17
rs9886703	A	T	A	T	-0.0136	-0.0104855	0.1671	0.207493	9	79631436	0.00836	0.21	9	82246351	0.0019	4.46E-13
rs9888796	T	C	T	C	0.0113	-0.0147549	0.2625	0.145533	16	68263686	0.00958	0.12	16	68297589	0.0016	1.20E-12
rs9916901	T	G	T	G	0.0092	0.0108158	0.7377	0.492555	18	42658370	0.006868	0.12	18	40238335	0.0016	8.40E-09
rs9929762	A	G	A	G	0.0096	0.00146015	0.5595	0.513689	16	78135778	0.0068	0.83	16	78169675	0.0014	1.03E-11
rs9933256	A	G	A	G	0.0119	-0.0138456	0.5558	0.854227	16	1196748	0.009645	0.15	16	1246748	0.0014	6.06E-17
rs995698	A	G	A	G	-0.0099	-0.0152692	0.538	0.10951	9	85382954	0.01083	0.16	9	87997869	0.0014	1.53E-12
rs9974899	T	C	T	C	-0.0093	0.00175851	0.2538	0.408501	21	40634515	0.006886	0.8	21	42006441	0.0016	1.22E-08

Supplementary Table 2b. List of SNPs for Monthly household income and their effects on TEWL

SNP	effect_alle	other_allel	effect_alle	other_allel	beta.Incon	beta.TEWL	eaf.Incom	eaf.TEWL	chr.TEWL	pos.TEWL	se.TEWL	pval.TEWL	chr.Incom	pos.Income	se.Income	pval.Income
chr1:2046	A	G	A	G	0.01154	0.003883	0.2029	0.298031	1	2.05E+08	0.007683	0.61	1	204617919	0.002014	9.99E-09
chr13:984	T	C	T	C	-0.01116	-0.0106	0.4852	0.432517	13	98447792	0.00689	0.12	13	98447792	0.002024	3.50E-08
chr17:458	C	A	C	A	-0.01329	0.03313	0.3822	0.007205	17	45898049	0.040709	0.42	17	45898049	0.002	3.04E-11
chr17:461	T	A	A	G	-0.01333	0.031673	0.3084	0.006964	17	46101518	0.041394	0.44	17	46101518	0.002	2.65E-11
chr17:461	T	C	T	A	-0.01317	-0.0104	0.3144	0.006964	17	46142465	0.0414	0.8	17	46142465	0.002001	4.69E-11
chr19:131	T	C	T	C	-0.01243	-0.00289	0.1178	0.022815	19	13101211	0.022787	0.9	19	13101211	0.001987	4.04E-10
chr2:1001	A	T	A	T	0.011346	-0.01354	0.3941	0.616955	2	1E+08	0.007068	0.055	2	100188811	0.002029	2.24E-08
chr2:1003	C	G	C	G	0.01202	0.002034	0.4534	0.725024	2	1E+08	0.007318	0.78	2	100322455	0.002025	2.92E-09
chr2:1887	G	C	G	C	-0.0112	-0.0239	0.02391	0.028098	2	1.89E+08	0.020917	0.25	2	188711617	0.001986	1.71E-08
chr2:1986	A	T	A	T	-0.01152	0.000859	0.4745	0.737992	2	1.99E+08	0.007448	0.91	2	198632391	0.002019	1.16E-08
chr2:2117	T	G	T	G	-0.01221	-0.00606	0.3549	0.553554	2	2.12E+08	0.006859	0.38	2	211727319	0.002018	1.46E-09
chr2:2118	G	A	G	A	0.012051	0.006375	0.3149	0.371278	2	2.12E+08	0.006976	0.36	2	211813206	0.002015	2.22E-09
chr3:4968	A	G	A	G	0.013162	-0.02325	0.2852	0.049472	3	49682296	0.015677	0.14	3	49682296	0.002018	6.98E-11
chr3:4971	C	A	C	A	0.011457	-0.04958	0.1256	0.01561	3	49712272	0.027068	0.067	3	49712272	0.002017	1.34E-08
chr5:6118	G	A	G	A	0.012492	0.02456	0.2934	0.93756	5	61188833	0.013357	0.066	5	61188833	0.002009	5.08E-10
chr5:6120	G	A	G	A	0.015664	0.008479	0.4178	0.861431	5	61204446	0.009692	0.38	5	61204446	0.002021	9.04E-15
chr5:6144	G	A	G	A	-0.01244	-0.01023	0.3222	0.941403	5	61441122	0.014746	0.49	5	61441122	0.002006	5.64E-10
chr6:9810	A	C	A	C	0.017284	0.005152	0.4818	0.413305	6	98102413	0.006938	0.46	6	98102413	0.002025	1.39E-17
chr7:1050	A	G	A	G	-0.01145	0.00129	0.4767	0.871278	7	1.05E+08	0.009368	0.89	7	105022415	0.002029	1.67E-08
chr9:2334	A	C	A	C	0.01204	-0.00196	0.4412	0.825648	9	23349984	0.008758	0.82	9	23349984	0.00202	2.51E-09
chr9:2337	G	A	G	A	0.011293	-0.00078	0.3679	0.520173	9	23378222	0.00676	0.91	9	23378222	0.002025	2.46E-08
rs1042958	C	T	C	T	0.017125	-0.00643	0.414	0.426513	9	23346852	0.006926	0.35	9	23346852	0.002016	1.98E-17
rs1087222	T	G	T	G	-0.0172	-0.00441	0.3953	0.588136	6	97987249	0.007009	0.53	6	97987249	0.002015	1.40E-17
rs1112382	A	G	A	G	0.011969	0.030112	0.3739	0.929635	2	1E+08	0.013099	0.022	2	100313672	0.002015	2.85E-09
rs1194353	T	C	T	C	0.011121	-0.02688	0.239	0.035783	4	96258935	0.017827	0.13	4	96258935	0.002024	3.92E-08
rs1211914	T	C	T	C	0.012652	0.001192	0.2668	0.739433	1	43777612	0.007634	0.88	1	43777612	0.002037	5.24E-10
rs1261488	G	A	G	A	0.01461	-0.00074	0.3777	0.542267	2	1E+08	0.006725	0.91	2	100232879	0.002019	4.66E-13
rs1296785	G	A	G	A	0.012344	0.003615	0.3308	0.635687	18	37558282	0.007326	0.62	18	37558282	0.002058	2.01E-09
rs1300294	A	T	A	T	0.013929	-0.00593	0.2672	0.548751	2	1E+08	0.006737	0.38	2	100185497	0.002022	5.70E-12
rs1315866	T	C	T	C	-0.01188	0.013105	0.4517	0.186599	5	60773230	0.008502	0.12	5	60773230	0.002022	4.21E-09
rs1338549	G	T	G	T	-0.01371	-0.00566	0.4657	0.588617	6	97864267	0.006991	0.42	6	97864267	0.00202	1.14E-11
rs1933720	C	T	C	T	0.013999	0.001946	0.3626	0.302594	6	97849956	0.007432	0.79	6	97849956	0.002014	3.63E-12
rs2094889	C	T	C	T	0.012113	-0.01062	0.3179	0.589097	9	23382384	0.006821	0.12	9	23382384	0.002018	1.93E-09
rs2255158	G	A	G	A	0.011484	0.009928	0.3671	0.909942	20	3149944	0.011758	0.4	20	3149944	0.002016	1.22E-08
rs2280406	A	G	A	G	-0.01274	0.00774	0.4837	0.154179	3	49904003	0.009527	0.42	3	49904003	0.002026	3.22E-10
rs2332719	G	A	G	A	-0.01333	7.41E-05	0.2784	0.486551	3	1.24E+08	0.006863	0.99	3	123994119	0.002068	1.16E-10
rs2388148	T	C	T	C	0.011448	0.001305	0.4312	0.301393	6	97785319	0.007555	0.86	6	97785319	0.002018	1.41E-08
rs2503776	T	A	T	A	-0.0137	-0.0108	0.4769	0.354947	6	98072650	0.007047	0.13	6	98072650	0.002021	1.21E-11
rs2563332	T	C	T	C	-0.01156	0.0106	0.2075	0.143852	5	1.41E+08	0.009794	0.28	5	140646169	0.002017	9.90E-09
rs2855855	C	T	C	T	0.011223	-0.00277	0.1066	0.15658	6	1.14E+08	0.009216	0.76	6	113863071	0.002046	4.11E-08
rs2889678	G	T	G	T	0.011228	-0.00673	0.2254	0.721662	4	1.32E+08	0.007679	0.38	4	131729441	0.002038	3.60E-08
rs306755	C	T	C	T	0.011925	0.001939	0.4714	0.556916	20	3119106	0.006898	0.78	20	3119106	0.002024	3.82E-09
rs32940	C	T	C	T	0.011072	0.004581	0.2976	0.700768	5	1.42E+08	0.007507	0.54	5	141752719	0.002012	3.73E-08
rs3619810	A	G	A	G	-0.01184	0.002232	0.3879	0.080932	5	61263329	0.012091	0.85	5	61263329	0.002018	4.47E-09

rs37976	T	C	T	C	0.011976	-0.00067	0.4404	0.564841	7	7975326	0.006851	0.92	7	7975326	0.002022	3.14E-09
rs4257287	G	A	G	A	-0.0126	-0.00742	0.1025	0.011768	18	55076600	0.031405	0.81	18	55076600	0.002033	5.80E-10
rs4443016	G	C	G	C	0.012947	0.002363	0.4941	0.603026	2	1E+08	0.006926	0.73	2	100260166	0.002016	1.36E-10
rs4583848	C	A	C	A	-0.01504	-0.00737	0.499	0.25048	5	61305607	0.007833	0.35	5	61305607	0.00203	1.29E-13
rs4587178	C	T	C	T	-0.0133	-0.0016	0.3846	0.396974	6	97974115	0.007033	0.82	6	97974115	0.002027	5.35E-11
rs4799936	A	G	A	G	0.011652	-0.00358	0.2871	0.550913	18	37524865	0.00695	0.61	18	37524865	0.002079	2.07E-08
rs5746822	C	T	C	T	0.01201	-0.00855	0.394	0.391931	5	60799177	0.006829	0.21	5	60799177	0.002017	2.62E-09
rs5977930	C	T	C	T	-0.01133	-0.01081	0.4123	0.323247	2	2.12E+08	0.007377	0.14	2	211778050	0.00202	2.06E-08
rs6218302	T	G	T	G	-0.01137	-0.01385	0.3147	0.061479	2	2.12E+08	0.013953	0.32	2	211766758	0.002008	1.48E-08
rs6236619	G	C	G	C	0.013793	-0.00252	0.3414	0.3756	5	61188108	0.007015	0.72	5	61188108	0.002011	7.00E-12
rs6477496	T	C	T	C	0.011258	-0.0171	0.1887	0.023775	9	96494130	0.022108	0.44	9	96494130	0.002028	2.85E-08
rs6699397	G	A	G	A	-0.0122	0.00609	0.3672	0.39121	1	90746659	0.007082	0.39	1	90746659	0.002021	1.59E-09
rs6931604	T	C	T	C	0.015038	0.016696	0.4019	0.579251	6	98130339	0.006899	0.016	6	98130339	0.002036	1.53E-13
rs6933464	T	C	T	C	0.011294	0.006809	0.2832	0.412584	6	98293860	0.006814	0.32	6	98293860	0.002012	1.97E-08
rs7159268	T	C	T	C	0.011341	-0.02196	0.2554	0.018972	5	60825411	0.023908	0.36	5	60825411	0.002006	1.56E-08
rs7566675	A	G	G	T	-0.01385	0.028852	0.222	0.006964	17	46012782	0.041398	0.49	17	46012782	0.002001	4.56E-12
rs784256	A	G	A	G	-0.01154	0.00835	0.1814	0.982949	18	55731395	0.025324	0.74	18	55731395	0.002005	8.58E-09
rs9517310	T	C	T	C	0.011125	0.006828	0.3107	0.173391	13	98448220	0.009026	0.45	13	98448220	0.002009	3.09E-08
rs9653442	T	C	T	C	0.011995	-0.01508	0.4652	0.60975	2	1E+08	0.006904	0.029	2	100208905	0.002029	3.38E-09

Supplementary Table 2c. List of SNPs for Diabetes Mellitus(DM) and their effects on TEWL

SNP	effect_alle	other_allel	effect_alle	other_allel	beta.DM	beta.TEWL	eaf.DM	eaf.TEWL	chr.TEWL	pos.TEWL	se.TEWL	pval.TEWL	chr.DM	pos.DM	se.DM	pval.DM
chr1:1199	T	C	T	C	0.086178	0.01411	0.1089	0.044909	1	1.2E+08	0.016405	0.39	1	1.21E+08	0.009363	1.72E-20
chr1:1779	C	A	C	A	0.04879	-0.00414	0.1981	0.128963	1	1.78E+08	0.010313	0.69	1	1.78E+08	0.007255	8.75E-12
chr1:2051	G	C	G	C	0.039221	0.004602	0.4417	0.925312	1	2.05E+08	0.012937	0.72	1	2.05E+08	0.004906	6.50E-16
chr1:2064	C	G	C	G	0.039221	-0.00226	0.494	0.767291	1	2.06E+08	0.00798	0.78	1	2.07E+08	0.007395	5.67E-08
chr1:2355	G	A	G	A	0.039221	0.000999	0.6322	0.681316	1	2.36E+08	0.007227	0.89	1	2.36E+08	0.007324	4.28E-08
chr10:112	T	C	T	C	0.314811	0.008164	0.295	0.052354	10	1.13E+08	0.014522	0.57	10	1.15E+08	0.007449	0
chr10:113	A	G	A	G	0.04879	0.005236	0.7162	0.971662	10	1.13E+08	0.020605	0.8	10	1.15E+08	0.007255	8.75E-12
chr10:791	G	C	G	C	0.067659	0.00309	0.533	0.663545	10	79193069	0.007273	0.67	10	80952826	0.00712	1.02E-21
chr11:173	C	T	C	T	0.067659	-0.00428	0.3624	0.360711	11	17386857	0.007089	0.55	11	17408404	0.00712	1.02E-21
chr11:273	A	G	A	G	0.04879	-0.01087	0.6896	0.780259	11	2734318	0.00824	0.19	11	2755548	0.007324	1.35E-11
chr11:283	C	A	C	A	0.113329	-0.00273	0.426	0.341739	11	2835964	0.007277	0.71	11	2857194	0.006803	1.32E-62
chr11:324	T	C	T	C	0.039221	0.006937	0.1614	0.09318	11	32439327	0.01167	0.55	11	32460873	0.009813	3.21E-05
chr11:932	C	T	C	T	0.039221	-0.00352	0.7057	0.82829	11	93280365	0.009159	0.7	11	93013531	0.007324	4.28E-08
chr12:117	A	G	A	G	0.058269	7.02E-05	0.1438	0.208213	12	1.18E+08	0.008518	0.99	12	1.18E+08	0.009628	7.14E-10
chr12:118	A	T	A	T	0.039221	0.001813	0.8278	0.877522	12	1.18E+08	0.010661	0.86	12	1.18E+08	0.009813	3.21E-05
chr12:127	G	T	G	T	0.04879	-0.00415	0.235	0.075889	12	12718165	0.012777	0.75	12	12871099	0.007324	1.35E-11
chr12:658	T	A	T	A	0.09531	0.0022	0.1042	0.106388	12	65827280	0.011199	0.84	12	66221060	0.011545	7.56E-17
chr13:109	G	A	G	A	0.039221	-0.00156	0.6685	0.550672	13	1.1E+08	0.00684	0.82	13	1.1E+08	0.007395	5.67E-08
chr14:103	G	T	G	T	0.039221	-0.00465	0.6532	0.853746	14	1.03E+08	0.009681	0.63	14	1.04E+08	0.007395	5.67E-08
chr14:228	G	C	G	C	0.039221	0.032606	0.228	0.050672	14	22819726	0.015459	0.035	14	23288935	0.007324	4.28E-08
chr15:570	G	A	G	T	0.09531	0.051691	0.0369	0.006724	15	57071996	0.039349	0.19	15	57456802	0.016313	2.57E-09
chr15:621	G	C	G	C	0.04879	0.007195	0.5663	0.664505	15	62102065	0.007235	0.32	15	62394264	0.007324	1.35E-11
chr15:756	G	T	G	T	0.04879	0.010789	0.7586	0.644813	15	75639788	0.007115	0.13	15	75932129	0.007324	1.35E-11
chr15:898	T	C	T	C	0.067659	-0.00041	0.2672	0.20389	15	89880061	0.008375	0.96	15	90423293	0.007186	2.37E-21
chr16:245	T	C	T	C	0.058269	0.005125	0.8246	0.585975	16	245796	0.007051	0.47	16	295795	0.007186	2.57E-16
chr16:815	T	C	T	C	0.04879	-0.00035	0.2996	0.407781	16	81501185	0.007059	0.96	16	81534790	0.007255	8.75E-12
chr17:377	T	A	T	A	0.076961	-0.01167	0.4808	0.245917	17	37739961	0.007851	0.14	17	36099952	0.007054	5.14E-28
chr17:392	G	C	G	C	0.04879	-0.00634	0.1568	0.386167	17	3924792	0.006963	0.36	17	3828086	0.009719	2.58E-07
chr17:988	C	G	C	G	0.039221	0.047094	0.3238	0.016331	17	9881870	0.025427	0.064	17	9785187	0.004906	6.50E-16
chr18:603	C	A	C	A	0.139762	-0.06031	0.9763	0.995677	18	60389333	0.052461	0.25	18	58056566	0.022197	1.52E-10
chr18:631	T	C	T	C	0.04879	-0.01238	0.6142	0.568684	18	63178651	0.006954	0.075	18	60845884	0.004859	5.05E-24
chr19:192	T	A	T	A	0.09531	0.003409	0.0769	0.086936	19	19277691	0.012168	0.78	19	19388500	0.011651	1.41E-16
chr19:333	C	G	C	G	0.039221	0.000139	0.5226	0.583573	19	33399932	0.006809	0.98	19	33890838	0.007395	5.67E-08
chr19:521	A	G	A	G	0.076961	-0.0065	0.0371	0.006484	19	5212300	0.042911	0.88	19	5224998	0.018905	2.34E-05
chr19:790	A	G	A	G	0.04879	2.71E-05	0.3902	0.413305	19	7905750	0.007035	1	19	7970635	0.007324	1.35E-11
chr2:2542	G	T	G	T	0.039221	0.012029	0.7314	0.963257	2	25420352	0.016992	0.48	2	25643221	0.004906	6.50E-16
chr2:4320	A	G	A	G	0.039221	-0.00155	0.5007	0.691402	2	43203301	0.007627	0.84	2	43430440	0.007395	5.67E-08
chr2:5908	G	A	G	A	0.039221	0.015115	0.392	0.361191	2	59080590	0.007092	0.033	2	59307725	0.007395	5.67E-08
chr2:6512	G	A	G	A	0.067659	-0.00342	0.3038	0.333333	2	65128136	0.007119	0.63	2	65355270	0.011979	8.11E-09
chr20:340	A	G	A	G	0.039221	0.015973	0.6572	0.837416	20	34008898	0.009299	0.086	20	32596704	0.007324	4.28E-08

chr22:301!	G	A	G	A	0.086178	0.005258	0.9136	0.876561	22	30194037	0.010516	0.62	22	30609554	0.011758	1.16E-13
chr22:320	C	T	C	T	0.067659	-0.01621	0.9117	0.987512	22	32079891	0.031091	0.6	22	32348841	0.011979	8.11E-09
chr22:499	C	T	C	T	0.039221	-0.0006	0.275	0.258165	22	49963202	0.007794	0.94	22	50356850	0.007395	5.67E-08
chr3:1244	G	T	G	T	0.058269	0.001585	0.4249	0.888329	3	12447843	0.010501	0.88	3	12489342	0.007255	4.79E-16
chr3:1252	T	C	T	C	0.039221	-3.96E-05	0.4651	0.231508	3	1.25E+08	0.008141	1	3	1.25E+08	0.004906	6.50E-16
chr3:1840	A	C	A	C	0.039221	0.00884	0.4538	0.168348	3	1.84E+08	0.00892	0.32	3	1.84E+08	0.007395	5.67E-08
chr3:1869!	A	G	A	G	0.076961	-0.00597	0.0576	0.06316	3	1.87E+08	0.014126	0.67	3	1.87E+08	0.014176	2.83E-08
chr3:1880	C	T	C	T	0.058269	-0.00804	0.6104	0.971662	3	1.88E+08	0.01967	0.68	3	1.88E+08	0.007186	2.57E-16
chr4:1361	A	C	A	C	0.039221	-0.00219	0.4457	0.862392	4	1.36E+08	0.00968	0.82	4	1.37E+08	0.007395	5.67E-08
chr4:1525!	T	A	T	A	0.058269	-0.01299	0.7045	0.502642	4	1.53E+08	0.006783	0.056	4	1.54E+08	0.007255	4.79E-16
chr4:1567	C	A	C	A	0.039221	-0.00892	0.6767	0.70341	4	1.57E+08	0.007419	0.23	4	1.58E+08	0.007324	4.28E-08
chr4:6300	A	G	A	G	0.04879	0.011821	0.7087	0.982709	4	6300792	0.026207	0.65	4	6302519	0.009719	2.58E-07
chr4:6305	C	G	C	G	0.058269	0.016561	0.588	0.896494	4	6305036	0.010938	0.13	4	6306763	0.009628	7.14E-10
chr5:1340	A	G	A	G	0.029559	-0.0154	0.7028	0.754323	5	1.34E+08	0.007994	0.054	5	1.33E+08	0.007395	3.20E-05
chr5:1476	C	G	C	G	0.067659	-0.0114	0.904	0.591258	5	14767983	0.006894	0.098	5	14768092	0.011979	8.11E-09
chr5:5411	C	A	C	A	0.039221	-0.00252	0.6909	0.725024	5	54116790	0.007515	0.74	5	53412620	0.004906	6.50E-16
chr5:7570	T	C	T	C	0.04879	0.003833	0.6053	0.433718	5	75707853	0.006927	0.58	5	75003678	0.007255	8.75E-12
chr5:8728	G	A	G	A	0.039221	0.003594	0.2585	0.465418	5	87281535	0.006777	0.6	5	86577352	0.007324	4.28E-08
chr6:2067!	G	A	G	A	0.139762	-0.00063	0.2735	0.435639	6	20679478	0.006845	0.93	6	20679709	0.008874	3.46E-56
chr6:5082	C	A	C	A	0.058269	-0.00191	0.1844	0.232229	6	50821065	0.008161	0.82	6	50788778	0.009628	7.14E-10
chr6:7035!	C	A	C	A	0.029559	0.013621	0.4085	0.910903	6	7035501	0.011772	0.25	6	7035734	0.007395	3.20E-05
chr7:1178!	A	C	A	C	0.039221	0.00165	0.3127	0.164265	7	1.18E+08	0.009183	0.86	7	1.17E+08	0.007324	4.28E-08
chr7:1303	C	A	C	A	0.029559	0.004418	0.3271	0.363112	7	1.3E+08	0.007088	0.53	7	1.3E+08	0.004954	1.21E-09
chr7:1571	G	C	G	C	0.058269	0.00313	0.6729	0.574688	7	1.57E+08	0.006971	0.65	7	1.57E+08	0.004813	4.93E-34
chr8:1091	C	G	C	G	0.039221	-0.00441	0.5342	0.475264	8	1.09E+08	0.006911	0.52	8	1.1E+08	0.007395	5.67E-08
chr8:3100	T	C	T	C	0.039221	0.009868	0.3135	0.067483	8	31006422	0.012531	0.43	8	30863938	0.007324	4.28E-08
chr8:4165	C	A	C	A	0.067659	0.007176	0.8292	0.864553	8	41651058	0.010157	0.48	8	41508577	0.009538	6.52E-13
chr9:2066	C	T	C	T	0.039221	0.013625	0.8431	0.767291	9	20662704	0.00818	0.096	9	20662703	0.007324	4.28E-08
chr9:2215	A	C	A	C	0.029559	-0.00142	0.6599	0.697646	9	22157909	0.007607	0.85	9	22157908	0.007395	3.20E-05
chr9:3965	A	C	A	C	0.029559	-0.00209	0.4906	0.485351	9	3965689	0.006778	0.76	9	3965689	0.004954	1.21E-09
rs1005752	A	C	A	C	0.076961	0.014514	0.7154	0.37464	15	77525786	0.007095	0.041	15	77818128	0.007054	5.14E-28
rs1009663	C	T	C	T	0.067659	0.009386	0.8766	0.884726	8	19973410	0.010885	0.39	8	19830921	0.009538	6.52E-13
rs1009761	T	C	T	C	0.039221	0.008129	0.4847	0.271374	8	94949398	0.007553	0.28	8	95961626	0.007324	4.28E-08
rs1019353	T	G	T	G	0.039221	0.008436	0.6103	0.423391	2	58753929	0.006867	0.22	2	58981064	0.007395	5.67E-08
rs1019525	T	C	T	C	0.067659	0.000905	0.5864	0.884726	2	1.65E+08	0.010655	0.93	2	1.66E+08	0.004768	5.36E-46
rs1022806	T	C	T	C	0.067659	0.013779	0.5373	0.705331	7	15023944	0.00743	0.064	7	15063569	0.00712	1.02E-21
rs1030574	A	G	A	G	0.24686	0.004906	0.0145	0.098463	1	1.51E+08	0.011433	0.67	1	1.51E+08	0.0538	2.23E-06
rs1040643	A	G	A	G	0.04879	0.004351	0.5625	0.635207	19	45653761	0.007074	0.54	19	46157019	0.004859	5.05E-24
rs1042725	T	C	T	C	0.04879	-0.00139	0.4902	0.826609	12	65964567	0.009109	0.88	12	66358347	0.007324	1.35E-11
rs1046914	G	A	G	A	0.029559	0.011398	0.4849	0.566042	18	63001037	0.006981	0.1	18	60668270	0.004954	1.21E-09
rs1061810	A	C	A	C	0.04879	-0.00031	0.288	0.286984	11	43856384	0.007626	0.97	11	43877934	0.007255	8.75E-12
rs1075039	A	G	A	G	0.04879	-0.00325	0.2817	0.21806	11	1.28E+08	0.008143	0.69	11	1.28E+08	0.007255	8.75E-12

rs1075728	T	C	T	C	0.10436	-0.00358	0.4298	0.584054	9	22134173	0.006955	0.61	9	22134172	0.009194	3.66E-30
rs1081166	G	A	G	A	0.239017	-0.00786	0.8282	0.615754	9	22134069	0.007021	0.26	9	22134068	0.010084	1.73E-124
rs1083096	G	C	G	C	0.09531	0.000667	0.2765	0.445245	11	92975544	0.006944	0.92	11	92708710	0.006926	2.20E-43
rs1084299	C	T	C	T	0.076961	-0.00643	0.8054	0.79659	12	27812217	0.008387	0.44	12	27965150	0.00712	1.55E-27
rs1084895	C	T	C	T	0.039221	-0.00193	0.8039	0.980548	12	3921938	0.024529	0.94	12	4031104	0.007324	4.28E-08
rs1088210	T	C	T	C	0.058269	-0.01064	0.5872	0.305716	10	92702670	0.007579	0.16	10	94462427	0.009628	7.14E-10
rs1089382	T	C	T	C	0.058269	-0.00436	0.8533	0.71926	11	1.28E+08	0.007783	0.57	11	1.28E+08	0.009628	7.14E-10
rs1093839	A	G	A	G	0.04879	0.00419	0.4289	0.264649	4	45184122	0.007607	0.58	4	45186139	0.007324	1.35E-11
rs10962	C	G	C	G	0.04879	0.017002	0.2257	0.252882	17	37686448	0.007863	0.031	17	36046451	0.009719	2.58E-07
rs1097443	C	A	C	A	0.04879	0.003686	0.3567	0.39097	9	4291928	0.006888	0.59	9	4291928	0.007255	8.75E-12
rs1104259	G	T	G	T	0.039221	0.001336	0.6649	0.50072	11	2097630	0.007008	0.85	11	2118860	0.004906	6.50E-16
rs1106302	C	T	C	T	0.058269	0.002384	0.18	0.180115	12	4191006	0.009086	0.79	12	4300172	0.007255	4.79E-16
rs1106529	A	G	A	G	0.058269	0.006964	0.0754	0.007925	12	1.21E+08	0.038887	0.86	12	1.21E+08	0.011979	5.74E-07
rs1107033	A	G	A	G	0.04879	0.001785	0.3578	0.178674	15	41517007	0.008695	0.84	15	41809205	0.004859	5.05E-24
rs1112718	A	G	A	G	0.058269	0.003003	0.5984	0.170749	10	92719350	0.008916	0.74	10	94479107	0.012092	7.23E-07
rs1113782	C	G	C	G	0.039221	-0.00382	0.5751	0.68684	9	78744197	0.007461	0.61	9	81359113	0.007395	5.67E-08
rs1125765	T	C	T	C	0.086178	0.001229	0.2184	0.506004	10	12265895	0.006706	0.85	10	12307894	0.00699	3.14E-35
rs1127215	C	T	C	T	0.04879	-0.02097	0.5838	0.722622	1	1.17E+08	0.007606	0.0058	1	1.18E+08	0.004859	5.05E-24
rs1144475	T	C	T	C	0.058269	-0.02083	0.0839	0.009366	4	52340927	0.034089	0.54	4	53207093	0.012092	7.23E-07
rs1149606	T	C	T	C	0.076961	0.065772	0.8181	0.985351	7	1.03E+08	0.027857	0.018	7	1.02E+08	0.014176	2.83E-08
rs1164243	G	C	G	C	0.039221	-0.00189	0.399	0.431076	16	30034468	0.006938	0.79	16	30045789	0.004906	6.50E-16
rs1165749	G	T	G	T	0.058269	-0.02154	0.1002	0.033381	17	67652311	0.019171	0.26	17	65648427	0.009628	7.14E-10
rs1167610	C	G	T	C	0.19062	-0.00418	0.9818	0.01561	10	1.13E+08	0.027455	0.88	10	1.15E+08	0.02532	2.57E-14
rs1168005	A	G	A	G	0.058269	0.013279	0.863	0.880403	2	16393401	0.010616	0.21	2	16574669	0.009628	7.14E-10
rs1168868	G	C	G	C	0.04879	0.005598	0.7278	0.92243	2	1.21E+08	0.012819	0.66	2	1.21E+08	0.007324	1.35E-11
rs1168893	C	G	C	G	0.039221	0.00058	0.8494	0.82781	2	1.21E+08	0.008993	0.95	2	1.21E+08	0.009813	3.21E-05
rs1169130	C	T	C	T	0.039221	0.019661	0.83	0.981508	7	44325950	0.025398	0.44	7	44365549	0.009813	3.21E-05
rs1169980	C	T	C	T	0.039221	0.000194	0.5359	0.438761	20	50215598	0.00685	0.98	20	48832135	0.007324	4.28E-08
rs1170806	A	G	A	G	0.086178	0.010881	0.7723	0.973823	3	1.23E+08	0.019372	0.57	3	1.23E+08	0.00699	3.14E-35
rs1170907	G	A	G	A	0.131028	0.016222	0.8765	0.95341	3	12295008	0.015736	0.3	3	12336507	0.01124	1.05E-31
rs1171795	G	T	G	T	0.039221	0.002197	0.6208	0.826849	3	1.86E+08	0.008914	0.81	3	1.86E+08	0.009813	3.21E-05
rs1175902	G	A	G	A	0.067659	-0.005	0.2321	0.467819	6	1.26E+08	0.006687	0.45	6	1.27E+08	0.007186	2.37E-21
rs1178699	A	C	A	C	0.029559	0.008333	0.644	0.79683	8	94672919	0.008586	0.33	8	95685147	0.004954	1.21E-09
rs1182001	T	C	T	C	0.14842	-0.03644	0.9733	0.986551	11	69633990	0.029972	0.22	11	69448758	0.019888	4.24E-14
rs1184287	G	T	G	T	0.039221	-0.01666	0.7345	0.856628	13	30468315	0.009753	0.088	13	31042452	0.007324	4.28E-08
rs1192670	C	T	C	T	0.239017	-0.00715	0.6262	0.597983	3	46884049	0.007035	0.31	3	46925539	0.042112	6.91E-09
rs1196726	G	C	G	C	0.039221	0.01173	0.4855	0.59438	6	43792590	0.006941	0.091	6	43760327	0.004906	6.50E-16
rs1200143	C	T	C	T	0.039221	0.000595	0.3722	0.557637	9	34074478	0.006791	0.93	9	34074476	0.007324	4.28E-08
rs1223690	T	C	T	C	0.139762	0.00148	0.9872	0.883045	9	94735212	0.010713	0.89	9	97497494	0.031094	3.48E-06
rs1260326	C	T	C	T	0.067659	0.003182	0.6069	0.538665	2	27508073	0.006816	0.64	2	27730940	0.004768	5.36E-46
rs1264025	C	A	C	A	0.039221	0.005181	0.7149	0.606868	4	17791246	0.006831	0.45	4	17792869	0.004906	6.50E-16
rs1271977	T	C	T	C	0.039221	0.008015	0.5376	0.601585	8	1.45E+08	0.006954	0.25	8	1.46E+08	0.004906	6.50E-16

rs1280297	A	G	A	G	0.029559	-7.97E-05	0.4281	0.743516	11	1683366	0.007431	0.99	11	1704596	0.007395	3.20E-05
rs1281140	A	G	A	G	0.04879	0.005877	0.3305	0.126081	12	1.32E+08	0.010348	0.57	12	1.33E+08	0.007255	8.75E-12
rs1291082	G	A	G	A	0.04879	0.00117	0.3612	0.964457	15	90968030	0.017228	0.95	15	91511260	0.007255	8.75E-12
rs1292002	A	T	A	T	0.04879	0.007536	0.1575	0.128002	16	89497647	0.010274	0.46	16	89564055	0.007255	8.75E-12
rs1304175	C	T	C	T	0.058269	-0.00503	0.1072	0.204611	20	21486157	0.008386	0.55	20	21466795	0.009628	7.14E-10
rs1308513	C	T	C	T	0.076961	0.065457	0.9283	0.993036	3	72816032	0.040034	0.1	3	72865183	0.011867	4.43E-11
rs1316776	C	A	C	A	0.04879	-0.01232	0.6476	0.573247	5	79134784	0.006944	0.076	5	78430607	0.007324	1.35E-11
rs1317617	G	A	G	A	0.039221	-0.00482	0.7976	0.745437	10	79336832	0.00789	0.54	10	81096589	0.009813	3.21E-05
rs1359790	G	A	G	A	0.086178	0.013943	0.7201	0.736071	13	80143021	0.007798	0.074	13	80717156	0.007054	1.26E-34
rs1377807	C	G	C	G	0.04879	0.008356	0.3118	0.135927	17	4142146	0.009816	0.39	17	4045440	0.007255	8.75E-12
rs1381622	A	T	G	A	0.122218	0.000219	0.9043	0.018252	2	43466909	0.02487	0.99	2	43698028	0.01124	7.70E-28
rs1412234	C	T	C	T	0.039221	-0.01309	0.3229	0.153698	9	28410685	0.009535	0.17	9	28410683	0.007324	4.28E-08
rs1412830	C	T	C	T	0.039221	0.005871	0.6278	0.881604	9	22043613	0.010652	0.58	9	22043612	0.007395	5.67E-08
rs1421085	C	T	C	T	0.122218	0.003589	0.415	0.153698	16	53767042	0.009546	0.71	16	53800954	0.006743	1.02E-73
rs1426371	G	A	G	A	0.04879	-0.00684	0.7389	0.45341	12	1.08E+08	0.006836	0.32	12	1.09E+08	0.007255	8.75E-12
rs1516728	A	T	A	T	0.029559	-0.00758	0.759	0.424111	3	1.86E+08	0.006809	0.27	3	1.86E+08	0.007395	3.20E-05
rs1531583	T	G	T	G	0.122218	-0.03882	0.0458	0.019452	4	751184	0.024763	0.12	4	744972	0.015878	6.95E-15
rs1561927	C	T	C	T	0.039221	-0.0197	0.2686	0.056196	8	1.29E+08	0.014496	0.17	8	1.3E+08	0.007324	4.28E-08
rs1562396	G	A	G	A	0.058269	-0.00922	0.3186	0.387368	7	1.31E+08	0.006976	0.19	7	1.3E+08	0.007186	2.57E-16
rs1575972	T	A	T	A	0.09531	0.002409	0.9671	0.962056	9	22301093	0.018327	0.9	9	22301092	0.018561	1.41E-07
rs1580278	C	A	C	A	0.039221	-0.0035	0.4728	0.419549	4	1.03E+08	0.006953	0.62	4	1.04E+08	0.004906	6.50E-16
rs1701331	G	A	G	A	0.10436	-0.0129	0.0313	0.111671	3	23468553	0.010788	0.23	3	23510044	0.018394	6.99E-09
rs1708302	C	T	C	T	0.09531	0.008128	0.5124	0.963016	7	28159058	0.017166	0.64	7	28198677	0.00699	1.22E-42
rs1716848	T	C	T	C	0.067659	0.007724	0.1808	0.465418	7	14858657	0.006787	0.26	7	14898282	0.00712	1.02E-21
rs1726117	T	C	T	C	0.039221	-0.00512	0.5172	0.613833	5	52495391	0.006937	0.46	5	51791225	0.007395	5.67E-08
rs1752212	T	G	T	G	0.039221	0.012074	0.4742	0.388569	14	32833676	0.0069	0.08	14	33302882	0.004906	6.50E-16
rs1768407	G	C	G	C	0.039221	-0.00978	0.7403	0.976945	18	57008153	0.021177	0.64	18	54675384	0.007324	4.28E-08
rs1768900	G	A	G	A	0.039221	0.003209	0.5329	0.962056	8	10117314	0.016705	0.85	8	9974824	0.004906	6.50E-16
rs177045	G	A	G	A	0.067659	-0.01136	0.3155	0.601825	10	69561523	0.006991	0.1	10	71321279	0.007186	2.37E-21
rs1777281	G	A	G	A	0.076961	0.000693	0.9151	0.990634	8	1.28E+08	0.035767	0.98	8	1.29E+08	0.014176	2.83E-08
rs1779151	A	G	A	G	0.09531	-0.01166	0.9317	0.939962	9	79290675	0.014235	0.41	9	81905590	0.011545	7.56E-17
rs1783541	T	C	T	C	0.058269	0.003521	0.2035	0.167867	11	65527328	0.009215	0.7	11	65294799	0.007186	2.57E-16
rs1796330	G	C	G	C	0.04879	-0.01402	0.5711	0.719741	12	71129173	0.007623	0.066	12	71522953	0.004859	5.05E-24
rs1800961	T	C	T	C	0.165514	-0.00754	0.0353	0.01489	20	44413724	0.027641	0.78	20	43042364	0.017156	2.52E-22
rs1866525	C	T	C	T	0.058269	0.004925	0.1233	0.876321	18	38736759	0.010368	0.63	18	36278709	0.009628	7.14E-10
rs1903002	G	C	G	C	0.039221	-0.0006	0.5005	0.736551	4	88819743	0.007467	0.94	4	89740894	0.007395	5.67E-08
rs2028150	C	G	C	G	0.04879	0.010517	0.5979	0.789385	2	65427878	0.008139	0.2	2	65655012	0.007324	1.35E-11
rs2102278	G	A	G	A	0.039221	0.001326	0.3186	0.591499	4	51952498	0.007064	0.85	4	52818664	0.007395	5.67E-08
rs2189301	G	A	G	A	0.04879	0.008121	0.8716	0.988233	17	37703678	0.030761	0.79	17	36063685	0.012092	2.73E-05
rs2197973	T	C	T	C	0.039221	0.01135	0.5375	0.725744	12	95534784	0.007693	0.14	12	95928560	0.007395	5.67E-08
rs2237897	C	T	C	T	0.207014	0.001222	0.9543	0.681556	11	2837316	0.007263	0.87	11	2858546	0.016598	5.28E-36
rs2238689	C	T	C	T	0.039221	-0.00416	0.4177	0.550432	19	45675403	0.006954	0.55	19	46178661	0.004906	6.50E-16

rs2249105	A	G	A	G	0.09531	0.003178	0.6343	0.663064	2	65060762	0.007093	0.65	2	65287896	0.011545	7.56E-17
rs2272163	C	A	C	A	0.039221	-0.00594	0.6184	0.560999	3	77622570	0.006923	0.39	3	77671721	0.007395	5.67E-08
rs2280141	T	G	T	G	0.04879	0.00474	0.5161	0.381604	10	1.22E+08	0.007118	0.51	10	1.24E+08	0.007324	1.35E-11
rs2283164	A	G	A	G	0.076961	-0.02037	0.9468	0.942603	11	2557933	0.015002	0.17	11	2579163	0.016464	1.47E-06
rs231349	T	C	T	C	0.067659	0.011793	0.1024	0.073247	11	2651591	0.013288	0.37	11	2672821	0.011867	5.95E-09
rs231361	A	G	A	G	0.076961	-0.01146	0.2564	0.76537	11	2670270	0.007784	0.14	11	2691500	0.007054	5.14E-28
rs234853	G	A	G	A	0.076961	-0.00525	0.2482	0.383525	11	2829598	0.006961	0.45	11	2850828	0.009449	1.90E-16
rs243024	A	G	A	G	0.058269	-0.0045	0.4599	0.638809	2	60356530	0.006999	0.52	2	60583665	0.004813	4.93E-34
rs2431115	A	G	A	G	0.039221	0.000268	0.4015	0.53146	5	56552842	0.006788	0.97	5	55848669	0.007324	4.28E-08
rs2456530	T	C	T	C	0.058269	0.003803	0.1272	0.46926	15	52799356	0.006822	0.58	15	53091553	0.009628	7.14E-10
rs2465043	G	A	G	A	0.029559	-0.00825	0.6441	0.407061	6	51315967	0.006897	0.23	6	51180765	0.004954	1.21E-09
rs2581787	T	G	T	G	0.039221	0.023552	0.5634	0.709894	3	53093661	0.007491	0.0017	3	53127677	0.007395	5.67E-08
rs2642588	G	T	G	T	0.04879	0.012138	0.7016	0.848463	10	69706822	0.009339	0.19	10	71466578	0.007255	8.75E-12
rs2727301	T	C	T	C	0.039221	-0.01121	0.7538	0.977426	17	63887683	0.022647	0.62	17	61965043	0.007395	5.67E-08
rs2767036	C	A	C	A	0.039221	-0.00236	0.2908	0.248319	11	34960601	0.007883	0.76	11	34982148	0.007395	5.67E-08
rs2796441	G	A	G	A	0.067659	0.00254	0.5924	0.413545	9	81694033	0.006913	0.71	9	84308948	0.007186	2.37E-21
rs2800733	A	G	A	G	0.04879	-0.01527	0.7165	0.873199	6	1.27E+08	0.010575	0.15	6	1.27E+08	0.007324	1.35E-11
rs2820446	C	G	C	G	0.058269	-0.00065	0.7055	0.78122	1	2.2E+08	0.008233	0.94	1	2.2E+08	0.007255	4.79E-16
rs2850590	G	A	G	A	0.086178	0.004911	0.752	0.954851	9	1.36E+08	0.0162	0.76	9	1.39E+08	0.009363	1.72E-20
rs2851085	C	G	A	G	0.058269	0.012724	0.8983	0.376561	3	1.3E+08	0.006989	0.069	3	1.29E+08	0.009628	7.14E-10
rs2852537	G	T	G	T	0.029559	-0.00554	0.4216	0.377041	2	42980732	0.006994	0.43	2	43207872	0.004954	1.21E-09
rs2863814	A	C	A	C	0.076961	-0.01149	0.0442	0.237032	12	1.21E+08	0.008064	0.15	12	1.22E+08	0.016617	1.82E-06
rs286925	A	G	A	G	0.039221	0.009563	0.1815	0.594621	11	34621121	0.006947	0.17	11	34642668	0.009813	3.21E-05
rs2871946	C	T	C	T	0.039221	0.004265	0.1586	0.026897	18	55784913	0.021155	0.84	18	53452144	0.009813	3.21E-05
rs2908334	T	C	T	C	0.029559	-0.00049	0.6308	0.706532	7	15166614	0.007287	0.95	7	15206239	0.004954	1.21E-09
rs2972144	G	A	G	A	0.09531	0.00038	0.6385	0.900817	2	2.26E+08	0.01129	0.97	2	2.27E+08	0.00699	1.22E-42
rs3111316	A	G	A	G	0.04879	-0.0117	0.5885	0.821326	19	12927601	0.008844	0.19	19	13038415	0.007324	1.35E-11
rs3115960	G	C	G	C	0.029559	0.004174	0.37	0.443324	16	75482636	0.006811	0.54	16	75516534	0.007395	3.20E-05
rs3217860	G	A	G	A	0.04879	0.002521	0.258	0.574448	12	4289884	0.006891	0.71	12	4399050	0.007324	1.35E-11
rs329122	A	G	A	G	0.039221	-0.00353	0.4286	0.401297	5	1.35E+08	0.007027	0.62	5	1.34E+08	0.004906	6.50E-16
rs340874	C	T	C	T	0.067659	-0.00686	0.5555	0.427714	1	2.14E+08	0.006794	0.31	1	2.14E+08	0.007186	2.37E-21
rs3429898	T	C	T	C	0.039221	0.002797	0.4967	0.597262	6	40441504	0.00692	0.69	6	40409243	0.004906	6.50E-16
rs3445410	A	T	A	T	0.039221	-0.01831	0.7709	0.850624	20	52607055	0.009706	0.059	20	51223594	0.007324	4.28E-08
rs3458416	A	G	A	G	0.04879	0.003621	0.7598	0.64025	13	26202862	0.006975	0.6	13	26776999	0.007324	1.35E-11
rs348330	G	A	G	A	0.04879	0.019638	0.3605	0.665466	1	2.3E+08	0.007205	0.0064	1	2.3E+08	0.007255	8.75E-12
rs3485540	C	G	C	G	0.04879	-0.00323	0.2772	0.23487	17	42579393	0.008153	0.69	17	40731411	0.007255	8.75E-12
rs3535284	T	C	T	C	0.067659	0.000795	0.7878	0.79755	3	23414091	0.008594	0.93	3	23455582	0.009538	6.52E-13
rs3549723	C	T	C	T	0.039221	-0.00646	0.3169	0.353026	3	1.53E+08	0.007086	0.36	3	1.52E+08	0.007395	5.67E-08
rs3565495	C	T	C	T	0.029559	-0.00905	0.367	0.274015	4	1016289	0.007826	0.25	4	1010077	0.007395	3.20E-05
rs3589568	C	A	C	A	0.058269	-0.00025	0.678	0.956052	17	48982960	0.016636	0.99	17	47060322	0.007255	4.79E-16
rs3591346	C	T	C	T	0.058269	-0.00146	0.8291	0.92123	2	653575	0.012854	0.91	2	653575	0.009628	7.14E-10
rs3599910	T	C	T	C	0.04879	-0.00616	0.1547	0.244476	2	1.47E+08	0.00808	0.45	2	1.48E+08	0.009719	2.58E-07

rs3751837	T	C	T	C	0.039221	0.013592	0.22	0.229587	16	3533173	0.007997	0.089	16	3583173	0.007324	4.28E-08
rs3768321	T	G	T	G	0.086178	-0.00335	0.2004	0.105187	1	39570256	0.011215	0.77	1	40035928	0.007054	1.26E-34
rs3772071	T	C	T	C	0.04879	-0.00297	0.7135	0.78146	2	1.6E+08	0.008226	0.72	2	1.61E+08	0.007324	1.35E-11
rs3774723	G	A	G	A	0.067659	0.007536	0.8442	0.618396	3	63976663	0.007133	0.29	3	63962339	0.009538	6.52E-13
rs3802177	G	A	G	A	0.10436	0.001203	0.6851	0.548031	8	1.17E+08	0.006858	0.86	8	1.18E+08	0.006864	1.67E-52
rs3810291	A	G	A	G	0.04879	0.018924	0.673	0.295149	19	47065746	0.007523	0.012	19	47569003	0.007324	1.35E-11
rs3811978	G	A	G	A	0.058269	0.002744	0.1668	0.167627	5	52804655	0.009319	0.77	5	52100489	0.007255	4.79E-16
rs3826482	A	T	A	T	0.029559	-0.01511	0.5756	0.766811	17	3957062	0.007951	0.057	17	3860356	0.007395	3.20E-05
rs3887925	T	C	T	C	0.067659	-0.00909	0.5468	0.505283	3	1.87E+08	0.006864	0.19	3	1.87E+08	0.007186	2.37E-21
rs39328	T	C	T	C	0.039221	-0.00157	0.4334	0.402017	7	1.04E+08	0.007017	0.82	7	1.03E+08	0.007395	5.67E-08
rs4148856	C	G	C	G	0.04879	-0.00326	0.7814	0.872238	12	1.23E+08	0.010336	0.75	12	1.23E+08	0.009719	2.58E-07
rs4238013	C	T	C	T	0.058269	-0.00495	0.2087	0.071806	12	4266923	0.013429	0.71	12	4376089	0.007255	4.79E-16
rs4279506	G	C	G	C	0.058269	0.01326	0.6102	0.149856	7	23473277	0.008938	0.14	7	23512896	0.009628	7.14E-10
rs4281707	G	A	G	A	0.039221	0.006339	0.5442	0.76585	16	53468034	0.00802	0.43	16	53501946	0.004906	6.50E-16
rs429358	T	C	T	C	0.076961	-0.00356	0.8458	0.909462	19	44908684	0.01192	0.77	19	45411941	0.009449	1.90E-16
rs445084	G	A	G	A	0.029559	0.018123	0.3613	0.138329	11	2887524	0.010008	0.07	11	2908754	0.007395	3.20E-05
rs4457053	G	A	G	A	0.058269	-0.00873	0.3036	0.079491	5	77129124	0.012517	0.49	5	76424949	0.007186	2.57E-16
rs465002	T	C	T	C	0.10436	-0.011	0.7421	0.498079	5	56512648	0.006812	0.11	5	55808475	0.006926	1.33E-51
rs4688760	T	C	T	C	0.039221	0.001824	0.6842	0.223583	3	49943163	0.00828	0.83	3	49980596	0.007324	4.28E-08
rs4709746	C	T	C	T	0.058269	-0.00281	0.8676	0.917627	6	1.64E+08	0.012284	0.82	6	1.64E+08	0.009628	7.14E-10
rs4736819	T	C	T	C	0.039221	0.014627	0.5537	0.37512	8	41652396	0.00702	0.037	8	41509915	0.007395	5.67E-08
rs474513	A	G	A	G	0.039221	0.007831	0.5169	0.702209	6	1.6E+08	0.007323	0.28	6	1.61E+08	0.004906	6.50E-16
rs4776970	A	T	A	T	0.039221	-0.00758	0.6406	0.239914	15	67788548	0.0079	0.34	15	68080886	0.004906	6.50E-16
rs4810426	T	C	T	C	0.086178	0.0005	0.1064	0.401537	20	44373081	0.006976	0.94	20	43001721	0.011651	6.97E-14
rs4925109	A	G	A	G	0.04879	-0.02274	0.3164	0.858549	17	17758488	0.009705	0.019	17	17661802	0.007324	1.35E-11
rs4929965	A	G	A	G	0.067659	1.81E-05	0.3829	0.081892	11	2176056	0.012395	1	11	2197286	0.00712	1.02E-21
rs4930091	C	T	C	T	0.039221	-0.00295	0.7594	0.303794	11	2351126	0.007219	0.68	11	2372356	0.007395	5.67E-08
rs4946812	G	A	G	A	0.039221	0.008109	0.6743	0.841499	6	1.07E+08	0.009545	0.4	6	1.07E+08	0.004906	6.50E-16
rs4977213	C	T	C	T	0.04879	-0.00609	0.3749	0.413064	8	1.44E+08	0.006994	0.38	8	1.46E+08	0.007255	8.75E-12
rs523288	T	A	T	A	0.04879	0.00661	0.2377	0.182757	18	60181136	0.008801	0.45	18	57848369	0.007255	8.75E-12
rs5565356	A	C	A	C	0.039221	-0.0018	0.7321	0.902257	9	94239400	0.011341	0.87	9	97001682	0.007324	4.28E-08
rs5593549	C	T	C	T	0.086178	0.024326	0.0378	0.974304	8	41698218	0.021559	0.26	8	41552046	0.018731	2.11E-06
rs5615804	G	C	A	G	0.04879	-0.01603	0.6889	0.012248	12	1.21E+08	0.030229	0.6	12	1.21E+08	0.007255	8.75E-12
rs5633723	C	T	C	T	0.058269	-0.01755	0.5026	0.284822	4	1782676	0.007477	0.019	4	1784403	0.007255	4.79E-16
rs5732734	A	T	A	T	0.039221	-0.01065	0.782	0.884966	8	10951177	0.010616	0.32	8	10808687	0.009813	3.21E-05
rs5758223	A	G	A	G	0.039221	0.004101	0.7167	0.973823	22	41093916	0.02047	0.84	22	41489920	0.004906	6.50E-16
rs576674	G	A	G	A	0.04879	0.000975	0.1694	0.213497	13	32980164	0.008307	0.91	13	33554302	0.007255	8.75E-12
rs5843219	C	T	C	T	0.067659	0.004396	0.8811	0.909942	1	50790419	0.011943	0.71	1	51256091	0.009538	6.52E-13
rs6015034	T	C	T	C	0.067659	0.055259	0.858	0.008886	4	1.85E+08	0.036731	0.13	4	1.86E+08	0.009538	6.52E-13
rs601945	G	A	G	A	0.058269	-0.00481	0.1775	0.073727	6	32605638	0.013004	0.71	6	32573415	0.009628	7.14E-10
rs6027634	T	C	T	C	0.04879	-0.01351	0.1397	0.022334	17	64125944	0.023215	0.56	17	62203304	0.009719	2.58E-07
rs6063048	G	A	G	A	0.04879	-0.00928	0.7246	0.96926	20	46969925	0.019148	0.63	20	45598564	0.007324	1.35E-11

rs6070625	G	C	G	C	0.04879	-0.00138	0.5174	0.67243	20	58819573	0.007259	0.85	20	57394628	0.004859	5.05E-24
rs6167654	C	G	C	G	0.058269	-0.00435	0.1924	0.638329	17	67896391	0.006812	0.52	17	65892507	0.007255	4.79E-16
rs6185020	C	G	C	G	0.039221	0.008447	0.2768	0.121998	10	69561902	0.010524	0.42	10	71321658	0.007395	5.67E-08
rs6188111	G	A	G	A	0.04879	0.003138	0.838	0.98415	11	69229758	0.0261	0.9	11	68997225	0.007324	1.35E-11
rs6237048	A	G	A	G	0.039221	0.01664	0.2195	0.010327	5	53478680	0.034115	0.63	5	52774510	0.007395	5.67E-08
rs6248240	G	T	G	T	0.04879	0.002888	0.0819	0.052113	7	1.03E+08	0.014583	0.84	7	1.03E+08	0.012092	2.73E-05
rs6249236	A	G	A	G	0.04879	-0.00734	0.3081	0.513689	7	1.51E+08	0.006812	0.28	7	1.51E+08	0.007324	1.35E-11
rs6458354	C	T	C	T	0.04879	0.006812	0.289	0.148655	6	43846453	0.009643	0.48	6	43814190	0.007255	8.75E-12
rs6647770	T	C	T	C	0.086178	0.007251	0.967	0.871037	2	1.21E+08	0.010139	0.47	2	1.21E+08	0.018731	2.11E-06
rs6714881	A	G	A	G	0.086178	0.012841	0.9373	0.009126	2	1.57E+08	0.036244	0.72	2	1.58E+08	0.011758	1.16E-13
rs6723254	T	C	T	C	0.058269	-0.01688	0.207	0.186359	11	1.29E+08	0.008783	0.055	11	1.28E+08	0.007255	4.79E-16
rs6780171	A	T	A	T	0.131028	-0.00054	0.3138	0.248799	3	1.86E+08	0.007762	0.94	3	1.86E+08	0.008952	8.16E-49
rs6821438	A	G	A	G	0.039221	0.017877	0.5342	0.300672	4	94170760	0.007373	0.015	4	95091911	0.007324	4.28E-08
rs6884702	G	A	G	A	0.039221	0.007401	0.3932	0.534102	5	44682487	0.00699	0.29	5	44682589	0.007324	4.28E-08
rs7022807	G	A	G	A	0.039221	-0.00693	0.4014	0.257685	9	19067835	0.007781	0.37	9	19067833	0.004906	6.50E-16
rs702634	A	G	A	G	0.04879	0.019809	0.69	0.847022	5	53975590	0.009706	0.041	5	53271420	0.007255	8.75E-12
rs7078559	T	C	T	C	0.029559	0.007582	0.5783	0.547791	10	92164906	0.006806	0.27	10	93924663	0.007395	3.20E-05
rs7115753	A	G	A	G	0.039221	0.003646	0.4494	0.680596	11	45890462	0.00736	0.62	11	45912013	0.004906	6.50E-16
rs7124681	A	C	A	C	0.039221	0.005463	0.4097	0.273535	11	47508395	0.007815	0.48	11	47529947	0.004906	6.50E-16
rs7137225	C	T	C	T	0.076961	0.039046	0.0642	0.011047	17	31086001	0.032993	0.24	17	29413019	0.011867	4.43E-11
rs7178762	C	T	C	T	0.039221	-0.00277	0.4595	0.900336	15	63579093	0.011175	0.8	15	63871292	0.004906	6.50E-16
rs718314	G	A	G	A	0.04879	-0.00206	0.2532	0.637368	12	26300350	0.006922	0.77	12	26453283	0.007324	1.35E-11
rs7240767	C	T	C	T	0.039221	0.008609	0.3762	0.712776	18	7070643	0.00754	0.25	18	7070642	0.007395	5.67E-08
rs7249758	A	G	A	G	0.04879	0.00061	0.2039	0.03074	19	4948850	0.018843	0.97	19	4948862	0.009719	2.58E-07
rs7268899	C	G	C	T	0.058269	0.003214	0.2171	0.006964	14	79388542	0.041458	0.94	14	79932041	0.009628	7.14E-10
rs7280234	C	A	C	A	0.157004	0.00353	0.9231	0.875841	16	75200974	0.010548	0.74	16	75234872	0.013085	1.80E-33
rs7318723	G	A	G	A	0.076961	0.004282	0.0512	0.894092	22	50163478	0.011393	0.71	22	50604696	0.014176	2.83E-08
rs738408	T	C	T	C	0.04879	0.00364	0.2261	0.352546	22	43928850	0.007073	0.61	22	44324730	0.009719	2.58E-07
rs7519705	C	A	A	G	0.09531	0.014282	0.9571	0.127041	3	1.53E+08	0.010161	0.16	3	1.52E+08	0.016313	2.57E-09
rs7525392	C	T	C	T	0.04879	-0.00993	0.1909	0.022334	19	7240837	0.022491	0.66	19	7240848	0.007324	1.35E-11
rs7615045	G	A	G	A	0.113329	-0.00045	0.2388	0.267291	3	1.86E+08	0.007569	0.95	3	1.86E+08	0.020602	1.89E-08
rs7629630	A	T	A	T	0.04879	-0.0216	0.8567	0.980548	3	1.69E+08	0.025087	0.39	3	1.68E+08	0.009719	2.58E-07
rs7746418	A	C	A	C	0.10436	-0.0055	0.8363	0.918828	11	72749353	0.012589	0.66	11	72460398	0.009194	3.66E-30
rs7786482	A	G	A	G	0.076961	0.019782	0.9324	0.957493	12	97454997	0.016976	0.24	12	97848775	0.014176	2.83E-08
rs7802029	A	G	A	G	0.086178	0.00819	0.0517	0.056196	16	53724808	0.014795	0.58	16	53758720	0.014046	4.25E-10
rs7802555	C	G	C	G	0.04879	0.010302	0.851	0.973343	10	1.13E+08	0.021199	0.63	10	1.15E+08	0.009719	2.58E-07
rs7840347	G	C	G	C	0.058269	-0.00994	0.8956	0.85927	9	1.36E+08	0.009975	0.32	9	1.39E+08	0.012092	7.23E-07
rs7867635	C	T	C	T	0.039221	-0.00054	0.4123	0.880644	9	20241071	0.010421	0.96	9	20241069	0.007395	5.67E-08
rs7918400	T	C	T	C	0.058269	0.002583	0.4761	0.910663	10	1.13E+08	0.011554	0.82	10	1.15E+08	0.007255	4.79E-16
rs7976843	G	T	G	T	0.113329	0.010043	0.0225	0.982709	1	2.14E+08	0.026163	0.7	1	2.14E+08	0.022792	3.31E-07
rs7987740	T	C	T	C	0.039221	0.005196	0.6094	0.493276	13	1.09E+08	0.006794	0.44	13	1.1E+08	0.007395	5.67E-08
rs8010237	G	T	G	T	0.139762	-0.04435	0.9822	0.987512	11	2612947	0.030479	0.15	11	2634177	0.026644	7.79E-08

rs8010382	G	A	G	A	0.039221	-0.0036	0.4214	0.471662	14	91497378	0.006884	0.6	14	91963722	0.004906	6.50E-16
rs8017808	G	T	G	T	0.039221	0.009563	0.7431	0.391451	14	38379215	0.007115	0.18	14	38848419	0.007324	4.28E-08
rs8032939	C	T	C	T	0.058269	0.010348	0.246	0.558838	15	38541832	0.006796	0.13	15	38834033	0.007255	4.79E-16
rs8046545	G	A	G	A	0.039221	-0.026	0.3589	0.07757	16	28903896	0.012818	0.043	16	28915217	0.007395	5.67E-08
rs862320	C	T	C	T	0.039221	0.002839	0.5783	0.865994	16	69617963	0.009888	0.77	16	69651866	0.007324	4.28E-08
rs878521	A	G	A	G	0.058269	-0.00114	0.2451	0.353026	7	44216044	0.007116	0.87	7	44255643	0.007255	4.79E-16
rs914744	C	T	C	T	0.039221	-0.00521	0.3311	0.800192	9	1.37E+08	0.008741	0.55	9	1.4E+08	0.007395	5.67E-08
rs917195	C	T	C	T	0.04879	-0.00049	0.77	0.823967	7	30688836	0.008889	0.96	7	30728452	0.007255	8.75E-12
rs9200655	C	T	C	T	0.04879	0.001631	0.3317	0.369597	9	1.33E+08	0.007032	0.82	9	1.36E+08	0.007324	1.35E-11
rs9379084	G	A	G	A	0.10436	0.005478	0.8873	0.883766	6	7231610	0.010704	0.61	6	7231843	0.011545	7.88E-20
rs9494624	A	G	A	G	0.039221	-0.00441	0.2899	0.213497	6	1.37E+08	0.008345	0.6	6	1.37E+08	0.007324	4.28E-08
rs9505097	C	T	C	T	0.04879	-0.01684	0.7994	0.934678	6	7255417	0.013612	0.22	6	7255650	0.009719	2.58E-07
rs9537803	C	T	C	T	0.039221	-0.00671	0.2771	0.092699	13	57792500	0.011889	0.57	13	58366634	0.007324	4.28E-08
rs9563615	A	T	A	T	0.04879	0.001391	0.7101	0.512728	13	58503272	0.006789	0.84	13	59077406	0.007324	1.35E-11
rs9569864	C	T	C	T	0.04879	-0.00214	0.8247	0.513929	13	58391301	0.006828	0.75	13	58965435	0.007324	1.35E-11
rs963740	A	T	A	T	0.039221	-0.00812	0.7128	0.357349	13	50521959	0.007223	0.26	13	51096095	0.004906	6.50E-16
rs96844	G	A	G	A	0.039221	0.000856	0.2622	0.764649	5	56900777	0.007817	0.91	5	56196604	0.004906	6.50E-16
rs9687832	A	G	A	G	0.076961	-0.00157	0.1975	0.136647	5	56565768	0.009921	0.87	5	55861595	0.009449	1.90E-16
rs9860730	A	G	A	G	0.058269	0.004185	0.7036	0.273055	3	64715470	0.007622	0.58	3	64701146	0.007255	4.79E-16
rs9873618	G	A	G	A	0.067659	-0.00504	0.71	0.785303	3	1.71E+08	0.008315	0.54	3	1.71E+08	0.007186	2.37E-21
rs9957145	G	A	G	A	0.04879	0.006362	0.829	0.799232	18	59208996	0.008693	0.46	18	56876228	0.009719	2.58E-07

Supplementary Table 2d. List of SNPs for HbA1c and their effects on TEWL

SNP	effect_alle	other_allel	effect_alle	other_allel	beta.HbA1	beta.TEWL	eaf.HbA1c	eaf.TEWL	chr.TEWL	pos.TEWL	se.TEWL	pval.TEWL	chr.HbA1c	pos.HbA1c	se.HbA1c	pval.HbA1c
chr1:1003	G	A	G	A	-0.0155	0.006565	0.351672	0.720461	1	1E+08	0.007595	0.39	1	1.01E+08	0.0026	1.33E-09
chr1:1111	C	G	C	G	0.0191	0.011418	0.224544	0.241114	1	1.11E+08	0.007971	0.15	1	1.12E+08	0.0029	4.90E-11
chr1:1585	C	T	C	T	0.0758	-0.00027	0.233997	0.633285	1	1.59E+08	0.007117	0.97	1	1.59E+08	0.0029	3.75E-152
chr1:1586	T	A	T	A	0.0822	0.00351	0.266075	0.585255	1	1.59E+08	0.006892	0.61	1	1.59E+08	0.0028	2.76E-194
chr1:2036	T	C	T	C	0.0297	-0.01705	0.097996	0.018012	1	2.04E+08	0.025019	0.5	1	2.04E+08	0.004	1.54E-13
chr1:2070	C	T	C	T	0.0149	-0.00203	0.406155	0.804995	1	2.07E+08	0.008794	0.82	1	2.07E+08	0.0025	2.08E-09
chr1:3778	G	A	G	A	-0.0366	0.033692	0.244157	0.983429	1	3778232	0.025327	0.18	1	3694796	0.003	1.59E-34
chr10:113	G	A	G	A	0.021	0.000567	0.461621	0.9378	10	1.13E+08	0.013838	0.97	10	1.15E+08	0.0025	1.20E-17
chr10:690	T	C	T	C	-0.0444	-0.02067	0.031689	0.914986	10	69085335	0.012301	0.093	10	70845091	0.0067	3.27E-11
chr10:691	A	C	A	C	-0.1319	-0.03143	0.027959	0.987752	10	69172435	0.030194	0.3	10	70932191	0.0074	1.19E-70
chr10:693	C	T	G	T	-0.133	0.013024	0.042886	0.008165	10	69380983	0.037225	0.73	10	71138133	0.006	2.46E-109
chr10:694	C	G	C	G	0.0241	-0.00237	0.189859	0.938281	10	69417164	0.014072	0.87	10	71176920	0.0031	1.22E-14
chr10:694	A	G	A	G	0.0266	0.036233	0.082039	0.993276	10	69448568	0.042124	0.39	10	71208324	0.0045	2.92E-09
chr10:696	G	C	G	C	-0.0207	-0.02627	0.23849	0.879923	10	69601005	0.010575	0.013	10	71360761	0.0029	6.01E-13
chr10:792	T	C	T	C	0.0154	-0.00487	0.413878	0.2817	10	79205218	0.007528	0.52	10	80964975	0.0025	5.66E-10
chr11:100	A	G	A	G	-0.0577	0.013631	0.08216	0.879443	11	1.01E+08	0.010399	0.19	11	1E+08	0.0047	4.03E-35
chr11:929	C	T	C	T	0.0524	0.003376	0.288752	0.539625	11	92940662	0.007018	0.63	11	92673828	0.0027	5.52E-86
chr11:971	G	A	G	A	0.028	0.008594	0.268988	0.98439	11	9712626	0.027062	0.75	11	9734173	0.0028	6.46E-24
chr13:112	C	A	C	A	-0.0175	0.002341	0.274908	0.238713	13	1.13E+08	0.008053	0.77	13	1.14E+08	0.0027	1.41E-10
chr15:775	G	A	G	A	-0.0201	-0.00131	0.361933	0.452209	15	77581207	0.006775	0.85	15	77873549	0.0025	1.90E-15
chr16:283	T	C	T	C	0.0148	0.011676	0.424416	0.555476	16	28326722	0.006785	0.085	16	28338043	0.0025	2.11E-09
chr16:537	T	G	T	G	0.0217	-0.00446	0.393744	0.848943	16	53779455	0.009623	0.64	16	53813367	0.0025	3.01E-18
chr16:752	T	G	T	G	-0.0256	-0.01318	0.093808	0.819885	16	75213347	0.008723	0.13	16	75247245	0.0042	6.98E-10
chr16:815	T	C	T	C	-0.0169	-0.00035	0.301774	0.407781	16	81501185	0.007059	0.96	16	81534790	0.0027	1.87E-10
chr17:377	T	C	T	C	-0.0157	-0.00471	0.368934	0.247118	17	37736525	0.007868	0.55	17	36096515	0.0027	3.16E-09
chr17:594	A	C	A	C	-0.0274	-0.00514	0.235861	0.418828	17	59419609	0.006806	0.45	17	57496970	0.0029	6.74E-22
chr17:767	T	C	T	C	-0.0254	-0.00213	0.125109	0.04635	17	7674797	0.016202	0.9	17	7578115	0.0036	2.95E-12
chr17:767	G	C	G	C	-0.0243	-0.01014	0.249945	0.449568	17	7676154	0.006827	0.14	17	7579472	0.0028	3.29E-18
chr17:781	A	T	A	T	0.0171	0.000696	0.474589	0.602305	17	78134494	0.006868	0.92	17	76130575	0.0024	2.22E-12
chr17:781	G	T	G	T	-0.0718	0.002686	0.032866	0.993996	17	78134906	0.042845	0.95	17	76130987	0.0068	5.07E-26
chr19:128	G	T	G	T	-0.0215	-0.01789	0.158898	0.927474	19	12836245	0.013267	0.18	19	12947059	0.0034	1.33E-10
chr19:807	G	C	G	C	-0.0154	-0.00136	0.396983	0.423391	19	807442	0.006971	0.85	19	807442	0.0025	7.76E-10
chr2:1688	C	T	C	T	0.0716	0.001811	0.033323	0.73439	2	1.69E+08	0.007643	0.81	2	1.7E+08	0.0066	3.45E-27
chr20:337	C	T	C	T	0.0244	0.024879	0.154197	0.988713	20	33720469	0.03276	0.45	20	32308275	0.0034	6.55E-13
chr21:443	C	T	C	T	-0.0208	0.009015	0.197466	0.583093	21	44335477	0.006832	0.19	21	45755360	0.003	8.56E-12
chr22:301	T	C	T	C	0.0319	-0.00526	0.092326	0.123439	22	30194037	0.010516	0.62	22	30592069	0.0042	5.04E-14
chr22:320	T	G	T	C	-0.0331	0.016207	0.081392	0.012488	22	32079891	0.031091	0.6	22	32383419	0.0045	2.15E-13
chr22:370	G	A	G	A	0.0533	-0.01553	0.123571	0.950528	22	37075250	0.016083	0.33	22	37471290	0.0037	6.19E-46
chr22:370	G	A	G	A	-0.0378	0.007348	0.387768	0.661864	22	37098875	0.007168	0.31	22	37494915	0.0025	8.11E-52
chr3:1869	G	A	G	A	0.0203	0.000112	0.273182	0.902017	3	1.87E+08	0.011105	0.99	3	1.87E+08	0.0027	1.31E-13
chr4:1440	A	C	A	C	0.0314	-0.0029	0.317148	0.51633	4	1.44E+08	0.006787	0.67	4	1.45E+08	0.0026	4.25E-33

chr4:14427	A	G	A	G	-0.0166	0.004126	0.499459	0.556196	4	1.44E+08	0.006706	0.54	4	1.45E+08	0.0024	1.21E-11
chr4:62815	T	C	T	C	0.0172	0.003217	0.337336	0.018492	4	6281526	0.023625	0.89	4	6283253	0.0026	3.25E-11
chr6:10933	G	A	G	A	-0.0193	0.00602	0.295123	0.831892	6	1.09E+08	0.009332	0.52	6	1.1E+08	0.0027	6.05E-13
chr6:13513	G	A	G	A	-0.0313	-0.00448	0.194094	0.785543	6	1.35E+08	0.008224	0.59	6	1.35E+08	0.0031	5.44E-24
chr6:15312	C	A	C	A	0.016	0.004961	0.306299	0.34438	6	1.53E+08	0.007141	0.49	6	1.53E+08	0.0027	1.49E-09
chr6:25331	C	T	C	T	-0.0346	-0.00085	0.168321	0.977185	6	25331902	0.022282	0.97	6	25332130	0.0033	4.27E-26
chr6:72471	C	A	C	A	-0.0354	-0.00456	0.186168	0.954131	6	7247111	0.016612	0.78	6	7247344	0.0032	2.77E-29
chr7:44181	C	A	C	A	-0.0434	0.017711	0.034377	0.993996	7	44181320	0.044601	0.69	7	44220919	0.0067	8.24E-11
chr7:44276	C	T	C	G	-0.0423	0.012947	0.035996	0.008165	7	44276433	0.03622	0.72	7	44283661	0.0066	1.63E-10
chr7:77417	A	G	A	G	-0.0154	-0.00815	0.326637	0.854227	7	77417785	0.00967	0.4	7	77047102	0.0026	3.40E-09
chr7:99774	C	T	C	T	0.05	-0.06739	0.024579	0.006004	7	99774507	0.044606	0.13	7	99307972	0.008	3.86E-10
chr8:11720	G	T	G	T	-0.0264	-0.00523	0.124607	0.106148	8	1.17E+08	0.011237	0.64	8	1.18E+08	0.0036	4.34E-13
chr8:41367	G	T	G	T	-0.0236	-0.00865	0.125051	0.201729	8	41367937	0.00853	0.31	8	41225456	0.0037	1.41E-10
chr8:41691	G	A	A	T	-0.1524	-0.01363	0.034696	0.036984	8	41691393	0.018153	0.45	8	41543675	0.0067	2.69E-113
chr8:41801	T	G	T	G	-0.0456	-0.00475	0.210612	0.644332	8	41801405	0.006983	0.5	8	41658923	0.003	2.16E-52
chr9:13325	G	T	G	T	-0.0396	0.002558	0.060123	0.819164	9	1.33E+08	0.009005	0.78	9	1.36E+08	0.0049	5.94E-16
chr9:13345	G	C	G	C	-0.0202	0.003975	0.307614	0.29707	9	1.33E+08	0.007489	0.6	9	1.36E+08	0.0026	1.58E-14
chr9:13721	G	A	G	A	-0.0268	-0.01381	0.105337	0.849183	9	1.37E+08	0.009638	0.15	9	1.4E+08	0.0039	1.07E-11
chr9:22136	A	T	A	T	-0.0515	0.003621	0.136442	0.879683	9	22136490	0.010409	0.73	9	22136489	0.0036	3.81E-47
rs1000778	A	G	A	G	0.0174	-0.00831	0.255369	0.325168	11	61887833	0.007263	0.25	11	61655305	0.0028	3.53E-10
rs1006632	G	A	G	A	-0.0269	-0.01113	0.099397	0.587896	5	14800567	0.006818	0.1	5	14800676	0.0041	3.54E-11
rs1019488	G	A	G	A	0.0158	0.011678	0.448551	0.188521	2	2.26E+08	0.00846	0.17	2	2.27E+08	0.0025	1.30E-10
rs1043192	G	T	G	T	-0.0171	-0.00275	0.484831	0.488953	16	68805360	0.006744	0.68	16	68839263	0.0024	1.97E-12
rs1047912	C	T	C	T	0.021	-0.0163	0.301803	0.852305	12	50745079	0.009667	0.092	12	51138862	0.0027	3.18E-15
rs1052373	C	T	C	T	0.0171	0.001872	0.322463	0.310519	11	47333236	0.007262	0.8	11	47354787	0.0026	6.20E-11
rs1057037	A	T	A	T	0.0281	-0.01563	0.079312	0.981028	21	44313984	0.024448	0.52	21	45733867	0.0045	3.03E-10
rs1063729	G	A	G	A	0.0171	-0.00984	0.316389	0.46926	2	1.69E+08	0.006882	0.15	2	1.7E+08	0.0026	5.99E-11
rs1075925	T	C	T	C	-0.0191	-0.00638	0.32364	0.550192	9	1.08E+08	0.006666	0.34	9	1.1E+08	0.0026	1.94E-13
rs1079098	G	A	G	A	0.0315	-0.00733	0.057302	0.878722	19	17321868	0.010452	0.48	19	17432677	0.0052	1.69E-09
rs1087574	A	C	A	C	0.0383	0.005382	0.236983	0.753122	12	48124481	0.007974	0.5	12	48518264	0.0029	1.47E-40
rs1099871	A	C	A	C	0.0404	0.008849	0.0527	0.4695	10	69313959	0.006717	0.19	10	71073715	0.0054	6.54E-14
rs1099871	A	G	A	G	0.075	-0.00837	0.012003	0.961575	10	69316145	0.018206	0.65	10	71075901	0.0098	1.57E-14
rs1104245	G	A	G	A	-0.0254	0.007603	0.235359	0.628482	11	9640056	0.007228	0.29	11	9661603	0.0029	5.78E-19
rs1104275	T	C	T	C	0.0188	-0.00251	0.244558	0.85951	11	2128634	0.009889	0.8	11	2149864	0.0029	6.27E-11
rs1106630	A	G	A	G	-0.0251	0.029923	0.424376	0.985351	12	1.12E+08	0.027851	0.28	12	1.13E+08	0.0025	8.17E-24
rs1116836	A	G	A	G	0.0475	0.002534	0.112544	0.869116	12	48026930	0.010006	0.8	12	48420713	0.0038	2.74E-35
rs1118709	T	C	T	C	-0.0228	0.029521	0.115439	0.918828	10	92614846	0.012516	0.018	10	94374603	0.0038	3.20E-09
rs1119550	C	G	C	G	-0.0209	-0.01847	0.149206	0.947166	10	1.11E+08	0.01432	0.2	10	1.13E+08	0.0034	8.37E-10
rs1122864	A	C	A	C	0.0481	-0.0347	0.027217	0.991595	17	82820524	0.037732	0.36	17	80778400	0.0075	1.27E-10
rs1136425	G	A	G	A	0.0349	0.063217	0.059936	0.990874	19	17098335	0.036256	0.081	19	17209145	0.0052	2.13E-11
rs1145970	T	C	T	C	-0.0279	-0.03164	0.086905	0.01561	5	88589681	0.026762	0.24	5	87780432	0.0043	9.80E-11
rs1158743	C	T	C	T	-0.0229	0.006507	0.128724	0.204371	1	6571371	0.00838	0.44	1	6631431	0.0036	1.48E-10
rs1159741	A	G	A	G	0.0494	0.004896	0.063665	0.970701	10	69343219	0.019986	0.81	10	71102975	0.0049	6.93E-24

rs1160698 T	C	T	C	0.0175	0.017617	0.246632	0.979347	11	2822446	0.024084	0.46	11	2843676	0.0028	8.23E-10
rs1162767 C	A	A	T	-0.0399	0.028453	0.042573	0.043708	14	64841755	0.017002	0.094	14	65349806	0.0061	6.47E-11
rs1166764 G	T	A	G	0.0413	0.036986	0.050162	0.019212	19	44876534	0.025232	0.14	19	45381292	0.0058	9.88E-13
rs1166832 G	C	G	C	0.0203	0.058105	0.175578	0.986311	19	44895376	0.029215	0.047	19	45398633	0.0034	1.91E-09
rs1167228 C	T	C	T	0.0272	-0.04785	0.11275	0.990154	19	17158700	0.03492	0.17	19	17269510	0.0039	2.56E-12
rs1167239 A	G	T	G	0.0349	0.037043	0.063537	0.019452	19	44873841	0.025081	0.14	19	45372329	0.0052	2.20E-11
rs1170605 A	G	A	G	0.0335	0.013012	0.108977	0.96806	3	49026677	0.0197	0.51	3	49064110	0.004	2.74E-17
rs1171513 G	A	G	A	0.0251	-0.01234	0.093827	0.749039	3	50515435	0.007874	0.12	3	50552866	0.0042	2.50E-09
rs1175121 G	A	G	A	-0.0477	-0.04207	0.047429	0.994957	11	72838300	0.04634	0.36	11	72549345	0.0058	2.04E-16
rs1177318 C	T	C	T	-0.0324	0.034959	0.070899	0.982709	10	68316137	0.026166	0.18	10	70075894	0.0048	1.54E-11
rs1178545 G	A	C	G	0.0269	0.014894	0.143507	0.008886	8	1.17E+08	0.036707	0.68	8	1.18E+08	0.0035	2.29E-14
rs1181291 C	T	C	T	0.0349	-0.05821	0.067927	0.989673	10	69241218	0.033358	0.081	10	71000974	0.0049	6.71E-13
rs1181326 T	C	T	C	0.0319	-0.03192	0.053778	0.993036	16	88777595	0.041388	0.44	16	88844003	0.0054	4.23E-09
rs1181756 T	C	T	C	0.0247	0.006025	0.237133	0.961575	10	69191590	0.017967	0.74	10	70951346	0.0029	2.02E-17
rs1181908 C	T	C	T	0.0369	-0.00845	0.174015	0.884246	10	69298258	0.010826	0.43	10	71058014	0.0032	1.80E-30
rs1191381 A	G	G	C	0.0258	0.014944	0.130383	0.019212	22	37051006	0.023523	0.53	22	37439033	0.0037	2.02E-12
rs1196453 G	A	G	A	-0.0235	-0.00136	0.289283	0.822286	6	1.1E+08	0.009041	0.88	6	1.1E+08	0.0027	3.20E-18
rs1209599 C	T	C	T	-0.0379	0.004699	0.088928	0.907541	1	50926173	0.011903	0.69	1	51391845	0.0042	3.87E-19
rs1219444 A	G	A	G	-0.031	-0.0172	0.082833	0.989914	6	24866267	0.034539	0.62	6	24866495	0.0045	5.59E-12
rs1222113 G	A	G	A	0.0299	-0.00354	0.239424	0.498319	10	12211598	0.006655	0.59	10	12253597	0.0029	1.69E-25
rs1222279 A	G	A	G	0.0231	-0.00187	0.449878	0.446206	11	92933881	0.006768	0.78	11	92667047	0.0024	3.24E-21
rs1234214 C	CA	T	G	-0.0176	-0.00356	0.393029	0.448367	10	87938433	0.006911	0.61	10	89655242	0.0025	2.73E-12
rs1235520 A	G	A	G	0.0361	-0.01252	0.372544	0.856868	10	69329923	0.009684	0.2	10	71089679	0.0025	1.01E-46
rs1241601 C	T	C	T	-0.0312	-0.01785	0.158072	0.847502	10	69533948	0.009749	0.067	10	71293704	0.0034	1.62E-20
rs1252957 G	C	G	C	-0.0195	0.007473	0.215363	0.825408	6	1.1E+08	0.008951	0.4	6	1.1E+08	0.003	4.18E-11
rs1292002 G	C	G	C	0.0231	0.014388	0.10751	0.806676	16	84559765	0.008765	0.1	16	84593371	0.0039	2.61E-09
rs1293259 G	A	G	A	0.0361	0.002215	0.149225	0.804755	1	25369344	0.008417	0.79	1	25695835	0.0034	1.01E-25
rs1293762 C	A	C	A	-0.034	-0.00653	0.153737	0.967099	17	82681872	0.018976	0.73	17	80639748	0.0034	1.43E-23
rs1294990 T	C	T	C	-0.0279	0.00349	0.176325	0.821566	17	28897781	0.008874	0.69	17	27224799	0.0032	1.66E-18
rs1297155 C	T	C	T	-0.0465	-0.00041	0.02786	0.927233	19	32679562	0.013037	0.97	19	33170468	0.0075	7.13E-10
rs1297523 G	C	G	C	-0.0175	-0.01081	0.284916	0.917867	19	51275698	0.012711	0.39	19	51778952	0.0027	1.14E-10
rs1298332 T	C	T	C	0.0238	-0.01245	0.150107	0.737512	19	17136051	0.007605	0.1	19	17246861	0.0034	2.45E-12
rs1307749 C	T	C	T	0.0334	0.009598	0.10882	0.969501	3	49276545	0.020284	0.64	3	49313978	0.0039	2.34E-17
rs1309495 T	C	T	C	-0.0212	0.001719	0.20749	0.801153	3	23415589	0.008667	0.84	3	23457080	0.003	1.82E-12
rs1316450 G	A	G	A	0.0252	-0.01503	0.170311	0.914265	5	1.59E+08	0.011992	0.21	5	1.58E+08	0.0032	7.23E-15
rs1320945 C	T	C	T	-0.0246	0.022485	0.146977	0.987992	6	1.09E+08	0.031679	0.48	6	1.1E+08	0.0035	1.39E-12
rs13928 A	G	A	G	0.0193	-0.00095	0.473678	0.722142	7	44114181	0.007743	0.9	7	44153780	0.0024	2.57E-15
rs1497609 T	C	T	C	0.0246	-0.0013	0.353186	0.928674	4	1.44E+08	0.012574	0.92	4	1.45E+08	0.0026	6.12E-22
rs1499517 A	G	A	G	0.0301	0.005566	0.094997	0.961816	14	64762288	0.018136	0.76	14	65229006	0.0042	4.19E-13
rs1609812 G	A	G	A	0.0196	-0.00764	0.15402	0.495437	11	5225911	0.006877	0.27	11	5247141	0.0033	4.36E-09
rs163168 T	C	T	C	0.0209	-0.01121	0.219668	0.364073	11	2803115	0.007014	0.11	11	2824345	0.0029	1.08E-12
rs1685624 C	T	C	T	0.0759	0.007713	0.013462	0.725985	2	1.69E+08	0.007687	0.32	2	1.7E+08	0.0095	1.01E-15
rs1692624 C	T	C	T	-0.2836	0.062263	0.13058	0.993516	10	69333636	0.042911	0.15	10	71093392	0.0036	1.00E-200

rs1698841	T	C	T	C	0.0349	0.006878	0.045399	0.67243	22	30221596	0.007333	0.35	22	30617585	0.0055	1.88E-10
rs1712277	A	G	A	G	0.0171	-0.03261	0.229714	0.949328	14	22819744	0.015459	0.035	14	23288953	0.0029	3.91E-09
rs1715615	C	T	C	T	0.0381	-0.0193	0.079415	0.987272	11	270514	0.030221	0.52	11	270514	0.0045	2.05E-17
rs1722661	C	A	C	A	0.0356	-0.00282	0.222587	0.882085	12	48304257	0.010466	0.79	12	48698040	0.0029	3.01E-34
rs174570	C	T	C	T	-0.0256	0.002748	0.12827	0.423391	11	61829740	0.00655	0.67	11	61597212	0.0037	2.83E-12
rs1772846	C	G	C	G	0.0198	0.006687	0.294722	0.821806	22	30202563	0.00893	0.45	22	30598552	0.0027	1.54E-13
rs1775015	C	T	C	T	-0.0251	0.009894	0.314079	0.897214	22	37101986	0.011121	0.37	22	37498026	0.0026	1.44E-21
rs1864585	A	G	A	G	0.0188	0.013194	0.257345	0.030019	8	10875395	0.017892	0.46	8	10732905	0.0028	1.74E-11
rs1867971	A	G	A	G	-0.0305	0.006109	0.218623	0.597022	10	69385730	0.007008	0.38	10	71145486	0.0029	3.16E-25
rs1930261	A	G	A	G	0.0163	-0.01128	0.435856	0.536263	1	1.59E+08	0.006776	0.096	1	1.59E+08	0.0025	2.84E-11
rs1975233	A	C	A	C	-0.0146	-0.00948	0.449562	0.642891	4	1.54E+08	0.007043	0.18	4	1.54E+08	0.0025	3.55E-09
rs2026882	C	T	C	T	-0.0208	-0.01412	0.160296	0.863593	21	44902938	0.010161	0.16	21	46322853	0.0033	3.62E-10
rs2065500	A	G	A	G	-0.0259	-0.00677	0.157067	0.414745	9	22145695	0.006685	0.31	9	22145694	0.0034	1.05E-14
rs2071208	G	A	G	A	-0.0188	-0.00341	0.177788	0.240874	3	50122246	0.007982	0.67	3	50159679	0.0032	3.51E-09
rs2074311	A	G	A	G	-0.0164	-0.00236	0.413265	0.351825	11	17400313	0.007226	0.74	11	17421860	0.0025	3.61E-11
rs2188553	G	A	G	A	-0.0179	-0.01031	0.199837	0.990394	6	1.11E+08	0.035336	0.77	6	1.11E+08	0.003	4.25E-09
rs2232328	C	G	C	G	0.0422	0.003879	0.098332	0.978146	2	1.69E+08	0.023721	0.87	2	1.7E+08	0.0041	3.40E-25
rs2235321	G	A	G	A	-0.0431	-0.00273	0.384312	0.57877	22	37066886	0.006874	0.69	22	37462926	0.0025	1.04E-66
rs2238368	C	T	C	T	0.0156	-0.0055	0.461803	0.788425	16	120329	0.008381	0.51	16	170328	0.0024	1.91E-10
rs2269434	T	C	T	C	0.0165	0.001934	0.346476	0.31316	11	47338861	0.007249	0.79	11	47360412	0.0026	1.23E-10
rs2279008	T	C	T	C	0.0168	-0.01032	0.251735	0.674111	19	17172493	0.00728	0.16	19	17283303	0.0028	2.27E-09
rs2293941	G	A	G	A	0.0342	0.008401	0.218254	0.554035	13	27917061	0.006821	0.22	13	28491198	0.0029	2.84E-31
rs231362	A	G	A	G	0.0205	0.014513	0.476085	0.103746	11	2670241	0.011217	0.2	11	2691471	0.0025	7.28E-17
rs2384000	A	G	A	G	0.0242	0.006298	0.095157	0.194044	12	1.13E+08	0.007956	0.43	12	1.13E+08	0.0041	2.83E-09
rs2384937	T	C	T	C	-0.0225	-0.03169	0.120249	0.980067	16	15953968	0.023376	0.18	16	16047825	0.0037	1.43E-09
rs2390731	G	A	G	A	0.0508	-0.04627	0.072857	0.016811	2	1.69E+08	0.026166	0.077	2	1.7E+08	0.0046	4.70E-28
rs2394534	T	G	T	G	0.0372	-0.01311	0.250317	0.9378	10	69269546	0.014261	0.36	10	71029302	0.0028	3.56E-39
rs2413447	G	A	G	A	-0.0185	0.000837	0.445873	0.51537	22	37005491	0.006728	0.9	22	37401532	0.0025	4.66E-14
rs2515236	A	G	A	G	0.0156	-0.00766	0.354315	0.825648	8	94922679	0.00894	0.39	8	95934907	0.0025	8.89E-10
rs2526353	G	T	G	T	0.0228	0.001841	0.198501	0.634726	17	59911246	0.007035	0.79	17	57988607	0.0031	9.34E-14
rs2528374	C	G	C	G	0.0487	-0.01145	0.050802	0.907541	7	43936949	0.011741	0.33	7	43976548	0.0056	1.85E-18
rs2613518	A	G	A	G	-0.0162	-0.00785	0.411729	0.370797	17	78118773	0.007045	0.27	17	76114854	0.0025	5.04E-11
rs271043	G	A	G	A	0.0347	-0.00675	0.476876	0.309798	11	92993923	0.007367	0.36	11	92727089	0.0025	2.06E-45
rs2731073	A	G	A	G	-0.0286	0.011329	0.104308	0.044909	12	48525773	0.016604	0.5	12	48919556	0.004	6.83E-13
rs282595	G	A	G	A	0.0478	-0.0069	0.162185	0.897935	13	1.13E+08	0.011315	0.54	13	1.13E+08	0.0033	1.01E-47
rs2864146	T	C	T	C	0.0372	-0.00581	0.243855	0.045149	9	1.36E+08	0.016194	0.72	9	1.39E+08	0.0028	1.90E-39
rs2872068	A	G	A	G	-0.0203	-0.00202	0.161579	0.808117	3	1.71E+08	0.008633	0.82	3	1.71E+08	0.0033	1.00E-09
rs2887944	G	T	G	T	0.0148	0.010969	0.450193	0.168588	3	27716784	0.009294	0.24	3	27758275	0.0025	2.00E-09
rs2954038	C	A	C	A	0.016	0.010541	0.29975	0.28074	8	1.25E+08	0.007517	0.16	8	1.27E+08	0.0027	1.87E-09
rs2971681	A	G	A	G	0.0348	0.0146	0.163644	0.604467	7	44143588	0.006897	0.034	7	44183187	0.0033	1.43E-26
rs2998005	A	G	A	G	0.0203	-0.00416	0.152799	0.227666	10	69040867	0.008123	0.61	10	70800623	0.0034	1.74E-09
rs3217833	A	G	A	G	0.0217	-0.00603	0.151202	0.251201	12	4284308	0.007965	0.45	12	4393474	0.0034	1.33E-10
rs3217843	G	T	G	T	0.0216	-0.00928	0.143298	0.848223	12	4286699	0.009455	0.33	12	4395865	0.0035	6.81E-10

rs3217863	C	T	C	T	0.0287	-0.00238	0.074674	0.706052	12	4290330	0.007516	0.75	12	4399496	0.0047	7.31E-10
rs3447934	G	A	G	A	-0.0241	0.004619	0.116508	0.76537	17	28997922	0.008267	0.58	17	27324940	0.0038	1.52E-10
rs3482381	G	A	G	A	0.0345	0.000802	0.102471	0.975745	3	49712543	0.022127	0.97	3	49749976	0.0041	2.16E-17
rs3503188	C	A	C	A	0.0196	0.002079	0.187186	0.982229	12	50383790	0.024595	0.93	12	50777573	0.0032	4.89E-10
rs3516979	C	T	C	T	0.0329	0.051701	0.06389	0.987272	11	64263769	0.030231	0.087	11	64031241	0.005	5.38E-11
rs3573282	C	A	C	A	0.0218	0.007192	0.162655	0.986551	17	46755851	0.028947	0.8	17	44833217	0.0033	6.55E-11
rs3608435	G	A	G	A	-0.0267	-0.00587	0.09242	0.963977	19	1079960	0.018493	0.75	19	1079959	0.0043	3.97E-10
rs3621174	T	C	T	C	0.0417	-0.00921	0.478929	0.554515	4	1.44E+08	0.006909	0.18	4	1.45E+08	0.0024	2.46E-65
rs3729931	G	A	G	A	-0.0234	0.005398	0.359383	0.92243	3	12585017	0.012606	0.67	3	12626516	0.0025	2.72E-20
rs3731211	T	A	T	A	0.022	0.005985	0.278913	0.205091	9	21986848	0.008456	0.48	9	21986847	0.0027	5.98E-16
rs3731620	G	A	G	A	-0.0274	0.025674	0.142974	0.941403	2	24039190	0.014371	0.074	2	24262060	0.0035	3.87E-15
rs3744802	G	A	G	A	-0.0156	-0.00022	0.462572	0.563641	17	78797518	0.006958	0.97	17	76793600	0.0025	2.29E-10
rs3751120	C	T	C	T	0.0285	-0.00403	0.068699	0.490394	11	64116981	0.006759	0.55	11	63884453	0.0046	8.16E-10
rs3757972	C	T	C	T	0.0206	0.000208	0.379636	0.505043	8	1.44E+08	0.00686	0.98	8	1.46E+08	0.0025	2.73E-16
rs3794738	C	T	C	T	-0.0539	-0.00858	0.078033	0.857589	17	78123212	0.009683	0.38	17	76119293	0.0046	4.28E-32
rs3811444	C	T	C	T	0.026	0.003563	0.332885	0.748319	1	2.48E+08	0.00786	0.65	1	2.48E+08	0.0026	9.77E-24
rs3813026	A	G	A	G	0.0498	-0.00713	0.070204	0.951489	17	78127447	0.015951	0.66	17	76123528	0.0047	7.76E-26
rs3842752	G	A	G	A	0.025	-0.02926	0.22752	0.957733	11	2159843	0.016625	0.078	11	2181073	0.0029	1.15E-17
rs3857286	C	T	C	T	-0.0159	0.008736	0.300748	0.341258	5	55567362	0.007137	0.22	5	54863190	0.0027	2.58E-09
rs3887417	G	A	G	A	0.0254	-0.00663	0.130804	0.188761	17	78257576	0.008768	0.45	17	76253657	0.0036	1.19E-12
rs4073729	G	A	G	A	0.0304	-0.01426	0.150868	0.713737	12	47943286	0.007442	0.055	12	48337069	0.0034	2.68E-19
rs4127080	A	C	A	C	0.0209	-0.00164	0.212175	0.81268	1	39336143	0.008722	0.85	1	39801815	0.003	3.77E-12
rs4127850	T	G	T	G	-0.0485	0.035883	0.04641	0.992555	10	68344810	0.038786	0.35	10	70104567	0.0059	1.62E-16
rs4130267	T	G	T	G	0.038	0.0026	0.091125	0.98391	9	1.33E+08	0.027516	0.92	9	1.36E+08	0.0043	6.27E-19
rs4135275	A	G	A	G	0.0273	-0.00125	0.202443	0.522094	3	12402345	0.006812	0.85	3	12443844	0.003	2.74E-19
rs4145930	G	A	G	A	0.0272	0.001915	0.110104	0.240874	10	69238982	0.007924	0.81	10	70998738	0.0038	1.22E-12
rs4148157	G	A	G	A	0.0275	0.005717	0.098321	0.75024	4	88099782	0.00792	0.47	4	89020934	0.0041	2.48E-11
rs4148796	A	G	A	G	0.0192	-0.00176	0.253474	0.660663	2	1.69E+08	0.007152	0.81	2	1.7E+08	0.0028	6.25E-12
rs4227	G	T	G	T	0.0174	0.002368	0.276619	0.231508	17	7587859	0.008149	0.77	17	7491177	0.0027	1.63E-10
rs4279506	G	C	G	C	-0.0152	0.01326	0.382513	0.149856	7	23473277	0.008938	0.14	7	23512896	0.0025	1.99E-09
rs4282786	G	A	G	A	-0.0255	-0.00422	0.261194	0.683477	1	2.14E+08	0.007314	0.56	1	2.14E+08	0.0028	4.85E-20
rs4420638	A	G	A	G	-0.0203	-0.00551	0.191028	0.895293	19	44919689	0.011151	0.62	19	45422946	0.0031	6.31E-11
rs4432643	C	T	C	T	0.0244	0.021388	0.139102	0.790106	3	1.71E+08	0.008498	0.012	3	1.7E+08	0.0035	6.10E-12
rs454139	T	C	T	C	0.0952	-0.00134	0.12218	0.81196	17	78130013	0.008757	0.88	17	76126094	0.0037	4.25E-146
rs4665630	C	T	C	T	0.0228	-0.01495	0.111498	0.011287	2	23675447	0.030791	0.63	2	23898317	0.0038	2.66E-09
rs4688992	C	G	C	G	0.0165	0.008605	0.330412	0.020413	4	6315193	0.024244	0.72	4	6316920	0.0026	2.21E-10
rs4731120	A	C	A	C	-0.0494	0.006708	0.080614	0.877041	7	1.24E+08	0.010254	0.51	7	1.23E+08	0.0045	1.10E-27
rs4812829	G	A	G	A	0.0221	-0.00174	0.158702	0.576129	20	44360627	0.006932	0.8	20	42989267	0.0033	2.59E-11
rs4889651	A	G	A	G	-0.0201	-0.00612	0.261869	0.157541	16	30884224	0.008464	0.47	16	30895545	0.0029	6.63E-12
rs4894799	G	A	G	A	0.0266	-0.00649	0.371809	0.223103	3	1.72E+08	0.0081	0.42	3	1.72E+08	0.0025	3.89E-26
rs5030915	A	G	A	G	-0.067	0.022812	0.022353	0.991595	10	69384500	0.036671	0.53	10	71144256	0.0083	5.41E-16
rs520161	T	C	T	C	-0.02	-0.00528	0.299578	0.23511	7	28171041	0.007954	0.51	7	28210660	0.0027	5.49E-14
rs5587333	C	T	C	T	0.0348	0.011377	0.102705	0.970941	3	49559622	0.020055	0.57	3	49597055	0.0041	1.02E-17

rs5604960	C	G	C	G	-0.029	0.020881	0.222255	0.986551	3	49547810	0.02945	0.48	3	49585243	0.003	1.53E-22
rs569805	A	T	A	T	0.0913	-0.00905	0.353966	0.042507	2	1.69E+08	0.016798	0.59	2	1.7E+08	0.0026	1.00E-200
rs5758922	T	C	T	C	0.0153	-0.00135	0.488752	0.82829	22	42764750	0.008859	0.88	22	43160756	0.0024	3.51E-10
rs5763560	C	T	C	T	-0.03	0.007438	0.093301	0.987512	22	29862778	0.029394	0.8	22	30221120	0.0042	1.19E-12
rs5774058	T	G	T	G	-0.0269	0.007065	0.238342	0.82829	3	1.71E+08	0.009128	0.44	3	1.71E+08	0.0029	4.86E-21
rs5805308	T	G	T	G	-0.0429	0.001517	0.071016	0.915226	7	44193014	0.012439	0.9	7	44232613	0.0047	1.30E-19
rs584007	A	G	A	G	-0.023	-0.00873	0.355942	0.577329	19	44913221	0.006813	0.2	19	45416478	0.0027	1.01E-17
rs6023895	A	G	A	G	-0.0174	-0.00643	0.234044	0.786263	7	1874714	0.008504	0.45	7	1914350	0.0029	2.52E-09
rs603424	G	A	G	A	0.0207	-0.00914	0.168952	0.885207	10	1E+08	0.010036	0.36	10	1.02E+08	0.0032	9.75E-11
rs6179107	T	C	T	A	-0.0586	0.030669	0.016173	0.013689	3	1.71E+08	0.028242	0.28	3	1.71E+08	0.0095	5.48E-10
rs6196107	A	G	A	G	0.04	-0.02815	0.04643	0.98391	13	1.13E+08	0.027068	0.3	13	1.13E+08	0.0059	8.55E-12
rs6257822	G	A	G	A	0.0326	-0.06107	0.074083	0.991114	9	1.33E+08	0.036744	0.097	9	1.36E+08	0.0047	5.85E-12
rs632057	T	G	T	G	-0.0199	-0.01029	0.371909	0.630163	6	1.4E+08	0.006839	0.13	6	1.4E+08	0.0025	2.82E-15
rs653178	C	T	C	T	0.0286	-0.02554	0.482445	0.013209	12	1.12E+08	0.028716	0.37	12	1.12E+08	0.0025	4.37E-31
rs6598057	C	T	C	T	0.024	-0.03115	0.156956	0.028098	11	251034	0.020866	0.14	11	251034	0.0034	1.17E-12
rs6686424	G	A	G	A	0.0537	-0.01004	0.021632	0.017291	1	2.14E+08	0.026163	0.7	1	2.14E+08	0.0084	1.96E-10
rs6709302	G	A	G	A	-0.0182	-0.00922	0.328303	0.977906	2	60500494	0.021924	0.67	2	60727629	0.0026	2.60E-12
rs6731171	T	A	T	A	0.0162	-0.0082	0.380959	0.288665	2	2.01E+08	0.007644	0.28	2	2.02E+08	0.0026	2.04E-10
rs6760053	C	G	C	G	-0.0144	-0.01431	0.454377	0.617195	2	1.11E+08	0.006969	0.04	2	1.12E+08	0.0024	3.66E-09
rs6772452	T	C	T	C	0.0277	0.000765	0.23245	0.016571	3	49003029	0.027111	0.98	3	49054692	0.0029	2.03E-21
rs6840504	C	T	C	T	0.0178	-0.00026	0.450645	0.64121	4	18203479	0.007128	0.97	4	18205102	0.0025	4.30E-13
rs6844670	A	G	A	G	0.0476	-0.0066	0.338219	0.428674	4	1.44E+08	0.006899	0.34	4	1.45E+08	0.0026	3.09E-77
rs6885904	G	A	G	A	-0.0225	0.004058	0.444817	0.542988	5	77143425	0.007002	0.56	5	76439250	0.0025	5.41E-20
rs6974831	A	G	A	G	0.0466	-0.00519	0.039353	0.955091	7	99533982	0.016491	0.75	7	99131605	0.0061	2.10E-14
rs7018475	T	G	T	G	0.0276	0.004759	0.255123	0.654419	9	22137686	0.007207	0.51	9	22137685	0.0028	3.44E-23
rs703143	G	A	G	A	0.0359	-0.00662	0.382476	0.566763	1	1.59E+08	0.006875	0.34	1	1.59E+08	0.0025	5.22E-46
rs7076989	A	G	A	G	-0.0154	-0.02179	0.332781	0.90658	10	3095280	0.01186	0.066	10	3137472	0.0026	3.21E-09
rs7081062	G	A	G	A	0.0271	-0.0023	0.374919	0.330211	10	1.13E+08	0.007245	0.75	10	1.15E+08	0.0025	5.49E-27
rs7090788	C	A	C	A	-0.0418	-0.00137	0.07742	0.626081	10	69453894	0.006963	0.84	10	71213650	0.0045	2.41E-20
rs7129781	T	C	T	C	0.0271	-0.00099	0.078073	0.824448	11	14890871	0.008815	0.91	11	14912417	0.0045	1.58E-09
rs7155671	C	T	C	T	0.0276	-0.01237	0.090616	0.969981	7	73440219	0.019456	0.52	7	72854549	0.0043	1.22E-10
rs7196626	G	A	G	A	-0.0241	-0.00247	0.243048	0.216859	16	68655516	0.008124	0.76	16	68689419	0.0028	2.72E-17
rs7255902	A	G	A	G	-0.0314	-9.81E-05	0.277207	0.587176	19	17128883	0.006926	0.99	19	17239693	0.0027	1.64E-30
rs7256794	T	C	T	C	0.0373	0.00328	0.037737	0.987272	19	12864794	0.030219	0.91	19	12975608	0.0063	3.79E-09
rs7274421	G	A	G	A	0.034	0.014549	0.09174	0.012728	9	77270084	0.029227	0.62	9	79919444	0.0042	1.11E-15
rs7274761	G	A	G	A	-0.0175	0.02056	0.218846	0.005524	5	34501273	0.044495	0.64	5	34507418	0.0029	2.83E-09
rs7278010	A	C	A	C	0.0518	-0.00609	0.090433	0.942603	2	23761964	0.015145	0.69	2	23984834	0.0043	1.30E-33
rs7279427	C	T	C	T	-0.0269	-0.00311	0.077252	0.810999	2	23669453	0.008528	0.72	2	23892323	0.0046	3.46E-09
rs7281422	C	T	C	T	0.0396	-0.00341	0.145421	0.158501	10	69240373	0.009294	0.71	10	71007677	0.0035	3.85E-30
rs7285197	G	C	G	C	0.036	0.003714	0.081199	0.672911	17	83060131	0.00708	0.6	17	81018007	0.0045	8.78E-16
rs7285432	G	A	G	A	0.0585	-0.00689	0.09563	0.969741	17	82746355	0.019838	0.73	17	80704231	0.0042	2.40E-44
rs7286283	C	T	C	T	-0.025	-0.01445	0.313088	0.719981	17	82963611	0.007576	0.056	17	80921487	0.0026	3.02E-21
rs7289783	C	T	C	T	0.0284	0.003235	0.094033	0.010567	17	78202506	0.032266	0.92	17	76200965	0.0042	1.22E-11

rs7297302	A	C	A	C	0.0194	-0.00465	0.166525	0.96806	19	3341453	0.019523	0.81	19	3341451	0.0033	3.20E-09
rs7301872	A	G	A	G	0.058	0.014679	0.051902	0.993276	2	1.69E+08	0.040671	0.72	2	1.7E+08	0.0054	6.45E-27
rs730497	G	A	G	A	0.1124	0.009388	0.177842	0.827329	7	44184122	0.008946	0.29	7	44223721	0.0032	1.00E-200
rs7305283	A	C	A	C	0.0231	0.024308	0.112976	0.985831	19	51657512	0.028764	0.4	19	52160765	0.0039	2.19E-09
rs730566	C	A	C	A	-0.0174	-0.00518	0.259618	0.383045	3	48445644	0.007001	0.46	3	48487048	0.0028	3.93E-10
rs7311287	T	C	T	C	-0.0382	-0.00633	0.045587	0.784102	12	48584516	0.008424	0.45	12	48978299	0.0057	1.58E-11
rs7337494	CT	C	C	T	-0.0568	-0.00038	0.021925	0.183958	17	78116884	0.00876	0.97	17	76115478	0.0081	1.78E-12
rs734312	G	A	G	A	0.0162	-0.00504	0.457357	0.204131	4	6301627	0.008358	0.55	4	6303354	0.0025	4.29E-11
rs7405821	G	A	G	A	0.0334	-0.0047	0.05724	0.982229	14	64755499	0.026197	0.86	14	65222217	0.0052	1.62E-10
rs7433381	C	T	C	T	-0.0382	-0.00155	0.156061	0.919068	11	72746442	0.012603	0.9	11	72457487	0.0034	8.52E-30
rs7515142	T	C	T	C	0.0565	-0.03819	0.015199	0.972863	12	6996714	0.021018	0.069	12	7104806	0.0093	1.26E-09
rs7572278	T	A	T	A	0.0282	0.008779	0.204031	0.832373	2	8422899	0.009208	0.34	2	8563029	0.003	9.86E-21
rs7594415	G	A	G	A	0.0366	0.000392	0.094609	0.936599	14	64815589	0.014151	0.98	14	65282307	0.0042	1.39E-18
rs7636294	G	C	G	C	0.0274	0.00813	0.123323	0.008165	3	49831371	0.038278	0.83	3	49868804	0.0038	3.30E-13
rs7682793	T	C	T	C	0.0329	-0.04312	0.054448	0.951729	1	1.08E+08	0.015615	0.0058	1	1.09E+08	0.0054	1.03E-09
rs769449	G	A	G	A	-0.0231	-0.00692	0.126903	0.923631	19	44906745	0.012974	0.59	19	45410002	0.0037	4.79E-10
rs7742267	T	C	T	C	0.0416	0.004196	0.03212	0.778578	12	1.23E+08	0.008189	0.61	12	1.23E+08	0.0068	1.06E-09
rs7760225	T	C	T	C	0.0362	0.003712	0.059579	0.899135	10	69351910	0.011251	0.74	10	71111666	0.0051	7.67E-13
rs7760783	T	C	T	C	-0.0401	0.012556	0.035342	0.868876	10	69262987	0.010136	0.22	10	71022743	0.0066	9.13E-10
rs7769970	C	T	C	T	-0.0379	0.017242	0.056842	0.962296	12	97451205	0.018122	0.34	12	97851002	0.0053	7.12E-13
rs7798781	G	T	G	T	-0.0289	-0.00225	0.078399	0.930596	4	1.57E+08	0.013551	0.87	4	1.58E+08	0.0046	3.28E-10
rs780094	T	C	T	C	0.0279	-0.00412	0.381035	0.451969	2	27518370	0.00683	0.55	2	27741237	0.0025	1.06E-28
rs7807030	A	C	A	C	-0.0572	0.005721	0.08862	0.961095	2	43628011	0.017638	0.75	2	43855150	0.0043	2.49E-40
rs7834392	C	T	C	T	0.0378	-0.01689	0.043671	0.954851	1	1.68E+08	0.016447	0.3	1	1.68E+08	0.0059	2.07E-10
rs7836908	G	A	G	A	-0.04	0.008239	0.03866	0.848943	16	194786	0.009767	0.4	16	244785	0.0063	2.18E-10
rs7843297	C	T	C	T	-0.0541	0.034095	0.055619	0.988713	9	22132577	0.032688	0.3	9	22132576	0.0054	1.33E-23
rs7847715	G	A	G	A	-0.0254	-0.03919	0.11059	0.004563	3	1.52E+08	0.048642	0.42	3	1.52E+08	0.0039	7.62E-11
rs7871226	C	T	T	G	-0.0335	0.011453	0.102893	0.023055	12	54205482	0.021184	0.59	12	54649978	0.004	5.89E-17
rs7923811	C	T	C	T	0.0239	-0.04435	0.136229	0.965658	3	1.72E+08	0.018508	0.017	3	1.72E+08	0.0036	2.43E-11
rs7948798	G	A	G	A	-0.0292	0.019773	0.074024	0.819645	11	1.19E+08	0.008943	0.027	11	1.19E+08	0.0045	1.10E-10
rs7970284	C	G	C	G	0.0487	-0.04402	0.046778	0.973823	17	77898604	0.021366	0.039	17	75894686	0.0058	2.49E-17
rs7984515	A	G	A	G	0.0288	-0.00732	0.196355	0.887128	13	32992027	0.010649	0.49	13	33566165	0.0031	4.96E-21
rs7985325	C	T	C	T	-0.0396	-0.01519	0.187998	0.190922	13	1.14E+08	0.00867	0.08	13	1.15E+08	0.0031	2.55E-36
rs8005181	T	C	T	C	0.0173	0.011441	0.326055	0.971182	2	27368937	0.019508	0.56	2	27591804	0.0026	4.20E-11
rs8059048	A	G	A	G	-0.021	0.001237	0.179865	0.363833	16	88714632	0.006811	0.86	16	88781040	0.0032	3.09E-11
rs8126070	A	G	A	G	-0.0199	-0.00485	0.176841	0.908501	20	8624657	0.01178	0.68	20	8605304	0.0032	5.73E-10
rs854793	G	A	G	A	0.0246	0.007134	0.227547	0.690682	17	18127210	0.007412	0.34	17	18030524	0.0029	1.44E-17
rs895636	C	T	C	T	0.0221	-0.00784	0.203145	0.586695	2	44961214	0.007027	0.26	2	45188353	0.003	1.96E-13
rs906220	A	G	A	G	0.1257	-0.05121	0.073092	0.021854	10	69300854	0.023478	0.029	10	71060610	0.0046	7.98E-166
rs9398642	C	T	C	T	-0.0217	-0.00429	0.139536	0.800913	6	1.22E+08	0.008411	0.61	6	1.22E+08	0.0035	5.33E-10
rs9438901	G	A	G	A	-0.0373	0.010531	0.141912	0.104227	1	25258485	0.011299	0.35	1	25584976	0.0035	2.96E-26
rs9577924	A	G	A	G	0.0378	-0.00287	0.259538	0.751681	13	1.14E+08	0.007779	0.71	13	1.15E+08	0.0028	8.39E-43
rs9579135	A	G	A	G	0.0227	-0.00143	0.299535	0.6561	13	27959601	0.007048	0.84	13	28533738	0.0027	1.71E-17

rs9590217 C	T	C	T	0.0163	0.002495	0.464168	0.517531	9	1.33E+08	0.006799	0.71	9	1.36E+08	0.0024	2.36E-11
rs9790301 T	C	T	C	0.0213	-0.0053	0.191058	0.479587	3	1.23E+08	0.006682	0.43	3	1.23E+08	0.0031	6.06E-12
rs9832454 G	T	A	G	0.0328	-0.00904	0.086239	0.038905	3	50180125	0.017115	0.6	3	50189531	0.0044	9.16E-14
rs9871778 C	T	C	T	-0.0217	0.059044	0.130645	0.008405	3	1.77E+08	0.036688	0.11	3	1.77E+08	0.0036	1.77E-09
rs9901502 T	C	T	C	-0.0348	-0.00099	0.356623	0.451249	17	83013225	0.006815	0.88	17	80971101	0.0025	2.13E-42
rs9921085 G	A	G	A	0.0554	-0.00372	0.184602	0.814121	9	1.33E+08	0.008722	0.67	9	1.36E+08	0.0032	4.58E-68

Supplementary Table 2e. List of SNPs for Insulin Resistance (HOMA-IR) and their effects on TEWL

SNP	effect_alle	other_allel	effect_alle	other_allel	beta.HOM	beta.TEWL	eaf.HOMA	eaf.TEWL	chr.TEWL	pos.TEWL	se.TEWL	pval.TEWL	chr.HOMA	pos.HOMA	se.HOMA_	pval.HOMA_IR
chr12:102	G	A	G	A	0.009	-0.00051	0.62	0.631364	12	1.02E+08	0.007118	0.94	12	1.02E+08	0.005	0.071861
chr2:2262	C	A	C	A	-0.01512	-0.00054	NA	0.892891	2	2.26E+08	0.010956	0.96	2	2.26E+08	0.0184	0.411225
chr6:3487	T	G	T	G	-0.0173	-0.01593	NA	0.316763	6	34877672	0.007252	0.028	6	34877672	0.02006	0.38846
rs2785980	T	C	T	C	0.02082	-0.00587	NA	0.762488	1	2.2E+08	0.00802	0.46	1	2.2E+08	0.01817	0.251859
rs4691380	C	T	C	T	-0.00337	0.002035	NA	0.666427	4	1.57E+08	0.007202	0.78	4	1.57E+08	0.01919	0.860762
rs4841132	A	G	A	G	-0.05486	-0.02347	NA	0.021854	8	9326086	0.023476	0.32	8	9326086	0.0284	0.053398
rs7607980	T	C	T	C	-0.07136	-0.02198	NA	0.988713	2	1.65E+08	0.030708	0.47	2	1.65E+08	0.02542	0.004997
rs780094	C	T	C	T	0.035	0.00412	0.62	0.548031	2	27518370	0.00683	0.55	2	27518370	0.004	2.13E-18

Supplementary Table 2f. List of SNPs for Systolic BP (SBP) and their effects on TEWL

SNP	effect_alle	other_allel	effect_alle	other_allel	beta.SBP	beta.TEWL	eaf.SBP	eaf.TEWL	chr.TEWL	pos.TEWL	se.TEWL	pval.TEWL	chr.SBP	pos.SBP	se.SBP	pval.SBP
chr1:1073	A	G	A	G	0.4161	-0.00697	0.3372	0.57781	1	10736490	0.006754	0.3	1	10736490	0.0345	1.83E-33
chr1:1748	A	G	A	G	-0.2039	-0.01184	0.4781	0.463737	1	1748780	0.006869	0.085	1	1748780	0.0338	1.62E-09
chr1:3798	T	C	T	C	0.196	0.015592	0.4106	0.301873	1	37983491	0.007602	0.04	1	37983491	0.0347	1.66E-08
chr10:112	T	C	T	C	0.2209	0.008164	0.2906	0.052354	10	1.13E+08	0.014522	0.57	10	1.13E+08	0.0354	4.41E-10
chr10:132	A	G	A	G	0.2524	-0.01922	0.7935	0.854227	10	1.33E+08	0.009741	0.049	10	1.33E+08	0.0341	1.37E-13
chr10:184	G	C	G	C	0.4531	0.002543	0.7071	0.029539	10	18439030	0.019178	0.89	10	18439030	0.0374	7.81E-34
chr10:804	T	C	T	C	0.1723	0.006433	0.4086	0.104467	10	80436193	0.010598	0.54	10	80436193	0.0269	1.64E-10
chr11:122	A	G	A	G	-0.1811	-0.00343	0.523	0.621278	11	1.23E+08	0.007024	0.63	11	1.23E+08	0.0267	1.21E-11
chr11:473	T	C	T	C	0.3127	-0.00896	0.5159	0.433718	11	47385041	0.006842	0.19	11	47385041	0.0327	1.23E-21
chr11:585	A	G	A	G	-0.2678	-0.01704	0.2175	0.168108	11	58590875	0.009186	0.064	11	58590875	0.0405	3.86E-11
chr12:124	A	G	A	G	-0.1727	0.008818	0.3295	0.154419	12	12474476	0.009472	0.35	12	12474476	0.0285	1.40E-09
chr12:501	A	G	A	G	0.2932	-0.00397	0.3506	0.086696	12	50116154	0.012188	0.74	12	50116154	0.0347	3.17E-17
chr12:576	A	G	A	G	-0.3025	-0.00183	0.8329	0.901777	12	57610139	0.011441	0.87	12	57610139	0.0417	4.26E-13
chr12:795	A	G	A	G	0.2499	0.009755	0.185	0.270653	12	79510878	0.007795	0.21	12	79510878	0.0363	5.83E-12
chr13:316	A	C	A	C	0.3258	-0.0032	0.4693	0.098703	13	31607820	0.011505	0.78	13	31607820	0.0266	1.80E-34
chr14:354	A	G	A	G	0.2118	-0.00327	0.4023	0.463737	14	35402011	0.006907	0.64	14	35402011	0.0331	1.58E-10
chr14:719	T	C	T	C	-0.2055	-0.00913	0.2515	0.207253	14	71999568	0.00832	0.27	14	71999568	0.0301	8.84E-12
chr15:400	A	C	A	C	0.1926	-0.00176	0.5181	0.324928	15	40029150	0.007355	0.81	15	40029150	0.0331	5.86E-09
chr15:486	A	G	A	G	-0.4312	0.041672	0.0949	0.013449	15	48610929	0.029463	0.16	15	48610929	0.0575	6.44E-14
chr15:747	A	C	A	C	-0.324	-0.00276	0.6516	0.191883	15	74785026	0.008763	0.75	15	74785026	0.0378	1.08E-17
chr16:247	T	C	T	C	-0.3316	-0.02046	0.1825	0.113112	16	24718909	0.01081	0.058	16	24718909	0.0446	1.04E-13
chr17:183	T	C	T	C	-0.2274	-0.01024	0.5569	0.759126	17	18318485	0.007964	0.2	17	18318485	0.0263	5.41E-18
chr17:592	C	T	C	T	-0.1846	0.000933	0.5749	0.132565	17	59299316	0.009744	0.92	17	59299316	0.033	2.32E-08
chr17:598	A	T	A	T	-0.2774	-0.00176	0.2771	0.384246	17	59880621	0.006921	0.8	17	59880621	0.045	7.01E-10
chr17:634	T	A	T	A	-0.2063	0.002736	0.629	0.334534	17	63476833	0.007275	0.71	17	63476833	0.0341	1.41E-09
chr17:727	T	C	T	C	0.2407	0.001474	0.5357	0.403458	17	7276955	0.006971	0.83	17	7276955	0.0351	6.74E-12
chr18:555	A	G	A	G	-0.2834	0.009273	0.8602	0.982949	18	55585157	0.025338	0.71	18	55585157	0.0392	5.03E-13
chr18:631	T	C	T	C	0.1747	-0.01238	0.6364	0.568684	18	63178651	0.006954	0.075	18	63178651	0.0283	7.19E-10
chr18:753	A	G	A	G	-0.1774	0.003811	0.5266	0.689721	18	75328421	0.007282	0.6	18	75328421	0.0261	1.13E-11
chr19:102	T	G	T	G	0.2156	-0.00512	0.3977	0.610231	19	10223987	0.0071	0.47	19	10223987	0.035	7.06E-10
chr19:114	T	G	T	G	-0.4121	-0.00282	0.4565	0.506484	19	11416089	0.006746	0.68	19	11416089	0.0329	4.32E-36
chr2:1121	A	G	A	G	0.1832	0.017433	0.6388	0.85951	2	1.12E+08	0.009648	0.071	2	1.12E+08	0.0282	8.72E-11
chr2:1576	A	T	C	G	-0.2424	0.002856	0.1229	0.066523	2	1.58E+08	0.013772	0.84	2	1.58E+08	0.0403	1.85E-09
chr2:1641	G	C	G	C	-0.3584	0.009404	0.5067	0.56172	2	1.64E+08	0.006947	0.18	2	1.64E+08	0.0344	1.90E-25
chr2:1821	A	T	A	T	-0.2755	-0.0067	0.2117	0.229827	2	1.82E+08	0.008062	0.41	2	1.82E+08	0.0328	4.85E-17
chr2:2067	C	T	C	T	-0.1924	-0.00566	0.4803	0.251201	2	20679060	0.007943	0.48	2	20679060	0.0324	2.94E-09
chr2:2178	A	G	A	G	0.2053	-0.01295	0.3689	0.378242	2	2.18E+08	0.007011	0.065	2	2.18E+08	0.0338	1.21E-09
chr2:2183	A	G	A	G	-0.1532	-0.00307	0.6249	0.649135	2	2.18E+08	0.007098	0.67	2	2.18E+08	0.0269	1.29E-08
chr2:2262	A	G	A	G	-0.2825	-0.00068	0.3475	0.099424	2	2.26E+08	0.011282	0.95	2	2.26E+08	0.0338	5.93E-17
chr2:2669	T	G	T	G	-0.5149	-0.00802	0.5692	0.280259	2	26692756	0.007348	0.27	2	26692756	0.0346	5.89E-50

chr2:3697!	C	A	C	A	-0.2062	0.019928	0.6686	0.033622	2	36975863	0.018171	0.27	2	36975863	0.0354	5.88E-09
chr2:4565:	T	G	T	G	0.2769	0.000534	0.239	0.0622	2	45651621	0.014152	0.97	2	45651621	0.041	1.42E-11
chr2:8620(	A	G	A	G	-0.2359	-0.01011	0.333	0.523775	2	86200698	0.006883	0.14	2	86200698	0.0362	6.80E-11
chr21:420!	A	G	A	G	0.1588	0.004337	0.4342	0.09294	21	42052039	0.011369	0.7	21	42052039	0.0265	2.07E-09
chr21:435:	A	G	C	G	-0.2418	-0.008	0.4052	0.225985	21	43578734	0.00804	0.32	21	43578734	0.0337	7.70E-13
chr3:1604!	T	C	T	C	-0.2361	-0.01162	0.4838	0.235351	3	1.6E+08	0.008079	0.15	3	1.6E+08	0.0329	7.37E-13
chr3:4198:	A	C	A	C	-0.4206	0.013403	0.0955	0.988233	3	41983305	0.030909	0.66	3	41983305	0.0592	1.25E-12
chr3:4560:	C	G	C	G	-0.1731	-0.00697	0.3978	0.086936	3	45602156	0.011883	0.56	3	45602156	0.0283	1.02E-09
chr3:4754:	T	C	T	C	-0.2714	-0.00639	0.2456	0.134726	3	47547159	0.009673	0.51	3	47547159	0.04	1.16E-11
chr3:4805(	T	C	T	C	-0.3319	-0.00414	0.256	0.147214	3	48050845	0.009508	0.66	3	48050845	0.0406	2.87E-16
chr4:1059!	C	T	C	T	0.3159	-0.00481	0.7315	0.337176	4	1.06E+08	0.007316	0.51	4	1.06E+08	0.0368	9.33E-18
chr4:1431‹	G	T	G	T	0.2669	-0.00112	0.5372	0.744957	4	1.43E+08	0.007401	0.88	4	1.43E+08	0.0345	1.07E-14
chr4:1687(	A	C	A	C	0.2082	-0.00618	0.3911	0.169068	4	1.69E+08	0.00908	0.5	4	1.69E+08	0.034	9.22E-10
chr4:2696(	T	C	T	C	-0.2065	-0.00131	0.4115	0.374159	4	2696637	0.007011	0.85	4	2696637	0.0268	1.35E-14
chr5:1087!	C	A	C	A	0.2124	0.002455	0.7287	0.089097	5	1.09E+08	0.011682	0.83	5	1.09E+08	0.0372	1.11E-08
chr5:1280:	A	T	A	T	-0.277	0.005563	0.8125	0.783141	5	1.28E+08	0.008131	0.49	5	1.28E+08	0.0388	9.42E-13
chr5:3283:	T	C	T	C	-0.43	-0.0267	0.4087	0.338377	5	32831833	0.007337	0.00027	5	32831833	0.0331	1.09E-38
chr6:1267:	C	T	C	T	-0.3614	-0.00099	0.583	0.435639	6	1.27E+08	0.006761	0.88	6	1.27E+08	0.0351	7.76E-25
chr6:2068(	A	C	A	C	0.2018	-0.00227	0.2637	0.338377	6	20686765	0.007258	0.75	6	20686765	0.0295	7.40E-12
chr6:2610‹	T	G	T	G	0.3513	-0.02704	0.1418	0.03194	6	26104404	0.019833	0.17	6	26104404	0.0483	3.33E-13
chr6:2717:	T	C	T	C	0.2089	-0.00784	0.1681	0.009846	6	27171269	0.034906	0.82	6	27171269	0.0351	2.69E-09
chr6:3213!	T	C	T	C	-0.2611	0.002562	0.3668	0.480788	6	32139362	0.006866	0.71	6	32139362	0.0335	6.19E-15
chr6:3419:	T	C	C	A	-0.3749	-0.01174	0.9498	0.009126	6	34197574	0.036234	0.75	6	34197574	0.0654	1.01E-08
chr6:4338:	A	G	A	G	0.3526	0.013186	0.3991	0.341499	6	43381477	0.007312	0.071	6	43381477	0.0347	2.63E-24
chr7:1315(	C	G	C	G	0.239	-0.00812	0.3679	0.460134	7	1.32E+08	0.006715	0.23	7	1.32E+08	0.0358	2.51E-11
chr7:2720(	C	T	C	T	0.6519	-0.00552	0.8778	0.347983	7	27206377	0.007036	0.43	7	27206377	0.0546	6.67E-33
chr7:4597(	G	C	G	C	0.3505	0.003803	0.5691	0.73487	7	45970501	0.007642	0.62	7	45970501	0.033	2.48E-26
chr8:1236(	T	C	T	C	-0.1918	-0.00138	0.7845	0.817483	8	1.24E+08	0.009006	0.88	8	1.24E+08	0.0322	2.46E-09
chr8:5077!	T	C	T	C	0.2518	0.001514	0.2063	0.248079	8	50778263	0.007886	0.85	8	50778263	0.0403	3.95E-10
chr8:7567!	A	C	A	C	0.2185	0.016664	0.5109	0.409462	8	75679645	0.006946	0.016	8	75679645	0.033	3.54E-11
chr9:1139‹	T	G	T	G	0.1708	0.010186	0.4946	0.620317	9	1.14E+08	0.007062	0.15	9	1.14E+08	0.0269	2.09E-10
chr9:2061:	T	C	T	C	-0.1843	-0.00825	0.3134	0.398175	9	20612517	0.006936	0.23	9	20612517	0.0291	2.36E-10
chr9:9241!	A	G	A	G	0.1884	-0.00905	0.6056	0.855427	9	92419346	0.009598	0.35	9	92419346	0.0275	7.78E-12
rs1006204	T	C	T	C	0.2352	0.000284	0.1473	0.170029	5	62258054	0.009212	0.98	5	62258054	0.0379	5.61E-10
rs1014987	T	C	T	C	0.2127	0.001989	0.3973	0.371758	14	99645618	0.00719	0.78	14	99645618	0.0342	4.93E-10
rs1018230	A	G	A	G	-0.1644	-0.01886	0.4315	0.411143	2	2.27E+08	0.006844	0.0059	2	2.27E+08	0.0264	4.58E-10
rs1018400	T	C	T	C	-0.2046	-0.00091	0.4314	0.115274	2	1.65E+08	0.010655	0.93	2	1.65E+08	0.0273	7.17E-14
rs1021606	A	G	A	G	-0.2386	-0.00861	0.8274	0.571085	7	30875648	0.006831	0.21	7	30875648	0.0382	4.40E-10
rs1025410	T	C	T	C	0.3459	-0.00188	0.2726	0.044188	7	1.52E+08	0.016421	0.91	7	1.52E+08	0.0374	2.11E-20
rs1025686	G	A	G	A	0.214	0.014472	0.7546	0.444524	18	50620757	0.006904	0.036	18	50620757	0.0389	3.78E-08
rs1034906	C	G	C	G	-0.2067	0.002778	0.1482	0.067483	16	4078852	0.013713	0.84	16	4078852	0.0373	2.89E-08
rs1036190	G	A	G	A	0.2165	0.002897	0.6023	0.271374	5	1.49E+08	0.007593	0.7	5	1.49E+08	0.034	1.89E-10

rs1036821	A	G	A	G	0.2143	0.007503	0.2954	0.522815	8	1.35E+08	0.006947	0.28	8	1.35E+08	0.0363	3.71E-09
rs1042392	A	T	A	T	0.1975	0.002042	0.1983	0.191883	19	45679046	0.008424	0.81	19	45679046	0.0328	1.81E-09
rs1047717	A	G	A	G	0.1845	0.015256	0.7439	0.933237	5	1.42E+08	0.013261	0.25	5	1.42E+08	0.03	7.45E-10
rs1073425	A	G	A	G	-0.3026	0.004378	0.6206	0.637128	11	17383292	0.007067	0.54	11	17383292	0.0359	3.82E-17
rs1077462	G	A	G	A	-0.481	-0.0255	0.6576	0.011047	12	1.11E+08	0.031029	0.41	12	1.11E+08	0.0537	3.14E-19
rs1078856	C	T	C	T	-0.2282	-0.00205	0.7503	0.345821	10	87844975	0.007144	0.77	10	87844975	0.0398	1.00E-08
rs1083027	T	A	T	A	0.259	0.005854	0.759	0.413545	11	89483257	0.007003	0.4	11	89483257	0.0425	1.10E-09
rs1084959	T	C	T	C	-0.1891	0.000188	0.8132	0.887608	12	934668	0.010921	0.99	12	934668	0.0346	4.53E-08
rs1085285	T	C	T	C	0.2287	0.000805	0.4153	0.275937	17	1454457	0.007574	0.92	17	1454457	0.0333	6.76E-12
rs1085714	T	A	T	A	-0.6298	-0.0028	0.7239	0.398655	4	80259918	0.007081	0.69	4	80259918	0.0372	3.05E-64
rs1092824	G	C	G	C	0.216	-0.00226	0.6014	0.716378	2	1.45E+08	0.007722	0.77	2	1.45E+08	0.0333	8.50E-11
rs1100075	T	G	T	G	-0.3584	0.011696	0.8665	0.813641	10	73710597	0.00865	0.18	10	73710597	0.0494	4.21E-13
rs1104059	T	G	T	G	-0.2779	0.003468	0.1304	0.215418	11	49857205	0.008285	0.68	11	49857205	0.041	1.24E-11
rs1106468	G	A	G	A	0.3775	-0.01468	0.9175	0.119837	12	1.1E+08	0.010421	0.16	12	1.1E+08	0.0684	3.40E-08
rs1106632	A	G	A	G	0.2464	-0.02939	0.4165	0.014409	12	1.12E+08	0.028512	0.3	12	1.12E+08	0.0362	1.00E-11
rs1110535	G	A	G	A	0.6003	-0.00055	0.8382	0.29635	12	89632746	0.007286	0.94	12	89632746	0.0443	7.71E-42
rs1115301	A	G	A	G	0.304	0.010801	0.1799	0.040346	6	96508261	0.016854	0.52	6	96508261	0.0449	1.25E-11
rs1115433	A	C	A	C	-0.2205	-0.00441	0.6055	0.472863	6	1.26E+08	0.006886	0.52	6	1.26E+08	0.0276	1.22E-15
rs1115909	A	G	A	G	-0.1777	-0.00534	0.5221	0.808357	14	74631610	0.008447	0.53	14	74631610	0.0266	2.49E-11
rs1116851	T	C	T	C	0.2472	-0.00417	0.2193	0.28194	12	48308282	0.007707	0.59	12	48308282	0.0396	4.47E-10
rs1117913	T	C	T	C	0.2053	-0.01527	0.1487	0.036744	9	19111189	0.017874	0.39	9	19111189	0.0375	4.45E-08
rs1122208	T	A	T	A	-0.2986	-0.00679	0.6594	0.030259	11	1.3E+08	0.019083	0.72	11	1.3E+08	0.0358	7.09E-17
rs1122925	T	C	T	C	-0.3524	0.003693	0.0986	0.122719	11	55362164	0.010283	0.72	11	55362164	0.0609	7.12E-09
rs1124886	A	G	A	G	-0.1726	0.004975	0.5811	0.884006	16	1315340	0.01044	0.63	16	1315340	0.0277	4.90E-10
rs1125683	A	G	A	G	0.2107	-0.02076	0.1956	0.013449	10	10798572	0.029476	0.48	10	10798572	0.0351	2.01E-09
rs1126344	A	G	A	G	-0.4016	-0.01133	0.1161	0.496878	11	69437232	0.006786	0.095	11	69437232	0.0583	5.77E-12
rs1133970	A	G	A	G	-0.5177	-0.00378	0.0827	0.111671	6	1.51E+08	0.010687	0.72	6	1.51E+08	0.0637	4.56E-16
rs1137442	T	G	T	G	-0.2173	0.006372	0.4792	0.099904	12	65979721	0.011408	0.58	12	65979721	0.0333	7.04E-11
rs1152620	C	A	A	T	0.5265	0.012153	0.9166	0.010327	2	42969554	0.034159	0.72	2	42969554	0.0638	1.54E-16
rs1159201	A	G	A	G	0.2092	0.008226	0.7406	0.825648	5	67015511	0.009015	0.36	5	67015511	0.03	2.90E-12
rs1159216	C	G	C	G	0.2318	0.00165	0.1955	0.163305	10	1.17E+08	0.009314	0.86	10	1.17E+08	0.0363	1.72E-10
rs1161744	A	G	A	G	0.3324	-0.00028	0.2889	0.10999	13	1.14E+08	0.010859	0.98	13	1.14E+08	0.0374	5.82E-19
rs1162256	T	C	T	C	-0.1756	-0.00694	0.3202	0.454371	14	77050805	0.006804	0.31	14	77050805	0.0283	5.80E-10
rs1163625	T	C	T	C	0.1914	0.000223	0.4702	0.487272	15	75946679	0.006735	0.97	15	75946679	0.0272	1.96E-12
rs1168868	C	G	C	G	-0.2222	-0.0056	0.2384	0.07757	2	1.21E+08	0.012819	0.66	2	1.21E+08	0.0368	1.55E-09
rs1178504	T	C	T	C	0.2385	-0.01101	0.2229	0.301873	8	38348724	0.00745	0.14	8	38348724	0.0314	2.93E-14
rs1187634	A	G	A	G	-0.2453	0.002483	0.7007	0.773535	18	51273621	0.008148	0.76	18	51273621	0.03	3.21E-16
rs1203421	T	G	T	G	-0.2434	0.00145	0.4093	0.284342	1	41909802	0.007594	0.85	1	41909802	0.0359	1.16E-11
rs1203575	C	T	C	T	-0.2125	0.00205	0.6445	0.310279	1	88866416	0.007371	0.78	1	88866416	0.0359	3.38E-09
rs1208844	A	C	A	C	-0.1631	-0.00491	0.6235	0.619837	1	2.18E+08	0.00703	0.49	1	2.18E+08	0.028	5.87E-09
rs1211663	A	G	A	G	0.1811	-0.02695	0.2801	0.021614	1	42600923	0.022828	0.24	1	42600923	0.0302	1.94E-09
rs1212578	G	C	G	C	-0.229	-0.0004	0.6523	0.181316	1	56151040	0.008966	0.96	1	56151040	0.0366	3.82E-10

rs1219424	T	C	T	C	-0.3776	-0.01599	0.1797	0.013929	6	1.34E+08	0.028483	0.57	6	1.34E+08	0.0607	4.83E-10
rs12321	C	G	C	G	-0.1946	0.004548	0.4127	0.473823	22	29057205	0.006973	0.51	22	29057205	0.0334	5.62E-09
rs1244917	T	C	T	C	0.3022	-0.00082	0.557	0.508646	16	75429114	0.006819	0.9	16	75429114	0.0334	1.35E-19
rs1250259	T	A	T	A	-0.4518	-0.0071	0.7447	0.118636	2	2.15E+08	0.010064	0.48	2	2.15E+08	0.0745	1.33E-09
rs1250618	T	A	T	A	-0.381	0.011699	0.8789	0.154419	4	85818748	0.00947	0.22	4	85818748	0.0524	3.63E-13
rs1263557	A	C	A	C	0.282	0.007964	0.249	0.414745	3	14913042	0.006937	0.25	3	14913042	0.0389	3.99E-13
rs1263886	G	A	G	A	0.258	-0.00337	0.7348	0.521854	3	1.7E+08	0.00683	0.62	3	1.7E+08	0.0373	4.80E-12
rs1264966	A	T	A	T	-0.2194	0.000555	0.2258	0.504563	4	82975665	0.006867	0.94	4	82975665	0.0332	3.93E-11
rs1270539	A	G	A	G	0.7392	-0.00102	0.1907	0.148895	7	1.07E+08	0.009498	0.91	7	1.07E+08	0.0421	5.50E-69
rs1279912	T	G	T	G	0.3084	0.006374	0.2006	0.231748	11	16286917	0.007934	0.42	11	16286917	0.0419	1.75E-13
rs1290696	C	T	C	T	-0.2324	-0.00365	0.6175	0.261768	15	94768842	0.007723	0.64	15	94768842	0.0354	5.45E-11
rs1290964	A	G	A	G	0.1911	0.008381	0.4629	0.050432	15	85681339	0.015189	0.58	15	85681339	0.0264	5.01E-13
rs1291687	A	C	A	C	0.1922	0.017733	0.5868	0.979107	15	74260401	0.023676	0.45	15	74260401	0.0289	3.12E-11
rs1293798	A	G	A	G	-0.2313	0.002541	0.3849	0.29731	17	6576133	0.007454	0.73	17	6576133	0.0355	6.91E-11
rs1295205	T	C	T	C	-0.244	0.001312	0.4188	0.254803	17	2079496	0.007677	0.86	17	2079496	0.0351	3.73E-12
rs1295564	C	G	C	G	0.2931	0.00047	0.8239	0.688761	18	771046	0.007455	0.95	18	771046	0.0362	5.75E-16
rs1314152	A	G	A	G	-0.1948	-0.00602	0.4113	0.260086	4	48787252	0.007891	0.45	4	48787252	0.0357	4.70E-08
rs1323440	A	G	A	G	-0.1928	0.012979	0.4645	0.342939	7	1.31E+08	0.007233	0.073	7	1.31E+08	0.0326	3.27E-09
rs1328223	A	G	A	G	0.1764	-0.00118	0.527	0.483429	8	1.42E+08	0.006816	0.86	8	1.42E+08	0.0272	9.43E-11
rs1343114	A	C	A	C	0.2055	-0.0083	0.3773	0.743756	2	55966530	0.007631	0.28	2	55966530	0.0339	1.35E-09
rs1407256	G	A	G	A	-0.2925	0.006717	0.8337	0.539866	20	32625845	0.006811	0.32	20	32625845	0.0503	5.96E-09
rs1415293	A	T	A	T	0.1745	-0.00193	0.6994	0.771614	1	2.2E+08	0.008132	0.81	1	2.2E+08	0.0288	1.43E-09
rs1440371	A	G	A	G	0.217	0.00407	0.2827	0.189241	15	66648746	0.008502	0.63	15	66648746	0.0363	2.30E-09
rs1449386	T	G	T	G	-0.2088	0.008237	0.3968	0.102546	3	85569929	0.010706	0.44	3	85569929	0.0276	4.29E-14
rs1490938	TTG	T	A	G	-0.2276	0.008046	0.5535	0.350624	11	1.07E+08	0.007322	0.27	11	1.07E+08	0.0332	7.50E-12
rs159960	G	A	G	A	-0.2382	-8.84E-05	0.4883	0.782421	1	8416368	0.008126	0.99	1	8416368	0.0342	3.49E-12
rs1692792	C	T	C	T	-0.2817	0.008094	0.8471	0.278338	9	1.25E+08	0.00766	0.29	9	1.25E+08	0.0457	7.18E-10
rs1709658	C	T	C	G	-0.2831	-0.0094	0.7962	0.227906	14	98168761	0.00808	0.24	14	98168761	0.0409	4.32E-12
rs1711464	G	T	G	T	0.4605	-0.0038	0.8951	0.381844	10	1.03E+08	0.007005	0.59	10	1.03E+08	0.0571	7.64E-16
rs1760876	C	T	C	T	-0.4971	-0.02922	0.8708	0.009126	17	46935905	0.034426	0.4	17	46935905	0.0507	1.09E-22
rs1767925	A	G	A	G	0.3393	-0.00182	0.869	0.945725	14	1.03E+08	0.015089	0.9	14	1.03E+08	0.0398	1.59E-17
rs1780435	T	C	T	C	0.1773	0.00364	0.5441	0.341979	8	1.28E+08	0.007261	0.62	8	1.28E+08	0.0267	2.99E-11
rs1801253	G	C	G	C	0.2724	0.008853	0.7099	0.262488	10	1.14E+08	0.007837	0.26	10	1.14E+08	0.0376	4.19E-13
rs1815614	A	G	A	G	0.2405	-0.0113	0.5401	0.552113	1	9381779	0.006913	0.1	1	9381779	0.0342	2.14E-12
rs1840221	T	C	T	C	0.216	-0.01726	0.2593	0.128722	19	4932267	0.010148	0.089	19	4932267	0.0304	1.14E-12
rs1860509	G	T	G	T	0.268	-0.00548	0.7757	0.221902	7	1.4E+08	0.008264	0.51	7	1.4E+08	0.0442	1.31E-09
rs1887320	A	G	A	G	0.3107	0.005138	0.4655	0.541787	20	10985350	0.006879	0.46	20	10985350	0.0323	6.00E-22
rs1995624	T	C	T	C	-0.3412	0.004399	0.1252	0.354707	10	94827994	0.007029	0.53	10	94827994	0.057	2.20E-09
rs2001337	A	G	A	G	0.3192	-0.00092	0.3599	0.239433	8	10393644	0.008129	0.91	8	10393644	0.028	5.13E-30
rs2012071	A	G	A	G	-0.1575	-0.00467	0.4827	0.850144	6	1.12E+08	0.009446	0.62	6	1.12E+08	0.0267	3.60E-09
rs2048240	C	T	C	T	0.2328	-0.00194	0.5273	0.396013	2	28417460	0.007059	0.78	2	28417460	0.033	1.83E-12
rs2071382	T	C	T	C	0.4092	0.002914	0.4353	0.180836	15	90884967	0.008652	0.74	15	90884967	0.0345	1.55E-32

rs2075066	A	G	A	G	0.2507	-0.01047	0.1851	0.185879	7	44225580	0.008712	0.23	7	44225580	0.0458	4.40E-08
rs2084600	G	A	G	A	-0.2336	-0.01778	0.5568	0.086696	11	10330748	0.01151	0.12	11	10330748	0.0332	1.97E-12
rs2107595	A	G	A	G	0.4172	0.00132	0.1847	0.332373	7	19009765	0.007365	0.86	7	19009765	0.0441	3.25E-21
rs2139629	A	G	A	G	0.2007	0.001671	0.7722	0.669068	2	50400459	0.007158	0.82	2	50400459	0.0324	5.89E-10
rs2179490	A	G	A	G	-0.2307	0.023121	0.3692	0.068684	1	1.69E+08	0.013652	0.09	1	1.69E+08	0.0342	1.56E-11
rs2192768	C	T	C	T	-0.2313	-0.00342	0.4407	0.339097	2	40298792	0.00719	0.63	2	40298792	0.0347	2.58E-11
rs2286525	G	A	G	A	0.353	-0.00893	0.732	0.399616	17	61394762	0.006965	0.2	17	61394762	0.0395	3.65E-19
rs2290273	T	C	T	C	-0.1511	-0.00324	0.4886	0.588136	15	85000819	0.006893	0.64	15	85000819	0.0272	2.93E-08
rs2300481	T	C	T	C	0.1857	0.013121	0.3787	0.161864	2	66555335	0.009186	0.15	2	66555335	0.027	6.03E-12
rs2306363	T	G	T	G	-0.3618	0.012567	0.1937	0.252882	11	65638129	0.007743	0.1	11	65638129	0.0432	5.08E-17
rs2476970	A	G	A	G	-0.3872	0.034049	0.1016	0.007925	10	1.01E+08	0.037706	0.37	10	1.01E+08	0.0551	2.03E-12
rs2487903	T	C	T	C	-0.1692	-0.00148	0.401	0.319404	1	2.1E+08	0.007483	0.84	1	2.1E+08	0.0272	4.82E-10
rs256904	A	T	A	T	-0.206	0.01098	0.2766	0.501921	5	56514478	0.006812	0.11	5	56514478	0.0295	3.02E-12
rs2581468	T	C	T	C	0.2435	-0.00862	0.8093	0.721662	15	99572263	0.007677	0.26	15	99572263	0.0356	7.84E-12
rs2643826	C	T	C	T	0.4106	-0.00431	0.4571	0.710615	3	27521497	0.007423	0.56	3	27521497	0.0366	2.87E-29
rs267539	A	G	A	G	-0.1784	-0.00205	0.4635	0.697406	3	37540761	0.007187	0.78	3	37540761	0.0264	1.40E-11
rs2707450	T	C	T	C	0.2125	0.005593	0.7357	0.595341	4	17940937	0.006892	0.42	4	17940937	0.0303	2.29E-12
rs2823139	A	G	A	G	0.25	0.002725	0.3392	0.229107	21	15204463	0.008109	0.74	21	15204463	0.0367	9.25E-12
rs3407044	A	G	A	G	-0.1971	0.00723	0.566	0.518972	5	1.48E+08	0.00681	0.29	5	1.48E+08	0.0263	6.44E-14
rs3429493	A	T	A	T	0.2079	0.004158	0.2063	0.097262	16	49850016	0.011625	0.72	16	49850016	0.0334	4.94E-10
rs3455689	T	G	T	G	0.2115	-0.00729	0.3107	0.009846	17	58749592	0.034918	0.83	17	58749592	0.0292	4.31E-13
rs3479779	A	G	A	G	-0.2473	-0.00601	0.4267	0.269452	5	78557262	0.00777	0.44	5	78557262	0.0333	1.07E-13
rs3486854	T	C	T	C	-0.2044	-0.00944	0.5701	0.25	10	74226399	0.007654	0.22	10	74226399	0.028	3.06E-13
rs35444	G	A	G	A	0.3671	-0.00973	0.6147	0.291787	12	1.15E+08	0.007503	0.19	12	1.15E+08	0.033	8.94E-29
rs356833	A	G	A	G	0.1719	0.006183	0.7496	0.776417	18	28743561	0.008135	0.45	18	28743561	0.0303	1.38E-08
rs357489	A	T	A	T	-0.2101	-0.00595	0.7092	0.658501	3	1.54E+08	0.007104	0.4	3	1.54E+08	0.0295	9.83E-13
rs3734345	TA	T	G	A	-0.3067	0.003218	0.8811	0.060038	6	1.18E+08	0.014319	0.82	6	1.18E+08	0.0513	2.34E-09
rs3740781	T	C	T	C	0.2255	0.00827	0.6604	0.307157	11	45234375	0.007299	0.26	11	45234375	0.0363	5.20E-10
rs3744010	A	G	A	G	-0.2759	-0.00338	0.292	0.115754	17	75844162	0.010689	0.75	17	75844162	0.0371	1.05E-13
rs3745348	T	C	T	C	0.2599	-0.0143	0.3421	0.378482	19	17101600	0.007103	0.044	19	17101600	0.0343	3.74E-14
rs3772840	T	C	T	C	-0.1846	0.001334	0.4362	0.664505	3	1.25E+08	0.007159	0.85	3	1.25E+08	0.0334	3.41E-08
rs3790604	A	C	A	C	0.6346	-0.00294	0.0813	0.292747	1	1.13E+08	0.007498	0.69	1	1.13E+08	0.0636	2.05E-23
rs3796592	C	T	C	T	0.2949	-0.01179	0.7958	0.208934	4	1.56E+08	0.008456	0.16	4	1.56E+08	0.042	2.24E-12
rs3802228	A	G	A	G	0.2144	0.003392	0.4325	0.653458	8	1.43E+08	0.006989	0.63	8	1.43E+08	0.0334	1.36E-10
rs3821843	G	A	G	A	0.3337	-0.01033	0.6923	0.536263	3	53523985	0.006701	0.12	3	53523985	0.0377	9.09E-19
rs3821964	T	C	T	C	-0.1676	-0.0016	0.6057	0.488953	4	95119553	0.006913	0.82	4	95119553	0.0271	6.22E-10
rs4109837	A	T	A	T	-0.2287	0.004095	0.302	0.298991	11	49350499	0.007576	0.59	11	49350499	0.0357	1.46E-10
rs414992	T	C	T	C	0.3128	0.007763	0.1365	0.133045	11	16872543	0.009972	0.44	11	16872543	0.0482	8.51E-11
rs42377	A	G	A	G	-0.2575	-0.0062	0.3172	0.113833	7	92614358	0.010957	0.57	7	92614358	0.0348	1.35E-13
rs42398	T	C	T	C	0.2195	-0.00092	0.8279	0.521134	5	96784751	0.00671	0.89	5	96784751	0.0353	4.80E-10
rs4572866	T	C	T	C	0.2015	0.014132	0.6682	0.90562	4	26793981	0.011473	0.22	4	26793981	0.0289	3.06E-12
rs4651224	T	C	T	C	0.178	-0.0019	0.4766	0.612152	1	1.85E+08	0.006974	0.79	1	1.85E+08	0.0276	1.13E-10

rs4681794	C	T	C	T	0.2105	-0.0034	0.6394	0.4378	3	56742880	0.006805	0.62	3	56742880	0.0345	1.02E-09
rs4712120	G	T	G	T	-0.1971	0.001881	0.576	0.191402	6	56228118	0.008524	0.83	6	56228118	0.0333	3.19E-09
rs4768862	G	A	G	A	-0.264	-0.00263	0.6197	0.303074	12	50801044	0.007456	0.72	12	50801044	0.0343	1.34E-14
rs4854572	A	G	A	G	-0.1819	-0.0101	0.4388	0.268732	3	1.33E+08	0.007807	0.2	3	1.33E+08	0.027	1.53E-11
rs4858758	T	C	T	C	-0.2318	-0.0153	0.3923	0.440442	3	20075741	0.006945	0.028	3	20075741	0.0269	7.49E-18
rs4980470	A	G	A	G	0.1561	-0.00368	0.5874	0.752642	11	50665628	0.00801	0.65	11	50665628	0.0274	1.15E-08
rs544625	A	G	A	G	-0.1805	0.000769	0.6674	0.487272	11	30494949	0.006894	0.91	11	30494949	0.0281	1.26E-10
rs555625	C	T	C	T	0.3617	0.033393	0.9023	0.009846	5	1.23E+08	0.034929	0.34	5	1.23E+08	0.0589	8.46E-10
rs5579955	G	C	G	C	0.2034	0.004051	0.6546	0.358549	2	1.76E+08	0.0072	0.57	2	1.76E+08	0.0372	4.46E-08
rs5607341	A	C	A	C	-0.4144	-0.00263	0.1057	0.018012	2	24911142	0.02543	0.92	2	24911142	0.058	9.40E-13
rs569550	T	G	T	G	-0.3991	0.001211	0.6064	0.324207	11	1865838	0.007168	0.87	11	1865838	0.0347	1.14E-30
rs5740056	A	G	A	G	-0.2174	-0.00459	0.2134	0.560759	4	88831125	0.006943	0.51	4	88831125	0.0397	4.34E-08
rs5759103	A	G	A	G	0.2358	0.009542	0.3041	0.42219	2	2.08E+08	0.006837	0.16	2	2.08E+08	0.0366	1.18E-10
rs5786676	C	T	C	T	0.306	0.001953	0.5435	0.540586	10	94263320	0.006882	0.78	10	94263320	0.0327	7.99E-21
rs5787428	A	C	A	C	0.156	0.002776	0.3989	0.244236	2	1.11E+08	0.007854	0.72	2	1.11E+08	0.0279	2.26E-08
rs5965208	T	C	T	C	-0.4044	0.01006	0.1735	0.269693	11	47885798	0.007713	0.19	11	47885798	0.0475	1.57E-17
rs6026739	T	A	T	A	-0.3899	0.003569	0.8587	0.063401	20	59164414	0.013878	0.8	20	59164414	0.0492	2.35E-15
rs6031431	G	A	G	A	-0.2792	0.001936	0.5418	0.398655	20	44166512	0.007024	0.78	20	44166512	0.0333	5.65E-17
rs6054200	A	G	A	G	0.1644	-0.01624	0.5898	0.866715	20	6384998	0.010052	0.11	20	6384998	0.0268	8.35E-10
rs6069199	C	T	C	T	0.3528	0.002064	0.6921	0.048031	12	20215335	0.015513	0.89	12	20215335	0.0368	1.03E-21
rs6202076	T	C	T	C	-0.1738	-0.00498	0.4871	0.3744	15	92737261	0.007055	0.48	15	92737261	0.0272	1.60E-10
rs6237997	T	G	G	A	0.2407	0.003358	0.4659	0.380884	5	1.15E+08	0.007113	0.64	5	1.15E+08	0.0325	1.39E-13
rs6438253	A	G	A	G	0.1737	0.006107	0.529	0.910663	3	1.15E+08	0.011843	0.61	3	1.15E+08	0.0267	7.67E-11
rs6454092	G	A	G	A	0.2277	-0.00351	0.47	0.295149	6	78992255	0.007487	0.64	6	78992255	0.0333	7.53E-12
rs6503413	T	C	T	C	-0.2513	0.002716	0.5323	0.221422	17	45063974	0.00804	0.74	17	45063974	0.0325	1.14E-14
rs6595838	A	G	A	G	0.3426	0.010828	0.3522	0.229347	5	1.29E+08	0.008076	0.18	5	1.29E+08	0.0357	9.06E-22
rs6669371	G	T	G	T	0.6877	0.013528	0.8429	0.146494	1	11822085	0.009582	0.16	1	11822085	0.0453	4.88E-52
rs6672350	A	G	A	G	0.2131	-0.00114	0.692	0.598223	2	59836628	0.006969	0.87	2	59836628	0.0288	1.38E-13
rs6689862	T	C	T	C	-0.3273	0.014542	0.9147	0.904419	1	28510931	0.01164	0.21	1	28510931	0.0519	2.85E-10
rs6731208	CTGAA	C	C	T	-0.2282	-0.00096	0.2463	0.228626	2	1.91E+08	0.008251	0.91	2	1.91E+08	0.0385	3.19E-09
rs6731373	A	G	A	G	0.1815	0.001533	0.3309	0.147935	2	68275912	0.009508	0.87	2	68275912	0.0292	5.27E-10
rs6760542	A	C	A	C	-0.2231	-0.00853	0.3838	0.682037	3	26998960	0.007271	0.24	3	26998960	0.0379	4.01E-09
rs6793656	G	A	G	A	-0.2657	-0.0101	0.8567	0.084774	3	13781845	0.012282	0.41	3	13781845	0.0463	9.53E-09
rs6815273	A	G	A	G	-0.3283	-0.007	0.3927	0.441883	4	1.1E+08	0.006915	0.31	4	1.1E+08	0.038	5.70E-18
rs693367	A	G	A	G	-0.1777	0.008148	0.659	0.886888	4	55394495	0.010059	0.42	4	55394495	0.0279	1.89E-10
rs6954	T	C	T	C	-0.1512	0.008471	0.3853	0.462056	1	43450971	0.006895	0.22	1	43450971	0.0269	1.82E-08
rs6963853	A	G	A	G	0.1966	-0.00448	0.4442	0.314841	7	1819089	0.007193	0.53	7	1819089	0.0337	5.41E-09
rs7023828	T	C	T	C	-0.2278	0.012777	0.4287	0.352065	9	1.26E+08	0.007279	0.079	9	1.26E+08	0.0267	1.38E-17
rs702814	T	C	T	C	-0.1963	-0.0047	0.4861	0.037464	7	28133113	0.01708	0.78	7	28133113	0.0349	1.81E-08
rs7042283	T	G	T	G	-0.2202	0.053549	0.8509	0.979347	9	68903111	0.024385	0.028	9	68903111	0.0369	2.52E-09
rs7075018	T	C	T	C	0.1987	0.011179	0.3436	0.411864	10	1.32E+08	0.007066	0.11	10	1.32E+08	0.0284	2.59E-12
rs7125196	C	T	C	T	0.2642	-0.00965	0.8548	0.361431	11	61505093	0.007129	0.18	11	61505093	0.0472	2.18E-08

rs7128382	A	C	A	C	0.2688	0.003341	0.2123	0.552354	11	1.17E+08	0.006981	0.63	11	1.17E+08	0.0406	3.69E-11
rs7134677	T	C	T	C	-0.3406	-0.00687	0.2925	0.394092	12	54047714	0.007002	0.33	12	54047714	0.0367	1.70E-20
rs7173086	T	A	T	A	0.2486	0.003697	0.4654	0.67219	15	41217090	0.007228	0.61	15	41217090	0.0344	5.21E-13
rs7218605	T	C	T	C	-0.1822	-0.01206	0.5037	0.494476	17	78815721	0.006813	0.077	17	78815721	0.0323	1.67E-08
rs7226575	T	C	T	C	-0.2999	0.027635	0.8304	0.962776	18	33589018	0.018475	0.13	18	33589018	0.0435	5.64E-12
rs7245273	A	G	A	G	-0.2354	0.010372	0.3347	0.329251	18	44494557	0.007259	0.15	18	44494557	0.0345	9.14E-12
rs7275634	T	G	T	G	-0.2047	-0.00243	0.2739	0.371998	15	69305593	0.007141	0.73	15	69305593	0.0375	4.79E-08
rs7282178	T	C	T	C	-0.3366	-0.01921	0.1307	0.040106	10	61684670	0.017306	0.27	10	61684670	0.0495	1.02E-11
rs7283444	A	G	A	G	0.2613	0.00769	0.1195	0.026657	10	1.22E+08	0.020125	0.7	10	1.22E+08	0.0426	8.41E-10
rs7286518	T	C	T	C	-0.2245	-0.01346	0.2381	0.079491	1	15468885	0.012483	0.28	1	15468885	0.0396	1.40E-08
rs7291006	T	C	T	C	0.314	-0.00853	0.1181	0.111671	11	46323584	0.010996	0.44	11	46323584	0.0433	4.25E-13
rs7293174	A	G	A	G	0.2997	-0.02966	0.901	0.981748	11	69979308	0.025228	0.24	11	69979308	0.046	7.25E-11
rs7309990	T	C	T	C	0.3724	0.003782	0.0767	0.0317	12	53046995	0.018608	0.84	12	53046995	0.0647	8.77E-09
rs7313782	A	G	A	G	0.2709	-0.00404	0.2666	0.176753	12	90143493	0.00892	0.65	12	90143493	0.0408	3.29E-11
rs731749	A	G	A	G	0.32	-0.00166	0.0783	0.037224	16	86322976	0.018471	0.93	16	86322976	0.0563	1.30E-08
rs7406178	T	C	C	G	-0.2909	-0.0139	0.1101	0.006484	1	27387075	0.042953	0.75	1	27387075	0.044	3.81E-11
rs7412	T	C	T	C	-0.3255	-0.01288	0.0833	0.081172	19	44908822	0.012548	0.3	19	44908822	0.0486	2.08E-11
rs750416	T	C	T	C	-0.1502	0.002779	0.4619	0.449808	2	1.21E+08	0.006855	0.69	2	1.21E+08	0.0271	3.12E-08
rs7539215	T	G	T	G	-0.5716	0.024615	0.1138	0.009846	19	7256232	0.034083	0.47	19	7256232	0.0546	1.31E-25
rs7547570	A	G	A	G	0.168	-0.01188	0.3986	0.518012	1	66542812	0.006739	0.078	1	66542812	0.027	4.55E-10
rs7550273	C	G	C	G	-0.1935	0.010495	0.3928	0.096542	1	2.08E+08	0.011446	0.36	1	2.08E+08	0.0336	8.50E-09
rs758374	C	T	C	T	-0.2097	-0.00793	0.71	0.237512	22	19984029	0.008027	0.32	22	19984029	0.0363	7.70E-09
rs7601637	G	A	G	A	0.2175	-0.00173	0.6938	0.383045	2	37766161	0.006981	0.8	2	37766161	0.0351	5.59E-10
rs7620672	A	G	A	G	-0.4119	-0.0103	0.107	0.464217	7	40408372	0.006803	0.13	7	40408372	0.0566	3.43E-13
rs7671947	C	T	C	T	0.3182	0.003797	0.8736	0.196686	4	53934592	0.008571	0.66	4	53934592	0.0532	2.22E-09
rs7672622	A	G	A	G	0.2297	0.000198	0.7352	0.716859	4	1.57E+08	0.0075	0.98	4	1.57E+08	0.0314	2.54E-13
rs7719885	G	A	G	A	0.3611	-0.00501	0.6222	0.300192	5	1.58E+08	0.007421	0.5	5	1.58E+08	0.0344	7.99E-26
rs7796089	C	G	C	G	-0.221	0.006774	0.3394	0.285062	7	77882781	0.007537	0.37	7	77882781	0.0281	3.93E-15
rs7837090	C	G	C	G	0.2201	1.80E-05	0.8081	0.81268	8	73309171	0.008919	1	8	73309171	0.034	9.24E-11
rs7847526	C	T	C	T	-0.2558	-0.00524	0.8484	0.377762	9	1.1E+08	0.007145	0.46	9	1.1E+08	0.0467	4.26E-08
rs7886611	A	ATG	C	T	-0.1883	0.005891	0.5353	0.349664	4	1.08E+08	0.007022	0.4	4	1.08E+08	0.0334	1.66E-08
rs7889	C	G	C	G	-0.2499	0.001493	0.3904	0.228146	6	31637671	0.00821	0.86	6	31637671	0.033	3.73E-14
rs7969298	T	C	T	C	-0.3226	-0.05436	0.0954	0.009366	3	1.55E+08	0.034054	0.11	3	1.55E+08	0.0586	3.70E-08
rs7977128	A	T	A	T	-0.2214	-0.03182	0.8345	0.963257	17	60894370	0.018457	0.085	17	60894370	0.0364	1.15E-09
rs8002127	G	A	G	A	-0.5679	-0.04384	0.9583	0.006484	13	73250287	0.04286	0.31	13	73250287	0.0894	2.13E-10
rs8078112	C	T	C	T	-0.1913	0.003433	0.5367	0.205331	17	62643001	0.008248	0.68	17	62643001	0.0344	2.65E-08
rs8104223	G	A	G	A	-0.2141	-0.00727	0.7067	0.123679	19	15252666	0.010301	0.48	19	15252666	0.0367	5.33E-09
rs8104559	T	C	T	C	-0.2282	0.008011	0.8418	0.858069	19	22022365	0.009769	0.41	19	22022365	0.0367	4.89E-10
rs821317	A	G	A	G	0.2004	-0.01015	0.1995	0.117435	9	1.38E+08	0.010703	0.34	9	1.38E+08	0.0353	1.42E-08
rs896693	A	G	A	G	-0.166	0.00389	0.422	0.131364	11	1.2E+08	0.010043	0.7	11	1.2E+08	0.0278	2.25E-09
rs9330353	A	T	A	T	0.2169	0.003796	0.4499	0.511287	4	1.38E+08	0.006869	0.58	4	1.38E+08	0.0273	2.03E-15
rs9349379	G	A	G	A	0.2254	0.010662	0.6016	0.65634	6	12903725	0.007174	0.14	6	12903725	0.0354	1.83E-10

rs9356632 A	G	A	G	-0.2743	-0.00756	0.1762	0.807157	6	1.7E+08	0.008213	0.36	6	1.7E+08	0.0367	7.27E-14
rs9401025 C	A	C	A	0.203	-0.01238	0.5712	0.21854	6	1.18E+08	0.007991	0.12	6	1.18E+08	0.0354	9.27E-09
rs9506725 T	C	T	C	0.2172	0.045487	0.6487	0.987032	13	21740007	0.029433	0.12	13	21740007	0.0272	1.37E-15
rs9508495 T	C	T	C	-0.2242	-0.00503	0.6709	0.362632	13	29572064	0.006939	0.47	13	29572064	0.037	1.36E-09
rs9532959 A	G	A	G	0.3137	-0.00939	0.0835	0.121037	13	41969380	0.010337	0.36	13	41969380	0.0499	3.37E-10
rs9549328 T	C	T	C	0.2665	0.010204	0.2246	0.235591	13	1.13E+08	0.008058	0.21	13	1.13E+08	0.0394	1.32E-11
rs9818220 T	C	T	C	-0.2034	0.005026	0.7293	0.51537	3	30242829	0.006786	0.46	3	30242829	0.031	5.65E-11
rs983016 G	C	C	T	0.1996	-0.00303	0.4953	0.405139	4	38386666	0.007128	0.67	4	38386666	0.0329	1.29E-09
rs9849301 T	C	T	C	0.2284	0.007925	0.1412	0.1878	3	1.59E+08	0.008868	0.37	3	1.59E+08	0.0383	2.47E-09
rs9912738 C	T	C	T	-0.2037	0.002966	0.6214	0.387848	17	49433222	0.006933	0.67	17	49433222	0.0354	8.62E-09

Supplementary Table 2g. List of SNPs for Diastolic BP (DBP) and their effects on TEWL

SNP	effect_alle	other_allel	effect_alle	other_allel	beta.DBP	beta.TEWL	eaf.DBP	eaf.TEWL	chr.TEWL	pos.TEWL	se.TEWL	pval.TEWL	chr.DBP	pos.DBP	se.DBP	pval.DBP
chr1:1410	T	G	T	G	0.4383	-0.00632	0.7778	0.104227	1	1410616	0.01126	0.57	1	1410616	0.0781	1.98E-08
chr1:2045	C	G	C	G	0.1577	0.015079	0.7334	0.946206	1	2.05E+08	0.014803	0.31	1	2.05E+08	0.0271	6.08E-09
chr12:111	T	C	T	C	0.1536	-0.00864	0.4816	0.012968	12	1.11E+08	0.028479	0.76	12	1.11E+08	0.0255	1.70E-09
chr15:713	C	A	C	A	0.1868	-0.00014	0.5618	0.334054	15	71314327	0.007159	0.98	15	71314327	0.0252	1.22E-13
chr16:562	T	G	T	G	-0.1519	-0.0079	0.4065	0.231508	16	56294901	0.008225	0.34	16	56294901	0.0271	2.04E-08
chr3:1015	C	G	C	G	0.1373	-0.00464	0.407	0.411623	3	1.02E+08	0.006901	0.5	3	1.02E+08	0.0192	9.23E-13
chr3:1341	T	A	T	A	0.1719	0.052649	0.6932	0.010327	3	1.34E+08	0.034158	0.12	3	1.34E+08	0.0299	9.13E-09
chr5:1235	A	G	A	G	0.1714	0.009167	0.4303	0.267051	5	1.24E+08	0.007887	0.25	5	1.24E+08	0.0253	1.26E-11
chr5:1323	C	T	C	T	0.1483	-0.04512	0.5313	0.011287	5	1.32E+08	0.031335	0.15	5	1.32E+08	0.0255	6.31E-09
chr5:7570	T	C	T	C	-0.1404	0.003833	0.5369	0.433718	5	75707853	0.006927	0.58	5	75707853	0.0257	4.46E-08
chr6:2586	T	C	T	A	-0.3103	0.031111	0.3528	0.019452	6	25866004	0.023883	0.19	6	25866004	0.0474	5.92E-11
chr7:1067	G	T	G	T	0.2573	-0.00234	0.6507	0.151537	7	1.07E+08	0.00946	0.8	7	1.07E+08	0.0306	4.42E-17
chr7:1900	G	T	G	T	0.2072	0.002374	0.6395	0.350624	7	19006554	0.007276	0.74	7	19006554	0.0311	2.82E-11
chr7:4592	C	G	C	G	0.228	0.005981	0.4472	0.714938	7	45921046	0.007427	0.42	7	45921046	0.0251	1.18E-19
rs1017699	C	T	C	T	-0.2275	-0.0133	0.5928	0.068924	2	19508931	0.013239	0.32	2	19508931	0.0262	3.40E-18
rs1092025	A	G	A	G	-0.1723	0.022366	0.4061	0.080452	1	2.02E+08	0.012709	0.078	1	2.02E+08	0.0277	5.30E-10
rs1092699	G	A	G	A	-0.2022	-0.00057	0.6023	0.242315	1	2.43E+08	0.007913	0.94	1	2.43E+08	0.0259	5.29E-15
rs1122208	T	A	T	A	0.2736	-0.00679	0.5851	0.030259	11	1.3E+08	0.019083	0.72	11	1.3E+08	0.0261	1.11E-25
rs1189988	G	A	G	A	-0.2549	-0.04927	0.6448	0.004083	2	55875609	0.053972	0.36	2	55875609	0.037	5.92E-12
rs1220317	G	C	G	C	0.219	-0.01187	0.6788	0.103026	6	56129439	0.011094	0.28	6	56129439	0.0326	1.89E-11
rs1220877	C	T	C	T	-0.2679	-0.00021	0.6101	0.430596	6	1.22E+08	0.006687	0.98	6	1.22E+08	0.0363	1.59E-13
rs1250259	T	A	T	A	0.4275	-0.0071	0.3196	0.118636	2	2.15E+08	0.010064	0.48	2	2.15E+08	0.0515	1.09E-16
rs1279755	A	G	A	G	0.177	-0.03477	0.3801	0.007205	11	1.17E+08	0.038211	0.36	11	1.17E+08	0.0292	1.36E-09
rs1294351	A	G	A	G	0.1865	0.006388	0.3937	0.14001	17	4180628	0.009687	0.51	17	4180628	0.028	2.53E-11
rs1303524	T	C	T	C	0.1606	-0.00477	0.5232	0.602546	2	24911140	0.007057	0.5	2	24911140	0.0283	1.33E-08
rs1322639	G	A	G	A	-0.2352	-0.01139	0.6172	0.420749	6	1.69E+08	0.006838	0.096	6	1.69E+08	0.0283	8.78E-17
rs1773776	C	T	C	T	-0.2621	0.006504	0.668	0.007685	2	46129994	0.038239	0.86	2	46129994	0.0402	6.78E-11
rs2009598	A	G	A	G	-0.1674	-0.00081	0.443	0.35951	17	6567278	0.007101	0.91	17	6567278	0.027	5.90E-10
rs2282978	T	C	T	C	-0.1578	0.011835	0.6482	0.869597	7	92635096	0.010177	0.24	7	92635096	0.0247	1.58E-10
rs2289124	A	G	A	G	0.2199	0.009791	0.3822	0.228626	11	89491309	0.008196	0.23	11	89491309	0.0328	1.98E-11
rs2505084	A	G	A	G	-0.1735	0.011235	0.4638	0.231268	10	30046591	0.008179	0.17	10	30046591	0.0248	2.88E-12
rs2543029	C	A	C	A	0.2381	-0.00044	0.633	0.303554	18	45518310	0.007546	0.95	18	45518310	0.03	1.95E-15
rs2683696	C	T	C	T	-0.3962	-0.00489	0.5807	0.174832	3	41873406	0.009117	0.59	3	41873406	0.0305	1.36E-38
rs268771	C	T	C	T	0.1344	0.006763	0.5043	0.213737	3	57952265	0.008164	0.41	3	57952265	0.0242	2.77E-08
rs2894251	C	T	C	T	0.3443	0.022956	0.6531	0.023055	6	32375459	0.022402	0.31	6	32375459	0.0406	2.16E-17
rs3130566	C	T	C	G	0.3056	0.008479	0.6478	0.153218	6	31134841	0.009523	0.37	6	31134841	0.0402	2.97E-14
rs3130631	A	G	C	G	-0.317	0.026295	0.3561	0.079251	6	31598391	0.012704	0.038	6	31598391	0.0419	3.98E-14
rs3407044	A	G	A	G	-0.1126	0.00723	0.5694	0.518972	5	1.48E+08	0.00681	0.29	5	1.48E+08	0.0178	2.45E-10
rs3437018	T	G	T	G	0.2019	0.003336	0.3823	0.177474	1	59191237	0.008842	0.71	1	59191237	0.0284	1.20E-12
rs365990	G	A	G	A	-0.1937	0.000975	0.5291	0.179635	14	23392602	0.008878	0.91	14	23392602	0.025	8.88E-15

rs3786130	C	T	C	T	0.1571	-0.0185	0.5616	0.323247	17	62687682	0.007225	0.01	17	62687682	0.0254	6.59E-10
rs3790228	G	T	G	T	0.1759	0.000564	0.6088	0.501681	20	19488506	0.00687	0.93	20	19488506	0.0271	9.24E-11
rs4894055	A	G	A	G	0.324	-0.03337	0.0864	0.974784	2	1.79E+08	0.021555	0.12	2	1.79E+08	0.0441	2.11E-13
rs4925166	T	C	A	C	-0.1569	0.002024	0.4931	0.467339	17	18307496	0.006863	0.77	17	18307496	0.0201	6.57E-15
rs6541001	C	CT	A	G	0.3061	0.012187	0.8965	0.14073	1	11777310	0.009787	0.21	1	11777310	0.0451	1.15E-11
rs6578061	A	G	A	G	0.1465	0.006611	0.5068	0.503602	8	1.4E+08	0.006925	0.34	8	1.4E+08	0.0261	2.00E-08
rs7500448	G	A	G	A	-0.2134	0.013632	0.6143	0.254563	16	83012185	0.007926	0.085	16	83012185	0.0303	1.95E-12
rs7766549	C	T	C	T	0.2259	-0.0068	0.6284	0.384246	6	1.52E+08	0.006853	0.32	6	1.52E+08	0.0349	9.45E-11
rs7987651	T	C	T	C	-0.1329	-0.00718	0.1763	0.03194	13	1.1E+08	0.019093	0.71	13	1.1E+08	0.0237	2.05E-08
rs8023114	T	TAAATC	T	C	0.2163	-0.00428	0.4099	0.369116	14	91925611	0.006965	0.54	14	91925611	0.0274	2.64E-15
rs8030926	A	G	A	G	-0.3319	0.020459	0.3552	0.947647	8	1.19E+08	0.015085	0.18	8	1.19E+08	0.0295	2.32E-29
rs9467704	T	C	T	C	-0.3191	0.007629	0.345	0.04635	6	26319258	0.016266	0.64	6	26319258	0.0446	8.34E-13
rs956006	T	C	T	C	0.1595	0.009245	0.4183	0.266811	15	62516340	0.007777	0.23	15	62516340	0.0285	2.28E-08
rs9644861	C	T	C	T	-0.1397	-0.00152	0.515	0.331412	9	22090936	0.007346	0.84	9	22090936	0.0256	4.77E-08
rs9821489	A	G	A	G	0.4962	0.025092	0.4813	0.052354	3	53541701	0.01516	0.098	3	53541701	0.067	1.32E-13

Supplementary Table 2h. List of SNPs for Pulse Pressure (PP) and their effects on TEWL

SNP	effect_alle	other_allel	effect_alle	other_allel	beta.PP	beta.TEWL	eaf.PP	eaf.TEWL	chr.TEWL	pos.TEWL	se.TEWL	pval.TEWL	chr.PP	pos.PP	se.PP	pval.PP
chr1:1073	G	C	G	C	-0.1644	0.001291	0.6558	0.694765	1	10738432	0.007175	0.86	1	10738432	0.0259	2.35E-10
chr1:2017	C	A	C	A	-0.1975	0.002837	0.622	0.29611	1	2.02E+08	0.007545	0.71	1	2.02E+08	0.0259	2.65E-14
chr1:5048	A	G	A	G	-0.2792	-0.0098	0.0866	0.086215	1	50488456	0.012202	0.42	1	50488456	0.0449	5.18E-10
chr1:5099	G	A	G	A	0.2785	0.001312	0.8998	0.089577	1	50991528	0.012038	0.91	1	50991528	0.0423	4.43E-11
chr1:5611	A	G	A	G	0.1956	-0.00138	0.328	0.179155	1	56111252	0.008985	0.88	1	56111252	0.0274	9.28E-13
chr10:112	T	C	T	C	0.2209	0.008164	0.2904	0.052354	10	1.13E+08	0.014522	0.57	10	1.13E+08	0.0266	8.86E-17
chr10:184	G	C	G	C	0.2252	0.002543	0.7069	0.029539	10	18439030	0.019178	0.89	10	18439030	0.0279	7.57E-16
chr10:564	C	G	C	G	-0.1789	-0.00783	0.1204	0.123919	10	5647187	0.010396	0.45	10	5647187	0.032	2.31E-08
chr11:117	G	A	G	A	0.2518	0.003299	0.4751	0.342939	11	1.17E+08	0.007163	0.65	11	1.17E+08	0.0241	1.74E-25
chr11:187	A	G	A	G	0.2148	-0.0046	0.3581	0.613593	11	1871700	0.006868	0.5	11	1871700	0.026	1.41E-16
chr12:123	T	A	T	A	0.2799	-0.0087	0.9265	0.282181	12	1.23E+08	0.007695	0.26	12	1.23E+08	0.0512	4.47E-08
chr13:316	T	C	T	C	0.1512	-0.0032	0.4717	0.098703	13	31605999	0.011505	0.78	13	31605999	0.0194	6.42E-15
chr14:354	A	G	A	G	0.1714	-0.00327	0.4011	0.463737	14	35402011	0.006907	0.64	14	35402011	0.0248	5.19E-12
chr14:746	T	C	T	C	-0.1245	-0.00815	0.5111	0.797791	14	74613001	0.008415	0.33	14	74613001	0.0191	6.37E-11
chr16:129	A	C	A	C	-0.1556	0.00648	0.5815	0.882565	16	1297716	0.010426	0.53	16	1297716	0.0201	1.13E-14
chr16:247	G	A	G	A	0.1758	-0.0202	0.7338	0.128963	16	24741320	0.010232	0.048	16	24741320	0.0279	2.80E-10
chr16:730	T	C	T	C	0.1276	0.005081	0.7376	0.902257	16	73064057	0.011431	0.66	16	73064057	0.0223	1.14E-08
chr17:643	T	C	T	C	-0.1442	0.001754	0.3926	0.308357	17	64304354	0.007422	0.81	17	64304354	0.0261	3.27E-08
chr18:455	A	C	A	C	0.3134	0.000423	0.219	0.301153	18	45517785	0.007546	0.96	18	45517785	0.0302	3.25E-25
chr18:555	A	G	A	G	-0.1747	0.009273	0.859	0.982949	18	55585157	0.025338	0.71	18	55585157	0.0284	7.30E-10
chr19:151	C	T	C	T	0.2094	0.003272	0.7008	0.405379	19	15156052	0.006895	0.64	19	15156052	0.0279	6.07E-14
chr19:498	T	C	T	C	0.1284	-0.01766	0.3558	0.276897	19	4980853	0.007609	0.02	19	4980853	0.0199	1.16E-10
chr19:664	T	C	T	C	-0.1668	0.001962	0.8137	0.960135	19	664278	0.017253	0.91	19	664278	0.027	6.61E-10
chr2:1055	A	G	A	G	-0.1758	-0.0194	0.1072	0.100865	2	1.06E+08	0.011111	0.081	2	1.06E+08	0.0313	1.87E-08
chr2:1821	C	G	C	G	-0.1918	-0.00947	0.1561	0.259606	2	1.82E+08	0.007779	0.22	2	1.82E+08	0.0349	4.03E-08
chr2:1952	A	C	A	C	-0.2836	-0.01375	0.3249	0.068444	2	19523852	0.013274	0.3	2	19523852	0.0259	7.93E-28
chr2:2031	G	C	G	C	0.1859	-0.00187	0.4763	0.346542	2	2.03E+08	0.007171	0.79	2	2.03E+08	0.0256	3.66E-13
chr2:2067	C	T	C	T	-0.2354	-0.00566	0.479	0.251201	2	20679060	0.007943	0.48	2	20679060	0.0243	3.51E-22
chr2:2262	A	C	A	C	-0.1542	-0.00025	0.3332	0.097743	2	2.26E+08	0.011375	0.98	2	2.26E+08	0.0259	2.65E-09
chr2:2669	T	G	T	G	-0.2461	-0.00802	0.5707	0.280259	2	26692756	0.007348	0.27	2	26692756	0.026	2.55E-21
chr20:194	A	C	A	C	0.2685	-0.00067	0.3181	0.513449	20	19488358	0.006844	0.92	20	19488358	0.0267	8.77E-24
chr20:667	C	G	C	G	-0.1563	0.01121	0.5245	0.771374	20	6675381	0.008212	0.17	20	6675381	0.0192	3.32E-16
chr3:1001	A	G	A	G	0.1876	0.031331	0.8232	0.990634	3	1E+08	0.034911	0.37	3	1E+08	0.026	5.66E-13
chr3:1153	A	G	A	G	0.1228	0.006508	0.5201	0.911383	3	1.15E+08	0.01191	0.58	3	1.15E+08	0.0192	1.75E-10
chr3:1604	T	C	T	C	-0.1376	-0.01162	0.4853	0.235351	3	1.6E+08	0.008079	0.15	3	1.6E+08	0.0247	2.43E-08
chr3:1697	C	T	C	T	0.1976	-0.00432	0.752	0.509846	3	1.7E+08	0.006846	0.53	3	1.7E+08	0.0291	1.07E-11
chr4:1458	G	A	G	A	-0.144	0.007321	0.5618	0.167867	4	1.46E+08	0.009095	0.42	4	1.46E+08	0.0261	3.28E-08
chr4:2721	T	C	T	C	-0.1395	-0.00054	0.3922	0.37536	4	2721066	0.007006	0.94	4	2721066	0.0196	1.07E-12
chr4:3941	A	T	A	T	-0.1256	-0.02317	0.6913	0.889289	4	39412030	0.010714	0.031	4	39412030	0.0211	2.58E-09
chr4:8581	G	C	G	C	-0.2485	0.007761	0.8606	0.153458	4	85810232	0.009555	0.42	4	85810232	0.036	5.41E-12

chr5:3283:	T	C	T	C	-0.2268	-0.0267	0.4085	0.338377	5	32831833	0.007337	0.00027	5	32831833	0.0248	5.54E-20
chr5:7335:	T	C	T	C	0.1618	-0.01399	0.1999	0.032181	5	73351691	0.019465	0.47	5	73351691	0.0274	3.54E-09
chr5:7570:	T	C	T	C	0.1593	0.003833	0.5943	0.433718	5	75707853	0.006927	0.58	5	75707853	0.0264	1.51E-09
chr6:1267:	C	T	C	T	-0.1482	-0.00099	0.5815	0.435639	6	1.27E+08	0.006761	0.88	6	1.27E+08	0.0262	1.58E-08
chr6:1592:	A	G	A	G	-0.1908	-0.00332	0.5843	0.374159	6	1.59E+08	0.006978	0.63	6	1.59E+08	0.0252	4.06E-14
chr6:2068(	A	C	A	C	0.1302	-0.00227	0.2643	0.338377	6	20686765	0.007258	0.75	6	20686765	0.0214	1.21E-09
chr6:4338:	A	G	A	G	0.196	0.013186	0.3962	0.341499	6	43381477	0.007312	0.071	6	43381477	0.026	4.42E-14
chr6:7213(	A	C	A	C	0.1613	-0.00463	0.8219	0.95293	6	7213674	0.016246	0.78	6	7213674	0.0257	3.62E-10
chr7:4597(	G	C	G	C	0.4633	0.003803	0.5689	0.73487	7	45970501	0.007642	0.62	7	45970501	0.0247	2.44E-78
chr8:1039(	T	C	T	C	0.1869	-0.00288	0.3578	0.220461	8	10390466	0.008358	0.73	8	10390466	0.0202	2.31E-20
chr8:3838!	A	T	A	T	0.152	-0.01091	0.2345	0.302354	8	38385709	0.007443	0.14	8	38385709	0.0226	1.83E-11
chr8:7567!	A	C	A	C	0.2502	0.016664	0.5121	0.409462	8	75679645	0.006946	0.016	8	75679645	0.0247	4.45E-24
chr9:1231(	G	C	G	C	0.2261	0.005242	0.8128	0.568924	9	1.23E+08	0.006734	0.44	9	1.23E+08	0.0354	1.64E-10
chr9:9241!	A	G	A	G	0.1275	-0.00905	0.6077	0.855427	9	92419346	0.009598	0.35	9	92419346	0.02	1.87E-10
rs1008628	A	G	A	G	0.1197	0.003292	0.3113	0.211335	8	94974699	0.008178	0.69	8	94974699	0.0209	9.61E-09
rs1008872	A	G	A	G	-0.189	0.003722	0.5801	0.479827	8	1.4E+08	0.006872	0.59	8	1.4E+08	0.0254	1.07E-13
rs1019525	T	C	T	C	0.1156	0.000905	0.5704	0.884726	2	1.65E+08	0.010655	0.93	2	1.65E+08	0.0197	4.51E-09
rs1027095	A	T	A	T	0.1431	0.012504	0.5227	0.204371	7	90177982	0.008438	0.14	7	90177982	0.0189	3.93E-14
rs1036821	A	G	A	G	0.1536	0.007503	0.2957	0.522815	8	1.35E+08	0.006947	0.28	8	1.35E+08	0.0272	1.66E-08
rs1048798	A	T	A	T	0.1543	0.043243	0.1707	0.017531	7	90857507	0.025035	0.084	7	90857507	0.0259	2.73E-09
rs1050923	G	GC	G	A	0.2473	-0.0187	0.1376	0.0305	1	1.54E+08	0.019461	0.34	1	1.54E+08	0.045	3.99E-08
rs1061808	T	G	T	G	-0.25	0.004217	0.3698	0.416667	6	32168770	0.007024	0.55	6	32168770	0.0249	1.09E-23
rs1073243	T	C	T	C	0.1784	0.010207	0.445	0.557877	10	20748365	0.007009	0.15	10	20748365	0.025	8.58E-13
rs1077061	G	A	G	A	0.2502	-0.02148	0.7997	0.349904	12	20077705	0.007368	0.0036	12	20077705	0.0314	1.62E-15
rs1083095	A	G	A	G	-0.1188	0.003649	0.6985	0.539385	11	92951950	0.007007	0.6	11	92951950	0.0209	1.21E-08
rs1085714	T	A	T	A	-0.2136	-0.0028	0.7235	0.398655	4	80259918	0.007081	0.69	4	80259918	0.0279	1.77E-14
rs1095679	A	T	A	T	0.1574	-0.00366	0.7017	0.616475	8	91242113	0.006924	0.6	8	91242113	0.0216	2.84E-13
rs1100075	T	G	T	G	-0.2218	0.011696	0.8663	0.813641	10	73710597	0.00865	0.18	10	73710597	0.037	2.12E-09
rs1101090	A	T	A	T	0.1336	-0.00117	0.507	0.566282	10	19645884	0.006964	0.87	10	19645884	0.0195	6.87E-12
rs1108239	T	C	T	C	-0.2353	0.010043	0.3331	0.329731	18	44491260	0.007262	0.17	18	44491260	0.0259	1.15E-19
rs1118784	A	C	A	C	-0.2729	-0.00724	0.1188	0.239433	10	94296872	0.008318	0.38	10	94296872	0.0383	1.10E-12
rs1119135	T	A	T	A	0.3377	-0.00361	0.896	0.37536	10	1.03E+08	0.007091	0.61	10	1.03E+08	0.043	4.25E-15
rs1122208	T	A	T	A	-0.4622	-0.00679	0.6581	0.030259	11	1.3E+08	0.019083	0.72	11	1.3E+08	0.0267	5.44E-67
rs1125683	A	G	A	G	0.1808	-0.02076	0.197	0.013449	10	10798572	0.029476	0.48	10	10798572	0.0253	9.68E-13
rs1126684	A	G	A	G	-0.3785	-0.00192	0.0779	0.017771	5	1.09E+08	0.02516	0.94	5	1.09E+08	0.0463	2.80E-16
rs1137442	T	G	T	G	-0.1662	0.006372	0.4796	0.099904	12	65979721	0.011408	0.58	12	65979721	0.025	2.78E-11
rs1146975	T	A	T	C	0.4622	-0.04272	0.9267	0.01537	12	94283783	0.028116	0.13	12	94283783	0.0494	8.50E-21
rs1155422	T	G	T	G	-0.1685	0.008404	0.5118	0.127041	4	1.55E+08	0.010245	0.41	4	1.55E+08	0.0261	1.11E-10
rs1177702	T	C	T	C	-0.3563	-0.00083	0.0435	0.06268	6	34161184	0.013879	0.95	6	34161184	0.0617	7.83E-09
rs1185335	A	G	A	G	0.1798	0.001987	0.3548	0.303554	15	71329185	0.007351	0.79	15	71329185	0.0258	2.94E-12
rs1189988	G	A	G	A	0.2701	-0.04927	0.8602	0.004083	2	55875609	0.053972	0.36	2	55875609	0.038	1.15E-12
rs1203421	T	G	T	G	-0.1666	0.00145	0.4104	0.284342	1	41909802	0.007594	0.85	1	41909802	0.0268	5.07E-10

rs1205445	A	G	A	G	0.1145	-0.00038	0.3373	0.248799	20	38249344	0.007976	0.96	20	38249344	0.021	4.93E-08
rs1210062	T	C	T	C	0.2067	0.009397	0.2024	0.772094	14	98169146	0.00808	0.24	14	98169146	0.0315	5.38E-11
rs1220317	G	C	G	C	-0.3528	-0.01187	0.8396	0.103026	6	56129439	0.011094	0.28	6	56129439	0.0333	3.70E-26
rs1222433	A	G	A	G	0.1406	0.006082	0.4863	0.508405	11	1.07E+08	0.006874	0.38	11	1.07E+08	0.0244	8.42E-09
rs1226418	T	C	T	C	0.1845	0.007809	0.1806	0.021374	10	32001058	0.023734	0.74	10	32001058	0.0319	7.03E-09
rs1240608	G	C	G	C	0.2275	0.020695	0.6564	0.206532	1	11861124	0.008325	0.013	1	11861124	0.0267	1.66E-17
rs1241231	A	G	A	G	0.1541	-0.01873	0.7737	0.853506	10	1.33E+08	0.00971	0.054	10	1.33E+08	0.0236	6.35E-11
rs1241633	A	T	A	T	-0.5987	-0.00533	0.0909	0.260807	10	1.03E+08	0.007897	0.5	10	1.03E+08	0.0441	5.94E-42
rs1242725	T	C	T	C	-0.1991	-0.00769	0.187	0.051873	12	50488738	0.014951	0.61	12	50488738	0.0349	1.18E-08
rs1244917	T	C	T	C	0.2264	-0.00082	0.5583	0.508646	16	75429114	0.006819	0.9	16	75429114	0.025	1.37E-19
rs1245312	G	A	G	A	0.1554	-0.00333	0.4698	0.458694	17	59968715	0.006762	0.62	17	59968715	0.0265	4.53E-09
rs1246327	A	T	A	T	0.242	-0.01011	0.2051	0.361431	19	7243073	0.007051	0.15	19	7243073	0.0329	1.79E-13
rs1248500	A	G	A	G	0.4076	0.015622	0.0889	0.094621	22	40239272	0.011741	0.18	22	40239272	0.0494	1.48E-16
rs1250259	T	A	T	A	-0.4236	-0.0071	0.7437	0.118636	2	2.15E+08	0.010064	0.48	2	2.15E+08	0.0517	2.54E-16
rs1251116	T	C	T	C	0.1351	0.005517	0.6456	0.541787	4	94652847	0.006834	0.42	4	94652847	0.02	1.39E-11
rs1256508	GT	G	G	T	0.2916	-0.00834	0.8959	0.07757	1	2.08E+08	0.012759	0.51	1	2.08E+08	0.0414	1.84E-12
rs1270539	A	G	A	G	0.7464	-0.00102	0.1908	0.148895	7	1.07E+08	0.009498	0.91	7	1.07E+08	0.0315	7.09E-124
rs1280722	A	G	A	G	-0.1585	-0.00101	0.3475	0.31316	11	1.02E+08	0.007345	0.89	11	1.02E+08	0.0203	6.28E-15
rs1296657	A	G	A	G	-0.1798	-0.03414	0.1232	0.012488	18	12736043	0.031075	0.27	18	12736043	0.0311	7.53E-09
rs1298229	T	G	T	G	0.1862	0.000285	0.3398	0.058598	19	40541454	0.014169	0.98	19	40541454	0.0209	5.83E-19
rs1302624	T	C	T	C	-0.2918	-0.00743	0.1744	0.379443	2	1.64E+08	0.007055	0.29	2	1.64E+08	0.0329	8.06E-19
rs1308340	A	G	A	G	0.1142	-0.0088	0.4304	0.465418	3	73197186	0.006744	0.19	3	73197186	0.0198	7.49E-09
rs1316353	C	G	C	G	-0.1563	-0.00504	0.1623	0.220701	5	71772334	0.008161	0.54	5	71772334	0.0283	3.24E-08
rs1322739	T	C	T	C	-0.2193	0.009564	0.901	0.971182	7	46510929	0.019967	0.63	7	46510929	0.0325	1.52E-11
rs1324167	C	T	C	T	-0.1716	0.001212	0.7785	0.048511	7	73903956	0.016322	0.94	7	73903956	0.0311	3.50E-08
rs13260	T	G	T	G	0.2548	-0.0238	0.1314	0.012968	13	1.1E+08	0.029949	0.43	13	1.1E+08	0.0401	2.10E-10
rs1326307	A	C	A	C	0.1863	-0.00514	0.8133	0.788184	8	1.05E+08	0.008286	0.53	8	1.05E+08	0.0266	2.31E-12
rs1328800	A	G	A	G	0.1587	-0.01233	0.6415	0.794669	9	1.26E+08	0.008427	0.14	9	1.26E+08	0.0207	1.69E-14
rs1329032	T	C	T	C	-0.1575	-0.00697	0.4919	0.380403	9	1.14E+08	0.006979	0.32	9	1.14E+08	0.0194	4.86E-16
rs1330812	T	C	T	C	-0.1827	0.012361	0.3345	0.404659	7	1.32E+08	0.006891	0.073	7	1.32E+08	0.0304	1.86E-09
rs1333045	C	T	C	T	-0.1728	0.001056	0.4894	0.510327	9	22119196	0.006952	0.88	9	22119196	0.0247	2.72E-12
rs134077	A	G	A	G	-0.1596	-0.00314	0.42	0.482469	22	27667264	0.006796	0.64	22	27667264	0.0254	3.47E-10
rs1436206	A	G	A	G	-0.1068	0.002334	0.4846	0.695965	10	50360852	0.007419	0.75	10	50360852	0.0191	2.12E-08
rs1489110	T	G	T	G	0.1715	0.000713	0.3777	0.825408	12	48318084	0.008438	0.93	12	48318084	0.0197	3.06E-18
rs1557765	T	C	T	C	0.2227	-0.0045	0.3713	0.362392	11	17382092	0.007071	0.52	11	17382092	0.0262	2.09E-17
rs160337	AT	A	T	G	-0.1597	0.013186	0.6681	0.057157	7	28588171	0.014705	0.37	7	28588171	0.0266	2.02E-09
rs1630736	T	C	T	C	-0.1271	0.000746	0.4609	0.547791	6	12295754	0.00676	0.91	6	12295754	0.0203	4.21E-10
rs1646010	C	G	C	G	0.1343	0.004124	0.2537	0.398655	16	58515300	0.007108	0.56	16	58515300	0.0224	2.06E-09
rs1675382	A	G	A	G	0.1325	-3.33E-06	0.5186	0.197646	3	57959516	0.008466	1	3	57959516	0.0243	4.81E-08
rs1695412	A	G	A	G	0.2116	0.021893	0.887	0.911383	16	80834007	0.012149	0.072	16	80834007	0.0333	2.11E-10
rs174564	G	A	G	A	0.1696	-0.0021	0.6728	0.588136	11	61820833	0.006612	0.75	11	61820833	0.0274	6.38E-10
rs1760876	G	A	G	A	-0.4936	-0.02922	0.8796	0.009126	17	46935905	0.034426	0.4	17	46935905	0.0399	3.58E-35

rs1773776	C	A	G	A	0.3482	0.006504	0.8922	0.007685	2	46129994	0.038239	0.86	2	46129994	0.041	1.86E-17
rs177992	A	G	A	G	-0.1226	0.011845	0.3544	0.133525	18	22483241	0.009864	0.23	18	22483241	0.0205	2.43E-09
rs1800629	A	G	A	G	0.2902	0.02391	0.166	0.083093	6	31575254	0.012441	0.055	6	31575254	0.0327	7.66E-19
rs1818275	T	C	T	C	0.5369	-0.04597	0.9048	0.986792	15	48591742	0.029724	0.12	15	48591742	0.0423	7.60E-37
rs1919865	T	A	T	A	0.3173	-0.00198	0.7563	0.431796	6	1.22E+08	0.00668	0.77	6	1.22E+08	0.0345	3.71E-20
rs193686	C	T	C	T	-0.1465	-0.00193	0.6767	0.100624	7	1.17E+08	0.011286	0.86	7	1.17E+08	0.0265	3.09E-08
rs200481	A	T	A	T	0.3585	0.002305	0.0932	0.070125	6	27806054	0.013494	0.86	6	27806054	0.0441	4.20E-16
rs2005950	A	G	A	G	-0.1763	-0.01798	0.1437	0.04611	21	45976680	0.016312	0.27	21	45976680	0.028	2.94E-10
rs2011603	A	G	A	G	0.1432	0.006136	0.7256	0.589817	4	18023861	0.006827	0.37	4	18023861	0.0213	1.91E-11
rs2023843	C	T	C	T	0.3618	-0.00606	0.9072	0.341499	7	27203602	0.007021	0.39	7	27203602	0.0436	9.92E-17
rs2069833	T	C	T	C	-0.1816	-0.0216	0.5874	0.981508	7	22728045	0.02332	0.35	7	22728045	0.0202	2.16E-19
rs2071382	T	C	T	C	0.2317	0.002914	0.4374	0.180836	15	90884967	0.008652	0.74	15	90884967	0.0257	2.31E-19
rs2075665	A	T	A	T	0.1672	0.006991	0.6078	0.575889	9	98987661	0.006986	0.32	9	98987661	0.0195	8.82E-18
rs210314	T	C	T	C	-0.1278	-0.00115	0.5558	0.65658	14	53655683	0.007336	0.88	14	53655683	0.0193	3.75E-11
rs2104038	G	A	G	A	0.1727	-0.01079	0.6819	0.067723	1	15649492	0.013351	0.42	1	15649492	0.027	1.69E-10
rs2107595	A	G	A	G	0.5121	0.00132	0.1834	0.332373	7	19009765	0.007365	0.86	7	19009765	0.0331	5.08E-54
rs2172745	T	TC	T	G	-0.2083	0.010436	0.2401	0.10927	5	1.58E+08	0.010862	0.34	5	1.58E+08	0.0293	1.09E-12
rs2206815	C	A	C	A	-0.3212	0.014663	0.5296	0.496878	20	10688540	0.006908	0.034	20	10688540	0.0257	6.34E-36
rs2240716	T	C	T	C	0.2045	-0.00793	0.2917	0.237512	22	19982173	0.008027	0.32	22	19982173	0.0271	4.49E-14
rs2241003	G	C	G	C	-0.156	0.006242	0.6014	0.473823	9	1.21E+08	0.006801	0.36	9	1.21E+08	0.0249	3.94E-10
rs2254240	A	G	A	G	-0.2275	-4.25E-05	0.2321	0.565562	11	47335619	0.006675	0.99	11	47335619	0.0301	4.41E-14
rs2282978	T	C	T	C	0.2988	0.011835	0.6456	0.869597	7	92635096	0.010177	0.24	7	92635096	0.0252	2.36E-32
rs2286525	G	A	G	A	0.1853	-0.00893	0.7332	0.399616	17	61394762	0.006965	0.2	17	61394762	0.0295	3.41E-10
rs2286870	G	A	G	A	0.192	0.005025	0.6454	0.519212	17	1468702	0.006875	0.46	17	1468702	0.0266	5.56E-13
rs2289125	A	C	A	C	-0.3421	0.004962	0.3076	0.439001	11	89491285	0.006928	0.47	11	89491285	0.0308	1.19E-28
rs2302417	A	T	A	T	0.14	0.000204	0.4666	0.3744	3	52780240	0.007073	0.98	3	52780240	0.0249	1.82E-08
rs2306363	T	G	T	G	-0.1782	0.012567	0.1948	0.252882	11	65638129	0.007743	0.1	11	65638129	0.0323	3.34E-08
rs2325885	T	C	T	C	-0.1969	-0.00582	0.2368	0.183718	17	48213013	0.008804	0.51	17	48213013	0.0228	6.69E-18
rs2366264	T	G	T	G	-0.183	0.002839	0.4894	0.28122	2	85261888	0.00752	0.71	2	85261888	0.0251	2.93E-13
rs2428939	A	G	A	G	0.1352	-0.0063	0.6344	0.709894	7	77932977	0.007489	0.4	7	77932977	0.0202	2.08E-11
rs2498323	A	G	A	G	0.3315	-0.03923	0.0983	0.042507	4	3449382	0.016883	0.02	4	3449382	0.0407	3.88E-16
rs2530244	A	G	A	G	0.1156	-0.00135	0.4934	0.519452	5	1.41E+08	0.006889	0.84	5	1.41E+08	0.0195	2.93E-09
rs255299	T	C	T	C	-0.1115	-0.00198	0.5572	0.213497	5	1.73E+08	0.008171	0.81	5	1.73E+08	0.0195	1.12E-08
rs256837	A	C	A	C	-0.148	0.016968	0.1941	0.024496	5	1.57E+08	0.02083	0.42	5	1.57E+08	0.0248	2.49E-09
rs2569842	T	C	T	C	0.1429	0.007965	0.2847	0.532661	6	1648798	0.006814	0.24	6	1648798	0.0213	2.00E-11
rs2643826	C	T	C	T	0.2353	-0.00431	0.457	0.710615	3	27521497	0.007423	0.56	3	27521497	0.0273	6.11E-18
rs267540	A	G	A	G	-0.1274	-0.00712	0.6007	0.837896	3	37540352	0.009161	0.44	3	37540352	0.0195	6.15E-11
rs2820444	A	G	A	G	-0.1621	0.000711	0.2801	0.217819	1	2.2E+08	0.008236	0.93	1	2.2E+08	0.0218	9.52E-14
rs2889121	T	C	T	C	0.254	-0.00968	0.2931	0.413305	5	1.23E+08	0.006932	0.16	5	1.23E+08	0.0269	3.45E-21
rs2911268	A	G	A	G	-0.184	0.018528	0.2289	0.151297	16	89441587	0.009508	0.051	16	89441587	0.033	2.57E-08
rs2971669	T	C	T	C	0.2183	-0.00184	0.2754	0.350865	7	44192179	0.007125	0.8	7	44192179	0.029	5.31E-14
rs2978456	C	T	C	T	-0.1907	-9.27E-06	0.5473	0.419308	8	42467247	0.006968	1	8	42467247	0.0271	1.95E-12

rs3109083	G	T	G	T	-0.1667	0.00958	0.7154	0.259846	2	1.77E+08	0.007884	0.22	2	1.77E+08	0.0301	3.15E-08
rs3176336	A	T	A	T	0.1609	0.001865	0.5666	0.316763	6	36681039	0.007267	0.8	6	36681039	0.0198	3.90E-16
rs3421960	A	G	A	G	-0.1681	0.005361	0.8531	0.855908	12	24607522	0.009772	0.58	12	24607522	0.0273	7.41E-10
rs35436	AT	A	A	G	0.1385	-0.0097	0.6052	0.291787	12	1.15E+08	0.007503	0.2	12	1.15E+08	0.0249	2.71E-08
rs356926	A	G	A	G	-0.1499	0.001291	0.1575	0.128722	18	28749090	0.010103	0.9	18	28749090	0.027	2.95E-08
rs3570285	C	G	C	G	-0.1798	0.003246	0.1982	0.170269	11	841284	0.008908	0.72	11	841284	0.0318	1.53E-08
rs3611438	A	G	A	G	0.1665	0.014019	0.8741	0.963497	6	84509360	0.018133	0.44	6	84509360	0.0302	3.47E-08
rs365990	G	A	G	A	0.2302	0.000975	0.5933	0.179635	14	23392602	0.008878	0.91	14	23392602	0.0256	2.34E-19
rs3740781	T	C	T	C	0.2127	0.00827	0.663	0.307157	11	45234375	0.007299	0.26	11	45234375	0.0272	5.67E-15
rs3767199	T	G	T	G	-0.1206	0.003877	0.3563	0.119116	1	1.8E+08	0.010369	0.71	1	1.8E+08	0.0202	2.41E-09
rs3798293	G	A	G	A	-0.1884	0.016233	0.7952	0.038184	6	96585494	0.01734	0.35	6	96585494	0.0324	5.90E-09
rs379862	T	G	T	G	-0.1184	-0.00589	0.6296	0.198607	19	34353658	0.008459	0.49	19	34353658	0.0211	2.03E-08
rs4073778	A	C	A	C	-0.1839	-0.00999	0.5529	0.334774	1	1.16E+08	0.007253	0.17	1	1.16E+08	0.0262	2.38E-12
rs4534535	T	C	T	C	-0.1739	0.000973	0.1384	0.557157	11	46767141	0.006817	0.89	11	46767141	0.0292	2.55E-09
rs464605	T	C	T	C	0.1332	-0.01101	0.7251	0.497839	5	56511543	0.006815	0.11	5	56511543	0.0214	4.42E-10
rs4744239	T	C	T	C	0.1152	-0.01078	0.5758	0.553554	9	93404100	0.006784	0.11	9	93404100	0.0197	5.31E-09
rs4795641	T	C	T	C	0.114	-0.01432	0.4935	0.372238	17	31760281	0.007092	0.043	17	31760281	0.0194	4.25E-09
rs4803455	A	C	A	C	-0.1617	0.001342	0.4959	0.357349	19	41345604	0.007103	0.85	19	41345604	0.0248	7.58E-11
rs4852780	T	C	T	C	0.1526	-0.01013	0.4262	0.148655	2	71392981	0.009532	0.29	2	71392981	0.0255	2.12E-09
rs4894055	A	G	A	G	-0.2589	-0.03337	0.086	0.974784	2	1.79E+08	0.021555	0.12	2	1.79E+08	0.0453	1.12E-08
rs499594	C	T	C	T	-0.1479	-0.01697	0.6926	0.089577	6	54111115	0.011897	0.15	6	54111115	0.0265	2.28E-08
rs5021979	T	C	T	C	-0.1203	-0.01178	0.7046	0.894332	8	1.16E+08	0.010596	0.27	8	1.16E+08	0.0218	3.61E-08
rs5582407	A	G	A	G	0.1923	-0.01173	0.153	0.016811	4	1.74E+08	0.025443	0.64	4	1.74E+08	0.0351	4.49E-08
rs5600066	C	A	C	A	0.1444	0.007532	0.6284	0.200048	17	75882573	0.008553	0.38	17	75882573	0.0263	3.93E-08
rs5602465	T	C	T	C	0.2303	0.014517	0.1807	0.197887	4	1.47E+08	0.008739	0.097	4	1.47E+08	0.033	3.04E-12
rs5628872	G	A	G	A	-0.2657	-0.01978	0.6049	0.310999	17	62689774	0.007321	0.0069	17	62689774	0.0256	3.00E-25
rs5635638	T	C	T	C	0.1561	-0.01118	0.7853	0.966138	19	4064059	0.018489	0.55	19	4064059	0.0243	1.37E-10
rs5772	C	G	C	G	-0.1188	0.00583	0.596	0.571326	8	1.09E+08	0.006893	0.4	8	1.09E+08	0.0193	7.68E-10
rs5847721	T	C	T	C	0.1437	-0.00052	0.2099	0.169789	4	1.03E+08	0.00915	0.95	4	1.03E+08	0.0248	6.48E-09
rs5864682	T	C	T	C	-0.2414	0.010878	0.2403	0.262488	15	62513935	0.007821	0.16	15	62513935	0.032	4.41E-14
rs5977682	T	C	T	C	0.2076	-0.00503	0.1634	0.09318	3	13785388	0.011818	0.67	3	13785388	0.0338	8.35E-10
rs5988496	T	C	T	C	0.2646	-0.00806	0.1864	0.223343	21	28753586	0.008189	0.32	21	28753586	0.0326	4.48E-16
rs6031431	G	A	G	A	-0.1523	0.001936	0.5416	0.398655	20	44166512	0.007024	0.78	20	44166512	0.025	1.08E-09
rs6090040	A	C	A	C	0.2159	0.001736	0.4804	0.358069	20	64060707	0.007143	0.81	20	64060707	0.0249	3.74E-18
rs6142381	A	G	A	G	-0.1471	0.002271	0.5966	0.673631	20	35420820	0.007121	0.75	20	35420820	0.0195	5.27E-14
rs6174863	C	G	C	G	-0.2793	0.012528	0.9302	0.916427	1	28466638	0.012412	0.31	1	28466638	0.0415	1.75E-11
rs6177738	T	C	T	C	0.1479	-0.02906	0.2982	0.021614	1	42536084	0.022824	0.2	1	42536084	0.0215	5.80E-12
rs6180887	A	G	A	G	-0.2267	-0.00667	0.3154	0.360951	1	1.69E+08	0.006874	0.33	1	1.69E+08	0.0269	3.61E-17
rs6182300	G	A	G	A	0.3774	-0.00419	0.9507	0.049712	1	1.77E+08	0.015951	0.79	1	1.77E+08	0.0594	2.04E-10
rs6202076	T	C	T	C	-0.1209	-0.00498	0.4898	0.3744	15	92737261	0.007055	0.48	15	92737261	0.0197	9.06E-10
rs6203340	A	G	A	G	-0.1219	0.000526	0.5983	0.504083	16	53790314	0.006837	0.94	16	53790314	0.0197	6.36E-10
rs6215551	G	A	G	A	0.1526	-0.00277	0.6164	0.443324	2	95991827	0.006849	0.69	2	95991827	0.0255	2.21E-09

rs6225071	A	G	A	G	0.1309	-0.01306	0.6008	0.802834	3	85466626	0.008263	0.11	3	85466626	0.0197	2.99E-11
rs6233428	A	C	A	C	0.2171	-0.00986	0.3944	0.303794	4	1.69E+08	0.007476	0.19	4	1.69E+08	0.0253	8.78E-18
rs6421389	C	G	C	G	-0.1173	-0.00625	0.5015	0.938521	10	88958770	0.013782	0.65	10	88958770	0.0199	3.59E-09
rs6511029	T	C	C	A	0.1871	0.022826	0.1682	0.036984	19	19360610	0.017355	0.19	19	19360610	0.0326	8.95E-09
rs6535419	A	G	A	G	0.1329	-6.51E-05	0.7117	0.478146	4	82969822	0.006849	0.99	4	82969822	0.0215	6.22E-10
rs6539284	T	C	T	C	0.1279	0.002841	0.5863	0.872719	12	79198900	0.010176	0.78	12	79198900	0.0202	2.26E-10
rs6595838	A	G	A	G	0.2073	0.010828	0.351	0.229347	5	1.29E+08	0.008076	0.18	5	1.29E+08	0.0268	9.45E-15
rs6599251	T	G	T	G	-0.1241	-1.40E-05	0.5548	0.612152	3	38744318	0.007167	1	3	38744318	0.0192	1.10E-10
rs6662330	T	C	T	C	0.1426	-0.00112	0.218	0.17243	1	66553045	0.008948	0.9	1	66553045	0.0236	1.42E-09
rs666720	T	C	T	C	-0.1165	0.007869	0.5439	0.378963	1	45555123	0.007093	0.27	1	45555123	0.0193	1.56E-09
rs6679045	A	G	A	G	0.438	0.043003	0.0774	0.023295	6	26106860	0.021858	0.049	6	26106860	0.0476	3.82E-20
rs6679817	T	C	T	C	0.1533	0.005988	0.3421	0.304275	1	88897581	0.007367	0.42	1	88897581	0.0267	9.02E-09
rs6730325	G	A	G	A	-0.1599	0.000304	0.638	0.152257	2	59088693	0.009535	0.97	2	59088693	0.0259	6.61E-10
rs6742041	A	G	A	G	0.1167	0.000555	0.693	0.597502	2	59841443	0.006948	0.94	2	59841443	0.0208	2.08E-08
rs6763107	T	C	T	C	0.2351	0.01651	0.5008	0.316042	1	37996149	0.007477	0.027	1	37996149	0.0254	1.93E-20
rs6766170	A	C	A	C	-0.1423	-0.00962	0.4843	0.261527	3	14837323	0.007733	0.21	3	14837323	0.0252	1.55E-08
rs6792918	A	C	A	C	0.2907	0.00037	0.9449	0.942603	3	44815512	0.014243	0.98	3	44815512	0.0429	1.21E-11
rs6797165	G	A	G	A	0.5241	-0.00536	0.7233	0.175552	3	41846285	0.009095	0.56	3	41846285	0.0312	2.21E-63
rs6848906	T	C	T	C	0.1817	0.003272	0.1864	0.388569	4	1.1E+08	0.006933	0.64	4	1.1E+08	0.0318	1.09E-08
rs6996562	A	C	A	C	0.1235	-0.01142	0.4732	0.641931	8	33534505	0.007088	0.11	8	33534505	0.0193	1.65E-10
rs704191	T	C	T	C	0.1492	0.018898	0.4449	0.342699	12	21862088	0.007274	0.0094	12	21862088	0.0195	1.73E-14
rs7095095	G	A	G	A	0.1809	0.008106	0.8071	0.103746	10	31072535	0.010982	0.46	10	31072535	0.0313	7.59E-09
rs7134677	T	C	T	C	-0.1695	-0.00687	0.2926	0.394092	12	54047714	0.007002	0.33	12	54047714	0.0275	6.71E-10
rs7158073	T	C	T	C	0.1877	-0.00086	0.453	0.375841	14	99675352	0.006909	0.9	14	99675352	0.0245	1.67E-14
rs7166269	T	C	T	C	0.1579	0.009967	0.7805	0.809318	15	85423058	0.008811	0.26	15	85423058	0.0252	3.82E-10
rs7197770	T	TTCTG	G	A	0.2026	-0.00435	0.7085	0.178674	16	30704587	0.008177	0.59	16	30704587	0.0298	9.96E-12
rs7219390	T	C	T	C	0.1067	-0.00128	0.575	0.64001	17	76688776	0.006991	0.85	17	76688776	0.0193	3.17E-08
rs7226020	C	T	C	T	-0.2759	-0.00126	0.5726	0.364313	17	6570508	0.007062	0.86	17	6570508	0.0264	1.61E-25
rs7315818	T	C	T	C	0.1837	-0.00169	0.2725	0.044669	7	1.52E+08	0.016346	0.92	7	1.52E+08	0.028	5.30E-11
rs7318734	T	C	T	C	0.2407	0.008025	0.0583	0.911383	7	1.07E+08	0.012013	0.5	7	1.07E+08	0.0418	8.25E-09
rs7320104	G	A	G	A	-0.2005	-0.00642	0.7861	0.069885	13	1.14E+08	0.013213	0.63	13	1.14E+08	0.0329	1.14E-09
rs7375405	A	T	A	T	0.1284	0.002012	0.2599	0.237272	5	52860947	0.008078	0.8	5	52860947	0.0219	4.41E-09
rs7412	T	C	T	C	-0.3606	-0.01288	0.0833	0.081172	19	44908822	0.012548	0.3	19	44908822	0.0356	3.71E-24
rs7418129	C	T	C	T	0.23	-0.00131	0.5797	0.32829	2	65056838	0.007166	0.86	2	65056838	0.0251	6.03E-20
rs7500448	G	A	G	A	0.3198	0.013632	0.7611	0.254563	16	83012185	0.007926	0.085	16	83012185	0.031	5.87E-25
rs7502355	ACT	A	A	G	-0.2349	0.012348	0.842	0.249039	15	78859743	0.007784	0.11	15	78859743	0.0336	2.57E-12
rs7512595	A	G	A	G	0.204	-0.01532	0.8558	0.98463	1	27530660	0.027663	0.58	1	27530660	0.0321	1.96E-10
rs7543389	G	A	G	A	0.3066	0.002763	0.7371	0.181316	1	59214864	0.008824	0.75	1	59214864	0.0287	1.26E-26
rs757462	T	C	T	C	-0.1346	-0.01605	0.6507	0.725264	16	4246784	0.007512	0.033	16	4246784	0.021	1.48E-10
rs7620672	A	G	A	G	-0.4336	-0.0103	0.1069	0.464217	7	40408372	0.006803	0.13	7	40408372	0.0423	1.18E-24
rs7632108	A	C	A	C	0.1326	0.011432	0.4674	0.879683	3	37216572	0.010512	0.28	3	37216572	0.0195	1.11E-11
rs7671947	C	T	C	T	0.4471	0.003797	0.8744	0.196686	4	53934592	0.008571	0.66	4	53934592	0.0398	2.89E-29

rs7672622	A	G	A	G	0.1327	0.000198	0.7358	0.716859	4	1.57E+08	0.0075	0.98	4	1.57E+08	0.0227	5.09E-09
rs7707918	C	A	C	A	-0.212	-0.01027	0.6171	0.298991	5	1.59E+08	0.007395	0.17	5	1.59E+08	0.0256	1.15E-16
rs7727978	A	C	A	C	-0.1212	-0.00494	0.6809	0.95269	5	1.2E+08	0.014691	0.74	5	1.2E+08	0.0207	5.25E-09
rs7752448	G	A	G	A	-0.3397	0.006572	0.9087	0.088136	6	28333322	0.011926	0.58	6	28333322	0.0436	6.22E-15
rs7757437	T	C	T	C	-0.1771	0.010416	0.8465	0.961095	9	19105640	0.017672	0.56	9	19105640	0.0271	6.58E-11
rs7813925	T	C	T	C	0.2697	0.009638	0.0892	0.131364	17	41800770	0.010349	0.35	17	41800770	0.0465	6.70E-09
rs7914598	T	C	T	C	-0.2319	0.007636	0.1769	0.269452	11	47839194	0.007684	0.32	11	47839194	0.035	3.28E-11
rs7968719	C	G	C	G	-0.1197	-0.0072	0.4861	0.676753	12	57146968	0.007213	0.32	12	57146968	0.0203	3.55E-09
rs8013933	T	C	T	C	0.122	-0.00977	0.703	0.646254	14	93999443	0.007198	0.17	14	93999443	0.0214	1.19E-08
rs8027524	T	C	T	C	0.1182	0.002134	0.3191	0.353506	15	49985422	0.007073	0.76	15	49985422	0.0206	8.80E-09
rs8030926	A	G	A	G	0.4663	0.020459	0.2092	0.947647	8	1.19E+08	0.015085	0.18	8	1.19E+08	0.0299	5.28E-55
rs8044595	G	A	G	A	0.1446	-0.00944	0.63	0.291547	16	15812273	0.007452	0.21	16	15812273	0.0262	3.53E-08
rs8102624	A	G	A	G	0.4062	-0.00585	0.1779	0.279059	19	2161444	0.007883	0.46	19	2161444	0.0395	9.03E-25
rs9289798	G	T	G	T	-0.1595	0.010157	0.6947	0.284102	3	1.5E+08	0.007516	0.18	3	1.5E+08	0.0266	1.93E-09
rs9305545	A	G	A	G	-0.1708	0.005685	0.1251	0.989914	21	34223520	0.032953	0.86	21	34223520	0.0311	4.07E-08
rs9337951	A	G	A	G	0.3282	0.019417	0.317	0.032901	10	30028144	0.018581	0.3	10	30028144	0.0298	3.23E-28
rs9340995	C	G	C	G	0.4665	-0.00757	0.1236	0.379683	6	1.52E+08	0.006839	0.27	6	1.52E+08	0.0397	6.11E-32
rs9349379	G	A	G	A	0.2448	0.010662	0.6014	0.65634	6	12903725	0.007174	0.14	6	12903725	0.0264	2.08E-20
rs9373387	G	A	G	A	-0.1856	0.00255	0.6348	0.01609	6	1.43E+08	0.024961	0.92	6	1.43E+08	0.0269	5.50E-12
rs9477927	T	C	T	C	0.1044	0.000258	0.561	0.563881	6	18913149	0.006975	0.97	6	18913149	0.0191	4.50E-08
rs9506725	T	C	T	C	0.1175	0.045487	0.6475	0.987032	13	21740007	0.029433	0.12	13	21740007	0.0198	2.90E-09
rs9549328	T	C	T	C	0.1996	0.010204	0.2244	0.235591	13	1.13E+08	0.008058	0.21	13	1.13E+08	0.0295	1.36E-11
rs9633643	T	C	T	C	0.17	0.003182	0.2236	0.257205	10	20224618	0.007756	0.68	10	20224618	0.0293	6.56E-09
rs9658584	C	G	C	G	-0.1421	0.001871	0.2018	0.132085	1	85581628	0.01025	0.86	1	85581628	0.0246	7.41E-09
rs9662255	A	C	A	C	-0.241	0.011152	0.4075	0.440922	1	9381890	0.006932	0.11	1	9381890	0.0256	4.68E-21
rs968267	G	A	G	A	0.1733	0.012209	0.6093	0.198367	2	9157461	0.008638	0.16	2	9157461	0.0281	7.16E-10
rs9708177	T	C	T	C	0.308	0.01968	0.0791	0.070845	15	90105840	0.0134	0.14	15	90105840	0.0501	7.64E-10
rs9937309	C	G	C	G	0.1181	-0.00578	0.2925	0.217819	16	11018255	0.008291	0.49	16	11018255	0.0209	1.70E-08
rs998382	T	G	T	C	-0.1418	0.001728	0.5957	0.098703	20	32796330	0.011335	0.88	20	32796330	0.0258	3.91E-08

Supplementary Table 2i. List of SNPs for Hypertension and their effects on TEWL

SNP	effect_alle	other_allel	effect_alle	other_allel	beta.hyper	beta.TEWL	eaf.hypert	eaf.TEWL	chr.TEWL	pos.TEWL	se.TEWL	pval.TEWL	chr.hypert	pos.hypert	se.hyperte	pval.hypertension
chr12:895	G	A	G	A	0.15	-0.00357	0.18	0.191883	12	89548613	0.0085	0.67	12	88466521	0.03	7.10E-07
chr12:896	C	T	C	T	0.14	-0.00108	0.19	0.29635	12	89619312	0.007286	0.88	12	88537220	0.03	8.40E-08
chr12:896	C	G	C	G	0.16	-0.00059	0.17	0.299712	12	89680664	0.007289	0.94	12	88598572	0.03	2.20E-08
rs1101416	T	A	T	A	0.11	-0.00533	0.17	0.158021	10	18419869	0.009171	0.56	10	18748804	0.02	7.80E-07
rs1110535	G	A	G	A	0.16	-0.00055	0.17	0.29635	12	89632746	0.007286	0.94	12	88550654	0.03	1.80E-08
rs1110536	G	T	G	T	0.16	-0.00059	0.17	0.299712	12	89675499	0.007289	0.94	12	88593407	0.03	2.10E-08
rs1110537	T	C	T	C	0.17	0.001347	0.16	0.293708	12	89696964	0.00735	0.85	12	88614872	0.03	2.80E-08
rs1223007	G	A	G	A	0.17	0.001554	0.16	0.293948	12	89697090	0.007351	0.83	12	88614998	0.03	2.80E-08
rs1257930	G	A	G	A	0.16	-0.00064	0.17	0.29731	12	89656726	0.007297	0.93	12	88574634	0.03	2.20E-08
rs1724975	A	G	A	G	0.16	-0.00052	0.17	0.299232	12	89666809	0.007288	0.94	12	88584717	0.03	2.20E-08
rs2681472	G	A	G	A	0.16	-0.00034	0.17	0.295629	12	89615182	0.007292	0.96	12	88533090	0.03	1.70E-08
rs4842666	C	T	C	T	0.15	0.003274	0.17	0.3122	12	89547772	0.007257	0.65	12	88465680	0.03	3.40E-07
rs743395	T	C	T	C	0.12	0.001795	0.38	0.111671	3	37556891	0.010542	0.86	3	37573386	0.02	7.50E-07
rs7640747	G	C	G	C	0.12	0.009521	0.38	0.160663	3	37555314	0.009211	0.3	3	37571809	0.02	4.80E-07

Supplementary Table 2j. List of SNPs for Heart Rate and their effects on TEWL

SNP	effect_alle	other_allel	effect_alle	other_allel	beta.HR	beta.TEWL	eaf.HR	eaf.TEWL	chr.TEWL	pos.TEWL	se.TEWL	pval.TEWL	chr.HR	pos.HR	se.HR	pval.HR
chr1:8742	A	G	A	G	-0.3061	-0.0122	0.33	0.46902	1	87427449	0.006868	0.076	1	87427449	0.0355	7.23E-18
chr14:853	G	A	G	A	-0.4096	0.017411	0.28	0.085255	14	85330220	0.012251	0.16	14	85330220	0.0367	6.55E-29
chr16:652	C	T	C	T	-0.2907	0.004106	0.43	0.183958	16	65252967	0.008434	0.63	16	65252967	0.0334	3.58E-18
chr2:1591	C	A	C	A	-0.1844	-0.0118	0.27	0.068444	2	1.59E+08	0.013047	0.37	2	1.59E+08	0.0372	7.00E-07
chr2:2272	C	T	C	T	0.2486	-0.00204	0.33	0.598463	2	2.27E+08	0.006973	0.77	2	2.27E+08	0.0353	1.85E-12
chr22:277	T	C	T	C	-0.1741	0.002381	0.45	0.401537	22	27785411	0.007065	0.74	22	27785411	0.0348	5.48E-07
chr6:7542	T	A	C	A	0.2403	-0.00581	0.16	0.016571	6	7542890	0.026328	0.83	6	7542890	0.0459	1.60E-07
chr7:1273	C	G	C	G	-0.2311	0.010075	0.27	0.93684	7	1.27E+08	0.014133	0.48	7	1.27E+08	0.0371	4.42E-10
rs1050288	T	C	T	C	-0.2125	0.01411	0.34	0.634486	12	27802363	0.007029	0.045	12	27802363	0.0357	2.74E-09
rs1073966	G	A	G	A	-0.2656	0.006537	0.45	0.346061	9	1.26E+08	0.007371	0.38	9	1.26E+08	0.0331	9.62E-16
rs1082061	C	G	C	G	-0.1854	-0.01872	0.21	0.039866	9	96433812	0.01725	0.28	9	96433812	0.0408	5.65E-06
rs1084148	C	T	C	T	-0.2382	0.000151	0.22	0.034102	12	20319268	0.018959	0.99	12	20319268	0.0401	2.98E-09
rs1108176	A	G	A	G	-0.2669	-0.00199	0.15	0.341979	18	32423359	0.007083	0.78	18	32423359	0.0469	1.25E-08
rs1108325	C	A	C	A	-0.2761	-0.01515	0.17	0.017531	18	28186254	0.025998	0.56	18	28186254	0.0445	5.51E-10
rs1178673	A	G	A	G	-0.6662	0.049782	0.04	0.993036	8	1.44E+08	0.040028	0.21	8	1.44E+08	0.0831	1.09E-15
rs1192057	A	G	A	G	0.2676	-0.00383	0.26	0.231028	3	1.22E+08	0.00818	0.64	3	1.22E+08	0.0371	5.18E-13
rs1250103	G	C	G	C	0.2878	0.006766	0.31	0.087416	4	23949395	0.011861	0.57	4	23949395	0.0362	1.83E-15
rs1257632	G	A	G	A	0.2528	0.002017	0.34	0.270893	11	44958832	0.007765	0.8	11	44958832	0.0356	1.20E-12
rs1257975	T	C	T	C	-0.2456	-0.02799	0.23	0.054275	12	81825597	0.015081	0.063	12	81825597	0.0394	4.81E-10
rs1260326	C	T	C	T	-0.2746	0.003182	0.39	0.538665	2	27508073	0.006816	0.64	2	27508073	0.0338	4.29E-16
rs1271340	T	G	T	G	-0.1992	0.000591	0.38	0.60951	2	59779570	0.006894	0.93	2	59779570	0.0347	9.33E-09
rs1272105	G	C	G	C	-0.2874	0.005947	0.18	0.104947	19	44918903	0.011143	0.59	19	44918903	0.0438	5.23E-11
rs1288926	G	A	G	A	0.4164	0.012985	0.16	0.047791	14	21074607	0.01581	0.41	14	21074607	0.0453	3.61E-20
rs1294135	G	A	G	A	-0.1809	0.008668	0.42	0.101345	17	17813217	0.011203	0.44	17	17813217	0.0333	5.49E-08
rs1300273	C	A	C	A	-0.3305	0.00048	0.24	0.262488	2	2.31E+08	0.007668	0.95	2	2.31E+08	0.0387	1.29E-17
rs1316553	T	A	T	A	-0.221	-0.00012	0.42	0.45389	5	30888476	0.006977	0.99	5	30888476	0.0335	4.31E-11
rs1320761	T	C	T	C	0.9018	0.000609	0.11	0.423391	6	1.22E+08	0.006678	0.93	6	1.22E+08	0.0531	1.22E-64
rs1456482	C	A	C	T	0.4038	0.005705	0.05	0.051153	18	22535366	0.015604	0.71	18	22535366	0.0768	1.48E-07
rs1483890	G	A	G	A	0.2836	0.007893	0.3	0.386167	3	69361574	0.006981	0.26	3	69361574	0.0359	2.54E-15
rs1549118	T	C	T	C	0.2003	0.003687	0.28	0.173631	14	77913341	0.008861	0.68	14	77913341	0.0367	4.67E-08
rs1592560	C	A	C	A	-0.1826	-0.00746	0.4	0.355187	16	10717830	0.007031	0.29	16	10717830	0.0346	1.34E-07
rs1697419	A	G	A	G	0.2436	-0.00733	0.32	0.475264	19	40327563	0.006895	0.29	19	40327563	0.0359	1.11E-11
rs1707989	A	T	A	G	0.5196	0.002671	0.47	0.258405	6	1.18E+08	0.007944	0.74	6	1.18E+08	0.0332	2.95E-55
rs1718048	C	G	C	G	-0.4896	0.004829	0.14	0.021854	14	72418763	0.022483	0.83	14	72418763	0.0554	9.15E-19
rs1726551	C	T	C	T	0.2398	0.012738	0.19	0.006964	20	41203988	0.041471	0.76	20	41203988	0.042	1.12E-08
rs1770596	G	A	G	A	0.3365	-0.02722	0.19	0.011768	2	1.87E+08	0.030178	0.37	2	1.87E+08	0.0416	5.99E-16
rs1788169	A	G	A	G	0.5784	0.000948	0.18	0.053794	7	1.01E+08	0.01528	0.95	7	1.01E+08	0.0428	1.18E-41
rs180239	C	G	C	G	-0.326	0.006077	0.35	0.748799	7	93921103	0.007894	0.44	7	93921103	0.0346	4.54E-21
rs1994135	C	T	C	T	0.4	-0.0193	0.47	0.067003	12	33529470	0.013292	0.15	12	33529470	0.033	7.19E-34
rs2076028	A	G	A	G	-0.2949	0.005767	0.29	0.424592	22	38754445	0.006902	0.4	22	38754445	0.0364	5.45E-16
rs2283274	C	G	C	G	-0.4051	-0.01161	0.18	0.417627	12	2075300	0.006941	0.094	12	2075300	0.0444	7.21E-20

rs2358740	T	G	T	G	-0.2079	0.00295	0.32	0.283862	3	53421542	0.007502	0.69	3	53421542	0.0352	3.58E-09
rs236349	G	A	G	A	0.2812	-0.01168	0.34	0.645293	6	36852789	0.007079	0.099	6	36852789	0.035	1.01E-15
rs272564	C	A	C	A	0.351	0.00142	0.28	0.56196	1	44546601	0.006834	0.84	1	44546601	0.0373	4.51E-21
rs3749237	A	G	A	G	0.2583	0.001087	0.32	0.176753	3	49732599	0.009021	0.9	3	49732599	0.0354	3.09E-13
rs3915499	A	G	A	G	0.303	-0.00828	0.32	0.289145	16	15816886	0.007481	0.27	16	15816886	0.0354	1.24E-17
rs4131241	G	C	G	C	-0.32	-0.03654	0.15	0.022815	3	38579746	0.021683	0.092	3	38579746	0.047	1.34E-11
rs4131799	A	G	A	G	0.6304	-0.00717	0.1	0.07757	1	2.08E+08	0.01276	0.57	1	2.08E+08	0.0545	5.42E-31
rs41748	G	T	G	T	-0.1926	0.004076	0.45	0.455331	7	1.17E+08	0.006808	0.55	7	1.17E+08	0.0333	7.14E-09
rs422068	C	T	C	T	0.7307	0.001897	0.36	0.179875	14	23395595	0.008847	0.83	14	23395595	0.0343	1.52E-100
rs4868243	A	G	A	G	-0.361	-0.00862	0.16	0.501441	5	1.73E+08	0.006785	0.2	5	1.73E+08	0.0444	4.08E-16
rs4894055	T	G	G	A	1.0613	0.033372	0.09	0.025216	2	1.79E+08	0.021555	0.12	2	1.79E+08	0.058	7.86E-75
rs4900069	C	A	C	A	0.1999	-0.01022	0.37	0.87488	14	91117029	0.010355	0.32	14	91117029	0.0343	5.38E-09
rs4963772	A	G	A	G	-0.7139	-0.00586	0.15	0.144813	12	24605546	0.009723	0.55	12	24605546	0.0465	3.23E-53
rs5843797	C	T	C	T	-0.2402	0.000727	0.5	0.341739	7	35218665	0.007219	0.92	7	35218665	0.0343	2.61E-12
rs6123471	C	T	C	T	-0.5953	0.012959	0.46	0.516811	20	38211754	0.006816	0.057	20	38211754	0.0332	6.63E-72
rs6214405	C	T	C	T	0.2246	-0.00483	0.28	0.177714	2	66497930	0.008837	0.58	2	66497930	0.0429	1.62E-07
rs6845865	C	T	C	T	-0.3423	-0.00431	0.16	0.25048	4	1.48E+08	0.007973	0.59	4	1.48E+08	0.0448	2.25E-14
rs7173389	T	A	T	A	-0.5393	0.030323	0.16	0.037464	15	73371562	0.017709	0.087	15	73371562	0.0453	1.31E-32
rs7274760	CT	C	G	C	0.2558	0.000782	0.26	0.432277	1	2.18E+08	0.00679	0.91	1	2.18E+08	0.0378	1.29E-11
rs7315870	G	A	G	A	0.3929	0.005263	0.16	0.410903	7	1.37E+08	0.007063	0.46	7	1.37E+08	0.0451	2.81E-18
rs748802	A	G	A	G	-0.2022	-0.00592	0.34	0.277858	9	35909521	0.007602	0.44	9	35909521	0.0354	1.13E-08
rs7519094	A	C	A	C	-0.4962	0.018153	0.09	0.080452	11	1.29E+08	0.012517	0.15	11	1.29E+08	0.0599	1.19E-16
rs7612445	T	G	T	G	-0.4276	0.003415	0.19	0.178915	3	1.79E+08	0.008753	0.7	3	1.79E+08	0.042	2.41E-24
rs7912176	T	C	T	C	-0.471	0.033607	0.09	0.009846	17	15291962	0.034101	0.32	17	15291962	0.0629	7.17E-14
rs867400	C	T	C	T	0.2983	0.000376	0.43	0.259606	12	64583070	0.007791	0.96	12	64583070	0.0334	4.58E-19
rs907683	T	G	T	G	-0.3335	-0.00268	0.43	0.725744	2	2.19E+08	0.007561	0.72	2	2.19E+08	0.0357	1.02E-20

Supplementary Table 2k. List of SNPs for C-reactive Protein and their effects on TEWL

SNP	effect_alle	other_allel	effect_alle	other_allel	beta.CRP	beta.TEWL	eaf.CRP	eaf.TEWL	chr.TEWL	pos.TEWL	se.TEWL	pval.TEWL	chr.CRP	pos.CRP	se.CRP	pval.CRP
chr1:1006	A	G	A	G	0.0173	-0.00496	0.1501	0.68684	1	10065349	0.007336	0.5	1	10125407	0.003	4.65E-09
chr1:1502	T	G	T	G	0.012	-0.00184	0.07157	0.239193	1	1.5E+08	0.008093	0.82	1	1.5E+08	0.002	3.79E-09
chr1:1779	A	G	A	G	0.0154	0.001808	0.2227	0.188521	1	1.78E+08	0.008712	0.84	1	1.78E+08	0.0023	4.82E-11
chr1:1984	A	G	A	G	0.0256	0.012539	0.1352	0.446926	1	1.98E+08	0.006748	0.063	1	1.98E+08	0.0033	3.92E-15
chr1:2003	T	C	T	C	-0.0131	-0.01367	0.2992	0.700288	1	2E+08	0.007454	0.067	1	2E+08	0.0022	4.96E-09
chr1:2149	A	G	A	G	-0.0182	-0.01042	0.4016	0.619116	1	21493549	0.007167	0.15	1	21820042	0.0021	1.71E-17
chr1:4359	A	G	A	G	-0.0131	-0.00763	0.3539	0.193324	1	43594812	0.008642	0.38	1	44060483	0.002	1.25E-10
chr1:6265	T	C	T	C	0.0136	-0.0104	0.3012	0.769693	1	62651339	0.007988	0.19	1	63117010	0.0021	2.01E-10
chr1:7679	T	G	T	G	0.0114	0.01226	0.4105	0.303314	1	7679080	0.007407	0.098	1	7739140	0.002	2.16E-08
chr1:9106	A	C	A	C	0.0349	0.003627	0.1899	0.865514	1	91064875	0.010028	0.72	1	91530432	0.0025	8.68E-43
chr11:103	A	ACT	A	C	-0.0133	0.003412	0.4682	0.480788	11	10381992	0.006837	0.62	11	10404252	0.0023	1.35E-08
chr11:117	A	C	A	C	0.0118	0.010833	0.494	0.242075	11	11798902	0.007932	0.17	11	11820449	0.002	6.84E-09
chr11:162	A	T	A	T	0.0129	0.009725	0.4165	0.504803	11	16229705	0.006873	0.16	11	16251251	0.002	2.37E-10
chr11:555	A	G	A	G	-0.0294	0.007202	0.1322	0.150817	11	55583022	0.009373	0.44	11	55371381	0.0035	2.01E-17
chr11:617	C	G	C	G	-0.0145	0.001069	0.338	0.870797	11	61752514	0.010132	0.92	11	61519986	0.0021	1.19E-11
chr12:120	A	G	A	G	0.0823	-0.00472	0.3897	0.585255	12	1.21E+08	0.006838	0.49	12	1.21E+08	0.0021	0
chr12:215	T	C	T	C	0.0185	-0.00514	0.2296	0.244476	12	21557815	0.007965	0.52	12	21710749	0.0025	3.64E-13
chr12:459	T	C	T	C	0.0139	0.017158	0.2396	0.151297	12	45928666	0.00953	0.072	12	46322449	0.0024	1.28E-08
chr12:900	T	C	T	C	0.0159	0.003602	0.4851	0.316042	12	90068229	0.007395	0.63	12	90462006	0.002	5.79E-15
chr14:964	T	C	T	C	0.0198	-0.00444	0.2843	0.818444	14	96467077	0.008905	0.62	14	96933414	0.0022	9.60E-19
chr15:583	A	G	A	G	0.0147	-0.00044	0.3648	0.463497	15	58391167	0.006731	0.95	15	58683366	0.002	5.23E-13
chr16:211	A	G	A	G	-0.0317	-0.03779	0.07157	0.009846	16	2119457	0.034911	0.28	16	2169458	0.0042	3.10E-14
chr16:537	A	G	A	G	-0.0266	-0.0025	0.4344	0.846302	16	53772541	0.009547	0.79	16	53806453	0.002	5.35E-39
chr16:839	T	C	T	C	-0.0165	0.004349	0.3837	0.209654	16	83945712	0.008289	0.6	16	83979317	0.0021	1.19E-14
chr17:161	T	C	T	C	0.0158	0.008833	0.4344	0.229827	17	16180370	0.008021	0.27	17	16083684	0.002	8.54E-15
chr17:639	T	C	T	C	0.0152	0.014919	0.2227	0.919549	17	63946431	0.012465	0.23	17	62023791	0.0026	9.36E-09
chr17:702	A	C	A	C	0.0166	-0.00188	0.2744	0.438521	17	70261597	0.006873	0.78	17	68257738	0.0023	1.35E-12
chr17:747	T	C	T	C	0.0281	-0.00871	0.2734	0.548031	17	74706775	0.006892	0.21	17	72702914	0.0023	3.56E-33
chr17:783	A	G	A	G	-0.0334	-0.00959	0.2137	0.48487	17	78361793	0.006762	0.16	17	76357874	0.0027	5.74E-34
chr18:127	T	C	T	C	-0.0237	-0.00582	0.4294	0.599424	18	12783899	0.007099	0.41	18	12783898	0.002	2.61E-31
chr18:481	T	C	T	C	0.0141	-0.00801	0.3489	0.582373	18	48137416	0.006984	0.25	18	45663787	0.0021	4.26E-11
chr19:350	T	C	T	C	-0.0305	-0.03135	0.06163	0.865994	19	35062437	0.009957	0.0016	19	35553341	0.0041	6.93E-14
chr19:357	T	C	T	C	-0.0118	-0.01016	0.3519	0.481028	19	35785184	0.006763	0.13	19	36276086	0.0021	3.41E-08
chr19:512	T	C	T	C	-0.02	0.000255	0.3101	0.182757	19	51225221	0.008829	0.98	19	51728477	0.0021	8.43E-21
chr19:807	C	G	C	G	-0.0132	0.001355	0.3648	0.576609	19	807442	0.006971	0.85	19	807442	0.0022	3.79E-09
chr2:1021	A	G	A	G	0.0223	-0.01644	0.2505	0.210134	2	1.02E+08	0.008424	0.051	2	1.03E+08	0.0023	1.68E-21
chr2:1773	T	C	T	C	0.0169	0.000218	0.2207	0.103026	2	1.77E+08	0.011083	0.98	2	1.78E+08	0.0024	4.64E-12
chr2:2131	A	G	A	G	0.021	0.005907	0.3936	0.091979	2	2.13E+08	0.011438	0.61	2	2.14E+08	0.002	6.16E-25
chr2:2314	T	C	T	C	-0.0133	-0.00159	0.3748	0.383766	2	2.31E+08	0.007108	0.82	2	2.32E+08	0.002	6.51E-11
chr2:2490	A	T	A	T	-0.0186	-0.00515	0.4274	0.602305	2	24907571	0.007067	0.47	2	25130440	0.002	6.58E-20
chr2:3683	C	G	C	G	-0.0144	-0.00442	0.499	0.695245	2	36832414	0.007345	0.55	2	37059557	0.002	1.53E-12

chr2:5907	A	C	A	C	0.0124	0.015232	0.3797	0.355668	2	59071163	0.007063	0.031	2	59298298	0.002	1.13E-09
chr2:6325	T	C	T	C	-0.026	0.004532	0.172	0.079011	2	632591	0.01284	0.72	2	632591	0.0026	9.06E-23
chr2:7227	C	G	C	G	-0.0161	0.031829	0.1123	0.039625	2	72273556	0.017497	0.069	2	72500685	0.0029	1.63E-08
chr21:390	A	C	A	C	-0.0344	0.007081	0.2694	0.169068	21	39093140	0.009051	0.43	21	40465066	0.0022	3.14E-53
chr21:450	T	C	T	C	0.0122	-0.02258	0.3569	0.135447	21	45075080	0.00997	0.024	21	46494995	0.0021	1.16E-08
chr22:410	T	C	T	C	-0.0195	0.001666	0.3529	0.412824	22	41013425	0.006859	0.81	22	41409429	0.0021	7.49E-20
chr3:1709	A	G	A	G	0.0196	0.002966	0.2883	0.246398	3	1.71E+08	0.007983	0.71	3	1.71E+08	0.0022	2.13E-18
chr3:2532	A	G	A	G	0.0113	0.010215	0.4105	0.146254	3	25321672	0.009694	0.29	3	25363163	0.002	2.87E-08
chr3:3695	T	C	T	C	-0.0112	0.001469	0.4702	0.064842	3	36957264	0.013675	0.91	3	36998755	0.002	3.79E-08
chr4:1480	C	G	C	G	-0.0138	0.002168	0.3807	0.831172	4	1.48E+08	0.008871	0.81	4	1.49E+08	0.0023	3.79E-09
chr4:3876	T	C	T	C	0.0137	0.027752	0.3181	0.991354	4	38764099	0.036294	0.44	4	38765720	0.0021	1.48E-10
chr5:5689	T	C	T	C	-0.0188	0.00325	0.1561	0.396013	5	56898941	0.006895	0.64	5	56194768	0.0029	4.26E-11
chr5:7368	A	G	A	G	0.0158	0.010303	0.2922	0.82781	5	73683611	0.00905	0.25	5	72979436	0.0022	1.74E-12
chr6:1265	T	C	T	C	-0.0279	-0.00817	0.493	0.929155	6	1.27E+08	0.012646	0.52	6	1.27E+08	0.002	9.94E-43
chr6:1300	C	G	C	G	-0.0224	0.000499	0.3211	0.168828	6	1.3E+08	0.009103	0.96	6	1.3E+08	0.0021	1.11E-25
chr6:1339	T	C	T	C	0.0116	-0.0011	0.497	0.844621	6	1.34E+08	0.009368	0.91	6	1.34E+08	0.002	1.22E-08
chr6:1496	A	G	A	G	-0.0119	0.005201	0.33	0.795869	6	1.5E+08	0.00818	0.52	6	1.5E+08	0.0021	2.61E-08
chr6:1849	T	G	T	G	0.0268	0.016796	0.4712	0.007685	6	18492119	0.03948	0.67	6	18492350	0.0047	1.05E-08
chr6:3508	A	G	A	G	-0.0164	0.003553	0.159	0.886407	6	35082729	0.010942	0.75	6	35050506	0.0027	2.43E-09
chr7:1022	A	C	A	C	0.0144	-3.54E-05	0.3867	0.098223	7	1.02E+08	0.011259	1	7	1.02E+08	0.002	1.53E-12
chr7:1505	A	T	A	T	0.0212	0.011968	0.2157	0.791787	7	1.51E+08	0.008453	0.16	7	1.5E+08	0.0024	4.10E-18
chr8:1435	T	C	T	C	-0.0244	-0.00345	0.2058	0.45365	8	1.44E+08	0.006948	0.62	8	1.45E+08	0.0025	9.14E-22
chr8:7252	T	C	T	C	-0.0135	0.007523	0.2793	0.690202	8	72522921	0.007456	0.31	8	73435156	0.0022	1.67E-09
chr8:9315	A	G	A	G	0.0499	-0.01415	0.4732	0.899856	8	9315699	0.010596	0.18	8	9173209	0.002	1.29E-132
chr9:6845	A	C	A	C	0.0344	-0.00535	0.01789	0.322046	9	68454919	0.007302	0.46	9	71069835	0.006	1.02E-08
rs1007821	A	C	A	C	0.0128	-0.00898	0.2137	0.898175	5	1.14E+08	0.011246	0.42	5	1.13E+08	0.0023	4.60E-08
rs1009593	T	C	T	C	0.0286	-0.00943	0.4264	0.650096	8	1.16E+08	0.007013	0.18	8	1.17E+08	0.002	8.23E-45
rs1010629	A	G	A	G	0.0151	0.004149	0.3539	0.204131	8	1.03E+08	0.008502	0.63	8	1.04E+08	0.0021	1.64E-12
rs1049742	T	G	T	G	-0.0134	0.001548	0.4294	0.6256	2	1.74E+08	0.006987	0.82	2	1.75E+08	0.002	4.68E-11
rs1049824	A	C	A	C	0.0133	0.000842	0.4414	0.243516	2	2.3E+08	0.007985	0.92	2	2.31E+08	0.0021	4.96E-10
rs1056441	T	C	T	C	-0.0208	-0.02383	0.2773	0.545149	20	63738996	0.006784	0.00044	20	62370349	0.0021	2.28E-22
rs1076069	A	G	A	G	-0.0172	-0.00304	0.3847	0.668828	9	99519101	0.007211	0.67	9	1.02E+08	0.0021	8.65E-16
rs1086408	A	G	A	G	-0.016	-0.00478	0.3072	0.518732	1	2.14E+08	0.006884	0.49	1	2.14E+08	0.0021	7.25E-14
rs1087177	A	G	A	G	-0.0196	-0.00661	0.2465	0.817243	18	60184530	0.008801	0.45	18	57851763	0.0023	5.76E-17
rs1092437	T	C	T	C	-0.0147	0.011044	0.3678	0.880884	1	2.36E+08	0.010363	0.29	1	2.36E+08	0.0021	6.19E-12
rs1096933	A	C	A	C	-0.0112	-0.01228	0.4503	0.444044	9	29717281	0.006849	0.073	9	29717279	0.002	3.79E-08
rs1101273	A	G	A	G	-0.0153	0.021946	0.3519	0.963737	10	21541175	0.016705	0.19	10	21830104	0.0021	8.31E-13
rs1105440	T	C	T	C	-0.012	0.005086	0.4612	0.186359	12	11638095	0.008587	0.55	12	11791029	0.002	3.79E-09
rs1107859	T	C	T	C	0.0187	-0.00162	0.169	0.799712	17	1715069	0.008475	0.85	17	1618363	0.0026	1.62E-12
rs1111862	A	G	A	G	0.0194	-0.01003	0.2604	0.911143	1	2.21E+08	0.012035	0.4	1	2.21E+08	0.0022	4.67E-18
rs1115689	A	T	A	T	-0.0163	-0.00179	0.1819	0.217819	14	21223782	0.008389	0.83	14	21691941	0.0025	1.52E-10
rs1120868	A	T	A	T	0.0974	0.009588	0.2078	0.263208	1	65604348	0.007155	0.18	1	66070031	0.0025	1.00E-200
rs1121719	T	G	T	G	0.0134	0.00815	0.2962	0.498799	11	1.19E+08	0.006774	0.23	11	1.19E+08	0.0023	1.05E-08

rs1135855	T	G	T	G	0.0184	0.034491	0.1093	0.029779	7	44758716	0.019833	0.082	7	44798315	0.0031	1.70E-09
rs1149471	T	C	T	C	-0.0157	0.002056	0.161	0.90562	7	1.06E+08	0.011467	0.86	7	1.06E+08	0.0026	3.01E-09
rs1162441	A	G	A	G	0.0138	0.012124	0.2594	0.411623	14	75270407	0.00696	0.082	14	75737110	0.0023	3.79E-09
rs1165382	T	C	T	C	0.0199	0.007518	0.08648	0.255524	17	29325998	0.007762	0.33	17	27653016	0.0035	8.99E-09
rs1166624	A	G	A	G	-0.0419	-0.02659	0.04573	0.010087	19	37739025	0.031565	0.4	19	38229926	0.0048	2.02E-18
rs1166651	A	C	A	G	0.0298	-0.00784	0.04076	0.056436	19	10777948	0.014782	0.6	19	11019581	0.0049	1.08E-09
rs1170806	A	G	A	G	-0.0129	0.010881	0.2913	0.973823	3	1.23E+08	0.019372	0.57	3	1.23E+08	0.0023	3.61E-08
rs1177247	A	G	A	G	-0.0273	0.005924	0.1561	0.363353	7	99583473	0.00714	0.41	7	99181096	0.0029	1.01E-21
rs1178213	T	G	T	G	0.0123	0.001483	0.3419	0.254323	8	22594844	0.007846	0.85	8	22452357	0.0021	8.78E-09
rs1179045	A	G	A	G	-0.0198	-0.03716	0.159	0.944525	9	1.01E+08	0.014586	0.011	9	1.04E+08	0.0027	5.90E-13
rs1192879	A	C	A	C	-0.0194	-0.0193	0.1014	0.017531	3	33416001	0.023816	0.42	3	33457493	0.0033	2.61E-09
rs1214049	T	C	T	C	-0.0173	-0.01713	0.2227	0.018012	1	2.22E+08	0.024453	0.48	1	2.22E+08	0.0024	1.44E-12
rs1219181	T	G	T	G	-0.0119	0.001311	0.2873	0.597262	6	1.36E+08	0.006991	0.85	6	1.36E+08	0.0021	2.61E-08
rs12220	T	C	T	C	-0.0151	0.01726	0.4533	0.550913	20	58715649	0.006824	0.011	20	57290705	0.002	1.21E-13
rs1222209	A	C	A	C	-0.0146	-0.00815	0.33	0.891451	11	30340123	0.010741	0.45	11	30361670	0.0021	8.58E-12
rs1223123	A	G	A	G	-0.0279	-0.00782	0.4076	0.255764	12	95463914	0.007825	0.32	12	95857690	0.002	9.94E-43
rs1223904	T	C	T	C	-0.0404	-0.00398	0.3539	0.392411	1	2.47E+08	0.006966	0.57	1	2.48E+08	0.002	1.34E-87
rs1227014	A	G	A	G	0.0139	0.003616	0.2038	0.572046	11	69348673	0.006767	0.59	11	69163441	0.0024	1.28E-08
rs1242836	T	C	T	C	-0.0142	-0.0004	0.2296	0.644573	13	1.1E+08	0.007248	0.96	13	1.1E+08	0.0023	1.33E-09
rs1251617	T	C	T	C	-0.0151	0.016273	0.2863	0.78074	5	1.51E+08	0.008347	0.051	5	1.5E+08	0.0022	1.57E-11
rs1260326	T	C	T	C	0.0758	-0.00318	0.4105	0.461335	2	27508073	0.006816	0.64	2	27730940	0.002	1.00E-200
rs1261375	A	G	A	G	-0.0121	-0.01097	0.3877	0.520653	2	2.28E+08	0.006829	0.11	2	2.29E+08	0.0021	1.52E-08
rs1262194	C	G	C	G	0.0126	0.002094	0.2753	0.768252	2	66531780	0.008284	0.8	2	66758912	0.0022	1.85E-08
rs1271697	A	G	A	G	-0.0115	9.38E-05	0.4821	0.56172	16	29925333	0.00685	0.99	16	29936654	0.002	1.63E-08
rs1292061	A	G	A	G	-0.0209	0.003297	0.4324	0.587176	17	59833869	0.006869	0.63	17	57911230	0.002	1.03E-24
rs1292717	A	G	A	G	0.0193	-0.00246	0.3718	0.576369	16	27313700	0.006983	0.72	16	27325021	0.002	2.59E-21
rs1292950	T	C	T	C	0.0161	0.008658	0.4245	0.868396	16	69531558	0.009975	0.39	16	69565461	0.002	2.65E-15
rs1306668	A	C	A	C	-0.0128	-0.00827	0.4115	0.371518	3	94356182	0.007192	0.25	3	94075026	0.002	3.26E-10
rs1329494	T	G	T	G	0.0137	7.02E-05	0.3797	0.402498	9	15852779	0.006841	0.99	9	15852777	0.0024	2.06E-08
rs1332328	T	C	T	C	0.0265	0.009636	0.3598	0.372238	10	89251701	0.006999	0.17	10	91011458	0.0021	2.81E-35
rs1337267	CT	C	G	A	0.0146	-0.013	0.3002	0.376081	13	85790261	0.006975	0.062	13	86367973	0.0025	9.70E-09
rs1338071	C	G	C	G	0.0163	-0.0081	0.2932	0.610231	6	1.53E+08	0.006991	0.25	6	1.53E+08	0.0022	3.41E-13
rs1419361	A	G	A	G	0.0127	0.002224	0.339	0.652257	4	1.02E+08	0.007297	0.76	4	1.03E+08	0.0021	2.85E-09
rs1464245	A	G	A	G	0.0159	0.004837	0.161	0.356628	14	35152187	0.007026	0.49	14	35621393	0.0027	7.30E-09
rs1470560	A	G	A	G	0.0121	0.006225	0.338	0.170029	3	35628658	0.009014	0.49	3	35670150	0.0021	1.52E-08
rs1635852	T	C	T	C	0.0141	0.010976	0.4811	0.773055	7	28149792	0.008127	0.18	7	28189411	0.002	4.38E-12
rs1705027	A	G	A	G	0.0112	0.004958	0.4284	0.456052	2	1.21E+08	0.006764	0.46	2	1.21E+08	0.002	3.79E-08
rs1705627	C	G	C	G	-0.0228	-0.02616	0.2724	0.929875	5	1.59E+08	0.013211	0.048	5	1.58E+08	0.0039	3.79E-09
rs1709882	C	G	C	G	-0.0129	0.008568	0.171	0.532901	12	63061567	0.006579	0.19	12	63455347	0.0023	3.61E-08
rs1713847	A	C	A	C	0.0338	0.003851	0.1133	0.278338	17	37713312	0.007645	0.61	17	36073320	0.0032	9.23E-27
rs1713852	T	C	T	C	0.0124	-0.00218	0.3101	0.438521	7	1.16E+08	0.006869	0.75	7	1.16E+08	0.0022	3.10E-08
rs1741725	A	G	A	G	-0.015	-0.00258	0.2823	0.59342	1	2.05E+08	0.006889	0.71	1	2.05E+08	0.0022	2.13E-11
rs174373	T	C	T	C	0.0135	-0.00274	0.2107	0.429635	6	1.12E+08	0.006912	0.69	6	1.12E+08	0.0024	3.30E-08

rs1761606	A	G	A	G	0.1338	-0.02146	0.07853	0.989193	16	51402971	0.033341	0.52	16	51436882	0.0044	1.00E-200
rs1800693	T	C	T	C	0.0218	0.010008	0.4254	0.861671	12	6330843	0.009817	0.31	12	6440009	0.002	9.56E-27
rs1800961	T	C	T	C	-0.1074	-0.00754	0.03678	0.01489	20	44413724	0.027641	0.78	20	43042364	0.0059	6.50E-74
rs1880241	A	G	A	G	0.0252	0.009847	0.4811	0.035062	7	22719850	0.016705	0.56	7	22759469	0.002	3.54E-35
rs1886008	T	C	T	C	0.0184	0.000465	0.1402	0.57805	20	62022567	0.006948	0.95	20	60597623	0.0033	1.63E-08
rs1969066	A	G	A	G	0.0133	0.004653	0.3082	0.456052	3	1.57E+08	0.006966	0.5	3	1.57E+08	0.0022	2.89E-09
rs197419	A	C	A	C	-0.0123	0.009997	0.4185	0.676273	1	1.12E+08	0.007276	0.17	1	1.12E+08	0.002	1.54E-09
rs2156407	A	G	A	G	0.0135	0.014785	0.2813	0.425312	21	36075413	0.006866	0.031	21	37447711	0.0022	1.67E-09
rs2161037	A	G	A	G	0.0205	-0.00874	0.4672	0.610471	2	1.69E+08	0.007136	0.22	2	1.7E+08	0.002	7.70E-24
rs2161374	T	C	T	C	-0.0191	-0.00323	0.4781	0.336215	5	1.73E+08	0.007278	0.66	5	1.72E+08	0.002	6.60E-21
rs2239222	A	G	A	G	-0.0359	-0.00253	0.3539	0.589337	14	72545177	0.006968	0.72	14	73011885	0.0021	2.85E-63
rs2261790	T	C	T	C	-0.0178	-0.00037	0.4334	0.120797	20	25282153	0.009612	0.97	20	25262789	0.002	2.30E-18
rs2274782	T	C	T	C	0.012	-0.00111	0.3002	0.302113	9	1.24E+08	0.007455	0.88	9	1.27E+08	0.0021	1.99E-08
rs2280406	A	G	A	G	0.0222	0.00774	0.4911	0.154179	3	49904003	0.009527	0.42	3	49941436	0.002	1.12E-27
rs2289852	A	G	A	G	-0.0313	0.00342	0.06262	0.275696	5	1.57E+08	0.007625	0.65	5	1.57E+08	0.0044	8.72E-13
rs2307377	A	G	A	G	-0.0221	0.006071	0.08847	0.89049	6	87657917	0.010755	0.57	6	88367635	0.0038	4.45E-09
rs2429473	A	C	A	C	0.0224	0.013715	0.1998	0.552354	12	46805116	0.006906	0.047	12	47198899	0.0025	1.36E-18
rs245767	A	G	A	G	-0.0145	0.008751	0.2734	0.123199	5	1.71E+08	0.010317	0.4	5	1.71E+08	0.0022	9.58E-11
rs2596937	T	C	T	C	-0.0173	0.000511	0.1769	0.873679	3	9439222	0.010422	0.96	3	9480906	0.0024	1.44E-12
rs2657896	T	C	T	C	-0.0233	0.00655	0.1789	0.157781	12	56535910	0.00948	0.49	12	56929694	0.0027	2.33E-17
rs2700938	T	C	T	C	-0.0236	-0.00937	0.3817	0.775696	7	36045532	0.00829	0.26	7	36085142	0.0021	2.51E-28
rs2858259	A	G	A	G	0.0157	-0.0043	0.2137	0.068924	1	97960920	0.013531	0.75	1	98426476	0.0026	3.01E-09
rs2860176	C	G	C	G	0.0211	0.010458	0.4076	0.847502	8	1.25E+08	0.009505	0.27	8	1.27E+08	0.002	3.69E-25
rs288183	T	G	T	G	0.0148	0.002447	0.167	0.65682	5	1.08E+08	0.007121	0.73	5	1.07E+08	0.0026	2.26E-08
rs2926851	T	C	T	C	0.0122	0.008484	0.4463	0.080452	5	1.54E+08	0.012393	0.49	5	1.53E+08	0.002	2.08E-09
rs303429	T	C	T	C	-0.0152	-0.01589	0.4026	0.350865	10	30419512	0.007202	0.027	10	30708441	0.002	8.35E-14
rs339969	A	C	A	C	0.0339	0.003982	0.3917	0.85975	15	60591082	0.009807	0.68	15	60883281	0.0021	1.29E-56
rs3476152	T	C	T	C	-0.0179	-0.00248	0.1909	0.355427	1	22354721	0.007127	0.73	1	22681214	0.0025	2.03E-12
rs3486435	A	G	A	G	-0.0301	0.00059	0.05865	0.989914	14	58292562	0.033685	0.99	14	58759280	0.0046	5.04E-11
rs3732356	T	G	T	G	-0.0235	-0.0109	0.05765	0.884486	3	1.2E+08	0.010637	0.31	3	1.2E+08	0.0042	1.81E-08
rs3768321	T	G	T	G	0.0338	-0.00335	0.1879	0.105187	1	39570256	0.011215	0.77	1	40035928	0.0025	3.06E-40
rs3808348	T	C	T	C	-0.0224	0.005494	0.2147	0.275937	7	988812	0.007647	0.47	7	1028448	0.0025	1.36E-18
rs3935032	T	C	T	C	-0.0153	0.005762	0.3579	0.089337	1	1628814	0.011835	0.63	1	1564194	0.0023	6.42E-11
rs424539	C	G	C	G	-0.012	-0.00736	0.337	0.53194	9	14442597	0.006715	0.27	9	14442595	0.0021	1.99E-08
rs4246598	A	C	A	C	0.0134	0.006568	0.3897	0.2195	2	88138531	0.008276	0.43	2	88438050	0.002	4.68E-11
rs4354188	T	C	T	C	0.018	-0.00784	0.2346	0.927233	6	1.16E+08	0.01282	0.54	6	1.16E+08	0.002	9.60E-19
rs4418728	T	G	T	G	-0.0166	0.000331	0.4871	0.817483	10	93079967	0.008723	0.97	10	94839724	0.002	3.58E-16
rs4468667	A	G	A	G	-0.0124	-0.00654	0.3032	0.21902	17	67907513	0.007991	0.41	17	65903629	0.0022	3.10E-08
rs4656241	T	C	T	C	-0.0884	0.022247	0.2416	0.975745	1	1.6E+08	0.021292	0.3	1	1.6E+08	0.0025	1.00E-200
rs4658403	T	C	T	C	-0.0184	0.006757	0.1779	0.737752	1	2.44E+08	0.007604	0.37	1	2.44E+08	0.0026	3.63E-12
rs4690098	T	C	T	C	0.0167	-0.00169	0.2346	0.427714	4	3445429	0.006864	0.81	4	3447156	0.0024	8.24E-12
rs4704552	T	G	C	T	0.0184	0.012717	0.338	0.420989	5	79284545	0.006961	0.068	5	78570219	0.0021	7.58E-18
rs4714508	A	G	A	G	-0.0179	0.009143	0.3449	0.600624	6	41703939	0.00689	0.18	6	41671677	0.0021	5.67E-17

rs4755720	T	C	T	C	-0.0165	-0.01105	0.3996	0.740634	11	43607199	0.007645	0.15	11	43628749	0.002	5.36E-16
rs4764939	T	C	T	C	-0.0201	-0.00307	0.494	0.801393	12	1.03E+08	0.008575	0.72	12	1.04E+08	0.002	5.56E-23
rs4788095	T	C	T	C	-0.0166	0.018461	0.331	0.909462	16	28820038	0.011876	0.12	16	28831359	0.002	3.58E-16
rs516316	C	G	C	G	0.0223	0.003176	0.4414	0.038665	19	48702888	0.017155	0.85	19	49206145	0.002	6.54E-28
rs5585523	T	C	T	C	-0.027	0.000944	0.332	0.722863	18	57422483	0.007561	0.9	18	55089715	0.0021	1.48E-36
rs5854292	T	C	T	C	0.0356	-0.00303	0.06759	0.069885	19	19268740	0.013267	0.82	19	19379549	0.0039	3.53E-20
rs5942708	T	C	T	C	-0.0125	0.000623	0.4642	0.336936	18	26218893	0.007114	0.93	18	23798857	0.002	8.32E-10
rs5973743	T	C	T	C	-0.015	0.005641	0.2386	0.511768	19	2671102	0.007027	0.42	19	2671100	0.0023	1.50E-10
rs5991640	T	G	T	G	0.0166	-0.0167	0.336	0.657541	2	2.41E+08	0.007105	0.019	2	2.42E+08	0.0021	8.23E-15
rs6003710	A	T	A	T	0.0183	0.00725	0.3032	0.466378	16	88462228	0.006797	0.29	16	88528636	0.0022	3.08E-16
rs6020459	T	C	T	C	-0.0178	-0.00708	0.326	0.334774	20	50344009	0.007194	0.33	20	48960546	0.0022	1.91E-15
rs6073958	T	C	T	C	0.0279	0.011717	0.2207	0.865754	20	45923216	0.010095	0.25	20	44551855	0.0025	5.87E-28
rs6201128	A	G	A	G	-0.0156	0.00647	0.3926	0.027137	15	63498926	0.019077	0.73	15	63791125	0.0021	2.96E-13
rs6443429	A	C	A	C	0.0134	0.009475	0.3022	0.259126	3	1.77E+08	0.007739	0.22	3	1.77E+08	0.0021	3.67E-10
rs6486122	T	C	T	C	0.0296	0.007754	0.2962	0.397935	11	13339977	0.006992	0.27	11	13361524	0.0021	1.38E-43
rs6500446	A	G	A	G	0.0114	0.011368	0.4175	0.052113	16	89782151	0.013928	0.41	16	89848559	0.002	2.16E-08
rs6519133	T	C	T	C	0.0313	-0.00399	0.4046	0.539625	22	38700597	0.006898	0.56	22	39096602	0.0021	1.58E-48
rs6595549	C	G	C	G	-0.0155	-0.00631	0.1769	0.32757	5	1.25E+08	0.007132	0.38	5	1.24E+08	0.0026	4.76E-09
rs663015	T	C	T	C	-0.0167	0.001665	0.2823	0.690922	11	72787990	0.007436	0.82	11	72499035	0.0021	5.69E-15
rs6734238	A	G	A	G	-0.0449	0.008379	0.4125	0.896013	2	1.13E+08	0.010904	0.44	2	1.14E+08	0.002	9.53E-108
rs6775319	A	T	A	T	-0.0131	0.002133	0.2644	0.517051	3	18717009	0.006916	0.76	3	18758501	0.0022	4.96E-09
rs6792725	A	G	A	G	0.0185	0.006767	0.329	0.48439	3	24478792	0.006783	0.32	3	24520283	0.0022	1.47E-16
rs6801781	A	G	A	G	0.0129	0.008013	0.2783	0.910903	3	71583115	0.01142	0.48	3	71632266	0.0022	8.45E-09
rs6808581	A	G	A	G	-0.0164	-0.01443	0.2575	0.030019	17	49058513	0.019468	0.46	17	47135875	0.0022	2.45E-13
rs687339	T	C	T	C	-0.0249	-0.00356	0.2296	0.865514	3	1.36E+08	0.00976	0.71	3	1.36E+08	0.0024	2.19E-24
rs6925389	A	G	A	G	-0.0149	0.014837	0.3777	0.958453	6	7112586	0.016226	0.36	6	7112819	0.0021	3.20E-12
rs704017	A	G	A	G	0.016	0.001389	0.4384	0.708694	10	79059375	0.007416	0.85	10	80819132	0.002	3.92E-15
rs7084062	A	G	A	G	-0.0133	0.003617	0.3439	0.42171	10	1.32E+08	0.006909	0.6	10	1.34E+08	0.0021	4.96E-10
rs7103411	T	C	T	C	0.0216	0.004957	0.4085	0.512248	11	27678578	0.006907	0.47	11	27700125	0.0024	9.60E-19
rs7127808	A	T	A	T	-0.0206	0.009406	0.1909	0.674832	11	66164448	0.007328	0.2	11	65931919	0.0026	7.14E-15
rs7269439	T	G	T	G	-0.0179	0.012757	0.4891	0.091018	14	24404987	0.011629	0.27	14	24874193	0.002	1.49E-18
rs7271255	A	G	A	G	-0.012	0.003176	0.4115	0.268732	4	1.4E+08	0.007692	0.68	4	1.41E+08	0.0021	1.99E-08
rs7279949	A	G	A	G	0.0166	0.018678	0.1451	0.130884	5	1.33E+08	0.010248	0.068	5	1.32E+08	0.0027	1.55E-09
rs7313714	A	G	A	G	0.0186	-0.00787	0.168	0.957733	7	74659260	0.016941	0.64	7	74073590	0.0026	2.12E-12
rs7357754	A	G	A	G	-0.0177	0.003421	0.4841	0.73367	9	89592393	0.007829	0.66	9	92207308	0.002	3.55E-18
rs7367251	T	G	T	G	-0.0175	0.002933	0.1412	0.101345	9	1.3E+08	0.011473	0.8	9	1.33E+08	0.0031	1.01E-08
rs7455288	T	C	T	C	0.0143	0.006064	0.2256	0.767291	7	6376984	0.00798	0.45	7	6416615	0.0023	1.02E-09
rs7490497	A	C	A	C	-0.019	-0.00534	0.09443	0.305956	4	88128874	0.007441	0.47	4	89050026	0.0033	5.49E-09
rs7522990	T	C	T	C	0.0372	-0.00421	0.1143	0.902257	7	73481616	0.011306	0.71	7	72895946	0.0031	4.02E-34
rs7526511	C	G	C	G	-0.0182	-0.02305	0.1551	0.989193	2	1.65E+08	0.031936	0.47	2	1.66E+08	0.0031	2.54E-09
rs7539332	T	C	G	C	0.0333	-0.00921	0.1541	0.034342	11	47244920	0.018117	0.61	11	47245389	0.0027	8.94E-34
rs7639927	A	T	A	T	-0.0134	-0.02059	0.4771	0.209174	3	4721050	0.008411	0.014	3	4762734	0.002	4.68E-11
rs7662058	A	G	A	G	0.019	0.00301	0.08946	0.931556	13	1.03E+08	0.013672	0.83	13	1.03E+08	0.0035	4.05E-08

rs7662792	A	T	A	T	-0.0166	-5.41E-05	0.3191	0.459894	4	45119856	0.006852	0.99	4	45121873	0.0021	8.23E-15
rs7684939	A	AC	T	C	-0.0134	0.008148	0.05865	0.322526	4	54643022	0.007192	0.26	4	55508495	0.0023	1.05E-08
rs7770473	T	C	T	C	0.0483	0.003965	0.02883	0.992555	5	52785075	0.04011	0.92	5	52080909	0.0052	1.38E-20
rs7773862	T	C	T	C	-0.0588	-0.02225	0.01093	0.012008	12	1.01E+08	0.031682	0.48	12	1.01E+08	0.0099	2.62E-09
rs7801838	T	C	T	C	-0.0128	0.014949	0.3101	0.100144	7	1.29E+08	0.011228	0.18	7	1.29E+08	0.0022	1.10E-08
rs7842899	T	C	T	C	-0.0203	-0.00219	0.3936	0.686359	9	1.36E+08	0.007415	0.77	9	1.39E+08	0.002	2.08E-23
rs787488	A	G	A	G	0.0136	-0.00666	0.2873	0.901777	1	67622756	0.011144	0.55	1	68088439	0.0022	1.27E-09
rs7908825	C	G	C	G	-0.0133	0.003833	0.3678	0.884726	10	73794783	0.010467	0.71	10	75554541	0.002	6.51E-11
rs7933202	A	C	A	C	0.021	0.002837	0.4652	0.901057	11	60169453	0.010585	0.79	11	59936926	0.002	6.16E-25
rs7956514	T	G	T	G	-0.0151	0.002326	0.2545	0.560038	12	12726320	0.006873	0.74	12	12879254	0.0022	1.57E-11
rs7993752	A	C	A	C	-0.0134	0.000535	0.4791	0.487272	13	41999844	0.007046	0.94	13	42573980	0.002	4.68E-11
rs8072566	A	G	A	G	-0.0204	0.001814	0.2058	0.35951	17	42324345	0.00714	0.8	17	40476363	0.0025	1.11E-15
rs878664	A	C	A	G	-0.014	-0.07394	0.2753	0.005283	18	62447188	0.047428	0.12	18	60203855	0.0022	4.10E-10
rs9200655	T	C	T	C	-0.0239	-0.00163	0.3678	0.630403	9	1.33E+08	0.007032	0.82	9	1.36E+08	0.0021	5.21E-29
rs930035	A	G	A	G	-0.0133	-0.00998	0.4722	0.675793	2	1.71E+08	0.007245	0.17	2	1.72E+08	0.002	6.51E-11
rs9604045	T	G	T	G	-0.0229	0.018881	0.2565	0.199087	13	1.13E+08	0.008795	0.032	13	1.14E+08	0.0027	8.06E-17
rs9634098	C	G	T	C	-0.0522	0.047421	0.04573	0.009846	12	24053144	0.034925	0.17	12	24195042	0.0054	3.90E-22
rs9738365	A	C	A	C	0.0212	0.016801	0.3072	0.175072	12	31844701	0.00904	0.063	12	31997635	0.0023	1.39E-19
rs9788721	T	C	T	C	-0.0121	-0.00127	0.3598	0.696686	15	78510527	0.007407	0.86	15	78802869	0.0021	1.52E-08
rs991946	T	C	T	C	0.0112	0.00134	0.4125	0.310038	6	1.66E+08	0.007419	0.86	6	1.66E+08	0.002	3.79E-08
rs994596	T	C	T	C	0.0137	-0.00823	0.4344	0.301153	4	18458205	0.007509	0.27	4	18459828	0.0021	1.48E-10
rs9965184	A	T	A	T	0.0118	0.006552	0.329	0.570605	18	43196641	0.006836	0.34	18	40776606	0.0021	3.41E-08

Supplementary Table 2l. List of SNPs for Total White Cell Count and their effects on TEWL

SNP	effect_alle	other_allel	effect_alle	other_allel	beta.TWC	beta.TEWL	eaf.TWC	eaf.TEWL	chr.TEWL	pos.TEWL	se.TEWL	pval.TEWL	chr.TWC	pos.TWC	se.TWC	pval.TWC
chr1:1012(	G	C	T	C	-0.14688	0.026492	0.003596	0.010807	1	1.01E+08	0.033362	0.43	1	1.01E+08	0.014471	3.56E-24
chr1:1459(	C	T	C	T	0.003395	-0.00986	0.780415	0.67195	1	1.46E+08	0.007304	0.18	1	1.46E+08	0.002193	0.121638
chr1:1696:	A	C	A	C	0.000917	-0.00451	0.219085	0.012728	1	1.7E+08	0.030213	0.88	1	1.7E+08	0.002098	0.662104
chr1:1740:	A	G	A	G	-0.01554	-0.01363	0.247801	0.567723	1	1.74E+08	0.006706	0.042	1	1.74E+08	0.001959	2.25E-15
chr1:1821;	G	C	G	C	0.014336	0.000686	0.310047	0.24976	1	1.82E+08	0.007859	0.93	1	1.82E+08	0.001742	1.95E-16
chr1:1985;	A	C	A	C	-0.03471	0.006662	0.094648	0.130644	1	1.99E+08	0.010164	0.51	1	1.99E+08	0.002814	6.63E-35
chr1:2036{	G	C	G	C	-0.02552	0.017649	0.89393	0.982229	1	2.04E+08	0.025177	0.48	1	2.04E+08	0.003063	8.39E-17
chr1:2790{	T	C	T	C	-0.01557	-0.00353	0.388104	0.512248	1	27908488	0.006951	0.61	1	27908488	0.00165	4.14E-21
chr1:3992(	T	C	T	C	0.013596	0.007953	0.259492	0.035303	1	39926080	0.01793	0.66	1	39926080	0.002044	3.02E-11
chr1:4585{	G	T	G	T	-0.0154	-0.00703	0.476458	0.608309	1	45858493	0.006951	0.31	1	45858493	0.001687	7.39E-20
chr1:9358!	G	T	G	C	0.015559	0.026688	0.202849	0.005764	1	93585354	0.045455	0.56	1	93585354	0.002226	2.88E-12
chr10:135(	C	A	C	A	0.013457	0.011663	0.513009	0.324928	10	13502285	0.007289	0.11	10	13502285	0.001651	3.79E-16
chr10:678;	G	A	G	A	0.014029	0.014619	0.56575	0.180596	10	67874444	0.008718	0.094	10	67874444	0.001771	2.49E-15
chr10:747:	T	C	T	C	-0.01474	-0.00143	0.769722	0.826369	10	74730578	0.008964	0.87	10	74730578	0.001917	1.51E-14
chr10:777!	T	C	T	C	0.011211	-0.00562	0.385489	0.43732	10	77792334	0.006982	0.42	10	77792334	0.001667	1.80E-11
chr10:942;	C	G	C	G	-0.01874	0.001952	0.442832	0.540106	10	94279840	0.00688	0.78	10	94279840	0.001618	5.67E-31
chr11:114:	A	T	A	T	0.027012	-0.01237	0.296945	0.291787	11	1.14E+08	0.007565	0.1	11	1.14E+08	0.001772	2.03E-52
chr11:116{	T	C	T	C	0.016408	-0.00582	0.766848	0.407061	11	1.17E+08	0.006935	0.4	11	1.17E+08	0.002196	8.28E-14
chr11:128‹	A	G	A	G	-0.02531	-0.00836	0.289159	0.184678	11	1.28E+08	0.00888	0.35	11	1.28E+08	0.001804	1.07E-44
chr11:308:	C	T	C	T	0.043503	-0.01147	0.521522	0.925552	11	308314	0.012474	0.36	11	308314	0.002047	4.03E-100
chr11:618(	G	C	G	C	-0.0196	-0.00254	0.327978	0.571326	11	61803876	0.006565	0.7	11	61803876	0.00173	1.02E-29
chr11:696{	T	G	T	G	-0.01592	0.01057	0.67551	0.836455	11	69685018	0.009263	0.25	11	69685018	0.001803	1.13E-18
chr11:880:	T	C	T	C	-0.01719	-0.00495	0.660818	0.370077	11	8801946	0.007059	0.48	11	8801946	0.001773	3.36E-22
chr12:111‹	C	T	C	T	-0.06635	0.008637	0.624937	0.987032	12	1.11E+08	0.028479	0.76	12	1.11E+08	0.001815	1.00E-200
chr12:121{	T	A	T	A	-0.0182	-0.00386	0.833149	0.954371	12	1.22E+08	0.016207	0.81	12	1.22E+08	0.002211	1.98E-16
chr12:127;	C	T	C	T	0.019285	-0.00035	0.30502	0.429875	12	12725831	0.006898	0.96	12	12725831	0.001768	1.17E-27
chr12:204;	C	G	C	G	-0.01619	0.006074	0.168602	0.076129	12	20426458	0.012942	0.64	12	20426458	0.002183	1.24E-13
chr12:478(	C	T	C	T	-0.01284	0.012101	0.608851	0.454371	12	47808263	0.006805	0.075	12	47808263	0.001761	3.20E-13
chr12:501{	G	T	G	T	0.01371	0.007914	0.416027	0.558598	12	50184922	0.006865	0.25	12	50184922	0.001687	4.64E-16
chr12:519:	T	C	T	C	0.022569	-0.00871	0.786583	0.852065	12	51912002	0.009598	0.36	12	51912002	0.00201	3.26E-29
chr12:572!	T	C	T	C	-0.01457	-0.01414	0.201016	0.082133	12	57298687	0.012459	0.26	12	57298687	0.002027	6.89E-13
chr12:681‹	A	G	A	G	0.011624	-4.35E-05	0.668577	0.742555	12	68149460	0.007612	1	12	68149460	0.001741	2.52E-11
chr13:113!	A	T	A	T	0.020044	0.009002	0.299899	0.388569	13	1.14E+08	0.007057	0.2	13	1.14E+08	0.001761	5.72E-30
chr13:517;	C	G	C	G	-0.01605	-0.0015	0.173799	0.151537	13	51771501	0.009385	0.87	13	51771501	0.002151	8.93E-14
chr14:103:	C	G	C	G	0.017372	-0.00229	0.623231	0.400096	14	1.03E+08	0.006959	0.74	14	1.03E+08	0.001776	1.48E-22
chr14:812(	C	G	C	G	-0.01296	-0.01233	0.543932	0.402978	14	81202363	0.006995	0.078	14	81202363	0.001615	1.06E-15
chr14:926:	C	G	C	G	-0.03061	-0.00653	0.176954	0.016811	14	92631994	0.026517	0.81	14	92631994	0.002358	1.76E-38
chr15:419:	T	C	T	C	-0.0402	-0.00179	0.115502	0.085735	15	41936843	0.011983	0.88	15	41936843	0.00255	6.22E-56
chr15:770:	C	T	C	T	-0.01386	0.004199	0.284938	0.194765	15	77013944	0.008493	0.62	15	77013944	0.001808	1.90E-14
chr16:304;	C	G	C	G	0.037905	0.025498	0.605975	0.897695	16	30474072	0.011365	0.025	16	30474072	0.001807	1.10E-97
chr16:887!	G	C	G	C	-0.0169	-0.00293	0.592978	0.636647	16	88795935	0.007203	0.68	16	88795935	0.001767	1.20E-21

chr17:162	C	G	C	G	0.024816	-0.01469	0.623539	0.913064	17	16237935	0.012024	0.22	17	16237935	0.001839	1.81E-41
chr17:297	C	T	C	T	-0.0212	0.009993	0.489511	0.311239	17	29738261	0.007415	0.18	17	29738261	0.001717	5.90E-35
chr17:399	G	T	G	T	0.071473	-0.00918	0.478787	0.54707	17	39968377	0.006957	0.19	17	39968377	0.001713	1.00E-200
chr17:481	T	A	T	A	0.016084	0.003442	0.239827	0.299232	17	48131431	0.007384	0.64	17	48131431	0.001895	2.26E-17
chr17:542	A	G	A	G	0.020133	0.017438	0.593963	0.955572	17	5422842	0.016745	0.3	17	5422842	0.001805	7.63E-29
chr17:733	T	C	T	C	-0.02527	0.003637	0.197914	0.07805	17	7337072	0.012945	0.78	17	7337072	0.002185	6.49E-31
chr17:746	T	C	T	C	0.021501	-0.01024	0.72912	0.553554	17	74699072	0.006903	0.14	17	74699072	0.001924	5.86E-29
chr17:764	C	G	C	G	-0.01499	0.007812	0.400289	0.262968	17	76402433	0.007732	0.31	17	76402433	0.001716	2.51E-18
chr18:488	A	G	C	G	0.021461	-0.02819	0.112308	0.010327	18	48806551	0.033345	0.4	18	48806551	0.002866	7.27E-14
chr19:102	G	T	G	T	-0.02504	0.003856	0.665226	0.420269	19	10280935	0.006766	0.57	19	10280935	0.001873	1.04E-40
chr19:138	G	T	G	T	0.019702	0.01747	0.350708	0.282901	19	13837583	0.007558	0.021	19	13837583	0.001733	6.34E-30
chr19:143	C	G	C	G	0.065963	-0.00608	0.018477	0.013209	19	14388802	0.03024	0.84	19	14388802	0.007483	1.26E-18
chr19:163	C	A	C	A	-0.039	-0.00235	0.762745	0.613593	19	16384963	0.007045	0.74	19	16384963	0.001946	2.55E-89
chr19:394	A	G	A	G	-0.01191	-0.00526	0.49863	0.527618	19	39456187	0.006928	0.45	19	39456187	0.00161	1.43E-13
chr19:512	T	C	T	C	-0.02471	0.000255	0.290492	0.182757	19	51225221	0.008829	0.98	19	51225221	0.001782	1.04E-43
chr2:1271	A	G	A	G	0.027625	0.023837	0.064416	0.057397	2	1.27E+08	0.014546	0.1	2	1.27E+08	0.00336	2.11E-16
chr2:1598	G	A	G	A	-0.03132	-0.00542	0.845951	0.896494	2	1.6E+08	0.011217	0.63	2	1.6E+08	0.002234	1.31E-44
chr2:1814	T	G	T	G	0.033947	-0.00278	0.517669	0.386647	2	1.81E+08	0.007013	0.69	2	1.81E+08	0.001629	2.06E-96
chr2:2317	A	G	A	G	-0.02104	-0.00318	0.211117	0.191162	2	2.32E+08	0.008589	0.71	2	2.32E+08	0.002047	9.32E-25
chr2:8531	T	C	A	C	-0.01427	-0.01026	0.584702	0.120797	2	85313657	0.010347	0.32	2	85313657	0.001839	8.96E-15
chr20:406	C	G	C	G	0.02227	0.006195	0.594701	0.432277	20	40630638	0.006879	0.37	20	40630638	0.001664	8.24E-41
chr20:637	G	A	G	A	0.01205	0.025577	0.612218	0.433477	20	63706054	0.006812	0.00017	20	63706054	0.001728	3.22E-12
chr22:199	T	G	T	G	0.017901	-0.00408	0.327066	0.264409	22	19983024	0.007675	0.59	22	19983024	0.001782	1.03E-23
chr22:391	C	G	C	G	-0.01932	-0.01522	0.3895	0.231988	22	39136415	0.008218	0.064	22	39136415	0.0017	7.16E-30
chr3:1369	T	A	T	A	0.014428	0.001892	0.659532	0.841739	3	1.37E+08	0.00937	0.84	3	1.37E+08	0.001735	9.65E-17
chr3:1512	A	C	A	C	0.018836	-0.01208	0.832934	0.845581	3	1.51E+08	0.009469	0.2	3	1.51E+08	0.002162	3.16E-18
chr3:1840	C	T	C	T	0.014659	-0.00449	0.622358	0.82757	3	1.84E+08	0.008902	0.61	3	1.84E+08	0.001729	2.39E-17
chr3:1872	G	A	G	A	0.016437	-0.02054	0.174075	0.129683	3	18722783	0.010279	0.046	3	18722783	0.002144	1.86E-14
chr3:7144	T	C	T	C	0.014255	-0.02444	0.593955	0.090058	3	71449831	0.011507	0.034	3	71449831	0.002016	1.59E-12
chr4:3103	A	G	A	G	-0.01402	0.003668	0.357323	0.410183	4	3103473	0.006975	0.6	4	3103473	0.001685	9.11E-17
chr4:5454	T	A	T	A	0.025668	0.005499	0.269486	0.350144	4	54542708	0.00726	0.45	4	54542708	0.001856	1.81E-43
chr4:6968	G	A	G	A	-0.01767	0.004655	0.375444	0.270653	4	6968192	0.007727	0.55	4	6968192	0.001667	3.14E-26
chr4:7175	T	G	T	G	-0.02655	0.00314	0.281416	0.260086	4	71752606	0.007673	0.68	4	71752606	0.001787	6.59E-50
chr4:8892	C	T	C	T	0.012076	0.00467	0.742801	0.541306	4	88927432	0.006902	0.5	4	88927432	0.001872	1.16E-10
chr5:1154	G	T	G	T	-0.01755	-0.00321	0.876036	0.835735	5	1.15E+08	0.009086	0.72	5	1.15E+08	0.002448	8.01E-13
chr5:1227	T	C	T	C	0.011133	-0.0086	0.566955	0.631604	5	1.23E+08	0.006971	0.22	5	1.23E+08	0.001702	6.32E-11
chr5:1285	A	C	A	C	0.02938	-6.94E-06	0.326633	0.396494	5	1285859	0.006994	1	5	1285859	0.001862	4.84E-56
chr5:5801	A	G	A	G	-0.01802	0.009654	0.174992	0.105908	5	58019808	0.01113	0.39	5	58019808	0.002136	3.52E-17
chr6:1224	G	A	G	A	0.018824	-0.00164	0.546455	0.458934	6	1.22E+08	0.006829	0.81	6	1.22E+08	0.001612	1.78E-31
chr6:1367	T	C	T	C	0.015465	0.003077	0.425862	0.504563	6	1.37E+08	0.006801	0.65	6	1.37E+08	0.001639	4.25E-21
chr6:3667	C	A	C	A	0.016089	0.000153	0.432065	0.349183	6	36670914	0.007075	0.98	6	36670914	0.001677	8.86E-22
chr7:1050	C	G	C	G	0.014501	0.001057	0.231881	0.502882	7	1.05E+08	0.006761	0.88	7	1.05E+08	0.00222	6.73E-11
chr7:1300	T	C	T	C	0.016954	-0.00906	0.301138	0.066042	7	1.3E+08	0.013509	0.5	7	1.3E+08	0.001862	9.34E-20

chr7:1397{	G	A	G	A	-0.01198	-0.00665	0.707979	0.579971	7	13979617	0.006911	0.34	7	13979617	0.00178	1.80E-11
chr7:1491{	T	C	T	C	-0.01435	7.85E-05	0.702687	0.887608	7	1.49E+08	0.010766	0.99	7	1.49E+08	0.001823	3.71E-15
chr7:1816{	C	T	C	T	-0.01351	-0.01204	0.221979	0.253362	7	18164395	0.007766	0.12	7	18164395	0.001936	3.05E-12
chr7:2354{	T	C	T	C	0.014103	-0.01199	0.213816	0.036023	7	23547541	0.016646	0.47	7	23547541	0.002019	2.95E-12
chr7:2716{	A	C	A	C	-0.02322	-0.0053	0.082454	0.164505	7	27165113	0.009207	0.56	7	27165113	0.002996	9.64E-15
chr7:4807{	C	T	C	T	-0.01052	-0.00153	0.433122	0.486551	7	48077226	0.006847	0.82	7	48077226	0.001622	9.34E-11
chr7:9954(	A	C	A	C	-0.02181	-0.01314	0.080172	0.009366	7	99540251	0.034888	0.71	7	99540251	0.00303	6.43E-13
chr8:1265(	T	C	T	C	0.01867	-0.00677	0.12922	0.288665	8	1.27E+08	0.007485	0.37	8	1.27E+08	0.002501	8.62E-14
chr8:1280(	T	C	T	C	0.017845	-0.00387	0.505807	0.565562	8	1.28E+08	0.006832	0.57	8	1.28E+08	0.001654	4.31E-27
chr8:1296(	T	C	T	C	-0.03731	0.00104	0.578197	0.371037	8	1.3E+08	0.007061	0.88	8	1.3E+08	0.001712	2.92E-105
chr9:1111{	G	A	G	A	-0.02465	0.003413	0.412105	0.165466	9	1.11E+08	0.009352	0.72	9	1.11E+08	0.001698	1.02E-47
chr9:1209{	A	G	A	G	0.011151	-0.00684	0.538442	0.520173	9	1.21E+08	0.00681	0.32	9	1.21E+08	0.00163	8.20E-12
chr9:4039{	T	G	T	G	-0.01307	0.013542	0.645549	0.79659	9	4039727	0.008512	0.11	9	4039727	0.001709	2.15E-14
chr9:8892(	C	T	C	T	-0.01741	0.011075	0.572279	0.650576	9	88920798	0.007265	0.13	9	88920798	0.001637	2.14E-26
chr9:9273{	A	C	A	C	0.017827	-0.00045	0.259563	0.70389	9	92732165	0.007173	0.95	9	92732165	0.001988	3.18E-19
rs1005006	C	A	C	A	-0.01274	0.012235	0.413689	0.285783	4	80000216	0.007622	0.11	4	80000216	0.001665	2.02E-14
rs1005807	A	G	A	G	-0.03311	-0.04512	0.362436	0.011287	5	1.32E+08	0.031335	0.15	5	1.32E+08	0.001821	7.54E-74
rs1012116	A	G	A	G	-0.01382	0.00458	0.7005	0.674592	9	1.13E+08	0.007295	0.53	9	1.13E+08	0.00178	8.66E-15
rs1012551	T	C	T	C	-0.01204	-0.00609	0.460924	0.460615	9	38197082	0.006915	0.38	9	38197082	0.001622	1.19E-13
rs1014696	C	T	C	T	-0.01239	-0.00375	0.436149	0.607589	14	1.01E+08	0.006992	0.59	14	1.01E+08	0.001749	1.47E-12
rs1019312	C	G	C	G	0.014524	0.010959	0.453915	0.244717	2	2.01E+08	0.007995	0.17	2	2.01E+08	0.001682	6.12E-18
rs1025687	C	T	C	T	-0.01994	0.008473	0.62446	0.628002	18	50621423	0.007101	0.23	18	50621423	0.001669	7.44E-33
rs1042286	T	C	T	C	-0.01432	-0.00011	0.626555	0.424111	19	33403940	0.006775	0.99	19	33403940	0.001713	6.75E-17
rs1043106	T	G	T	G	0.016202	0.004057	0.429197	0.43828	11	1.08E+08	0.006791	0.55	11	1.08E+08	0.001626	2.36E-23
rs1045242	G	A	G	A	-0.01977	-0.00732	0.245596	0.157541	5	1.19E+08	0.009311	0.43	5	1.19E+08	0.001887	1.20E-25
rs1047891	A	C	A	C	-0.01973	-0.00569	0.282512	0.165946	2	2.11E+08	0.009281	0.54	2	2.11E+08	0.001818	2.03E-27
rs1050991	A	T	A	T	-0.02061	0.001932	0.168296	0.308117	10	1.1E+08	0.007368	0.79	10	1.1E+08	0.002191	5.44E-21
rs1051762	T	C	T	C	-0.01126	-0.00471	0.244402	0.20317	4	1.56E+08	0.008685	0.59	4	1.56E+08	0.001904	3.49E-09
rs1074960	A	G	A	G	0.017602	-0.01006	0.807023	0.858549	10	80489996	0.009726	0.3	10	80489996	0.00204	6.69E-18
rs1075728	T	A	T	A	-0.03163	0.007482	0.248405	0.579011	9	22143571	0.006729	0.27	9	22143571	0.002027	8.06E-55
rs1078632	G	C	G	C	0.039541	0.009482	0.603099	0.682037	10	97308981	0.007281	0.19	10	97308981	0.001648	3.67E-127
rs1080633	A	T	A	T	0.012971	-0.00323	0.443108	0.536744	6	85729611	0.006743	0.63	6	85729611	0.001677	1.10E-14
rs1082872	T	G	T	G	-0.04863	0.002415	0.287684	0.044669	10	24929314	0.016524	0.88	10	24929314	0.001861	1.80E-150
rs1083173	A	G	A	G	0.016267	0.002741	0.678861	0.630644	11	12114765	0.007135	0.7	11	12114765	0.001766	3.44E-20
rs1086436	C	T	C	T	0.020594	-0.01726	0.570129	0.848223	1	8858254	0.008815	0.05	1	8858254	0.001703	1.30E-33
rs1088957	A	G	A	G	-0.03901	-0.00965	0.455637	0.787464	1	65683658	0.007711	0.21	1	65683658	0.001806	2.13E-103
rs1093547	T	G	T	G	0.01307	-0.0015	0.449586	0.444284	3	98698056	0.006985	0.83	3	98698056	0.001627	1.01E-15
rs1093658	A	G	A	G	-0.0111	0.01174	0.656136	0.738232	3	1.7E+08	0.007714	0.13	3	1.7E+08	0.001696	6.09E-11
rs1094803	A	C	A	C	0.020635	-0.00153	0.190908	0.154899	6	42542567	0.009531	0.87	6	42542567	0.002057	1.16E-23
rs1095199	C	T	C	T	0.019729	-0.00612	0.487741	0.620317	7	6719307	0.007075	0.39	7	6719307	0.001779	1.57E-28
rs109536	C	G	C	G	0.016144	0.00519	0.337819	0.630644	9	1.34E+08	0.007222	0.47	9	1.34E+08	0.001838	1.68E-18
rs1099547	C	T	C	T	-0.0257	-0.00912	0.467959	0.301393	10	63250912	0.007321	0.21	10	63250912	0.001617	7.68E-57
rs1102713	C	A	C	A	-0.00163	-0.00051	0.426687	0.331172	1	1.73E+08	0.00712	0.94	1	1.73E+08	0.001697	0.337047

rs1104252	A	G	A	G	-0.01157	0.001353	0.492945	0.422911	11	9880564	0.007112	0.85	11	9880564	0.001625	1.11E-12
rs1104842	G	C	G	C	0.011895	0.012613	0.477652	0.391451	12	26187053	0.007014	0.072	12	26187053	0.001621	2.26E-13
rs1105287	G	A	G	A	0.023737	-0.00153	0.365734	0.389289	12	9753094	0.006997	0.83	12	9753094	0.001673	1.18E-45
rs1110081	T	C	T	C	0.018143	-0.00529	0.432863	0.416907	4	1.44E+08	0.006961	0.45	4	1.44E+08	0.00166	8.97E-28
rs1110487	T	C	T	C	-0.02177	0.00281	0.668701	0.802113	12	88435517	0.008483	0.74	12	88435517	0.001782	2.81E-34
rs1124009	A	G	A	G	0.000209	-0.00983	0.796311	0.662344	1	1.48E+08	0.007394	0.18	1	1.48E+08	0.002296	0.927404
rs1125007	G	A	G	A	0.020425	-0.01171	0.479336	0.096542	8	10790313	0.010752	0.28	8	10790313	0.001745	1.28E-31
rs1135704	A	G	A	G	-0.01805	-0.00487	0.200343	0.254083	7	76025659	0.008	0.54	7	76025659	0.002104	1.01E-17
rs1148947	T	C	T	C	-0.04628	-0.00357	0.031526	0.014649	10	88017202	0.02876	0.9	10	88017202	0.00463	1.70E-23
rs1158619	C	T	C	T	-0.02554	0.052561	0.192388	0.013689	1	2.08E+08	0.028247	0.063	1	2.08E+08	0.002093	3.23E-34
rs1160232	G	T	G	T	0.030765	0.002581	0.113921	0.097743	11	1.23E+08	0.011604	0.82	11	1.23E+08	0.00255	1.75E-33
rs1163825	C	T	C	T	0.017243	-0.00329	0.129433	0.15586	15	58480608	0.009397	0.73	15	58480608	0.002433	1.43E-12
rs1164412	T	C	T	C	-0.01621	-0.00325	0.505864	0.200528	16	57025062	0.008367	0.7	16	57025062	0.001724	5.92E-21
rs1164628	T	C	T	C	-0.01673	0.015144	0.368288	0.39001	16	3669343	0.006895	0.028	16	3669343	0.001722	2.87E-22
rs1164913	T	C	T	C	-0.01902	-0.0087	0.130817	0.049952	1	26846193	0.015524	0.58	1	26846193	0.002415	3.49E-15
rs1165641	G	A	G	A	0.013816	0.001596	0.564421	0.525456	17	45260603	0.006771	0.81	17	45260603	0.001631	2.61E-17
rs1167071	A	C	A	C	-0.0144	0.013702	0.298241	0.088617	19	11808063	0.011875	0.25	19	11808063	0.001833	4.11E-15
rs1168830	T	C	T	C	0.020983	0.009388	0.122939	0.18732	2	1.28E+08	0.008683	0.28	2	1.28E+08	0.002513	7.15E-17
rs1168989	T	C	T	C	-0.02705	0.007185	0.072261	0.20341	2	70128819	0.008473	0.4	2	70128819	0.003288	2.03E-16
rs1172570	G	A	G	A	0.058085	-0.00269	0.379726	0.504563	4	74094279	0.006774	0.69	4	74094279	0.001664	1.00E-200
rs1180355	A	G	A	G	7.60E-05	0.008447	0.404899	0.555716	1	1.18E+08	0.006957	0.22	1	1.18E+08	0.001699	0.964267
rs1181210	A	G	A	G	-0.03855	-0.02545	0.039746	0.009846	17	57385975	0.034091	0.46	17	57385975	0.004141	1.38E-20
rs1182182	T	C	T	C	0.017111	-0.00715	0.282124	0.20293	7	2833600	0.008671	0.41	7	2833600	0.00179	1.25E-21
rs1188720	G	T	G	T	-0.02104	0.004111	0.627144	0.638569	2	61547338	0.007155	0.57	2	61547338	0.001663	1.16E-36
rs1196051	G	A	G	A	0.019768	-0.0032	0.226192	0.096542	5	35883139	0.011572	0.78	5	35883139	0.001947	3.52E-24
rs1196064	C	A	C	A	-0.01661	0.000395	0.621366	0.745677	5	1.49E+08	0.007698	0.96	5	1.49E+08	0.001791	1.86E-20
rs1197477	G	T	G	T	0.018542	0.001556	0.111518	0.323967	7	80583740	0.007356	0.83	7	80583740	0.002724	1.04E-11
rs1206287	G	T	G	T	0.019572	0.009027	0.185431	0.069164	1	2.24E+08	0.013703	0.51	1	2.24E+08	0.002116	2.36E-20
rs1220359	T	C	T	C	0.022541	-0.01488	0.200408	0.01489	6	396321	0.028085	0.6	6	396321	0.002335	5.01E-22
rs1227995	T	C	T	C	0.024074	0.001449	0.656352	0.669308	11	47938658	0.007295	0.84	11	47938658	0.001705	3.36E-45
rs1228038	C	T	C	T	0.022897	-0.03475	0.063872	0.024496	11	1.22E+08	0.021826	0.11	11	1.22E+08	0.003346	8.08E-12
rs1237806	T	C	T	C	-0.0158	-0.00182	0.573011	0.382565	9	1.24E+08	0.00698	0.79	9	1.24E+08	0.001713	3.18E-20
rs1245035	A	C	A	C	0.012359	-0.00295	0.549187	0.32781	12	64582269	0.00734	0.69	12	64582269	0.001695	3.15E-13
rs1254030	T	C	T	C	-0.02867	0.000856	0.093536	0.142651	7	66197780	0.009779	0.93	7	66197780	0.003016	2.14E-21
rs1255061	A	G	A	G	-0.02954	0.004466	0.815735	0.805716	8	23109256	0.008786	0.61	8	23109256	0.002116	2.95E-44
rs1260326	C	T	C	T	-0.03131	0.003182	0.575362	0.538665	2	27508073	0.006816	0.64	2	27508073	0.001644	8.68E-81
rs1269170	T	G	T	G	-0.01206	0.003028	0.748598	0.845581	2	1.45E+08	0.009614	0.75	2	1.45E+08	0.001862	9.80E-11
rs1271056	G	A	G	A	0.013297	-0.00307	0.21021	0.251681	22	37633716	0.008013	0.7	22	37633716	0.002103	2.64E-10
rs1275153	G	A	G	A	-0.01873	-0.00561	0.144329	0.260327	1	40891108	0.007824	0.47	1	40891108	0.002339	1.22E-15
rs1276379	C	T	C	T	0.016144	-0.01102	0.473507	0.361191	10	1.02E+08	0.007114	0.12	10	1.02E+08	0.001654	1.79E-22
rs1279246	G	C	G	C	0.015364	0.005697	0.235611	0.173391	11	18054892	0.009221	0.54	11	18054892	0.00192	1.26E-15
rs1285879	C	T	C	T	0.025035	-0.00063	0.607445	0.124159	6	7137130	0.010097	0.95	6	7137130	0.002009	1.27E-35
rs1292254	C	T	C	T	0.013471	-0.00265	0.680733	0.645773	16	50045479	0.007082	0.71	16	50045479	0.001744	1.19E-14

rs1292995	A	G	A	G	-0.03005	-0.02568	0.074569	0.009126	16	2094893	0.036229	0.48	16	2094893	0.003578	4.74E-17
rs1293085	G	A	G	A	0.015442	-0.00452	0.458787	0.208453	16	81568607	0.008291	0.59	16	81568607	0.001692	7.42E-20
rs1294135	G	A	G	A	-0.01661	0.008668	0.463769	0.101345	17	17813217	0.011203	0.44	17	17813217	0.001846	2.44E-19
rs1301326	T	C	T	C	0.022612	-0.01221	0.075778	0.070845	2	1.92E+08	0.01339	0.36	2	1.92E+08	0.00307	1.85E-13
rs1303374	T	G	T	G	-0.01653	0.006213	0.619078	0.435639	2	66433271	0.006723	0.36	2	66433271	0.001731	1.37E-21
rs1306357	A	T	A	T	0.027327	-0.00179	0.401584	0.307637	3	47046347	0.007486	0.81	3	47046347	0.001805	9.44E-52
rs1315623	G	T	G	T	-0.01167	-0.00098	0.66371	0.480307	5	55634423	0.006819	0.89	5	55634423	0.00201	6.55E-09
rs1318072	A	G	A	G	-0.01847	-0.00193	0.789384	0.769452	5	1.8E+08	0.008099	0.81	5	1.8E+08	0.002035	1.21E-19
rs1320717	T	C	T	C	-0.02066	0.003738	0.135984	0.168108	6	41198081	0.009107	0.68	6	41198081	0.002435	2.30E-17
rs1322599	T	C	T	C	-0.02778	0.000673	0.266935	0.598463	6	16758194	0.00678	0.92	6	16758194	0.00203	1.34E-42
rs1328328	G	C	G	C	-0.02319	0.001076	0.130574	0.084774	9	1.29E+08	0.012318	0.93	9	1.29E+08	0.002511	2.75E-20
rs1329166	G	A	G	A	0.025828	0.037578	0.180746	0.017531	9	282738	0.024433	0.12	9	282738	0.002335	2.06E-28
rs1358980	T	C	T	C	0.014867	0.014474	0.490836	0.585014	6	43796814	0.006844	0.034	6	43796814	0.00166	3.51E-19
rs1366045	C	T	C	T	-0.02356	-0.01096	0.427321	0.658501	3	42867558	0.007362	0.14	3	42867558	0.001649	2.90E-46
rs1366689	C	T	C	T	0.014961	0.003045	0.193292	0.333093	4	1.47E+08	0.007307	0.68	4	1.47E+08	0.002092	8.99E-13
rs138918	A	G	A	G	0.014738	0.005681	0.29934	0.028098	22	43163445	0.019277	0.77	22	43163445	0.001937	2.86E-14
rs1412444	T	C	T	C	0.018435	0.011747	0.321679	0.347022	10	89243170	0.007105	0.098	10	89243170	0.001729	1.62E-26
rs1434077	C	G	C	G	-0.01369	-0.00822	0.209788	0.089337	4	78719704	0.011483	0.47	4	78719704	0.002024	1.40E-11
rs1446707	G	A	G	A	0.395514	-0.00805	0.997167	0.008405	2	2.18E+08	0.03774	0.83	2	2.18E+08	0.017605	1.05E-111
rs1456055	T	C	T	C	-0.05219	-0.00853	0.254829	0.044909	19	45239644	0.015978	0.59	19	45239644	0.002065	7.86E-141
rs1456896	T	C	T	C	0.015801	-0.00242	0.637986	0.411623	7	50264865	0.006613	0.71	7	50264865	0.001688	8.49E-21
rs1468102	G	C	G	C	0.011468	-0.0123	0.376468	0.648655	11	2983296	0.007175	0.086	11	2983296	0.001748	5.57E-11
rs1533299	T	C	T	C	0.011253	-0.0042	0.384855	0.447887	2	1.11E+08	0.00679	0.54	2	1.11E+08	0.001711	5.04E-11
rs1536760	T	C	T	C	0.015182	-0.01042	0.402967	0.663064	13	1.14E+08	0.007173	0.15	13	1.14E+08	0.001813	5.78E-17
rs1539009	T	C	T	C	-0.01236	0.007506	0.634095	0.824207	1	2.14E+08	0.008955	0.4	1	2.14E+08	0.001712	5.41E-13
rs1693037	C	T	C	T	0.01552	0.012584	0.168441	0.086455	12	3278531	0.0123	0.31	12	3278531	0.002197	1.67E-12
rs1700589	A	G	A	G	-0.04393	5.10E-05	0.211959	0.336455	4	82626709	0.007295	0.99	4	82626709	0.001999	5.53E-107
rs1704143	C	A	C	A	0.034578	-0.00092	0.05961	0.083814	12	1.01E+08	0.012534	0.94	12	1.01E+08	0.003438	9.23E-24
rs1710014	G	A	G	A	0.015728	0.004746	0.178957	0.157301	5	1.43E+08	0.009524	0.62	5	1.43E+08	0.002136	1.85E-13
rs1738074	C	T	C	T	-0.01958	0.007922	0.50973	0.48439	6	1.59E+08	0.006633	0.23	6	1.59E+08	0.001642	9.39E-33
rs1738668	G	T	G	T	0.019768	-0.02127	0.152276	0.061239	14	35132881	0.013903	0.13	14	35132881	0.002261	2.38E-18
rs1770596	G	A	G	A	-0.01999	-0.02722	0.193134	0.011768	2	1.87E+08	0.030178	0.37	2	1.87E+08	0.002309	5.20E-18
rs1771000	A	G	A	G	0.021027	0.011888	0.164636	0.082373	6	1.53E+08	0.01248	0.34	6	1.53E+08	0.002171	3.78E-22
rs1800961	T	C	T	C	-0.034	-0.00754	0.026449	0.01489	20	44413724	0.027641	0.78	20	44413724	0.005016	1.25E-11
rs180942	G	T	G	T	-0.01222	-0.00852	0.581572	0.488713	10	1.14E+08	0.006802	0.21	10	1.14E+08	0.001668	2.45E-13
rs1863176	C	G	C	G	0.015695	-0.00592	0.552717	0.818924	2	12790966	0.008661	0.49	2	12790966	0.001649	1.87E-21
rs1883587	A	G	A	T	-0.03195	0.088895	0.071663	0.005043	12	1.2E+08	0.048578	0.067	12	1.2E+08	0.003499	7.11E-20
rs1894401	A	G	A	G	-0.0157	-0.00376	0.587311	0.816042	15	90885812	0.008629	0.66	15	90885812	0.001746	2.55E-19
rs1926168	T	C	T	C	0.199981	-0.04495	0.005104	0.021854	11	1.18E+08	0.022703	0.048	11	1.18E+08	0.01373	5.19E-48
rs1929863	T	C	T	C	-0.01771	0.012623	0.392805	0.177474	1	2.27E+08	0.008886	0.16	1	2.27E+08	0.00178	2.73E-23
rs1933295	G	A	G	A	-0.01541	-0.01397	0.800867	0.910663	1	61641349	0.011515	0.22	1	61641349	0.002124	4.27E-13
rs1985157	C	T	C	T	0.014651	-0.00947	0.377911	0.182277	19	18402784	0.00877	0.28	19	18402784	0.001761	9.46E-17
rs2012011	A	G	A	G	-0.02518	0.006737	0.135421	0.159702	6	46925618	0.009414	0.47	6	46925618	0.002354	1.14E-26

rs2038700	C	T	C	T	0.034194	-0.00916	0.421037	0.450528	14	24992783	0.006918	0.19	14	24992783	0.00165	2.52E-95
rs205438	T	C	T	C	0.013686	-0.01215	0.707266	0.683958	16	28082353	0.007323	0.097	16	28082353	0.001837	9.79E-14
rs2062250	A	G	A	G	0.043089	-0.016	0.92808	0.908982	15	64379803	0.011195	0.15	15	64379803	0.003232	1.62E-40
rs2073081	C	T	C	T	-0.01732	0.004812	0.225362	0.362152	22	43939864	0.007013	0.49	22	43939864	0.00201	7.13E-18
rs2075998	C	T	C	T	-0.02128	0.004366	0.660041	0.790346	1	23523871	0.008267	0.6	1	23523871	0.001754	7.79E-34
rs2096507	A	G	A	G	-0.01416	0.009214	0.465457	0.43756	21	46522003	0.006922	0.18	21	46522003	0.001615	1.95E-18
rs2111485	G	A	G	A	-0.01697	-0.00912	0.508109	0.201009	2	1.62E+08	0.008372	0.28	2	1.62E+08	0.001713	4.19E-23
rs2212930	G	A	G	A	0.014763	0.011937	0.532356	0.340298	21	38486979	0.00728	0.1	21	38486979	0.001676	1.32E-18
rs2219143	A	G	A	G	0.013959	-0.00504	0.392297	0.370317	9	2622278	0.007119	0.48	9	2622278	0.001788	6.07E-15
rs2240128	C	T	C	T	0.016375	0.001681	0.143782	0.239914	19	2187277	0.008148	0.84	19	2187277	0.002449	2.38E-11
rs2267630	A	G	A	G	0.032622	-0.02739	0.057081	0.084054	19	42251546	0.012311	0.026	19	42251546	0.003549	4.13E-20
rs2269996	A	G	A	G	-0.01457	0.002232	0.268424	0.532421	7	1.4E+08	0.006601	0.74	7	1.4E+08	0.001933	5.00E-14
rs2271400	T	C	T	C	-0.02271	-0.00149	0.699045	0.754323	8	55878685	0.007896	0.85	8	55878685	0.001852	1.56E-34
rs2286599	A	G	A	G	0.037565	-0.01926	0.140546	0.045149	12	6390367	0.016667	0.25	12	6390367	0.00273	5.05E-43
rs2304130	G	A	G	A	0.01811	0.003437	0.091892	0.127041	19	19678719	0.010267	0.74	19	19678719	0.0028	1.02E-10
rs2347696	C	T	C	T	-0.01585	0.004325	0.36298	0.276897	7	1.35E+08	0.007719	0.58	7	1.35E+08	0.00168	4.35E-21
rs2369842	T	G	T	G	0.017518	-0.00416	0.247619	0.107109	16	1316424	0.010839	0.7	16	1316424	0.001989	1.33E-18
rs2371108	T	G	T	G	0.019353	-0.01471	0.486269	0.839097	3	27715527	0.009271	0.11	3	27715527	0.001743	1.29E-28
rs2458280	G	A	G	A	-0.0174	-0.00605	0.282275	0.364073	8	1.03E+08	0.007012	0.39	8	1.03E+08	0.001841	3.70E-21
rs2462015	C	T	C	T	0.014524	0.00366	0.571654	0.399135	10	30194159	0.006862	0.59	10	30194159	0.001722	3.45E-17
rs2469434	C	T	C	T	0.011856	-0.00672	0.401843	0.350144	18	69876810	0.007244	0.35	18	69876810	0.001654	7.80E-13
rs247826	T	C	T	C	0.032607	-0.00359	0.204419	0.173631	16	84549359	0.009145	0.69	16	84549359	0.002013	5.84E-59
rs2497833	T	A	T	A	0.010357	-0.00403	0.502341	0.60903	10	17983041	0.006976	0.56	10	17983041	0.001629	2.11E-10
rs2501309	C	T	C	T	-0.01414	0.018203	0.582509	0.323967	1	22015221	0.007168	0.011	1	22015221	0.001713	1.55E-16
rs2561758	G	A	G	A	-0.03566	0.042767	0.727742	0.993996	5	1.74E+08	0.044591	0.34	5	1.74E+08	0.002048	6.86E-68
rs2583839	A	G	A	G	-0.01327	-0.00192	0.458589	0.617915	9	1.31E+08	0.00694	0.78	9	1.31E+08	0.00165	9.45E-16
rs2665405	A	G	A	G	0.03069	-0.00279	0.504866	0.413064	17	59797931	0.006866	0.68	17	59797931	0.001635	1.38E-78
rs2710804	C	T	C	T	0.018236	0.009366	0.330595	0.224304	7	36044919	0.00829	0.26	7	36044919	0.001732	6.82E-26
rs2713573	T	C	T	C	0.02426	0.003313	0.719776	0.763208	3	1.29E+08	0.008071	0.68	3	1.29E+08	0.001807	4.92E-41
rs2807742	A	G	A	G	0.031059	-0.00103	0.811673	0.914986	10	28492438	0.012074	0.93	10	28492438	0.002101	2.16E-49
rs2853075	A	G	A	G	0.053472	0.020268	0.040239	0.011768	4	36310920	0.032006	0.53	4	36310920	0.00424	1.98E-36
rs2857622	A	G	A	G	0.021127	0.004725	0.183354	0.318684	15	1.01E+08	0.007322	0.52	15	1.01E+08	0.002219	1.79E-21
rs2861524	C	T	C	T	-0.02142	-0.00945	0.195268	0.207493	8	54538633	0.008471	0.26	8	54538633	0.002102	2.29E-24
rs2867816	T	G	T	G	-0.01443	-0.00458	0.309506	0.301873	17	43020464	0.007289	0.53	17	43020464	0.001802	1.24E-15
rs2875187	T	A	T	A	-0.02047	0.014434	0.126353	0.043948	5	71009726	0.016459	0.38	5	71009726	0.003013	1.12E-11
rs2883497	C	T	C	T	-0.01375	0.006321	0.32825	0.325648	8	27337604	0.007242	0.38	8	27337604	0.001729	1.88E-15
rs2889525	A	G	A	G	0.065484	-0.01379	0.453955	0.335495	6	32477302	0.007215	0.056	6	32477302	0.001701	1.00E-200
rs2904270	A	G	A	G	-0.01295	-0.00732	0.542493	0.604707	20	50568735	0.007096	0.3	20	50568735	0.001629	1.94E-15
rs2915607	C	T	C	T	0.01693	0.00883	0.771819	0.774015	8	30422400	0.008012	0.27	8	30422400	0.001936	2.31E-18
rs2963471	A	G	A	G	0.013951	-0.00049	0.225004	0.334534	5	1.59E+08	0.00734	0.95	5	1.59E+08	0.001918	3.63E-13
rs303753	A	G	A	G	-0.01823	-0.0085	0.284667	0.086215	18	23494958	0.011967	0.48	18	23494958	0.001852	8.04E-23
rs3131610	A	G	A	G	-0.02358	-0.01305	0.310521	0.533381	15	50507898	0.006773	0.054	15	50507898	0.00181	9.10E-39
rs333947	A	G	A	G	0.021734	-0.01382	0.178853	0.285062	1	1.1E+08	0.007455	0.064	1	1.1E+08	0.002207	7.34E-23

rs337637	A	G	A	G	0.016784	-0.00461	0.353088	0.287464	4	38602849	0.007465	0.54	4	38602849	0.001689	3.14E-23
rs3411241	G	A	G	A	0.026172	0.00495	0.068514	0.117675	14	69374185	0.010434	0.64	14	69374185	0.003221	4.67E-16
rs3418057	T	C	T	C	0.018208	0.011366	0.170595	0.041547	1	2.21E+08	0.016939	0.5	1	2.21E+08	0.002135	1.55E-17
rs342242	C	T	C	T	-0.01674	-0.00805	0.392777	0.251921	7	1.07E+08	0.007785	0.3	7	1.07E+08	0.001691	4.52E-23
rs3495231	A	G	A	G	-0.03328	0.035669	0.041361	0.014169	20	11196407	0.029242	0.22	20	11196407	0.004267	6.58E-15
rs3573424	C	T	C	T	0.017843	-0.00365	0.434888	0.449568	4	712911	0.007046	0.6	4	712911	0.001695	6.91E-26
rs3732378	A	G	A	G	0.01992	-0.01315	0.142533	0.028818	3	39265671	0.019969	0.51	3	39265671	0.002314	7.82E-18
rs3735007	A	G	A	G	-0.01095	-0.00417	0.470249	0.363353	7	1.39E+08	0.006991	0.55	7	1.39E+08	0.00163	1.89E-11
rs3735485	G	A	G	A	0.040271	0.008335	0.864755	0.900576	7	44969742	0.01152	0.47	7	44969742	0.002392	1.56E-63
rs3747869	C	A	C	A	0.038139	-0.00446	0.887996	0.808838	10	71760875	0.008657	0.61	10	71760875	0.002665	2.12E-46
rs3781454	A	G	A	G	0.023296	0.008515	0.649431	0.668348	10	1.25E+08	0.0072	0.24	10	1.25E+08	0.001719	8.60E-42
rs3793537	C	G	C	G	0.017823	0.012812	0.295123	0.289145	9	35687559	0.007535	0.089	9	35687559	0.001772	8.89E-24
rs3795503	T	C	T	C	-0.01422	0.001332	0.346145	0.46926	1	1.81E+08	0.006811	0.84	1	1.81E+08	0.001753	5.18E-16
rs3812849	C	A	C	A	0.018345	0.006585	0.247233	0.163064	13	74127599	0.009065	0.47	13	74127599	0.001913	9.64E-22
rs3825761	C	T	C	T	-0.01196	-0.00255	0.61884	0.54635	14	1.05E+08	0.006833	0.71	14	1.05E+08	0.001724	4.13E-12
rs385893	C	T	C	T	0.01611	0.008994	0.562514	0.770173	9	4763176	0.00796	0.26	9	4763176	0.001665	4.18E-22
rs38859	T	C	T	C	0.011484	0.0045	0.456869	0.471902	7	1.17E+08	0.006811	0.51	7	1.17E+08	0.001626	1.70E-12
rs3917932	G	C	G	C	-0.04258	-0.00705	0.585271	0.610471	1	36478315	0.007002	0.31	1	36478315	0.001642	3.64E-148
rs3931	G	A	G	A	0.023208	-0.00464	0.318355	0.259126	2	1.69E+08	0.007782	0.55	2	1.69E+08	0.00173	5.62E-41
rs398474	A	G	A	G	0.01361	-0.00467	0.325327	0.56244	15	62506836	0.006814	0.49	15	62506836	0.001758	1.03E-14
rs413141	G	A	G	A	0.015911	0.007688	0.813462	0.756484	19	6675978	0.008056	0.34	19	6675978	0.002195	4.33E-13
rs4142967	T	C	T	C	-0.01939	-0.00832	0.369719	0.085735	6	90286630	0.011747	0.48	6	90286630	0.001774	9.18E-28
rs4147108	G	A	G	A	0.015583	0.014503	0.254718	0.347262	1	38833751	0.007264	0.046	1	38833751	0.00187	8.40E-17
rs4256842	T	C	T	C	0.003978	-0.00126	0.710618	0.744236	1	1.07E+08	0.007763	0.87	1	1.07E+08	0.001822	0.029001
rs4272720	G	C	G	A	-0.02342	0.010305	0.223329	0.051873	10	49055156	0.015438	0.5	10	49055156	0.002153	1.63E-27
rs4355178	A	G	A	G	-0.01831	0.004657	0.368474	0.793948	20	1950227	0.008426	0.58	20	1950227	0.001812	5.42E-24
rs439749	A	G	A	G	-0.01447	0.009814	0.707674	0.923391	20	57393057	0.012236	0.42	20	57393057	0.00188	1.46E-14
rs4413892	A	G	A	G	0.029225	-0.00283	0.23589	0.042027	9	1.36E+08	0.016977	0.87	9	1.36E+08	0.002003	3.73E-48
rs4432538	A	G	A	G	-0.0196	0.007534	0.485651	0.335975	20	8626746	0.007385	0.31	20	8626746	0.001616	8.54E-34
rs445	T	C	T	C	-0.08936	-0.00889	0.145643	0.332133	7	92779056	0.007238	0.22	7	92779056	0.002361	1.00E-200
rs447802	C	T	C	T	-0.01474	-0.00171	0.369561	0.728866	19	48613102	0.007511	0.82	19	48613102	0.001913	1.38E-14
rs4519779	A	G	A	G	0.017152	-0.00301	0.489981	0.397695	4	1.51E+08	0.007137	0.67	4	1.51E+08	0.001644	1.89E-25
rs4534048	G	A	G	A	0.011192	-0.00996	0.239541	0.154659	7	21904567	0.009386	0.29	7	21904567	0.001939	8.02E-09
rs4568584	T	C	T	C	0.014753	0.01854	0.75381	0.942363	8	1.29E+08	0.013948	0.18	8	1.29E+08	0.001951	4.21E-14
rs4574603	T	C	T	C	-0.016	0.0095	0.503268	0.618876	6	87107187	0.007067	0.18	6	87107187	0.001617	4.46E-23
rs4632345	A	G	A	G	-0.01328	0.000229	0.602441	0.268492	2	16521386	0.007461	0.98	2	16521386	0.001718	1.12E-14
rs464609	A	G	A	G	0.010524	-0.00076	0.502579	0.404899	5	34654372	0.006883	0.91	5	34654372	0.001629	1.09E-10
rs4703133	G	C	G	C	-0.01345	0.020352	0.363088	0.23439	5	1.01E+08	0.007892	0.0099	5	1.01E+08	0.00168	1.27E-15
rs4703881	A	G	A	G	-0.03324	-0.02291	0.894852	0.960135	5	72446024	0.017565	0.19	5	72446024	0.002636	2.03E-36
rs4704835	A	G	A	G	-0.02041	0.018223	0.846064	0.933958	5	1.57E+08	0.013926	0.19	5	1.57E+08	0.002212	2.96E-20
rs4710971	G	A	G	A	-0.0192	-0.00591	0.622674	0.608549	6	21383185	0.007114	0.41	6	21383185	0.001656	4.67E-31
rs473068	G	A	G	A	0.014143	-0.0064	0.155267	0.184198	1	1.12E+08	0.008691	0.46	1	1.12E+08	0.002251	3.44E-10
rs4734879	G	A	G	A	-0.02032	-0.00763	0.301273	0.427474	8	1.06E+08	0.006899	0.27	8	1.06E+08	0.00176	8.18E-31

rs4760	G	A	G	A	-0.06975	0.010138	0.147921	0.016811	19	43648948	0.026562	0.7	19	43648948	0.002563	5.50E-163
rs4761234	C	T	C	T	0.013948	-0.00975	0.458309	0.431076	12	69338325	0.006916	0.16	12	69338325	0.001631	1.27E-17
rs476386	T	C	T	C	0.016191	0.011225	0.45272	0.49952	1	1.08E+08	0.006845	0.1	1	1.08E+08	0.001616	1.33E-23
rs4803336	T	C	T	C	-0.01582	0.013597	0.16326	0.101105	19	40420075	0.011302	0.23	19	40420075	0.002225	1.19E-12
rs4807440	T	G	T	G	0.022625	-0.00202	0.602487	0.428674	19	1026478	0.007014	0.77	19	1026478	0.001773	2.87E-37
rs4811528	A	G	A	G	-0.01276	0.000246	0.732254	0.644092	20	38159027	0.007085	0.97	20	38159027	0.001876	1.08E-11
rs4836600	T	A	T	A	-0.01516	0.00866	0.710271	0.448847	9	1.28E+08	0.006889	0.21	9	1.28E+08	0.00211	7.10E-13
rs4842200	C	T	C	T	0.017095	0.010577	0.305443	0.392411	9	1.35E+08	0.007052	0.13	9	1.35E+08	0.001778	7.44E-22
rs4842316	T	C	T	C	-0.01439	-0.00794	0.594967	0.42147	12	79580785	0.007065	0.26	12	79580785	0.001758	2.88E-16
rs4899652	G	A	G	A	0.015629	-0.00388	0.376514	0.106388	14	77382629	0.010856	0.72	14	77382629	0.001776	1.44E-18
rs4948097	G	A	G	A	-0.01634	-0.00924	0.723378	0.68732	7	55988878	0.007592	0.22	7	55988878	0.001814	2.27E-19
rs4978410	T	G	T	G	0.020583	-0.00452	0.174088	0.213497	9	1.1E+08	0.008451	0.59	9	1.1E+08	0.00216	1.67E-21
rs519464	G	A	G	A	-0.00588	-0.01221	0.743743	0.7805	1	1.78E+08	0.008265	0.14	1	1.78E+08	0.001873	0.001687
rs554743	T	C	T	C	-0.01256	0.01427	0.684246	0.60879	20	3681495	0.007009	0.042	20	3681495	0.001845	1.06E-11
rs5608240	C	T	C	T	0.013923	0.004333	0.448707	0.660183	3	1.57E+08	0.007139	0.54	3	1.57E+08	0.001672	8.73E-17
rs5618886	C	T	C	T	-0.02408	-0.00416	0.383224	0.382565	1	2.47E+08	0.006971	0.55	1	2.47E+08	0.001657	8.58E-48
rs5638817	T	G	T	G	0.056711	-0.00633	0.283687	0.184918	7	28684757	0.008702	0.47	7	28684757	0.001829	1.00E-200
rs571497	A	G	A	G	-0.03518	-0.03028	0.157579	0.009606	19	7762944	0.035329	0.39	19	7762944	0.002506	9.49E-45
rs5752360	T	C	T	C	-0.01505	0.001495	0.202984	0.367195	22	26629953	0.007202	0.84	22	26629953	0.002164	3.65E-12
rs5840842	C	T	C	T	-0.02343	-0.00409	0.229036	0.376801	4	56903658	0.007021	0.56	4	56903658	0.001958	5.62E-33
rs5963187	C	T	C	T	-0.03579	-0.00324	0.937229	0.717819	15	79984036	0.007533	0.67	15	79984036	0.003967	1.97E-19
rs6046684	A	C	A	C	-0.02075	0.007336	0.119365	0.232949	7	7985209	0.008159	0.37	7	7985209	0.00257	7.17E-16
rs605783	A	T	A	T	-0.0128	-0.00375	0.576137	0.644332	6	10526140	0.007233	0.6	6	10526140	0.001661	1.36E-14
rs610578	G	A	G	A	-0.01539	-0.00491	0.6975	0.833573	12	1.21E+08	0.009105	0.59	12	1.21E+08	0.001769	3.50E-18
rs6185065	A	G	A	G	-0.02199	0.011299	0.125102	0.144332	1	2.26E+08	0.009879	0.25	1	2.26E+08	0.002439	2.07E-19
rs6194205	G	C	G	C	-0.01298	0.004329	0.314764	0.01561	12	48707963	0.025939	0.87	12	48707963	0.001942	2.39E-11
rs6198501	T	C	A	T	-0.03821	0.026487	0.063807	0.01489	14	68740170	0.028527	0.35	14	68740170	0.003308	8.14E-31
rs6211989	A	G	A	G	0.0172	-0.01324	0.129467	0.215658	19	17559147	0.008337	0.11	19	17559147	0.002445	2.05E-12
rs6214107	T	C	T	C	0.022694	-0.00916	0.350726	0.214697	2	65417482	0.008059	0.26	2	65417482	0.001734	4.17E-39
rs6226202	G	A	G	A	-0.02345	0.032708	0.13803	0.995917	3	1.08E+08	0.053938	0.54	3	1.08E+08	0.002376	6.11E-23
rs6246631	T	C	T	C	-0.02053	0.006255	0.178468	0.107589	7	73627755	0.01106	0.57	7	73627755	0.00219	7.72E-21
rs6251850	G	A	G	A	-0.03652	-0.00223	0.111948	0.790586	8	78260489	0.008326	0.79	8	78260489	0.002586	3.01E-45
rs6445826	C	T	C	T	0.011816	-0.00716	0.459288	0.322526	3	56814971	0.00727	0.32	3	56814971	0.001671	1.62E-12
rs646698	A	G	A	G	-0.01646	-0.01056	0.804527	0.920749	9	76702806	0.012314	0.39	9	76702806	0.00208	2.72E-15
rs6584278	T	C	T	C	-0.01662	-0.00257	0.422933	0.506004	10	99518342	0.006782	0.7	10	99518342	0.001643	5.06E-24
rs6585139	C	T	C	T	0.013142	-0.01093	0.730192	0.770653	10	1.12E+08	0.00818	0.18	10	1.12E+08	0.001811	4.16E-13
rs6651495	A	G	A	G	-0.01983	-0.00514	0.453446	0.408261	5	69315756	0.006951	0.46	5	69315756	0.001694	1.24E-31
rs6700311	G	C	G	C	0.012775	-0.01819	0.301315	0.256724	19	34997029	0.007813	0.02	19	34997029	0.001788	9.37E-13
rs6714557	C	T	C	T	-0.03978	-0.003	0.042045	0.774496	5	1.51E+08	0.008208	0.71	5	1.51E+08	0.00444	3.41E-19
rs6734238	G	A	G	A	0.036121	-0.00838	0.325488	0.103987	2	1.13E+08	0.010904	0.44	2	1.13E+08	0.001851	1.04E-84
rs674451	C	T	C	T	0.014444	0.000102	0.278062	0.1244	6	1.38E+08	0.010328	0.99	6	1.38E+08	0.001856	7.59E-15
rs6779340	G	C	G	C	-0.01728	-0.0093	0.265086	0.035783	3	58047974	0.017195	0.59	3	58047974	0.001916	2.00E-19
rs681167	A	G	A	G	0.013	0.002457	0.592277	0.849183	20	2919333	0.009682	0.8	20	2919333	0.001727	5.33E-14

rs6826663	C	T	C	T	0.017565	0.010959	0.140605	0.148895	4	53351440	0.009426	0.24	4	53351440	0.002327	4.56E-14
rs6882226	A	T	A	T	0.020847	0.005397	0.625132	0.634486	5	1.42E+08	0.007024	0.44	5	1.42E+08	0.00166	4.06E-36
rs6985508	A	G	A	G	-0.02398	0.014968	0.369475	0.457733	8	1.41E+08	0.006742	0.026	8	1.41E+08	0.001731	1.35E-43
rs6990622	A	G	A	G	0.01487	-0.00273	0.525465	0.535543	8	1.25E+08	0.00676	0.69	8	1.25E+08	0.001623	5.30E-20
rs710241	A	G	A	G	-0.01202	-0.00811	0.406158	0.60975	1	41732629	0.006919	0.24	1	41732629	0.001718	2.77E-12
rs7107912	A	G	A	G	0.018214	-0.00516	0.435368	0.245917	11	65835781	0.00782	0.51	11	65835781	0.001681	2.57E-27
rs7127313	T	C	T	C	0.016429	-0.00646	0.330652	0.237512	11	1.01E+08	0.008085	0.42	11	1.01E+08	0.001726	1.90E-21
rs720419	T	C	T	C	-0.01297	-0.01326	0.318841	0.336695	4	1.1E+08	0.007295	0.069	4	1.1E+08	0.001728	6.57E-14
rs7223465	G	A	G	A	-0.01452	-0.00097	0.379896	0.20341	17	78260451	0.008433	0.91	17	78260451	0.001831	2.28E-15
rs7225843	C	T	C	T	-0.02572	0.007028	0.211516	0.225744	17	2098531	0.008141	0.39	17	2098531	0.001991	3.91E-38
rs7279086	C	T	C	T	-0.02586	-0.00452	0.262334	0.087416	10	44384812	0.012174	0.71	10	44384812	0.00186	6.53E-44
rs7280064	T	C	T	C	-0.0169	-0.00045	0.211243	0.304515	21	15005329	0.007515	0.95	21	15005329	0.002075	3.92E-16
rs7297371	T	A	T	A	-0.03229	0.074693	0.065131	0.004323	18	76360290	0.049713	0.13	18	76360290	0.003665	1.32E-18
rs7301953	A	G	A	G	-0.01776	0.002609	0.280067	0.128242	12	1.24E+08	0.010331	0.8	12	1.24E+08	0.001811	1.13E-22
rs7326825	A	G	A	G	0.017847	0.002637	0.685511	0.642651	13	49539314	0.007228	0.72	13	49539314	0.001753	2.56E-24
rs7426631	T	G	T	G	-0.07831	0.000318	0.966481	0.825648	2	1.36E+08	0.008795	0.97	2	1.36E+08	0.005095	2.93E-53
rs7537229	A	G	A	G	0.027418	0.025811	0.914613	0.955572	1	56440602	0.016861	0.13	1	56440602	0.002928	8.14E-21
rs7547562	G	C	G	C	0.024332	0.007669	0.133559	0.3439	2	54560455	0.007029	0.28	2	54560455	0.002597	7.73E-21
rs7549164	T	C	T	C	0.018922	0.004695	0.239067	0.475504	1	30751346	0.006805	0.49	1	30751346	0.002064	5.14E-20
rs7578550	A	G	A	G	-0.01269	-0.00223	0.41618	0.677474	1	37474398	0.00723	0.76	1	37474398	0.00173	2.33E-13
rs7586834	T	C	T	C	-0.01779	0.017442	0.200583	0.381364	2	1.52E+08	0.007041	0.013	2	1.52E+08	0.002056	5.24E-18
rs7596385	A	G	A	G	0.059494	-0.02549	0.984009	0.963977	11	95363157	0.018619	0.17	11	95363157	0.007013	2.32E-17
rs7626444	C	G	C	G	-0.02937	-0.007	0.39967	0.251441	3	1.97E+08	0.008011	0.38	3	1.97E+08	0.001652	1.12E-70
rs7641325	A	G	A	G	-0.02428	0.00927	0.402435	0.267531	3	12227104	0.007829	0.24	3	12227104	0.001649	4.75E-49
rs7752183	A	C	A	C	0.017681	0.002578	0.114405	0.123919	6	45604762	0.010551	0.81	6	45604762	0.002549	4.20E-12
rs7771156	A	G	A	G	-0.0108	0.000217	0.393536	0.754083	6	1.51E+08	0.007889	0.98	6	1.51E+08	0.001701	2.20E-10
rs7776054	G	A	G	A	-0.04531	0.002046	0.274602	0.204851	6	1.35E+08	0.008406	0.81	6	1.35E+08	0.001803	3.09E-139
rs7776857	T	G	T	G	-0.01803	-0.01104	0.673183	0.983429	7	22715149	0.025282	0.66	7	22715149	0.001926	8.51E-21
rs7781509	A	G	A	G	-0.0137	0.010302	0.38275	0.325648	7	47325075	0.007254	0.16	7	47325075	0.001653	1.20E-16
rs7803075	G	A	G	A	-0.02291	-0.01492	0.594596	0.130644	7	1.31E+08	0.009942	0.13	7	1.31E+08	0.001924	1.20E-32
rs7846314	T	A	T	A	0.043352	-0.00661	0.190981	0.136407	8	60738272	0.009799	0.5	8	60738272	0.002053	5.99E-99
rs7867734	G	A	G	A	0.027015	0.027672	0.062115	0.005524	11	1.02E+08	0.046388	0.55	11	1.02E+08	0.003743	5.49E-13
rs7901056	G	T	G	T	0.014829	-0.01602	0.343837	0.074448	10	26423554	0.012926	0.22	10	26423554	0.001751	2.58E-17
rs7924975	C	G	C	G	0.012909	0.007398	0.369394	0.348703	11	1.2E+08	0.007144	0.3	11	1.2E+08	0.001672	1.21E-14
rs7926034	A	C	A	C	-0.02019	-0.01261	0.059726	0.082853	4	26296666	0.01266	0.32	4	26296666	0.003438	4.41E-09
rs7955734	G	C	G	C	-0.02722	-0.00868	0.23172	0.184438	12	4223993	0.00883	0.33	12	4223993	0.00196	8.37E-44
rs796003	T	G	T	G	-0.02248	0.002459	0.262161	0.267531	9	83980886	0.007808	0.75	9	83980886	0.001844	3.80E-34
rs796056	C	A	C	A	0.018991	0.023204	0.656902	0.764649	2	1.01E+08	0.008026	0.0038	2	1.01E+08	0.001706	9.43E-29
rs7971658	A	G	A	G	-0.03613	-0.02724	0.120139	0.021374	2	1.43E+08	0.023447	0.25	2	1.43E+08	0.002799	4.40E-38
rs832187	T	C	T	C	-0.01377	0.005351	0.60147	0.465898	3	63847374	0.0069	0.44	3	63847374	0.001659	1.09E-16
rs835574	T	C	T	C	-0.00125	0.001819	0.125241	0.146254	1	1.2E+08	0.009465	0.85	1	1.2E+08	0.002553	0.623561
rs853198	C	A	C	A	-0.01719	-0.00546	0.705839	0.950288	17	37489236	0.015484	0.72	17	37489236	0.001822	4.17E-21
rs874356	T	A	T	A	0.011326	-0.01637	0.57487	0.431556	22	31157368	0.006929	0.018	22	31157368	0.001716	4.20E-11

rs899013	G	A	G	A	0.012627	-0.00524	0.626473	0.745437	11	10455142	0.007708	0.5	11	10455142	0.001673	4.59E-14
rs900835	T	C	T	C	-0.01765	0.000674	0.175798	0.158982	1	42946784	0.009441	0.94	1	42946784	0.002142	1.81E-16
rs9287604	C	G	C	G	0.024289	-0.01	0.701333	0.690682	2	2.37E+08	0.007437	0.18	2	2.37E+08	0.001797	1.35E-41
rs935655	G	T	G	T	0.013546	-0.00296	0.77267	0.698127	2	45840306	0.007452	0.69	2	45840306	0.001935	2.68E-12
rs9375447	G	A	G	A	-0.01365	-0.006	0.593063	0.931076	6	1.27E+08	0.012807	0.64	6	1.27E+08	0.00179	2.60E-14
rs9375700	C	T	C	T	0.010475	-0.00238	0.680105	0.761287	6	1.3E+08	0.007989	0.77	6	1.3E+08	0.001752	2.30E-09
rs9390460	C	T	C	T	0.01482	-0.00024	0.481383	0.306436	6	1.47E+08	0.007319	0.97	6	1.47E+08	0.00165	2.89E-19
rs9487043	T	C	T	C	0.019581	-0.00994	0.482771	0.346302	6	1.09E+08	0.007082	0.16	6	1.09E+08	0.001614	8.02E-34
rs9608255	C	T	C	T	-0.01788	-0.00189	0.283797	0.215898	22	24227169	0.008337	0.82	22	24227169	0.001829	1.51E-22
rs9672995	C	T	C	T	-0.01107	0.011358	0.244907	0.451009	15	75005260	0.006835	0.097	15	75005260	0.00193	1.00E-08
rs9747839	G	C	G	C	0.018677	-0.00398	0.474686	0.379203	17	83136753	0.006985	0.57	17	83136753	0.002157	5.10E-18
rs9773718	T	C	T	C	-0.03677	0.003717	0.200499	0.185879	9	1.33E+08	0.008722	0.67	9	1.33E+08	0.002016	2.77E-74
rs9819371	T	C	T	C	-0.04528	-0.022	0.063948	0.005764	3	1.41E+08	0.045474	0.63	3	1.41E+08	0.003714	3.72E-34
rs9832919	G	A	G	A	0.011269	0.000405	0.366529	0.409222	3	1.32E+08	0.006966	0.95	3	1.32E+08	0.00169	2.70E-11
rs9867398	T	C	T	C	0.019695	0.002079	0.122047	0.146013	3	1.86E+08	0.009648	0.83	3	1.86E+08	0.002555	1.33E-14
rs9905106	C	T	C	T	0.01703	-0.00899	0.688425	0.46878	17	1470224	0.006682	0.18	17	1470224	0.001856	4.85E-20
rs9965539	A	G	A	G	0.024537	0.009347	0.155232	0.178915	18	44393814	0.008914	0.29	18	44393814	0.002268	3.03E-27
rs9970896	C	T	T	A	-0.0574	-0.01319	0.895898	0.025696	1	2.36E+08	0.021555	0.54	1	2.36E+08	0.00293	2.04E-85
rs9977672	A	G	A	G	-0.02149	0.006739	0.237136	0.167867	21	39091357	0.009079	0.46	21	39091357	0.001919	4.70E-29

Supplementary Table 2m. List of SNPs for Neutrophil Count and their effects on TEWL

SNP	effect_alle	other_allel	effect_alle	other_allel	beta.Neu	beta.TEWL	eaf.Neu	eaf.TEWL	chr.TEWL	pos.TEWL	se.TEWL	pval.TEWL	chr.Neu	pos.Neu	se.Neu	pval.Neu
chr1:1740	A	G	A	G	-0.0178	-0.01363	0.296649	0.567723	1	1.74E+08	0.006706	0.042	1	1.74E+08	0.001954	9.15E-20
chr1:2003	C	T	C	T	-0.01331	-0.01388	0.370849	0.700048	1	2E+08	0.007455	0.063	1	2E+08	0.001847	6.04E-13
chr1:2051	A	G	G	C	0.014831	-0.03894	0.610037	0.027618	1	2.05E+08	0.020845	0.062	1	2.05E+08	0.001872	2.44E-15
chr1:3075	A	G	A	G	0.022591	0.007383	0.65746	0.2817	1	30757821	0.007411	0.32	1	30757821	0.001888	5.82E-33
chr10:102	G	A	G	A	-0.0263	0.001069	0.283885	0.506484	10	1.03E+08	0.006855	0.88	10	1.03E+08	0.00195	2.15E-41
chr10:944	C	T	C	G	-0.0125	-0.00885	0.413957	0.189481	10	94450643	0.008576	0.3	10	94450643	0.001803	4.27E-12
chr11:114	C	T	T	A	0.022599	-0.01237	0.51893	0.291787	11	1.14E+08	0.007565	0.1	11	1.14E+08	0.001766	1.90E-37
chr11:122	C	A	T	C	0.016627	0.001008	0.212663	0.103987	11	1.23E+08	0.011166	0.93	11	1.23E+08	0.002184	2.76E-14
chr11:128	T	C	T	C	-0.01454	-0.00836	0.560419	0.184678	11	1.28E+08	0.00888	0.35	11	1.28E+08	0.001875	9.26E-15
chr11:308	G	A	G	A	0.012747	-0.01147	0.693756	0.925552	11	308314	0.012474	0.36	11	308314	0.001942	5.44E-11
chr11:618	A	G	A	G	-0.01678	0.002437	0.178613	0.428674	11	61813896	0.006566	0.71	11	61813896	0.002311	3.95E-13
chr11:658	T	A	A	G	-0.02225	0.002263	0.126825	0.363353	11	65857720	0.006956	0.74	11	65857720	0.00307	4.49E-13
chr11:696	C	T	C	A	-0.03762	-0.01057	0.884995	0.163545	11	69685018	0.009263	0.25	11	69685018	0.002772	6.32E-42
chr11:880	C	T	C	T	0.021507	0.004952	0.488272	0.629923	11	8801946	0.007059	0.48	11	8801946	0.001783	1.74E-33
chr12:111	A	G	A	G	0.032993	-0.00864	0.066471	0.012968	12	1.11E+08	0.028479	0.76	12	1.11E+08	0.0038	4.11E-18
chr12:121	A	G	A	G	0.017151	-0.00427	0.454748	0.426753	12	1.22E+08	0.007041	0.54	12	1.22E+08	0.001803	2.02E-21
chr12:519	T	C	T	C	-0.04651	-0.00871	0.043534	0.852065	12	51912002	0.009598	0.36	12	51912002	0.004681	3.08E-23
chr12:884	G	T	A	G	-0.01825	-0.00244	0.618526	0.198847	12	88449697	0.008477	0.77	12	88449697	0.001808	6.45E-24
chr13:280	A	G	A	G	-0.02106	0.014336	0.106018	0.185879	13	28038749	0.008973	0.11	13	28038749	0.002947	9.26E-13
chr13:495	T	G	C	T	-0.01568	-0.00301	0.220402	0.473343	13	49548545	0.006839	0.66	13	49548545	0.002152	3.35E-13
chr14:926	A	G	G	C	0.014167	-0.00653	0.382296	0.016811	14	92631994	0.026517	0.81	14	92631994	0.00189	6.82E-14
chr15:770	G	A	G	A	0.028221	0.004199	0.858024	0.194765	15	77013944	0.008493	0.62	15	77013944	0.002556	2.62E-28
chr15:775	A	G	A	G	-0.01106	-0.02065	0.387605	0.758646	15	77535756	0.008038	0.01	15	77535756	0.001802	8.73E-10
chr16:211	T	C	T	C	-0.02684	-0.00494	0.123697	0.963737	16	2115629	0.018323	0.79	16	2115629	0.002676	1.20E-23
chr16:304	T	C	C	G	-0.08968	-0.0255	0.128316	0.102305	16	30474072	0.011365	0.025	16	30474072	0.002691	1.00E-200
chr16:815	T	C	T	C	0.020647	-0.00396	0.474144	0.212296	16	81569795	0.008266	0.63	16	81569795	0.001754	6.21E-32
chr17:298	T	C	T	C	-0.02585	0.009617	0.399891	0.315802	17	29831797	0.007337	0.19	17	29831797	0.001809	2.89E-46
chr17:399	C	G	C	G	0.010857	-0.00684	0.62945	0.685879	17	39958724	0.007424	0.36	17	39958724	0.001827	2.90E-09
chr17:480	T	C	T	C	0.016764	-0.01062	0.169513	0.229587	17	48000601	0.008096	0.19	17	48000601	0.002351	1.04E-12
chr17:542	C	T	C	T	0.015447	-0.01744	0.167048	0.044428	17	5422842	0.016745	0.3	17	5422842	0.002386	9.94E-11
chr17:733	C	T	C	T	-0.0227	-0.00364	0.19406	0.92195	17	7337072	0.012945	0.78	17	7337072	0.002222	1.81E-24
chr19:102	T	G	T	G	-0.06521	-0.00512	0.990098	0.610231	19	10223987	0.0071	0.47	19	10223987	0.00968	1.68E-11
chr19:103	G	T	T	C	0.258476	-0.00414	0.991269	0.317963	19	1036019	0.00736	0.57	19	1036019	0.01093	1.45E-123
chr19:394	A	G	A	G	0.018756	-0.00526	0.336694	0.527618	19	39456187	0.006928	0.45	19	39456187	0.001905	7.52E-23
chr19:412	G	A	C	G	-0.02022	-0.00399	0.312816	0.464697	19	41205703	0.006789	0.56	19	41205703	0.001949	3.44E-25
chr19:512	T	C	T	C	0.022198	0.000255	0.114012	0.182757	19	51225221	0.008829	0.98	19	51225221	0.002923	3.21E-14
chr2:1011	C	T	A	C	-0.01461	-0.02356	0.617881	0.235351	2	1.01E+08	0.008026	0.0033	2	1.01E+08	0.001879	7.82E-15
chr2:1447	T	C	G	C	-0.04273	0.002749	0.117949	0.212296	2	1.45E+08	0.008372	0.74	2	1.45E+08	0.002831	1.95E-51
chr2:2416	T	C	T	C	0.020046	-0.01143	0.782204	0.190922	2	24164663	0.008792	0.19	2	24164663	0.00217	2.72E-20
chr2:3124	A	C	C	T	0.013783	0.003398	0.577451	0.116234	2	31240984	0.010678	0.75	2	31240984	0.001839	7.00E-14
chr2:6642	A	G	C	G	0.020672	0.006213	0.200116	0.43756	2	66425850	0.006723	0.36	2	66425850	0.002232	2.12E-20

chr2:8531	G	A	A	C	0.014306	-0.01026	0.770754	0.120797	2	85313657	0.010347	0.32	2	85313657	0.002235	1.61E-10
chr20:637	A	G	A	G	-0.01318	-0.02558	0.370454	0.566523	20	63706054	0.006812	0.00017	20	63706054	0.00186	1.47E-12
chr22:311	C	T	T	G	0.017454	-0.01092	0.185236	0.112872	22	31184360	0.010295	0.29	22	31184360	0.00246	1.34E-12
chr3:1075	A	G	A	C	-0.0129	-0.03844	0.360293	0.003362	3	1.08E+08	0.059382	0.52	3	1.08E+08	0.00187	5.46E-12
chr3:6385	C	G	C	T	0.020366	-0.00301	0.573614	0.530019	3	63856953	0.006888	0.66	3	63856953	0.001897	7.24E-27
chr3:7147	T	C	T	C	-0.01914	-0.01685	0.356008	0.104947	3	71479792	0.010466	0.11	3	71479792	0.001885	3.46E-24
chr4:2628	T	G	C	T	-0.01828	-0.00645	0.315597	0.088136	4	26281184	0.012217	0.6	4	26281184	0.001938	4.15E-21
chr4:5454	A	G	A	T	-0.03413	0.005499	0.043075	0.350144	4	54542708	0.00726	0.45	4	54542708	0.004355	4.87E-15
chr4:6968	C	G	G	A	0.070939	0.004655	0.54133	0.270653	4	6968192	0.007727	0.55	4	6968192	0.001782	1.00E-200
chr5:1228	T	C	T	C	-0.01293	-0.01121	0.641169	0.631604	5	1.23E+08	0.006991	0.11	5	1.23E+08	0.00188	6.43E-12
chr5:1285	T	C	T	G	0.031911	-6.94E-06	0.139022	0.396494	5	1285859	0.006994	1	5	1285859	0.002554	8.84E-36
chr5:1796	T	G	G	A	0.004189	-0.00339	0.529222	0.376081	5	1.8E+08	0.007085	0.63	5	1.8E+08	0.001767	0.017797
chr5:5801	C	T	C	T	-0.0208	-0.00965	0.623998	0.894092	5	58019808	0.01113	0.39	5	58019808	0.00182	3.15E-30
chr6:1093	C	A	G	A	-0.024	-0.01264	0.452649	0.362872	6	1.09E+08	0.007051	0.073	6	1.09E+08	0.001928	1.52E-35
chr6:1265	C	T	C	T	-0.02642	0.008171	0.381089	0.070845	6	1.27E+08	0.012646	0.52	6	1.27E+08	0.00181	3.27E-48
chr6:1367	G	C	T	C	-0.05029	0.003077	0.582546	0.504563	6	1.37E+08	0.006801	0.65	6	1.37E+08	0.001787	3.01E-174
chr6:1675	C	A	G	C	0.017671	0.000347	0.653842	0.414505	6	16752633	0.006748	0.96	6	16752633	0.001848	1.25E-21
chr6:2138	C	T	A	C	-0.00642	0.007061	0.091031	0.39025	6	21382534	0.007104	0.32	6	21382534	0.003315	0.0529
chr6:8727	T	C	C	G	0.020089	-0.01024	0.177091	0.385687	6	87271017	0.007074	0.15	6	87271017	0.002295	2.21E-18
chr7:1491	T	C	T	C	-0.01726	7.85E-05	0.247272	0.887608	7	1.49E+08	0.010766	0.99	7	1.49E+08	0.002092	1.64E-16
chr7:5600	G	A	G	A	0.016542	-0.00726	0.634381	0.691402	7	56004317	0.007594	0.34	7	56004317	0.001884	1.71E-18
chr7:6652	A	G	A	G	-0.03924	0.00587	0.410983	0.06196	7	6652974	0.013891	0.67	7	6652974	0.001874	2.59E-97
chr7:9830	G	A	G	A	0.013424	0.002121	0.446468	0.331892	7	98304289	0.007042	0.76	7	98304289	0.001789	6.39E-14
chr8:1296	A	G	A	G	-0.03871	0.00104	0.032733	0.371037	8	1.3E+08	0.007061	0.88	8	1.3E+08	0.005277	2.29E-13
chr8:5687	G	T	G	A	0.013223	0.000206	0.204533	0.058838	8	5687469	0.014421	0.99	8	5687469	0.002203	1.99E-09
chr8:7043	T	G	C	G	-0.03075	-0.0009	0.098781	0.389529	8	7043782	0.006907	0.9	8	7043782	0.003157	2.16E-22
chr9:1280	A	T	A	T	0.020549	0.009796	0.133782	0.451969	9	1.28E+08	0.006896	0.16	9	1.28E+08	0.002582	1.84E-15
rs1004874	A	G	A	G	0.018526	-0.01589	0.658978	0.055956	2	68735005	0.014624	0.28	2	68735005	0.001884	8.56E-23
rs1007893	C	A	G	A	-0.02804	0.008114	0.595863	0.442843	21	46457681	0.006897	0.24	21	46457681	0.00188	2.95E-50
rs1010109	G	A	G	A	-0.07491	0.007872	0.148643	0.091499	5	1.43E+08	0.012054	0.51	5	1.43E+08	0.002662	3.38E-174
rs1014696	G	A	G	A	-0.01894	-0.00375	0.639771	0.607589	14	1.01E+08	0.006992	0.59	14	1.01E+08	0.002066	5.09E-20
rs1025687	C	T	C	T	0.021917	0.008473	0.544162	0.628002	18	50621423	0.007101	0.23	18	50621423	0.001799	4.20E-34
rs1038193	T	C	C	G	0.014396	0.012984	0.372709	0.345581	9	1.35E+08	0.007301	0.075	9	1.35E+08	0.001845	6.37E-15
rs1042286	C	T	C	T	0.049171	0.000111	0.465718	0.575889	19	33403940	0.006775	0.99	19	33403940	0.002089	2.12E-122
rs1047891	C	T	T	G	-0.01371	-0.00569	0.286789	0.165946	2	2.11E+08	0.009281	0.54	2	2.11E+08	0.001967	3.38E-12
rs1050991	T	C	T	A	-0.06947	0.001932	0.572933	0.308117	10	1.1E+08	0.007368	0.79	10	1.1E+08	0.001792	1.00E-200
rs1074960	A	G	A	G	0.055498	-0.01006	0.043478	0.858549	10	80489996	0.009726	0.3	10	80489996	0.004455	1.40E-35
rs1078579	C	T	A	C	-0.01379	0.005407	0.434028	0.475985	1	1.07E+08	0.006938	0.44	1	1.07E+08	0.001841	6.97E-14
rs1078632	C	T	G	C	0.019588	-0.00948	0.431375	0.317963	10	97308981	0.007281	0.19	10	97308981	0.001845	2.62E-26
rs1080856	A	G	A	G	0.015275	-0.0118	0.724068	0.435159	8	1.28E+08	0.006907	0.088	8	1.28E+08	0.001995	1.97E-14
rs1082339	A	G	A	G	0.018957	-0.00131	0.27664	0.194765	10	69483278	0.008755	0.88	10	69483278	0.001971	7.18E-22
rs1083173	A	T	G	A	-0.02103	-0.00274	0.846625	0.369356	11	12114765	0.007135	0.7	11	12114765	0.002467	1.63E-17
rs1084902	C	T	C	G	-0.03217	-0.00886	0.265227	0.184678	12	4222843	0.008814	0.31	12	4222843	0.002007	9.09E-58

rs1086436	A	G	A	G	0.013724	0.017261	0.597136	0.151777	1	8858254	0.008815	0.05	1	8858254	0.001817	4.40E-14
rs1088957	A	G	A	G	-0.01272	-0.00965	0.424266	0.787464	1	65683658	0.007711	0.21	1	65683658	0.001811	2.28E-12
rs1091269	A	G	A	G	0.01345	0.003423	0.679635	0.356148	1	1.71E+08	0.007018	0.63	1	1.71E+08	0.001923	2.75E-12
rs1094803	A	G	T	G	-0.01801	-0.00153	0.226686	0.154899	6	42542567	0.009531	0.87	6	42542567	0.002189	2.03E-16
rs1098172	A	C	G	C	-0.01291	0.019124	0.408151	0.068924	9	1.13E+08	0.01303	0.14	9	1.13E+08	0.001821	1.39E-12
rs1099243	A	T	A	G	-0.03078	0.000463	0.058146	0.288425	9	92721221	0.00728	0.95	9	92721221	0.004199	2.40E-13
rs1099547	A	C	G	A	-0.02291	-0.00912	0.442172	0.301393	10	63250912	0.007321	0.21	10	63250912	0.001845	2.26E-35
rs1102713	T	C	G	T	-0.01619	-0.00051	0.268743	0.331172	1	1.73E+08	0.00712	0.94	1	1.73E+08	0.001987	3.93E-16
rs1107251	C	T	T	A	0.016932	-0.00526	0.345277	0.016811	15	74932469	0.026911	0.85	15	74932469	0.001868	1.35E-19
rs1108239	A	G	A	G	0.016761	0.008696	0.389722	0.175793	18	44492751	0.009029	0.34	18	44492751	0.001915	2.26E-18
rs1121262	C	T	C	T	0.014388	0.003884	0.448579	0.43828	11	1.08E+08	0.006784	0.57	11	1.08E+08	0.001764	3.67E-16
rs1124009	G	A	G	A	0.016756	0.009831	0.424257	0.337656	1	1.48E+08	0.007394	0.18	1	1.48E+08	0.001781	5.32E-21
rs1125841	C	T	C	T	-0.02142	0.010685	0.410286	0.334294	10	13494086	0.007227	0.14	10	13494086	0.001801	1.42E-32
rs1126380	C	T	C	T	-0.01305	0.007238	0.402639	0.20365	2	70121038	0.008472	0.39	2	70121038	0.001886	4.79E-12
rs1148947	A	G	A	G	0.019305	-0.00357	0.147402	0.014649	10	88017202	0.02876	0.9	10	88017202	0.002476	6.67E-15
rs1164412	G	A	G	A	-0.01967	0.003247	0.293602	0.799472	16	57025062	0.008367	0.7	16	57025062	0.001931	2.51E-24
rs1164628	A	G	A	G	-0.01723	0.015144	0.183132	0.39001	16	3669343	0.006895	0.028	16	3669343	0.002298	6.77E-14
rs1164866	G	A	G	A	-0.02031	-0.00992	0.264948	0.776657	16	53088574	0.008168	0.22	16	53088574	0.002034	1.85E-23
rs1167071	G	A	T	G	0.461355	0.013702	0.997133	0.088617	19	11808063	0.011875	0.25	19	11808063	0.018138	1.20E-142
rs1167309	T	C	T	C	0.034541	-0.00853	0.042013	0.044909	19	45238836	0.015978	0.59	19	45238836	0.004511	1.98E-14
rs1169520	T	C	T	C	0.01662	-0.02181	0.409791	0.942123	2	1.27E+08	0.014498	0.13	2	1.27E+08	0.001872	7.25E-19
rs1171255	T	C	T	C	0.018617	0.013282	0.208969	0.775216	3	1.41E+08	0.008174	0.1	3	1.41E+08	0.002181	1.45E-17
rs1181210	C	G	A	G	-0.02033	-0.02545	0.195041	0.009846	17	57385975	0.034091	0.46	17	57385975	0.00223	8.36E-20
rs1182183	A	G	A	G	0.042574	-0.00762	0.907878	0.201489	7	2833502	0.008692	0.38	7	2833502	0.003069	1.00E-43
rs1182394	G	T	T	C	-0.01576	0.006382	0.557966	0.276897	11	47701960	0.007724	0.41	11	47701960	0.001781	9.04E-19
rs1192740	A	G	A	G	0.015375	-0.00255	0.197679	0.443804	3	98684125	0.006987	0.72	3	98684125	0.002213	3.85E-12
rs1196064	A	G	T	G	-0.04836	-0.00039	0.256335	0.254323	5	1.49E+08	0.007698	0.96	5	1.49E+08	0.002151	7.60E-112
rs1215624	A	C	A	G	-0.01923	-0.00564	0.295218	0.467579	8	86061448	0.006856	0.41	8	86061448	0.001943	4.78E-23
rs1219604	C	T	G	T	-0.05864	-0.00247	0.894316	0.243276	6	1.21E+08	0.007675	0.75	6	1.21E+08	0.003052	3.13E-82
rs1220674	C	T	G	C	0.015905	-0.01626	0.722099	0.071806	6	52420596	0.012993	0.21	6	52420596	0.001955	4.31E-16
rs1242570	C	T	C	T	-0.01715	0.003841	0.752333	0.278818	12	50216538	0.00763	0.61	12	50216538	0.002057	8.16E-17
rs1245035	A	G	T	G	-0.02004	-0.00295	0.517527	0.32781	12	64582269	0.00734	0.69	12	64582269	0.001783	2.78E-29
rs12453	T	C	T	C	-0.01627	-0.00199	0.499979	0.745917	11	60178272	0.007739	0.8	11	60178272	0.001776	5.61E-20
rs1248126	T	A	C	T	0.014729	-0.00293	0.473819	0.323007	20	8146273	0.007281	0.69	20	8146273	0.001908	1.24E-14
rs1250568	T	C	T	C	0.014385	0.005471	0.503755	0.544428	10	79285523	0.006814	0.42	10	79285523	0.001759	3.09E-16
rs1255061	G	A	G	A	0.028228	-0.00447	0.062875	0.194284	8	23109256	0.008786	0.61	8	23109256	0.003668	1.48E-14
rs1260326	G	A	G	A	-0.11341	0.003182	0.009374	0.538665	2	27508073	0.006816	0.64	2	27508073	0.010279	2.83E-28
rs1263309	C	T	C	T	0.017203	0.003981	0.490945	0.554035	4	3050213	0.006896	0.56	4	3050213	0.001787	6.65E-22
rs1274279	T	C	C	A	-0.01274	0.008119	0.593897	0.027618	1	27061994	0.020331	0.69	1	27061994	0.001801	1.54E-12
rs1275932	A	G	A	G	0.032828	0.007179	0.796308	0.454851	1	41757169	0.006794	0.29	1	41757169	0.002216	1.31E-49
rs1288587	A	G	A	G	0.017922	-0.00613	0.162423	0.181556	14	1.04E+08	0.00885	0.49	14	1.04E+08	0.002417	1.28E-13
rs1290950	T	G	A	G	-0.01425	0.009112	0.668116	0.433237	15	50812153	0.006833	0.18	15	50812153	0.002012	1.46E-12
rs1292076	C	T	C	T	0.017551	-0.00997	0.636468	0.120797	16	1312186	0.010297	0.33	16	1312186	0.001918	5.91E-20

rs1292735	G	C	A	C	0.021764	0.011972	0.56731	0.01585	16	74562720	0.026462	0.65	16	74562720	0.001851	6.93E-32
rs1321309	C	A	T	C	-0.0028	0.000153	0.44346	0.349183	6	36670859	0.007075	0.98	6	36670859	0.001831	0.126647
rs1328909	G	A	A	C	0.030745	0.001031	0.13831	0.085735	9	1.29E+08	0.01227	0.93	9	1.29E+08	0.00256	3.48E-33
rs1329166	G	C	G	A	0.027441	0.037578	0.11172	0.017531	9	282738	0.024433	0.12	9	282738	0.003007	7.65E-20
rs1362622	T	C	T	C	-0.01649	0.005249	0.723805	0.095821	16	49851826	0.011717	0.65	16	49851826	0.002052	9.57E-16
rs1366045	T	C	T	C	-0.0166	0.010964	0.633161	0.341499	3	42867558	0.007362	0.14	3	42867558	0.001943	1.35E-17
rs1375493	A	T	A	G	0.015196	-0.00293	0.430279	0.386167	2	1.81E+08	0.007002	0.68	2	1.81E+08	0.00194	5.02E-15
rs1386451	T	C	C	A	0.046954	-0.00833	0.032234	0.263208	10	67826634	0.007824	0.29	10	67826634	0.005313	1.03E-18
rs1412444	T	A	T	C	0.031072	0.011747	0.26649	0.347022	10	89243170	0.007105	0.098	10	89243170	0.002027	5.47E-53
rs1445171	A	C	A	G	-0.01277	0.02573	0.32388	0.276897	5	1.01E+08	0.007643	0.00076	5	1.01E+08	0.00193	3.89E-11
rs1446707	G	A	G	A	0.013163	-0.00805	0.36386	0.008405	2	2.18E+08	0.03774	0.83	2	2.18E+08	0.001842	9.31E-13
rs1468102	A	C	G	C	0.034007	0.012299	0.325839	0.351345	11	2983296	0.007175	0.086	11	2983296	0.002	9.02E-65
rs1490077	T	C	T	C	-0.01429	0.02181	0.637002	0.101105	7	50330658	0.011449	0.057	7	50330658	0.00193	1.35E-13
rs1684335	G	C	T	A	0.014053	0.002066	0.348547	0.126321	1	1.99E+08	0.010348	0.84	1	1.99E+08	0.001913	2.10E-13
rs1693037	G	A	G	A	0.013358	0.012584	0.195939	0.086455	12	3278531	0.0123	0.31	12	3278531	0.002218	1.77E-09
rs1700589	C	T	C	T	-0.01552	-5.10E-05	0.438304	0.663545	4	82626709	0.007295	0.99	4	82626709	0.001796	5.86E-18
rs1738668	C	G	G	T	-0.02354	-0.02127	0.501502	0.061239	14	35132881	0.013903	0.13	14	35132881	0.001757	6.77E-41
rs1746782	T	C	T	C	-0.02284	-0.00418	0.212491	0.742555	4	71739800	0.007681	0.59	4	71739800	0.002295	2.63E-23
rs1800961	C	G	T	C	-0.00117	-0.00754	0.756915	0.01489	20	44413724	0.027641	0.78	20	44413724	0.00344	0.733369
rs1883587	G	C	T	A	0.057242	0.088895	0.459185	0.005043	12	1.2E+08	0.048578	0.067	12	1.2E+08	0.001802	1.00E-200
rs1894401	C	T	C	T	-0.01436	0.00376	0.731641	0.183958	15	90885812	0.008629	0.66	15	90885812	0.002001	7.56E-13
rs1926168	T	C	T	C	0.013391	-0.04495	0.299603	0.021854	11	1.18E+08	0.022703	0.048	11	1.18E+08	0.001928	3.93E-12
rs1948760	C	T	A	T	0.015422	-0.01283	0.674774	0.011287	5	1.57E+08	0.032665	0.69	5	1.57E+08	0.001912	7.63E-16
rs1966479	C	T	C	T	0.026487	-0.00542	0.090033	0.896494	5	1.19E+08	0.010832	0.62	5	1.19E+08	0.00312	2.18E-17
rs1985157	G	T	G	A	-0.02708	-0.00947	0.093418	0.182277	19	18402784	0.00877	0.28	19	18402784	0.003392	1.50E-15
rs1990760	A	G	A	G	0.040748	-0.00784	0.927283	0.223103	2	1.62E+08	0.007982	0.33	2	1.62E+08	0.003515	4.85E-31
rs1991651	G	A	C	G	0.039918	-0.01422	0.359097	0.273535	8	10848901	0.007654	0.063	8	10848901	0.001874	1.19E-100
rs2012011	T	A	A	G	-0.03181	0.006737	0.051128	0.159702	6	46925618	0.009414	0.47	6	46925618	0.004192	3.39E-14
rs2038700	C	T	C	T	-0.01392	-0.00916	0.549685	0.450528	14	24992783	0.006918	0.19	14	24992783	0.00181	1.56E-14
rs2062250	T	G	C	T	0.069426	0.016001	0.289812	0.091018	15	64379803	0.011195	0.15	15	64379803	0.001976	1.00E-200
rs2072448	T	C	T	C	0.016072	9.81E-05	0.421117	0.605668	12	8058101	0.006936	0.99	12	8058101	0.001795	3.66E-19
rs211440	G	T	C	T	-0.01369	0.007575	0.087028	0.214938	6	1.33E+08	0.008269	0.36	6	1.33E+08	0.002365	7.21E-09
rs211959	C	T	C	T	-0.02474	-0.00244	0.106037	0.504803	21	17578144	0.006686	0.72	21	17578144	0.003161	5.28E-15
rs212409	A	G	A	G	-0.02121	0.007718	0.756889	0.479827	6	1.59E+08	0.00661	0.24	6	1.59E+08	0.002061	8.32E-25
rs2212931	T	C	T	C	-0.018	-0.01434	0.547649	0.837896	21	38487267	0.009161	0.12	21	38487267	0.001908	4.06E-21
rs2219143	T	G	T	C	-0.01907	-0.00504	0.469235	0.370317	9	2622278	0.007119	0.48	9	2622278	0.001762	2.89E-27
rs2240775	T	G	G	A	-0.02586	0.005401	0.135095	0.240154	17	76714046	0.007988	0.5	17	76714046	0.002686	6.36E-22
rs2261003	T	C	A	C	-0.01594	0.009327	0.281648	0.388809	13	1.14E+08	0.007058	0.19	13	1.14E+08	0.001957	3.96E-16
rs2271400	G	C	C	T	0.011636	0.001486	0.682082	0.245677	8	55878685	0.007896	0.85	8	55878685	0.001905	1.04E-09
rs2290846	T	C	T	C	-0.02365	-0.0013	0.075201	0.261527	4	1.5E+08	0.007847	0.87	4	1.5E+08	0.003576	3.89E-11
rs2338823	G	C	T	C	-0.01049	-0.0054	0.458354	0.365514	5	1.42E+08	0.007024	0.44	5	1.42E+08	0.001792	4.97E-09
rs2412771	A	G	A	G	0.026004	0.000635	0.517184	0.570605	4	56895251	0.00683	0.93	4	56895251	0.001782	3.35E-48
rs2421200	T	G	T	G	0.021989	0.004179	0.295409	0.330211	2	61484680	0.00723	0.56	2	61484680	0.001931	5.13E-30

rs2458280	G	A	G	A	-0.01577	-0.00605	0.211419	0.364073	8	1.03E+08	0.007012	0.39	8	1.03E+08	0.002194	6.97E-13
rs2469434	G	A	G	A	0.015716	-0.00672	0.275131	0.350144	18	69876810	0.007244	0.35	18	69876810	0.00204	1.38E-14
rs2475787	T	C	T	C	-0.02333	0.000853	0.20664	0.544188	6	85705518	0.006778	0.9	6	85705518	0.002193	2.04E-26
rs247826	T	C	T	C	0.014113	-0.00359	0.301343	0.173631	16	84549359	0.009145	0.69	16	84549359	0.001945	4.20E-13
rs2482819	T	C	T	C	0.016692	0.006461	0.697022	0.278578	10	30218170	0.007522	0.39	10	30218170	0.002303	4.41E-13
rs2497833	T	G	T	A	0.015532	0.004026	0.61808	0.39097	10	17983041	0.006976	0.56	10	17983041	0.001889	2.12E-16
rs2522051	A	G	A	G	-0.01332	0.019594	0.502337	0.25072	5	1.32E+08	0.007915	0.013	5	1.32E+08	0.001758	3.64E-14
rs2561758	T	C	T	C	-0.0187	-0.04277	0.299754	0.006004	5	1.74E+08	0.044591	0.34	5	1.74E+08	0.001926	3.00E-22
rs257063	C	T	C	T	0.023319	-0.00313	0.391786	0.370077	5	1.15E+08	0.007053	0.66	5	1.15E+08	0.001893	7.79E-35
rs2665405	C	T	C	T	-0.02291	0.002786	0.193448	0.586936	17	59797931	0.006866	0.68	17	59797931	0.002238	1.45E-24
rs2710804	G	A	G	A	0.016766	0.009366	0.447647	0.224304	7	36044919	0.00829	0.26	7	36044919	0.001783	5.56E-21
rs2732625	T	C	T	C	-0.01195	-0.02925	0.379026	0.007925	17	46255063	0.037689	0.44	17	46255063	0.001812	4.36E-11
rs2734031	A	C	A	G	0.02132	-0.01141	0.173155	0.332373	3	1.29E+08	0.007171	0.11	3	1.29E+08	0.002346	1.08E-19
rs2807742	T	C	T	C	-0.01284	-0.00103	0.616525	0.914986	10	28492438	0.012074	0.93	10	28492438	0.001818	1.72E-12
rs2853075	T	C	T	C	0.014036	0.020268	0.221856	0.011768	4	36310920	0.032006	0.53	4	36310920	0.002129	4.51E-11
rs2861524	A	T	C	T	0.014448	-0.00945	0.541795	0.207493	8	54538633	0.008471	0.26	8	54538633	0.001811	1.56E-15
rs2867816	A	G	A	C	-0.02866	-0.00458	0.123048	0.301873	17	43020464	0.007289	0.53	17	43020464	0.002705	3.46E-26
rs2875187	C	T	A	T	0.01549	0.014434	0.405612	0.043948	5	71009726	0.016459	0.38	5	71009726	0.001799	7.62E-18
rs2900156	A	G	A	G	0.003019	-0.01369	0.819349	0.335255	6	32479564	0.007214	0.058	6	32479564	0.002564	0.23902
rs2910686	A	G	A	G	0.020977	-0.00065	0.451223	0.586455	5	96916885	0.006931	0.93	5	96916885	0.001879	6.43E-29
rs2915607	G	A	G	A	0.014639	0.00883	0.223519	0.774015	8	30422400	0.008012	0.27	8	30422400	0.002106	3.75E-12
rs2980869	C	T	C	T	-0.03078	0.001348	0.588603	0.480788	8	1.25E+08	0.006828	0.84	8	1.25E+08	0.001799	1.39E-65
rs303753	C	G	A	G	0.032329	-0.0085	0.254004	0.086215	18	23494958	0.011967	0.48	18	23494958	0.002069	5.55E-55
rs305083	A	G	A	G	-0.02598	-0.00021	0.363379	0.92243	16	85902272	0.012695	0.99	16	85902272	0.001891	6.38E-43
rs325925	T	C	T	G	0.020949	0.009731	0.329652	0.365274	1	1.11E+08	0.007174	0.17	1	1.11E+08	0.00187	4.13E-29
rs328491	G	A	G	A	0.019537	-0.00953	0.587716	0.095341	20	57394436	0.01126	0.4	20	57394436	0.002067	3.53E-21
rs334563	T	C	G	T	-0.01318	0.000718	0.537064	0.429395	3	1.2E+08	0.006866	0.92	3	1.2E+08	0.001856	1.28E-12
rs3395198	G	A	G	A	0.011519	-0.00542	0.727271	0.899616	7	73615107	0.011373	0.63	7	73615107	0.002119	5.58E-08
rs3411241	C	T	C	T	-0.01333	0.00495	0.322135	0.117675	14	69374185	0.010434	0.64	14	69374185	0.001907	2.82E-12
rs3418057	T	C	T	C	-0.0168	0.011366	0.684378	0.041547	1	2.21E+08	0.016939	0.5	1	2.21E+08	0.001953	8.46E-18
rs342242	T	C	T	C	-0.014	0.00805	0.493707	0.748079	7	1.07E+08	0.007785	0.3	7	1.07E+08	0.001983	1.75E-12
rs354707	T	C	T	A	0.037239	-0.0139	0.120884	0.247839	2	1.43E+08	0.007831	0.076	2	1.43E+08	0.002765	2.60E-41
rs3573424	A	G	A	G	-0.0202	0.003648	0.211722	0.550432	4	712911	0.007046	0.6	4	712911	0.002163	1.07E-20
rs3578917	C	T	C	A	0.032447	0.005516	0.415272	0.395773	2	1.02E+08	0.006858	0.42	2	1.02E+08	0.001803	2.20E-72
rs3735485	T	C	T	C	-0.04818	-0.00833	0.027913	0.099424	7	44969742	0.01152	0.47	7	44969742	0.005333	1.76E-19
rs3747869	G	A	T	G	-0.02243	0.004457	0.057626	0.191162	10	71760875	0.008657	0.61	10	71760875	0.003802	3.72E-09
rs3749440	C	T	C	T	0.019927	-0.00433	0.707003	0.4061	3	1.84E+08	0.006958	0.53	3	1.84E+08	0.002029	9.93E-23
rs3760232	G	A	G	A	0.036785	-0.00613	0.043508	0.774736	17	2111555	0.008197	0.45	17	2111555	0.005186	1.37E-12
rs3777755	G	C	T	C	0.001321	0.001674	0.471473	0.229827	6	12159466	0.008042	0.84	6	12159466	0.001799	0.462856
rs3781454	T	C	T	C	-0.01437	0.008515	0.711635	0.668348	10	1.25E+08	0.0072	0.24	10	1.25E+08	0.001961	2.50E-13
rs3795445	T	C	G	T	-0.01421	0.011738	0.620645	0.178915	1	2.27E+08	0.008846	0.18	1	2.27E+08	0.001832	9.42E-15
rs3825761	T	C	T	C	-0.01986	0.002552	0.682661	0.45365	14	1.05E+08	0.006833	0.71	14	1.05E+08	0.001937	1.25E-24
rs385893	T	C	T	C	0.022558	-0.00899	0.368348	0.229827	9	4763176	0.00796	0.26	9	4763176	0.001855	5.41E-34

rs386243	A	G	A	G	0.022892	-0.00853	0.259541	0.665466	19	48624233	0.007063	0.23	19	48624233	0.002237	1.50E-24
rs38859	C	T	C	T	0.02183	-0.0045	0.549433	0.528098	7	1.17E+08	0.006811	0.51	7	1.17E+08	0.001826	6.91E-33
rs3917932	G	A	G	C	0.01612	0.007055	0.556407	0.389529	1	36478315	0.007002	0.31	1	36478315	0.001802	3.98E-19
rs3923776	C	G	C	G	-0.01884	-0.00354	0.406605	0.044669	9	1.36E+08	0.016369	0.83	9	1.36E+08	0.001797	1.09E-25
rs3931	G	C	G	A	0.015762	-0.00464	0.407347	0.259126	2	1.69E+08	0.007782	0.55	2	1.69E+08	0.001845	1.38E-17
rs4006418	G	C	T	C	0.019701	-0.0012	0.281002	0.200288	18	22130372	0.008654	0.89	18	22130372	0.001986	3.72E-23
rs410876	G	A	G	T	0.013989	-0.00427	0.391446	0.373199	9	1.34E+08	0.007229	0.56	9	1.34E+08	0.001804	9.17E-15
rs427044	C	T	C	T	-0.01318	-0.00689	0.451253	0.854227	7	66043558	0.009615	0.47	7	66043558	0.001838	8.01E-13
rs4375747	C	T	C	T	-0.1954	-0.07469	0.994909	0.995677	18	76359827	0.049713	0.13	18	76359827	0.014302	1.87E-42
rs4420029	C	T	C	T	-0.02119	-0.0162	0.27746	0.049232	1	45959828	0.015851	0.31	1	45959828	0.001981	1.14E-26
rs445	A	G	A	G	-0.0227	-0.00889	0.243782	0.332133	7	92779056	0.007238	0.22	7	92779056	0.002072	6.88E-28
rs4660482	C	A	T	C	-0.03958	-0.00503	0.315243	0.260086	1	40892982	0.007824	0.52	1	40892982	0.001952	2.49E-91
rs4707609	A	C	G	A	0.023381	0.004051	0.189166	0.14049	6	90236760	0.00974	0.68	6	90236760	0.00228	1.19E-24
rs4721668	C	T	C	T	0.022706	0.101548	0.541006	0.988713	7	17902783	0.031972	0.0015	7	17902783	0.001816	7.94E-36
rs4734879	C	G	G	A	0.025412	-0.00763	0.709931	0.427474	8	1.06E+08	0.006899	0.27	8	1.06E+08	0.001973	6.54E-38
rs4751697	C	T	C	T	0.013417	0.002819	0.531504	0.377522	10	1.19E+08	0.007067	0.69	10	1.19E+08	0.001771	3.69E-14
rs4757956	G	A	G	A	-0.03341	0.009884	0.727739	0.28146	11	12855119	0.007439	0.18	11	12855119	0.002139	6.16E-55
rs4760	A	T	G	A	-0.04794	0.010138	0.02143	0.016811	19	43648948	0.026562	0.7	19	43648948	0.007111	1.63E-11
rs4761234	T	C	T	C	-0.03172	0.009745	0.233005	0.568924	12	69338325	0.006916	0.16	12	69338325	0.002251	4.93E-45
rs4812447	G	A	G	A	0.013936	0.006796	0.178084	0.439001	20	40643980	0.006893	0.32	20	40643980	0.002373	4.40E-09
rs4843080	T	C	T	C	0.030045	0.009882	0.169334	0.583333	15	85603872	0.007086	0.16	15	85603872	0.00252	1.00E-32
rs4844622	T	G	A	G	-0.0134	0.054889	0.656565	0.014169	1	2.08E+08	0.027818	0.048	1	2.08E+08	0.001908	2.21E-12
rs4903580	C	T	C	T	-0.01505	0.006348	0.413598	0.899616	14	77384635	0.011079	0.57	14	77384635	0.001815	1.17E-16
rs4917628	A	G	A	G	-0.02646	0.011651	0.199223	0.230307	10	1.12E+08	0.008191	0.15	10	1.12E+08	0.002224	1.30E-32
rs4970764	T	C	G	C	-0.04769	0.005981	0.033652	0.422911	1	1.09E+08	0.006862	0.38	1	1.09E+08	0.004904	2.53E-22
rs547364	A	G	G	C	0.014138	-0.01074	0.802686	0.498559	1	1.08E+08	0.006845	0.12	1	1.08E+08	0.002209	1.60E-10
rs551125	T	A	C	T	0.024618	0.004998	0.782567	0.166667	12	1.21E+08	0.009088	0.58	12	1.21E+08	0.002162	5.37E-30
rs5572910	A	G	A	G	0.014763	0.003237	0.636898	0.809318	2	2.32E+08	0.008566	0.71	2	2.32E+08	0.001827	6.79E-16
rs5608240	G	A	G	A	0.033833	0.004333	0.168666	0.660183	3	1.57E+08	0.007139	0.54	3	1.57E+08	0.002397	3.35E-45
rs5617956	C	T	C	T	0.159817	0.010115	0.002635	0.605427	7	1.3E+08	0.006995	0.15	7	1.3E+08	0.022367	9.36E-13
rs5618886	G	C	C	T	-0.02291	-0.00416	0.223998	0.382565	1	2.47E+08	0.006971	0.55	1	2.47E+08	0.002151	1.84E-26
rs5636531	G	T	C	T	-0.01378	0.007979	0.544809	0.263208	2	1.28E+08	0.007713	0.3	2	1.28E+08	0.001767	6.60E-15
rs5638817	C	T	A	C	0.013865	-0.00633	0.550276	0.184918	7	28684757	0.008702	0.47	7	28684757	0.00187	1.28E-13
rs5640811	C	A	G	A	0.01566	0.003827	0.626156	0.126801	19	19682736	0.010273	0.71	19	19682736	0.002011	7.29E-15
rs5692122	A	G	A	G	0.015628	0.010021	0.555076	0.148175	4	53485997	0.009452	0.29	4	53485997	0.001892	1.56E-16
rs571497	T	C	T	C	0.546141	-0.03028	0.976761	0.009606	19	7762944	0.035329	0.39	19	7762944	0.012303	1.00E-200
rs5746451	A	G	A	G	-0.02414	0.004407	0.449277	0.180596	22	17643254	0.008861	0.62	22	17643254	0.001817	3.12E-40
rs5763096	T	C	T	C	-0.03865	-0.00554	0.582725	0.43708	22	29247432	0.00694	0.42	22	29247432	0.00184	6.92E-98
rs5988287	G	A	G	A	0.015492	0.005725	0.376575	0.746398	7	76009103	0.007999	0.47	7	76009103	0.001856	7.36E-17
rs618145	G	C	A	C	-0.013	0.011111	0.649558	0.079011	9	76704644	0.012365	0.37	9	76704644	0.001905	9.14E-12
rs6188983	C	T	C	T	-0.03073	-0.00703	0.042119	0.98391	11	10281374	0.026753	0.79	11	10281374	0.004691	5.91E-11
rs619450	G	T	A	G	-0.01813	-0.00707	0.518771	0.225024	20	2914144	0.008242	0.39	20	2914144	0.001969	3.61E-20
rs6229295	T	C	T	A	0.018023	-0.00379	0.485318	0.14121	3	1.32E+08	0.010044	0.71	3	1.32E+08	0.001821	4.64E-23

rs6251850	A	G	A	G	0.022025	0.002235	0.533263	0.209414	8	78260489	0.008326	0.79	8	78260489	0.001775	2.53E-35
rs643177	G	A	G	A	0.014556	0.005172	0.246309	0.883285	6	1.38E+08	0.010718	0.63	6	1.38E+08	0.002054	1.43E-12
rs6432335	C	T	A	C	0.01575	0.006282	0.335405	0.182997	2	12788845	0.008608	0.47	2	12788845	0.001867	3.41E-17
rs6442061	C	T	C	A	-0.01889	0.004189	0.185112	0.330451	3	47162293	0.007377	0.57	3	47162293	0.002561	1.71E-13
rs6510834	G	C	T	C	0.076409	-0.01567	0.989933	0.225504	19	5008662	0.008254	0.058	19	5008662	0.009741	4.57E-15
rs6651495	G	A	G	A	-0.01572	0.005143	0.273388	0.591739	5	69315756	0.006951	0.46	5	69315756	0.00211	9.67E-14
rs665723	G	C	T	C	0.018947	-0.00334	0.458171	0.114073	6	7138831	0.010452	0.75	6	7138831	0.00176	5.40E-27
rs6674304	C	G	C	T	-0.01676	0.000441	0.52083	0.114553	1	1.16E+08	0.010551	0.97	1	1.16E+08	0.001791	8.66E-21
rs6690335	T	A	G	A	0.010367	0.007126	0.495688	0.383766	1	38826036	0.00702	0.31	1	38826036	0.001774	5.21E-09
rs6714557	T	C	T	C	-0.02579	0.002998	0.323156	0.225504	5	1.51E+08	0.008208	0.71	5	1.51E+08	0.002083	3.59E-35
rs6717590	A	G	A	G	0.023086	0.008532	0.108547	0.197166	15	1.01E+08	0.008699	0.33	15	1.01E+08	0.00283	3.60E-16
rs6734238	G	A	G	A	0.036704	-0.00838	0.067113	0.103987	2	1.13E+08	0.010904	0.44	2	1.13E+08	0.00354	3.69E-25
rs6734347	A	C	A	C	0.058058	0.010439	0.029522	0.033381	7	7979532	0.019438	0.59	7	7979532	0.005437	1.39E-26
rs6779340	T	C	C	G	-0.04022	-0.0093	0.032142	0.035783	3	58047974	0.017195	0.59	3	58047974	0.00537	7.27E-14
rs6859727	T	C	T	C	-0.03754	0.023217	0.065119	0.039625	5	72446795	0.017612	0.19	5	72446795	0.003831	1.21E-22
rs6905891	G	A	G	A	-0.03551	0.017989	0.044393	0.866475	6	43791452	0.010009	0.072	6	43791452	0.004846	2.42E-13
rs6985508	A	G	A	G	0.048573	0.014968	0.065789	0.457733	8	1.41E+08	0.006742	0.026	8	1.41E+08	0.003549	1.38E-42
rs704984	T	C	G	T	-0.01417	0.012594	0.296734	0.177474	1	1.46E+08	0.009027	0.16	1	1.46E+08	0.002065	7.11E-12
rs7210990	T	C	T	G	-0.02207	0.013484	0.693963	0.116955	17	16267450	0.010537	0.2	17	16267450	0.001929	2.93E-30
rs7217986	T	C	T	C	-0.03801	-0.02563	0.193304	0.946206	17	77373496	0.014355	0.074	17	77373496	0.002233	6.57E-65
rs7272605	T	C	T	C	-0.02004	-0.00454	0.109516	0.104947	15	41978076	0.011003	0.68	15	41978076	0.002813	1.09E-12
rs7275491	T	G	A	G	-0.02238	0.000171	0.171532	0.207733	9	1.1E+08	0.008431	0.98	9	1.1E+08	0.002502	4.04E-19
rs7275928	C	T	C	T	-0.05243	0.001157	0.874243	0.180596	9	1.24E+08	0.008875	0.9	9	1.24E+08	0.002769	6.36E-80
rs7279086	T	C	T	C	0.014685	0.004525	0.444088	0.912584	10	44384812	0.012174	0.71	10	44384812	0.001783	1.87E-16
rs7280331	A	G	A	G	-0.02909	0.004634	0.8186	0.0622	16	89300741	0.013978	0.74	16	89300741	0.002329	9.04E-36
rs738408	C	A	A	G	0.063318	0.00364	0.983351	0.352546	22	43928850	0.007073	0.61	22	43928850	0.010425	1.29E-09
rs7426631	C	G	G	T	-0.02947	-0.00032	0.177471	0.174352	2	1.36E+08	0.008795	0.97	2	1.36E+08	0.002462	5.68E-33
rs7462171	A	C	T	C	0.015969	-0.00184	0.445235	0.119837	7	1.38E+08	0.010397	0.86	7	1.38E+08	0.001857	8.64E-18
rs7496362	T	C	G	C	0.013419	0.001298	0.283854	0.23487	15	65466536	0.007945	0.87	15	65466536	0.00195	6.09E-12
rs749780	A	T	C	A	-0.01699	0.0089	0.753765	0.452209	17	74703245	0.006907	0.2	17	74703245	0.002319	2.49E-13
rs7536608	C	T	T	G	-0.0287	-0.00618	0.592435	0.026657	1	2.24E+08	0.021211	0.77	1	2.24E+08	0.001907	3.79E-51
rs7537229	C	T	C	T	-0.02773	-0.02581	0.469695	0.044428	1	56440602	0.016861	0.13	1	56440602	0.001761	7.68E-56
rs7547562	C	T	C	G	-0.02338	0.007669	0.675233	0.3439	2	54560455	0.007029	0.28	2	54560455	0.001938	1.81E-33
rs7572922	G	A	G	A	0.022615	0.010379	0.658793	0.789625	2	65425022	0.00815	0.2	2	65425022	0.001889	5.36E-33
rs7626444	G	A	G	C	0.016596	-0.007	0.123403	0.251441	3	1.97E+08	0.008011	0.38	3	1.97E+08	0.00268	6.12E-10
rs7679673	C	T	G	T	0.013008	-0.00413	0.421299	0.211335	4	1.05E+08	0.008279	0.62	4	1.05E+08	0.001932	1.71E-11
rs7724386	A	G	C	A	-0.03669	-0.00535	0.157187	0.450288	5	1.77E+08	0.006848	0.43	5	1.77E+08	0.002622	1.98E-44
rs7776857	C	T	C	A	0.021973	0.011041	0.063137	0.016571	7	22715149	0.025282	0.66	7	22715149	0.003658	1.95E-09
rs7781509	A	C	T	C	-0.08747	0.010302	0.013295	0.325648	7	47325075	0.007254	0.16	7	47325075	0.008263	3.71E-26
rs7803075	A	G	A	G	-0.0123	0.014918	0.586998	0.869356	7	1.31E+08	0.009942	0.13	7	1.31E+08	0.001833	2.01E-11
rs7807030	G	A	G	T	0.016578	-0.00572	0.63433	0.038905	2	43628011	0.017638	0.75	2	43628011	0.001898	2.55E-18
rs7817850	T	A	G	A	0.060858	0.007444	0.192342	0.071326	17	19984410	0.013208	0.57	17	19984410	0.002239	1.21E-162
rs7846314	T	C	A	T	-0.0143	-0.00661	0.546042	0.136407	8	60738272	0.009799	0.5	8	60738272	0.001834	6.62E-15

rs7873858	A	G	A	G	-0.02103	-0.02163	0.135031	0.036023	13	42269494	0.017993	0.23	13	42269494	0.002573	3.16E-16
rs789858	A	C	A	G	-0.03546	-0.00155	0.422238	0.244476	3	1.95E+08	0.007886	0.84	3	1.95E+08	0.001844	2.41E-82
rs796007	A	G	A	G	0.014661	0.002016	0.242227	0.267291	9	83962626	0.007817	0.8	9	83962626	0.002083	2.02E-12
rs8004096	T	C	C	A	0.01253	-0.01215	0.451298	0.404419	14	81234738	0.007001	0.083	14	81234738	0.001776	1.77E-12
rs8013915	G	T	G	A	-0.02156	0.007711	0.564288	0.014409	20	10746012	0.029002	0.79	20	10746012	0.001955	3.02E-28
rs8020004	A	G	A	G	0.01269	0.000972	0.577901	0.159222	1	42958261	0.009421	0.92	1	42958261	0.001892	2.05E-11
rs878664	A	G	A	G	-0.01897	-0.07394	0.257495	0.005283	18	62447188	0.047428	0.12	18	62447188	0.002011	4.27E-21
rs9131	A	C	A	G	0.015797	-0.00568	0.690295	0.316763	4	74097332	0.007246	0.43	4	74097332	0.00197	1.11E-15
rs915125	A	G	A	G	0.02478	0.00613	0.912761	0.334054	6	81753659	0.007346	0.4	6	81753659	0.00334	1.22E-13
rs9287604	A	G	C	G	-0.01713	0.010002	0.563703	0.309318	2	2.37E+08	0.007437	0.18	2	2.37E+08	0.001859	3.34E-20
rs9390460	A	G	A	G	-0.02263	0.000239	0.173173	0.693564	6	1.47E+08	0.007319	0.97	6	1.47E+08	0.00235	6.32E-22
rs9394283	A	G	T	A	-0.0044	0.005804	0.397436	0.310759	6	35221859	0.007352	0.43	6	35221859	0.001836	0.01653
rs9402685	T	C	T	C	0.022336	-0.00369	0.187163	0.793708	6	1.35E+08	0.008388	0.66	6	1.35E+08	0.002285	1.55E-22
rs9749453	T	G	C	T	-0.09681	-0.00324	0.979626	0.236071	19	11276185	0.008123	0.69	19	11276185	0.00704	5.60E-43
rs9773718	C	T	C	T	0.014759	-0.00372	0.432619	0.814121	9	1.33E+08	0.008722	0.67	9	1.33E+08	0.001817	4.78E-16
rs9804265	G	C	C	A	-0.01524	0.000427	0.290385	0.045389	10	24925121	0.016322	0.98	10	24925121	0.002	2.63E-14
rs9905106	C	T	C	T	0.02018	-0.00899	0.761459	0.46878	17	1470224	0.006682	0.18	17	1470224	0.00208	3.10E-22
rs9970896	C	T	T	A	0.018342	-0.01319	0.171157	0.025696	1	2.36E+08	0.021555	0.54	1	2.36E+08	0.00238	1.35E-14
rs9977672	C	T	C	T	0.022798	-0.00674	0.622184	0.832133	21	39091357	0.009079	0.46	21	39091357	0.001853	9.37E-35

Supplementary Table 2n. List of SNPs for Vitamin D and their effects on TEWL

SNP	effect_alle	other_alle	effect_alle	other_alle	beta.VitD	beta.TEWL	eaf.VitD	eaf.TEWL	chr.TEWL	pos.TEWL	se.TEWL	pval.TEWL	chr.VitD	pos.VitD	se.VitD	pval.VitD
chr1:1550	G	T	G	T	0.016904	0.026789	0.733745	0.928194	1	1.55E+08	0.013223	0.043	1	154994978	0.00229	1.57E-13
chr1:2207	A	G	A	G	0.014498	-0.00336	0.315487	0.226465	1	2.21E+08	0.00806	0.68	1	220972343	0.002152	1.60E-11
chr1:2301	C	T	C	T	-0.01747	-0.00091	0.199128	0.688761	1	2.3E+08	0.007285	0.9	1	230303512	0.002509	3.37E-12
chr1:4137	C	T	C	T	0.013438	-0.00461	0.433767	0.615034	1	41370013	0.00701	0.51	1	41835685	0.002025	3.25E-11
chr12:975	G	T	G	T	0.022014	0.005789	0.929281	0.830451	12	97588923	0.009219	0.53	12	97982701	0.00388	1.40E-08
chr14:100	G	C	G	C	-0.01175	0.005567	0.326561	0.947406	14	1.01E+08	0.014032	0.69	14	101176212	0.002132	3.52E-08
chr14:390	G	C	G	C	0.037944	0.008267	0.176673	0.336695	14	39086981	0.00739	0.26	14	39556185	0.00261	6.84E-48
chr15:583	T	C	T	C	0.028378	-0.00075	0.644772	0.580692	15	58387979	0.006845	0.91	15	58680178	0.002087	4.14E-42
chr15:584	C	T	C	T	0.033844	-0.01157	0.784797	0.646254	15	58431476	0.007076	0.1	15	58723675	0.002423	2.54E-44
chr16:118	T	TG	G	A	-0.01371	0.006785	0.726827	0.384246	16	11814919	0.00698	0.33	16	11906659	0.002253	1.15E-09
chr16:569	C	T	C	T	0.013859	-0.00043	0.676894	0.836936	16	56960616	0.009319	0.96	16	56994528	0.002246	6.85E-10
chr18:496	T	A	T	A	0.019288	0.000734	0.154667	0.245917	18	49617853	0.007841	0.93	18	47144223	0.002778	3.85E-12
chr19:358	C	T	C	T	0.012475	0.002104	0.688405	0.458934	19	35851310	0.006743	0.75	19	36342212	0.00215	6.57E-09
chr19:449	A	G	A	G	0.015846	-0.01093	0.384386	0.573487	19	44918620	0.006787	0.11	19	45421877	0.002098	4.29E-14
chr2:6293	G	A	G	A	0.011447	-0.00146	0.471877	0.434198	2	62939244	0.007006	0.84	2	63166379	0.001996	9.81E-09
chr4:9931	T	C	T	C	0.046667	-0.00391	0.024889	0.665466	4	99318162	0.006684	0.56	4	100239319	0.006374	2.45E-13
chr5:8864	T	C	T	C	-0.01321	-0.00461	0.290469	0.680355	5	88644208	0.007124	0.52	5	87940026	0.002198	1.82E-09
chr6:1316	G	A	G	A	0.023362	-0.00183	0.834778	0.676033	6	1.32E+08	0.007337	0.8	6	131924689	0.002683	3.07E-18
chr6:6079	C	T	C	T	-0.01176	5.56E-05	0.488637	0.563881	6	60799829	0.006885	0.99	6	57767576	0.002001	4.21E-09
chr7:1049	C	T	C	T	0.011683	-0.00089	0.537368	0.128482	7	1.05E+08	0.009371	0.92	7	104618318	0.002038	9.85E-09
chr8:1175	A	G	A	G	-0.01489	-0.00658	0.416395	0.029059	8	11754356	0.019714	0.74	8	11611865	0.002036	2.63E-13
chr8:2603	G	A	G	A	-0.01495	0.012938	0.747064	0.666186	8	26035403	0.007165	0.071	8	25892919	0.0023	8.14E-11
chr8:5848	A	G	A	G	-0.01231	-0.00838	0.334478	0.244236	8	58480714	0.007882	0.29	8	59393273	0.002115	5.91E-09
rs1008588	T	C	T	C	0.014558	0.010177	0.717815	0.918588	7	21538342	0.012378	0.41	7	21577960	0.002238	7.85E-11
rs1038165	C	T	C	T	-0.01206	-0.00087	0.416651	0.486071	12	68272160	0.006758	0.9	12	68665940	0.002018	2.31E-09
rs1040196	A	AG	G	A	-0.04179	0.003581	0.926847	0.088857	19	19296909	0.012039	0.77	19	19393890	0.003833	1.13E-27
rs10426	G	A	G	A	-0.0252	-0.01654	0.786567	0.849664	19	51014542	0.009714	0.089	19	51517798	0.002431	3.53E-25
rs1045408	C	T	C	T	0.013531	0.000217	0.715178	0.566523	17	42583623	0.006928	0.97	17	40735641	0.002207	8.71E-10
rs1047891	C	A	C	A	0.015214	0.005686	0.684179	0.834054	2	2.11E+08	0.009281	0.54	2	211540507	0.002141	1.18E-12
rs1085999	T	C	T	C	0.043773	-0.00765	0.417366	0.527858	12	95981904	0.006862	0.26	12	96375682	0.002082	3.76E-98
rs1088771	C	T	C	T	0.01117	0.008481	0.471787	0.055235	10	80282868	0.014727	0.56	10	82042624	0.001998	2.28E-08
rs1090841	G	A	G	A	0.012127	-0.00366	0.510067	0.467339	1	1.55E+08	0.00677	0.59	1	154567699	0.001994	1.18E-09
rs1090846	C	T	C	T	-0.0201	0.010491	0.732668	0.352305	1	1.55E+08	0.007015	0.13	1	155389688	0.002292	1.79E-18
rs1107617	A	G	A	G	-0.0177	0.005412	0.821642	0.864793	16	56972466	0.010159	0.59	16	57006378	0.002748	1.17E-10
rs1110836	A	G	A	G	-0.01442	-0.01038	0.393855	0.500961	12	95992360	0.006923	0.13	12	96386138	0.002154	2.15E-11
rs1112718	T	C	T	C	-0.01136	0.002453	0.504205	0.661623	2	28658541	0.007159	0.73	2	28881407	0.002035	2.35E-08
rs1118242	T	C	T	C	0.012535	-0.0025	0.480005	0.819164	12	38132585	0.008855	0.78	12	38526387	0.001994	3.24E-10
rs1126432	G	A	G	A	0.013028	0.012939	0.570235	0.162824	1	1.55E+08	0.009267	0.16	1	155087933	0.002049	2.03E-10
rs1149605	T	C	T	C	-0.02308	-0.01534	0.829603	0.643132	11	76774172	0.007059	0.03	11	76485216	0.002662	4.28E-18
rs1154246	G	A	G	A	0.023866	-0.01536	0.865656	0.982229	16	82000205	0.025183	0.54	16	82033810	0.002919	2.90E-16
rs11606	C	G	C	G	-0.01227	0.000287	0.574838	0.785062	19	54154365	0.008249	0.97	19	54658102	0.002051	2.21E-09
rs1173289	G	A	G	A	0.016005	0.006897	0.701209	0.691402	4	87366841	0.00741	0.35	4	88287993	0.002174	1.79E-13

rs1205676	T	G	T	G	0.025414	0.005599	0.417091	0.353506	8	1.16E+08	0.007006	0.42	8	116988527	0.002048	2.28E-35
rs1231726	A	G	A	G	0.020897	-0.00524	0.848996	0.567723	12	21199607	0.00684	0.44	12	21352541	0.002785	6.21E-14
rs1260326	T	C	T	C	-0.02088	-0.00318	0.393435	0.461335	2	27508073	0.006816	0.64	2	27730940	0.002038	1.24E-24
rs1279471	G	A	G	A	0.112807	0.004545	0.578197	0.629443	11	14892029	0.007061	0.52	11	14913575	0.002056	1.00E-200
rs1280325	A	G	A	G	-0.06733	0.015547	0.223268	0.652978	11	71421822	0.007174	0.03	11	71132868	0.003899	8.13E-67
rs1310426	G	A	G	A	-0.02019	-0.01378	0.743062	0.992075	4	69482372	0.036665	0.71	4	70348090	0.002387	2.70E-17
rs1328405	T	C	T	C	-0.01757	-0.00309	0.882273	0.933718	9	1.05E+08	0.013816	0.82	9	107669073	0.003134	2.08E-08
rs1352846	A	G	A	G	0.195884	-0.00508	0.708567	0.751921	4	71752058	0.007772	0.51	4	72617775	0.002322	1.00E-200
rs1421589	G	A	G	A	-0.02549	-0.04087	0.885392	0.982229	19	11079858	0.026209	0.12	19	11190534	0.003146	5.43E-16
rs1660839	G	A	G	A	-0.0151	0.004231	0.751151	0.657061	11	71383186	0.007238	0.56	11	71094232	0.002336	1.03E-10
rs1721670	T	C	T	C	0.032453	0.033267	0.817316	0.945965	20	54115823	0.014906	0.026	20	52732362	0.00272	8.30E-33
rs1933064	G	A	G	A	-0.01512	-0.0106	0.53039	0.15586	1	1.52E+08	0.009327	0.26	1	152301576	0.002106	6.93E-13
rs2012736	C	A	C	A	0.048307	0.002059	0.919186	0.758405	2	2.34E+08	0.008084	0.8	2	234622379	0.003666	1.21E-39
rs2037511	G	A	G	A	-0.01808	-0.00593	0.833993	0.888088	18	63698973	0.010959	0.59	18	61366207	0.00268	1.52E-11
rs2074735	G	C	G	C	-0.02773	-0.01023	0.935904	0.627041	22	31139886	0.007094	0.15	22	31535872	0.004071	9.62E-12
rs212100	T	C	T	C	0.065857	-0.03168	0.164001	0.010567	19	47873738	0.033111	0.34	19	48376995	0.002692	3.83E-132
rs2131925	G	T	G	T	0.022894	0.008814	0.356375	0.2805	1	62560271	0.007588	0.25	1	63025942	0.002085	4.71E-28
rs2229742	G	C	G	C	0.025148	0.016608	0.896549	0.991114	21	14966851	0.036709	0.65	21	16339172	0.003271	1.49E-14
rs2346264	A	C	A	C	0.013883	-0.01359	0.217315	0.057397	7	1.34E+08	0.014812	0.36	7	133536351	0.002436	1.21E-08
rs2585442	C	G	C	G	-0.02663	0.011977	0.759346	0.876801	20	54120584	0.010474	0.25	20	52737123	0.002438	8.95E-28
rs2725371	A	G	A	G	-0.01197	0.008406	0.302309	0.066523	8	30996517	0.012631	0.51	8	30854033	0.002183	4.17E-08
rs2762943	T	G	T	G	-0.04535	-0.01454	0.076929	0.017531	20	54174247	0.026377	0.58	20	52790786	0.003775	3.07E-33
rs2837596	A	AT	G	A	0.034618	0.020248	0.52465	0.293708	4	69098070	0.007511	0.007	4	69968825	0.002058	1.71E-63
rs2840795	C	T	C	T	0.013428	-0.00475	0.756438	0.658021	6	32658571	0.007166	0.51	6	32623367	0.002325	7.69E-09
rs2847500	G	A	G	A	0.022158	-0.00847	0.876497	0.884006	11	1.2E+08	0.010637	0.43	11	120114421	0.003028	2.51E-13
rs2952289	C	T	C	T	-0.01772	0.001089	0.201968	0.472863	17	68468273	0.006774	0.87	17	66464414	0.002492	1.18E-12
rs31612	T	C	T	C	0.014528	-0.00351	0.825562	0.370797	5	1.1E+08	0.006993	0.62	5	108996643	0.002649	4.16E-08
rs325384	C	T	C	T	0.014173	0.001626	0.715795	0.71806	15	99689556	0.007684	0.83	15	100229761	0.002218	1.66E-10
rs3429076	C	G	C	G	0.038727	0.036401	0.970888	0.991835	8	9327669	0.038374	0.34	8	9185179	0.005976	9.16E-11
rs3505790	A	T	A	T	-0.01597	-0.00382	0.568691	0.872238	4	68506364	0.010229	0.71	4	69372082	0.002096	2.62E-14
rs3540843	C	T	C	T	0.021388	0.005484	0.657806	0.742075	1	17233700	0.007765	0.48	1	17560195	0.0021	2.34E-24
rs3849374	G	C	G	C	0.016102	0.006347	0.821971	0.404659	2	1.01E+08	0.006965	0.36	2	101443397	0.002616	7.47E-10
rs3925446	G	A	G	A	-0.0158	-0.00251	0.800871	0.653458	10	89735565	0.007165	0.73	10	91495322	0.002497	2.50E-10
rs4327060	C	T	C	T	0.024675	-0.01108	0.945604	0.588136	16	72773539	0.006881	0.11	16	72807438	0.004393	1.94E-08
rs4364259	G	A	G	A	-0.01591	0.013089	0.797852	0.896974	4	15890536	0.011261	0.25	4	15892159	0.002506	2.16E-10
rs4418728	G	T	G	T	-0.0112	-0.00033	0.548312	0.182517	10	93079967	0.008723	0.97	10	94839724	0.002	2.17E-08
rs4575545	G	A	G	A	0.015891	-0.02025	0.695172	0.731268	16	79721549	0.007646	0.0081	16	79755446	0.002174	2.65E-13
rs4616820	C	T	C	T	0.012286	-0.0034	0.535046	0.465178	4	56879315	0.006828	0.62	4	57745481	0.002018	1.13E-09
rs541041	G	A	G	A	0.01623	-0.03823	0.180761	0.01513	2	21072103	0.027456	0.16	2	21294975	0.002594	3.93E-10
rs5582999	T	C	T	C	0.0188	-0.00699	0.655996	0.972382	15	63498443	0.018949	0.71	15	63790642	0.002103	3.93E-19
rs590215	C	T	C	T	0.012876	-0.00883	0.734073	0.818444	18	60236855	0.008895	0.32	18	57904088	0.002258	1.17E-08
rs6003456	T	A	T	A	0.013214	-9.75E-05	0.765336	0.967819	22	23013933	0.018396	1	22	23356100	0.002364	2.27E-08
rs6123359	A	G	A	G	-0.02506	0.002784	0.897775	0.48511	20	54098167	0.006692	0.68	20	52714706	0.003344	6.72E-14
rs613808	A	G	A	G	-0.01566	-0.00128	0.280007	0.726945	11	1.17E+08	0.007687	0.87	11	116710968	0.00245	1.63E-10

rs6189138	T	G	T	G	-0.013	-0.0039	0.544079	0.829971	11	66312347	0.009007	0.67	11	66079818	0.002008	9.72E-11
rs6200729	G	A	G	A	0.013341	-0.00738	0.287463	0.628963	15	77419377	0.007126	0.3	15	77711719	0.0022	1.33E-09
rs6550617	A	ATTTC	T	C	0.012408	0.009179	0.28438	0.497598	3	18736344	0.00684	0.18	3	18794313	0.002216	2.14E-08
rs6671730	G	A	G	A	0.014788	-0.00669	0.565714	0.722863	1	2407700	0.007565	0.38	1	2339139	0.002011	1.92E-13
rs6782190	G	A	G	A	0.018046	0.004798	0.352488	0.099904	3	85590522	0.010892	0.66	3	85639672	0.002089	5.74E-18
rs705117	C	T	C	T	-0.03191	-0.00383	0.1477	0.474304	4	71742398	0.006787	0.57	4	72608115	0.002923	9.62E-28
rs7149014	T	C	T	C	0.013167	-0.0067	0.370807	0.565802	14	29333705	0.006871	0.33	14	29802911	0.002086	2.77E-10
rs727857	G	A	G	A	0.01401	-0.00827	0.388511	0.576369	2	58754832	0.006863	0.23	2	58981967	0.002062	1.08E-11
rs7299762	C	A	C	A	-0.02793	0.008828	0.915338	0.784822	11	75777009	0.008481	0.3	11	75488054	0.003582	6.39E-15
rs7341359	T	C	T	C	-0.0217	-0.0154	0.926146	0.953891	12	1.11E+08	0.016502	0.35	12	111582630	0.003826	1.41E-08
rs7412	C	T	C	T	-0.03033	0.012884	0.917927	0.918828	19	44908822	0.012548	0.3	19	45412079	0.003636	7.44E-17
rs7528419	A	G	A	G	-0.01974	0.003734	0.775329	0.914745	1	1.09E+08	0.01203	0.76	1	109817192	0.002387	1.36E-16
rs7569755	G	A	G	A	-0.01425	0.010088	0.70942	0.833573	2	1.18E+08	0.008959	0.26	2	118648261	0.002212	1.18E-10
rs7574138	C	G	C	G	0.016153	-7.03E-05	0.852362	0.614553	7	1.01E+08	0.00692	0.99	7	100809458	0.002826	1.09E-08
rs7604788	C	T	C	T	-0.0362	0.02464	0.966567	0.991595	2	20967152	0.036715	0.5	2	21190024	0.005567	7.88E-11
rs7640441	T	TG	T	G	-0.01384	0.000333	0.727909	0.216859	3	1.25E+08	0.008387	0.97	3	125125159	0.002268	1.04E-09
rs7753286	C	T	C	T	-0.02701	0.017905	0.945958	0.981748	10	86321681	0.025547	0.48	10	88081438	0.004402	8.49E-10
rs7784802	A	T	A	T	-0.01381	0.007011	0.639009	0.82781	7	64555001	0.00912	0.44	7	64015379	0.002072	2.63E-11
rs7792461	G	A	G	A	0.016641	-0.00901	0.806515	0.85951	16	20381010	0.010221	0.38	16	20392332	0.002552	6.96E-11
rs7815119	A	C	A	C	0.016696	-0.01913	0.871284	0.976705	6	25618779	0.022772	0.4	6	25619007	0.002974	1.98E-08
rs7864991	T	A	T	A	0.021195	0.008628	0.893821	0.784582	4	3480486	0.008482	0.31	4	3482213	0.003252	7.17E-11
rs8091117	C	A	C	A	0.026363	0.001136	0.934702	0.926753	18	31339831	0.013264	0.93	18	28919794	0.004029	6.00E-11
rs8113404	C	T	C	T	-0.0127	-0.0141	0.695414	0.939241	19	52562326	0.014226	0.32	19	53065579	0.002172	4.92E-09
rs9490317	T	C	T	C	-0.01105	-0.00122	0.554106	0.615514	6	1.22E+08	0.006994	0.86	6	121859499	0.002012	3.96E-08
rs964184	G	C	G	C	-0.03495	0.008342	0.131624	0.231988	11	1.17E+08	0.008313	0.32	11	116648917	0.003222	2.05E-27
rs9861009	T	C	T	C	-0.01402	-0.00204	0.272485	0.211335	3	1.42E+08	0.008298	0.81	3	141654685	0.002253	4.87E-10
rs9870992	C	T	C	T	0.016694	-0.00094	0.860311	0.985831	3	84318463	0.028276	0.97	3	84440527	0.002879	6.70E-09

Supplementary Table 2o. List of SNPs for Atopic Dermatitis (AD) and their effects on TEWL

SNP	effect_alle	other_allel	effect_alle	other_allel	beta.AD	beta.TEWL	eaf.TEWL	chr.TEWL	pos.TEWL	se.TEWL	pval.TEWL	chr.AD	pos.AD	se.AD	pval.AD
chr11:363!	T	C	T	C	0.072571	0.0142	0.67123	11	36350084	0.007228	0.049	11	36350207	0.032928	8.70E-07
chr11:657!	G	A	G	A	0.113329	0.00575	0.505764	11	65791795	0.006852	0.4	11	65791795	0.027339	2.10E-19
chr16:111:	T	C	T	C	0.083382	-0.0016	0.674832	16	11135732	0.007127	0.82	16	11135732	0.022186	2.50E-11
chr20:636:	A	C	A	C	0.105361	0.01616	0.365034	20	63671762	0.006794	0.017	20	63671762	0.034026	7.00E-13
chr5:1326!	C	T	C	T	0.131028	0.001878	0.17219	5	1.33E+08	0.009043	0.84	5	1.33E+08	0.031476	4.00E-17
rs1019960	G	A	G	A	0.040822	0.015429	0.774736	2	8354967	0.00823	0.061	2	8354967	0.016029	4.00E-08
rs1021423	T	C	T	C	0.061875	-0.00887	0.830211	5	35883632	0.009029	0.33	5	35883632	0.016372	2.90E-14
rs1057258	C	T	C	T	0.061875	-0.00431	0.591258	2	2.33E+08	0.007087	0.54	2	2.33E+08	0.021714	1.70E-10
rs1121114	A	G	A	G	0.061875	-0.01119	0.772334	2	70872975	0.007911	0.16	2	70872975	0.021714	9.40E-09
rs1126561	A	G	A	G	0.076961	0.001614	0.637848	1	1.54E+08	0.006963	0.82	1	1.54E+08	0.014176	6.10E-11
rs2143950	T	C	T	C	0.076961	0.004009	0.361431	14	35103151	0.007008	0.57	14	35103151	0.018899	1.80E-15
rs2212434	T	C	T	C	0.086178	-0.00838	0.430596	11	76570549	0.006805	0.22	11	76570549	0.023301	4.60E-13
rs2227483	T	A	T	A	0.061875	-0.00064	0.476225	12	68254396	0.00681	0.93	12	68254396	0.016198	6.70E-16
rs2918307	G	A	G	A	0.113329	-0.00145	0.117915	19	8679458	0.01071	0.89	19	8679458	0.036459	4.60E-12
rs2944542	G	C	G	C	0.061875	0.00533	0.475024	10	62610240	0.006921	0.44	10	62610240	0.021714	1.20E-06
rs6419573	T	C	T	C	0.10436	0.009158	0.467819	2	1.02E+08	0.006871	0.18	2	1.02E+08	0.027585	1.50E-13
rs6473227	C	A	C	A	0.061875	-0.01212	0.538184	8	80373657	0.006752	0.073	8	80373657	0.010856	2.20E-16
rs6602364	G	C	G	C	0.04879	0.011613	0.319164	10	5996890	0.007301	0.11	10	5996890	0.014648	1.30E-10
rs7127307	T	C	T	C	0.061875	0.002691	0.394092	11	1.28E+08	0.007122	0.71	11	1.28E+08	0.016372	1.50E-18
rs7512552	C	T	C	T	0.040822	0.00067	0.758405	1	1.5E+08	0.008049	0.93	1	1.5E+08	0.016029	5.40E-09
