# Supplementary material for: Investigating causal relationships between obesity and skin barrier function in a multi-ethnic Asian general population cohort
Source: Int J Obes (Lond). 2023 Jul 21;47(10):963–9. doi: 10.1038/s41366-023-01343-z (PMC10511308; doi:10.1038/s41366-023-01343-z)
Supplement: Supplementary file 4 — Supplementary Table 3 [file 41366_2023_1343_MOESM4_ESM.docx]

**Supplementary Table 3. Characteristics of study participants**

| **Characteristics** | **Chinese (n=6913)** | **Malays (n=1279)** | **Indians (n=1798)** | | **Total**  **(n=9990)** | **P-value*** |
| --- | --- | --- | --- | --- | --- | --- |
| **Age (years, mean ± SD)** | 52.6 ± 11.9 | 50.4 ± 11.0 | 52.5 ± 11.4 | 52.3 ± 11.7 | | ***2.57E-09*** |
| **Gender (%)** |  |  |  |  | |  |
| Female | 60.9 | 64.1 | 52.5 | 59.8 | | ***3.23E-12*** |
| **Education level (%)** |  |  |  |  | | ***1.05E-117*** |
| Primary School or no education | 3.5 | 8.3 | 5.0 | 4.4 | |  |
| O levels or ITE | 20.8 | 45.1 | 29.3 | 25.4 | |  |
| A levels or diploma | 25.7 | 27.7 | 23.7 | 25.6 | |  |
| Undergraduate/Graduate | 50.0 | 18.9 | 42.0 | 44.6 | |  |
| **Household income ($) per month** | | | | | | ***3.11E-74*** |
| <2000 | 11.5 | 16.6 | 15.4 | 12.9 | |  |
| 2000 - 3999 | 14.0 | 27.9 | 21.0 | 17.1 | |  |
| 4000 - 5999 | 17.5 | 23.8 | 20.8 | 18.9 | |  |
| 6000 - 9999 | 25.1 | 20.6 | 22.3 | 24.0 | |  |
| >= 10000 | 31.9 | 11.0 | 20.5 | 27.1 | |  |
| **Alcohol Consumption (%)** | | | | | | ***2.65E-64*** |
| Never drink | 29.9 | 92.7 | 49.3 | 41.5 | |  |
| Occasional | 48.4 | 5.2 | 26.3 | 38.7 | |  |
| 1-3x /month | 10.3 | 0.9 | 9.7 | 9.0 | |  |
| 1-2x/week | 6.8 | 0.6 | 9.0 | 6.4 | |  |
| 3-4x/week | 3.0 | 0.5 | 3.9 | 2.9 | |  |
| Almost daily | 1.6 | 0.2 | 1.9 | 1.5 | |  |
| **Smoking (%)** | | | | | | ***1.78E-54*** |
| Never smoke | 75.5 | 60.1 | 69.8 | 72.5 | |  |
| Ex-smoker | 18.5 | 21.4 | 19.4 | 19.1 | |  |
| Current smoker | 5.9 | 18.5 | 10.8 | 8.4 | |  |
| **Atopic dermatitis (%)** | 9.2 | 8.4 | 8.5 | 9.0 | | 4.44E-01 |
| **Diabetes mellitus (%)** | 7.2 | 12.7 | 14.4 | 9.2 | | ***4.94E-24*** |
| **Hba1c (%, mean ± SD)** | 5.59 ± 0.64 | 5.95 ± 1.20 | 6.06 ± 1.23 | 5.73 ± 0.89 | | ***2.61E-108*** |
| **Insulin Resistance (HOMA-IR)** | 2.12 ± 1.77 | 2.94 ± 2.64 | 3.52 ± 2.95 | 2.49 ± 2.24 | | ***2.93E-70*** |
| **Supplementary Table 3** (*continued)* | | | | | | |
| **Characteristics** | **Chinese (n=6913)** | **Malays (n=1279)** | **Indians (n=1798)** | **Total**  **(n=9990)** | | **P-value*** |
| **Hypertension (%)** | 19.9 | 20.7 | 21.4 | 20.3 | | 3.37E-01 |
| **Systolic BP (mmHg)** | 120.2 ± 19.2 | 123.1 ± 19.0 | 122.3 ± 19.1 | 121.0 ± 19.2 | | ***2.01E-08*** |
| **Diastolic BP (mmHg)** | 70.7 ± 11.3 | 70.4 ± 11.2 | 71.3 ± 11.3 | 70.8 ± 11.3 | | 5.50E-02 |
| **Pulse Pressure (mmHg)** | 49.5 ± 14.5 | 52.7 ± 14.1 | 51.0 ± 14.7 | 50.2 ± 14.5 | | ***1.99E-13*** |
| **Heart Rate** | 68.4 ± 11.7 | 69.9 ± 10.7 | 69.7 ± 12.3 | 68.8 ± 11.7 | | **1.76E-07** |
| **Hyperlipidemia (%)** | 36.8 | 34.4 | 36.2 | 36.4 | | 2.81E-01 |
| **LDL(mmol/L)** | 3.10 ± 0.84 | 3.25 ± 0.95 | 3.09 ± 0.86 | 3.12 ± 0.86 | | ***1.21E-07*** |
| **Total cholesterol (mmol/L)** | 5.26 ± 0.96 | 5.31 ± 1.07 | 5.01 ± 0.98 | 5.22 ± 0.86 | | ***3.66E-22*** |
| **BMI (kg m^-2^, mean)** | 23.6 ± 3.8 | 28.3 ± 5.5 | 27.1 ± 4.9 | 24.9 ± 4.7 | | ***6.68E-16*** |
| **Total White Cell (10^9^/L)** | 5.68 ± 1.62 | 6.55 ± 1.62 | 6.86 ± 1.68 | 6.01 ± 1.71 | | ***1.42E-187*** |
| **Neutrophils (10^9^/L)** | 3.28 ± 1.10 | 3.74 ± 1.19 | 3.83 ± 1.21 | 3.44 ± 1.16 | | ***1.02E-93*** |
| **Vitamin D (ng/ml)** | 50.05 ± 15.73 | 39.74 ± 18.88 | 42.97 ± 19.93 | 47.37 ± 17.49 | | ***3.58E-58*** |
| **C-reactive protein (mg/dL)** | 1.59 ± 0.08 | 3.17 ± 0.21 | 3.92 ± 0.20 | 2.29 ± 0.07 | | ***1.36E-41*** |
| **TEWL (mean ± SD)** | 7.02 ± 1.34 | 7.22 ± 1.14 | 7.08 ± 1.25 | 7.06 ± 1.30 | | ***2.73E-07*** |
| **Moist (mean ± SD)** | 28.71 ± 12.90 | 26.02 ± 10.14 | 24.78 ± 10.80 | 27.90 ± 12.34 | | ***2.58E-44*** |
| **pH (mean ± SD)** | 5.21 ± 0.52 | 5.23 ± 0.50 | 5.34 ± 0.51 | 5.23 ± 0.52 | | ***9.62E-21*** |

ITE: Institute of Technical Education; HOMA-IR (Homeostatic Model Assessment for Insulin Resistance); BP: Blood Pressure, LDL: Low-Density Lipoprotein; BMI: Body Mass Index; TEWL: Trans-Epidermal Water Loss

*P-value of Analysis of Variance (ANOVA) test between ethnic groups
