# Supplementary material for: Investigating causal relationships between obesity and skin barrier function in a multi-ethnic Asian general population cohort
Source: Int J Obes (Lond). 2023 Jul 21;47(10):963–9. doi: 10.1038/s41366-023-01343-z (PMC10511308; doi:10.1038/s41366-023-01343-z)
Supplement: Supplementary file 5 — Supplementary Table 4 [file 41366_2023_1343_MOESM5_ESM.docx]

| **Supplementary Table 4. Relationship of skin physiology measures with BMI (kg/m^2^), split by ethnicity of participants** | | | | | | | | |  | | |  |  |
| --- | --- | --- | --- | --- | --- | --- | --- | --- | --- | --- | --- | --- | --- |
|  | **Model 1** | | | **Model 2** | | **Model 3** | | |  |  |  |  |  |
|  | **Beta** | **SE** | **P-value** | **Beta** | **P-value** | | **Beta** | **P-value** | |  | | |  |
| **Chinese** |  |  |  |  |  | |  |  | | |  | | |
| TEWL | 0.335 | 6.46E-02 | ***2.17E-07*** | 0.336 | ***2.00E-06*** | | 0.310 | ***1.57E-02*** | | |  | | |
| Moisture | -0.515 | 0.133 | ***1.07E-04*** | -0.416 | ***3.80E-03*** | | -0.270 | 3.35E-01 | | |  | | |
| pH | -0.190 | 2.72E-02 | ***3.21E-12*** | -0.202 | ***8.55E-12*** | | -0.266 | ***1.20E-05*** | | |  | | |
| **Malay** |  |  |  |  |  | |  |  | | |  | | |
| TEWL | 0.196 | 7.44E-02 | ***8.46E-03*** | 0.218 | ***8.02E-03*** | | 0.339 | ***3.17E-02*** | | |  | | |
| Moisture | -0.106 | 0.189 | 5.76E-01 | -0.147 | 4.63E-01 | | -0.249 | 4.98E-01 | | |  | | |
| pH | -0.240 | 4.46E-02 | ***9.14E-08*** | -0.222 | ***6.00E-06*** | | -6.26E-02 | 5.06E-01 | | |  | | |
| **Indian** |  |  |  |  |  | |  |  | | |  | | |
| TEWL | 0.322 | 7.62E-02 | ***2.50E-05*** | 0.362 | ***1.60E-05*** | | 0.505 | ***1.74E-03*** | | |  | | |
| Moisture | 0.189 | 0.199 | 3.42E-01 | -0.192 | 3.83E-01 | | -0.111 | 7.68E-01 | | |  | | |
| pH | -0.146 | 4.39E-02 | ***8.94E-04*** | -0.151 | ***1.67E-03*** | | -0.104 | 2.24E-01 | | |  | | |

Model 1: adjusted for age, gender
Model 2: adjusted for Model 1 + education level, household income
Model 3: adjusted for Model 2 + smoking, atopic dermatitis, diabetes mellitus, Hba1c, Insulin Resistance (HOMA-IR), systolic blood pressure, diastolic blood pressure, heart rate, low-density lipoprotein,total white blood cell count, Vitamin D, C-reactive protein.
