# Supplementary material for: Investigating causal relationships between obesity and skin barrier function in a multi-ethnic Asian general population cohort
Source: Int J Obes (Lond). 2023 Jul 21;47(10):963–9. doi: 10.1038/s41366-023-01343-z (PMC10511308; doi:10.1038/s41366-023-01343-z)
Supplement: Supplementary file 6 — Supplementary Table 5 [file 41366_2023_1343_MOESM6_ESM.docx]

| **Supplementary Table 5. Univariate analysis of characteristics of study participant against skin physiology measures, adjusted by age, gender and ethnicity** | | | | | | | | | | | | | | | | | | | |
| --- | --- | --- | --- | --- | --- | --- | --- | --- | --- | --- | --- | --- | --- | --- | --- | --- | --- | --- | --- |
| **Characteristics** |  | **TEWL** |  |  | **Moisture** | | |  |  | | **pH** | | |  | | |  |  |  |
|  | **Beta** | **SE** | **P-value** | **Beta** | **SE** | | | **P-value** | **Beta** | | | **SE** | | **P-value** | | |  |  |  |
| **Age (years)*** | -0.210 | 1.46E-02 | ***2.37E-46*** | -0.858 | 3.50E-02 | ***1.40E-02*** | | | -1.46E-04 | | | 7.60E-04 | | 9.85E-01 | | |  |  |  |
| **Female Gender*** | -6.84 | 3.44 | ***4.41E-86*** | -4.24 | 0.836 | ***4.01E-07*** | | | 3.42 | | | 0.178 | | ***1.04E-80*** | | |  |  |  |
| **Ethnic Groups*** |  |  |  |  |  |  | | |  | | |  | |  | | |  |  |  |
| Chinese | - | - | - | - | - | - | | | - | | | - | | - | | |  |  |  |
| Malay | 2.31 | 0.626 | ***2.22E-04*** | -8.24 | 1.23 | ***2.47E-11*** | | | 0.475 | | | 0.268 | | 7.70E-02 | | |  |  |  |
| Indian | -1.02 | 0.454 | ***2.40E-02*** | -14.7 | 1.07 | ***3.33E-42*** | | | 2.47 | | | 0.234 | | ***5.47E-26*** | | |  |  |  |
| **Education level** |  |  |  |  |  |  | | |  | | |  | |  | | |  |  |  |
| Primary School or no education | - | - | - | - | - | - | | | - | | | - | | - | | |  |  |  |
| O levels or ITE | -8.69 | 0.871 | 0.318 | 6.30 | 2.13 | ***3.04E-03*** | | | 1.01 | | | 0.451 | | ***2.50E-02*** | | |  |  |  |
| A levels or diploma | -1.32 | 0.884 | 0.135 | 8.74 | 2.16 | ***5.20E-05*** | | | 0.228 | | | 0.457 | | 6.19E-01 | | |  |  |  |
| Undergraduate/Graduate | -2.27 | 0.877 | ***1.00E-02*** | 11.5 | 2.14 | ***8.65E-08*** | | | -6.01E-03 | | | 0.454 | | 9.89E-01 | | |  |  |  |
| **Household income ($)/month** | |  |  |  |  |  | | |  | | |  | |  | | |  |  |  |
| <2000 | - | - | - | - | - | ***-*** | | | - | | | - | | ***-*** | | |  |  |  |
| 2000 - 3999 | -0.318 | 0.635 | 0.616 | -1.89 | 1.62 | 2.44E-01 | | | 1.26 | | | 0.350 | | ***3.14E-04*** | | |  |  |  |
| 4000 - 5999 | -0.322 | 0.643 | 0.617 | 1.05 | 1.61 | 5.12E-01 | | | 0.905 | | | 0.346 | | ***8.83E-03*** | | |  |  |  |
| 6000 - 9999 | 0.737 | 0.663 | 0.266 | 1.03 | 1.56 | 5.10E-01 | | | 0.816 | | | 0.336 | | ***1.52E-02*** | | |  |  |  |
| >= 10000 | 0.647 | 0.671 | 0.335 | -0.469 | 1.54 | 7.60E-01 | | | 1.38 | | | 0.332 | | ***3.40E-05*** | | |  |  |  |
| **Supplementary Table 5** *(continued)* | |  |  |  |  | | |  |  | |  | | |  | | |  |  |  |
| **Characteristics** |  | **TEWL** |  |  | **Moisture** | | |  |  | | **pH** | | |  | | |  |  |  |
|  | **Beta** | **SE** | **P-value** | **Beta** | **SE** | **P-value** | | | **Beta** | | **SE** | | | **P-value** | | |  |  |  |
| **Alcohol Consumption** |  |  |  |  |  |  | | |  | |  | | | | |  |  |  |  |
| Never drink | - | - | ***-*** | - | - | ***-*** | | | - | | - | | | | | ***-*** | |  |  |
| Occasional | -0.248 | 0.417 | 5.51E-01 | 2.67 | 1.01 | ***8.43E-03*** | | | 1.49E-03 | | 2.17E-03 | | | | | 4.93E-01 | |  |  |
| 1-3x /month | 0.712 | 0.649 | 2.73E-01 | 2.89 | 1.59 | 6.90E-02 | | | -1.48E-03 | | 3.39E-03 | | | | | 6.62E-01 | |  |  |
| 1-2x/week | -0.271 | 0.747 | 7.17E-01 | 0.698 | 1.81 | 7.00E-01 | | | 2.30E-03 | | 3.90E-03 | | | | | 5.55E-01 | |  |  |
| 3-4x/week | -0.132 | 1.04 | 9.00E-01 | 1.67 | 2.54 | 5.11E-01 | | | 1.08E-02 | | 5.44E-03 | | | | | ***4.60E-02*** | | |  |
| Almost daily | 4.05 | 1.41 | ***4.16E-03*** | 7.91 | 3.46 | ***2.20E-02*** | | | 8.02E-03 | | 7.35E-03 | | | | | 2.76E-01 | | |  |
| **Smoking** |  |  |  |  |  |  | | |  | |  | | | | |  | | |  |
| Never smoke | - | - | ***-*** | - | - | ***-*** | | | - | | - | | | | | ***-*** | | |  |
| Ex-smoker | 0.806 | 0.451 | 7.40E-02 | 6.54 | 1.1 | 5.51E-01 | | | 0.353 | | 0.235 | | | | | 1.32E-01 | | |  |
| Current smoker | -0.785 | 0.651 | 2.28E-01 | -5.67 | 1.58 | ***3.37E-04*** | | | 1.43 | | 0.338 | | | | | ***2.40E-05*** | | |  |
| **Atopic dermatitis** | 0.924 | 0.588 | 1.16E-01 | -5.52 | 1.43 | ***1.09E-04*** | | | 0.984 | | 0.306 | | | | | ***1.28E-03*** | | |  |
| **Diabetes mellitus** | -0.715 | 0.586 | 2.22E-01 | -0.36 | 1.43 | 8.01E-01 | | | -0.844 | | 0.304 | | | | | ***5.50E-03*** | | |  |
| **HbA1c (%)** | -0.131 | 0.202 | 5.17E-01 | -1.86 | 0.496 | ***1.72E-04*** | | | -0.548 | | 0.106 | | | | | ***2.44E-07*** | | |  |
| **Insulin Resistance (HOMA-IR)** | 6.27E-02 | 0.295 | 8.32E-01 | -1.32 | 0.273 | ***1.00E-06*** | | | -0.126 | | 5.99E-02 | | | | | ***3.50E-01*** | | |  |
| **Hypertension** | 0.435 | 0.422 | 3.02E-01 | -0.364 | 1.03 | 7.23E-01 | | | -0.152 | | 0.22 | | | | | 4.90E-01 | | |  |
| **Systolic blood pressure (mmHg)** | 1.04E-02 | 9.60E-03 | 2.81E-01 | -7.81E-02 | 2.32E-02 | ***7.83E-04*** | | | -1.93E-03 | | 5.00E-03 | | | | | 6.97E-01 | | |  |
| **Diastolic blood pressure (mmHg)** | -5.81E-03 | 1.54E-02 | 7.07E-01 | -0.129 | 3.75E-02 | ***5.82E-04*** | | | 3.53E-03 | | 8.00E-03 | | | | | 6.61E-01 | | |  |
| **Pulse pressure (mmHg)** | 2.23E-02 | 1.28E-02 | 8.20E-02 | -5.02E-02 | 3.09E-02 | 1.04E-01 | | | -5.79E-03 | | 7.00E-03 | | | | | 3.80E-01 | |  |  |
| **Supplementary Table 5** *(continued)* | |  |  |  |  |  |  |  |  |  |  |  |  |  |  |  |  |  |  |
| **Characteristics** |  | **TEWL** |  |  | **Moisture** | | |  |  | | **pH** | | |  | | |  |  |  |
|  | **Beta** | **SE** | **P-value** | **Beta** | **SE** | | **P-value** | | | **Beta** | | | **SE** | | **P-value** | |  |  |  |
| **Heart Rate** | 6.41E-02 | 1.44E-02 | ***8.00E-06*** | -5.02E-02 | 3.52E-02 | | 1.39E-01 | | | -1.44E-02 | | | 7.50E-03 | | 5.40E-02 | |  |  |  |
| **Total White Count X10^9^/L** | 0.184 | 0.103 | 7.40E-02 | -1.18 | 0.274 | | ***1.80E-05*** | | | -0.108 | | | 5.46E-02 | | ***4.70E-02*** | |  |  |  |
| **Neutrophils, X10^9^/L** | 0.389 | 0.149 | ***9.00E-03*** | -1.92 | 0.366 | | ***1.55E-07*** | | | -5.66E-02 | | | 7.87E-02 | | 4.72E-01 | |  |  |  |
| **Vitamin D (ng/ml)** | 2.54E-02 | 1.55E-02 | 1.01E-01 | 0.12 | 3.47E-02 | | ***5.61E-04*** | | | -1.60E-02 | | | 7.60E-03 | | ***3.60E-02*** | |  |  |  |
| **C-reactive protein (mg/dL)** | 7.77E-02 | 5.91E-02 | 1.89E-01 | -0.348 | 0.128 | | ***6.64E-03*** | | | -5.67E-02 | | | 2.89E-02 | | ***5.00E-02*** | |  |  |  |

*univariate analysis against skin physiology measures.
