# Supplementary material for: Investigating causal relationships between obesity and skin barrier function in a multi-ethnic Asian general population cohort
Source: Int J Obes (Lond). 2023 Jul 21;47(10):963–9. doi: 10.1038/s41366-023-01343-z (PMC10511308; doi:10.1038/s41366-023-01343-z)
Supplement: Supplementary file 7 — Supplementary Table 6 [file 41366_2023_1343_MOESM7_ESM.pdf]

Supplementary Table 6: Body Mass Index (BMI) SNPs and annotated associations with possible confounders

snp	trait	study	pmid	ancestry	year	beta	se	p	n	n_cases	n_controls	n_studies	unit	dataset
rs1000096	Hayfever, allergic rhinitis or eczema	Neale B	UKBB	European	2017	0.007504	0.001057	1.23E-12	336782	77891	258891	1	risk diff	Neale-B_UKBB_EUR_2017
rs1000096	No blood clot, bronchitis, emphysema, asthma, rhinitis, eczema o	Neale B	UKBB	European	2017	-0.00584	0.001171	6.21E-07	336782	228530	108252	1	risk diff	Neale-B_UKBB_EUR_2017
rs1000096	Qualifications: college or university degree	Neale B	UKBB	European	2017	0.005623	0.001172	1.61E-06	334070	106305	227765	1	risk diff	Neale-B_UKBB_EUR_2017
rs10035289	Pack years adult smoking as proportion of life span exposed to sn	Neale B	UKBB	European	2017	0.01954	0.004387	8.42E-06	101726	0	101726	1	IVNT	Neale-B_UKBB_EUR_2017
rs1035010	Diastolic blood pressure	Neale B	UKBB	European	2017	0.01724	0.002815	9.07E-10	317756	0	317756	1	IVNT	Neale-B_UKBB_EUR_2017
rs1035010	Systolic blood pressure	Neale B	UKBB	European	2017	0.01454	0.002813	2.35E-07	317754	0	317754	1	IVNT	Neale-B_UKBB_EUR_2017
rs10733051	Asthma	Neale B	UKBB	European	2017	-0.00359	0.00078	4.06E-06	336782	38791	297991	1	risk diff	Neale-B_UKBB_EUR_2017
rs10733051	Hayfever, allergic rhinitis or eczema	Neale B	UKBB	European	2017	-0.00495	0.001029	1.47E-06	336782	77891	258891	1	risk diff	Neale-B_UKBB_EUR_2017
rs10733051	No blood clot, bronchitis, emphysema, asthma, rhinitis, eczema o	Neale B	UKBB	European	2017	0.005385	0.00114	2.31E-06	336782	228530	108252	1	risk diff	Neale-B_UKBB_EUR_2017
rs10733051	Self-reported asthma	Neale B	UKBB	European	2017	-0.00348	0.000781	8.57E-06	337159	39049	298110	1	risk diff	Neale-B_UKBB_EUR_2017
rs10797115	Qualifications: college or university degree	Neale B	UKBB	European	2017	0.006757	0.001146	3.69E-09	334070	106305	227765	1	risk diff	Neale-B_UKBB_EUR_2017
rs10734604	Diastolic blood pressure	Neale B	UKBB	European	2017	0.01655	0.003218	2.70E-07	317756	0	317756	1	IVNT	Neale-B_UKBB_EUR_2017
rs10734604	Self-reported hypertension	Neale B	UKBB	European	2017	0.008266	0.001393	2.95E-09	337159	87690	249469	1	risk diff	Neale-B_UKBB_EUR_2017
rs10734604	Systolic blood pressure	Neale B	UKBB	European	2017	0.01748	0.003216	5.50E-08	317754	0	317754	1	IVNT	Neale-B_UKBB_EUR_2017
rs10734604	Vascular or heart problems diagnosed by doctor: high blood press	Neale B	UKBB	European	2017	0.008566	0.001412	1.31E-09	336683	91033	245650	1	risk diff	Neale-B_UKBB_EUR_2017
rs479971	Past tobacco smoking	Neale B	UKBB	European	2017	0.0182	0.003547	2.86E-07	310749	0	310749	1	-	Neale-B_UKBB_EUR_2017
rs479971	Treatment with blood pressure medication	Neale B	UKBB	European	2017	-0.00633	0.00142	8.38E-06	180203	31488	148715	1	risk diff	Neale-B_UKBB_EUR_2017
rs10955841	Qualifications: college or university degree	Neale B	UKBB	European	2017	0.006453	0.001213	1.03E-07	334070	106305	227765	1	risk diff	Neale-B_UKBB_EUR_2017
rs10955841	Qualifications: none	Neale B	UKBB	European	2017	-0.00435	0.000983	9.77E-06	334070	57567	276503	1	risk diff	Neale-B_UKBB_EUR_2017
rs11030385	Qualifications: college or university degree	Neale B	UKBB	European	2017	0.005289	0.001164	5.52E-06	334070	106305	227765	1	risk diff	Neale-B_UKBB_EUR_2017
rs11030385	Wheeze or whistling in the chest in last year	Neale B	UKBB	European	2017	-0.005	0.001017	9.04E-07	331257	68531	262726	1	risk diff	Neale-B_UKBB_EUR_2017
rs11030385	Former smoker	TAG	20418890	European	2010	0.0738	0.0165	7.84E-06	41278	23969	17309	14	log OR	TAG_FORMER_EUR_2010
rs11066301	Diastolic blood pressure	BPExome	27618447	Mixed	2016	NA	NA	4.49E-09	192763	0	192763	51	INVT	BPExome_DBP_Mixed_2016
rs11066301	Hypertension	BPExome	27618447	Mixed	2016	NA	NA	1.87E-08	183273	92652	90621	51	log OR	BPExome_Hypertension_Mixed_2016
rs11066301	Diastolic blood pressure	ICBP	21909115	European	2011	NA	NA	6.59E-11	69395	-	-	-	-	GRASP
rs11066301	Systolic blood pressure	ICBP	21909115	European	2011	NA	NA	4.94E-08	69395	-	-	-	-	GRASP
rs11066301	Diastolic blood pressure	ICBP	21909115	European	2011	NA	NA	6.59E-11	69395	0	69395	29	mmHg	ICBP_DBP_EUR_2011
rs11066301	Systolic blood pressure	ICBP	21909115	European	2011	NA	NA	4.94E-08	69395	0	69395	29	mmHg	ICBP_SBP_EUR_2011
rs11066301	Diastolic blood pressure	Neale B	UKBB	European	2017	-0.02776	0.002491	7.94E-29	317756	0	317756	1	IVNT	Neale-B_UKBB_EUR_2017
rs11066301	Doctor diagnosed hayfever or allergic rhinitis	Neale B	UKBB	European	2017	0.01018	0.002075	9.39E-07	83529	18934	64595	1	risk diff	Neale-B_UKBB_EUR_2017
rs11066301	Ever smoked	Neale B	UKBB	European	2017	-0.00554	0.001204	4.17E-06	336067	202585	133482	1	risk diff	Neale-B_UKBB_EUR_2017
rs11066301	Hayfever, allergic rhinitis or eczema	Neale B	UKBB	European	2017	0.00682	0.001039	5.34E-11	336782	77891	258891	1	risk diff	Neale-B_UKBB_EUR_2017
rs11066301	Illnesses of siblings: high blood pressure	Neale B	UKBB	European	2017	-0.00526	0.001121	2.70E-06	262361	54270	208091	1	risk diff	Neale-B_UKBB_EUR_2017
rs11066301	No blood clot, bronchitis, emphysema, asthma, rhinitis, eczema o	Neale B	UKBB	European	2017	-0.00585	0.001152	3.74E-07	336782	228530	108252	1	risk diff	Neale-B_UKBB_EUR_2017
rs11066301	Past tobacco smoking	Neale B	UKBB	European	2017	0.01753	0.003205	4.49E-08	310749	0	310749	1	-	Neale-B_UKBB_EUR_2017
rs11066301	Self-reported hypertension	Neale B	UKBB	European	2017	-0.00871	0.001079	6.77E-16	337159	87690	249469	1	risk diff	Neale-B_UKBB_EUR_2017
rs11066301	Smoking status: previous	Neale B	UKBB	European	2017	-0.00626	0.001177	1.07E-07	336024	118419	217605	1	risk diff	Neale-B_UKBB_EUR_2017
rs11066301	Systolic blood pressure	Neale B	UKBB	European	2017	-0.01728	0.00249	4.01E-12	317754	0	317754	1	IVNT	Neale-B_UKBB_EUR_2017
rs11066301	Treatment with blood pressure medication	Neale B	UKBB	European	2017	-0.00761	0.00128	2.75E-09	180203	31488	148715	1	risk diff	Neale-B_UKBB_EUR_2017
rs11066301	Vascular or heart problems diagnosed by doctor: high blood press	Neale B	UKBB	European	2017	-0.00915	0.001094	6.09E-17	336683	91033	245650	1	risk diff	Neale-B_UKBB_EUR_2017
rs4321967	Diastolic blood pressure	Neale B	UKBB	European	2017	0.01651	0.002489	3.30E-11	317756	0	317756	1	IVNT	Neale-B_UKBB_EUR_2017
rs4321967	Self-reported hypertension	Neale B	UKBB	European	2017	0.007122	0.001078	3.97E-11	337159	87690	249469	1	risk diff	Neale-B_UKBB_EUR_2017
rs4321967	Systolic blood pressure	Neale B	UKBB	European	2017	0.01787	0.002487	6.71E-13	317754	0	317754	1	IVNT	Neale-B_UKBB_EUR_2017
rs4321967	Treatment with blood pressure medication	Neale B	UKBB	European	2017	0.006239	0.001281	1.11E-06	180203	31488	148715	1	risk diff	Neale-B_UKBB_EUR_2017
rs4321967	Vascular or heart problems diagnosed by doctor: high blood press	Neale B	UKBB	European	2017	0.007476	0.001093	7.97E-12	336683	91033	245650	1	risk diff	Neale-B_UKBB_EUR_2017
rs11259933	Asthma	Neale B	UKBB	European	2017	-0.00368	0.000779	2.34E-06	336782	38791	297991	1	risk diff	Neale-B_UKBB_EUR_2017
rs11259933	Self-reported asthma	Neale B	UKBB	European	2017	-0.00379	0.00078	1.18E-06	337159	39049	298110	1	risk diff	Neale-B_UKBB_EUR_2017
rs11496125	Systolic blood pressure	Neale B	UKBB	European	2017	-0.01152	0.0025	4.03E-06	317754	0	317754	1	IVNT	Neale-B_UKBB_EUR_2017
rs11672660	2 hour glucose	MAGIC	20081857	European	2010	NA	NA	4.06E-06	15234	-	-	-	-	GRASP
rs11672660	2 hour glucose tolerance test	MAGIC	22885924	European	2012	NA	NA	2.72E-27	133010	-	-	-	-	GRASP
rs11672660	Fasting blood glucose	MAGIC	22885924	European	2012	NA	NA	5.83E-09	133010	-	-	-	-	GRASP
rs11672660	2 hour fasting glucose	MAGIC	22885924	European	2012	-0.12	0.014	2.40E-16	42854	0	42854	20	mmol/l	MAGIC_2HG-METABO_EUR_2012
rs11672660	2 hour fasting glucose	MAGIC	20081857	European	2010	-0.15	0.032	4.06E-06	15234	0	15234	9	mmol/l	MAGIC_2HG_EUR_2010
rs11672660	Fasting glucose	MAGIC	22885924	European	2012	0.016	0.0028	5.83E-09	133010	0	133010	66	mmol/l	MAGIC_FG-METABO_EUR_2012
rs11753081	Type II diabetes	DIAGRAM	26551672	European	2015	0.124	0.01623	3.50E-14	84780	27206	57574	23	log OR	DIAGRAM_T2D-METABO_EUR_2015
rs11753081	Type II diabetes adjusted for BMI	DIAGRAM	28566273	European	2017	0.2	0.019	4.60E-26	159208	26676	132532	18	log OR	DIAGRAM_T2D-adjusted-BMI_EUR_2017
rs11753081	Type II diabetes	DIAGRAM	22885922	European	2012	0.207	0.02491	1.70E-17	63390	9580	53810	12	log OR	DIAGRAM_T2D_EUR_2012

rs11753081	Type II diabetes	DIAGRAM	28566273	European	2017	0.17	0.016	1.30E-27	159208	26676	132532	18	log OR	DIAGRAM_T2D_EUR_2017
rs11753081	Type II diabetes	DIAGRAM	24509480	Mixed	2014	0.1484	0.01546	6.80E-23	110452	26488	83964	4	log OR	DIAGRAM_T2D_Mixed_2014
rs11753081	Glycosylated hemoglobin in nondiabetic subjects	Ryu J	22290723	East Asian	2012	NA	NA	1.61E-08	4275	-	-	-	-	GRASP
rs11753081	Type 2 diabetes	Cho YS	22158537	Mixed	2011	NA	NA	4.47E-16	18817	-	-	-	-	GRASP
rs11753081	Type 2 diabetes	Imamura M	22456796	East Asian	2012	NA	NA	6.52E-06	7541	-	-	-	-	GRASP
rs11753081	Fasting glucose	MAGIC	22885924	European	2012	0.015	0.0028	2.27E-08	133010	0	133010	66	mmol/l	MAGIC_FG-METABO_EUR_2012
rs11753081	Diabetes diagnosed by doctor	Neale B	UKBB	European	2017	0.007004	0.000687	1.97E-24	336473	16183	320290	1	risk diff	Neale-B_UKBB_EUR_2017
rs11753081	Illnesses of father: diabetes	Neale B	UKBB	European	2017	0.005092	0.000997	3.25E-07	293407	28197	265210	1	risk diff	Neale-B_UKBB_EUR_2017
rs11753081	Illnesses of mother: diabetes	Neale B	UKBB	European	2017	0.004668	0.00096	1.16E-06	309953	28875	281078	1	risk diff	Neale-B_UKBB_EUR_2017
rs11753081	Self-reported diabetes	Neale B	UKBB	European	2017	0.005398	0.000623	4.35E-18	337159	13243	323916	1	risk diff	Neale-B_UKBB_EUR_2017
rs11753081	Self-reported type 2 diabetes	Neale B	UKBB	European	2017	0.001317	0.000255	2.33E-07	337159	2133	335026	1	risk diff	Neale-B_UKBB_EUR_2017
rs11753081	Treatment with gliclazide	Neale B	UKBB	European	2017	0.001432	0.000292	9.19E-07	337159	2807	334352	1	risk diff	Neale-B_UKBB_EUR_2017
rs11855821	Qualifications: college or university degree	Neale B	UKBB	European	2017	0.007124	0.001286	3.00E-08	334070	106305	227765	1	risk diff	Neale-B_UKBB_EUR_2017
rs11855821	Qualifications: none	Neale B	UKBB	European	2017	-0.00569	0.001042	4.89E-08	334070	57567	276503	1	risk diff	Neale-B_UKBB_EUR_2017
rs11855821	Years of educational attainment	SSGAC	27225129	European	2016	0.014	0.003	6.68E-07	328917	0	328917	64	years	SSGAC_EduYears_EUR_2016
rs11889536	Years of education	SSGAC	23722424	European	2013	NA	NA	9.13E-06	101069	-	-	-	-	GRASP
rs11889536	Years of educational attainment	SSGAC	23722424	European	2013	-0.024	0.005	9.13E-06	101069	0	101069	42	years	SSGAC_EduYears_EUR_2013
rs12042908	Ever smoked	Neale B	UKBB	European	2017	0.006194	0.0012	2.42E-07	336067	202585	133482	1	risk diff	Neale-B_UKBB_EUR_2017
rs12042908	Past tobacco smoking	Neale B	UKBB	European	2017	-0.01695	0.003196	1.14E-07	310749	0	310749	1	-	Neale-B_UKBB_EUR_2017
rs10927006	Exposure to tobacco smoke outside home	Neale B	UKBB	European	2017	-0.01152	0.002101	4.14E-08	286550	0	286550	1	-	Neale-B_UKBB_EUR_2017
rs10927006	Qualifications: none	Neale B	UKBB	European	2017	-0.0061	0.001312	3.36E-06	334070	57567	276503	1	risk diff	Neale-B_UKBB_EUR_2017
rs12044597	Systolic blood pressure	Neale B	UKBB	European	2017	0.01232	0.002465	5.81E-07	317754	0	317754	1	IVNT	Neale-B_UKBB_EUR_2017
rs12101393	Qualifications: A levels or as levels or equivalent	Neale B	UKBB	European	2017	-0.006	0.001325	5.86E-06	334070	91710	242360	1	risk diff	Neale-B_UKBB_EUR_2017
rs12411886	Systolic blood pressure	ICBP	21909115	European	2011	NA	NA	3.26E-09	69395	-	-	-	-	GRASP
rs12411886	Systolic blood pressure	ICBP	21909115	European	2011	NA	NA	3.26E-09	69395	0	69395	29	mmHg	ICBP_SBP_EUR_2011
rs12411886	Diastolic blood pressure	Neale B	UKBB	European	2017	-0.03529	0.004624	2.31E-14	317756	0	317756	1	IVNT	Neale-B_UKBB_EUR_2017
rs12411886	Illnesses of mother: high blood pressure	Neale B	UKBB	European	2017	-0.01151	0.00218	1.30E-07	311681	94817	216864	1	risk diff	Neale-B_UKBB_EUR_2017
rs12411886	Self-reported hypertension	Neale B	UKBB	European	2017	-0.01476	0.002002	1.67E-13	337159	87690	249469	1	risk diff	Neale-B_UKBB_EUR_2017
rs12411886	Systolic blood pressure	Neale B	UKBB	European	2017	-0.04758	0.004621	7.43E-25	317754	0	317754	1	IVNT	Neale-B_UKBB_EUR_2017
rs12411886	Treatment with blood pressure medication	Neale B	UKBB	European	2017	-0.01347	0.002387	1.67E-08	180203	31488	148715	1	risk diff	Neale-B_UKBB_EUR_2017
rs12411886	Vascular or heart problems diagnosed by doctor: high blood press	Neale B	UKBB	European	2017	-0.0154	0.00203	3.36E-14	336683	91033	245650	1	risk diff	Neale-B_UKBB_EUR_2017
rs12448738	Diastolic blood pressure	Neale B	UKBB	European	2017	-0.01686	0.003626	3.35E-06	317756	0	317756	1	IVNT	Neale-B_UKBB_EUR_2017
rs12593036	Self-reported hypertension	Neale B	UKBB	European	2017	0.006869	0.001161	3.26E-09	337159	87690	249469	1	risk diff	Neale-B_UKBB_EUR_2017
rs12593036	Vascular or heart problems diagnosed by doctor: high blood press	Neale B	UKBB	European	2017	0.006902	0.001177	4.48E-09	336683	91033	245650	1	risk diff	Neale-B_UKBB_EUR_2017
rs12638746	Smoking status: previous	Neale B	UKBB	European	2017	-0.00577	0.001237	3.13E-06	336024	118419	217605	1	risk diff	Neale-B_UKBB_EUR_2017
rs12680842	Pack years adult smoking as proportion of life span exposed to sm	Neale B	UKBB	European	2017	0.02164	0.004729	4.75E-06	101726	0	101726	1	IVNT	Neale-B_UKBB_EUR_2017
rs13246064	Self-reported hypertension	Neale B	UKBB	European	2017	0.005227	0.001084	1.41E-06	337159	87690	249469	1	risk diff	Neale-B_UKBB_EUR_2017
rs13246064	Vascular or heart problems diagnosed by doctor: high blood press	Neale B	UKBB	European	2017	0.005848	0.001099	1.02E-07	336683	91033	245650	1	risk diff	Neale-B_UKBB_EUR_2017
rs13246064	Wheeze or whistling in the chest in last year	Neale B	UKBB	European	2017	0.006413	0.001012	2.32E-10	331257	68531	262726	1	risk diff	Neale-B_UKBB_EUR_2017
rs12713433	Hayfever, allergic rhinitis or eczema	Neale B	UKBB	European	2017	0.006565	0.001285	3.26E-07	336782	77891	258891	1	risk diff	Neale-B_UKBB_EUR_2017
rs12713433	No blood clot, bronchitis, emphysema, asthma, rhinitis, eczema o	Neale B	UKBB	European	2017	-0.00632	0.001424	9.14E-06	336782	228530	108252	1	risk diff	Neale-B_UKBB_EUR_2017
rs12964689	Qualifications: college or university degree	Neale B	UKBB	European	2017	-0.00574	0.001138	4.57E-07	334070	106305	227765	1	risk diff	Neale-B_UKBB_EUR_2017
rs12964689	Years of educational attainment	SSGAC	27225129	European	2016	-0.014	0.002	1.19E-08	328917	0	328917	64	years	SSGAC_EduYears_EUR_2016
rs13021737	Diastolic blood pressure	Neale B	UKBB	European	2017	-0.016	0.003262	9.36E-07	317756	0	317756	1	IVNT	Neale-B_UKBB_EUR_2017
rs13021737	Past tobacco smoking	Neale B	UKBB	European	2017	0.02006	0.004193	1.71E-06	310749	0	310749	1	-	Neale-B_UKBB_EUR_2017
rs13021737	Systolic blood pressure	Neale B	UKBB	European	2017	-0.01604	0.00326	8.66E-07	317754	0	317754	1	IVNT	Neale-B_UKBB_EUR_2017
rs13227433	Age completed full time education	Neale B	UKBB	European	2017	-0.01406	0.002755	3.32E-07	226899	0	226899	1	-	Neale-B_UKBB_EUR_2017
rs13227433	Self-reported hypertension	Neale B	UKBB	European	2017	0.006764	0.001213	2.47E-08	337159	87690	249469	1	risk diff	Neale-B_UKBB_EUR_2017
rs13227433	Vascular or heart problems diagnosed by doctor: high blood press	Neale B	UKBB	European	2017	0.006739	0.00123	4.24E-08	336683	91033	245650	1	risk diff	Neale-B_UKBB_EUR_2017
rs7794705	Ever smoked	Neale B	UKBB	European	2017	0.008999	0.001644	4.41E-08	336067	202585	133482	1	risk diff	Neale-B_UKBB_EUR_2017
rs7794705	Past tobacco smoking	Neale B	UKBB	European	2017	-0.02164	0.004372	7.47E-07	310749	0	310749	1	-	Neale-B_UKBB_EUR_2017
rs13290794	Diastolic blood pressure	Neale B	UKBB	European	2017	-0.01395	0.00255	4.49E-08	317756	0	317756	1	IVNT	Neale-B_UKBB_EUR_2017
rs13290794	Self-reported hypertension	Neale B	UKBB	European	2017	-0.00506	0.001104	4.60E-06	337159	87690	249469	1	risk diff	Neale-B_UKBB_EUR_2017
rs13290794	Vascular or heart problems diagnosed by doctor: high blood press	Neale B	UKBB	European	2017	-0.00506	0.001119	6.27E-06	336683	91033	245650	1	risk diff	Neale-B_UKBB_EUR_2017
rs13321566	Qualifications: college or university degree	Neale B	UKBB	European	2017	0.006738	0.001516	8.78E-06	334070	106305	227765	1	risk diff	Neale-B_UKBB_EUR_2017
rs13321566	Years of educational attainment in females	SSGAC	27225129	European	2016	0.022	0.004	2.72E-07	181443	0	181443	62	years	SSGAC_EduYears-F_EUR_2016
rs13321566	Years of educational attainment	SSGAC	27225129	European	2016	0.016	0.003	1.30E-06	328917	0	328917	64	years	SSGAC_EduYears_EUR_2016
rs62250504	Ever smoked	Neale B	UKBB	European	2017	-0.00769	0.001231	4.23E-10	336067	202585	133482	1	risk diff	Neale-B_UKBB_EUR_2017
rs62250504	Past tobacco smoking	Neale B	UKBB	European	2017	0.02201	0.003278	1.88E-11	310749	0	310749	1	-	Neale-B_UKBB_EUR_2017

rs62250504	Qualifications: A levels or as levels or equivalent	Neale B	UKBB	European	2017	0.005926	0.001131	1.59E-07	334070	91710	242360	1	risk diff	Neale-B_UKBB_EUR_2017
rs62250504	Qualifications: college or university degree	Neale B	UKBB	European	2017	0.008009	0.001179	1.12E-11	334070	106305	227765	1	risk diff	Neale-B_UKBB_EUR_2017
rs62250504	Smoking status: previous	Neale B	UKBB	European	2017	-0.00641	0.001205	1.04E-07	336024	118419	217605	1	risk diff	Neale-B_UKBB_EUR_2017
rs62250504	Wheeze or whistling in the chest in last year	Neale B	UKBB	European	2017	-0.00478	0.001031	3.54E-06	331257	68531	262726	1	risk diff	Neale-B_UKBB_EUR_2017
rs62250504	Years of educational attainment in males	SSGAC	27225129	European	2016	0.018	0.004	3.33E-06	147474	0	147474	6	years	SSGAC_EduYears-M_EUR_2016
rs62250504	Years of educational attainment	SSGAC	27225129	European	2016	0.013	0.003	4.27E-07	328917	0	328917	64	years	SSGAC_EduYears_EUR_2016
rs1394	Diastolic blood pressure	Neale B	UKBB	European	2017	0.01794	0.002554	2.18E-12	317756	0	317756	1	IVNT	Neale-B_UKBB_EUR_2017
rs1394	Self-reported hypertension	Neale B	UKBB	European	2017	0.006632	0.001106	2.04E-09	337159	87690	249469	1	risk diff	Neale-B_UKBB_EUR_2017
rs1394	Systolic blood pressure	Neale B	UKBB	European	2017	0.01467	0.002553	9.19E-09	317754	0	317754	1	IVNT	Neale-B_UKBB_EUR_2017
rs1394	Vascular or heart problems diagnosed by doctor: high blood press	Neale B	UKBB	European	2017	0.007113	0.001122	2.28E-10	336683	91033	245650	1	risk diff	Neale-B_UKBB_EUR_2017
rs1399054	Diastolic blood pressure	Neale B	UKBB	European	2017	0.01459	0.00305	1.71E-06	317756	0	317756	1	IVNT	Neale-B_UKBB_EUR_2017
rs1405348	Self-reported hypertension	Neale B	UKBB	European	2017	0.005287	0.001075	8.72E-07	337159	87690	249469	1	risk diff	Neale-B_UKBB_EUR_2017
rs1405348	Systolic blood pressure	Neale B	UKBB	European	2017	0.01306	0.002481	1.41E-07	317754	0	317754	1	IVNT	Neale-B_UKBB_EUR_2017
rs1405348	Vascular or heart problems diagnosed by doctor: high blood press	Neale B	UKBB	European	2017	0.00563	0.00109	2.39E-07	336683	91033	245650	1	risk diff	Neale-B_UKBB_EUR_2017
rs1452075	Type 2 diabetes	Zeggini E	18372903	European	2008	NA	NA	6.16E-07	10128	-	-	-	-	GRASP
rs1452075	Qualifications: college or university degree	Neale B	UKBB	European	2017	-0.00665	0.001292	2.59E-07	334070	106305	227765	1	risk diff	Neale-B_UKBB_EUR_2017
rs17056301	Self-reported hypertension	Neale B	UKBB	European	2017	0.006794	0.001228	3.14E-08	337159	87690	249469	1	risk diff	Neale-B_UKBB_EUR_2017
rs17056301	Systolic blood pressure	Neale B	UKBB	European	2017	0.01877	0.002833	3.46E-11	317754	0	317754	1	IVNT	Neale-B_UKBB_EUR_2017
rs17056301	Vascular or heart problems diagnosed by doctor: high blood press	Neale B	UKBB	European	2017	0.00678	0.001245	5.14E-08	336683	91033	245650	1	risk diff	Neale-B_UKBB_EUR_2017
rs17080319	Qualifications: college or university degree	Neale B	UKBB	European	2017	0.01066	0.001727	6.77E-10	334070	106305	227765	1	risk diff	Neale-B_UKBB_EUR_2017
rs17080319	Years of educational attainment	SSGAC	27225129	European	2016	0.021	0.004	1.01E-07	328917	0	328917	64	years	SSGAC_EduYears_EUR_2016
rs3123794	Systolic blood pressure	Neale B	UKBB	European	2017	0.01499	0.002466	1.22E-09	317754	0	317754	1	IVNT	Neale-B_UKBB_EUR_2017
rs794369	Age completed full time education	Neale B	UKBB	European	2017	0.01143	0.002461	3.43E-06	226899	0	226899	1	-	Neale-B_UKBB_EUR_2017
rs794369	Diastolic blood pressure	Neale B	UKBB	European	2017	-0.01285	0.002497	2.69E-07	317756	0	317756	1	IVNT	Neale-B_UKBB_EUR_2017
rs794369	Qualifications: A levels or as levels or equivalent	Neale B	UKBB	European	2017	0.00541	0.001107	1.03E-06	334070	91710	242360	1	risk diff	Neale-B_UKBB_EUR_2017
rs794369	Qualifications: college or university degree	Neale B	UKBB	European	2017	0.005756	0.001155	6.27E-07	334070	106305	227765	1	risk diff	Neale-B_UKBB_EUR_2017
rs794369	Self-reported hypertension	Neale B	UKBB	European	2017	-0.00488	0.001081	6.38E-06	337159	87690	249469	1	risk diff	Neale-B_UKBB_EUR_2017
rs794369	Systolic blood pressure	Neale B	UKBB	European	2017	-0.01209	0.002496	1.28E-06	317754	0	317754	1	IVNT	Neale-B_UKBB_EUR_2017
rs794369	Vascular or heart problems diagnosed by doctor: high blood press	Neale B	UKBB	European	2017	-0.00494	0.001096	6.67E-06	336683	91033	245650	1	risk diff	Neale-B_UKBB_EUR_2017
rs17522122	Age completed full time education	Neale B	UKBB	European	2017	0.01107	0.002434	5.40E-06	226899	0	226899	1	-	Neale-B_UKBB_EUR_2017
rs17681708	Type II diabetes	DIAGRAM	28566273	European	2017	0.064	0.013	5.60E-07	159208	26676	132532	18	log OR	DIAGRAM_T2D_EUR_2017
rs17724992	Systolic blood pressure	Neale B	UKBB	European	2017	0.01399	0.002787	5.15E-07	317754	0	317754	1	IVNT	Neale-B_UKBB_EUR_2017
rs964782	Current employment status: unable to work because of sickness (	Neale B	UKBB	European	2017	0.002623	0.000522	5.07E-07	336252	12554	323698	1	risk diff	Neale-B_UKBB_EUR_2017
rs964782	Diastolic blood pressure	Neale B	UKBB	European	2017	0.01285	0.002782	3.87E-06	317756	0	317756	1	IVNT	Neale-B_UKBB_EUR_2017
rs964782	Qualifications: college or university degree	Neale B	UKBB	European	2017	-0.00592	0.001287	4.29E-06	334070	106305	227765	1	risk diff	Neale-B_UKBB_EUR_2017
rs964782	Qualifications: none	Neale B	UKBB	European	2017	0.005714	0.001044	4.40E-08	334070	57567	276503	1	risk diff	Neale-B_UKBB_EUR_2017
rs964782	Systolic blood pressure	Neale B	UKBB	European	2017	0.02007	0.00278	5.25E-13	317754	0	317754	1	IVNT	Neale-B_UKBB_EUR_2017
rs964782	Vascular or heart problems diagnosed by doctor: high blood press	Neale B	UKBB	European	2017	0.005402	0.001222	9.78E-06	336683	91033	245650	1	risk diff	Neale-B_UKBB_EUR_2017
rs1899951	Type II diabetes	DIAGRAM	26551672	European	2015	-0.09713	0.01851	1.40E-07	84780	27206	57574	23	log OR	DIAGRAM_T2D-METABO_EUR_2015
rs1899951	Type II diabetes adjusted for BMI	DIAGRAM	28566273	European	2017	-0.14	0.022	7.30E-11	159208	26676	132532	18	log OR	DIAGRAM_T2D-adjusted-BMI_EUR_2017
rs1899951	Type II diabetes	DIAGRAM	22885922	European	2012	-0.1484	0.0241	3.80E-09	69033	12171	56862	12	log OR	DIAGRAM_T2D_EUR_2012
rs1899951	Type II diabetes	DIAGRAM	28566273	European	2017	-0.12	0.019	5.20E-11	159208	26676	132532	18	log OR	DIAGRAM_T2D_EUR_2017
rs1899951	Type II diabetes	DIAGRAM	24509480	Mixed	2014	-0.131	0.02024	2.40E-10	110452	26488	83964	4	log OR	DIAGRAM_T2D_Mixed_2014
rs1899951	Type 2 diabetes	DIAGRAM	22885922	Mixed	2012	NA	NA	3.70E-07	69033	-	-	-	-	GRASP
rs1899951	Diabetes diagnosed by doctor	Neale B	UKBB	European	2017	-0.00412	0.000794	2.10E-07	336473	16183	320290	1	risk diff	Neale-B_UKBB_EUR_2017
rs1899951	Illnesses of mother: diabetes	Neale B	UKBB	European	2017	-0.00521	0.001111	2.79E-06	309953	28875	281078	1	risk diff	Neale-B_UKBB_EUR_2017
rs1899951	Self-reported diabetes	Neale B	UKBB	European	2017	-0.0036	0.00072	6.00E-07	337159	13243	323916	1	risk diff	Neale-B_UKBB_EUR_2017
rs200968	Type 1 diabetes	Hakonarso	17632545	European	2007	NA	NA	3.33E-09	3105	-	-	-	-	GRASP
rs200968	Diastolic blood pressure	Neale B	UKBB	European	2017	0.02614	0.0031	3.45E-17	317756	0	317756	1	IVNT	Neale-B_UKBB_EUR_2017
rs200968	Treatment with insulin	Neale B	UKBB	European	2017	-0.00194	0.000366	1.10E-07	180203	1377	178826	1	risk diff	Neale-B_UKBB_EUR_2017
rs200968	Treatment with insulin product	Neale B	UKBB	European	2017	-0.00217	0.000303	8.42E-13	337159	3319	333840	1	risk diff	Neale-B_UKBB_EUR_2017
rs200968	Asthma	TAGC	29273806	European	2018	-0.07503	0.0167	7.05E-06	127669	19954	107715	56	log OR	TAGC_Asthma_EUR_2018
rs2075650	Diabetes diagnosed by doctor	Neale B	UKBB	European	2017	0.003293	0.000736	7.72E-06	336473	16183	320290	1	risk diff	Neale-B_UKBB_EUR_2017
rs2075650	Illnesses of mother: diabetes	Neale B	UKBB	European	2017	0.006424	0.001029	4.26E-10	309953	28875	281078	1	risk diff	Neale-B_UKBB_EUR_2017
rs2075650	Treatment with atorvastatin	Neale B	UKBB	European	2017	-0.00667	0.000588	7.86E-30	337159	10167	326992	1	risk diff	Neale-B_UKBB_EUR_2017
rs2075650	Treatment with rosuvastatin	Neale B	UKBB	European	2017	-0.00157	0.00027	6.10E-09	337159	2089	335070	1	risk diff	Neale-B_UKBB_EUR_2017
rs2075650	Treatment with simvastatin	Neale B	UKBB	European	2017	-0.01266	0.001089	3.02E-31	337159	38492	298667	1	risk diff	Neale-B_UKBB_EUR_2017
rs215632	Nicotine dependence smoking cigarettes per day	Thorgeirss	20418888	European	2010	NA	NA	1.90E-08	31266	-	-	-	-	GRASP
rs215632	Difficulty not smoking for 1 day	Neale B	UKBB	European	2017	0.0398	0.008675	4.50E-06	23332	0	23332	1	-	Neale-B_UKBB_EUR_2017

rs215632	Pack years adult smoking as proportion of life span exposed to sn	Neale B	UKBB	European	2017	0.02985	0.004545	5.14E-11	101726	0	101726	1	IVNT	Neale-B_UKBB_EUR_2017
rs215632	Pack years of smoking preview only	Neale B	UKBB	European	2017	0.02861	0.004529	2.70E-10	101726	0	101726	1	IVNT	Neale-B_UKBB_EUR_2017
rs215632	Qualifications: A levels or as levels or equivalent	Neale B	UKBB	European	2017	-0.00573	0.001134	4.39E-07	334070	91710	242360	1	risk diff	Neale-B_UKBB_EUR_2017
rs215632	Qualifications: college or university degree	Neale B	UKBB	European	2017	-0.00658	0.001183	2.64E-08	334070	106305	227765	1	risk diff	Neale-B_UKBB_EUR_2017
rs7919685	Age completed full time education	Neale B	UKBB	European	2017	0.01436	0.002425	3.19E-09	226899	0	226899	1	-	Neale-B_UKBB_EUR_2017
rs7919685	Diastolic blood pressure	Neale B	UKBB	European	2017	-0.01753	0.002464	1.14E-12	317756	0	317756	1	IVNT	Neale-B_UKBB_EUR_2017
rs7919685	Qualifications: A levels or as levels or equivalent	Neale B	UKBB	European	2017	0.005654	0.001093	2.30E-07	334070	91710	242360	1	risk diff	Neale-B_UKBB_EUR_2017
rs7919685	Qualifications: college or university degree	Neale B	UKBB	European	2017	0.005944	0.00114	1.85E-07	334070	106305	227765	1	risk diff	Neale-B_UKBB_EUR_2017
rs7919685	Qualifications: none	Neale B	UKBB	European	2017	-0.00624	0.000924	1.52E-11	334070	57567	276503	1	risk diff	Neale-B_UKBB_EUR_2017
rs7919685	Years of educational attainment in males	SSGAC	27225129	European	2016	0.02	0.004	1.02E-07	147474	0	147474	6	years	SSGAC_EduYears-M_EUR_2016
rs7919685	Years of educational attainment	SSGAC	27225129	European	2016	0.014	0.002	2.33E-08	328917	0	328917	64	years	SSGAC_EduYears_EUR_2016
rs2185027	Fasting glucose	MAGIC	22885924	European	2012	-0.01	0.0022	2.55E-06	133010	0	133010	66	mmol/l	MAGIC_FG-METABO_EUR_2012
rs9848497	Age completed full time education	Neale B	UKBB	European	2017	-0.01505	0.002425	5.48E-10	226899	0	226899	1	-	Neale-B_UKBB_EUR_2017
rs9848497	Diabetes diagnosed by doctor	Neale B	UKBB	European	2017	0.002357	0.000521	5.97E-06	336473	16183	320290	1	risk diff	Neale-B_UKBB_EUR_2017
rs9848497	Diastolic blood pressure	Neale B	UKBB	European	2017	0.01361	0.002461	3.18E-08	317756	0	317756	1	IVNT	Neale-B_UKBB_EUR_2017
rs9848497	Qualifications: A levels or as levels or equivalent	Neale B	UKBB	European	2017	-0.0106	0.001091	2.51E-22	334070	91710	242360	1	risk diff	Neale-B_UKBB_EUR_2017
rs9848497	Qualifications: college or university degree	Neale B	UKBB	European	2017	-0.01373	0.001138	1.64E-33	334070	106305	227765	1	risk diff	Neale-B_UKBB_EUR_2017
rs9848497	Qualifications: none	Neale B	UKBB	European	2017	0.006491	0.000923	1.99E-12	334070	57567	276503	1	risk diff	Neale-B_UKBB_EUR_2017
rs9848497	Qualifications: other professional qualifications	Neale B	UKBB	European	2017	-0.00513	0.001112	3.95E-06	334070	97524	236546	1	risk diff	Neale-B_UKBB_EUR_2017
rs9848497	Self-reported diabetes	Neale B	UKBB	European	2017	0.002247	0.000472	1.94E-06	337159	13243	323916	1	risk diff	Neale-B_UKBB_EUR_2017
rs9848497	Wheeze or whistling in the chest in last year	Neale B	UKBB	European	2017	0.00692	0.000995	3.44E-12	331257	68531	262726	1	risk diff	Neale-B_UKBB_EUR_2017
rs9848497	Years of educational attainment in females	SSGAC	27225129	European	2016	-0.023	0.003	2.52E-12	181443	0	181443	62	years	SSGAC_EduYears-F_EUR_2016
rs9848497	Years of educational attainment in males	SSGAC	27225129	European	2016	-0.021	0.004	9.19E-09	147474	0	147474	6	years	SSGAC_EduYears-M_EUR_2016
rs9848497	Years of educational attainment	SSGAC	27225129	European	2016	-0.021	0.002	1.39E-17	328917	0	328917	64	years	SSGAC_EduYears_EUR_2016
rs56038006	Qualifications: college or university degree	Neale B	UKBB	European	2017	-0.00981	0.001513	9.00E-11	334070	106305	227765	1	risk diff	Neale-B_UKBB_EUR_2017
rs56038006	Years of educational attainment in males	SSGAC	27225129	European	2016	-0.025	0.005	2.46E-07	147474	0	147474	6	years	SSGAC_EduYears-M_EUR_2016
rs56038006	Years of educational attainment	SSGAC	27225129	European	2016	-0.017	0.003	5.66E-08	328917	0	328917	64	years	SSGAC_EduYears_EUR_2016
rs2304130	Type II diabetes	DIAGRAM	26551672	European	2015	-0.1231	0.02277	5.50E-08	84780	27206	57574	23	log OR	DIAGRAM_T2D-METABO_EUR_2015
rs2304130	Type 2 diabetes	DIAGRAM	22885922	Mixed	2012	NA	NA	1.10E-97	69033	-	-	-	-	GRASP
rs2304130	Diabetes diagnosed by doctor	Neale B	UKBB	European	2017	-0.00471	0.000929	3.97E-07	336473	16183	320290	1	risk diff	Neale-B_UKBB_EUR_2017
rs2304130	Self-reported diabetes	Neale B	UKBB	European	2017	-0.00404	0.000842	1.63E-06	337159	13243	323916	1	risk diff	Neale-B_UKBB_EUR_2017
rs2304607	Diastolic blood pressure	Neale B	UKBB	European	2017	0.02104	0.003622	6.31E-09	317756	0	317756	1	IVNT	Neale-B_UKBB_EUR_2017
rs2304607	Qualifications: college or university degree	Neale B	UKBB	European	2017	0.01006	0.001676	2.00E-09	334070	106305	227765	1	risk diff	Neale-B_UKBB_EUR_2017
rs9942416	Self-reported diabetes	Neale B	UKBB	European	2017	0.002203	0.000491	7.23E-06	337159	13243	323916	1	risk diff	Neale-B_UKBB_EUR_2017
rs2397061	Elevated blood glucose level	Neale B	UKBB	European	2017	0.000389	7.37E-05	1.33E-07	337199	125	337074	1	risk diff	Neale-B_UKBB_EUR_2017
rs2516739	Diastolic blood pressure	Neale B	UKBB	European	2017	0.01444	0.002975	1.22E-06	317756	0	317756	1	IVNT	Neale-B_UKBB_EUR_2017
rs2516739	Systolic blood pressure	Neale B	UKBB	European	2017	0.0142	0.002973	1.78E-06	317754	0	317754	1	IVNT	Neale-B_UKBB_EUR_2017
rs2590942	Ever smoked	Neale B	UKBB	European	2017	0.007059	0.001481	1.86E-06	336067	202585	133482	1	risk diff	Neale-B_UKBB_EUR_2017
rs2590942	Past tobacco smoking	Neale B	UKBB	European	2017	-0.02173	0.003946	3.65E-08	310749	0	310749	1	-	Neale-B_UKBB_EUR_2017
rs2590942	Self-reported hypertension	Neale B	UKBB	European	2017	0.006167	0.001327	3.36E-06	337159	87690	249469	1	risk diff	Neale-B_UKBB_EUR_2017
rs2590942	Smoking status: previous	Neale B	UKBB	European	2017	0.007754	0.001448	8.63E-08	336024	118419	217605	1	risk diff	Neale-B_UKBB_EUR_2017
rs2590942	Vascular or heart problems diagnosed by doctor: high blood press	Neale B	UKBB	European	2017	0.005998	0.001345	8.26E-06	336683	91033	245650	1	risk diff	Neale-B_UKBB_EUR_2017
rs2817419	Diabetes diagnosed by doctor	Neale B	UKBB	European	2017	0.002799	0.000592	2.28E-06	336473	16183	320290	1	risk diff	Neale-B_UKBB_EUR_2017
rs2817419	Self-reported diabetes	Neale B	UKBB	European	2017	0.002413	0.000537	6.95E-06	337159	13243	323916	1	risk diff	Neale-B_UKBB_EUR_2017
rs273504	Diastolic blood pressure	Neale B	UKBB	European	2017	-0.01175	0.002493	2.46E-06	317756	0	317756	1	IVNT	Neale-B_UKBB_EUR_2017
rs273504	Qualifications: college or university degree	Neale B	UKBB	European	2017	0.006087	0.001154	1.32E-07	334070	106305	227765	1	risk diff	Neale-B_UKBB_EUR_2017
rs7655341	Systolic blood pressure	Neale B	UKBB	European	2017	0.0139	0.002764	4.93E-07	317754	0	317754	1	IVNT	Neale-B_UKBB_EUR_2017
rs7655341	Treatment with simvastatin	Neale B	UKBB	European	2017	0.004029	0.000865	3.18E-06	337159	38492	298667	1	risk diff	Neale-B_UKBB_EUR_2017
rs2820311	Diastolic blood pressure	Neale B	UKBB	European	2017	-0.01662	0.002602	1.67E-10	317756	0	317756	1	IVNT	Neale-B_UKBB_EUR_2017
rs2820311	Qualifications: college or university degree	Neale B	UKBB	European	2017	0.006038	0.001203	5.25E-07	334070	106305	227765	1	risk diff	Neale-B_UKBB_EUR_2017
rs2820311	Qualifications: other professional qualifications	Neale B	UKBB	European	2017	0.006058	0.001176	2.57E-07	334070	97524	236546	1	risk diff	Neale-B_UKBB_EUR_2017
rs2820311	Self-reported hypertension	Neale B	UKBB	European	2017	-0.00507	0.001126	6.68E-06	337159	87690	249469	1	risk diff	Neale-B_UKBB_EUR_2017
rs2820311	Vascular or heart problems diagnosed by doctor: high blood press	Neale B	UKBB	European	2017	-0.00505	0.001142	9.97E-06	336683	91033	245650	1	risk diff	Neale-B_UKBB_EUR_2017
rs2836961	No blood clot, bronchitis, emphysema, asthma, rhinitis, eczema o	Neale B	UKBB	European	2017	-0.00547	0.001174	3.16E-06	336782	228530	108252	1	risk diff	Neale-B_UKBB_EUR_2017
rs1041448	Qualifications: college or university degree	Neale B	UKBB	European	2017	-0.00623	0.001192	1.74E-07	334070	106305	227765	1	risk diff	Neale-B_UKBB_EUR_2017
rs1041448	Years of educational attainment	SSGAC	27225129	European	2016	-0.012	0.003	5.55E-06	328917	0	328917	64	years	SSGAC_EduYears_EUR_2016
rs2842385	Qualifications: A levels or as levels or equivalent	Neale B	UKBB	European	2017	-0.00614	0.001367	7.04E-06	334070	91710	242360	1	risk diff	Neale-B_UKBB_EUR_2017
rs2842385	Qualifications: college or university degree	Neale B	UKBB	European	2017	-0.00872	0.001426	9.66E-10	334070	106305	227765	1	risk diff	Neale-B_UKBB_EUR_2017
rs2842385	Years of educational attainment	SSGAC	27225129	European	2016	-0.016	0.003	5.09E-07	328917	0	328917	64	years	SSGAC_EduYears_EUR_2016

rs1757457	Hayfever, allergic rhinitis or eczema	Neale B	UKBB	European	2017	-0.00579	0.001238	2.93E-06	336782	77891	258891	1	risk diff	Neale-B_UKBB_EUR_2017
rs1757457	No blood clot, bronchitis, emphysema, asthma, rhinitis, eczema o	Neale B	UKBB	European	2017	0.007501	0.001372	4.55E-08	336782	228530	108252	1	risk diff	Neale-B_UKBB_EUR_2017
rs329124	Current tobacco smoking	Neale B	UKBB	European	2017	0.006439	0.001341	1.57E-06	337030	0	337030	1	-	Neale-B_UKBB_EUR_2017
rs329124	Qualifications: college or university degree	Neale B	UKBB	European	2017	-0.00605	0.001155	1.66E-07	334070	106305	227765	1	risk diff	Neale-B_UKBB_EUR_2017
rs329124	Years of educational attainment	SSGAC	27225129	European	2016	-0.012	0.002	8.81E-07	328917	0	328917	64	years	SSGAC_EduYears_EUR_2016
rs3734572	Self-reported psoriasis	Neale B	UKBB	European	2017	-0.0093	0.000635	1.69E-48	337159	3871	333288	1	risk diff	Neale-B_UKBB_EUR_2017
rs3734572	Self-reported psoriatic arthropathy	Neale B	UKBB	European	2017	-0.00148	0.000262	1.54E-08	337159	650	336509	1	risk diff	Neale-B_UKBB_EUR_2017
rs7201850	Type II diabetes	DIAGRAM	26551672	European	2015	-0.1266	0.01281	2.80E-23	84780	27206	57574	23	log OR	DIAGRAM_T2D-METABO_EUR_2015
rs7201850	Type II diabetes	DIAGRAM	22885922	European	2012	-0.1133	0.01823	7.70E-10	63390	9580	53810	12	log OR	DIAGRAM_T2D_EUR_2012
rs7201850	Type II diabetes	DIAGRAM	28566273	European	2017	-0.12	0.012	2.50E-22	159208	26676	132532	18	log OR	DIAGRAM_T2D_EUR_2017
rs7201850	Type II diabetes	DIAGRAM	24509480	Mixed	2014	-0.08618	0.01405	4.00E-10	110452	26488	83964	4	log OR	DIAGRAM_T2D_Mixed_2014
rs7201850	Diabetes diagnosed by doctor	Neale B	UKBB	European	2017	-0.00482	0.000529	7.83E-20	336473	16183	320290	1	risk diff	Neale-B_UKBB_EUR_2017
rs7201850	Illnesses of father: diabetes	Neale B	UKBB	European	2017	-0.00386	0.000768	5.07E-07	293407	28197	265210	1	risk diff	Neale-B_UKBB_EUR_2017
rs7201850	Illnesses of mother: diabetes	Neale B	UKBB	European	2017	-0.00375	0.000739	3.87E-07	309953	28875	281078	1	risk diff	Neale-B_UKBB_EUR_2017
rs7201850	Self-reported diabetes	Neale B	UKBB	European	2017	-0.00383	0.00048	1.37E-15	337159	13243	323916	1	risk diff	Neale-B_UKBB_EUR_2017
rs7201850	Self-reported hypertension	Neale B	UKBB	European	2017	-0.00752	0.001082	3.68E-12	337159	87690	249469	1	risk diff	Neale-B_UKBB_EUR_2017
rs7201850	Treatment with blood pressure medication	Neale B	UKBB	European	2017	-0.00706	0.001286	4.01E-08	180203	31488	148715	1	risk diff	Neale-B_UKBB_EUR_2017
rs7201850	Vascular or heart problems diagnosed by doctor: high blood pres	Neale B	UKBB	European	2017	-0.00741	0.001097	1.42E-11	336683	91033	245650	1	risk diff	Neale-B_UKBB_EUR_2017
rs3768486	Vascular or heart problems diagnosed by doctor: high blood pres	Neale B	UKBB	European	2017	-0.00636	0.001399	5.38E-06	336683	91033	245650	1	risk diff	Neale-B_UKBB_EUR_2017
rs3800229	Qualifications: college or university degree	Neale B	UKBB	European	2017	-0.00633	0.001266	5.81E-07	334070	106305	227765	1	risk diff	Neale-B_UKBB_EUR_2017
rs3800229	Qualifications: none	Neale B	UKBB	European	2017	0.005058	0.001026	8.28E-07	334070	57567	276503	1	risk diff	Neale-B_UKBB_EUR_2017
rs3803286	Hayfever, allergic rhinitis or eczema	Neale B	UKBB	European	2017	0.005949	0.001087	4.47E-08	336782	77891	258891	1	risk diff	Neale-B_UKBB_EUR_2017
rs3803286	No blood clot, bronchitis, emphysema, asthma, rhinitis, eczema o	Neale B	UKBB	European	2017	-0.00539	0.001205	7.63E-06	336782	228530	108252	1	risk diff	Neale-B_UKBB_EUR_2017
rs3803286	Qualifications: none	Neale B	UKBB	European	2017	-0.00467	0.000978	1.79E-06	334070	57567	276503	1	risk diff	Neale-B_UKBB_EUR_2017
rs3803286	Self-reported eczema or dermatitis	Neale B	UKBB	European	2017	0.00192	0.00041	2.77E-06	337159	8718	328441	1	risk diff	Neale-B_UKBB_EUR_2017
rs3810291	Diabetes diagnosed by doctor	Neale B	UKBB	European	2017	0.003169	0.000557	1.24E-08	336473	16183	320290	1	risk diff	Neale-B_UKBB_EUR_2017
rs3810291	Self-reported diabetes	Neale B	UKBB	European	2017	0.002298	0.000505	5.26E-06	337159	13243	323916	1	risk diff	Neale-B_UKBB_EUR_2017
rs3810291	Wheeze or whistling in the chest in last year	Neale B	UKBB	European	2017	0.005471	0.001063	2.68E-07	331257	68531	262726	1	risk diff	Neale-B_UKBB_EUR_2017
rs3814883	Self-reported hypertension	Neale B	UKBB	European	2017	0.005114	0.00107	1.75E-06	337159	87690	249469	1	risk diff	Neale-B_UKBB_EUR_2017
rs3814883	Vascular or heart problems diagnosed by doctor: high blood pres	Neale B	UKBB	European	2017	0.005311	0.001084	9.72E-07	336683	91033	245650	1	risk diff	Neale-B_UKBB_EUR_2017
rs3922853	Qualifications: A levels or as levels or equivalent	Neale B	UKBB	European	2017	0.006996	0.001438	1.14E-06	334070	91710	242360	1	risk diff	Neale-B_UKBB_EUR_2017
rs3922853	Qualifications: O levels or GCSEs or equivalent	Neale B	UKBB	European	2017	0.007139	0.001605	8.64E-06	334070	159730	174340	1	risk diff	Neale-B_UKBB_EUR_2017
rs149457	Systolic blood pressure	Neale B	UKBB	European	2017	-0.01744	0.003282	1.08E-07	317754	0	317754	1	IVNT	Neale-B_UKBB_EUR_2017
rs10496345	Years of education	SSGAC	23722424	European	2013	NA	NA	4.47E-07	101069	-	-	-	-	GRASP
rs10496345	Qualifications: A levels or as levels or equivalent	Neale B	UKBB	European	2017	0.006314	0.001115	1.48E-08	334070	91710	242360	1	risk diff	Neale-B_UKBB_EUR_2017
rs10496345	Qualifications: college or university degree	Neale B	UKBB	European	2017	0.01083	0.001163	1.27E-20	334070	106305	227765	1	risk diff	Neale-B_UKBB_EUR_2017
rs10496345	Qualifications: none	Neale B	UKBB	European	2017	-0.00482	0.000943	3.21E-07	334070	57567	276503	1	risk diff	Neale-B_UKBB_EUR_2017
rs10496345	Years of educational attainment in females	SSGAC	27225129	European	2016	0.021	0.003	6.08E-10	181443	0	181443	62	years	SSGAC_EduYears-F_EUR_2016
rs10496345	Years of educational attainment in males	SSGAC	27225129	European	2016	0.025	0.004	8.78E-11	147474	0	147474	6	years	SSGAC_EduYears-M_EUR_2016
rs10496345	Years of educational attainment	SSGAC	23722424	European	2013	0.021	0.004	4.47E-07	101069	0	101069	42	years	SSGAC_EduYears_EUR_2013
rs10496345	Years of educational attainment	SSGAC	27225129	European	2016	0.022	0.003	8.01E-18	328917	0	328917	64	years	SSGAC_EduYears_EUR_2016
rs4372296	Qualifications: college or university degree	Neale B	UKBB	European	2017	0.007896	0.001374	9.06E-09	334070	106305	227765	1	risk diff	Neale-B_UKBB_EUR_2017
rs4372296	Wheeze or whistling in the chest in last year	Neale B	UKBB	European	2017	-0.00623	0.001201	2.07E-07	331257	68531	262726	1	risk diff	Neale-B_UKBB_EUR_2017
rs4515655	Current tobacco smoking	Neale B	UKBB	European	2017	0.006197	0.001349	4.34E-06	337030	0	337030	1	-	Neale-B_UKBB_EUR_2017
rs4516268	Qualifications: college or university degree	Neale B	UKBB	European	2017	0.006659	0.001442	3.86E-06	334070	106305	227765	1	risk diff	Neale-B_UKBB_EUR_2017
rs388614	Self-reported hypertension	Neale B	UKBB	European	2017	-0.00517	0.001081	1.73E-06	337159	87690	249469	1	risk diff	Neale-B_UKBB_EUR_2017
rs4653017	Ever smoked	Neale B	UKBB	European	2017	-0.0067	0.001282	1.73E-07	336067	202585	133482	1	risk diff	Neale-B_UKBB_EUR_2017
rs4653017	Past tobacco smoking	Neale B	UKBB	European	2017	0.01777	0.003412	1.91E-07	310749	0	310749	1	-	Neale-B_UKBB_EUR_2017
rs1559625	Qualifications: college or university degree	Neale B	UKBB	European	2017	-0.0058	0.001165	6.38E-07	334070	106305	227765	1	risk diff	Neale-B_UKBB_EUR_2017
rs4744275	Qualifications: none	Neale B	UKBB	European	2017	0.004856	0.001051	3.83E-06	334070	57567	276503	1	risk diff	Neale-B_UKBB_EUR_2017
rs4759073	Qualifications: college or university degree	Neale B	UKBB	European	2017	0.005864	0.001155	3.80E-07	334070	106305	227765	1	risk diff	Neale-B_UKBB_EUR_2017
rs4759073	Qualifications: other professional qualifications	Neale B	UKBB	European	2017	0.005149	0.001128	5.01E-06	334070	97524	236546	1	risk diff	Neale-B_UKBB_EUR_2017
rs4759228	Allergic disease	Ferreira M	29083406	European	2017	-0.0379	0.0065	4.78E-09	360838	180129	180709	13	log OR	Ferreira-M_Allergic-Disease_EUR_2017
rs4759228	Asthma	Neale B	UKBB	European	2017	-0.0043	0.000854	4.66E-07	336782	38791	297991	1	risk diff	Neale-B_UKBB_EUR_2017
rs4759228	Hayfever, allergic rhinitis or eczema	Neale B	UKBB	European	2017	-0.00758	0.001126	1.72E-11	336782	77891	258891	1	risk diff	Neale-B_UKBB_EUR_2017
rs4759228	No blood clot, bronchitis, emphysema, asthma, rhinitis, eczema o	Neale B	UKBB	European	2017	0.008588	0.001248	5.95E-12	336782	228530	108252	1	risk diff	Neale-B_UKBB_EUR_2017
rs4759228	Qualifications: college or university degree	Neale B	UKBB	European	2017	-0.00553	0.001249	9.56E-06	334070	106305	227765	1	risk diff	Neale-B_UKBB_EUR_2017
rs4759228	Self-reported asthma	Neale B	UKBB	European	2017	-0.00455	0.000855	1.04E-07	337159	39049	298110	1	risk diff	Neale-B_UKBB_EUR_2017
rs4759228	Years of educational attainment	SSGAC	27225129	European	2016	-0.014	0.003	1.37E-06	328917	0	328917	64	years	SSGAC_EduYears_EUR_2016

rs4796243	Self-reported allergy or anaphylactic reaction to drug	Neale B	UKBB	European	2017	-0.00116	0.000252	4.08E-06	337159	3043	334116	1	risk diff	Neale-B_UKBB_EUR_2017
rs487060	Treatment with atorvastatin	Neale B	UKBB	European	2017	0.002086	0.000418	5.91E-07	337159	10167	326992	1	risk diff	Neale-B_UKBB_EUR_2017
rs487060	Treatment with simvastatin	Neale B	UKBB	European	2017	0.003458	0.000774	7.80E-06	337159	38492	298667	1	risk diff	Neale-B_UKBB_EUR_2017
rs11857450	Type II diabetes adjusted for BMI	DIAGRAM	28566273	European	2017	0.074	0.016	4.00E-06	159208	26676	132532	18	log OR	DIAGRAM_T2D-adjusted-BMI_EUR_2017
rs11857450	Type II diabetes	DIAGRAM	28566273	European	2017	0.078	0.014	1.40E-08	159208	26676	132532	18	log OR	DIAGRAM_T2D_EUR_2017
rs11857450	Type II diabetes	DIAGRAM	24509480	Mixed	2014	0.08618	0.01405	3.60E-11	110452	26488	83964	4	log OR	DIAGRAM_T2D_Mixed_2014
rs11857450	Diabetes diagnosed by doctor	Neale B	UKBB	European	2017	0.003093	0.000581	9.98E-08	336473	16183	320290	1	risk diff	Neale-B_UKBB_EUR_2017
rs11857450	Self-reported diabetes	Neale B	UKBB	European	2017	0.002497	0.000527	2.11E-06	337159	13243	323916	1	risk diff	Neale-B_UKBB_EUR_2017
rs4916661	Years of educational attainment	SSGAC	27225129	European	2016	0.011	0.003	7.05E-06	328917	0	328917	64	years	SSGAC_EduYears_EUR_2016
rs1569979	Qualifications: A levels or as levels or equivalent	Neale B	UKBB	European	2017	-0.00664	0.0013	3.24E-07	334070	91710	242360	1	risk diff	Neale-B_UKBB_EUR_2017
rs1569979	Qualifications: college or university degree	Neale B	UKBB	European	2017	-0.0063	0.001357	3.38E-06	334070	106305	227765	1	risk diff	Neale-B_UKBB_EUR_2017
rs1569979	Years of educational attainment	SSGAC	27225129	European	2016	-0.015	0.003	5.68E-07	328917	0	328917	64	years	SSGAC_EduYears_EUR_2016
rs5396	Current employment status: unable to work because of sickness o	Neale B	UKBB	European	2017	-0.00233	0.000517	6.22E-06	336252	12554	323698	1	risk diff	Neale-B_UKBB_EUR_2017
rs6121381	Self-reported hypertension	Neale B	UKBB	European	2017	-0.00665	0.001471	6.20E-06	337159	87690	249469	1	risk diff	Neale-B_UKBB_EUR_2017
rs6121381	Vascular or heart problems diagnosed by doctor: high blood press	Neale B	UKBB	European	2017	-0.00673	0.001491	6.33E-06	336683	91033	245650	1	risk diff	Neale-B_UKBB_EUR_2017
rs6235	3233 split proinsulin in non diabetics	MAGIC	21873549	European	2011	NA	NA	4.50E-06	10701	-	-	-	-	GRASP
rs6235	Diabetes mellitus type 2	MAGIC	21873549	European	2011	NA	NA	1.00E-26	-	-	-	-	-	dbGaP
rs11030104	Ever smoked	Neale B	UKBB	European	2017	0.00705	0.001476	1.79E-06	336067	202585	133482	1	risk diff	Neale-B_UKBB_EUR_2017
rs11030104	Past tobacco smoking	Neale B	UKBB	European	2017	-0.02319	0.003928	3.56E-09	310749	0	310749	1	-	Neale-B_UKBB_EUR_2017
rs6442101	Diastolic blood pressure	ICBP	21909115	European	2011	NA	NA	8.80E-06	69395	-	-	-	-	GRASP
rs6442101	Diastolic blood pressure	ICBP	21909115	European	2011	NA	NA	8.80E-06	69395	0	69395	29	mmHg	ICBP_DBP_EUR_2011
rs6442101	Diastolic blood pressure	Neale B	UKBB	European	2017	-0.01355	0.002638	2.79E-07	317756	0	317756	1	IVNT	Neale-B_UKBB_EUR_2017
rs6442101	Qualifications: college or university degree	Neale B	UKBB	European	2017	-0.00601	0.001221	8.65E-07	334070	106305	227765	1	risk diff	Neale-B_UKBB_EUR_2017
rs6442101	Self-reported hypertension	Neale B	UKBB	European	2017	-0.00521	0.001143	5.06E-06	337159	87690	249469	1	risk diff	Neale-B_UKBB_EUR_2017
rs6449531	Qualifications: A levels or as levels or equivalent	Neale B	UKBB	European	2017	-0.0058	0.001137	3.35E-07	334070	91710	242360	1	risk diff	Neale-B_UKBB_EUR_2017
rs6449531	Qualifications: college or university degree	Neale B	UKBB	European	2017	-0.00806	0.001186	1.04E-11	334070	106305	227765	1	risk diff	Neale-B_UKBB_EUR_2017
rs6449531	Qualifications: none	Neale B	UKBB	European	2017	0.005503	0.000961	1.04E-08	334070	57567	276503	1	risk diff	Neale-B_UKBB_EUR_2017
rs6449531	Qualifications: other professional qualifications	Neale B	UKBB	European	2017	-0.0054	0.001158	3.11E-06	334070	97524	236546	1	risk diff	Neale-B_UKBB_EUR_2017
rs6449531	Years of educational attainment	SSGAC	27225129	European	2016	-0.012	0.003	2.12E-06	328917	0	328917	64	years	SSGAC_EduYears_EUR_2016
rs663129	Type II diabetes	DIAGRAM	28566273	European	2017	-0.064	0.014	4.60E-06	159208	26676	132532	18	log OR	DIAGRAM_T2D_EUR_2017
rs663129	Type II diabetes	DIAGRAM	24509480	Mixed	2014	-0.07696	0.01418	4.50E-08	110452	26488	83964	4	log OR	DIAGRAM_T2D_Mixed_2014
rs663129	Diabetes diagnosed by doctor	Neale B	UKBB	European	2017	-0.00309	0.000614	4.71E-07	336473	16183	320290	1	risk diff	Neale-B_UKBB_EUR_2017
rs663129	Vascular or heart problems diagnosed by doctor: high blood press	Neale B	UKBB	European	2017	-0.00596	0.001274	2.94E-06	336683	91033	245650	1	risk diff	Neale-B_UKBB_EUR_2017
rs6738445	Diastolic blood pressure	Neale B	UKBB	European	2017	0.01485	0.002729	5.29E-08	317756	0	317756	1	IVNT	Neale-B_UKBB_EUR_2017
rs6818414	Tobacco smoking: smokes on most or all days	Neale B	UKBB	European	2017	0.003229	0.000728	9.26E-06	83133	1869	81264	1	risk diff	Neale-B_UKBB_EUR_2017
rs6843738	Diastolic blood pressure	Neale B	UKBB	European	2017	-0.01385	0.002622	1.29E-07	317756	0	317756	1	IVNT	Neale-B_UKBB_EUR_2017
rs6843738	Self-reported hypertension	Neale B	UKBB	European	2017	-0.0064	0.001135	1.69E-08	337159	87690	249469	1	risk diff	Neale-B_UKBB_EUR_2017
rs6843738	Treatment with blood pressure medication	Neale B	UKBB	European	2017	-0.00675	0.001349	5.70E-07	180203	31488	148715	1	risk diff	Neale-B_UKBB_EUR_2017
rs6843738	Vascular or heart problems diagnosed by doctor: high blood press	Neale B	UKBB	European	2017	-0.00652	0.001151	1.51E-08	336683	91033	245650	1	risk diff	Neale-B_UKBB_EUR_2017
rs6922855	Diastolic blood pressure	Neale B	UKBB	European	2017	0.01572	0.002475	2.12E-10	317756	0	317756	1	IVNT	Neale-B_UKBB_EUR_2017
rs6922855	Self-reported hypertension	Neale B	UKBB	European	2017	0.004962	0.001072	3.68E-06	337159	87690	249469	1	risk diff	Neale-B_UKBB_EUR_2017
rs6922855	Vascular or heart problems diagnosed by doctor: high blood press	Neale B	UKBB	European	2017	0.00533	0.001087	9.38E-07	336683	91033	245650	1	risk diff	Neale-B_UKBB_EUR_2017
rs6938239	Hayfever, allergic rhinitis or eczema	Neale B	UKBB	European	2017	0.006602	0.001478	7.90E-06	336782	77891	258891	1	risk diff	Neale-B_UKBB_EUR_2017
rs7006629	Qualifications: college or university degree	Neale B	UKBB	European	2017	0.005403	0.001139	2.10E-06	334070	106305	227765	1	risk diff	Neale-B_UKBB_EUR_2017
rs398015	Systolic blood pressure	Neale B	UKBB	European	2017	-0.01348	0.002711	6.57E-07	317754	0	317754	1	IVNT	Neale-B_UKBB_EUR_2017
rs398015	Treatment with simvastatin	Neale B	UKBB	European	2017	-0.00405	0.000848	1.81E-06	337159	38492	298667	1	risk diff	Neale-B_UKBB_EUR_2017
rs7122539	Qualifications: college or university degree	Neale B	UKBB	European	2017	-0.00553	0.001206	4.62E-06	334070	106305	227765	1	risk diff	Neale-B_UKBB_EUR_2017
rs7123876	Fasting glucose	MAGIC	22885924	European	2012	-0.011	0.0024	3.55E-06	133010	0	133010	66	mmol/l	MAGIC_FG-METABO_EUR_2012
rs7123876	Years of educational attainment	SSGAC	27225129	European	2016	-0.013	0.003	3.26E-06	328917	0	328917	64	years	SSGAC_EduYears_EUR_2016
rs7131262	Diastolic blood pressure	Neale B	UKBB	European	2017	0.01317	0.002724	1.34E-06	317756	0	317756	1	IVNT	Neale-B_UKBB_EUR_2017
rs7185232	Qualifications: college or university degree	Neale B	UKBB	European	2017	-0.00754	0.001206	4.04E-10	334070	106305	227765	1	risk diff	Neale-B_UKBB_EUR_2017
rs7217226	Self-reported hypertension	Neale B	UKBB	European	2017	0.004957	0.001115	8.77E-06	337159	87690	249469	1	risk diff	Neale-B_UKBB_EUR_2017
rs7217226	Vascular or heart problems diagnosed by doctor: high blood press	Neale B	UKBB	European	2017	0.005399	0.00113	1.79E-06	336683	91033	245650	1	risk diff	Neale-B_UKBB_EUR_2017
rs7243357	Diabetes diagnosed by doctor	Neale B	UKBB	European	2017	-0.00324	0.000687	2.32E-06	336473	16183	320290	1	risk diff	Neale-B_UKBB_EUR_2017
rs7334078	Years of education	SSGAC	23722424	European	2013	NA	NA	1.40E-06	101069	-	-	-	-	GRASP
rs7334078	Qualifications: college or university degree	Neale B	UKBB	European	2017	0.006198	0.001264	9.39E-07	334070	106305	227765	1	risk diff	Neale-B_UKBB_EUR_2017
rs7334078	Years of educational attainment	SSGAC	23722424	European	2013	0.021	0.004	1.40E-06	101069	0	101069	42	years	SSGAC_EduYears_EUR_2013
rs7334078	Years of educational attainment	SSGAC	27225129	European	2016	0.015	0.003	1.10E-07	328917	0	328917	64	years	SSGAC_EduYears_EUR_2016
rs7377083	Type II diabetes	DIAGRAM	28566273	European	2017	0.06	0.013	9.30E-06	159208	26676	132532	18	log OR	DIAGRAM_T2D_EUR_2017

rs11039798	Diastolic blood pressure	Neale B	UKBB	European	2017	-0.01728	0.003666	2.43E-06	317756	0	317756	1	IVNT	Neale-B_UKBB_EUR_2017
rs11039798	Illnesses of siblings: high blood pressure	Neale B	UKBB	European	2017	-0.00791	0.00165	1.67E-06	262361	54270	208091	1	risk diff	Neale-B_UKBB_EUR_2017
rs11039798	Self-reported hypertension	Neale B	UKBB	European	2017	-0.01202	0.001588	3.67E-14	337159	87690	249469	1	risk diff	Neale-B_UKBB_EUR_2017
rs11039798	Systolic blood pressure	Neale B	UKBB	European	2017	-0.01962	0.003664	8.57E-08	317754	0	317754	1	IVNT	Neale-B_UKBB_EUR_2017
rs11039798	Treatment with blood pressure medication	Neale B	UKBB	European	2017	-0.00843	0.001884	7.70E-06	180203	31488	148715	1	risk diff	Neale-B_UKBB_EUR_2017
rs11039798	Vascular or heart problems diagnosed by doctor: high blood pres	Neale B	UKBB	European	2017	-0.01201	0.00161	8.41E-14	336683	91033	245650	1	risk diff	Neale-B_UKBB_EUR_2017
rs7531118	Qualifications: college or university degree	Neale B	UKBB	European	2017	0.008878	0.001149	1.09E-14	334070	106305	227765	1	risk diff	Neale-B_UKBB_EUR_2017
rs7531118	Qualifications: none	Neale B	UKBB	European	2017	-0.00434	0.000931	3.13E-06	334070	57567	276503	1	risk diff	Neale-B_UKBB_EUR_2017
rs7531118	Qualifications: other professional qualifications	Neale B	UKBB	European	2017	0.005436	0.001122	1.27E-06	334070	97524	236546	1	risk diff	Neale-B_UKBB_EUR_2017
rs7531118	Vascular or heart problems diagnosed by doctor: high blood pres	Neale B	UKBB	European	2017	0.005382	0.00109	7.86E-07	336683	91033	245650	1	risk diff	Neale-B_UKBB_EUR_2017
rs7531118	Years of educational attainment	SSGAC	27225129	European	2016	0.012	0.003	1.84E-06	328917	0	328917	64	years	SSGAC_EduYears_EUR_2016
rs115590225	Diastolic blood pressure	Neale B	UKBB	European	2017	0.02306	0.004818	1.70E-06	317756	0	317756	1	IVNT	Neale-B_UKBB_EUR_2017
rs9905385	Systolic blood pressure	Neale B	UKBB	European	2017	-0.01301	0.002624	7.17E-07	317754	0	317754	1	IVNT	Neale-B_UKBB_EUR_2017
rs7607369	Qualifications: A levels or as levels or equivalent	Neale B	UKBB	European	2017	-0.00508	0.001101	3.89E-06	334070	91710	242360	1	risk diff	Neale-B_UKBB_EUR_2017
rs769674	Current employment status: unable to work because of sickness (	Neale B	UKBB	European	2017	-0.00247	0.000493	5.58E-07	336252	12554	323698	1	risk diff	Neale-B_UKBB_EUR_2017
rs769674	Years of educational attainment	SSGAC	27225129	European	2016	0.014	0.003	2.38E-07	328917	0	328917	64	years	SSGAC_EduYears_EUR_2016
rs7711753	Diastolic blood pressure	Neale B	UKBB	European	2017	0.01636	0.002483	4.42E-11	317756	0	317756	1	IVNT	Neale-B_UKBB_EUR_2017
rs7965658	Systolic blood pressure	Neale B	UKBB	European	2017	-0.0154	0.003114	7.58E-07	317754	0	317754	1	IVNT	Neale-B_UKBB_EUR_2017
rs799449	Years of educational attainment	SSGAC	27225129	European	2016	0.011	0.002	4.69E-06	328917	0	328917	64	years	SSGAC_EduYears_EUR_2016
rs8047395	Type II diabetes	DIAGRAM	26551672	European	2015	0.1213	0.01266	8.10E-22	84780	27206	57574	23	log OR	DIAGRAM_T2D-METABO_EUR_2015
rs8047395	Type II diabetes	DIAGRAM	22885922	European	2012	0.1044	0.01839	7.30E-08	63390	9580	53810	12	log OR	DIAGRAM_T2D_EUR_2012
rs8047395	Type II diabetes	DIAGRAM	28566273	European	2017	0.1	0.012	3.20E-16	159208	26676	132532	18	log OR	DIAGRAM_T2D_EUR_2017
rs8047395	Type II diabetes	DIAGRAM	24509480	Mixed	2014	0.07696	0.01418	1.80E-07	110452	26488	83964	4	log OR	DIAGRAM_T2D_Mixed_2014
rs8047395	Diabetes diagnosed by doctor	Neale B	UKBB	European	2017	0.004025	0.000522	1.25E-14	336473	16183	320290	1	risk diff	Neale-B_UKBB_EUR_2017
rs8047395	Illnesses of father: diabetes	Neale B	UKBB	European	2017	0.003551	0.000758	2.81E-06	293407	28197	265210	1	risk diff	Neale-B_UKBB_EUR_2017
rs8047395	Illnesses of siblings: high blood pressure	Neale B	UKBB	European	2017	0.005355	0.001109	1.38E-06	262361	54270	208091	1	risk diff	Neale-B_UKBB_EUR_2017
rs8047395	Self-reported diabetes	Neale B	UKBB	European	2017	0.003339	0.000473	1.73E-12	337159	13243	323916	1	risk diff	Neale-B_UKBB_EUR_2017
rs8047395	Self-reported hypertension	Neale B	UKBB	European	2017	0.006877	0.001068	1.21E-10	337159	87690	249469	1	risk diff	Neale-B_UKBB_EUR_2017
rs8047395	Systolic blood pressure	Neale B	UKBB	European	2017	0.01092	0.002466	9.59E-06	317754	0	317754	1	IVNT	Neale-B_UKBB_EUR_2017
rs8047395	Treatment with blood pressure medication	Neale B	UKBB	European	2017	0.006135	0.001267	1.27E-06	180203	31488	148715	1	risk diff	Neale-B_UKBB_EUR_2017
rs8047395	Vascular or heart problems diagnosed by doctor: high blood pres	Neale B	UKBB	European	2017	0.00677	0.001083	4.06E-10	336683	91033	245650	1	risk diff	Neale-B_UKBB_EUR_2017
rs8070454	Allergic disease	Ferreira M	29083406	European	2017	-0.0388	0.0058	2.97E-11	360838	180129	180709	13	log OR	Ferreira-M_Allergic-Disease_EUR_2017
rs8070454	Asthma	GABRIEL	20860503	European	2010	-0.1054	0.02037	2.28E-07	26475	10365	16110	24	log OR	GABRIEL_Asthma_EUR_2010
rs8070454	Asthma	GABRIEL	20860503	European	2010	NA	NA	2.28E-07	26475	-	-	-	-	GRASP
rs8070454	Asthma childhood and later onset	GABRIEL	20860503	European	2010	NA	NA	1.99E-06	26475	-	-	-	-	GRASP
rs8070454	Asthma childhood later and unknown onset and severe and indus	GABRIEL	20860503	European	2010	NA	NA	2.28E-07	26475	-	-	-	-	GRASP
rs8070454	Asthma childhood later and unknown onset and severe asthma	GABRIEL	20860503	European	2010	NA	NA	3.29E-07	26475	-	-	-	-	GRASP
rs8070454	Asthma childhood onset	GABRIEL	20860503	European	2010	NA	NA	6.60E-08	26475	-	-	-	-	GRASP
rs8070454	Asthma	Neale B	UKBB	European	2017	-0.00627	0.000798	4.03E-15	336782	38791	297991	1	risk diff	Neale-B_UKBB_EUR_2017
rs8070454	Doctor diagnosed asthma	Neale B	UKBB	European	2017	-0.008	0.001672	1.73E-06	83529	10589	72940	1	risk diff	Neale-B_UKBB_EUR_2017
rs8070454	Hayfever, allergic rhinitis or eczema	Neale B	UKBB	European	2017	-0.0055	0.001053	1.76E-07	336782	77891	258891	1	risk diff	Neale-B_UKBB_EUR_2017
rs8070454	No blood clot, bronchitis, emphysema, asthma, rhinitis, eczema o	Neale B	UKBB	European	2017	0.008389	0.001167	6.47E-13	336782	228530	108252	1	risk diff	Neale-B_UKBB_EUR_2017
rs8070454	Self-reported asthma	Neale B	UKBB	European	2017	-0.00647	0.0008	5.96E-16	337159	39049	298110	1	risk diff	Neale-B_UKBB_EUR_2017
rs8070454	Wheeze or whistling in the chest in last year	Neale B	UKBB	European	2017	-0.00494	0.00102	1.28E-06	331257	68531	262726	1	risk diff	Neale-B_UKBB_EUR_2017
rs8070454	Asthma	TAGC	29273806	European	2018	-0.1057	0.01273	1.04E-16	127669	19954	107715	56	log OR	TAGC_Asthma_EUR_2018
rs8070454	Asthma	TAGC	29273806	Mixed	2018	-0.09901	0.01191	9.29E-17	142486	23948	118538	66	log OR	TAGC_Asthma_Mixed_2018
rs8075273	Qualifications: college or university degree	Neale B	UKBB	European	2017	0.005734	0.001276	7.02E-06	334070	106305	227765	1	risk diff	Neale-B_UKBB_EUR_2017
rs8097544	Self-reported hypertension	Neale B	UKBB	European	2017	-0.00705	0.001518	3.39E-06	337159	87690	249469	1	risk diff	Neale-B_UKBB_EUR_2017
rs8097544	Vascular or heart problems diagnosed by doctor: high blood pres	Neale B	UKBB	European	2017	-0.00705	0.001538	4.65E-06	336683	91033	245650	1	risk diff	Neale-B_UKBB_EUR_2017
rs8102137	Diastolic blood pressure	Neale B	UKBB	European	2017	0.02299	0.002618	1.61E-18	317756	0	317756	1	IVNT	Neale-B_UKBB_EUR_2017
rs8102137	Self-reported hypertension	Neale B	UKBB	European	2017	0.005083	0.001134	7.34E-06	337159	87690	249469	1	risk diff	Neale-B_UKBB_EUR_2017
rs8102137	Vascular or heart problems diagnosed by doctor: high blood pres	Neale B	UKBB	European	2017	0.005486	0.001149	1.81E-06	336683	91033	245650	1	risk diff	Neale-B_UKBB_EUR_2017
rs820077	Asthma	Neale B	UKBB	European	2017	0.004737	0.000973	1.13E-06	336782	38791	297991	1	risk diff	Neale-B_UKBB_EUR_2017
rs820077	Self-reported asthma	Neale B	UKBB	European	2017	0.004942	0.000975	3.99E-07	337159	39049	298110	1	risk diff	Neale-B_UKBB_EUR_2017
rs853679	Asthma	Neale B	UKBB	European	2017	-0.00494	0.001048	2.47E-06	336782	38791	297991	1	risk diff	Neale-B_UKBB_EUR_2017
rs853679	Diabetes diagnosed by doctor	Neale B	UKBB	European	2017	-0.00317	0.000702	6.41E-06	336473	16183	320290	1	risk diff	Neale-B_UKBB_EUR_2017
rs853679	Diastolic blood pressure	Neale B	UKBB	European	2017	0.01864	0.003316	1.92E-08	317756	0	317756	1	IVNT	Neale-B_UKBB_EUR_2017
rs853679	Self-reported asthma	Neale B	UKBB	European	2017	-0.00469	0.00105	7.96E-06	337159	39049	298110	1	risk diff	Neale-B_UKBB_EUR_2017
rs853679	Treatment with insulin	Neale B	UKBB	European	2017	-0.00188	0.000391	1.48E-06	180203	1377	178826	1	risk diff	Neale-B_UKBB_EUR_2017

rs853679	Treatment with insulin product	Neale B	UKBB	European	2017	-0.00234	0.000324	4.99E-13	337159	3319	333840	1	risk diff	Neale-B_UKBB_EUR_2017
rs8567	Vascular or heart problems diagnosed by doctor: high blood pres	Neale B	UKBB	European	2017	-0.00481	0.001083	8.85E-06	336683	91033	245650	1	risk diff	Neale-B_UKBB_EUR_2017
rs879620	Diastolic blood pressure	Neale B	UKBB	European	2017	0.01407	0.002539	2.99E-08	317756	0	317756	1	IVNT	Neale-B_UKBB_EUR_2017
rs889398	Diabetes diagnosed by doctor	Neale B	UKBB	European	2017	-0.00301	0.000531	1.45E-08	336473	16183	320290	1	risk diff	Neale-B_UKBB_EUR_2017
rs889398	Past tobacco smoking	Neale B	UKBB	European	2017	0.01623	0.003228	4.98E-07	310749	0	310749	1	-	Neale-B_UKBB_EUR_2017
rs889398	Self-reported diabetes	Neale B	UKBB	European	2017	-0.00261	0.000482	5.76E-08	337159	13243	323916	1	risk diff	Neale-B_UKBB_EUR_2017
rs900144	Diastolic blood pressure	Neale B	UKBB	European	2017	0.01386	0.002484	2.41E-08	317756	0	317756	1	IVNT	Neale-B_UKBB_EUR_2017
rs900144	Self-reported hypertension	Neale B	UKBB	European	2017	0.005249	0.001075	1.05E-06	337159	87690	249469	1	risk diff	Neale-B_UKBB_EUR_2017
rs900144	Systolic blood pressure	Neale B	UKBB	European	2017	0.01241	0.002483	5.78E-07	317754	0	317754	1	IVNT	Neale-B_UKBB_EUR_2017
rs900144	Vascular or heart problems diagnosed by doctor: high blood pres	Neale B	UKBB	European	2017	0.005017	0.00109	4.19E-06	336683	91033	245650	1	risk diff	Neale-B_UKBB_EUR_2017
rs13194250	Diastolic blood pressure	Neale B	UKBB	European	2017	0.01313	0.002482	1.23E-07	317756	0	317756	1	IVNT	Neale-B_UKBB_EUR_2017
rs13194250	Exposure to tobacco smoke outside home	Neale B	UKBB	European	2017	0.007214	0.001494	1.37E-06	286550	0	286550	1	-	Neale-B_UKBB_EUR_2017
rs13194250	Qualifications: A levels or as levels or equivalent	Neale B	UKBB	European	2017	-0.00803	0.001101	2.93E-13	334070	91710	242360	1	risk diff	Neale-B_UKBB_EUR_2017
rs13194250	Qualifications: CSEs or equivalent	Neale B	UKBB	European	2017	0.005261	0.000837	3.31E-10	334070	44362	289708	1	risk diff	Neale-B_UKBB_EUR_2017
rs13194250	Qualifications: O levels or GCSEs or equivalent	Neale B	UKBB	European	2017	-0.00546	0.001229	8.97E-06	334070	159730	174340	1	risk diff	Neale-B_UKBB_EUR_2017
rs13194250	Qualifications: college or university degree	Neale B	UKBB	European	2017	-0.01217	0.001148	3.01E-26	334070	106305	227765	1	risk diff	Neale-B_UKBB_EUR_2017
rs13194250	Qualifications: none	Neale B	UKBB	European	2017	0.006648	0.000931	9.34E-13	334070	57567	276503	1	risk diff	Neale-B_UKBB_EUR_2017
rs13194250	Years of educational attainment in females	SSGAC	27225129	European	2016	-0.019	0.003	8.16E-09	181443	0	181443	62	years	SSGAC_EduYears-F_EUR_2016
rs13194250	Years of educational attainment in males	SSGAC	27225129	European	2016	-0.022	0.004	4.23E-09	147474	0	147474	6	years	SSGAC_EduYears-M_EUR_2016
rs13194250	Years of educational attainment	SSGAC	27225129	European	2016	-0.02	0.002	2.77E-15	328917	0	328917	64	years	SSGAC_EduYears_EUR_2016
rs903959	Qualifications: college or university degree	Neale B	UKBB	European	2017	-0.00782	0.001164	1.86E-11	334070	106305	227765	1	risk diff	Neale-B_UKBB_EUR_2017
rs903959	Years of educational attainment in females	SSGAC	27225129	European	2016	-0.021	0.003	3.57E-10	181443	0	181443	62	years	SSGAC_EduYears-F_EUR_2016
rs903959	Years of educational attainment	SSGAC	27225129	European	2016	-0.016	0.003	3.35E-10	328917	0	328917	64	years	SSGAC_EduYears_EUR_2016
rs905938	Hayfever, allergic rhinitis or eczema	Neale B	UKBB	European	2017	0.005435	0.001162	2.88E-06	336782	77891	258891	1	risk diff	Neale-B_UKBB_EUR_2017
rs919433	Hayfever, allergic rhinitis or eczema	Neale B	UKBB	European	2017	-0.00644	0.001071	1.83E-09	336782	77891	258891	1	risk diff	Neale-B_UKBB_EUR_2017
rs9460306	Qualifications: college or university degree	Neale B	UKBB	European	2017	0.01038	0.00204	3.60E-07	334070	106305	227765	1	risk diff	Neale-B_UKBB_EUR_2017
rs9527706	Qualifications: A levels or as levels or equivalent	Neale B	UKBB	European	2017	0.007632	0.001227	4.91E-10	334070	91710	242360	1	risk diff	Neale-B_UKBB_EUR_2017
rs9527706	Qualifications: college or university degree	Neale B	UKBB	European	2017	0.01092	0.001279	1.42E-17	334070	106305	227765	1	risk diff	Neale-B_UKBB_EUR_2017
rs9527706	Qualifications: none	Neale B	UKBB	European	2017	-0.0071	0.001037	7.65E-12	334070	57567	276503	1	risk diff	Neale-B_UKBB_EUR_2017
rs9527706	Years of educational attainment in females	SSGAC	27225129	European	2016	0.026	0.004	3.11E-12	181443	0	181443	62	years	SSGAC_EduYears-F_EUR_2016
rs9527706	Years of educational attainment in males	SSGAC	27225129	European	2016	0.02	0.004	1.01E-06	147474	0	147474	6	years	SSGAC_EduYears-M_EUR_2016
rs9527706	Years of educational attainment	SSGAC	27225129	European	2016	0.022	0.003	1.18E-15	328917	0	328917	64	years	SSGAC_EduYears_EUR_2016
rs954018	Systolic blood pressure	Neale B	UKBB	European	2017	0.01641	0.002685	9.75E-10	317754	0	317754	1	IVNT	Neale-B_UKBB_EUR_2017
rs73455661	Diabetes diagnosed by doctor	Neale B	UKBB	European	2017	-0.00284	0.00058	9.66E-07	336473	16183	320290	1	risk diff	Neale-B_UKBB_EUR_2017
rs10954284	Type II diabetes	DIAGRAM	22885922	European	2012	-0.09531	0.01617	1.20E-08	69033	12171	56862	12	log OR	DIAGRAM_T2D_EUR_2012
rs10954284	Diabetes diagnosed by doctor	Neale B	UKBB	European	2017	-0.00241	0.000521	3.81E-06	336473	16183	320290	1	risk diff	Neale-B_UKBB_EUR_2017
rs10954284	Self-reported hypertension	Neale B	UKBB	European	2017	-0.00661	0.001066	5.52E-10	337159	87690	249469	1	risk diff	Neale-B_UKBB_EUR_2017
rs10954284	Treatment with blood pressure medication	Neale B	UKBB	European	2017	-0.00879	0.001266	3.98E-12	180203	31488	148715	1	risk diff	Neale-B_UKBB_EUR_2017
rs10954284	Treatment with simvastatin	Neale B	UKBB	European	2017	-0.00355	0.000771	4.18E-06	337159	38492	298667	1	risk diff	Neale-B_UKBB_EUR_2017
rs10954284	Vascular or heart problems diagnosed by doctor: high blood pres	Neale B	UKBB	European	2017	-0.00671	0.001081	5.49E-10	336683	91033	245650	1	risk diff	Neale-B_UKBB_EUR_2017
rs987237	Diabetes diagnosed by doctor	Neale B	UKBB	European	2017	-0.0037	0.000681	5.67E-08	336473	16183	320290	1	risk diff	Neale-B_UKBB_EUR_2017
rs987237	Self-reported diabetes	Neale B	UKBB	European	2017	-0.00313	0.000617	4.04E-07	337159	13243	323916	1	risk diff	Neale-B_UKBB_EUR_2017
rs9931494	Type II diabetes	DIAGRAM	26551672	European	2015	-0.1275	0.0128	1.70E-23	84780	27206	57574	23	log OR	DIAGRAM_T2D-METABO_EUR_2015
rs9931494	Type II diabetes	DIAGRAM	22885922	European	2012	-0.1133	0.01823	6.80E-10	63390	9580	53810	12	log OR	DIAGRAM_T2D_EUR_2012
rs9931494	Type II diabetes	DIAGRAM	28566273	European	2017	-0.12	0.012	1.00E-22	159208	26676	132532	18	log OR	DIAGRAM_T2D_EUR_2017
rs9931494	Type II diabetes	DIAGRAM	24509480	Mixed	2014	-0.08618	0.01165	3.70E-11	110452	26488	83964	4	log OR	DIAGRAM_T2D_Mixed_2014
rs9931494	Diabetes diagnosed by doctor	Neale B	UKBB	European	2017	-0.00486	0.000529	4.33E-20	336473	16183	320290	1	risk diff	Neale-B_UKBB_EUR_2017
rs9931494	Illnesses of father: diabetes	Neale B	UKBB	European	2017	-0.00389	0.000769	4.32E-07	293407	28197	265210	1	risk diff	Neale-B_UKBB_EUR_2017
rs9931494	Illnesses of mother: diabetes	Neale B	UKBB	European	2017	-0.00373	0.00074	4.58E-07	309953	28875	281078	1	risk diff	Neale-B_UKBB_EUR_2017
rs9931494	Illnesses of siblings: high blood pressure	Neale B	UKBB	European	2017	-0.00498	0.001124	9.31E-06	262361	54270	208091	1	risk diff	Neale-B_UKBB_EUR_2017
rs9931494	Self-reported diabetes	Neale B	UKBB	European	2017	-0.00386	0.00048	8.34E-16	337159	13243	323916	1	risk diff	Neale-B_UKBB_EUR_2017
rs9931494	Self-reported hypertension	Neale B	UKBB	European	2017	-0.00758	0.001083	2.54E-12	337159	87690	249469	1	risk diff	Neale-B_UKBB_EUR_2017
rs9931494	Treatment with blood pressure medication	Neale B	UKBB	European	2017	-0.00714	0.001286	2.85E-08	180203	31488	148715	1	risk diff	Neale-B_UKBB_EUR_2017
rs9931494	Treatment with gliclazide	Neale B	UKBB	European	2017	-0.001	0.000225	8.46E-06	337159	2807	334352	1	risk diff	Neale-B_UKBB_EUR_2017
rs9931494	Vascular or heart problems diagnosed by doctor: high blood pres	Neale B	UKBB	European	2017	-0.00747	0.001098	1.00E-11	336683	91033	245650	1	risk diff	Neale-B_UKBB_EUR_2017
rs998584	Diastolic blood pressure	BPExome	27618447	Mixed	2016	NA	NA	3.87E-06	192763	0	192763	51	INVT	BPExome_DBP_Mixed_2016
rs998584	Self-reported hypertension	Neale B	UKBB	European	2017	-0.00474	0.001069	9.17E-06	337159	87690	249469	1	risk diff	Neale-B_UKBB_EUR_2017
rs998584	Treatment with simvastatin	Neale B	UKBB	European	2017	-0.0036	0.000773	3.10E-06	337159	38492	298667	1	risk diff	Neale-B_UKBB_EUR_2017
rs998584	Vascular or heart problems diagnosed by doctor: high blood pres	Neale B	UKBB	European	2017	-0.00501	0.001084	3.72E-06	336683	91033	245650	1	risk diff	Neale-B_UKBB_EUR_2017

rs12146443	Type II diabetes	DIAGRAM	26551672	European	2015	-0.07511	0.01301	5.50E-09	84780	27206	57574	23	log OR	DIAGRAM_T2D-METABO_EUR_2015
rs12146443	Type II diabetes adjusted for BMI	DIAGRAM	28566273	European	2017	-0.074	0.015	5.30E-07	159208	26676	132532	18	log OR	DIAGRAM_T2D-adjusted-BMI_EUR_2017
rs12146443	Type II diabetes	DIAGRAM	28566273	European	2017	-0.061	0.012	7.90E-07	159208	26676	132532	18	log OR	DIAGRAM_T2D_EUR_2017
rs12146443	Diabetes diagnosed by doctor	Neale B	UKBB	European	2017	-0.00319	0.000536	2.61E-09	336473	16183	320290	1	risk diff	Neale-B_UKBB_EUR_2017
rs12146443	Forced expiratory volume in 1-second, predicted	Neale B	UKBB	European	2017	-0.01264	0.002801	6.44E-06	110423	0	110423	1	IVNT	Neale-B_UKBB_EUR_2017
rs12146443	Self-reported diabetes	Neale B	UKBB	European	2017	-0.00306	0.000486	2.98E-10	337159	13243	323916	1	risk diff	Neale-B_UKBB_EUR_2017
rs12146443	Systolic blood pressure	Neale B	UKBB	European	2017	-0.01199	0.002531	2.17E-06	317754	0	317754	1	IVNT	Neale-B_UKBB_EUR_2017
rs12146443	Treatment with metformin	Neale B	UKBB	European	2017	-0.00191	0.00039	9.38E-07	337159	8392	328767	1	risk diff	Neale-B_UKBB_EUR_2017
rs13001104	Ever smoked	Neale B	UKBB	European	2017	-0.00578	0.001198	1.41E-06	336067	202585	133482	1	risk diff	Neale-B_UKBB_EUR_2017
rs13270194	Diastolic blood pressure	Neale B	UKBB	European	2017	0.01616	0.002463	5.35E-11	317756	0	317756	1	IVNT	Neale-B_UKBB_EUR_2017
rs13270194	Forced expiratory volume in 1-second	Neale B	UKBB	European	2017	-0.0096	0.002082	3.96E-06	307638	0	307638	1	IVNT	Neale-B_UKBB_EUR_2017
rs13270194	Peak expiratory flow	Neale B	UKBB	European	2017	-0.01137	0.002163	1.48E-07	307638	0	307638	1	IVNT	Neale-B_UKBB_EUR_2017
rs13270194	Self-reported hypertension	Neale B	UKBB	European	2017	0.005967	0.001067	2.22E-08	337159	87690	249469	1	risk diff	Neale-B_UKBB_EUR_2017
rs13270194	Systolic blood pressure	Neale B	UKBB	European	2017	0.01458	0.002462	3.20E-09	317754	0	317754	1	IVNT	Neale-B_UKBB_EUR_2017
rs13270194	Treatment with bendroflumethiazide	Neale B	UKBB	European	2017	0.00279	0.000564	7.41E-07	337159	19084	318075	1	risk diff	Neale-B_UKBB_EUR_2017
rs13270194	Treatment with blood pressure medication	Neale B	UKBB	European	2017	0.006249	0.001267	8.24E-07	180203	31488	148715	1	risk diff	Neale-B_UKBB_EUR_2017
rs1609906	Ever smoked	Neale B	UKBB	European	2017	0.005531	0.001189	3.26E-06	336067	202585	133482	1	risk diff	Neale-B_UKBB_EUR_2017
rs1609906	Past tobacco smoking	Neale B	UKBB	European	2017	-0.01772	0.003166	2.20E-08	310749	0	310749	1	-	Neale-B_UKBB_EUR_2017
rs1609906	Smoking status: previous	Neale B	UKBB	European	2017	0.005484	0.001163	2.40E-06	336024	118419	217605	1	risk diff	Neale-B_UKBB_EUR_2017
rs34320273	Systolic blood pressure	Neale B	UKBB	European	2017	0.01431	0.003123	4.59E-06	317754	0	317754	1	IVNT	Neale-B_UKBB_EUR_2017
rs35267732	Psoriasis	Neale B	UKBB	European	2017	0.000923	0.000156	3.58E-09	337199	342	336857	1	risk diff	Neale-B_UKBB_EUR_2017
rs35267732	Self-reported psoriasis	Neale B	UKBB	European	2017	0.002527	0.000524	1.39E-06	337159	3871	333288	1	risk diff	Neale-B_UKBB_EUR_2017
rs35267732	Self-reported psoriatic arthropathy	Neale B	UKBB	European	2017	0.001386	0.000216	1.30E-10	337159	650	336509	1	risk diff	Neale-B_UKBB_EUR_2017
rs4766502	Diastolic blood pressure	Neale B	UKBB	European	2017	-0.02344	0.002918	9.47E-16	317756	0	317756	1	IVNT	Neale-B_UKBB_EUR_2017
rs4766502	Medication for cholesterol, blood pressure or diabetes: blood pre	Neale B	UKBB	European	2017	-0.00824	0.001852	8.64E-06	154702	38548	116154	1	risk diff	Neale-B_UKBB_EUR_2017
rs4766502	Self-reported hypertension	Neale B	UKBB	European	2017	-0.00702	0.001264	2.86E-08	337159	87690	249469	1	risk diff	Neale-B_UKBB_EUR_2017
rs4766502	Systolic blood pressure	Neale B	UKBB	European	2017	-0.0185	0.002917	2.28E-10	317754	0	317754	1	IVNT	Neale-B_UKBB_EUR_2017
rs55797265	Self-reported psoriasis	Neale B	UKBB	European	2017	0.00249	0.000435	1.01E-08	337159	3871	333288	1	risk diff	Neale-B_UKBB_EUR_2017
rs62395851	Hayfever, allergic rhinitis or eczema	Neale B	UKBB	European	2017	0.009446	0.001921	8.84E-07	336782	77891	258891	1	risk diff	Neale-B_UKBB_EUR_2017
rs62395851	Medication for cholesterol, blood pressure or diabetes: insulin	Neale B	UKBB	European	2017	-0.00393	0.000782	5.04E-07	154702	2116	152586	1	risk diff	Neale-B_UKBB_EUR_2017
rs62395851	No blood clot, bronchitis, emphysema, asthma, rhinitis, eczema o	Neale B	UKBB	European	2017	-0.00971	0.002129	5.09E-06	336782	228530	108252	1	risk diff	Neale-B_UKBB_EUR_2017
rs62395851	Self-reported psoriasis	Neale B	UKBB	European	2017	-0.00253	0.000486	1.93E-07	337159	3871	333288	1	risk diff	Neale-B_UKBB_EUR_2017
rs62395851	Treatment with insulin product	Neale B	UKBB	European	2017	-0.00241	0.00045	8.74E-08	337159	3319	333840	1	risk diff	Neale-B_UKBB_EUR_2017
rs6577585	Medication for cholesterol, blood pressure or diabetes: blood pre	Neale B	UKBB	European	2017	0.009035	0.001646	4.07E-08	154702	38548	116154	1	risk diff	Neale-B_UKBB_EUR_2017
rs6577585	Medication for cholesterol, blood pressure or diabetes: cholester	Neale B	UKBB	European	2017	0.007332	0.001606	4.97E-06	154702	35840	118862	1	risk diff	Neale-B_UKBB_EUR_2017
rs6577585	Medication for cholesterol, blood pressure or diabetes: none of t	Neale B	UKBB	European	2017	-0.00866	0.001795	1.43E-06	154702	103004	51698	1	risk diff	Neale-B_UKBB_EUR_2017
rs6577585	Self-reported hypertension	Neale B	UKBB	European	2017	0.005049	0.001126	7.33E-06	337159	87690	249469	1	risk diff	Neale-B_UKBB_EUR_2017
rs67081976	Self-reported hypertension	Neale B	UKBB	European	2017	0.006714	0.001354	7.08E-07	337159	87690	249469	1	risk diff	Neale-B_UKBB_EUR_2017
rs7033236	Forced expiratory volume in 1-second	Neale B	UKBB	European	2017	0.01028	0.002093	9.01E-07	307638	0	307638	1	IVNT	Neale-B_UKBB_EUR_2017
rs7033236	Forced vital capacity	Neale B	UKBB	European	2017	0.01418	0.001984	8.84E-13	307638	0	307638	1	IVNT	Neale-B_UKBB_EUR_2017
rs7033236	Forced vital capacity, best measure	Neale B	UKBB	European	2017	0.01352	0.002177	5.27E-10	255492	0	255492	1	IVNT	Neale-B_UKBB_EUR_2017
rs7974266	Diastolic blood pressure	Neale B	UKBB	European	2017	-0.01807	0.002681	1.61E-11	317756	0	317756	1	IVNT	Neale-B_UKBB_EUR_2017
rs7974266	Systolic blood pressure	Neale B	UKBB	European	2017	-0.01689	0.00268	2.95E-10	317754	0	317754	1	IVNT	Neale-B_UKBB_EUR_2017
rs8092496	Forced expiratory volume in 1-second, predicted	Neale B	UKBB	European	2017	0.01521	0.00315	1.39E-06	110423	0	110423	1	IVNT	Neale-B_UKBB_EUR_2017
rs8092496	Forced expiratory volume in 1-second, predicted percentage	Neale B	UKBB	European	2017	-0.02474	0.00492	4.94E-07	110423	0	110423	1	IVNT	Neale-B_UKBB_EUR_2017
rs860262	Type II diabetes	DIAGRAM	26551672	European	2015	-0.08434	0.01266	3.30E-11	84780	27206	57574	23	log OR	DIAGRAM_T2D-METABO_EUR_2015
rs860262	Type II diabetes adjusted for BMI	DIAGRAM	28566273	European	2017	-0.098	0.015	1.60E-11	159208	26676	132532	18	log OR	DIAGRAM_T2D-adjusted-BMI_EUR_2017
rs860262	Type II diabetes	DIAGRAM	28566273	European	2017	-0.091	0.012	5.30E-14	159208	26676	132532	18	log OR	DIAGRAM_T2D_EUR_2017
rs860262	Asthma	Neale B	UKBB	European	2017	-0.00441	0.000778	1.48E-08	336782	38791	297991	1	risk diff	Neale-B_UKBB_EUR_2017
rs860262	Diabetes diagnosed by doctor	Neale B	UKBB	European	2017	-0.0041	0.000521	3.36E-15	336473	16183	320290	1	risk diff	Neale-B_UKBB_EUR_2017
rs860262	Forced expiratory volume in 1-second, predicted	Neale B	UKBB	European	2017	-0.01537	0.002727	1.76E-08	110423	0	110423	1	IVNT	Neale-B_UKBB_EUR_2017
rs860262	Forced expiratory volume in 1-second, predicted percentage	Neale B	UKBB	European	2017	0.0189	0.004259	9.06E-06	110423	0	110423	1	IVNT	Neale-B_UKBB_EUR_2017
rs860262	Hayfever, allergic rhinitis or eczema	Neale B	UKBB	European	2017	-0.00613	0.001026	2.38E-09	336782	77891	258891	1	risk diff	Neale-B_UKBB_EUR_2017
rs860262	No blood clot, bronchitis, emphysema, asthma, rhinitis, eczema o	Neale B	UKBB	European	2017	0.007423	0.001137	6.70E-11	336782	228530	108252	1	risk diff	Neale-B_UKBB_EUR_2017
rs860262	Self-reported asthma	Neale B	UKBB	European	2017	-0.00436	0.000779	2.26E-08	337159	39049	298110	1	risk diff	Neale-B_UKBB_EUR_2017
rs860262	Self-reported diabetes	Neale B	UKBB	European	2017	-0.00369	0.000472	5.62E-15	337159	13243	323916	1	risk diff	Neale-B_UKBB_EUR_2017
rs860262	Treatment with metformin	Neale B	UKBB	European	2017	-0.00253	0.000379	2.53E-11	337159	8392	328767	1	risk diff	Neale-B_UKBB_EUR_2017
rs9468156	Diastolic blood pressure	Neale B	UKBB	European	2017	0.01423	0.003184	7.85E-06	317756	0	317756	1	IVNT	Neale-B_UKBB_EUR_2017
rs9939726	Self-reported hypertension	Neale B	UKBB	European	2017	-0.00474	0.001065	8.45E-06	337159	87690	249469	1	risk diff	Neale-B_UKBB_EUR_2017

rs9939726	Systolic blood pressure	Neale B	UKBB	European	2017	-0.01099	0.002458	7.78E-06	317754	0	317754	1	IVNT	Neale-B_UKBB_EUR_2017
