# Supplementary material for: Investigating causal relationships between obesity and skin barrier function in a multi-ethnic Asian general population cohort
Source: Int J Obes (Lond). 2023 Jul 21;47(10):963–9. doi: 10.1038/s41366-023-01343-z (PMC10511308; doi:10.1038/s41366-023-01343-z)
Supplement: Supplementary file 8 — Supplementary Table 7 [file 41366_2023_1343_MOESM8_ESM.docx]

**Supplementary Table 7. Summary results of other MR methods for Third-party One-sample Mendelian Randomization for effect of potential exposures on TEWL**

| **MR Method** | **Beta [95% CI]** | | **P value** | |  |  |
| --- | --- | --- | --- | --- | --- | --- |
| *Education (Number of years education completed): % change in TEWL per unit (years) increase in education* | | | | | |  |
| Weighted Median Method | | -2.45E-01 [-9.22E-01, 4.33E-01] | | 0.479 | |  |
| MR Egger (Random Effects) | | -4.75E-01 [-1.14, 1.94E-01] | | 0.164 | |  |
| Egger Intercept | | 1.25E-02 [-6.58E-02, 9.08E-02] | | 0.754 | |  |
| *Monthly household income: % change in TEWL per unit (income tiers) increase in monthly household income* | | | | | |  |
| Weighted Median Method | | 1.82E-01 [-4.69, 5.06] | | 0.942 | |  |
| MR Egger (Random Effects) | | 9.97E-01 [-4.09, 6.08] | | 0.702 | |  |
| Egger Intercept | | -6.56E-02 [-4.24E-01, 2.92E-01] | | 0.721 | |  |
| *Diabetes mellitus (DM): % change in TEWL between those with DM and those without* | | | | | |  |
| Weighted Median Method | | 1.30 [-11.9, 14.5] | | 0.847 | | |
| MR Egger (Random Effects) | | -3.08 [-14.7, 8.57] | | 0.605 | | |
| Egger Intercept | | 4.63E-02 [-9.17E-02, 1.84E-01] | | 0.511 | | |
| *HbA1c: % change in TEWL per unit (%) increase in HbA1c* | | | | | | |
| Weighted Median Method | | 2.83 [-1.18, 6.85] | | 0.167 | | |
| MR Egger (Random Effects) | | 2.87 [-4.53, 6.20] | | 0.091 | | |
| Egger Intercept | | -8.49E-02 [-2.37E-01, 6.76E-02] | | 0.276 | | |
| *Insulin Resistance (HOMA-IR): % change in TEWL per unit (%) increase in HOMA-IR* | | | | | | |
| Weighted Median Method | | -4.80E-01 [-4.17E02, 4.16E02] | | 0.998 | | |
| MR Egger (Random Effects) | | 6.18E-01 [-10.3, 11.6] | | 0.915 | | |
| Egger Intercept | | -4.49E-01 [-1.49, 5.97E-01] | | 0.432 | | |
| *Systolic BP (SBP): % change in TEWL per unit (mmHg) increase in SBP* | | | | | |  |
| Weighted Median Method | | 2.19E-01 [-6.29E-03, 4.45E-01] | | 0.056 | | |
| MR Egger (Random Effects) | | 8.71E-02 [-1.33E-01, 3.07E-01] | | 0.438 | | |
| Egger Intercept | | -2.60E-04 [-1.50E-01, 1.49E-01] | | 0.997 | | |

**Table 3. *(continued)***

| \| **MR Method** \| **Beta [95% CI]** \| **P value** \| \| --- \| --- \| --- \| | | | | | | | | | | | | | | | | | |
| --- | --- | --- | --- | --- | --- | --- | --- | --- | --- | --- | --- | --- | --- | --- | --- | --- | --- | --- | --- | --- |
| *Diastolic BP (DBP): % change in TEWL per unit (mmHg) increase in DBP* | | | | | | | | | | | | | | | | | |
| Weighted Median Method | | | | 1.72E-01 [-7.20E-01, 1.06] | | | 0.705 | | | | | | | | | | |
| MR Egger (Random Effects) | | | | 4.05E-01 [-5.07E-01, 1.32] | | | 0.388 | | | | | | | | | | |
| Egger Intercept | | | | 3.72E-02 [-3.40E-01, 4.14E-01] | | | 0.848 | | | | | | | | | | |
| *Pulse Pressure (PP): % change in TEWL per unit (mmHg) increase in PP* | | | | | | | | | | | |  |  |  |  |  |  |
| Weighted Median Method | 1.53E-03 [-3.75E-01, 3.78E-01] | | | | | | | | 0.994 | | | | |  |  |  |  |
| MR Egger (Random Effects) | 1.73E-01 [-2.06E-01, 5.52E-01] | | | | | | | | 0.373 | | | | |  |  |  |  |
| Egger Intercept | -4.33E-02 [-2.12E-01, 1.26E-01] | | | | | | | | 0.616 | | | | |  |  |  |  |
| *Hypertension: % change in TEWL between those with and without hypertension* | | | | | | | | | | | | | | | |  |  |
| Weighted Median Method | | | -4.82E-02 [-5.22E-01, 4.25E-01] | | | | | 0.842 | | | | | |  |  |  |  |
| MR Egger (Random Effects) | | | 1.12 [-2.74E-01, 2.51] | | | | | 0.138 | | | | | |  |  |  |  |
| Egger Intercept | | | -1.25 [-2.74, 2.38E-01] | | | | | 0.122 | | | | | |  |  |  |  |
| *Heart rate: % change in TEWL per unit increase in Heart rate* | | | | | | | | | | | |  |  |  |  |  |  |
| Weighted Median Method | | | | | 1.61E-01 [-6.17E-01, 9.39E-01] | | | | | | 0.685 |  |  |  |  |  |  |
| MR Egger (Random Effects) | | | | | 1.27E-01 [-7.13E-01, 9.66E-01] | | | | | | 0.768 |  |  |  |  |  |  |
| Egger Intercept | | | | | 2.15E-02 [-3.21E-01, 3.64E-01] | | | | | | 0.903 |  |  |  |  |  |  |
| C-reactive protein (CRP): % change in TEWL per unit (mg/dL) increase in CRP | | | | | | | | | | | | |  |  |  |  |  |
| Weighted Median Method | | 6.01E-01 [-3.33E-01, 1.53] | | | | | | | | 0.208 | | |  |  |  |  |  |
| MR Egger (Random Effect) | | 9.88E-02 [-7.98E-01, 9.95E-01] | | | | | | | | 0.829 | | |  |  |  |  |  |
| Egger Intercept | | -5.23E-02 [-2.15E-01, 1.10E-01] | | | | | | | | 0.529 | | |  |  |  |  |  |
| *Total White Count: % change in TEWL per unit (x10^9^) increase in Total White Count* | | | | | | | | | | | | | | |  |  |  |
| Weighted Median Method | | --4.75E-02 [-2.07, 1.97] | | | | 0.963 | | | | | | | | | | |  |
| MR Egger (Random Effect) | | -3.28E-01 [-2.52, 1.87] | | | | 0.769 | | | | | | | | | | |  |
| Egger Intercept | | 6.63E-02 [-6.85E-02, 2.01E-01] | | | | 0.336 | | | | | | | | | | |  |

**Table 3. *(continued)***

| **MR Method** | | **Beta [95% CI]** | | **P value** | | | |
| --- | --- | --- | --- | --- | --- | --- | --- |
| *Neutrophil Count: % change in TEWL per unit (x 10^9^) increase in Neutrophil Count* | | | | | |  |  |
| Weighted Median Method | -4.71E-01 [-3.75, 2.81] | | 0.778 | | | |  |
| MR Egger (Random Effect) | -3.28E-01 [-3.79, 3.14] | | 0.853 | | | |  |
| Egger Intercept | 5.94E-02 [-1.02E-01, 2.21E-01] | | 0.472 | | | |  |
| Vitamin D: % change in *TEWL* per unit (ng/ml) increase in Vitamin D | | | | | |  |  |
| Weighted Median Method | -1.03E-01 [-3.63E-01, 1.58E-01] | | | | 0.440 |  |  |
| MR Egger (Random Effect) | -3.82-02 [-2.58E-01, 1.82E-01] | | | | 0.294 |  |  |
| Egger Intercept | 9.54E-02 [-1.17E-01, 3.08E-01] | | | | 0.381 |  |  |
| *Atopic dermatitis (AD): % change in TEWL between those with AD and those without* | | | | | |  |  |
| Weighted Median Method | 1.71E-01 [-3.95E-03, 7.37] | | 0.554 | | |  |  |
| MR Egger (Random Effect) | 4.36E-01 [-3.55E-03, 1.23] | | 0.294 | | |  |  |
| Egger Intercept | -2.43E-01 [-9.27E-03, 4.42E-01] | | 0.496 | | |  |  |
